# A pyridoxal phosphate-dependent enzyme platform for stereoselective synthesis of γ-tertiary-nitro-noncanonical amino acids

**DOI:** 10.1038/s41467-026-76813-9

**Published:** 2026-08-19

**Authors:** Wenping Ding, Shenggan Luo, Yike Zou, Zhijun Tang, Wen Liu

**Affiliations:** 1 State Key Laboratory of Microbial Metabolism, School of Life Science & Biotechnology, Shanghai Jiao Tong University, Shanghai, China; 2 State Key Laboratory of Chemical Biology, Shanghai Institute of Organic Chemistry, University of Chinese Academy of Sciences, Shanghai, China; 3 School of Pharmaceutical Sciences, Shanghai Jiao Tong University, Shanghai, China; 4 Zhangjiang Institute for Advanced Study, Shanghai Jiao Tong University, Shanghai, China

**Keywords:** Enzymes, Biocatalysis, Enzyme mechanisms

## Abstract

Non-canonical amino acids (ncAAs) are privileged building blocks for the synthesis of natural products, biocatalysts, and drug molecules. Among them, γ-tertiary-nitro-α-amino acids are highly valuable yet remain scarcely explored due to challenges in synthetic accessibility. Although pyridoxal-5′-phosphate (PLP)-dependent enzymes are powerful biocatalysts for ncAA production, no enzymatic platform has, to the best of our knowledge, enabled efficient access to γ-tertiary-nitro-α-amino acids. Here, we report a PLP-dependent enzymatic platform that selectively couples *O*-acetyl-serine with secondary nitroalkanes to yield γ-tertiary-nitro-α-amino acids. Through reaction design, enzyme screening, and directed evolution, we repurpose SidM, an enzyme that catalyzes pyrrole ring formation in the siderochelin biosynthetic pathway, into a highly active and stereoselective biocatalyst, affording γ-tertiary-nitro-α-amino acids in up to 99% yield and 99% diastereomeric excess. This enzyme exhibits broad substrate scope and good evolvability, allowing enhancement of catalytic efficiency or inversion of diastereoselectivity at the Cγ nitro center, and is readily scalable to gram synthesis. Mechanistic analyses combining computation and mutagenesis elucidate the structural determinants of activity and stereocontrol. This work establishes a general, tunable and scalable biocatalytic platform for the synthesis of γ-tertiary-nitro-α-amino acids, expanding the synthetic repertoire of PLP-dependent enzymes for asymmetric C–C bond formation.

## Abstract (web summary)

γ-Tertiary-nitro-α-amino acids are valuable non-canonical amino acids that remain scarcely explored due to challenges in synthetic accessibility. Here, the authors report a pyridoxal-5′-phosphate (PLP)- dependent enzymatic platform that selectively couples O-acetyl-serine and secondary nitroalkanes to yield γ-tertiary-nitro-α-amino acids.

## Introduction

**Fig. 1:**
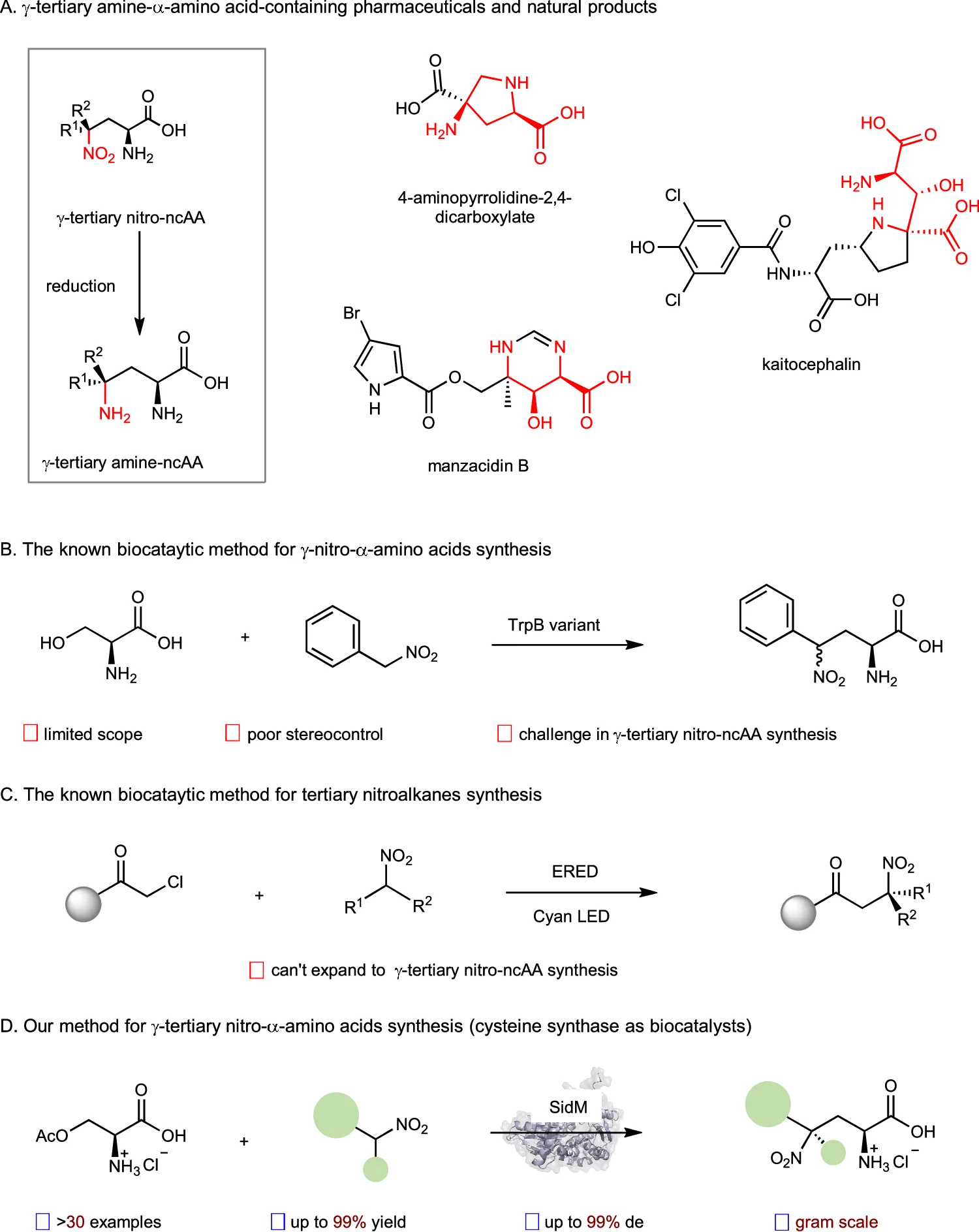
Biocatalytic strategies for the synthesis of γ-tertiary nitro-α-amino acid. **A** Representative pharmaceuticals and natural products containing chiral α,γ-diamino acid. **B** Known biocatalytic method to γ-nitro-α-amino acid^32^. **C** Established biocatalytic method for the synthesis of tertiary nitroalkanes^33^. **D** Our method for the synthesis of γ-tertiary nitro-α-amino acid.

Non-canonical amino acids (ncAAs) have become indispensable molecular tools in modern bioscience, offering structural and functional diversity far beyond the 20 proteinogenic amino acids. Their incorporation into peptides and proteins enables precise modulation of molecular properties, thereby driving advances in chemical biological, therapeutics, and biocatalysis^1–5^. More than 100 FDA-approved drugs feature at least one ncAA moiety, highlighting their profound impact on pharmaceutical chemistry^3^. Among these, ncAAs bearing tertiary stereocenters represent a particularly valuable yet underexplored subclass. A notable example is γ-tertiary-nitro-α-amino acids, which serve as key intermediates for the synthesis of advanced chiral α, γ-diamino acids—structural motifs found in bioactive natural products such as manzacidin B^6,7^, kaitocephalin^8^, as well as in drug molecules including the mGluR2 agonist (*2R,4R*)*-*4-aminopyrrolidine-2,4-dicarboxylate (*2R,4R-*APDC) (Fig. 1A)^9^. However, efficient access to such molecules remains highly challenging owing to the intrinsic difficulty of constructing quaternary stereocenters within amino acid scaffolds. Conventional chemical routes generally require multistep protecting-group manipulations and are often limited by poor stereocontrol and low overall efficiency^10–12^.

Biocatalysis provides a sustainable and powerful alternative for the stereoselective construction of chiral molecules under mild conditions^13–21^. Recent studies have enabled enzymatic access to a range of α-, β-, and γ-functionalized ncAAs^22–26^, including certain tertiary derivatives^27–31^. Yet, a general strategy for γ-tertiary-nitro-α-amino acids remains elusive. Recently, Arnold and co-workers engineered the pyridoxal 5′-phosphate (PLP)-dependent tryptophan synthase β-subunit (TrpB) to couple serine with nitroalkanes, forming γ-nitro-α-amino acids (Fig. 1B)^32^. However, this enzyme shows poor reactivity toward secondary nitroalkanes and limited stereocontrol at the resulting γ-nitro center. Hyster and co-workers developed a photoenzymatic *C*-alkylation strategy to construct tertiary nitroalkanes with high stereoselectivity (Fig. 1C)^33^, but this system is incompatible with amino acid substrates, precluding access to γ-tertiary-nitro-amino acids. These limitations highlight the need for new biocatalytic platforms that integrate broad substrate tolerance with precise stereochemical control to enable the construction of γ-tertiary-nitro-amino acids.

PLP-dependent enzymes represent a privileged class of biocatalysts for asymmetric transformations of amino acids^34–37^. Their ability to mediate diverse C–C bond formations and cleavages makes them especially attractive for ncAAs synthesis^30,38–40^, and has enabled the exploration of a wide range of biomimetic asymmetric transformations^41–43^. Nevertheless, current efforts have focused primarily on a small subset of well-characterized PLP enzymes, such as threonine aldolase (TA)^44–50^, TrpB^32,39,51–54^, and UstD^27,28,38,55,56^. While these enzymes hold significant potential, their substrate scope and reaction types remain limited. Given the vast and largely untapped sequence diversity of the PLP enzyme superfamily, there is a strong impetus to discover new enzymatic platforms with expanded capabilities. Motivated by the catalytic versatility of PLP-dependent enzymes and the potential for nitroalkane-based conjugate addition, we envisioned that an enzyme scaffold capable of efficiently generating electrophilic amino-acrylate intermediates and accommodating sterically demanding secondary nitroalkanes could serve as a biocatalyst for constructing γ-tertiary-nitro-α-amino acids from simple α-amino acid precursors.

Here, we report a PLP-dependent enzymatic platform for the stereoselective synthesis of γ-tertiary-nitro-α-amino acids. Guide by reaction design and enzyme screening, we identified SidM, a PLP-dependent enzyme from the siderochelin biosynthetic pathway, as an efficient and stereoselective catalyst that couples *O*-acetyl-serine with secondary nitroalkanes to afford γ-tertiary-nitro-α-amino acids. Subsequent reaction optimization and directed evolution yielded a robust and generalizable system, affording γ-tertiary-nitro-α-amino acids in high yield (up to 99%) and excellent diastereoselectivity (up to 99% de). Mechanistic analysis combining computational modeling and site-directed mutagenesis elucidated the catalytic basis and origin of stereoselectivity. This work establishes a general and scalable strategy for accessing chiral ncAAs, expanding the enzymatic toolbox for asymmetric C–C bond formation and offering new opportunities in peptide engineering and drug discovery.

## Results and discussion

### Enzyme screening and reaction condition optimization

We selected the coupling of *O*-acetyl-serine (**1**) and 1-nitroethylbenzene (**2a**) to produce **3a** as a model reaction, as the resulting γ-tertiary nitro center lacks an acidic proton and thus avoids the racemization typically seen with primary and secondary nitroalkanes. We focused our investigation on the PLP-dependent cysteine synthase family as potential biocatalysts, given their mechanistic suitability and structural diversity. Their native substrate **1** contains an acetyl leaving group superior to the hydroxyl group of serine, which is expected to facilitate efficient aminoacrylate intermediate formation and subsequent nitroalkane addition. This enzyme family is known to accept a broad spectrum of nucleophiles, including sulfur, oxygen, nitrogen, and even carbon species, and has been shown to catalyze non-native transformations^57^, making it a promising source for new catalytic reactivities.

**Fig. 2:**
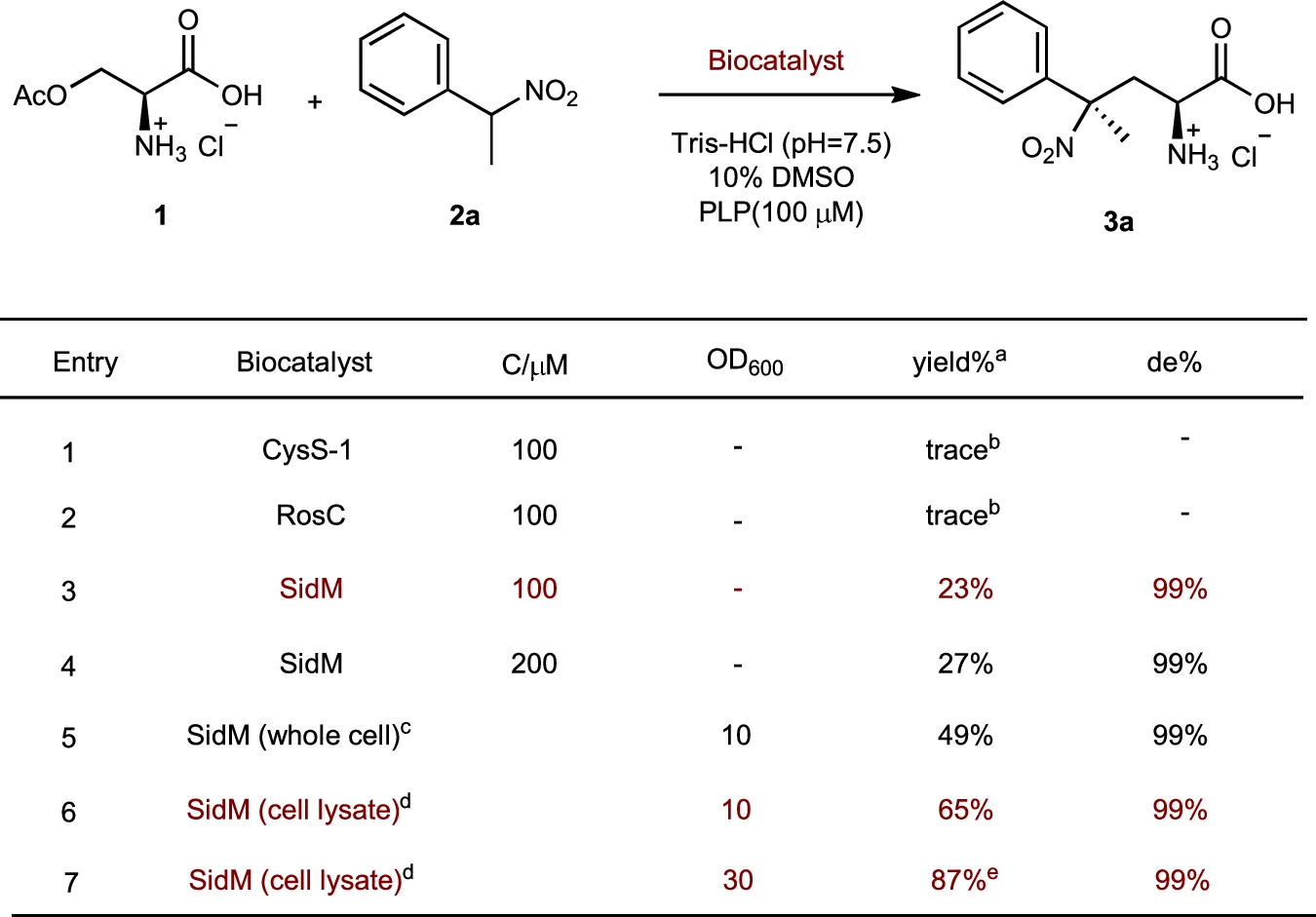
Discovery and optimization of biocatalytic synthesis of γ-tertiary nitro-α-amino acid. Reaction was conducted in 100 μL mixtures containing 100 mM Tris-HCl (pH 7.5) buffer, 50 mM NaCl, 6 mM **1**, 2 mM **2a**, 100 µM PLP, 10% (or 2%) DMSO and cysteine synthase biocatalysts from different sources. Reaction mixtures were shaken at 350 rpm at 30 °C for 12 h. ^a^Yield determined via the standard curves for product **3a** at 280 nm. ^b^Trace means that the product **3a** is detectable at the LC-MS level. ^c^Frozen cell of *E. coli* harboring SidM. ^d^Cell lysate of *E. coli* harboring SidM. ^e^2% DMSO as co-solvant, and isolated yield was reported.

An in-house panel of cysteine synthase homologs, sourced either from natural product biosynthetic pathways in our laboratory or retrieved from NCBI database, was selected for initial screening. Each enzyme was heterologously expressed in *Escherichia coli*, purified, and incubated with **1** and **2a**, with product formation monitored by high-performance liquid chromatography (HPLC). Most enzymes showed no or only trace activity under the assay conditions. However, SidM, a PLP-dependent enzyme natively involved in pyrrole ring formation in the siderochelin biosynthetic pathway^58^, displayed notable activity, converting **2a** to the desired product **3a** in 23% yield with >99:1 dr (Fig. 2, Table S1). The product **3a** contains two stereocenters at the Cα and Cγ positions, allowing for up to four possible stereoisomers. Given that PLP-dependent enzymes typically retain the Cα stereochemistry of amino acid substrates, we hypothesized that the observed dr value reflects the stereoselectivity at the newly formed Cγ center. To verify this, **2a** was replaced with primary nitroalkane **4**, yielding γ-nitro-α-amino acid **5**, which upon acid hydrolysis afforded a single enantiomer of the corresponding γ-keto acid **6**, supporting above hypothesis (Fig. S3). Scaling up the reaction between **1** and **2a** enabled isolated and further structural characterization of **3a**. NMR spectroscopy confirmed the exclusive formation of one diastereomer, consistent with HPLC analysis. Single-crystal X-ray diffraction further unambiguously established **3a** as (2*S*, 4*R*)-2-amino-4-nitro-4-phenylpentanoic acid (Fig. 2). These results indicate that SidM catalyzes the formation of **3a** with excellent stereoselectivity at the Cγ position.

Encouraged by the initial activity and selectivity, we next sought to optimize the reaction conditions (Fig. 2). Increasing the concentration of purified enzyme yield only slight improvements (from 23 to 27%) (Fig. S5). In contrast, using whole-cell or crude lysate significantly enhanced product formation, affording **3a** in 49% and 65% yield, respectively (Fig. 2). While the exact mechanism remains unclear, the improved performance likely reflects enhanced enzyme solubility in the cellular environment, as reactions using purified enzyme fused to a *N*-terminal TrX solubility tag show higher yields compared with those lacking such a tag (Fig. S5). The higher efficiency of crude lysate compared to whole cells may arise from better substrate accessibility, as supported by the narrowing yield gap between the two systems at higher cell densities (Fig. S6). We further examined the influence of pH on reaction efficiency (Fig. S7), reasoning that higher pH would enhance the nucleophilicity of nitroalkane substrates and thereby improve their conversion. Consistent with this hypothesis, the yields increased significantly between pH 7.5 and 8.0. Accordingly, pH 7.5 was selected as the optimal condition. Furthermore, 2% DMSO was introduced to improve the aqueous solubility of nitroalkane substrate **2a**, which further enhanced the yield of **3a** (Figs. S8–S9). Overall, under optimized conditions (OD_600_ = 30, Tris-HCl buffer pH = 7.5, 2% DMSO at 30 °C for 5 h), product **3a** was obtained in up to 87% isolate yield with up to 99% de. Notably, increasing the concentration of **2a** from 2 mM to 5 mM had minimal impact on yield and stereoselectivity (Fig. S10). Even at 20 mM substrate loading, product **3a** was obtained with maintained high stereoselectivity (99% de), albeit with a reduced yield (36%), highlighting the application potential of this system (Fig. 5).

### Substrate scope

Next, we explored the substrate scope of this biocatalytic transformation across of a panel of structurally diverse secondary nitroalkanes. All reactions were performed on preparative scales using *E. coli* cell lysates expressing SidM or its variant. The resulting γ-tertiary-nitro-α-amino acids were isolated and fully characterized by NMR spectroscopy and specific optical rotation.

**Fig. 3:**
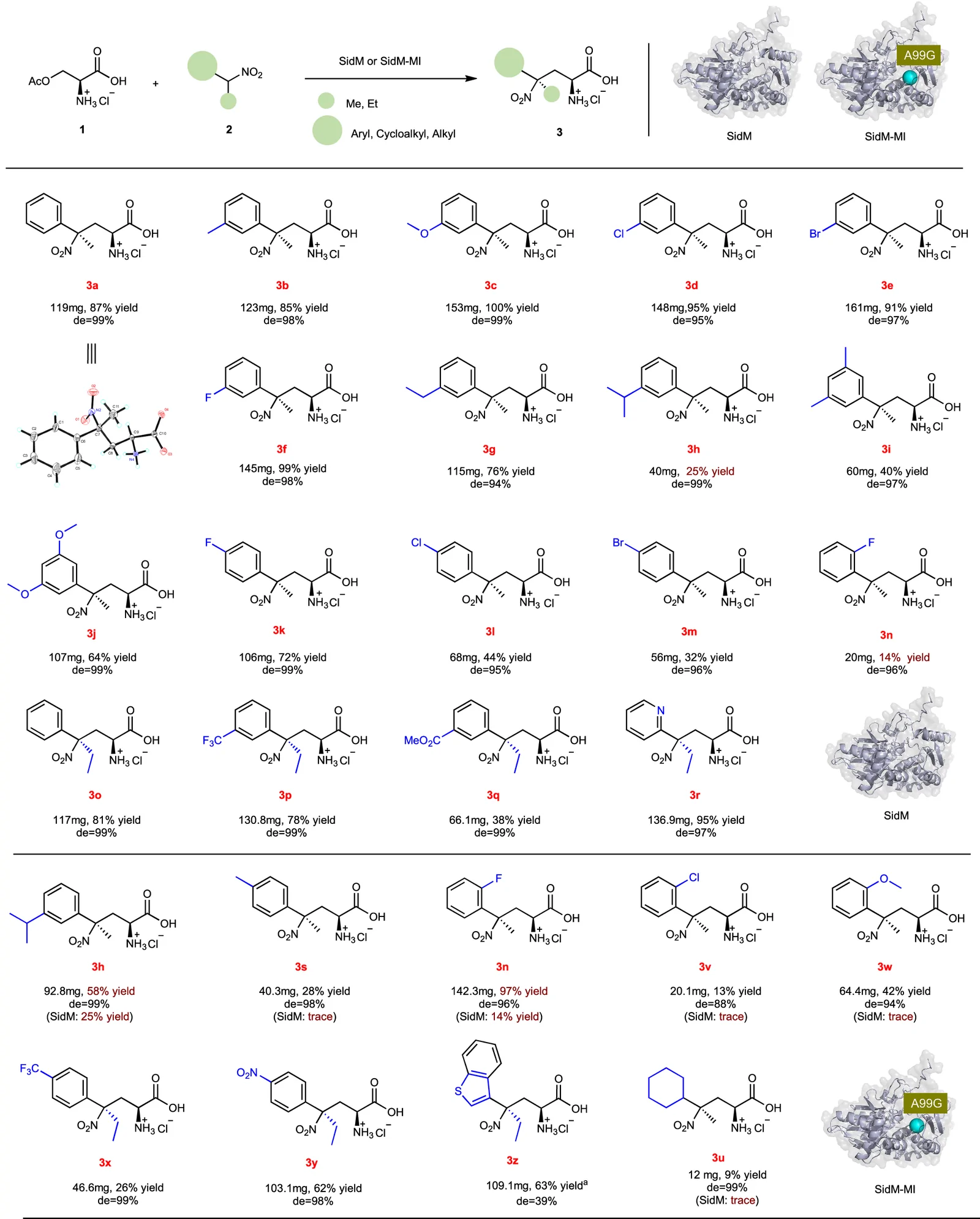
Substrate scope. Reaction was conducted at 30 °C in mixtures containing 100 mM Tris-HCl (pH 7.5) buffer, 50 mM NaCl, 9 mM **1** (1.8 equiv), 5 mM **2** (1 equiv), 100 µM PLP, 2% DMSO, and *E. coli* cell lysate expressing SidM or SidM-MI (OD_600_ = 30). The reaction was performed on a 0.5 mmol scale, and isolated yield are reported. ^a^For substrate **2z**, the reaction was carried out with 2.5 mM **2z** (1 equiv) and 4.5 mM **1** (1.8 equiv).

As depicted in Fig. 3, SidM displayed broad reactivity toward a range of 1-aryl-1-nitroalkane substrates. *Meta*-substituted aryl derivatives (**2b** ~ **2j**) were efficiently converted to their corresponding products (**3b** ~ **3j**) with high yields (up to 99%) and excellent stereoselectivity (94–99% de). Substrates bearing less sterically hindered substituents, such as methyl (**2b**), methoxy (**2c**)) or halogens (**2d**–**2f**), were particularly well tolerated, while bulkier *meta*-substituents (e.g., ethyl (**3g**), isopropyl (**3h**), 5-dimethyl (**3i**) and 3,5-dimethoxy (**3j**)) led to modestly reduced yields (25–76%) but retained high stereoselectivity. Similarly, *para*-halogen-substituted aryl nitroalkanes (**2k**–**2m**) were also well accepted with only slight diminished efficiencies. Beyond aryl ring substitution, SidM tolerated variations at the nitroalkane side chain, converting substrates with extended alkyl chains (i.e., **2o**–**2r**) to the corresponding γ-tertiary-nitro-ethyl-α-amino acids in good to excellent yields and excellent stereoselectivity (99% de). Moreover, heteroaryl substrates were also accepted, for example, 2-(1-nitropropyl)-pyridine (**2r**) was smoothly transformed into **3r** in 95% yield and 97% de. Although substituent electronic effects were less apparent in the final yield under the prolonged reaction conditions used for substrate scope evaluation, they influenced the reaction kinetics. For example, the fluorine-substituted substrate **2f** exhibited a 3-fold higher *k*_cat_ value than **2a** (Figs. S14 and S16), consistent with the electron-withdrawing fluorine substituent enhancing benzylic nucleophilicity and accelerating the reaction. In contrast, the pyridine-substituted substrate **2r** showed a reaction rate comparable to that of **2a**, despite the electron-withdrawing nature of the pyridine ring (Figs. S15 and S16), which may arise from additional interactions between pyridine nitrogen and residues in the enzyme active site. Notably, SidM showed poor activity toward *para*-alkylated and most *ortho*-substituted substrates. Only the minimally hindering *ortho*-fluoro derivative (**2n**) giving product **3n** in 14% yield, indicating that steric encumbrance near the reaction center strongly impedes catalysis. Aliphatic nitroalkanes such as 2-nitropropyl-benzene (**2t**) and 1-nitroethyl-cyclohexane (**2u**) were essentially unreactive, likely due to their reduced nucleophilicity of the nitro centers.

To expand the substrate scope and synthetic potential, we performed structure-guided protein engineering based on AlphaFold3-predicated model and molecular docking. 7 residues located within 5 Å of the docked transition state **3a-TS3** (see mechanistic studies) were subjected to site-saturation mutagenesis (Fig. S13). This effort led to identification of the SidM-MI, which containing a single A99G mutation, exhibited markedly enhanced activity and substrate tolerance (Fig. 3). Using the template substrate **2a**, SidM-MI displayed a 6.3-fold increase in *k*_cat_ (1.76 × 10^−2^ ± 6.1 × 10^−4^ s^−1^) relative to wild-type SidM (2.76 × 10^−3^ ± 7.4 × 10^−5^ s^−1^), accompanied by a 1.6-fold increase in *k*_cat_/*K*_*m*_ (2.07 M^−1^ s^−1^ vs. 1.28 M^−1^ s^−1^) (Figs. S16 and S17).

SidM-MI could efficiently convert challenging substrates. For example, *ortho*-fluoro (**2n**) and *meta*-isopropyl (**2h**) analogues, improving yields from 14 to 97% and 25 to 58%, respectively, while maintaining excellent diastereoselectivity (99% de). Beyond aryl-substituted nitroalkanes, SidM-MI also retained high activity toward side-chain-modified nitroalkanes (**2x** and **2y**), affording the corresponding γ-tertiary-nitro-ethyl-α-amino acids in 26% ~ 62% yield with up to 99% de. Moreover, SidM-MI accepted heteroaryl substrates such as benzo[*b*]thiophene derivative **2z**, producing **3z** in 63% yield, albeit with reduced diastereoselectivity (39% de). Notably, SidM-MI enabled transformations of substrates completely unreactive toward the wild-type enzyme, including *ortho*-substituted (**2v** and **2w**) and *para*-methyl (**2s**) analogues, affording the corresponding products **3v**–**3s** in mediate to good yields with excellent stereoselectivity. It also unlocked reactivity toward aliphatic nitroalkanes (**2t** and **2u**), giving **3t** (74% de, 23% yield) and **3u** (99% de, 9% yield), respectively. These results establish SidM could serve as a versatile biocatalytic platform for the stereoselective construction of diverse γ-tertiary-nitro-α-amino acids from secondary nitroalkane substrates.

### Evolvable stereoselectivity

**Fig. 4:**
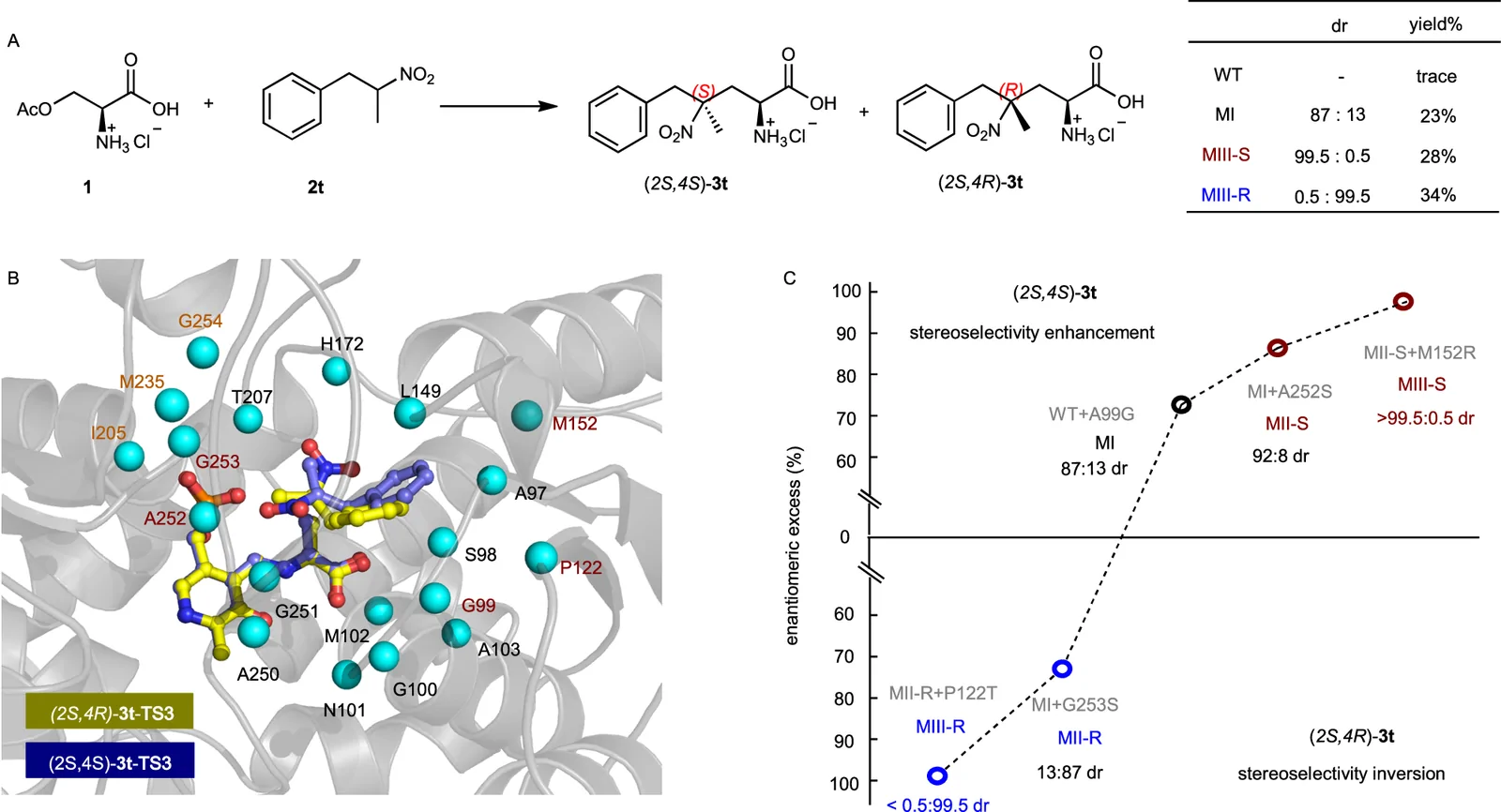
Directed evolution of SidM enabling enhancement or inversion of stereoselectivity toward 3t formation. **A** Reaction profile. **B** Docking of (*2S,4R*)- and (*2S,4S*)-**3t-TS3** into the AlphaFold3-predicated structure of SidM-MI. Residues subjected to site-saturation mutagenesis and screening are highlighted in green and shown as spheres. The (*2S,4R*)- and (*2S,4S*)-**3t-TS3** are colored in yellow and purple, respectively, and displayed as sticks. **C** Representative variants identified during the directed evolution of SidM-MI. For the experimental details, see Tables S3–S6.

Encouraged by the broad substrate tolerance of SidM, we next investigated whether its stereochemical preference could be fine-tuned through directed evolution. The coupling of **2t** with **1**, which afforded **3t** with 74% de in the SidM-MI-catalyzed reaction, was chosen as a model. Starting from SidM-MI, 18 residues located within 5 Å of the docked transition states corresponding to the (2*S*,4*S*)*-* and (2*S*,4*R*)-**3t** isomers (see mechanistic studies), were subjected to site-saturation mutagenesis. First-round screening identified five beneficial positions (M152, I205, M235, A252 and G254), among which the A252S variant (SidM-MII-S) exhibited the most pronounced improvement, producing (2*S*,4*S*)-**3t** in 27% yield and enhancing Cγ stereoselectivity from 87:13 to 92:8 dr (Fig. 4). Building on this variant, substitution of M152 with larger residues, including Gln, Arg and Tyr, progressively improved Cγ control, with M152R achieving nearly perfect selectivity. The resulting triple mutant SidM-MIII-S (A99G/A252S/M152R) enabled preparative-scale synthesis of (2*S*,4*S*)-**3t** in 28% isolated yield and 99% de (Fig. 4).

Intriguingly, SidM variants were also capable of inverting their Cγ stereochemical preference. Introduction of a G235S mutation into SidM-MI (SidM-MII-R) reversed the stereochemical outcome, affording (2*S*,4*R*)-**3t** with an inverted ration of 13:87 dr (Fig. 4). Further incorporation of a P122T mutation into SidM-MII-R (SidM-MIII-R, A99G/G253S/P122T) markedly enhanced Cγ *R*-selectivity, yielding (2*S*,4*R*)-**3t** in 34% isolated yield and 99% de (Fig. 4). Together, these complementary engineering trajectories enabled both enhancement and inversion of stereoselectivity within a common enzyme scaffold, providing a modular framework for tailor biocatalysts toward the asymmetric synthesis of either enantiomer. Notably, the stereoselectivity-inversed variant SidM-MIII-R was unable to alter the stereochemical outcome of the benzylic substrate **2a**, which still exclusively affording (2*S*,3*R*)-**3a**, as observed for SidM and SidM-MI. This is likely attributable to steric effects arising from the α-substituent of the nitroalkane substrate. Compared with the more flexible phenethyl group in **2t**, the benzylic substituent in **2a** is more rigid and sterically more demanding, thereby restricting the accessible nucleophilic attack trajectory required for formation of the inverted stereoisomer. This is supported by molecular dynamics simulations, which revealed that the *(2S,4S)*-**3a-TS3** experiences a substantially higher frequency of steric clashes than the corresponding *(2S,4R)*-**3a-TS3** within the SidM-MIII-R active site (Fig. S32). Further protein engineering will be required to achieve the stereochemistry inverse of these benzylic substrates.

### Gram-scale reaction and product transformation

**Fig. 5:**
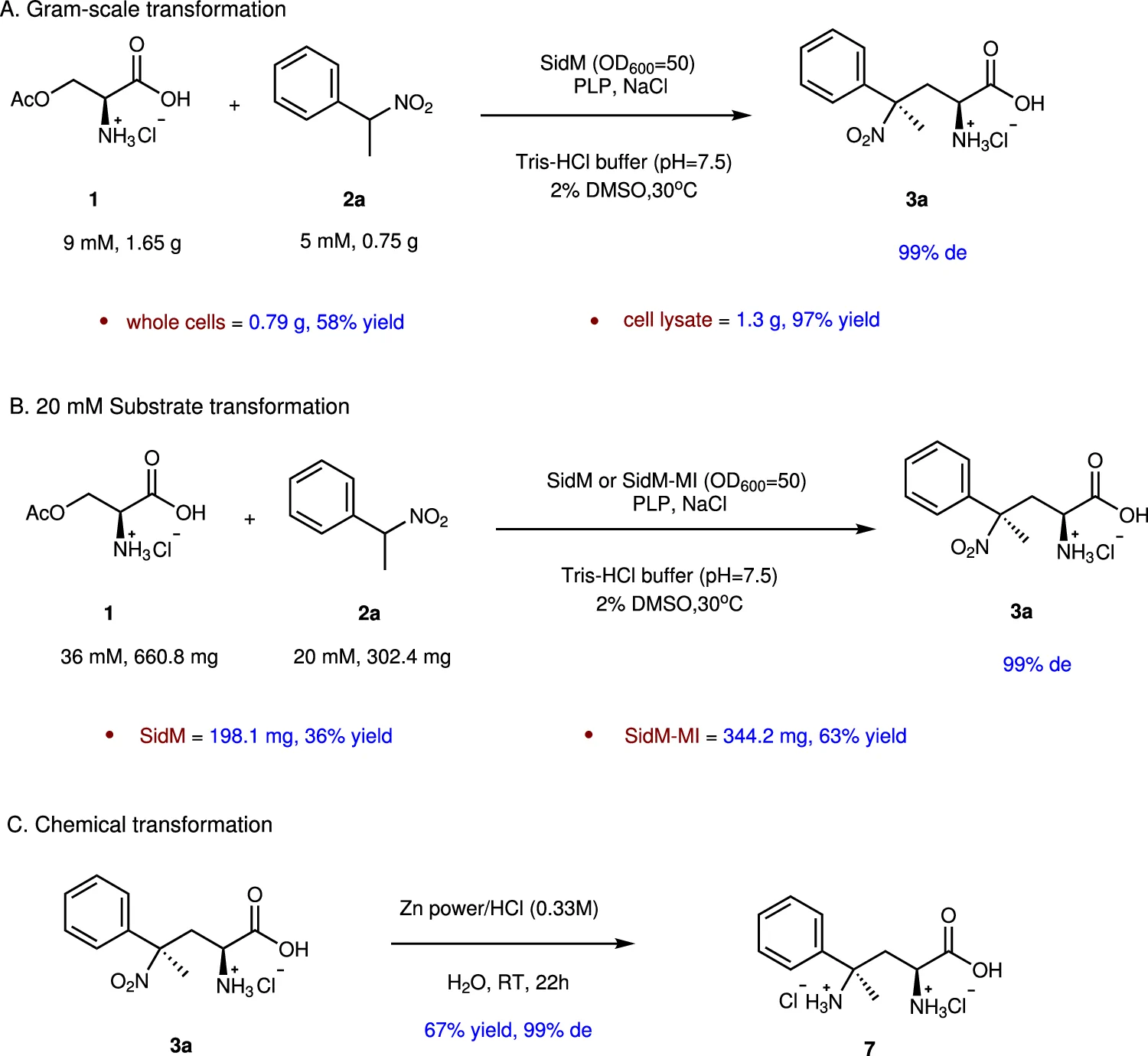
Gram-scale reaction and product transformation. **A** Biocatalytic asymmetric synthesis of product **3a** on 5 mmol scale. **B** Biocatalytic asymmetric synthesis of product **3a** on 20 mM scale. **C** Chemical transformation of product **3a** into diamino acid **7**.

To demonstrate the practical utility of this biocatalytic platform, we enlarged the model reaction into gram scale using SidM (Fig. 5A). Whole-cell biotransformation of **2a** (5 mmol) and **1** (9 mmol) at OD_600_ = 50 in a 1 L reaction volume afforded **3a** in 58% isolated yield (0.79 g) and 99% de. When using its cell lysate under otherwise identical conditions, the yield increased to 97% (1.3 g) with maintained 99% de, showing the efficiency and scalability of this enzymatic platform. Moreover, increasing the substrate loading of **2a** to 20 mM showed that wild-type SidM afforded **3a** in 36% yield, and SidM-MI delivered 63% yield, further demonstrating the robustness of this system (Fig. 5B). Given the synthetic versatility of nitro group, we explored the downstream transformation of **3a** under aqueous conditions (Fig. 5C). Simply reduction with zinc powder cleanly converted **3a** to the corresponding chiral α,γ-diamino acid **7** in 67% yield, with complete retention of γ-stereocenter. This product could be a valuable entry point to a range of γ-tertiary ncAAs, including γ-azide and γ-lactam through amine functionalization, showing its potential for further diversification and application.

### Mechanistic studies

**Fig. 6:**
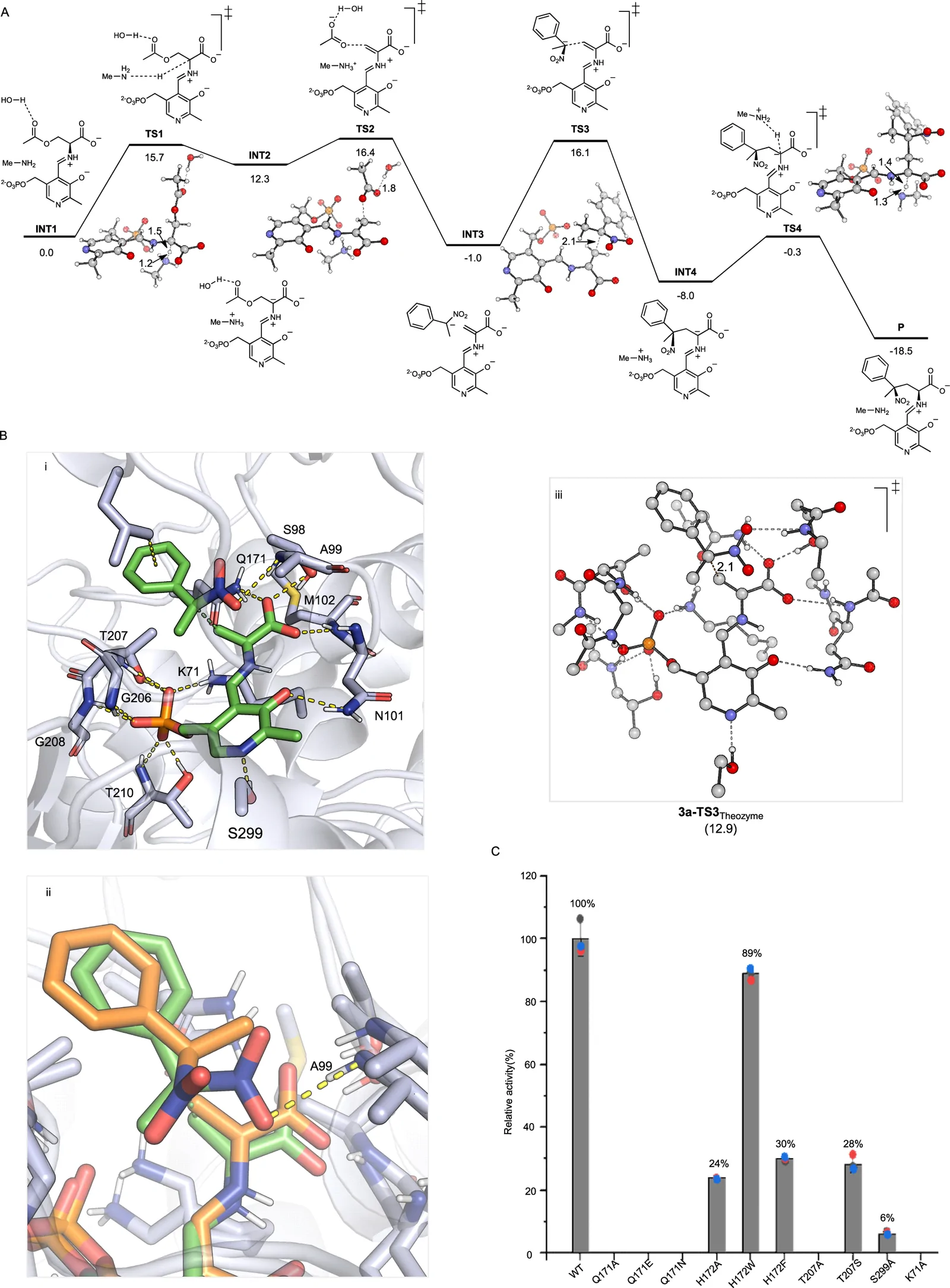
Mechanistic insights into the biocatalytic formation of γ-tertiary-nitro-α-amino acids. **A** Computed intrinsic reaction pathway. MeNH₂ models the catalytic lysine residue. Gibbs free energies (kcal/mol) were calculated at the ωB97X-D/6-31G(d,p)/SMD(water) level at 298 K. **B** The most representative conformation obtained from molecular dynamics simulations of (*2S,4R*)-**3a**-**TS3-**SidM complex and the key binding interactions are shown in (i) and the stereoselective models of (*2S,4R*)-**3a**-**TS3** (green) vs. (*2S,4S*)-**3a**-**TS3** (orange) are shown in (ii), and the theozyme model **3a-TS3**_Theozyme_ is shown in (iii). **C** Determination of the activities of SidM and its variants. The relative activity was examined by quantitative analysis of the production of **3a**. The bar height represents the average amount of product formed from three independent replicates with the error bars representing the standard deviation.

To elucidate the molecular basis underlying the outstanding activity and stereoselectivity of SidM, we performed density functional theory (DFT) calculations and molecular dynamics (MD) simulations. DFT calculations were first conducted to map the intrinsic reaction pathway between **1** and **2a** (Fig. 6A). The PLP-**1** aldimine adduct (**INT1**) was assigned as the zero-energy reference with a methylamine molecule included to represent the active-site lysine residue^59^. Deprotonation of **INT1** proceeds via **TS1** (15.7 kcal/mol) to yield the aldimine intermediate **INT2** (12.3 kcal/mol). Subsequent β-elimination through **TS2** (16.4 kcal/mol) generates the α,β-unsaturated intermediate **INT3** (−1.0 kcal/mol). Conjugate addition of **INT3** through **TS3**, with a 17.1 kcal/mol energy barrier as the rate-determining step of the overall reaction, to form the γ-tertiary-nitro amino acid adduct **INT4** (−8.0 kcal/mol). Final Cα-protonation of **INT4** via **TS4** (−0.3 kcal/mol) furnishes the γ-tertiary-nitro amino acid aldimine **P** (−18.5 kcal/mol). To determine the extent to which SidM reduces the barrier for **TS3**, we performed theozyme calculations. The results indicate that the barrier for **3a-TS3**_theozyme_ is 12.9 kcal/mol, suggesting that SidM can lower the barrier for **3a-TS3** by 4.2 kcal/mol, thereby facilitating the C–C bond formation reaction (Fig. 6B and Fig. S18).

To rationalize the exclusive formation of product **3a** with a *R*-configuration at the Cγ position, we next investigated how SidM affects the key stereo-determining transition state **TS3**. As no experimental structure of SidM is available, we therefore used AlphaFold 3^60^ to predict the structure of SidM. Both (*2S,4R*)**-3a-TS3** and (*2S,4S)***-3a-TS3** transition states were docked into the structure of SidM and relaxed to yield the corresponding **TS**-enzyme complex structure (Fig. 6B and Fig. S20). Analyzing from the complex structure that led to **3a**, we identified the key lysine residue K71 is responsible for covalent linkage with the PLP co-factor. We also identified conserved PLP-binding interactions^37,61^, specifically, S299 forms a hydrogen bond with the pyridinium nitrogen, and T207 and T210 of the phosphate binding pocket forming a hydrogen bond network with the phosphate group of PLP. From mutation experiments, substitution of either residue with alanine led to complete loss of activity, while more conservative T207S or S299T mutations partially retained activity (Fig. 6C). Residue Gln171, conserved among several cysteine synthases, was observed to interact with the carboxylate group of (*2S,4R*)**-3a-TS3** (Fig. S21). Mutations (Q171A, Q171N and Q171E) completely abolished activity, suggesting a possible role in amino acid substrate binding, while a potential structural function cannot be excluded. His172, positioned with 3.0 Å of the nitro group of (*2S,4R*)**-3a-TS3** in the model (Fig. S20), appeared to anchor the nitroalkane substrate through hydrogen bonding, its mutation to alanine resulted in a dramatic loss of activity (Fig. 6C).

To investigate how the conformational dynamics of SidM influence the transition state binding and the reaction stereoselectivity, we performed multiple 1 μs MD simulations (Figs. S20 and S22–S23). The stereoselective model derived from MD simulations suggests that the nitro group of *(2S,4R)*-**3a-TS3** forms a hydrogen bond with the backbone N atom of A99, whereas in the *(2S,4S)*-**3a-TS3** this hydrogen bond interaction is not observed (Fig. 6B). As a result, this interaction enforces a nucleophilic attack from the *Re* face of the nitro substrate, resulting in the *R*-configuration at Cγ. Consistent with this model, MMPBSA calculations revealed that SidM preferentially binds the *(2S,4R)*-**3a-TS3** transition state, with a binding free energy 10.7 kcal/mol lower than that of (*2S,4S*)**-3a-TS3** (Fig. S24).

Further mutations of SidM led to the discovery of SidM-MIII-R that led to greater *R* stereoselectivity for another substrate **3t**. MD analysis of SidM-MIII-R-*(2S,4R)-***3t-TS3** suggests that the key water bridged hydrogen bond of NO_2_-H_2_O-A97 (backbone C=O) is mainly responsible for the superior *R* selectivity (Figs. S25–26). Interestingly, SidM-MIII-S inverted the stereoselectivity to *S*. The inversed stereoselectivity was explained by analysis of the SidM-MIII-S-*(2S,4S)-***3t**-**TS3** where the NO_2_ of the substrate forms stable salt bridge interaction with R152 side chain guanidinium.

In summary, we report a pyridoxal 5′-phosphate-dependent enzymatic platform that enables the stereoselective construction of γ-tertiary-nitro-α-amino acids. By combining enzyme screening with directed evolution, SidM from siderochelin biosynthesis was repurposed to forge C–C bonds between serine derivatives and structurally diverse secondary nitroalkanes, furnishing the products in high yields and with excellent diastereoselectivity under mild aqueous conditions. This transformation not only delivers gram-scale access to valuable γ-tertiary-nitro-α-amino acids, but also outperforms existing chemical and biocatalytic methods, which have been largely restricted to secondary nitro-amino acids, narrow substrate scope, and harsher conditions. More broadly, our findings expand the mechanistic repertoire of PLP-dependent enzymes to encompass a challenging C–C bond-forming reaction, revealing their untapped potential as platforms for biocatalytic reaction discovery. By opening a biocatalytic route to previously elusive amino acid scaffolds, this work lays the groundwork for new opportunities in medicinal chemistry, peptide engineering, and natural product synthesis, and illustrates how the reprogramming of enzyme reactivity can transform long-standing synthetic challenges into practical solutions.

## Methods

### Protein expression and purification

Briefly, the corresponding plasmids (see the Plasmids Table in the Supplementary Materials), generated by inserting gene sequences synthesized by Shanghai Sangon Biotech Co., Ltd. (see the Gene Sequences section in the Supplementary Materials) into the pET-28a vector, were transferred into *E. coli* BL21 (DE3) for expression. A 50-mL culture of each *E. coli* transformant was incubated overnight in Lysogeny Broth (LB) medium containing 50 μg/mL kanamycin, diluted 100-fold in fresh LB medium and then incubated at 37 °C and 220 rpm until the cell density reached 0.6–0.8 at OD_600_. Protein expression was induced by the addition of isopropyl β-D-1-thiogalactopyranoside (IPTG) to the final concentration of 500 μM, followed by further incubation at 16 °C for 20 h. The cells were harvested by centrifugation and stored at −80 °C before lysis.

The thawed cells were re-suspended in lysis buffer (50 mM PBS buffer, 300 mM NaCl, 2.5 mM imidazole, 20% (v/v) glycerol, pH 8.0). After disruption by FB-110X Low Temperature Ultrapressure Continuous Flow Cell Disrupter (Shanghai Litu Mechanical Equipment Engineering), the soluble fraction was collected and subjected to purification of each target protein using a HisTrap FF column (GE Healthcare, USA). The desired protein fractions, as determined by SDS-PAGE, were concentrated and desalted using a PD-10 Desalting Column (GE Healthcare, USA) according to the manufacturer’s protocol. The resulting proteins were concentrated by a Vivaspin (GE Healthcare) and stored at −80 °C for in vitro assays.

### General procedure for biocatalytic assays

The frozen cells harboring SidM or its variants were resuspended in 100 mM Tris-HCl buffer (pH 7.5) and adjusted to the appropriate OD_600_. Cell lysis was then performed by sonication for 5 min. Each reaction was conducted in 100 μL reaction mixture containing 100 mM Tris-HCl buffer (pH 7.5), 50 mM NaCl, 6 mM **1**, 100 μM PLP, 2% DMSO, 2 mM substrate **2** (100 mM in DMSO) and cell lysate of *E. coli* harboring SidM or its variants (OD_600_ = 30) in 1.5 mL tip bottom centrifuge tube. The reaction mixtures were shaken at 350 rpm at 30 °C for 12 h, then quenched with acetonitrile (100 μL) and HCl solution (1 M, 200 μL). After standing for 1 h at room temperature, the reaction mixtures were centrifuged (12,000 *g*, 10 min, 20 °C) to remove insoluble components. To determine the analytical yield and diastereoselectivity, the quenched reaction mixtures were subjected to high-performance liquid chromatography (HPLC) analysis on Agilent Eclipse column (Plus C18, 5 μm, 4.6 × 250 mm) by gradient elution of solvent A (H_2_O with 1‰ formic acid) and solvent B (ethanol with 1‰ formic acid) at a flow rate of 1 mL/min, over a 14 min period as follow: *t* = 0 min, 30% B in A; *t* = 2 min, 30% B in A; *t* = 10 min, 60% B in A; *t* = 10.1 min, 100% B; *t* = 12 min, 100% B; *t* = 12.1 min, 30% B in A; *t* = 14 min, 30% B in A (λ = 280 nm).

### Library screening of SidM

*E. coli* BL21 (DE3) cells carrying *sidM* and its variants were grown in 2 mL round bottom 96-well plates (400 µL Lysogeny Broth (LB) medium per well, 50 μg/mL kanamycin) at 37 °C. After shaking at 800 rpm overnight, 20 µL of the overnight cultures were transferred to new 96-well plates containing 400 µL Terrific Broth (TB) per well (50 μg/mL kanamycin) for growing at 37 °C until OD_600_ reached 0.6, protein expression was induced by adding isopropyl β-D-1-thiogalactopyranoside (IPTG) to the final concentration of 500 μM, followed by further incubation at 16 °C for 20 h. Then, the cells were harvested by centrifugation (4000 *g*, 20 min, 4 °C) for biocatalytic assays.

### DFT calculation

Conformational search was performed using Molecular Operating Environment (MOE) program package with the default setting^62^. DFT calculation of each low energy conformer was performed using Gaussian 16 program package^63^. The structure of each species was submitted for geometry optimization at ωB97X-D/6-31G(d,p) level with SMD solvation model for water and with the integration grid set to ultrafine level, followed by frequency calculation at the same theoretical level. All reported Gibbs free energies are for 298 K and are after Truhlar-type quasi-harmonic correction using the GoodVibes program^64^.

### MD simulations

For the molecular dynamic simulations, the Amber 24 program^65^ and AmberTools 24 package^66^ were utilized. To simulate the proteins in complex with **TS3**, the structures from the docking calculations served as the input. The Protonate3D functionality of MOE was employed to determine the protonation state of proteins. The General Amber Force Field (GAFF) served as the source for deriving the ligand parameters. Using the Gaussian 16 program package, the charges were determined according to the Merz-Singh-Kollman scheme. For the simulations of transition state bound complexes (**TS3**-SidM), restraints were applied to the Cα-Cγ bond-forming distances of the ligand. These distances were set to match those in the QM-optimized transition state structure, using a bond parameter whose force constant corresponded to the imaginary frequency obtained from DFT calculations. Using the leap module, each protein complex was solvated in a pre-equilibrated cubic box, with a 15-Å buffer of TIP3P water molecules. To neutralize the systems, explicit counter ions (12 Na+ ions) were introduced. The particle mesh Ewald method, combined with periodic boundary conditions, was employed to model long-range electrostatic effects. Lennard-Jones and electrostatic interactions were truncated at a 10-Å cutoff. We conducted the molecular dynamics simulations through the steps described below:

(1) Each system underwent energy minimization for a maximum of 10,000 cycles. The steepest descent algorithm was applied for the first 8000 cycles, under periodic boundary conditions with constant volume (NVT). The SHAKE algorithm was not activated during this process. A positional restraint with a force constant of 5 kcal mol^−1^ ∙ Å^−2^ was imposed on the atoms of both the protein and the ligand. (2) A full-system minimization was performed using the identical protocols, in the absence of any restrictions. (3) Under NVT periodic boundary conditions, a 500 ps heating process was carried out with a 2 fs timestep and the SHAKE algorithm activated. The system was heated from 0 to 300 K over the course of 500 ps, driven by Langevin dynamics at a collision frequency of 2 ps^−1^. The protein and ligand atoms were held in place by a positional restraint of 5 kcal mol^−1^ ∙ Å^−2^. (4) Under NPT periodic boundary conditions, a 1 ns equilibration was carried out using the same timestep, with the temperature held constant at 300 K. The water box reached a stable density of 1.0 g/cm^3^, and its volume stabilized at approximately 660000.0 Å^3^. (5) A 1000 ns production run was conducted under standard NPT conditions, using the same timestep and a constant temperature of 300 K.

### Reporting summary

Further information on research design is available in the Nature Portfolio Reporting Summary linked to this article.

## Supplementary information

Supplementary Information

Description of Additional Supplementary Files

Supplementary_Data_1

Reporting Summary

Transparent Peer Review file

## Source data

Source data

## Data Availability

The data underlying the findings of this study are available in this article, its Supplementary Data and from corresponding author(s) upon request. The X-ray crystallographic coordinates for **3a** reported in this study have been deposited at the Cambridge Crystallographic Data Centre (CCDC), under deposition number CCDC 2379776. These data can be obtained free of charge from The Cambridge Crystallographic Data Centre via www.ccdc.cam.ac.uk/data_request/cif. Source data are provided with this paper.
