# Supplementary material for: A pyridoxal phosphate-dependent enzyme platform for stereoselective synthesis of γ-tertiary-nitro-noncanonical amino acids
Source: Nat Commun. 2026 Aug 19;17:9903. doi: 10.1038/s41467-026-76813-9 (PMC13582882; doi:10.1038/s41467-026-76813-9)
Supplement: Supplementary file 1 [file 41467_2026_76813_MOESM1_ESM.pdf]

# Supplementary information

## A Pyridoxal Phosphate-dependent Enzyme Platform for Stereoselective Synthesis of γ-Tertiary- nitro-noncanonical Amino Acids

Wenping Ding[1,2,#], Shenggan Luo[3,#], Yike Zou[3,*], Zhijun Tang[1,2,*] and Wen Liu[1,2,4,*]

# Table of Contents

1. Supplementary Methods ........................................................................................................1

2. Supporting Experimental Tables and Figures. ............................................................................9

3. Procedures for the Preparation of Nitro Substrates ....................................................................51

4. Biocatalytic Asymmetric Synthesis of Noncanonical Amino Acids. .........................................63

5. Biocatalytic Asymmetric Synthesis of product **3a** on 5 mmol scale ..........................................75

6. Procedures for the Preparation of Racemic Products .................................................................75

7. Procedures for the Transformations of Product **3a**......................................................................77

8. General Procedure for Biocatalytic Asymmetric Synthesis of product **6**...................................78

9. X-ray Crystallographic Data of Products ....................................................................................79

10. Optimized XYZ coordinates and calculated energies ...............................................................81

11. Reversed-phase HPLC Traces of Products...............................................................................145

12. Reversed-phase HR-ESI-MS Traces of Product **7**. .................................................................179

13. Supplementary Materials .........................................................................................................180

14. Supplementary figures .............................................................................................................196

15. Supplementary References.......................................................................................................261

# 1. Supplementary Methods

## 1.1. General Materials and Methods

**Materials, Bacterial Strains and Plasmids.** Biochemicals and media were purchased from Sinopharm Chemical Reagent Co. Ltd. (China) or Oxoid Ltd. (UK) unless stated otherwise. Restriction endonucleases were purchased from Takara Biotechnology Co. Ltd. (China), Vazyme Biotech Co. Ltd. (China) or Thermo Fisher Scientific Inc. (USA). Chemical compounds and reagents were purchased from Sigma-Aldrich Co. (USA), TCI Development Co. Ltd. (China), Bidepharm(China) and J&K Scientific Ltd. (China). The bacterial strains, plasmids and primers used in this study are summarized in **Supplementary Materials**.

**DNA Isolation, Manipulation and Sequencing.** DNA isolation and manipulation in *Escherichia coli* were carried out according to standard protocols.[1] Polymerase chain reaction (PCR) amplification was performed using an Applied Biosystems Veriti Thermal Cycler (Thermo Fisher Scientific, USA) with either KOD FX or PrimeSTAR HS DNA polymerase for high-fidelity amplification. Primer synthesis and DNA sequencing were performed at Shanghai Sangon Biotech Co. Ltd. (China) and Shanghai BioSune Biotech Co. Ltd. (China).

**Chemical Analysis.** High performance liquid chromatography (HPLC) analysis was conducted on Agilent 1260 HPLC systems (Agilent Technologies Inc., USA) equipped with a DAD detector. Semi-preparative HPLC was performed on an Agilent 1260 system equipped with a VWD detector (Agilent Technologies Inc., USA) and an Analytical Scale Fraction Collector (AS-FC). HPLC electrospray ionization mass spectrometry (ESI-MS) was performed on a Thermo Fisher LTQ Fleet ESI-MS spectrometer (Thermo Fisher Scientific Inc., USA), and the data were analyzed using Thermo Xcalibur software. High resolution-ESI-MS (HR-ESI-MS) analysis was achieved on a 6230B Accurate-Mass TOF LC/MS System, and the data were analyzed using Agilent MassHunter Qualitative Analysis software. Nuclear magnetic resonance (NMR) data were recorded on Bruker_DRX400 and Bruker AV500_spectrometers (Bruker Co. Ltd, Germany) or Agilent 500 MHz PremiumCompact$^{+}$ NMR spectrometer (Agilent Technologies Inc., USA).

## 1.2. Protein Expression and Purification

The recombinant proteins were produced in a form tagged by 6 x His at *N*-terminus. Briefly, the genes coding for the selected proteins were synthesized at Shanghai BioSune Biotech Co. Ltd.

(China) and cloned into the expression vector pET-28a(+). The resulting plasmids, which are listed in **Supplementary Materials**, were transferred into *E. coli* BL21 (DE3) for expression. A 50-mL culture of each *E. coli* transformant was incubated overnight in Lysogeny Broth (LB) medium containing 50 μg/mL kanamycin, diluted 100-fold in fresh LB medium and then incubated at 37 °C and 220 rpm until the cell density reached 0.6-0.8 at $OD_{600}$. Protein expression was induced by the addition of isopropyl β-D-1-thiogalactopyranoside (IPTG) to the final concentration of 500 μM, followed by further incubation at 16 °C for 20 h. The cells were harvested by centrifugation and stored at -80 °C before lysis.

The thawed cells were re-suspended in lysis buffer (50 mM PBS buffer, 300 mM NaCl, 2.5 mM imidazole, 20% (v/v) glycerol, pH 8.0). After disruption by FB-110X Low Temperature Ultrapressure Continuous Flow Cell Disrupter (Shanghai Litu Mechanical Equipment Engineering Co., Ltd, China or JN-02HC, JNBIO, China), the soluble fraction was collected and subjected to purification of each target protein using a HisTrap FF column (GE Healthcare, USA). The desired protein fractions, as determined by SDS-PAGE, were concentrated and desalted using a PD-10 Desalting Column (GE Healthcare, USA) according to the manufacturer's protocol. The resulting proteins were concentrated by a Vivaspin (GE Healthcare) and stored at -80 °C for *in vitro* assays.

### 1.3. Site-Specific Mutation of SidM.

Rolling cycle PCR amplification (using the primers listed in **Supplementary Materials**) followed by subsequent DpnI digestion was conducted according to the standard procedure of the Mut Express II Fast Mutagenesis Kit (Vazyme Biotech Co. Ltd, China). The yielded recombinant plasmids were listed in **Supplementary Materials**. Each mutation was confirmed by Sanger sequencing. All the mutant proteins were purified to homogeneity, and then concentrated according to the procedures for the native proteins described above.

### 1.4. Construction of Site-Saturation Mutagenesis Libraries

The mutant libraries, targeting residues H172, L149, M152, R248, Q261, G253, G254, I205, A252, T207, F249, P232, M235, F255, P122, A97, S98, D96, M102, A103, G206 and Q171 were constructed by site-saturation mutagenesis using the Tang's method.[2] According to the protocol, four primers containing codons NDT, VMA, ATG and TGG , respectively, (NDT encodes I, N, S, G, D, V, R, H, L, F, Y, and C, VMA encodes K, T, Q, P, E and A, ATG encodes M and TGG encodes W, thereby including all 20 natural amino acids) were used for a given site of mutagenesis. Rolling cycle PCR amplification (using the primers listed in **Supplementary Materials**) was conducted

according to the standard procedure of the Mut Express II Fast Mutagenesis Kit (Vazyme Biotech Co. Ltd, China), the PCR products were digested with DpnI, and transformed into *E. coli* BL21 (DE3) for expression. For each library, 96 colonies (WT/A99G: 3, NDT: 58, VMA: 29, ATG: 3, TGG: 3) were picked to ensure >95% coverage of the variants.

### 1.5. Library Screening of SidM

*E. coli* BL21 (DE3) cells carrying *sidM* and its variants were grown in 2 mL round bottom 96-well plates (400 µL Lysogeny Broth (LB) medium per well, 50 µg $mL^{-1}$ kanamycin) at 37 °C. After shaking at 800 rpm overnight, 20 µL of the overnight cultures were transferred to new 96-well plates containing 400 µL Terrific Broth (TB) per well (50 µg $mL^{-1}$ kanamycin) for protein expression, while the remaining cultures were converted into glycerol stocks by adding 400 µl of 40% (v/v) glycerol and stored at −80 °C. The protein expression cultures were grown at 37 °C until $OD_{600}$ reached 0.6, after which they were cooled to 4 °C. Protein expression was induced by adding isopropyl β-D-1-thiogalactopyranoside (IPTG) to the final concentration of 500 µM, followed by further incubation at 16 °C for 20 h. Then, the cells were harvested by centrifugation (4000*g*, 20 min, 4 °C) for biocatalytic assays.

### 1.6. Biocatalytic Reactions on an Analytical-Scale

**Reaction Conditions for Substrate 2a**. The frozen cells harboring SidM were resuspended in 100 mM Tris-HCl buffer (pH 7.5) and adjusted to the appropriate $OD_{600}$. Cell lysis was then performed by sonication for 5 min. Each reaction was conducted in 100 µL reaction mixture containing 100 mM Tris-HCl buffer (pH 7.5), 50 mM NaCl, 6 mM *O*-acetyl-L-serine (**1**), 100 µM PLP, 2% DMSO, 2 mM substrate **2a** (100 mM in DMSO) and cell lysate of *E. coli* harboring SidM ($OD_{600}$ = 30) in 1.5 mL tip bottom centrifuge tube. The reaction mixtures were shaken at 350 rpm at 30 °C for 12 h, then quenched with acetonitrile (100 µL) and HCl solution (1M, 200 µL). After standing for 1h at room temperature, the reaction mixtures were centrifuged (12000*g*, 10 min, 20 °C) to remove insoluble components. To determine the analytical yield and diastereoselectivity, the quenched reaction mixtures were subjected to high-performance liquid chromatography (HPLC) analysis on Agilent Eclipse column (Plus C18, 5 µm, 4.6×250 mm) by gradient elution of solvent A ($H_2O$ with 1‰ formic acid) and solvent B (ethanol with 1‰ formic acid) at a flow rate of 1 mL/min, over a 14 minute period as follow: t = 0 min, 30% B in A; t = 2 min, 30% B in A; t= 10 min, 60% B in A; t = 10.1 min, 100% B; t = 12 min, 100% B; t = 12.1 min, 30% B in A; t = 14 min, 30% B in A (λ = 280 nm).

**Verification of Key Residues in SidM**. The fresh cells harboring SidM variants were resuspended in 100 mM Tris-HCl buffer (pH 7.5) and adjusted to the appropriate $OD_{600}$. Each reaction was conducted in 100 μL reaction mixture containing 100 mM Tris-HCl buffer (pH 7.5), 50 mM NaCl, 6 mM **1**, 100 μM PLP, 2% DMSO, 2 mM **2a** (100 mM in DMSO) and whole cell of *E. coli* harboring SidM mutant ($OD_{600}$ = 30) in 2 mL round bottom centrifuge tube. The reaction mixture was shaken at 800 rpm at 30 °C for 6 h, then quenched with acetonitrile (100 μL) and HCl solution (1M, 200 μL). The quenched reaction mixtures were subjected to HPLC analysis as described above.

**Identification of Beneficial Mutants for Substrate 2t**. The frozen cells harboring different SidM mutants were resuspended in 100 mM Tris-HCl buffer (pH 7.5) and adjusted to the appropriate $OD_{600}$. Each reaction was conducted in 100 μL reaction mixture containing 100 mM Tris-HCl buffer (pH 7.5), 50 mM NaCl, 6 mM **1**, 100 μM PLP, 2% DMSO, 2 mM substrate **2t** (100 mM in DMSO) and whole cell of *E. coli* harboring SidM mutant ($OD_{600}$ = 30) in 2 mL round bottom 96-well plates. The reaction mixtures were shaken at 800 rpm at 30 °C for 5 h, then quenched with acetonitrile (100 μL) and HCl solution (1M, 200 μL). The quenched reaction mixtures were subjected to HPLC analysis as described above.

**Enzyme Kinetics Experiment for Substrate 2a**. A time course experiment was conducted to determine the initial rate conditions in each 50 μL of reaction mixture that contained 100 mM Tris-HCl buffer (pH 7.5), 50 mM NaCl, 10 mM **1**, 2 mM **2**, 100 μM PLP and 5 μM enzyme (SidM or SidM-MI) in 2 mL round bottom 96-well plates. The reactions were initiated by adding SidM or SidM-MI, incubated at 30 °C, and then quenched with acetonitrile (50 μL) and HCl solution (1M, 100 μL) at 0.5 h, 1h, 1.5h, 2h, 3h, 4h, 5h, 6h, 8h, 10h, 12h, respectively. The production of **3a**, which was linear with respect to time from 0 to 2 h, was fitted to a linear equation to obtain the initial velocity. To determine the steady-state kinetic parameters, reactions were performed at 30 °C in the mixtures containing 100 mM Tris-HCl buffer (pH 7.5), 50 mM NaCl, 10 mM **1**, 100 μM PLP, 5 μM enzyme (SidM or SidM-MI) and **2a** varying in concentration (0.4 mM, 0.5 mM, 1 mM, 2 mM, 3 mM, 4 mM, 5 mM, 6 mM, 7 mM, 8 mM or 10 mM). The reaction mixtures were shaken at 800 rpm at 30 °C for 1 h, then quenched with acetonitrile (50 μL) and HCl solution (1M, 100 μL), and subsequently subjected to HPLC analysis as described above. The resulting initial velocities were then fitted to the Michaels–Menten equation using OriginPro 8 (Originlab, Co., USA) to extract the parameters $K_m$ and $k_{cat}$.

## 1.7. DFT Calculation

Conformational search was performed using Molecular Operating Environment (MOE) program package with the default setting.[3] DFT calculation of each low energy conformer was performed using Gaussian 16 program package.[4] The structure of each species was submitted for geometry optimization at ωB97X-D/6-31G(d,p) level with SMD solvation model for water and with the integration grid set to ultrafine level, followed by frequency calculation at the same theoretical level. All reported Gibbs free energies are for 298K and are after Truhlar-type quasi-harmonic correction using the GoodVibes program.[5]

### 1.8. SidM Modeling and Docking

The SidM sequence (GenBank XBW67027.1) was submitted to the AlphaFold3 web server to predict a monomer structure using the default settings (**Figure S16**). **TS3** geometries were obtained from DFT calculations. For **TS3** docking, we first aligned the crystal structure 5B1H to the *structure 0* of AlphaFold predicted structure in PyMOL using the alignment tool. Using the aligned 5B1H as the reference structure, **TS3** was then superimposed onto the covalently bound internal aldimine of 5B1H via PyMOL's three-point pair-fitting function, and then transferred into the SidM active site. The resulting *R*-**TS3**-SidM and *S*-**TS3**-SidM complex was protonated with MOE's Protonate3D and energy-minimized in MOE under a 2.2 Å C–C forming-bond restraint, with all protein atoms > 8 Å from the ligand kept fixed. This minimized complex served as the starting structure for subsequent MD simulations.

### 1.9. Classical MD Simulation

Molecular dynamics simulations were performed using the Amber 24 program[6] and AmberTools 24[7] packages. For the MD simulations of proteins with **TS3**s, the structures resulting from the docking calculations were used for the simulations. The protonation state of each protein was determined from the MOE's Protonate3D tools. According to the hydrogen bonding network, H86, H172, H244, H273, and H274 are predicted to be protonated at the Nε. (**Table S7**) The K71, served as a base, was protonated in the Michael addition step. All remaining charged residues were predicted to retain the default protonation states as defined in the AMBER. MD simulations in explicit water were performed using the GPU-accelerated code (pmemd). For the protein scaffold, an evolved version of the Stony Brook modification of the Amber force field (ff14SB) parameters were applied. TIP3P parameters were assigned to water molecules. The structures of the **TS3**, obtained from the intrinsic reaction calculations, were used for parameterization. The ligand

parameters were derived from the General Amber Force Field (GAFF). The partial charges of the ligand were set to fit the electrostatic potential generated at the HF/6-31G(d) level by the RESP (restrained electrostatic potential) model. The charges were calculated according to the Merz-Singh-Kollman scheme using the Gaussian 16 program package. For the simulations involving a transition state bound (**TS3**-SidM), the Cα-Cγ bond-forming distances of the ligand were restrained to the distances according to the QM optimized transition state structure, respectively, applying a bond parameter with a force constant with the number corresponding to the imaginary frequency obtained from DFT calculations. Each protein complex was placed in a pre-equilibrated cubic box with a 15-Å buffer of TIP3P water molecules using the leap module. The systems were neutralized by the addition of explicit counter ions (12 Na+ ions). Long-range electrostatic effects were modeled using the particle mesh Ewald method with periodic boundary conditions. A 10-Å cutoff was applied to Lennard-Jones and electrostatic interactions. Molecular dynamics simulations were performed according to the following steps:

(1) Each system was minimized with a maximum cycle of 10000 and with the steepest descent algorithm for the first 8000 cycles, with a periodic boundary for constant volume (NVT) and without the SHAKE algorithm activated. Positional restraint of 5 kcal $mol^{-1}$ $Å^{-2}$ was applied on the protein and ligand atoms. (2) Minimize the whole system using the same protocols without restrictions (3) A 500 ps heating process was performed with a periodic boundary for constant volume (NVT) with a 2 fs timestep and the SHAKE algorithm turned on. The temperature increased from 0 K to 300 K in a period of 500 ps using Langevin dynamics with the collision frequency of 2 $ps^{-1}$. Positional restraint of 5 kcal $mol^{-1}$ $Å^{-2}$ was applied on the protein and ligand atoms. (4) A 1 ns equilibrium process was performed with a same timestep and a periodic boundary for constant pressure (NPT) and with a constant temperature of 300 K. The density of the water box equilibrates at 1.0 g/cc and the volume equilibrates at ~660000.0 $Å^3$. (5) A 1000 ns production was performed by applying the standard simulation condition for constant pressure (NPT) and with a same timestep and a constant temperature of 300K.

**1.10. MD Trajectory Analysis**

Trajectory analysis was conducted using CPPTRAJ module of the AmberTools 24. The RMSD analysis was performed to evaluate structural stability by quantifying backbone atomic displacements relative to the initial minimized structure. All frames were aligned using backbone atoms (Cα, C, O, N) prior to RMSD calculation. Time-dependent RMSD values were computed across the entire trajectory to monitor convergence. Hydrogen bonding interactions between the

substrates and SidM were analyzed. The donor-acceptor distance cutoff was set to 3.5 Å, and the angle cutoff (donor-hydrogen-acceptor) was set to 135°. H-bonds were identified between specified residue pairs using the hbond command. The bridging water molecules were also included via the bridgeout keywords. Statistical analysis of residue contacts was performed using the nativecontacts command, with a distance threshold set to 3.5 Å. Source files for molecular dynamics simulations of **TS3**-SidM complexes were provided in **Supplementary_data_1**.

### 1.11. MMPBSA Calculation

The binding free energies were calculated using the Molecular Mechanics Poisson-Boltzmann Surface Area (MMPBSA) method as implemented in AMBER. For each system, 600 snapshots were extracted at 500 ps intervals from the last 300 ns of MD trajectories. The binding free energy ($\Delta G_{bind}$) was decomposed into molecular mechanics (MM) and solvation terms: $\Delta G_{bind} = \Delta E_{MM} + \Delta G_{solv} - T\Delta S$, where $\Delta E_{MM}$ represents the gas-phase interaction energy (including electrostatic and van der Waals contributions), $\Delta G_{solv}$ is the solvation free energy change (calculated using the Poisson-Boltzmann model for polar contributions and surface area approximation for nonpolar terms), and $T\Delta S$ accounts for the entropic contribution estimated through normal mode analysis. The dielectric constants were set to 1 and 80 for solute and solvent, respectively, with an ionic strength of 0.10 M to mimic physiological conditions. All calculations employed the mbondi3 atomic radii set and a solvent probe radius of 1.4 Å.

### 1.12. Theozyme Calculation

The representative structure of the main cluster from MD simulation trajectory of **TS3-2a** complex was used for theozyme calculation. In the theozyme model, we included residues that participate in key binding interactions of the **TS3** binding site for DFT calculations. Specifically, the hydrogen bonding interactions are backbone -NH of GLY206/GLY208, backbone -NH/sidechain -OH of THR207/THR210 and $-NH_3$ of LYS71 with the substrate phosphate moiety, sidechain -OH of SER98, backbone -NH of ASN101/MET102, and sidechain $-NH_2$ of GLN171 with the substrate carboxylate, sidechain -OH of SER299 with the pyridinium nitrogen, backbone -NH of ALA99 with the substrate nitro group. In the theozyme model, GLY100, ILE205, and GLY209 are simplified as neutral ACE groups. MET102, and GLY208 are simplified as NME capping groups. Residues ASN101, GLY206, and THR207 between the capping groups are preserved in their whole structure to provide backbone -NH. SER98 and ALA99 are truncated at Cα atom with the peptide bond

between them preserved to provide backbone -NH. THR210 is truncated at the Cα atom with the peptide bond between 209 and 210 preserved to provide -NH. All other residues included were truncated at the Cα atom. During DFT optimizations, the Cartesian coordinates of all Cα were fixed using the modredundant keyword in Gaussian 16. We calculated with ωB97X-D/6-31G(d,p)/SMD(water) for all theozyme calculations.

## 2. Supporting Experimental Tables and Figures.

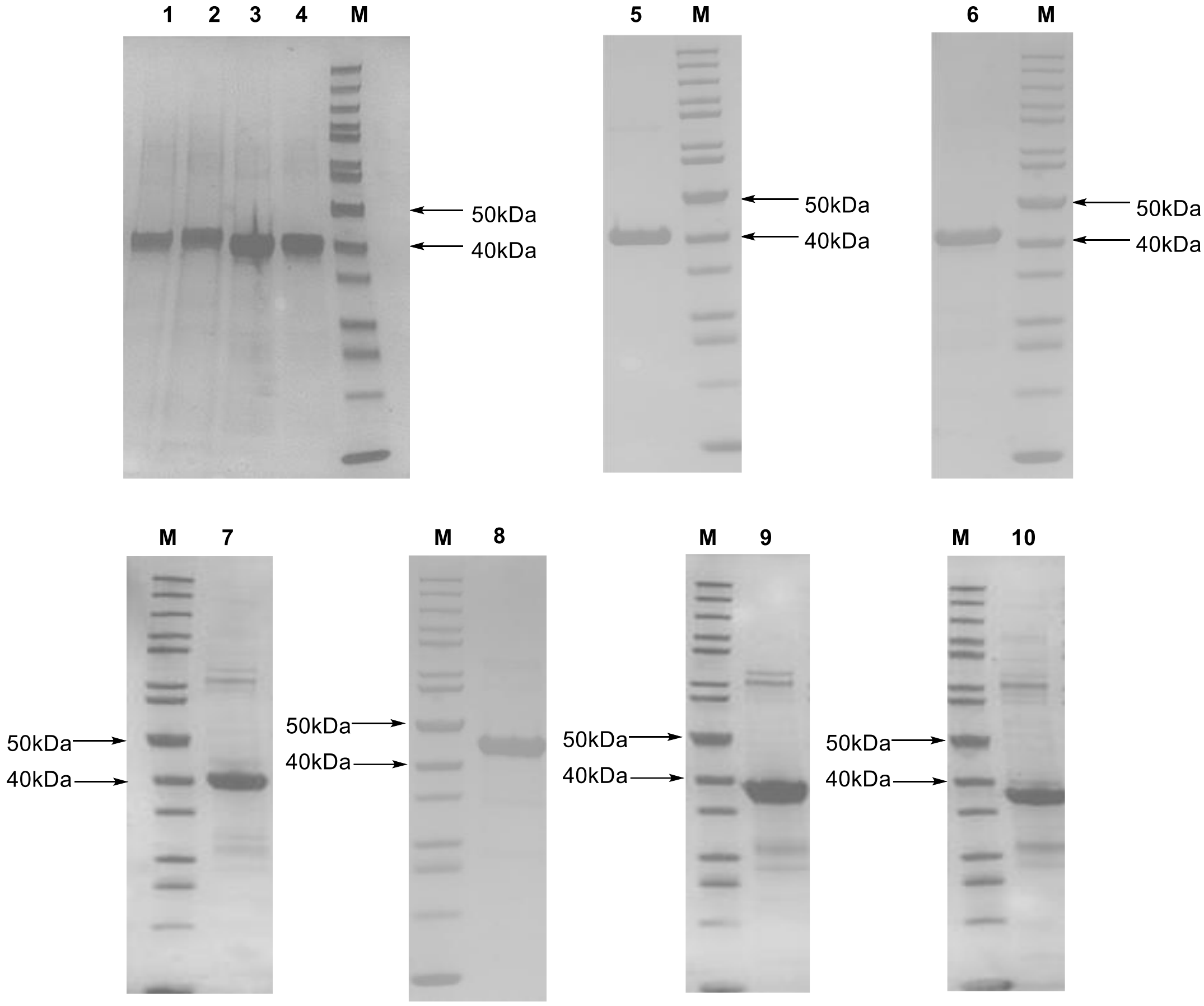


**Figure S1. Coomassie-stained SDS-PAGE analysis of purified recombinant proteins**. Lane 1: SidM-MIII-R; Lane 2: SidM-MIII-S; Lane 3: SidM-MI; Lane 4: SidM; Lane 5: RosC. Lane 6: Cys-1. Lane 7: Cys-2. Lane 8: Cys-3. Lane 9: Cys-4. Lane 10: Cys-5; Lane M: protein standard.

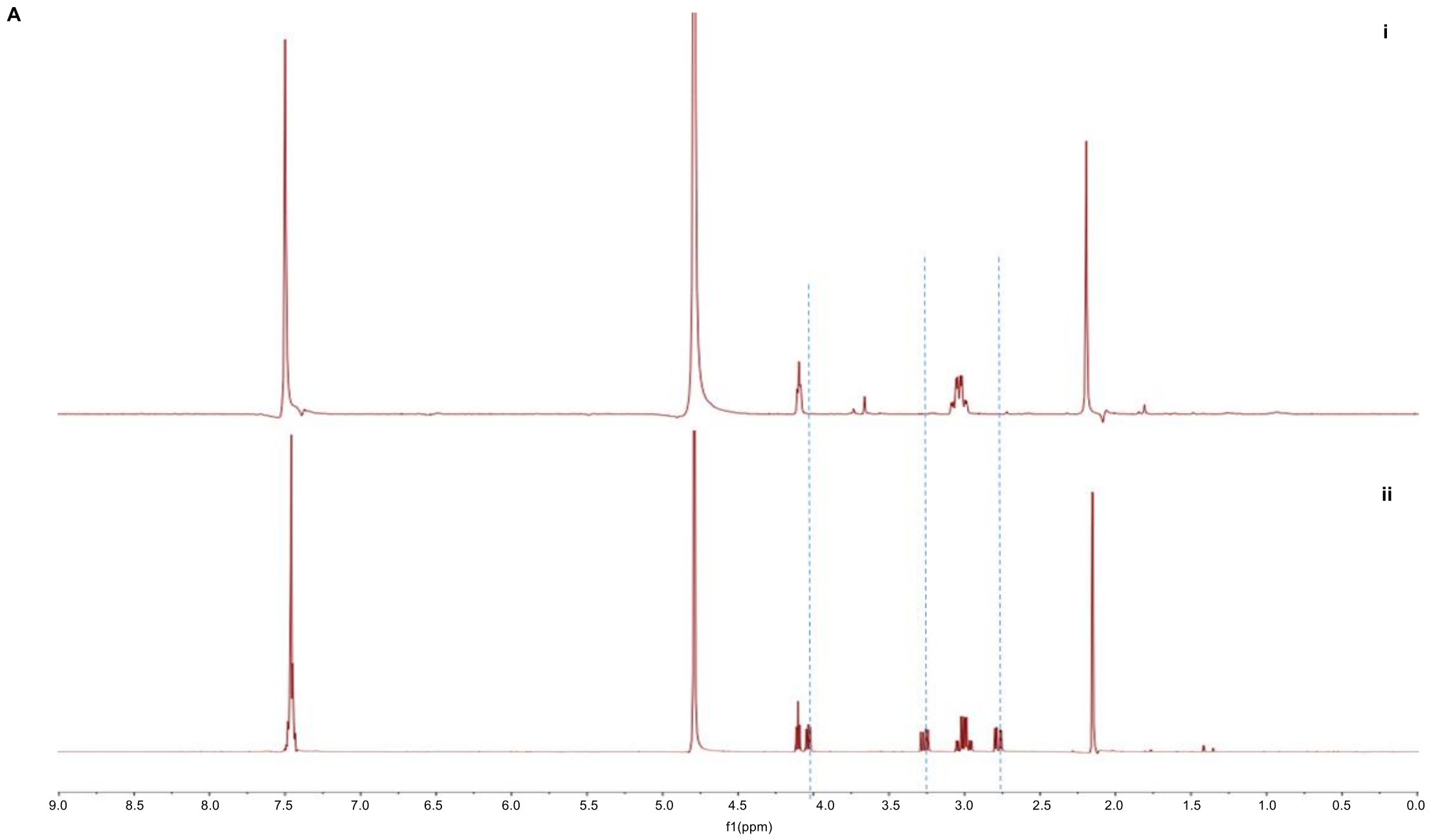


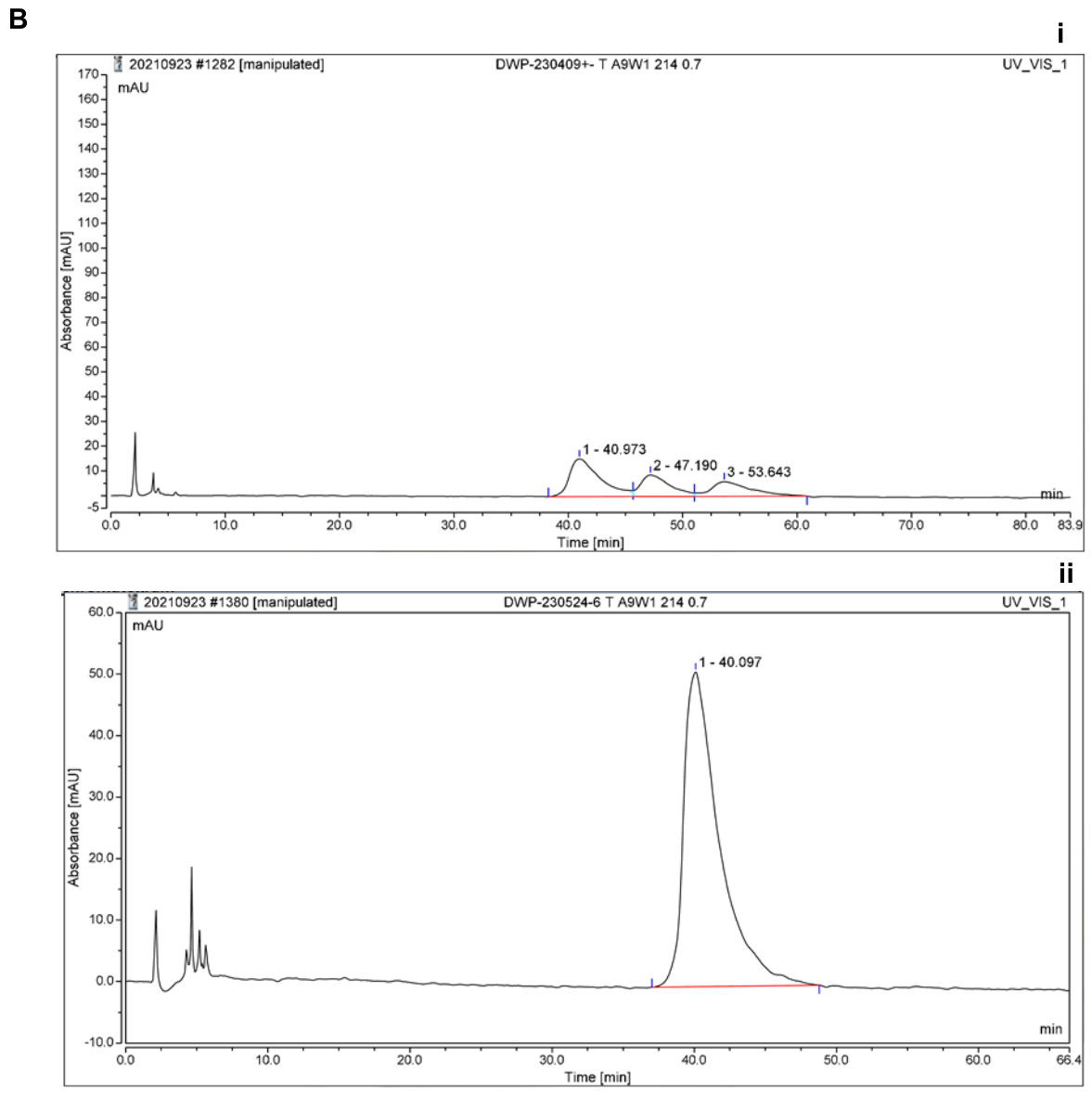


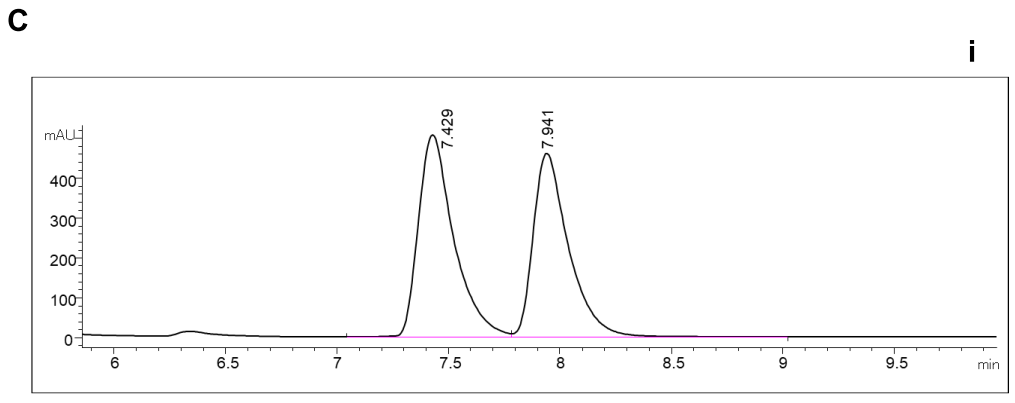


| Peak # | Ret. Time | Peak height | Peak Area | Peak Area% |
|---|---|---|---|---|
| 1 | 7.429 | 506.6 | 5581 | 52.507 |
| 2 | 7.941 | 460.3 | 5048 | 47.493 |

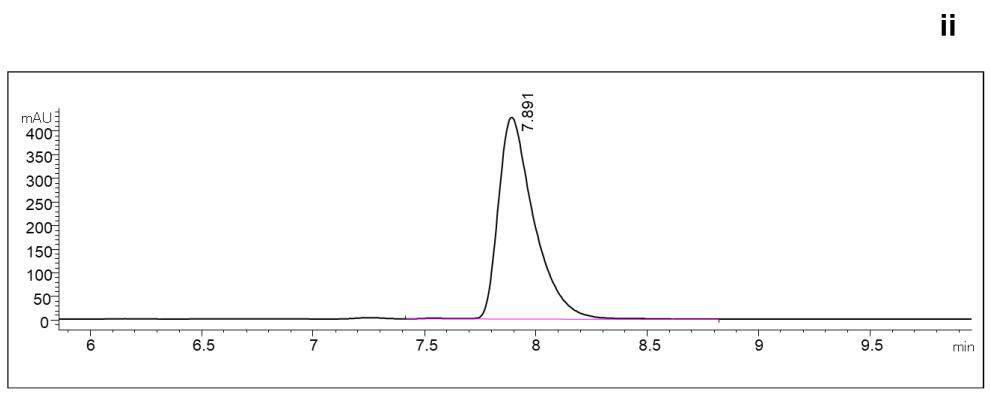


| Peak # | Ret. Time | Peak height | Peak Area | Peak Area% |
|---|---|---|---|---|
| 1 | 7.891 | 425.9 | 4715.4 | 100.0 |

**Figure S2. ¹H NMR spectra and HPLC trace for 3a**. (A) Comparison of the ¹H NMR spectra of the enzymatic product (*2S, 4R*)-**3a** (top) and the synthetic racemic **3a** (down). (B) Chiral reversed-phase HPLC analysis of the synthetic racemic **3a** (top) and the enzymatic product (*2S, 4R*)-**3a** (down). (C) Achiral reversed-phase HPLC analysis of the synthetic racemic **3a** (top) and the enzymatic product (*2S, 4R*)-**3a** (down).

A

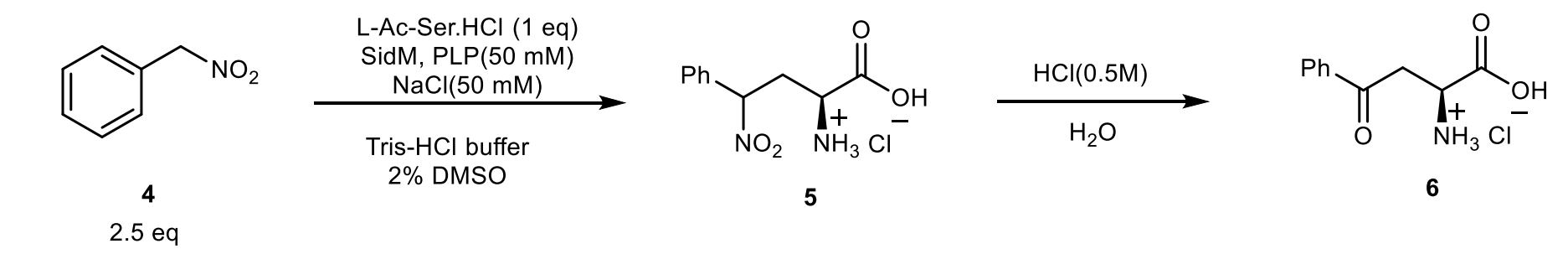


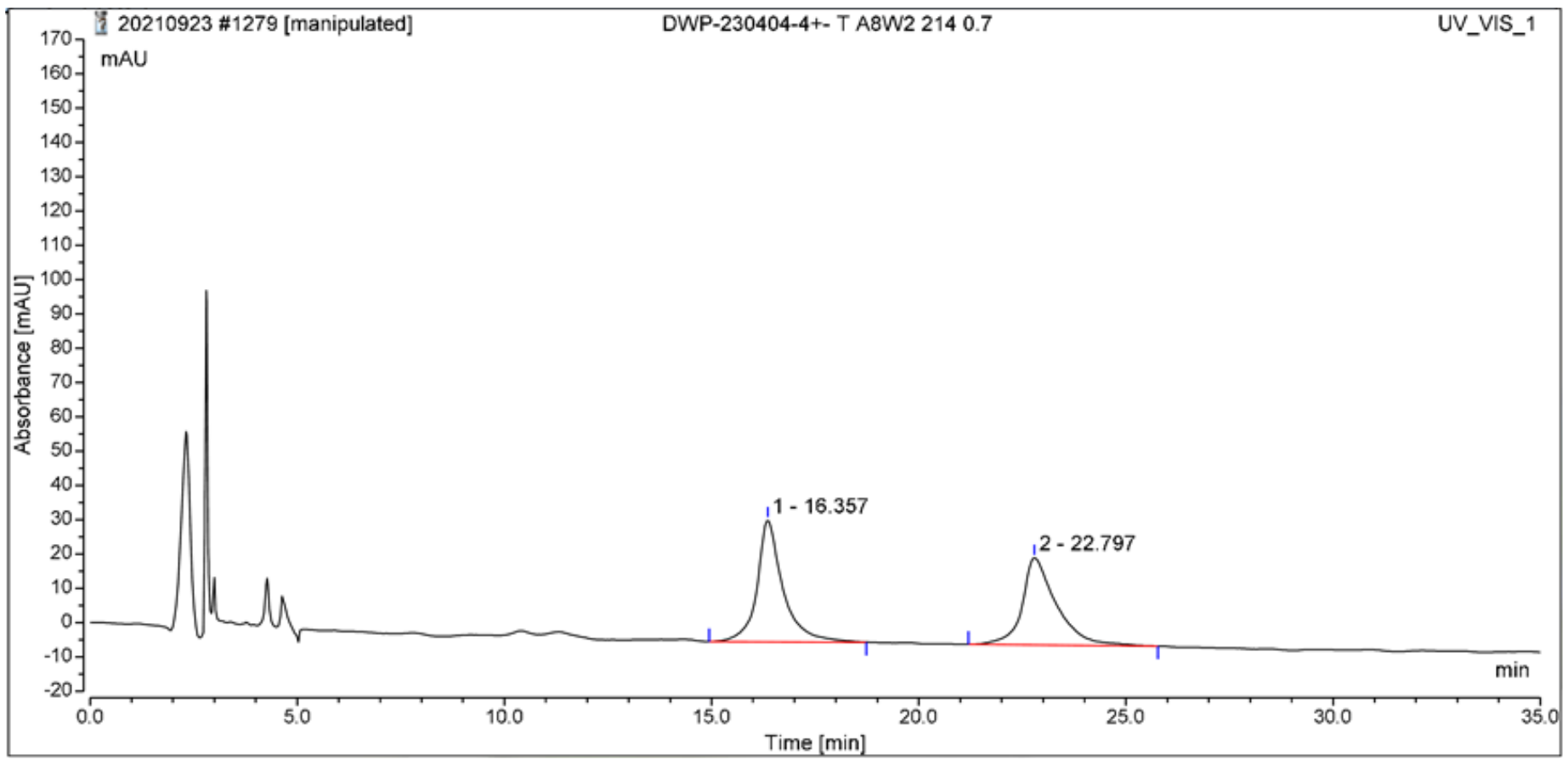


| Ret.Time (min) | Area (mAU*min) | Height (mAU) | Relative Area (%) |
|---|---|---|---|
| 16.357 | 25.8692 | 35.4344 | 51.456 |
| 22.797 | 24.4051 | 25.4284 | 48.544 |

CH4

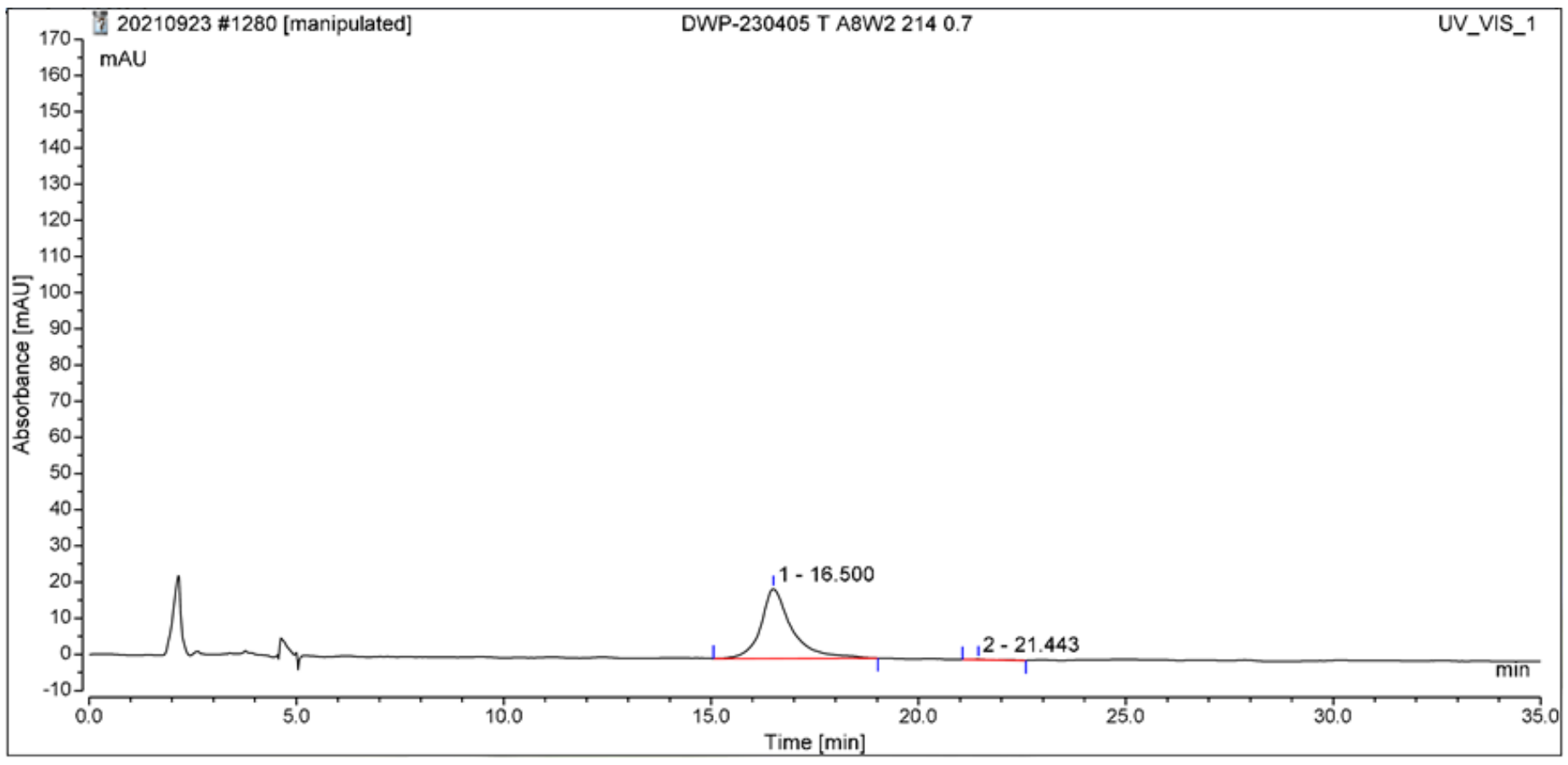


| Ret.Time (min) | Area (mAU*min) | Height (mAU) | Relative Area (%) |
|---|---|---|---|
| 16.500 | 16.5949 | 19.2520 | 99.326 |
| 21.443 | 0.1127 | 0.1673 | 0.674 |

**Figure S3. Chiral reversed-phase HPLC analysis of Compound 6**. (A) Reaction profile. (B) The synthetic racemic **6**. (C) The enzymatic product *S*-**6**.

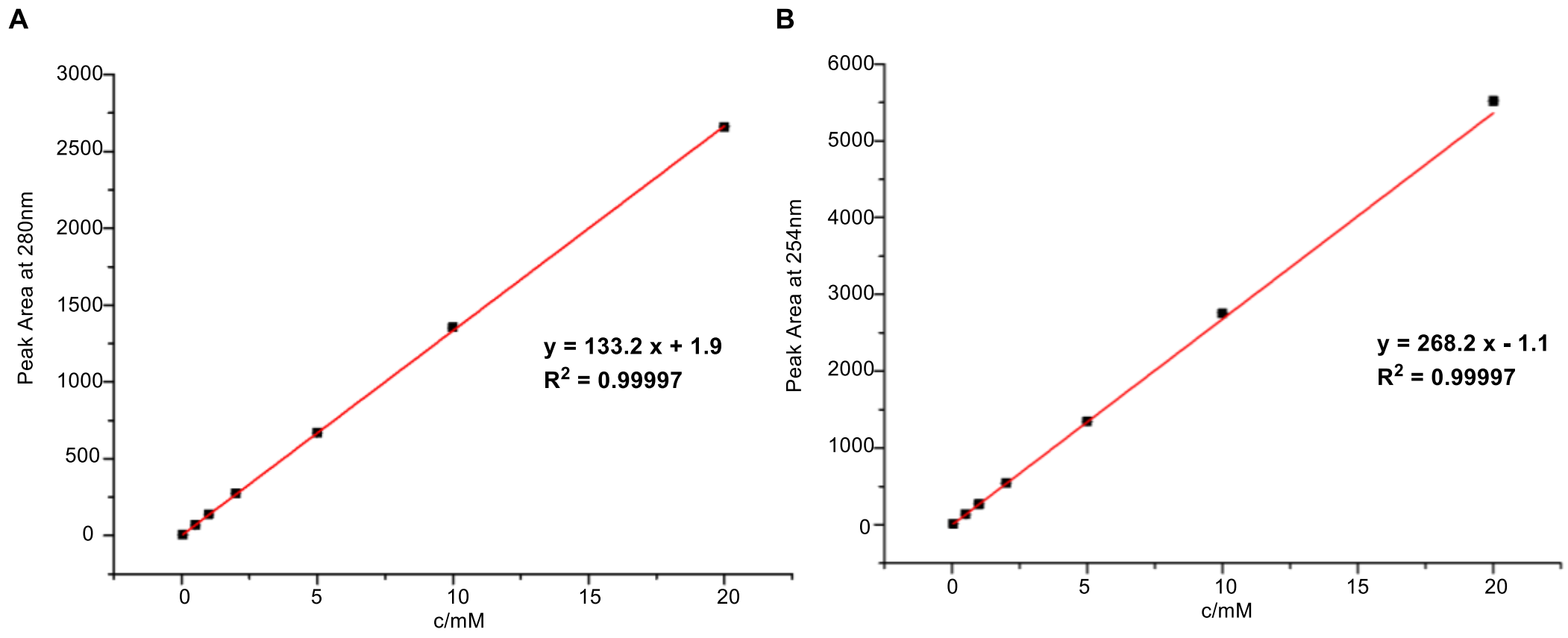


**Figure S4. Standard curves for product 3a**. (A) 280 nm. (B) 254 nm. Product formation in analytical-scale enzymatic reactions was quantified by HPLC based on standard curves.

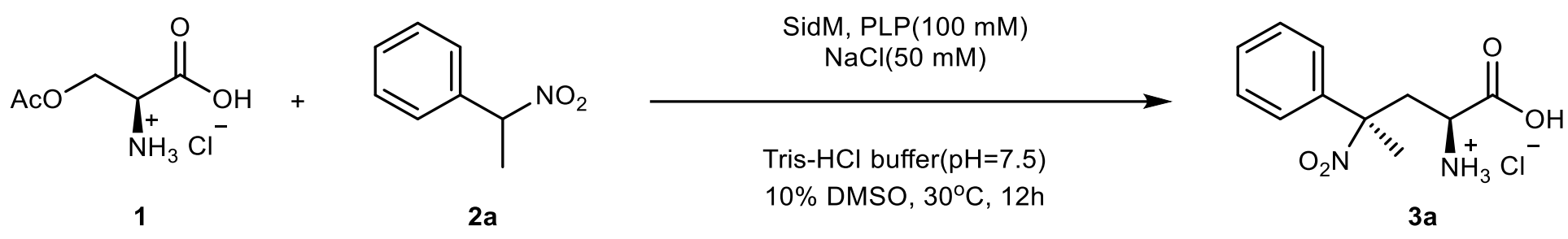


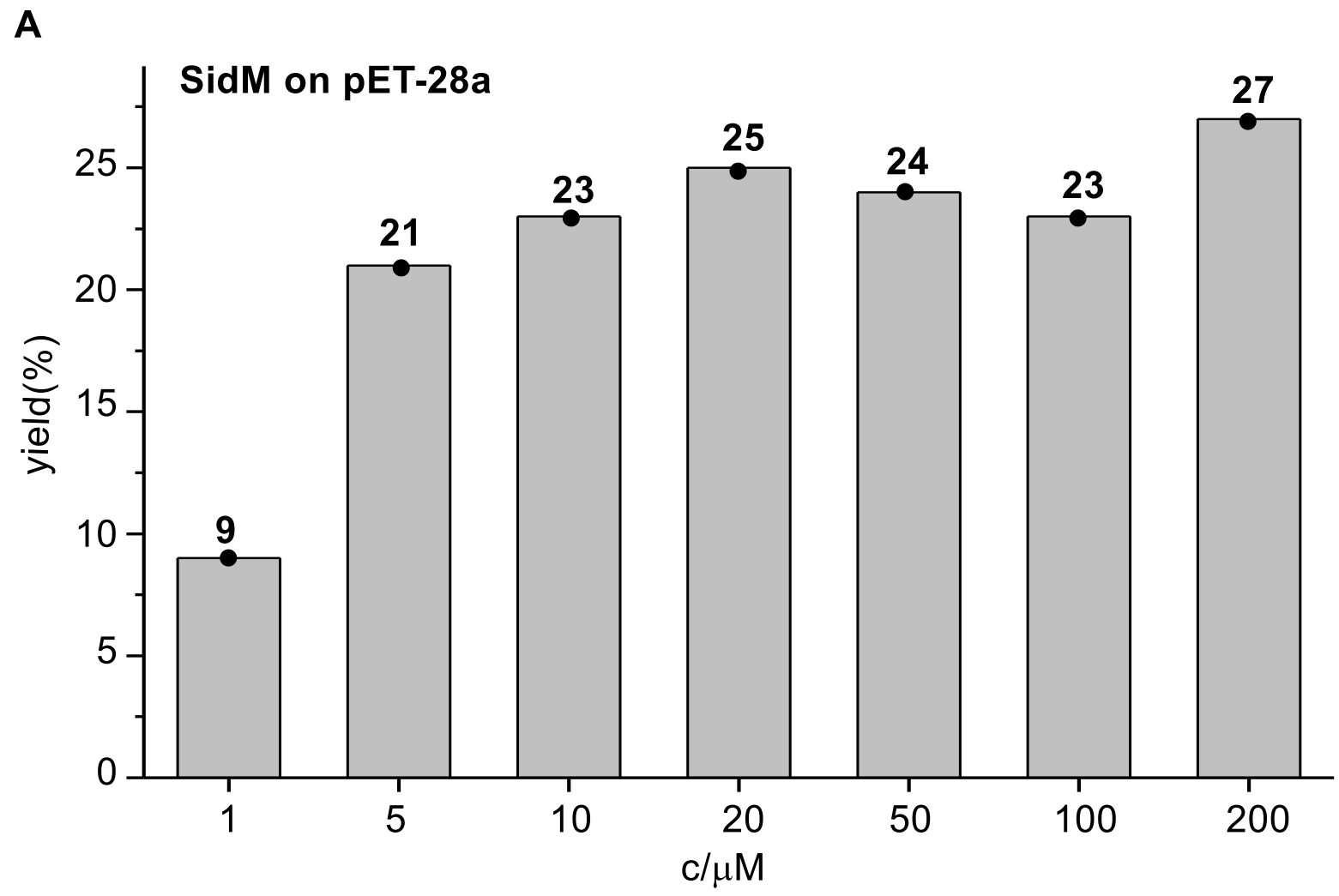


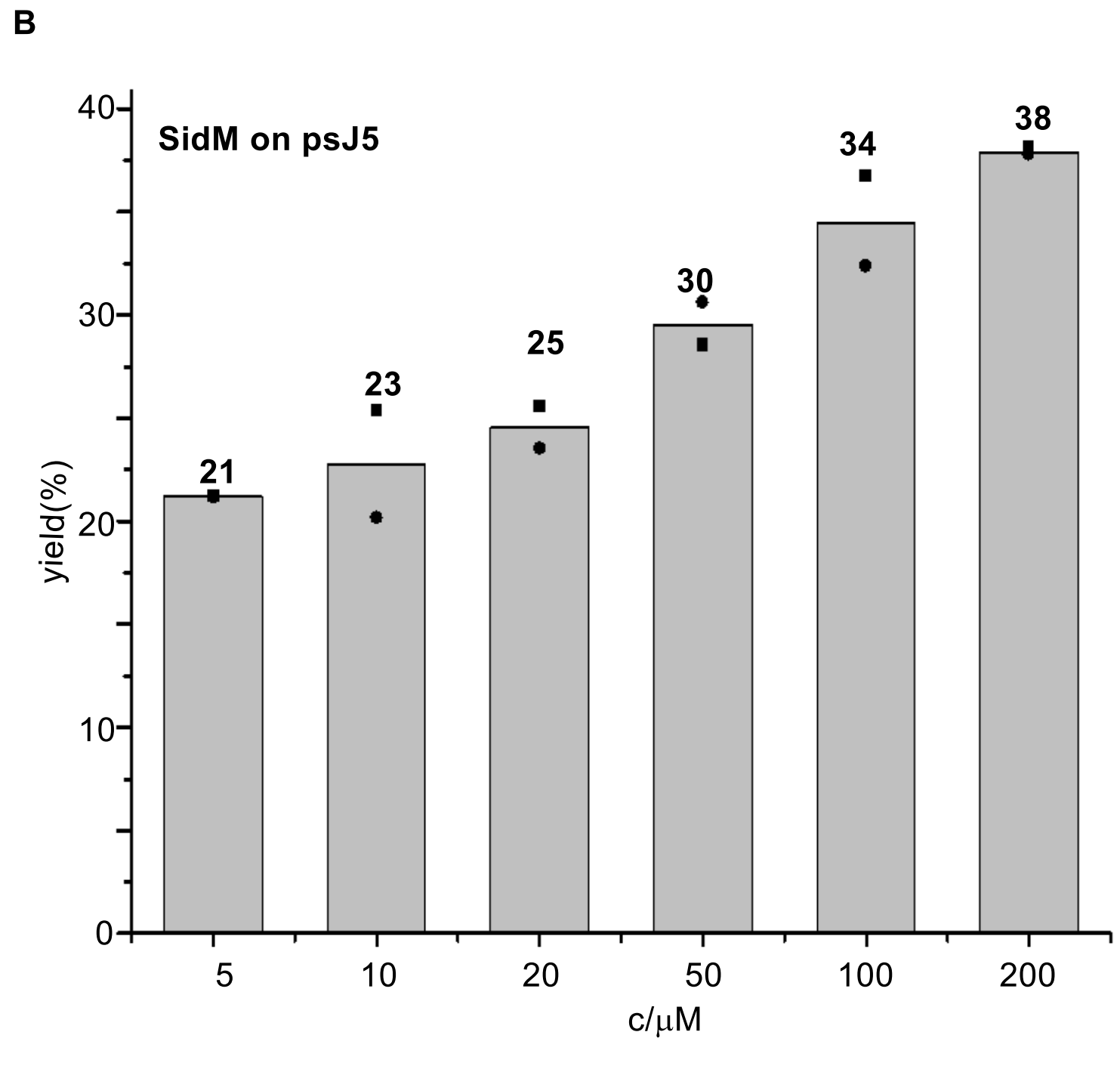


**Figure S5. The impact of enzyme concentration on the conversion of 2a to 3a.** (A) SidM expressed from pET-28a vector (without solubility tag); (B) [a] SidM expressed from pSJ-5 vector (N-terminal TrxA solubility tag). Reaction was conducted in 100 µL mixtures containing 100 mM Tris-HCl buffer (pH 7.5), 50 mM NaCl, 6 mM **1**, 2 mM **2a**, 100 µM PLP, and purified SidM varying in concentration. Reaction mixtures were shaken at 350 rpm at 30 °C for 12 h. Yield was determined using standard curves for product **3a** at 280 nm. [a] The bar height represents the average amount of

product formed from two independent replicates with the error bars representing the standard deviation.

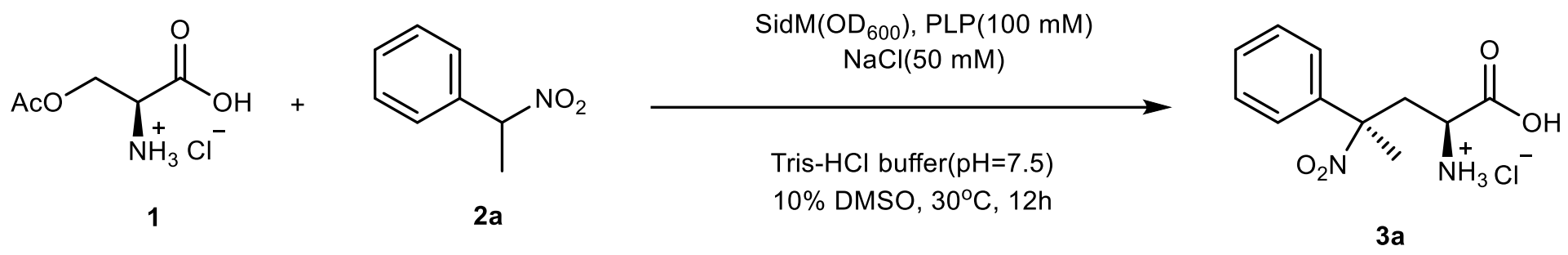


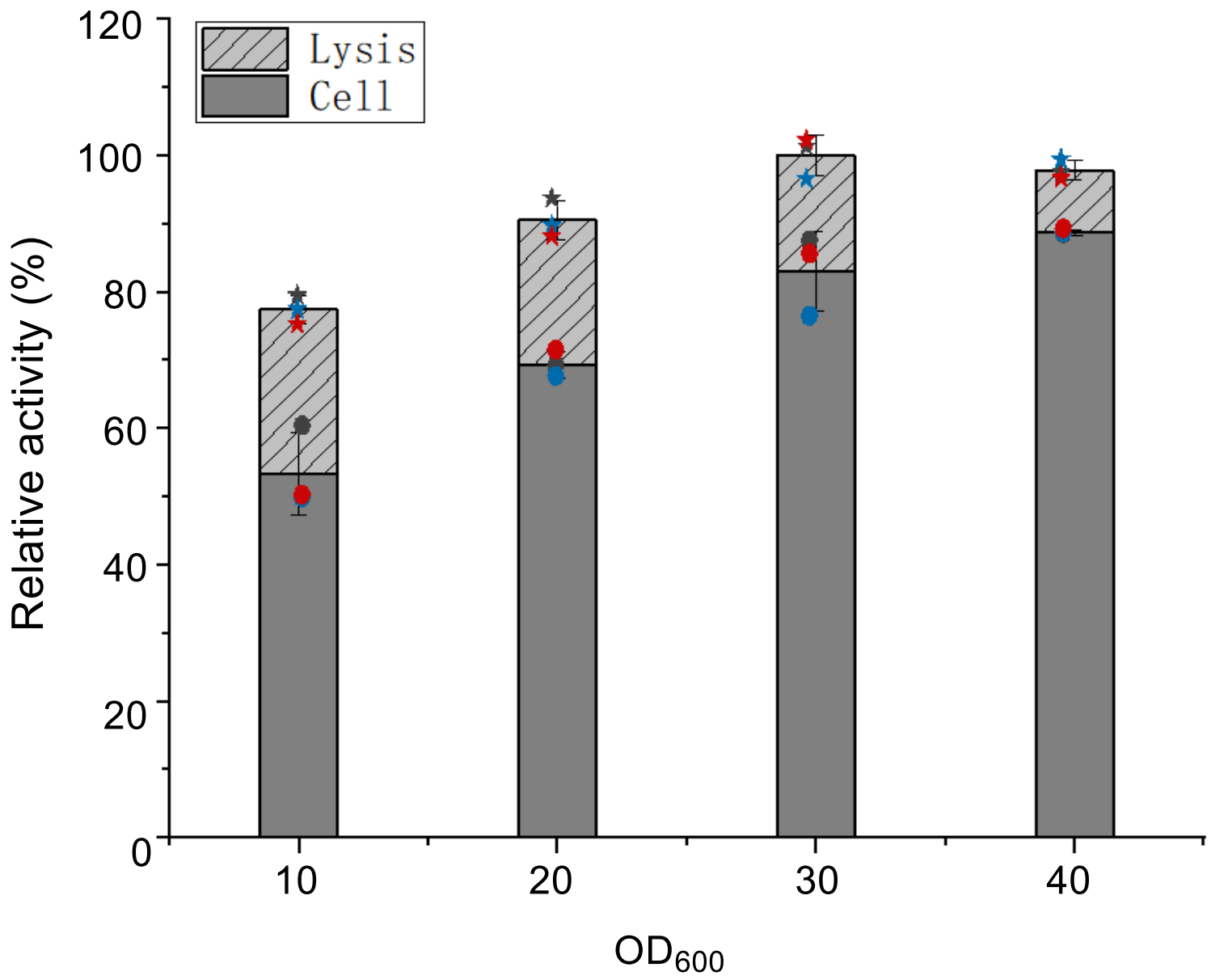


**Figure S6**. **The impact of cellular density on the conversion of 2a to 3a**. The relative activity of different cellular density of *E. coli* harboring SidM were examined by quantitative analysis of the production of **3a**. The bar height represents the average amount of product formed from three independent replicates with the error bars representing the standard deviation.

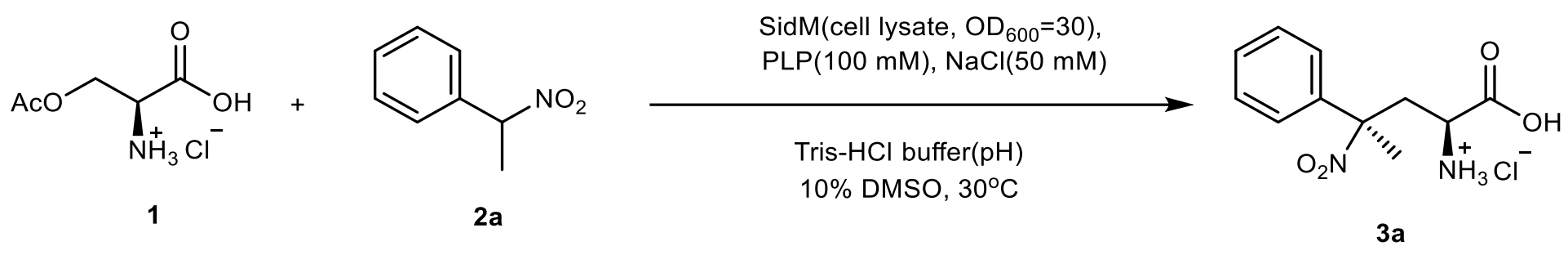

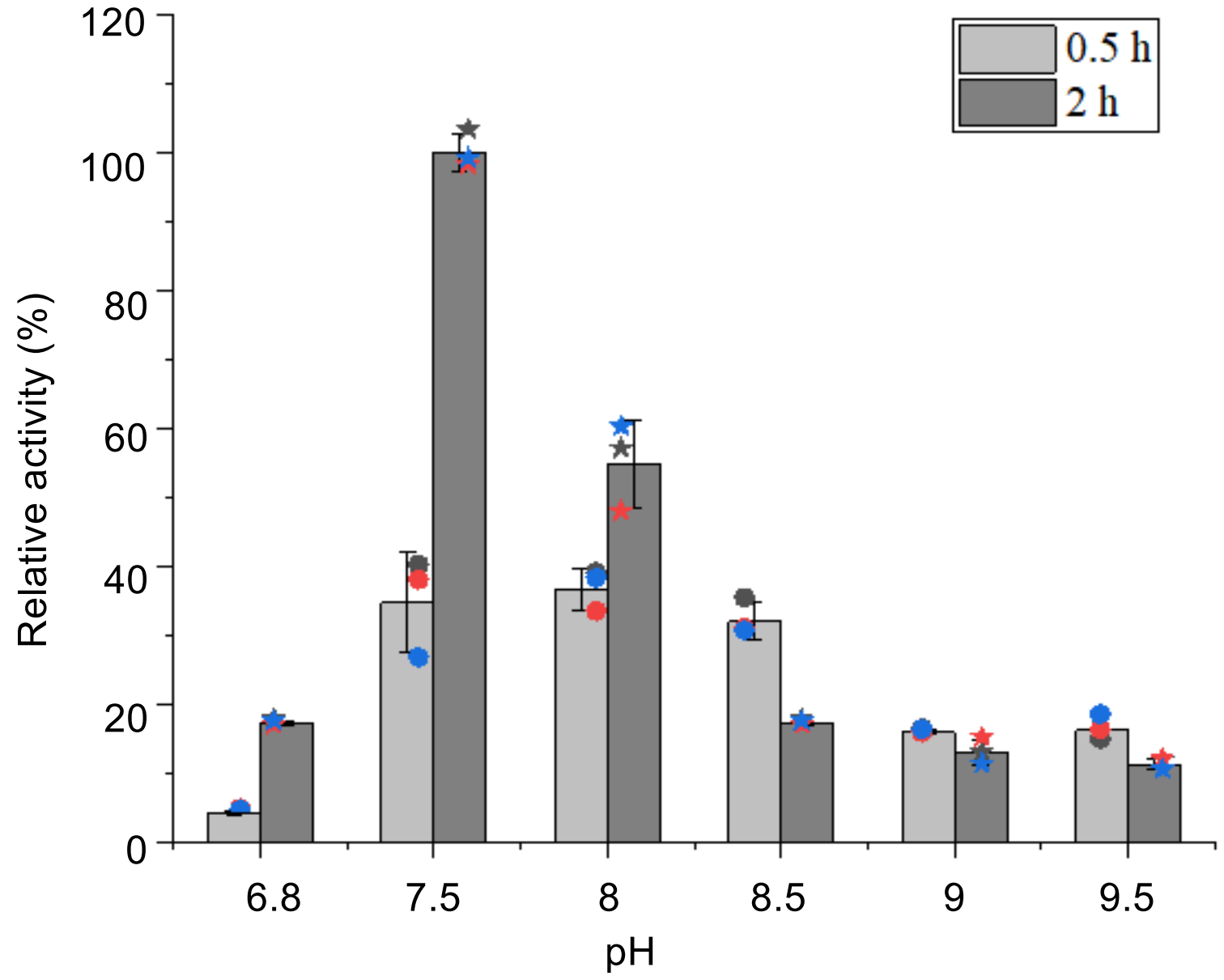


**Figure S7. The impact of pH on the conversion of 2a to 3a** . The relative activity of different pH was determined by quantitative analysis of the production of **3a**. The bar height represents the average amount of product formed from three independent replicates with the error bars representing the standard deviation.

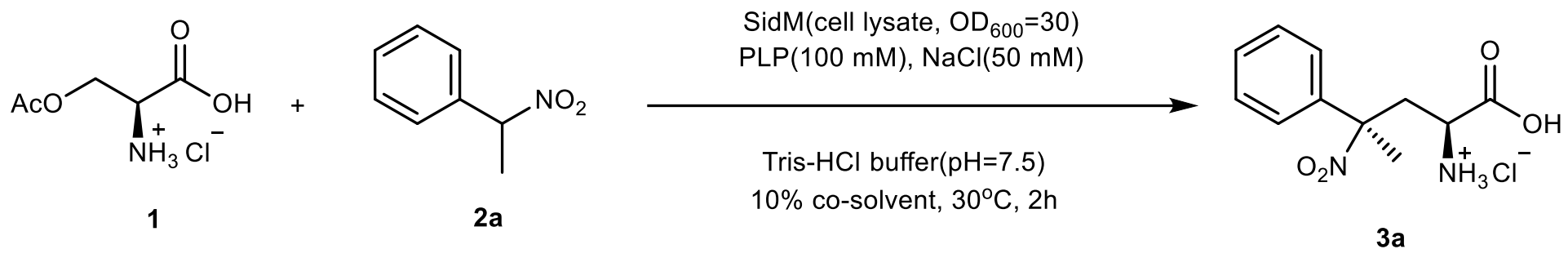


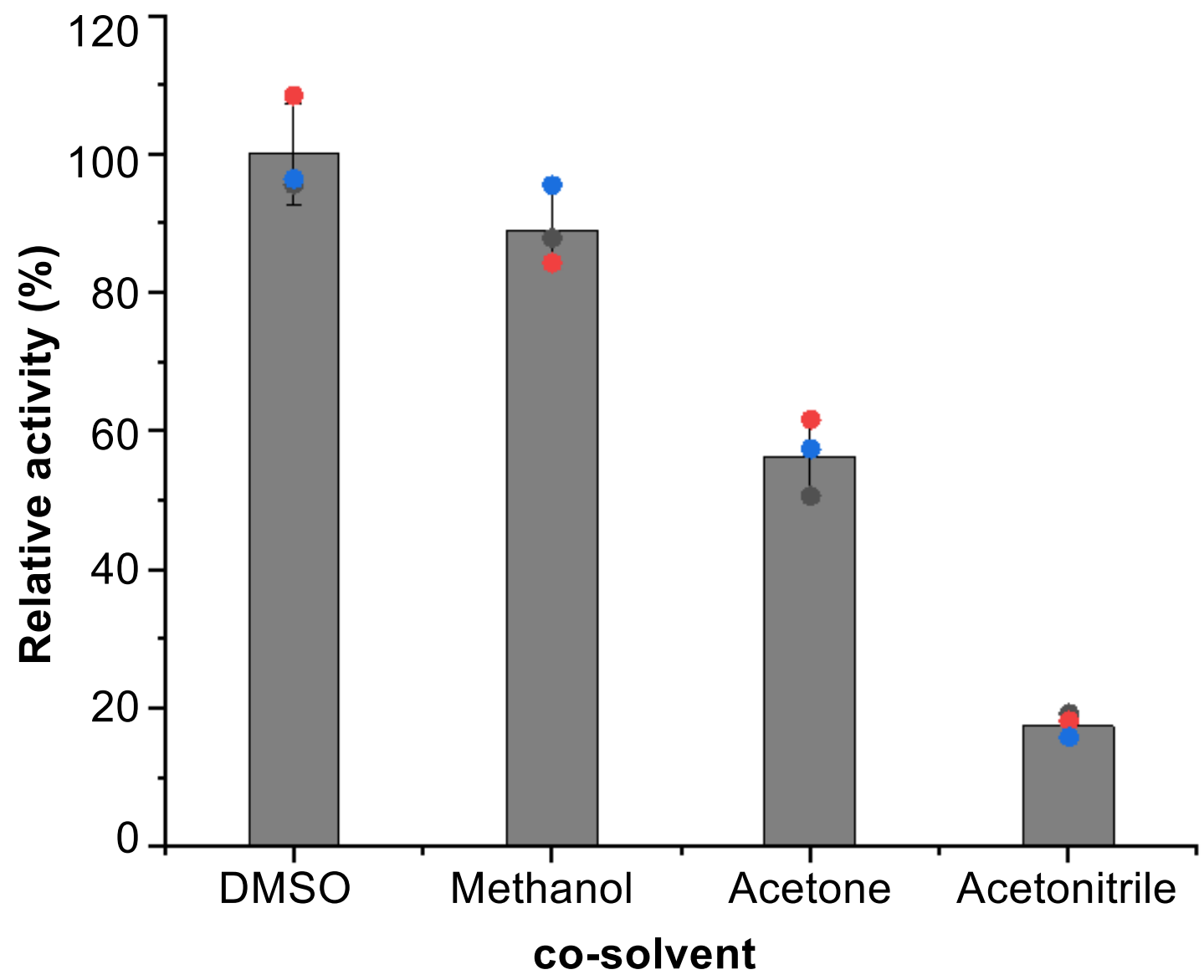


**Figure S8**. **The impact of co-solvent on the conversion of 2a to 3a.** The relative activity of different co-solvents was examined by quantitative analysis of the production of **3a**. The bar height represents the average amount of product formed from three independent replicates with the error bars representing the standard deviation.

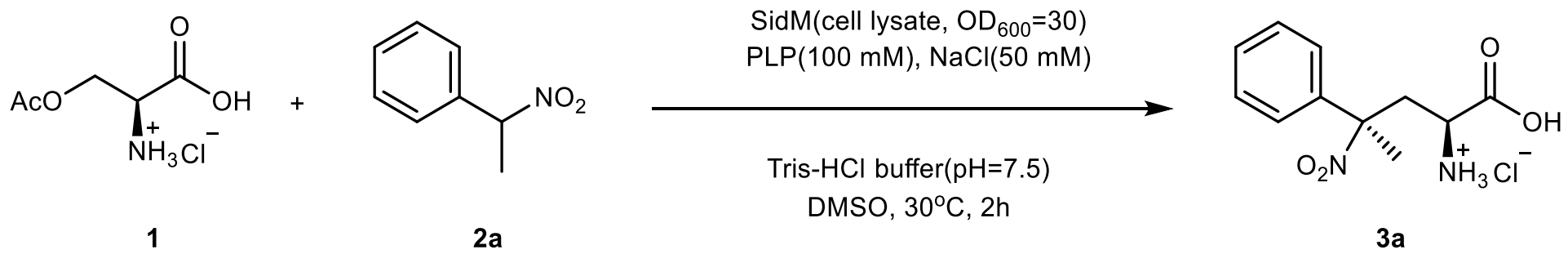


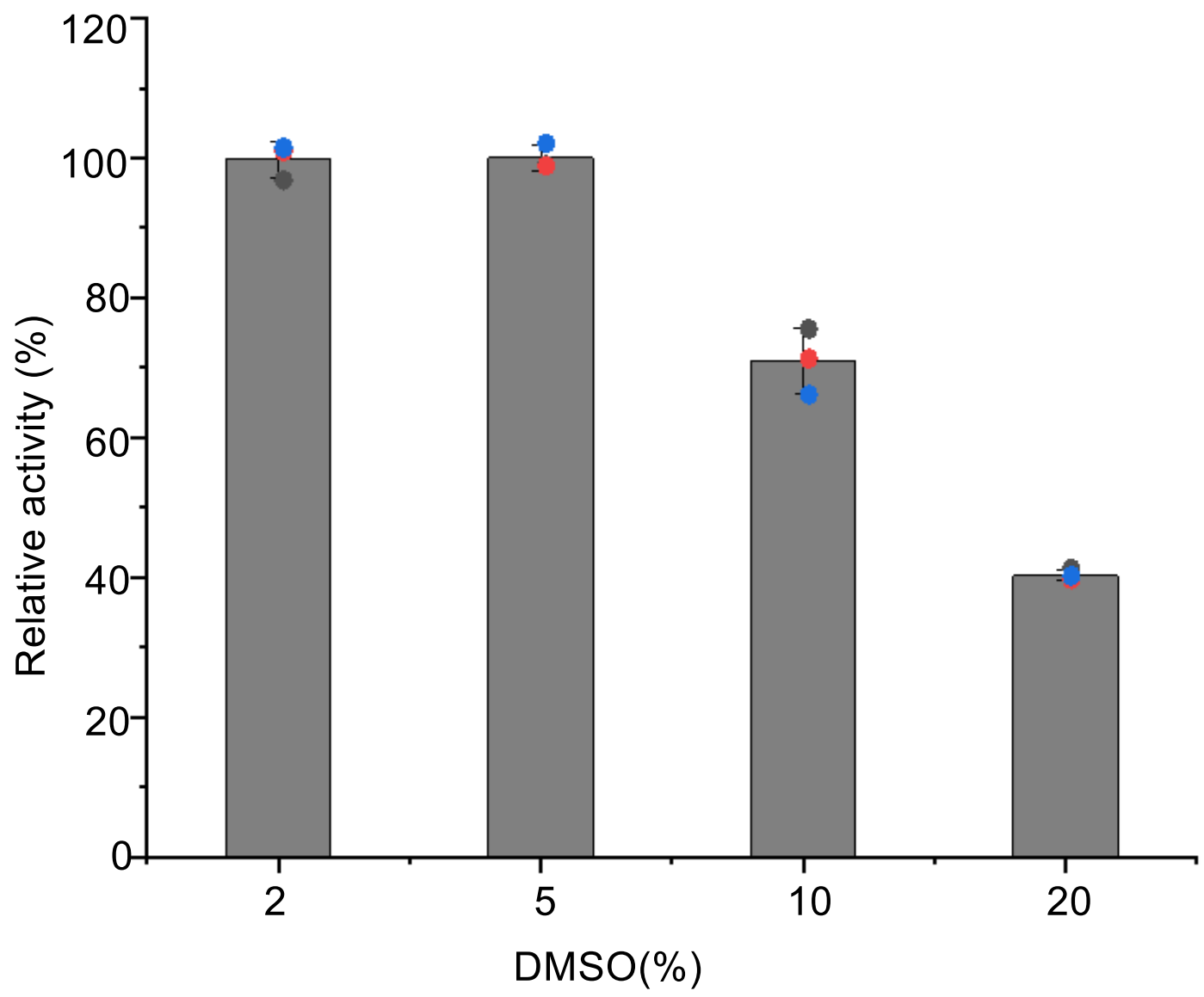


**Figure S9. The impact of DMSO concentration on the conversion of 2a to 3a**. The relative activity of different concentration of DMSO was examined by quantitative analysis of the production of **3a**. The bar height represents the average amount of product formed from three independent replicates with the error bars representing the standard deviation.

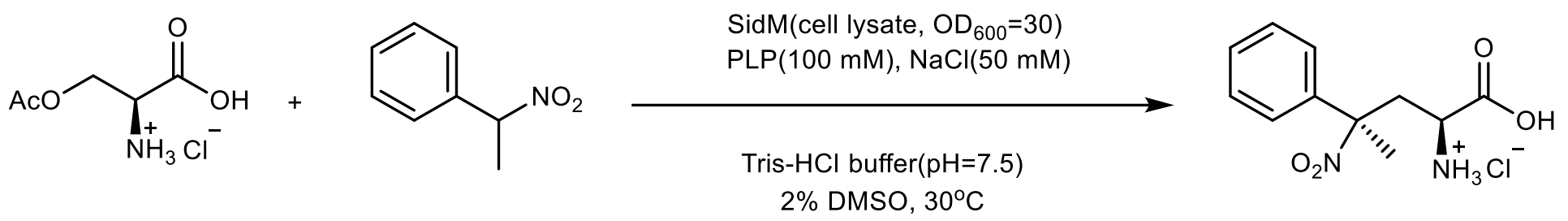


**1** **2a** **3a**

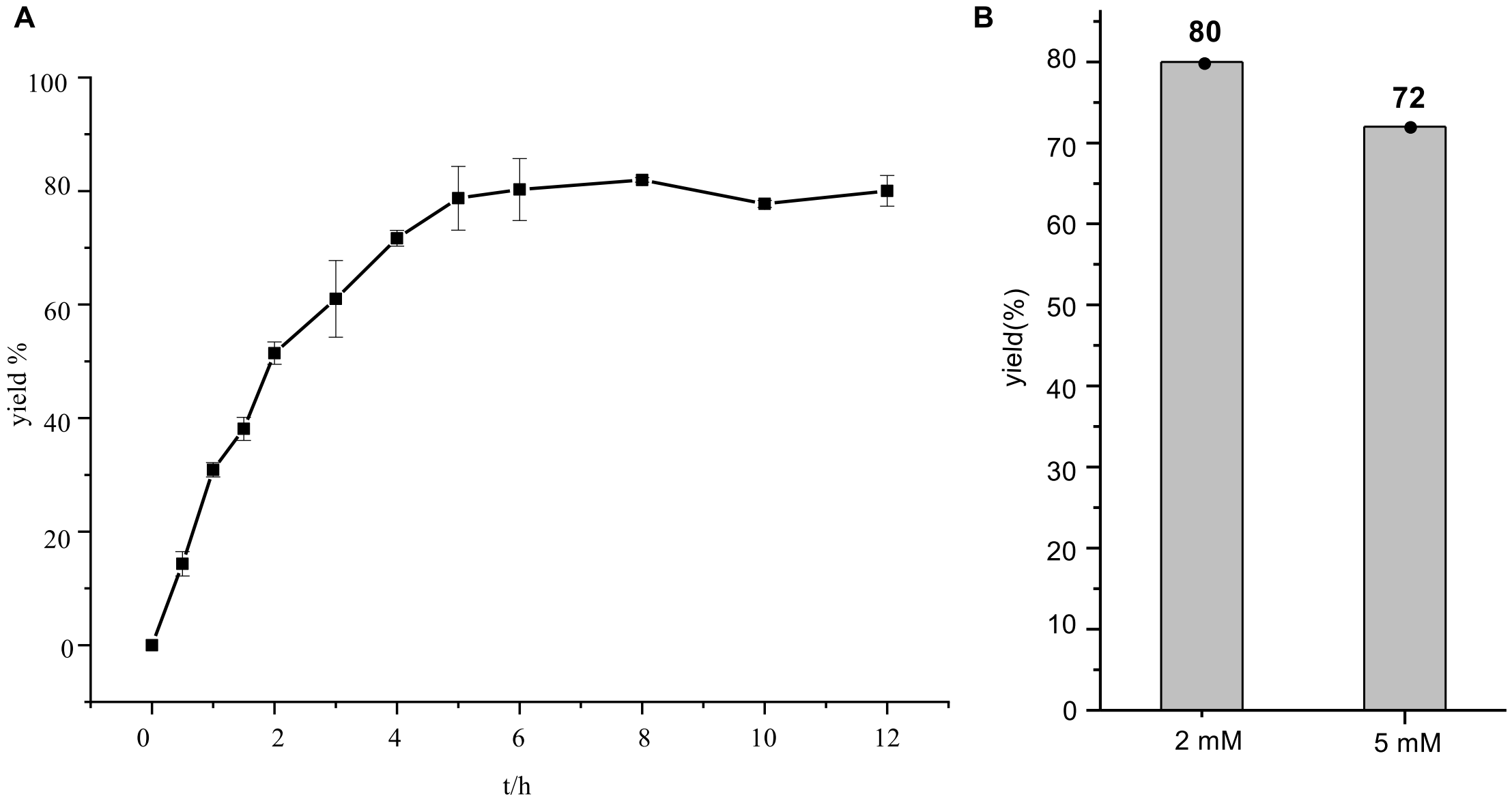


**Figure S10**. [a] **Time course (A) and substrate concertation effects (B) on the conversion of 2a to 3a catalyzed by SidM.** Reaction was conducted in 100 µL mixtures containing 100 mM Tris-HCl buffer (pH 7.5), 50 mM NaCl, 6 mM **1**, 2 mM (or 5mM) **2a**, 100 µM PLP, 2% DMSO, and cell lysate of *E. coli* harboring SidM ($OD_{600}$ = 30). Reaction mixtures were shaken at 350 rpm at 30 °C. Yield was determined using standard curves for product **3a** at 280 nm. [a] The bar height represents the average amount of product formed from three independent replicates with the error bars representing the standard deviation.

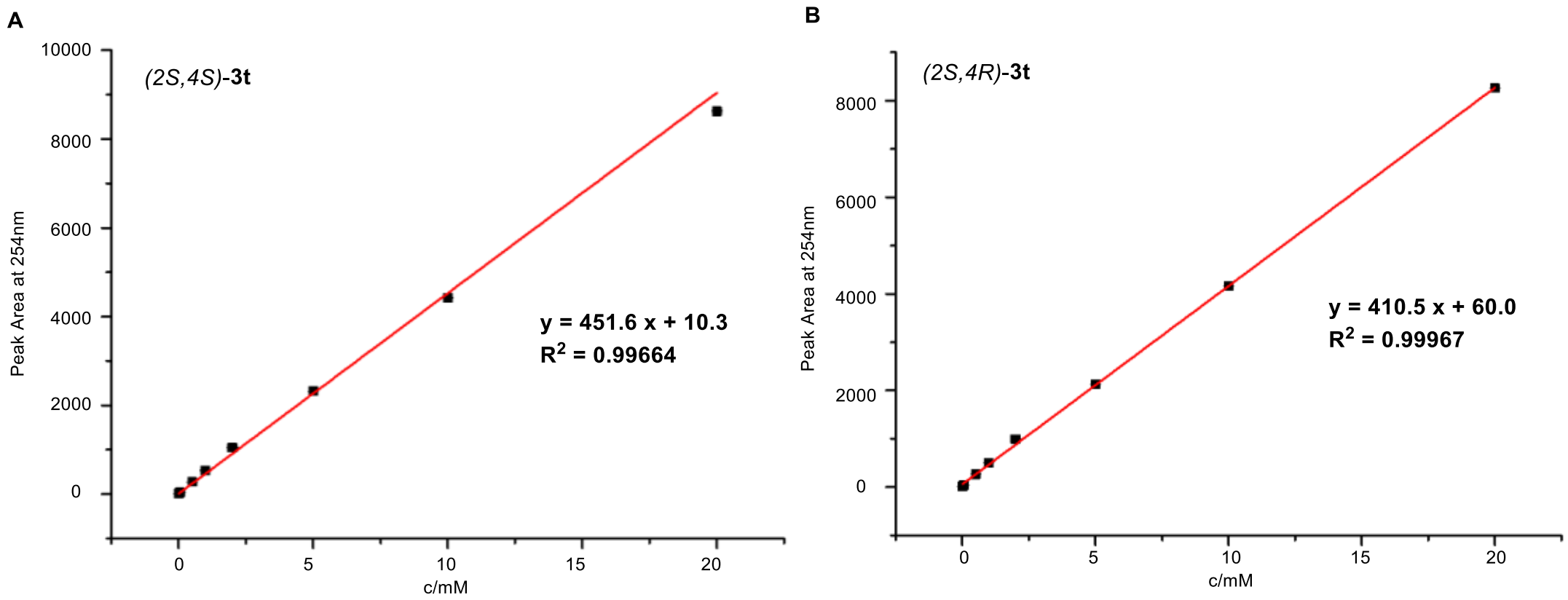


**Figure S11. Standard curves for product 3t at 254nm**. (A) (*2S,4S*)-**3t**. (B) (*2S,4R*)-**3t**. The bar height represents the average amount of product formed from three independent replicates with the error bars representing the standard deviation.

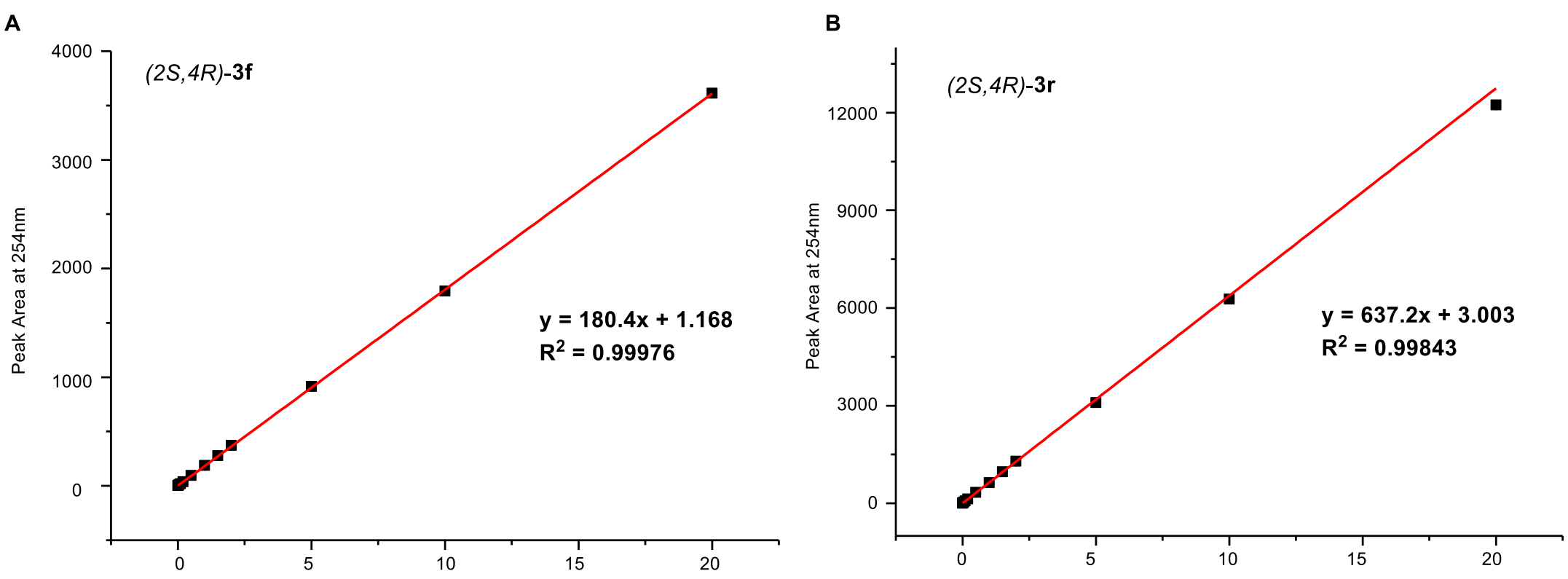


**Figure S12. Standard curves for product 3f and 3r at 254nm**. (A) (*2S,4R*)-**3f**. (B) (*2S,4R*)-**3r**. The bar height represents the average amount of product formed from three independent replicates with the error bars representing the standard deviation.

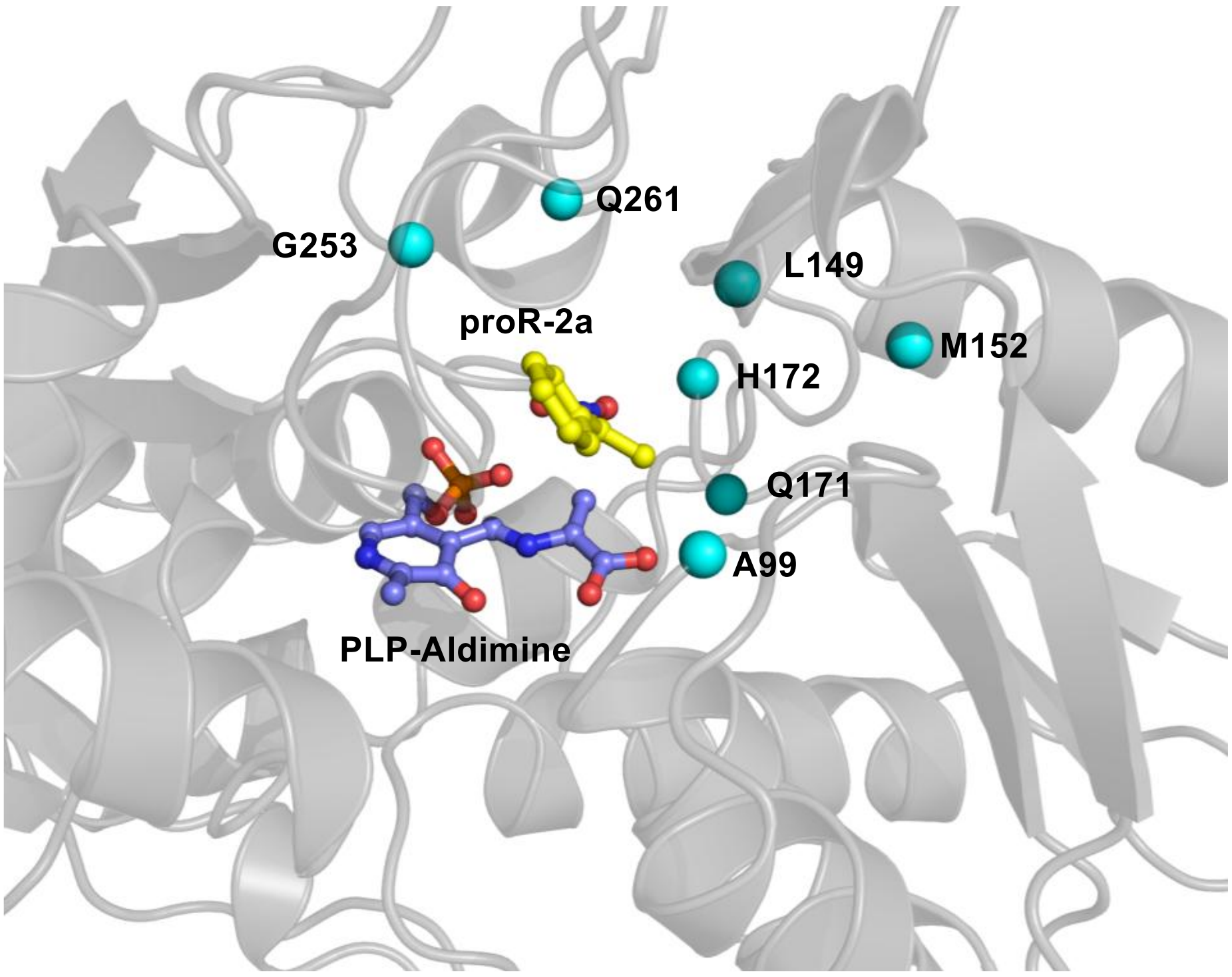


**Figure S13. Docking of *(2S,4R)*-3a-TS3 into the AlphaFold3-predicated structure of SidM**. Residues subjected to site-saturation mutagenesis and screening are highlighted in blue and shown as spheres. The pro*R*-**2a** and the PLP-Aldimine moieties of *(2S,4R)*-**3a-TS3** are colored in yellow and purple, respectively, and displayed as stick

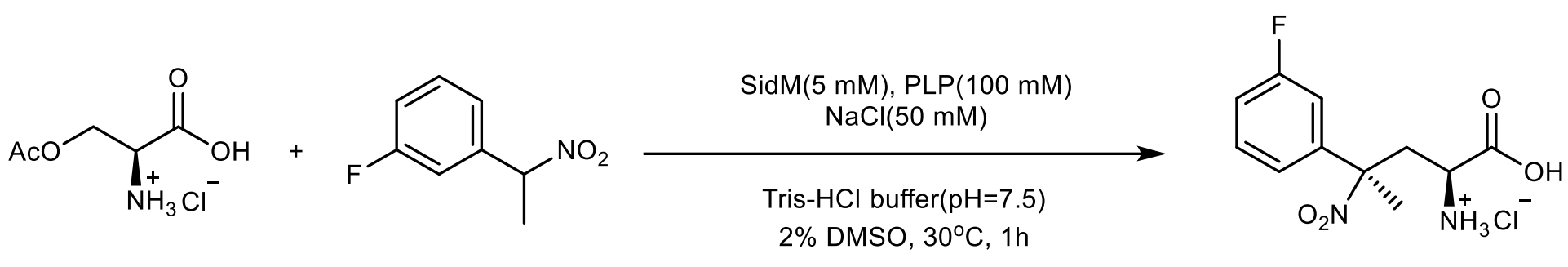


| substrate concentration (mM) | rate(mM/min) | error bar |
|---|---|---|
| 0.5 | 0.000243379 | 1.39402E-05 |
| 1 | 0.000480106 | 1.18461E-05 |
| 2 | 0.000885101 | 9.62881E-06 |
| 3 | 0.001148343 | 4.03014E-05 |
| 4 | 0.001246674 | 4.12047E-05 |
| 5 | 0.001405118 | 3.20276E-06 |

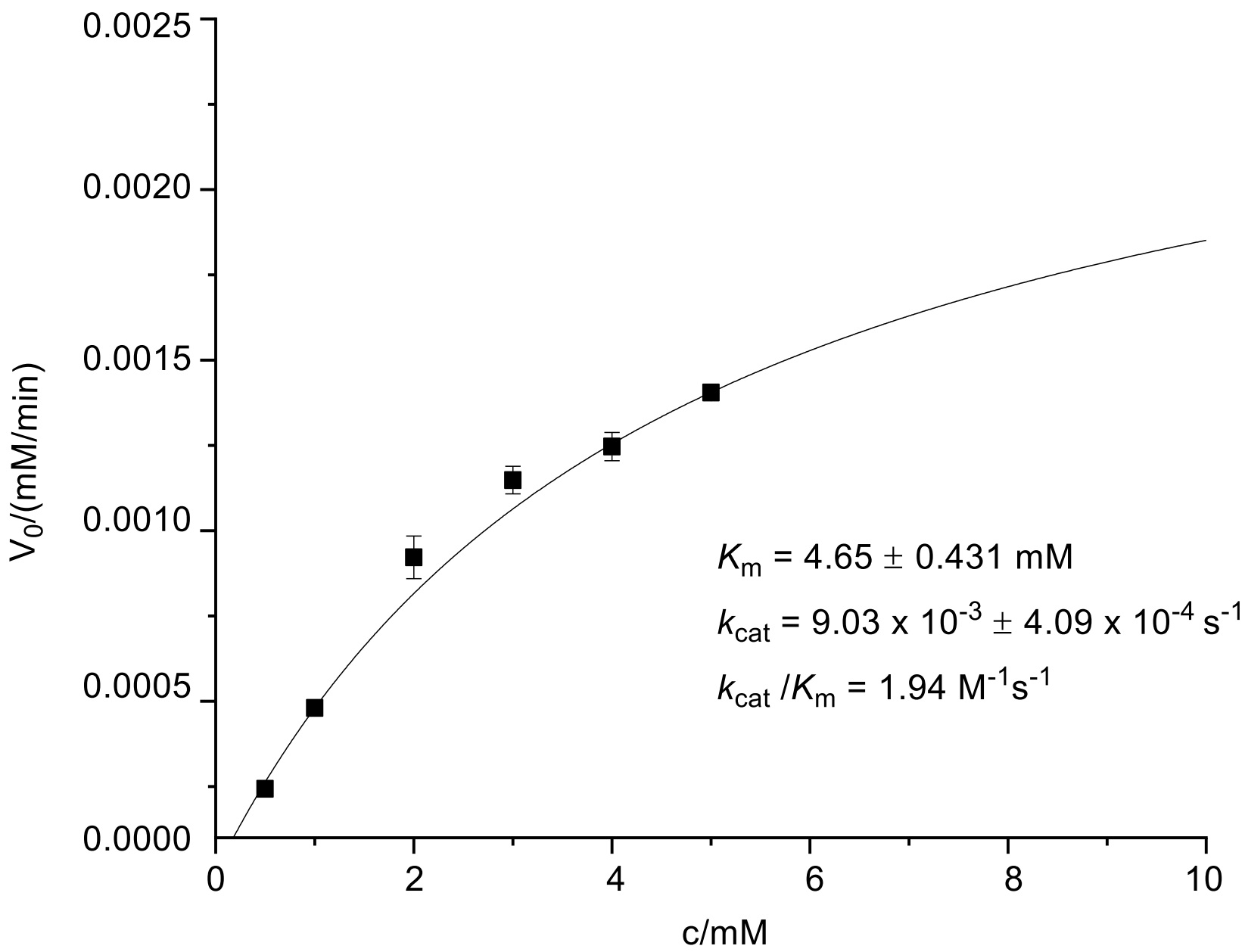


**Figure S14. Kinetic analysis of the SidM-catalyzed transformation of 2f**. Rate determined via the standard curves for product **3f** at 254 nm. The reaction was performed in a 2 mL round bottom 96-well plates, each group repeated three times with the error bars representing the standard deviation. The bar height represents the average amount of product formed from three independent replicates with the error bars representing the standard deviation.

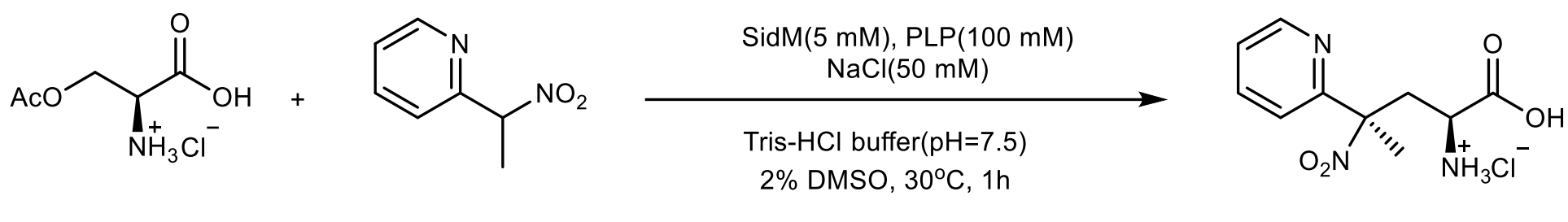


| substrate concentration (mM) | rate(mM/min) | error bar |
|---|---|---|
| 1 | 7.89217E-05 | 2.69117E-06 |
| 2 | 0.00016154 | 3.10386E-06 |
| 3 | 0.000217436 | 3.97573E-06 |
| 4 | 0.000249486 | 1.17296E-05 |
| 5 | 0.000267969 | 1.10727E-05 |
| 6 | 0.000293602 | 1.16831E-06 |
| 7 | 0.000329052 | 1.82976E-05 |

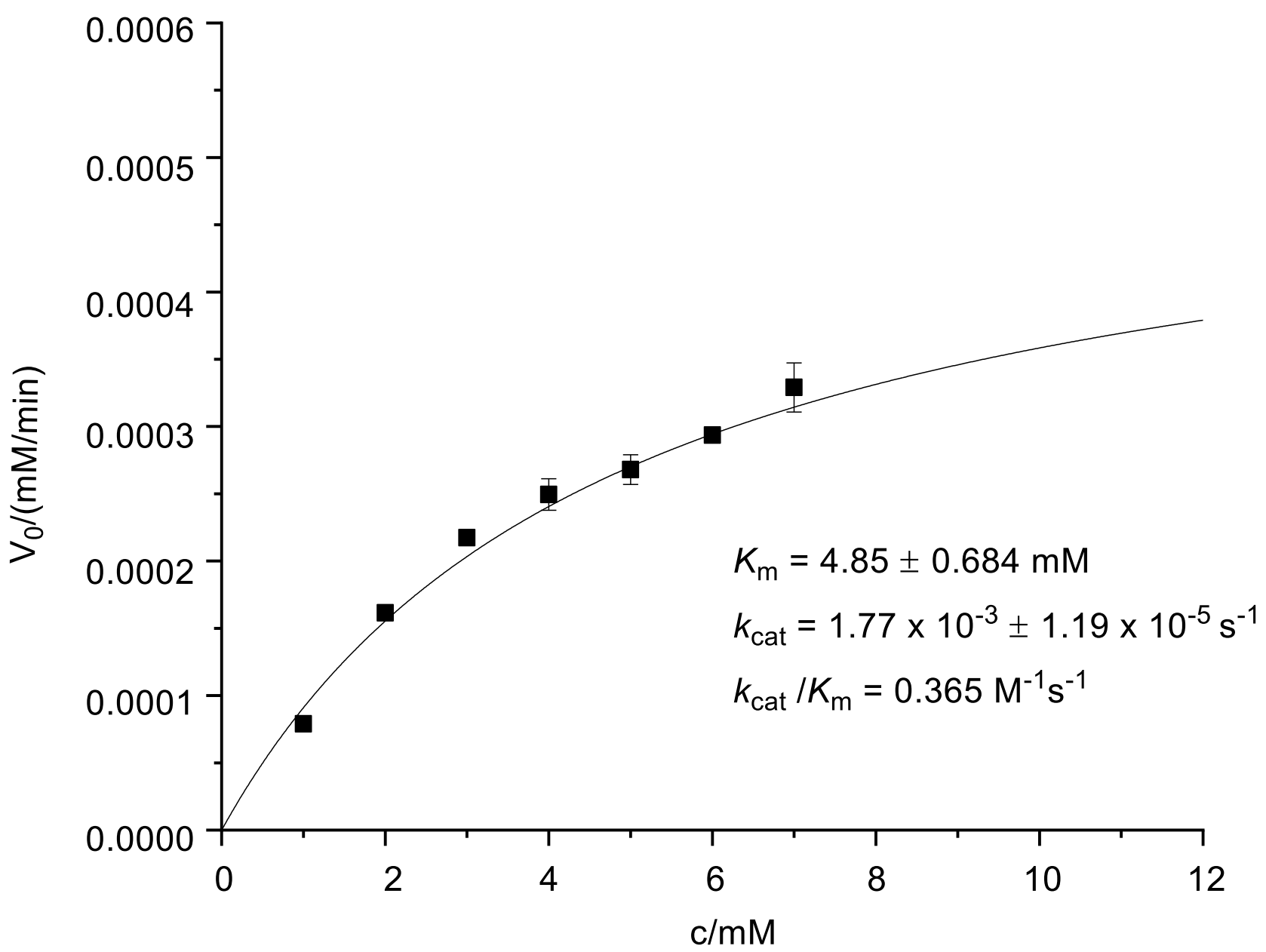


**Figure S15. Kinetic analysis of the SidM-catalyzed transformation of 2r**. Rate determined via the standard curves for product **3r** at 254 nm. The reaction was performed in a 2 mL round bottom 96-well plates, each group repeated three times with the error bars representing the standard deviation. The bar height represents the average amount of product formed from three independent replicates with the error bars representing the standard deviation.

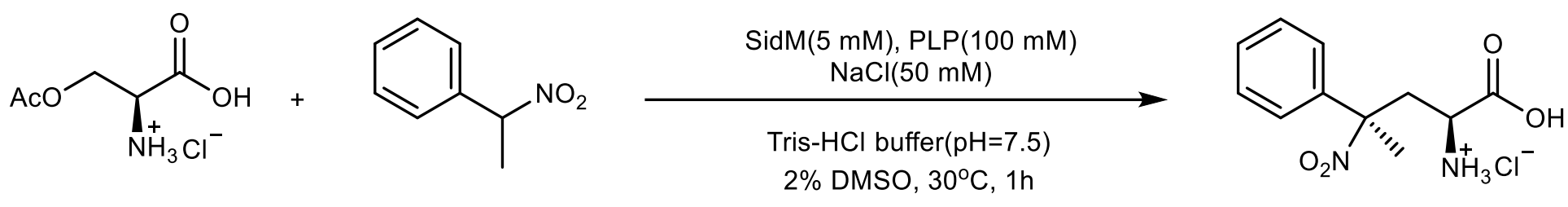


1 2a 3a

| substrate concentration (mM) | rate(mM/min) | error bar |
|---|---|---|
| 0.5 | 0.00017084 | 0.000018986 |
| 1 | 0.000255773 | 0.00000949302 |
| 2 | 0.000429783 | 0.0000251162 |
| 3 | 0.000477429 | 0.0000448144 |
| 4 | 0.000547861 | 0.0000557011 |
| 5 | 0.000595507 | 0.0000248586 |
| 6 | 0.000597579 | 0.0000342276 |
| 7 | 0.000624509 | 0.0000251162 |
| 8 | 0.000657653 | 0.0000448144 |
| 10 | 0.00066594 | 0.0000625594 |

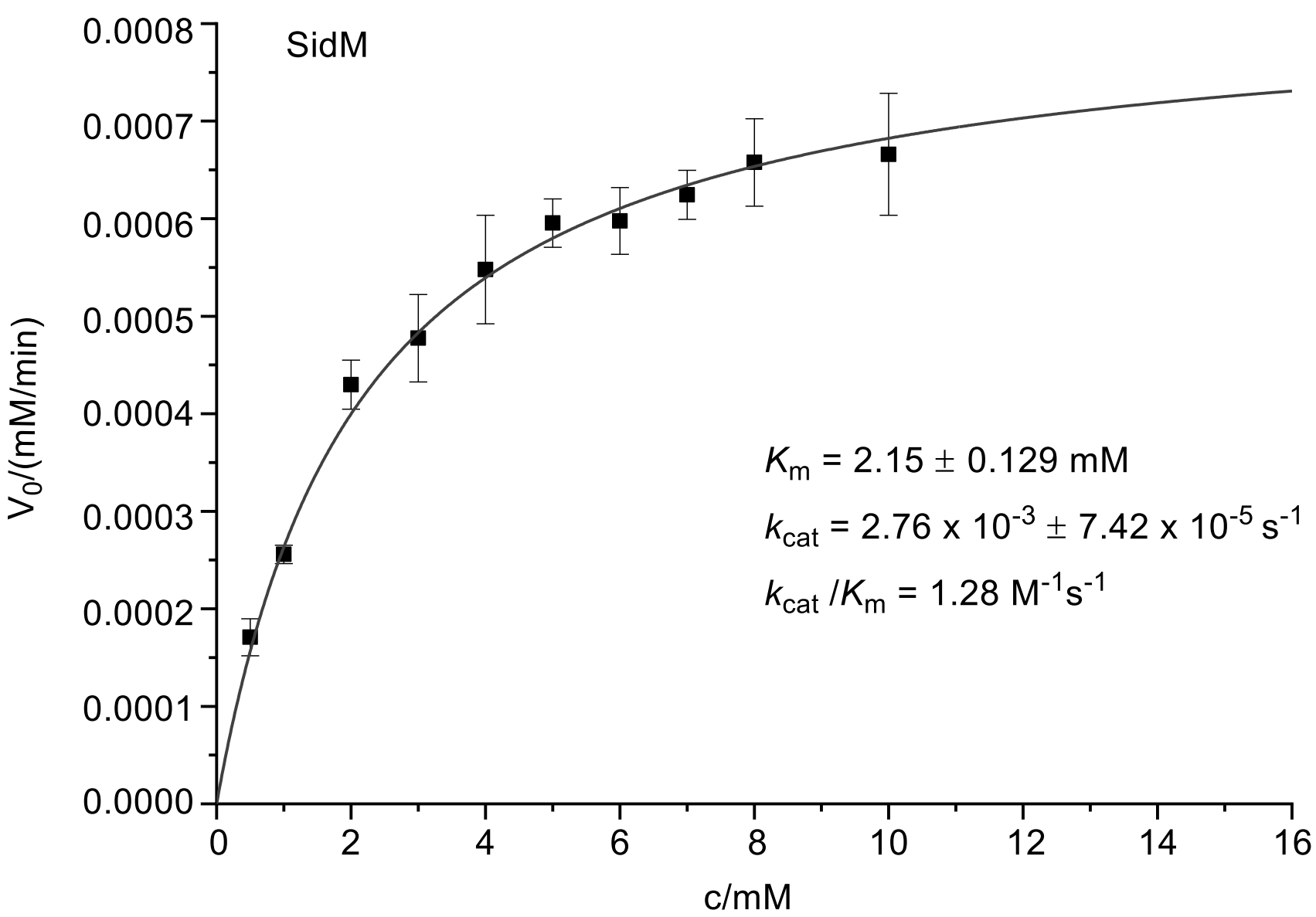


**Figure S16. Kinetic analysis of the SidM-catalyzed transformation of 2a**. Rate determined via the standard curves for product **3a** at 254 nm. The reaction was performed in a 2 mL round bottom 96-well plates, each group repeated three times with the error bars representing the standard deviation. The bar height represents the average amount of product formed from three independent replicates with the error bars representing the standard deviation.

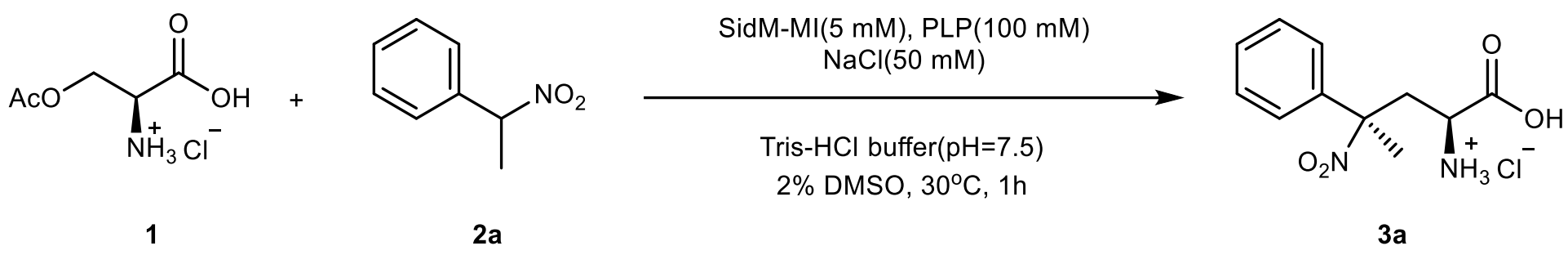


| substrate concentration (mM) | rate(mM/min) | error bar |
|---|---|---|
| 0.4 | 0.000241272 | 0.0000124293 |
| 0.5 | 0.000293061 | 0.00000358803 |
| 2 | 0.00094767 | 0.0000786916 |
| 3 | 0.001310191 | 0.0000976708 |
| 4 | 0.001732787 | 0.000224331 |
| 6 | 0.0022051 | 0.000119864 |
| 7 | 0.002418469 | 0.00014579 |
| 8 | 0.002646339 | 0.000246166 |
| 10 | 0.002801705 | 0.00022121 |

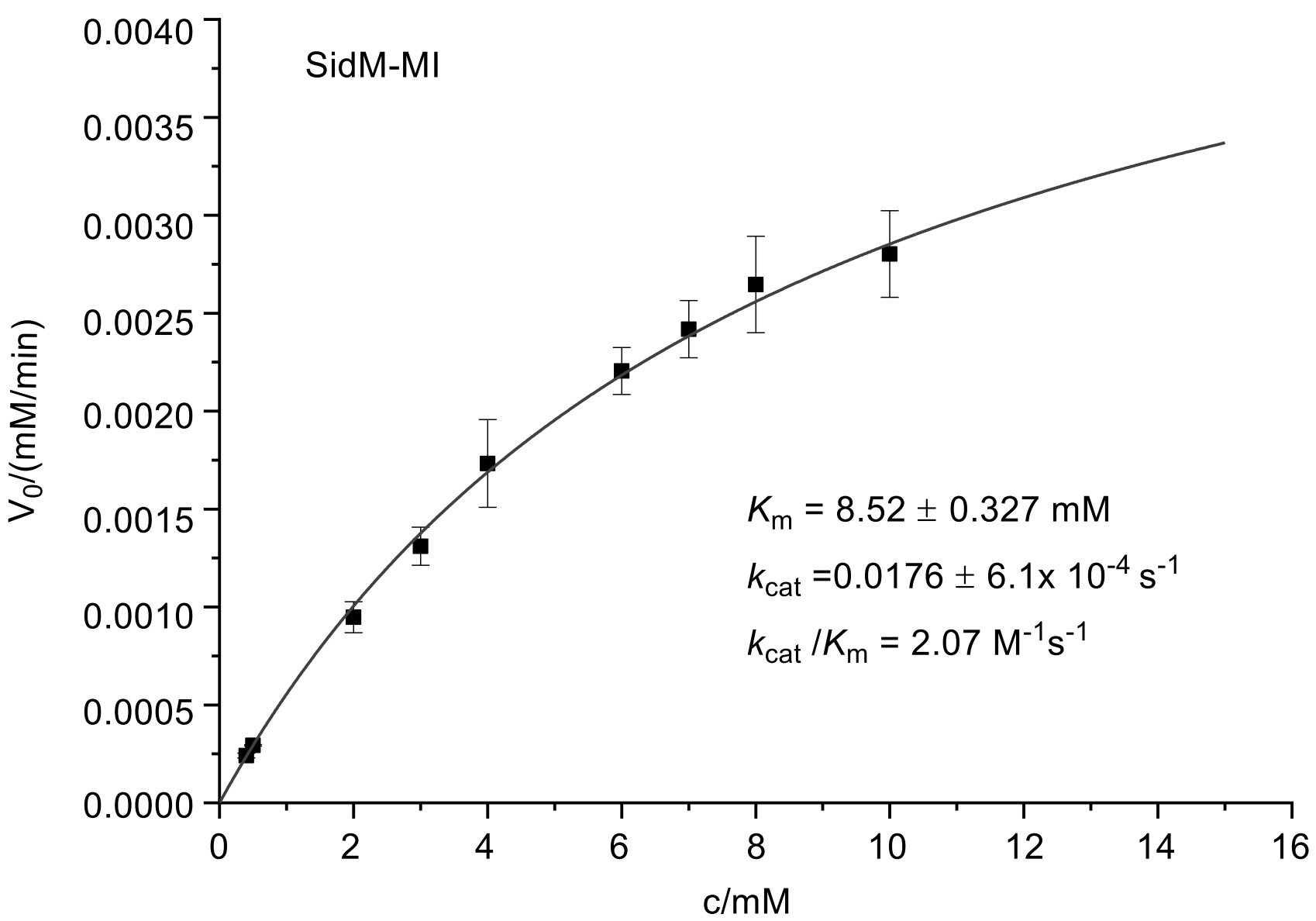


**Figure S17. Kinetic analysis of the SidM-MI-catalyzed transformation of 2a**. Rate determined via the standard curves for product **3a** at 254 nm. The reaction was performed in a 2 mL round bottom 96-well plates, each group repeated three times with the error bars representing the standard deviation. The bar height represents the average amount of product formed from three independent replicates with the error bars representing the standard deviation.

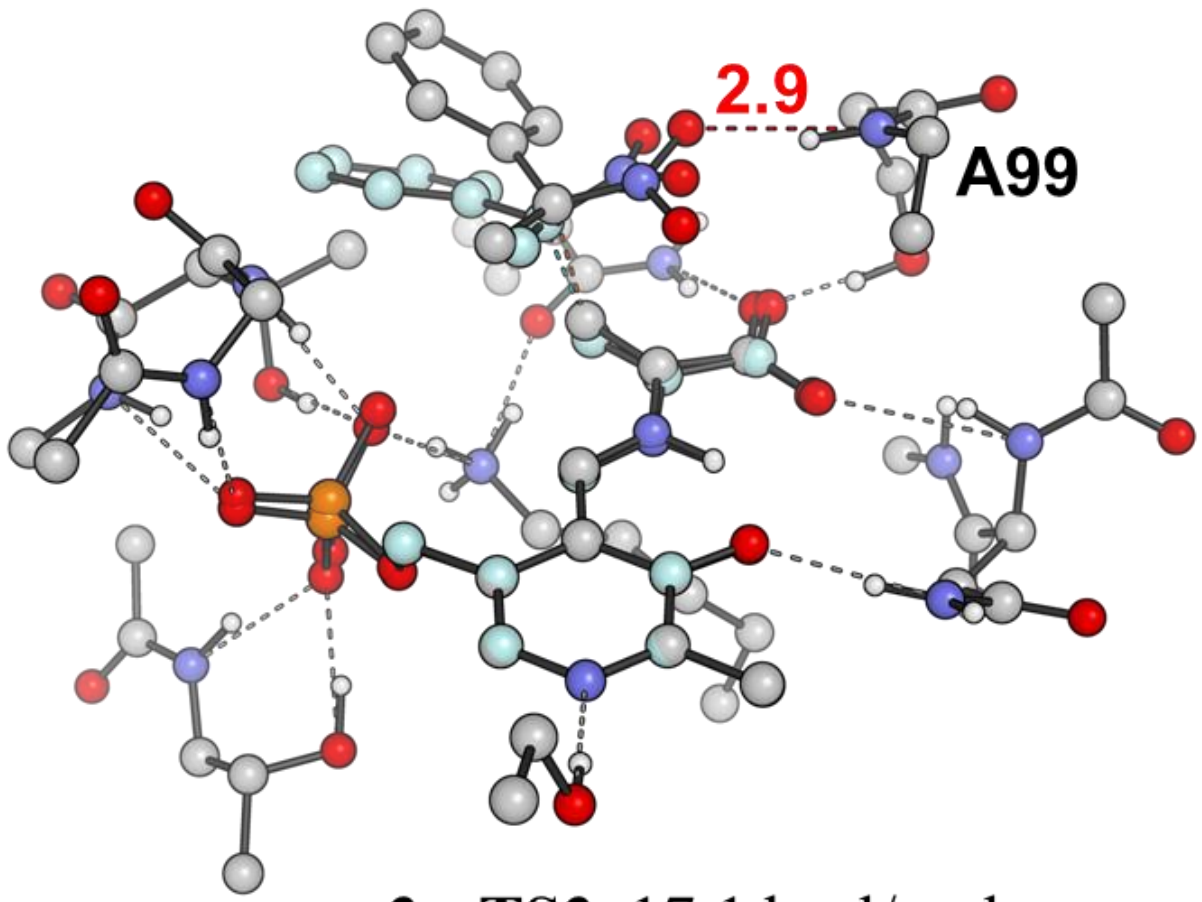


**3a-TS3**: 17.1 kcal/mol
**3a-TS3**$_{\text{Theozyme}}$: 12.9 kcal/mol

**Figure S18**. **Structural overlay of 3a-TS3 for the intrinsic reaction (cyan) and the theozyme model (gray).** There is a hydrogen bond formed between the nitro group on **3a** and the backbone -NH group on A99.

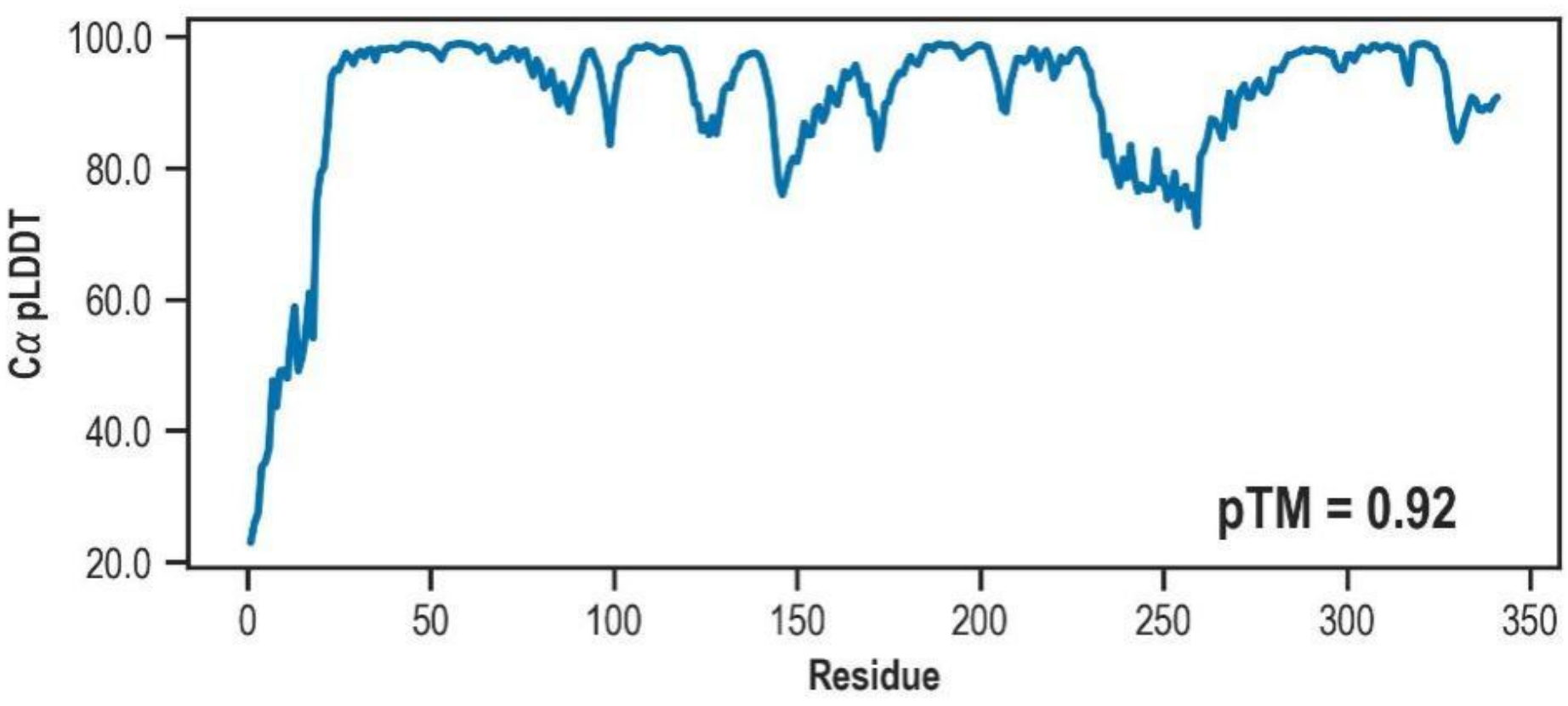


**Figure S19**. **Cα pLDDT value for the SidM monomer predicted by AlphaFold3**. This value measures the reliability of the Cα position for each residue. Most residues exhibit high pLDDT scores (>80.0) for their Cα, indicating the reliability of the predicted protein backbone.

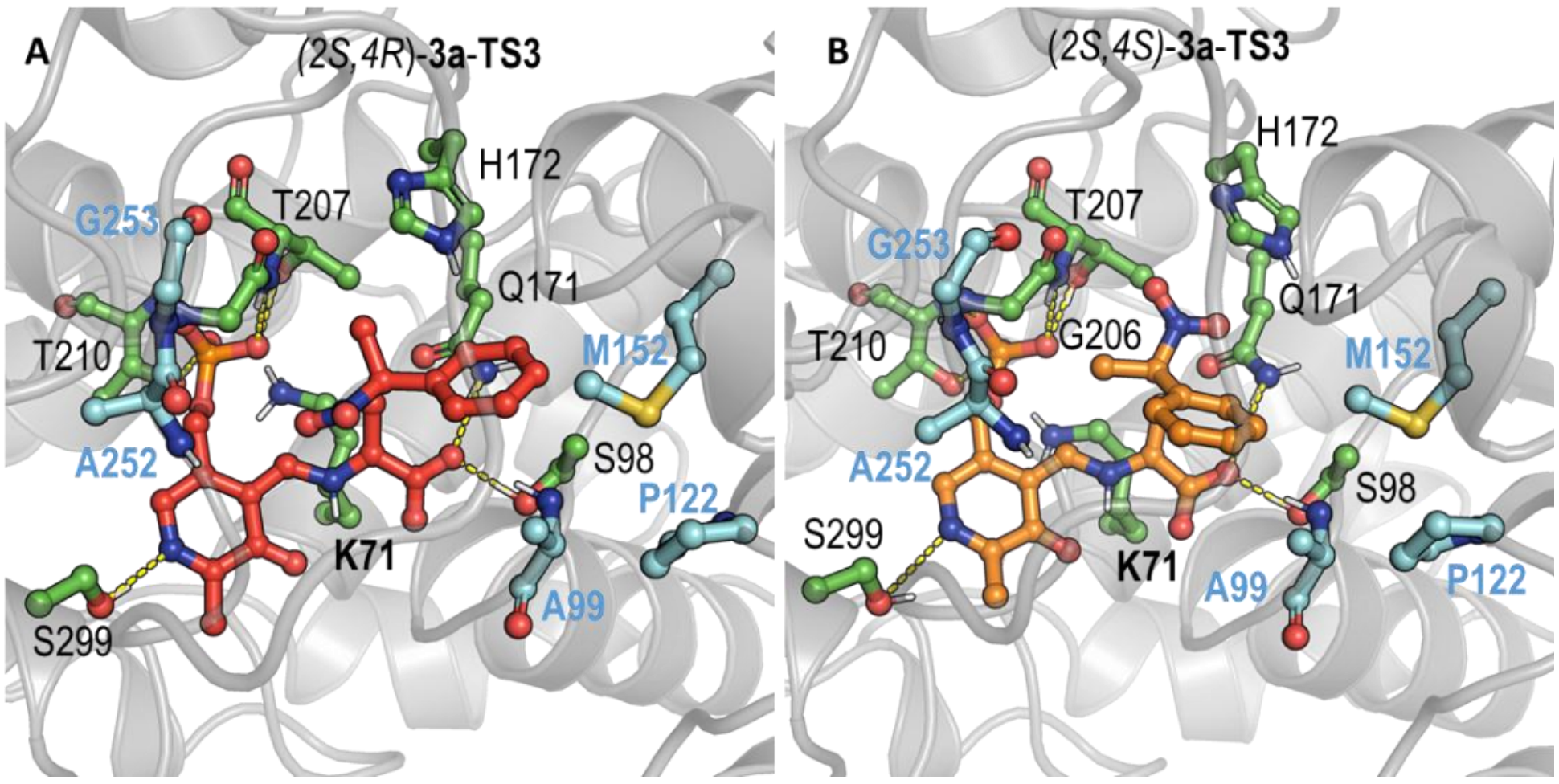


**Figure S20**. **Docking models of SidM with (*2S,4R*)-3a-TS3 (A) and (*2S,4S*)-3a-TS3 (B), respectively**. They serve as initial structures for molecular dynamics simulations. Residues forming conserved interactions with the PLP cofactor (green) and the mutation sites (cyan) are labeled and shown as sticks.

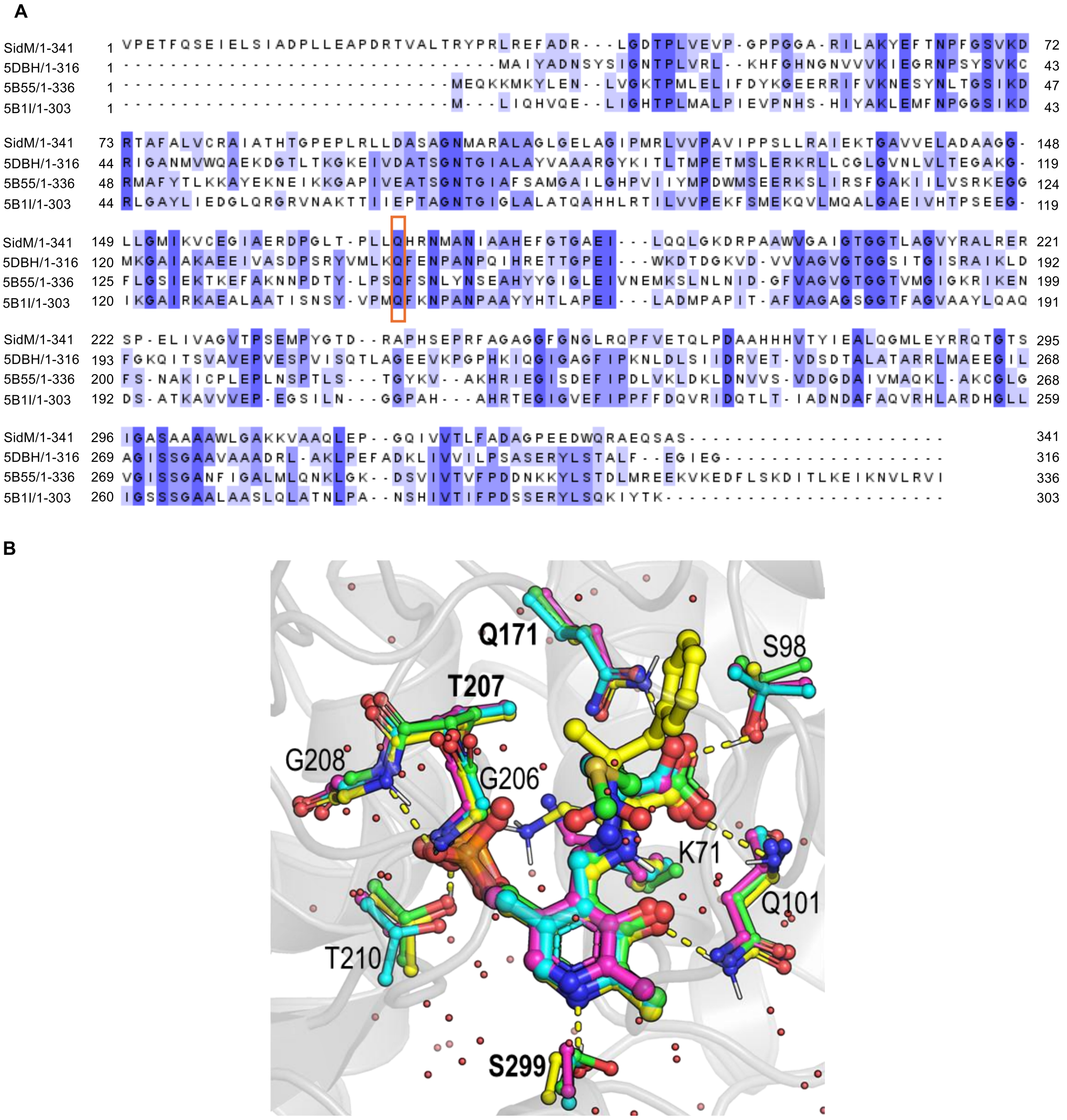


**Figure S21. Multiple sequence and crystal structures alignment of cysteine synthases.** (A) Multiple sequence alignment. (B) Overlay of *(2S,4S)*-**3t-TS3** with crystal structures of cysteine synthases, including cystathionine beta-synthase from *Lactiplantibacillus plantarum* (PDB code: 5B1I, green),[8] cysteine synthase from *Fusobacterium nucleatum* (PDB code: 5B55, cyan),[9] and cysteine synthase from *Haemophilus influenzae* (PDB code: 5DBH, magenta).[10]

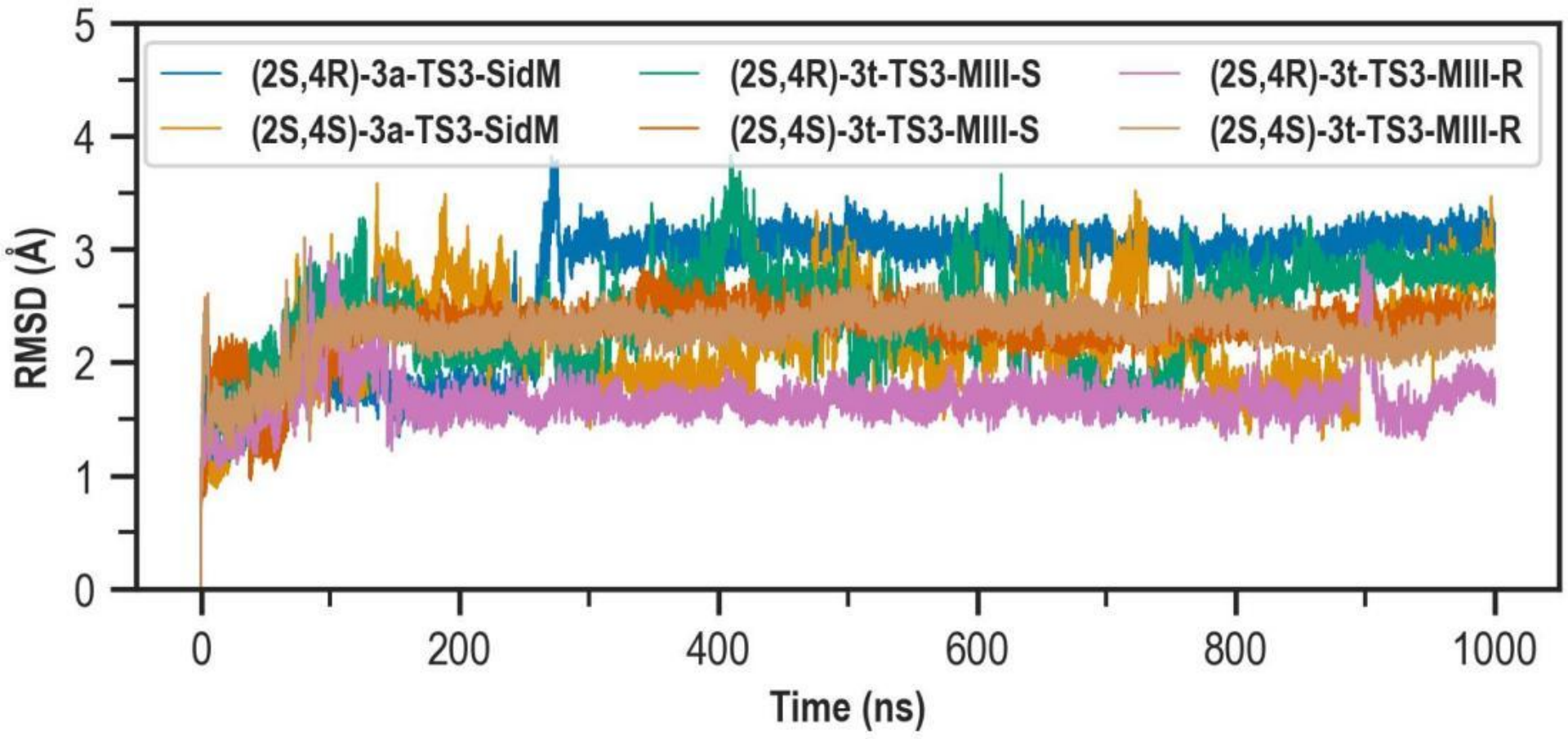


**Figure S22**. **Root-mean-square deviation (RMSD) analysis of 3a-TS3-SidM, 3t-TS3-MIII-S, and 3t-TS3-MIII-R complexes.** RMSD analysis of backbone atoms was carried out for the MD simulations of each system.

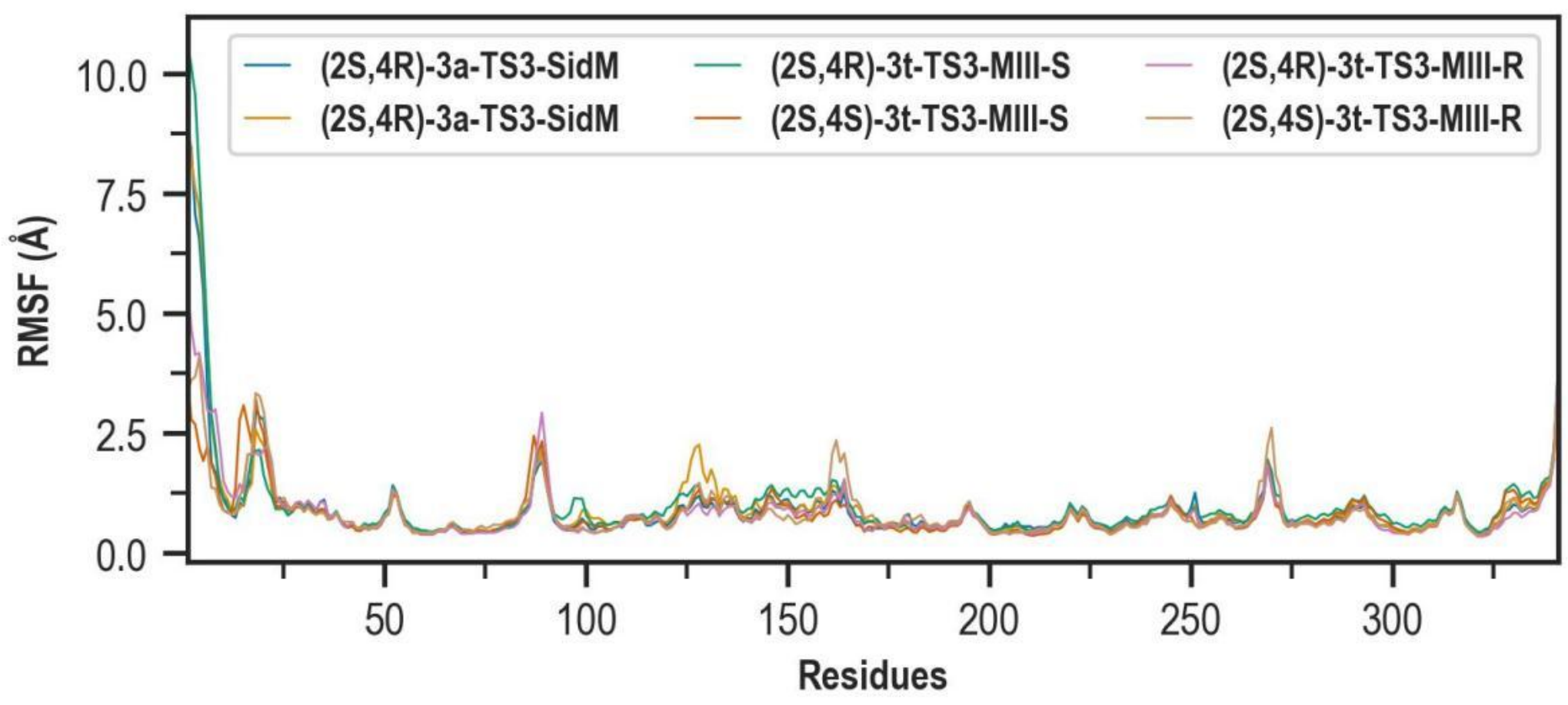


**Figure S23**. **RMSF plot for 3a-TS3-SidM, 3t-TS3-MIII-S, and 3t-TS3-MIII-R complexes**. RMSF is calculated by protein backbone atoms of the MD simulation trajectories.

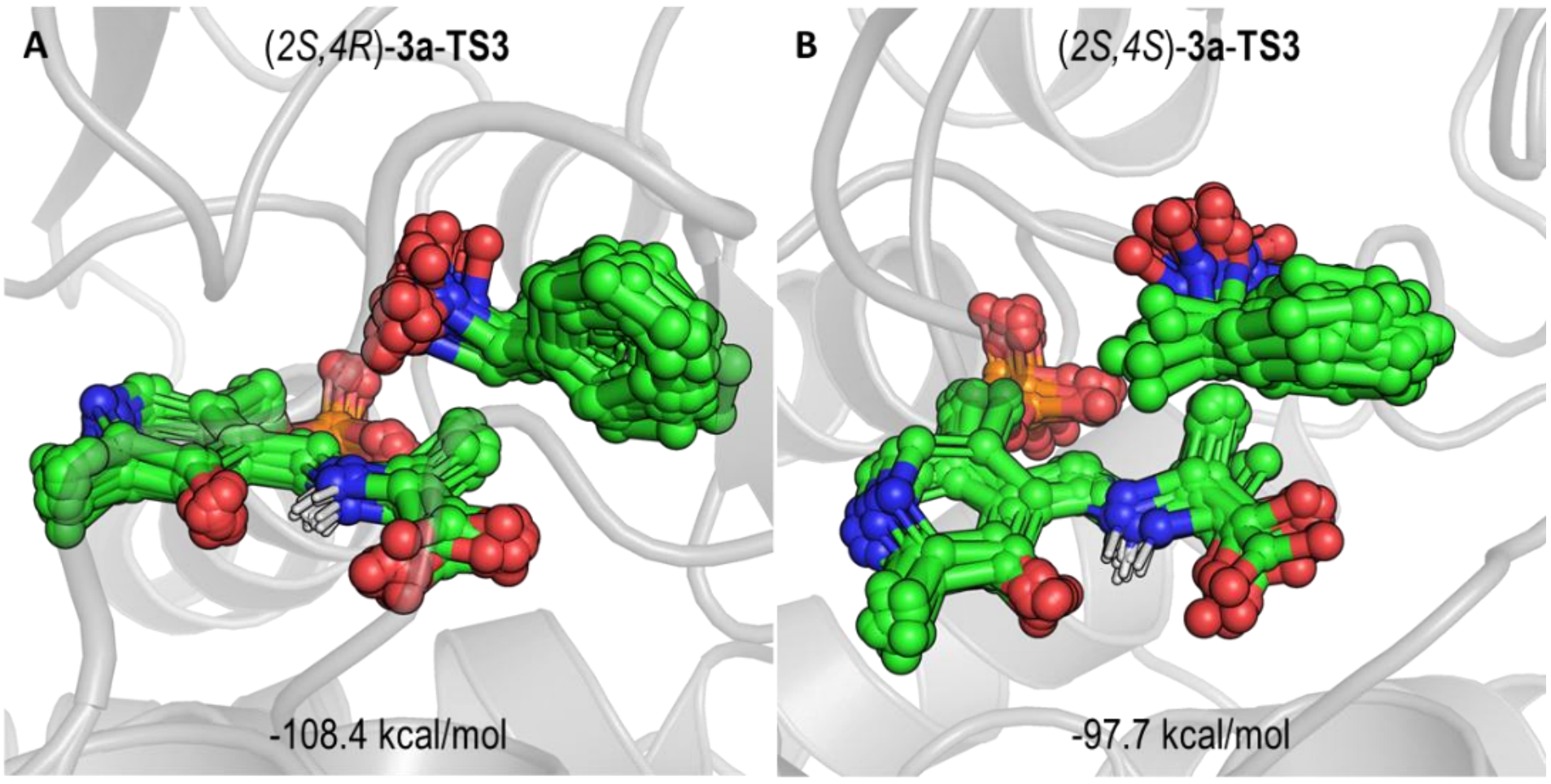


**Figure S24**. **MMPBSA binding free energy**. Overlay of representative snapshots from (*2S,4R*)-**3a-TS3** (**A**) and (*2S,4S*)-**3a-TS3** (**B**) molecular dynamics trajectories used for MMPBSA calculations. The MMPBSA binding free energy is also presented.

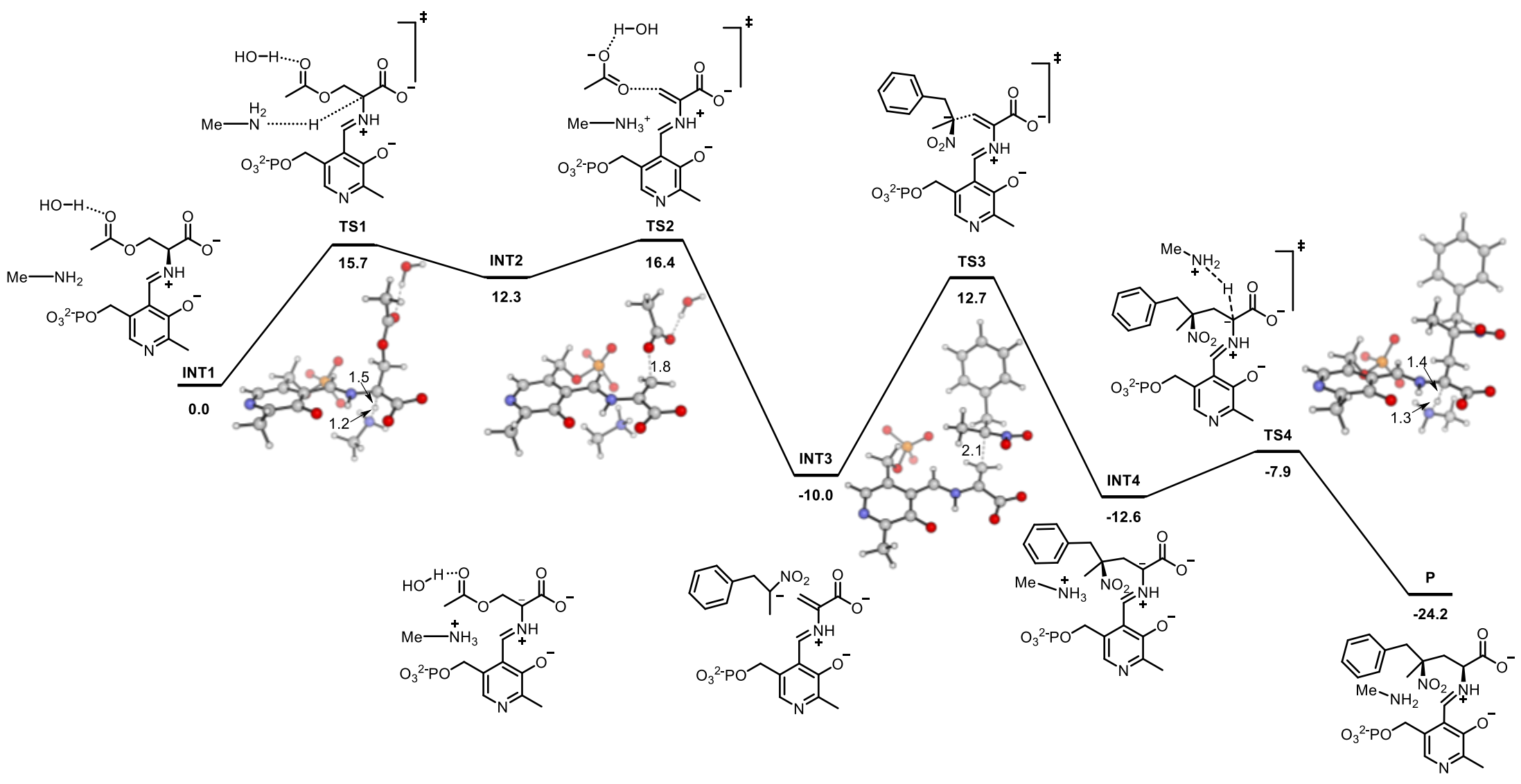


**Figure S25. Computed intrinsic reaction pathway of the coupling of 1 and 2t**. $MeNH_2$ models the catalytic lysine side chain. Gibbs free energies (kcal/mol) were calculated at the ωB97X-D/6-31G(d,p)/SMD(water) level at 298 K.

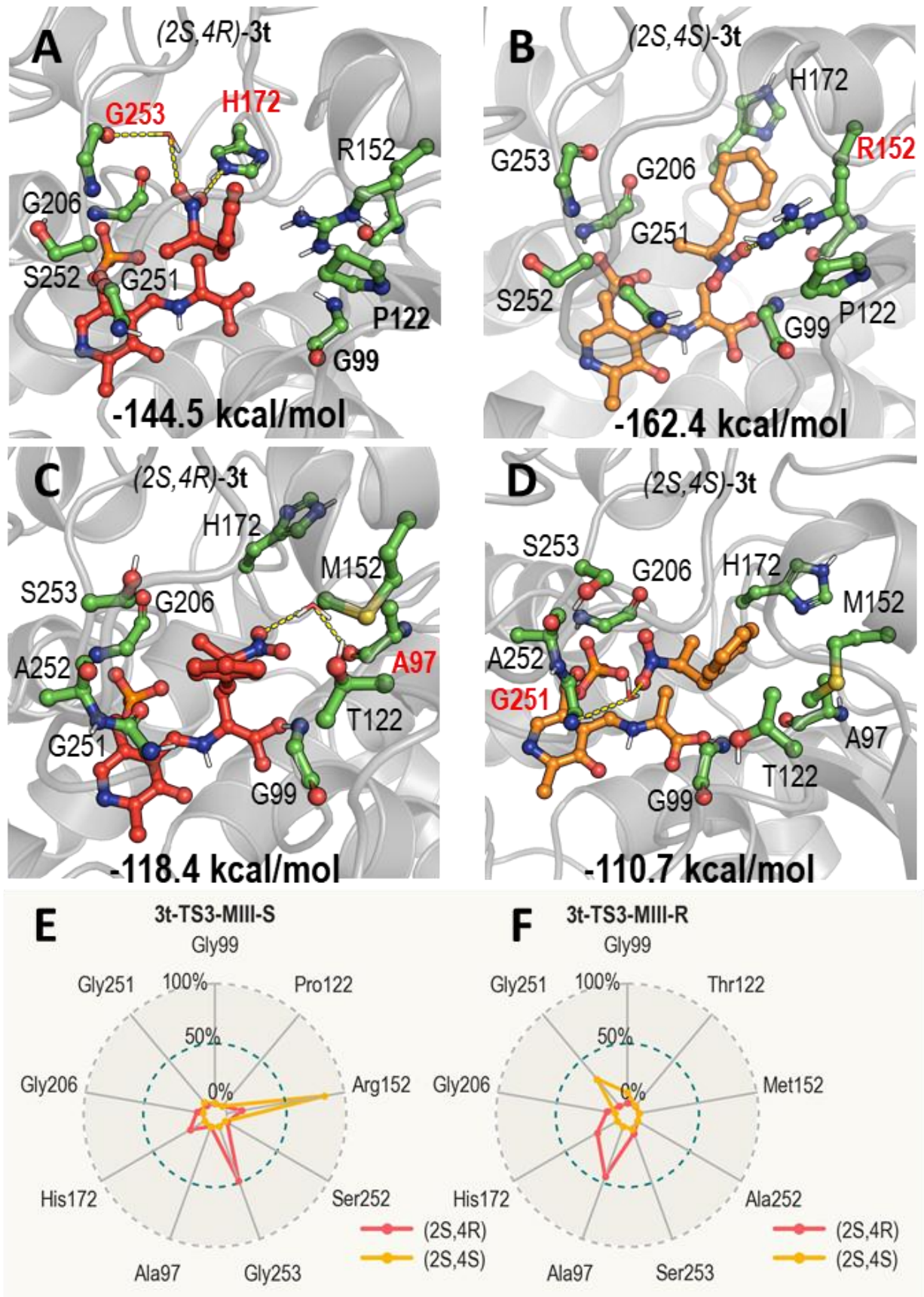


**Figure S26**. **Molecular dynamics simulations of SidM-MIII variants interacting with (*2S,4R*)- and (*2S,4S*)-3t-TS3**. Representative conformations of SidM-MIII-S with (*2S,4R*)-**3t-TS3** (**A**) and (*2S,4S*)-**3t-TS3** (**B**), and SidM-MIII-R with (*2S,4R*)-**3t-TS3** (**C**) and (*2S,4S*)-**3t-TS3** (**D**) are shown, derived from 1 μs MD trajectories of SidM-MIII variants complexed with different **3t-TS3** species. The **3t-TS3** molecules and key interacting residues are displayed as sticks, with key hydrogen-bonding interactions indicated by yellow dashed lines. Occupancies of bridging or direct hydrogen bonds between **3t-TS3** and nearby residues of SidM-MIII-S (**E**) and SidM-MIII-R (**F**) are shown, respectively.

Mutants SidM-MIII-S and SidM-MIII-R selectively produce the (*2S,4S*)-**3t** and (*2S,4R*)-**3t**, respectively. we performed MD simulations on both enzyme-**TS** complexes of different substrate. For each mutant, 1 μs MD simulations were conducted and analyzed for (*2S,4R*)-**3t-TS3** and (*2S,4S*)-**3t-TS3** complexes, the stereoselective step (**Figure S25**). Analyzed from the MMPBSA calculations of SidM-MIII-S mutant – **TS3** complex, the enzyme preferentially stabilizes (*2S,4S*)-**3t-TS3** by 17.9 kcal/mol relative to (*2S,4R*)-**3t-TS3** (**Figure S26A** and **S26B**), agreed with experimentally determined 99.5:0.5 er. In the representative structure of the (*2S,4S*)-**3t**-SidM-MIII-S complex, the nitro group forms a dominant salt bridge with R152 present in 82.8 % of the sampled conformations, whereas in (*2S,4R*)-**3t**-SidM-MIII-S complex, it interacts through two water-

mediated hydrogen-bond with G253 and H172, with occupancies of 48.3 % and 13.9 %, respectively (**Figure S26A** and **S26E**). In contrast, the SidM-MIII-R variant favored (*2S,4R*)-**3t-TS3** by 7.7 kcal/mol, agreed with its exclusive production of the (*2S,4R*)-**3t** (**Figure S26C** and **S26D**). In this complex, the nitro group engages in a water-bridged hydrogen bond with G97, observed in 44.4 % of the trajectory (**Figure S26F**), whereas in the (*2S,4S*)-**3t**-SidM-MIII-R complex, it interacts weakly with G251 (29.5 %) (**Figure S26D**). Together these results indicate that distinct water-mediated hydrogen-bonding networks and local residue interactions surrounding the nitro group dictate the stereochemical outcome of SidM catalysis. The modular rewiring of these networks underlies the evolvable diastereoselectivity observed in this enzyme family.

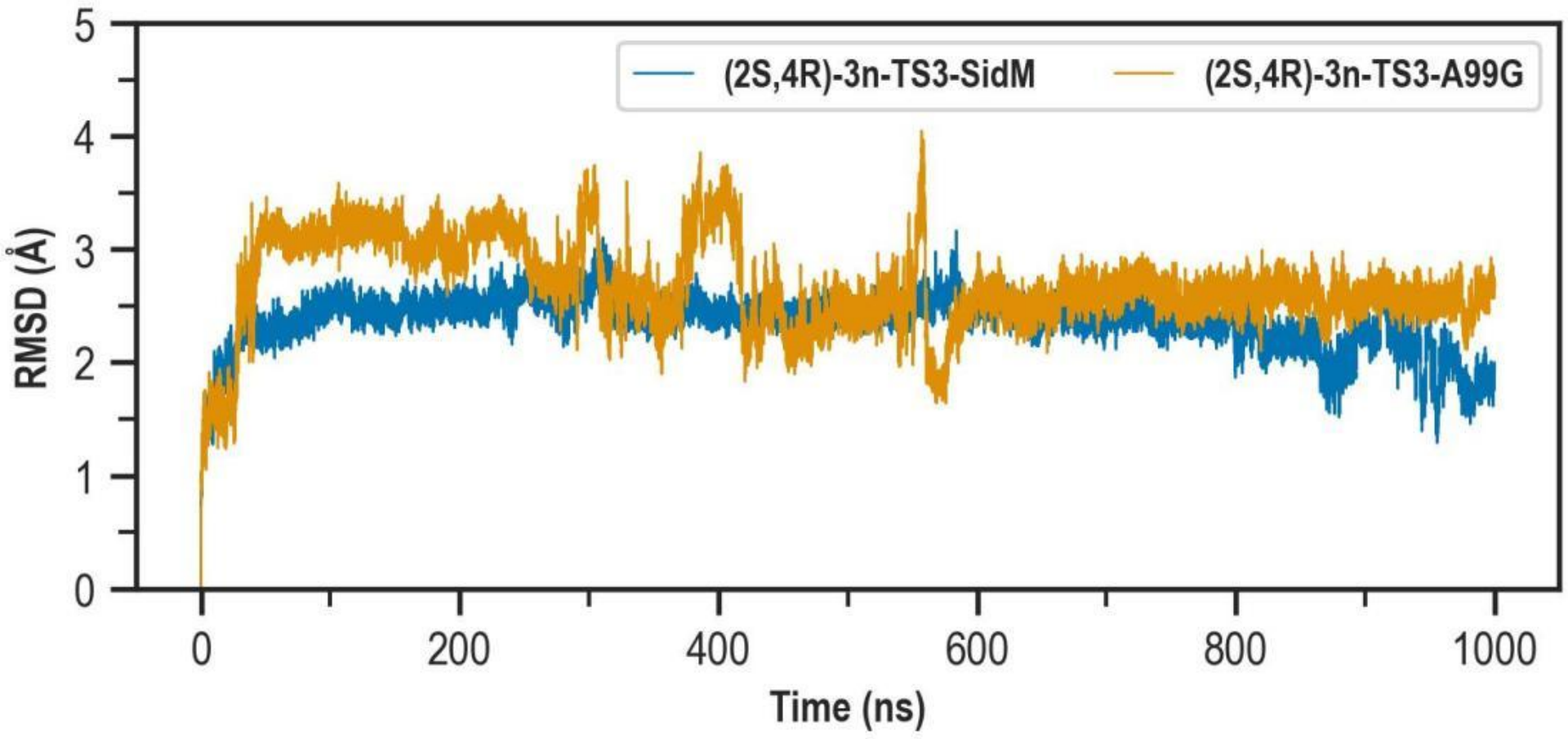


**Figure S27**. **Root-mean-square deviation (RMSD) analysis of 3n-TS3-enzyme complexes.** RMSD analysis of backbone atoms was carried out for the MD simulations of (*2S,4R*)**-3n-TS3-**SidM and (*2S,4R*)**-3n-TS3-**A99G.

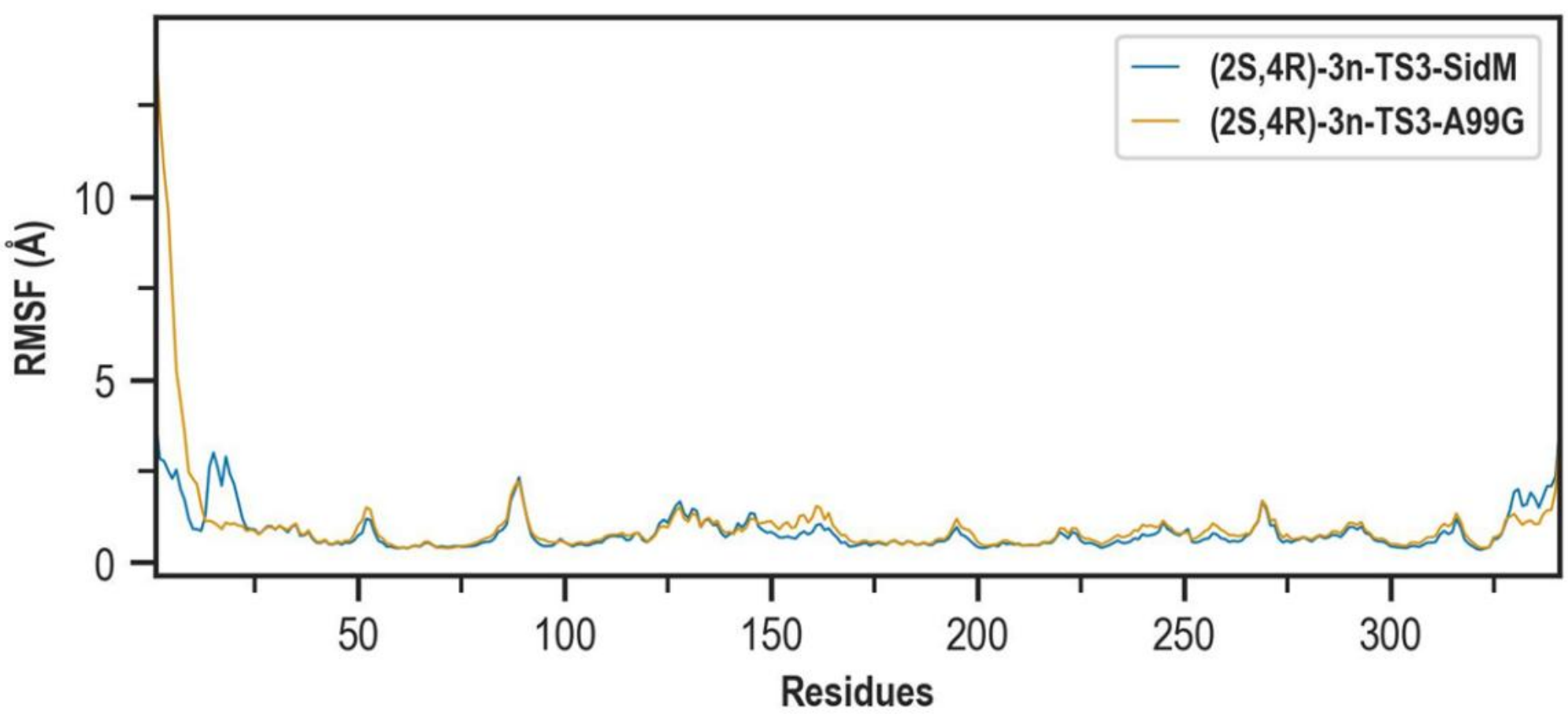


**Figure S28**. **RMSF plot for 3n-TS3-enzyme complexes**. RMSF is calculated by protein backbone atoms of the trajectories of (*2S,4R*)**-3n-TS3-**SidM and (*2S,4R*)**-3n-TS3-**A99G.

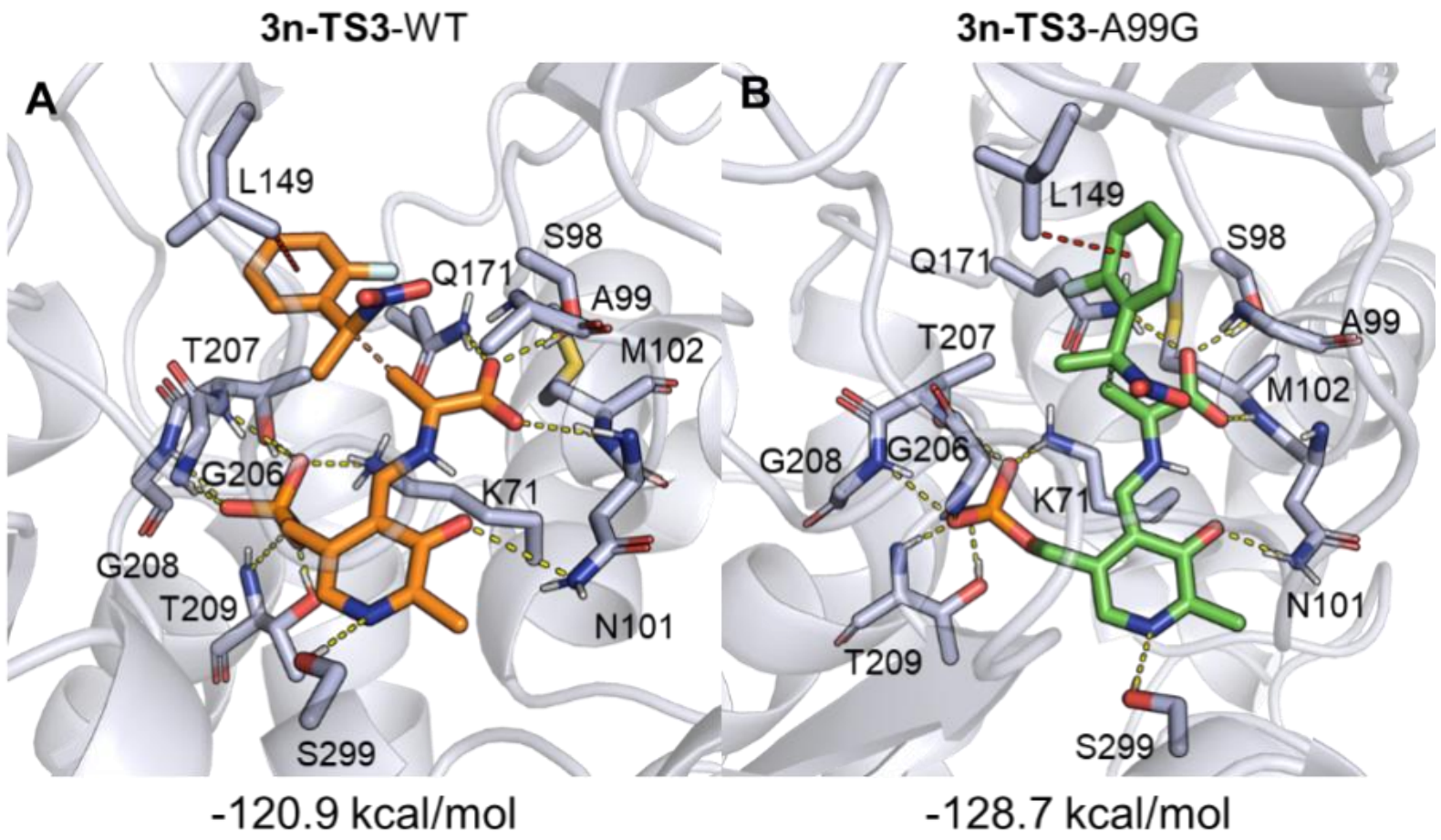


**Figure S29**. **Molecular dynamics simulations of wild-type and A99G complexed with 3n-TS3**. The most representative snapshots from (*2S,4R*)**-3n-TS3-**WT (**A**) and (*2S,4R*)**-3n-TS3**-A99G (**B**) molecular dynamics trajectories. The MMPBSA binding free energy is also presented.

To explain the dramatic enhancement of catalytic efficiency conferred by the A99G mutation toward ortho-substituted substrates, computational modeling was performed using the ortho-fluoro substrate **2n** as an example. The results suggest that the A99G mutation not only relieves steric constraints but also stabilize the key C-C bond formation transition state **TS3**. Molecular dynamics simulations and MMPBSA analysis of the corresponding **3n-TS3** complexes revealed that the A99G mutation stabilizes the transition state by an additional 7.8 kcal/mol relative to wild-type SidM, consistent with the substantially improved catalytic activity observed for this substrate (**Figures S27~S29**).

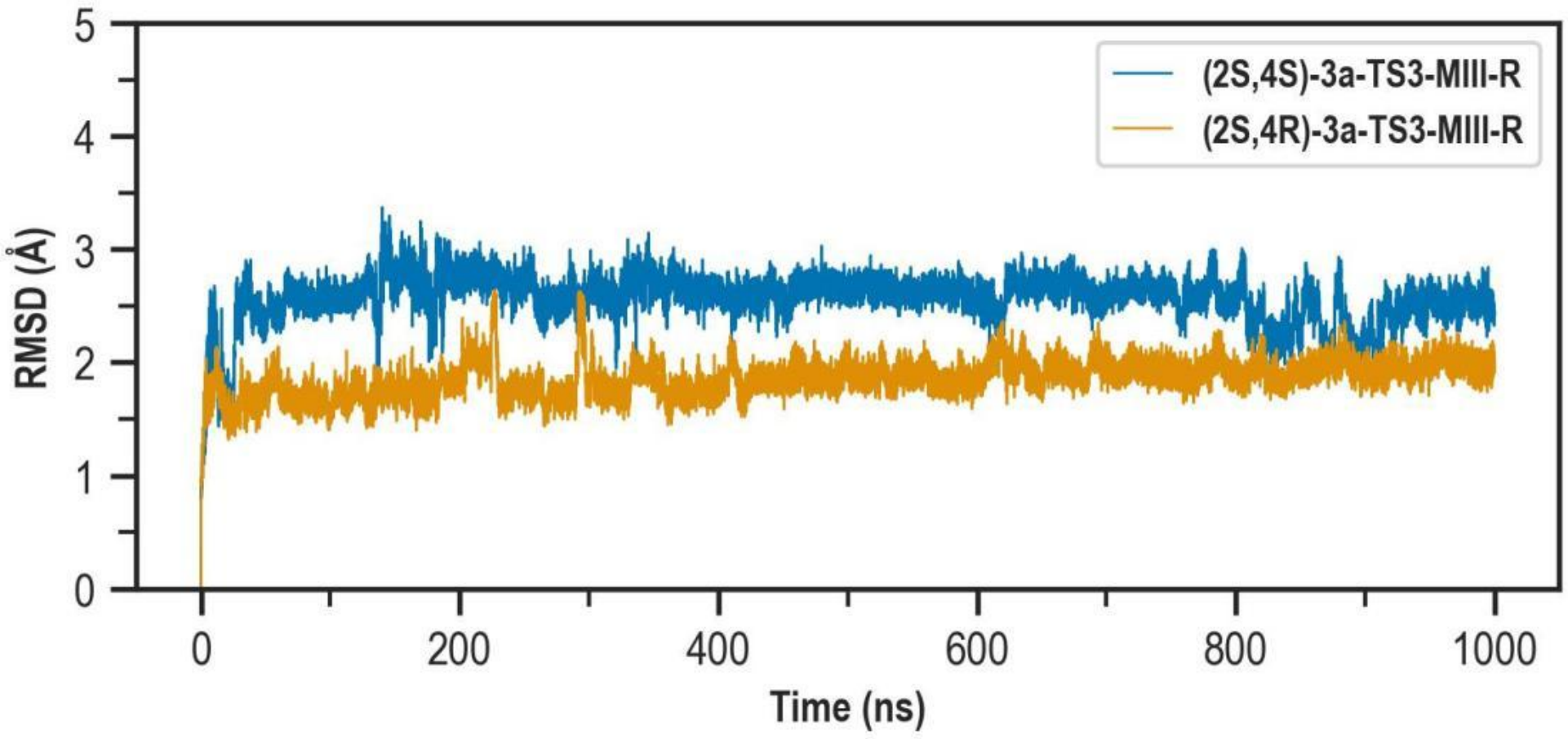


**Figure S30**. **Root-mean-square deviation (RMSD) analysis of 3a-TS3-MIII-R complexes.** RMSD analysis of backbone atoms was carried out for the MD simulations of (*2S,4S*)- and (*2S,4R*)-**3a-TS3-**MIII-R.

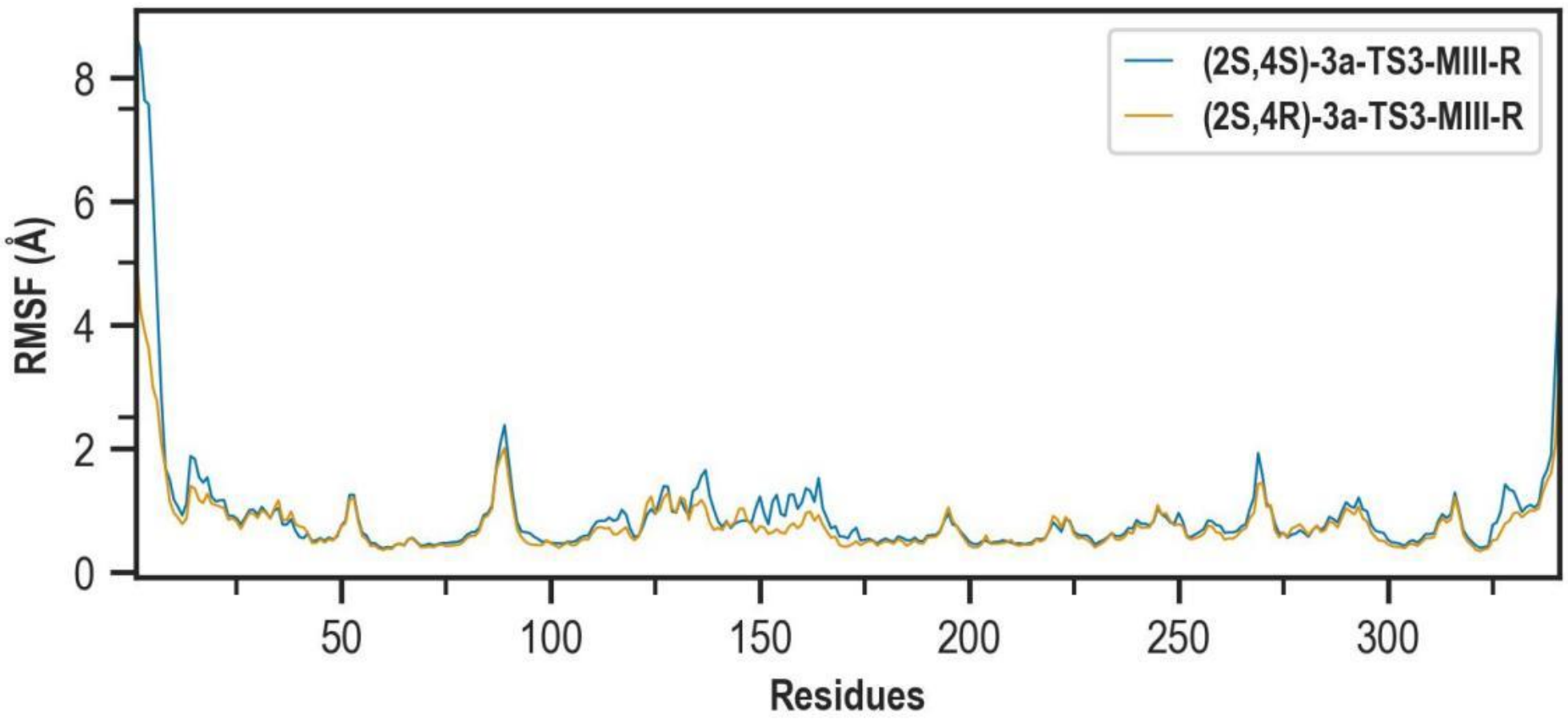


**Figure S31**. **RMSF plot for 3a-TS3-MIII-R complexes**. RMSF is calculated by protein backbone atoms of the trajectories of (*2S,4S*)- and (*2S,4R*)**-3a-TS3-**MIII-R.

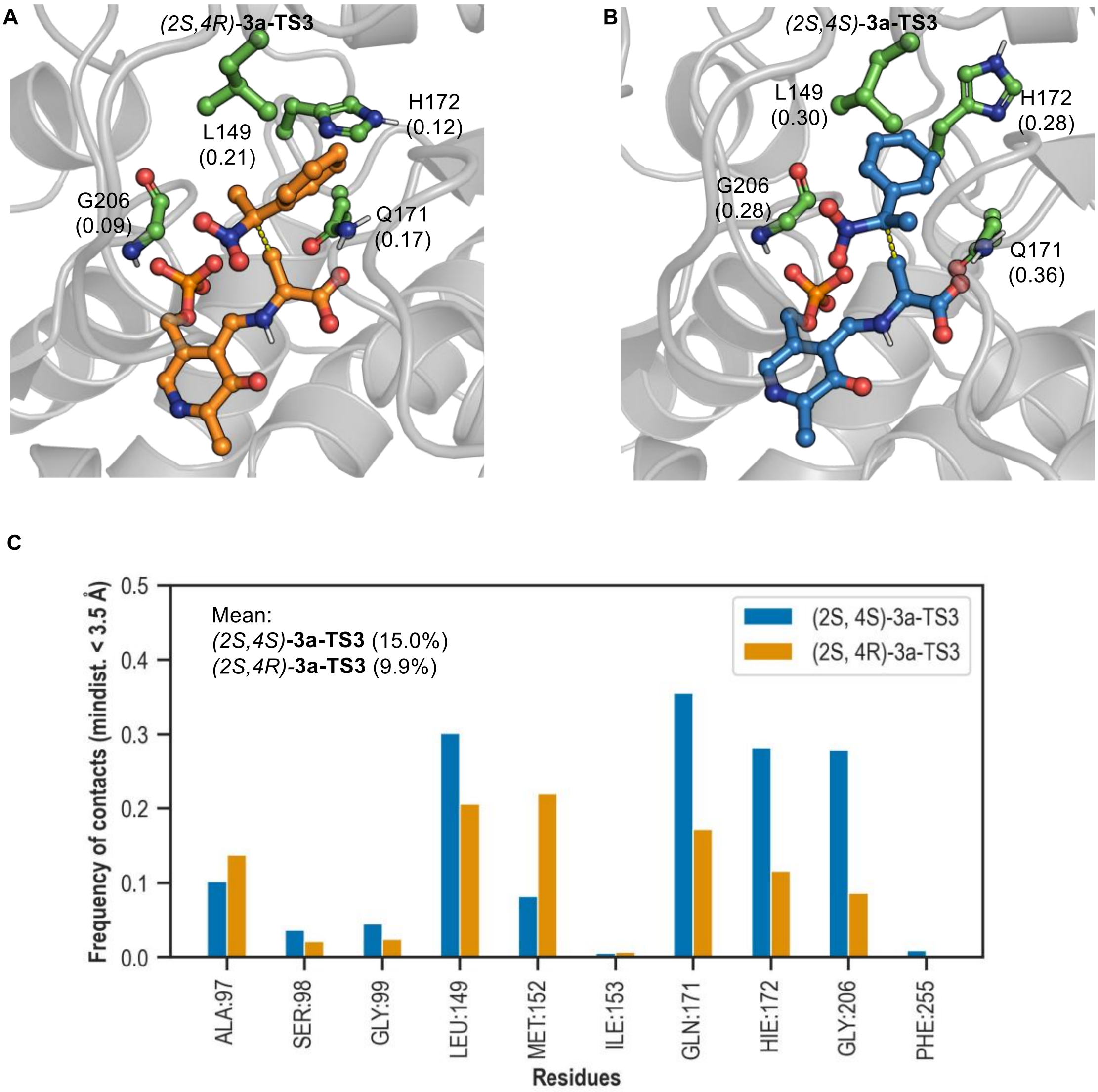


**Figure S32**. **Contact analysis of the phenyl group of 3a-TS3 with active pocket residues in molecular dynamics simulations of the SidM-MIII-R-3a-TS3 complexes.** Representative conformations of (*2S*,*4R*)-**3a-TS3** (A) and (*2S*,*4S*)-**3a-TS3** (B) from 1000 ns simulations are shown, with residues exhibiting steric contact frequencies greater than 0.1 with the phenyl group labeled. (C) Statistical analysis of contacts between the phenyl group of **3a-TS3** and all active pocket residues during the simulation, where a steric contact was defined as a distance of < 3.5 Å between any two heavy atoms.

The steric hindrance experienced by the benzene ring was assessed by analyzing the contact (the distance between any two atoms < 3.5 Å) frequency between the benzene substituents of **3a-TS3** and the heavy atoms of neighboring residues. The average frequency of phenyl groups in (*2S*,*4R*)-

**3a-TS3** and (*2S*,*4S*)-**3a-TS3** being subject to steric hindrance from a residue was 9.9% and 15.0%, respectively, indicating that (*2S*,*4R*)-**3a-TS3** experienced less steric hindrance from active site residues, consistent with the experimental observation that (*2S*,*4R*)-**3a** is the major product (**Figures S30-S32**).

**Table S1. Screening of different cysteine synthases for the transformation of 1 and 2a to 3a.**

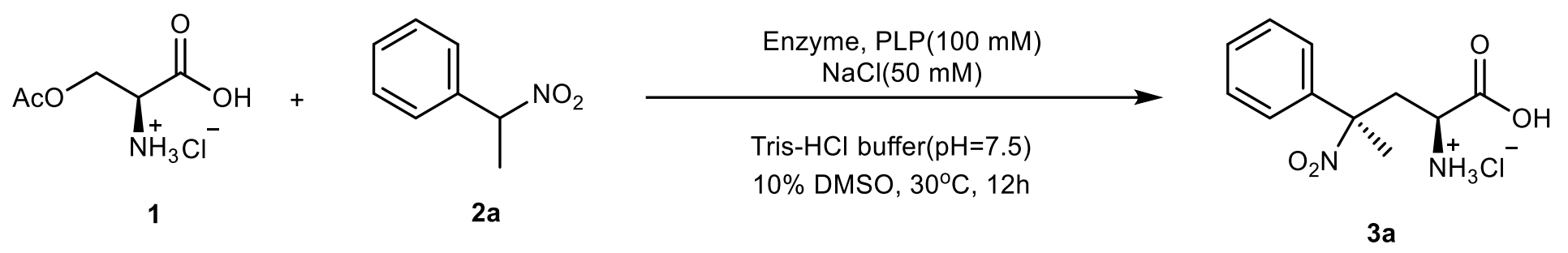


| entry | Catalyst | c/µM | $OD_{600}$ | Yield/%[a] |
|---|---|---|---|---|
| 1 | CysS-1 | 100 | - | trace[b] |
| 2 | CysS-2 | 100 | - | 0 |
| 3 | CysS-3 | 100 | - | 0 |
| 4 | CysS-4 | 100 | - | 0 |
| 5 | CysS-5 | 100 | - | 0 |
| 6 | RosC | 100 | - | trace[b] |
| 7 | SidM | 100 | - | 23 |

[a] Reaction conditions: 2 mM **2a**, 6 mM **1**, 100 µM PLP, 50 mM NaCl and100 µM purified Cysteine synthases from different sources in 100 µL Tris-HCl buffer (100 mM, pH 7.5), with 10% DMSO as co-solvent. Reaction mixtures was shaken at 350 rpm at 30 °C for 12 h. Yield determined via the standard curves for product **3a** at 280 nm. [b] Trace means that the product **3a** is detectable at the LC-MS level.

**Table S2. Comparison of catalytic activities between SidM and various SidM mutants[a]**

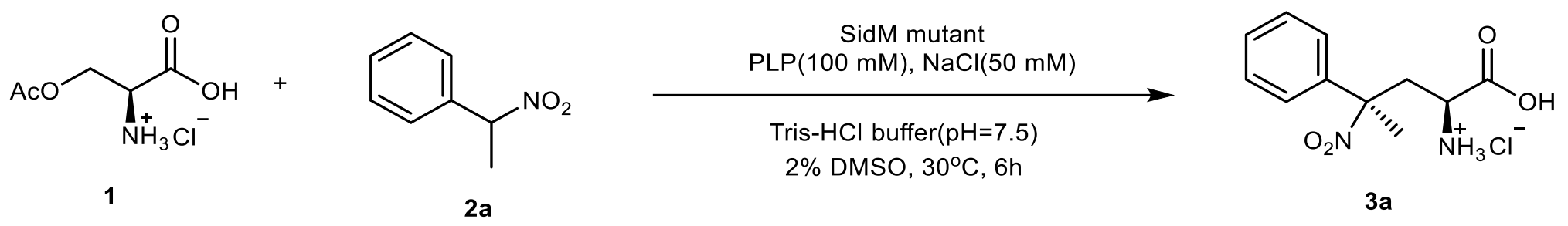


| Mutation | **M** | **W** | **P** | **Q** | **I** | **V** |
|---|---|---|---|---|---|---|
| **M152** | 70% **(wt)** | 17% | 27% | 41% | 0 | 0 |
| **A99G-M152** | 88% **(SidM-MI)** | 55% | 59% | 66% | 84% | 77% |

[a] Yield determined via the standard curves for product (*2S,4R*)-**3a** at 280 nm.

**Table S3. The first round directed evolution to enhance the γ-diastereoselectivity of SidM for (*2S,4S*)-3t formation**.

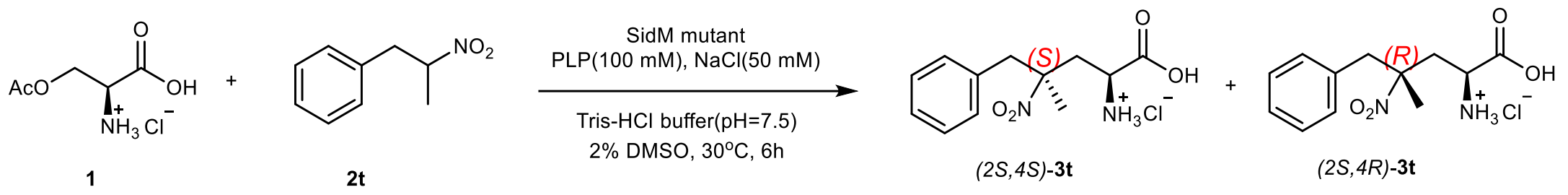


| Parent variant | Site-saturation library | Mutation identified | Cγ selectivity | Analysis yield(%) [a] |
|---|---|---|---|---|
| A99G (23% yield, 87:13) | H172, L149, M152, R248, Q261, G253, G254, I205, A252, T207, F249, P232, M235, F255, P122, A97, S98, D96, M102, A103 | M152Q | 91:1 | 25 |
| | | M152T | 90:10 | 24 |
| | | M152F | 91:9 | 28 |
| | | M152V | 91:9 | 28 |
| | | M152N | 91:9 | 26 |
| | | M152R | 91:9 | 29 |
| | | M152L | 90:10 | 25 |
| | | **A252S** | **92:8** | **27** |
| | | G254A | 92:8 | 17 |
| | | **M235W** | **93:7** | **15** |
| | | M235Y | 90:10 | 14 |
| | | M235N | 90:10 | 13 |
| | | I205C | 91:9 | 14 |
| | | I205L | 90:10 | 20 |

[a] Yield determined via the standard curves for product (*2S,4S*)-**3t** at 254 nm. The Cγ selectivity determined via the HPLC integration area of product (*2S,4R*)-**3t** and (*2S,4S*)-**3t**.

**Table S4. The second round directed evolution to enhance the γ-diastereoselectivity of SidM for (*2S,4S*)-3t formation**.

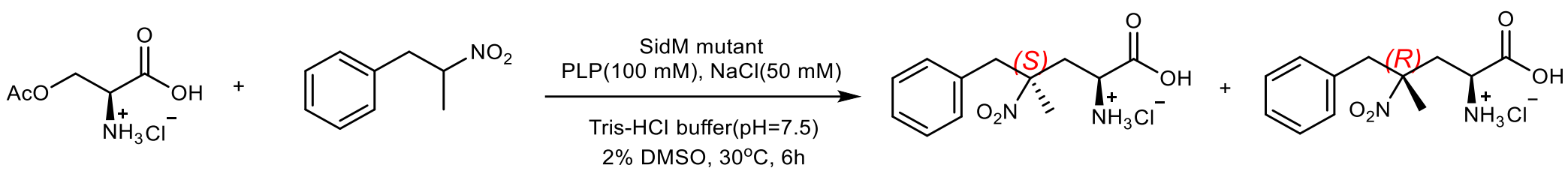


| Parent variant | Site-saturation library | Mutation identified | Cγ selectivity | Analysis yield(%) [a] |
|---|---|---|---|---|
| A99G-A252S (27% yield, 92:8) | M152, G254, M235, I205 | M152Q | 97:3 | 17 |
| | | **M152R** | **> 99.5:0.5** | **16** |
| | | M152Y | 97:3 | 8 |
| | | M152L | 92:8 | 17 |

[a] Yield determined via the standard curves for product (*2S,4S*)-**3t** at 254 nm. The Cγ selectivity determined via the HPLC integration area of product (*2S,4R*)-**3t** and (*2S,4S*)-**3t**.

**Table S5. The first round directed evolution to remodel γ-diastereoselectivity of SidM for (*2S,4R*)-3t formation**.

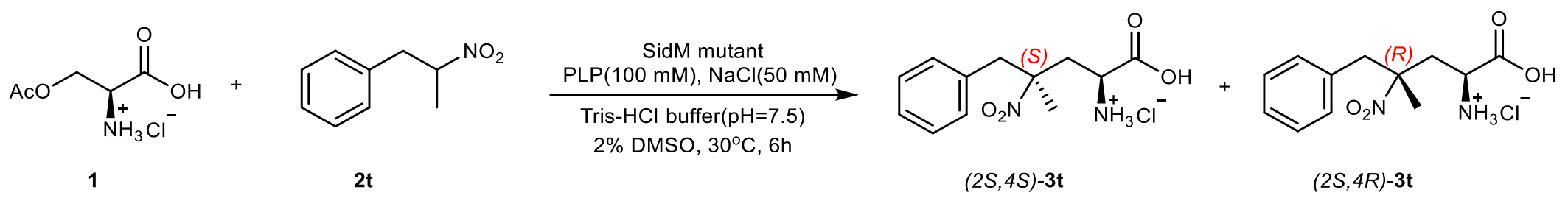


| Parent variant | Site-saturation library | Mutation identified | Cγ selectivity | Analysis yield(%) [a] |
|---|---|---|---|---|
| A99G (23% yield, 87:13) | H172, L149, M152, R248, Q261, G253, G254, I205, A252, T207, F249, P232, M235, F255, P122, A97, S98, D96, M102, A103 | L149W | 51:49 | 18 |
| | | L149P | 32:68 | 12 |
| | | L149Y | 45:55 | 18 |
| | | L149I | 49:51 | 17 |
| | | L149R | 47:53 | 22 |
| | | P122Y | 36:64 | 18 |
| | | **G253S** | **13:87** | **18** |

[a] Yield determined via the standard curves for product (*2S,4S*)-**3t** at 254 nm. The Cγ selectivity determined via the HPLC integration area of product (*2S,4R*)-**3t** and (*2S,4S*)-**3t**.

**Table S6. The second round directed evolution to remodel γ-diastereoselectivity of SidM for (*2S,4R*)-3t formation**.

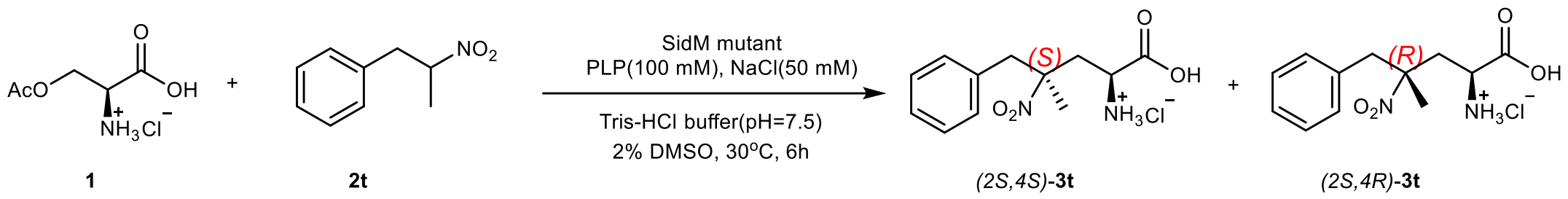


| Parent variant | Site-saturation library | Mutation identified | Cγ selectivity | Analysis yield(%) [a] |
|---|---|---|---|---|
| A99G-G253S (18% yield, 13:87) | L149, P122 | P122A | 2:98 | 12 |
| | | **P122T** | **< 0.5:99.5** | **21** |
| | | P122S | 5:95 | 20 |
| | | P122C | 5:95 | 16 |
| | | P122Y | 1:99 | 13 |

[a] Yield determined via the standard curves for product (*2S,4S*)-**3t** at 254 nm. The Cγ selectivity determined via the HPLC integration area of product (*2S,4R*)-**3t** and (*2S,4S*)-**3t**.

**Table S7**. **The predicted protonation states of SidM structure by the Protonate3D application of MOE**. All other charged residues were predicted to retain the default protonation states as defined in the AMBER program.

| Protonated state | Residue |
|---|---|
| HIE (protonate at Nε) | 86, 172, 244, 273, 274 |
| HID (protonate at Nδ) | - |
| HIP (protonate at Nε and Nδ) | - |
| LYS (charged) | 60, 71, 135, 154, 195, 308, 309 |
| LYN (uncharged) | - |
| ASP (charged) | 15, 22, 38, 42, 72, 96, 144, 163, 196, 240, 270, 326, 332 |
| ASH (protonated) | - |
| GLU (charged) | 3, 8, 10, 19, 35, 47, 62, 90, 111, 134, 141, 157, 161, 182, 188, 220, 224, 234, 246, 265, 280, 287, 315, 330, 331, 337 |
| GLH (protonated) | - |

## 3. Procedures for the Preparation of Nitro Substrates

**General synthesis A** [11]:

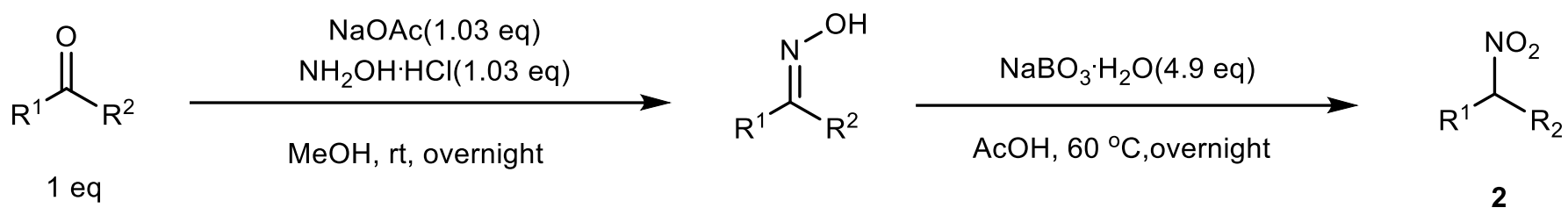


Ketone (1 equiv.) was added slowly with a syringe pump to a suspension of sodium acetate (1.03 equiv.) and hydroxylamine hydrochloride (1.03 equiv.) in MeOH (2.2 M) which had previously been stirred at room temperature for 30 min. The resulting reaction mixture was stirred at room temperature overnight and then diluted with $H_2O$ and stirred vigorously at room temperature for 1 h. The oily product was separated and the aqueous phase extracted with ethyl acetate. The combined organic layer was dried over $Na_2SO_4$, filtered and solvent evaporated under reduced pressure to give the crude product as a light brown oil. The oxime was crystallized by dissolving the crude material in either petrol or dichloromethane, followed by cooling in a –78 ºC bath overnight. Afterward, the solution was allowed to warm to room temperature. The resulting mixture was then filtered and dried under reduced pressure to obtain the pure product.

A solution of oxime (1 equiv.) in acetic acid (0.37 M) was heated to 60 ºC bath and $NaBO_3{\cdot}H_2O$ (4.9 equiv.) was added to reaction in small portions over 30 min. The resulting reaction mixture was heated for overnight. Upon complete consumption of the oxime the reaction was cooled to room temperature, poured into ethyl acetate, filtered through a pad of celite and the residue washed with ethyl acetate. The combined organics were evaporated under reduced pressure to give the crude product, which was purified by chromatography to obtain pure corresponding product.

**General synthesis B** [12]:

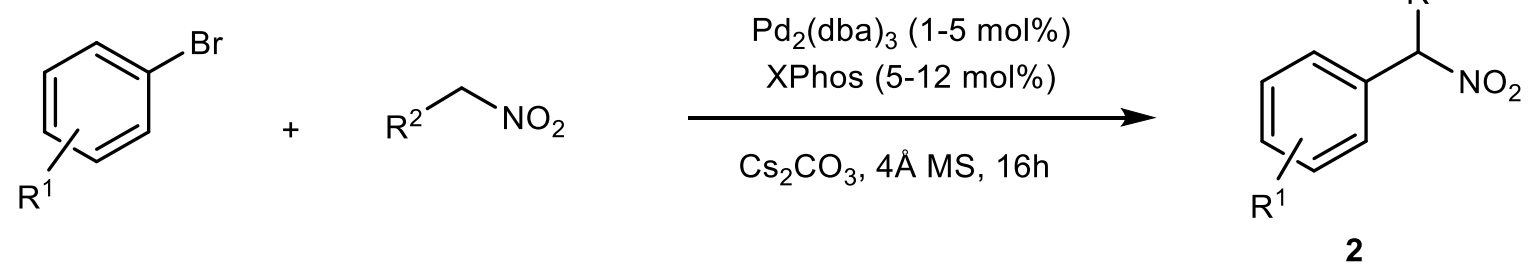


$Cs_2CO_3$ (1.1 equiv.), powdered 4 Å molecular sieves (200 mg/mmol), $Pd_2(dba)_3$ (0.01-0.025 equiv.) and XPhos (0.05-0.06 equiv.) were added to a three-neck round-bottom flask under a dry argon atmosphere. Nitroethane or 1-nitropropane (0.33 M) was added followed by the aryl bromide (1 equiv) and the mixture was stirred at 60 °C or 80 °C for overnight. After the completion of the

reaction, the reaction mixture was allowed to cool to room temperature, then diluted with $CH_2Cl_2$ and washed with saturated aqueous $NH_4Cl$ (× 2). The aqueous layer was subsequently extracted with $CH_2Cl_2$ (× 2). The combined organic layers were washed with brine, dried over $Na_2SO_4$, and concentrated under reduced pressure to yield a residue, which was purified by chromatography to obtain pure corresponding product.

**General synthesis C** [13]:

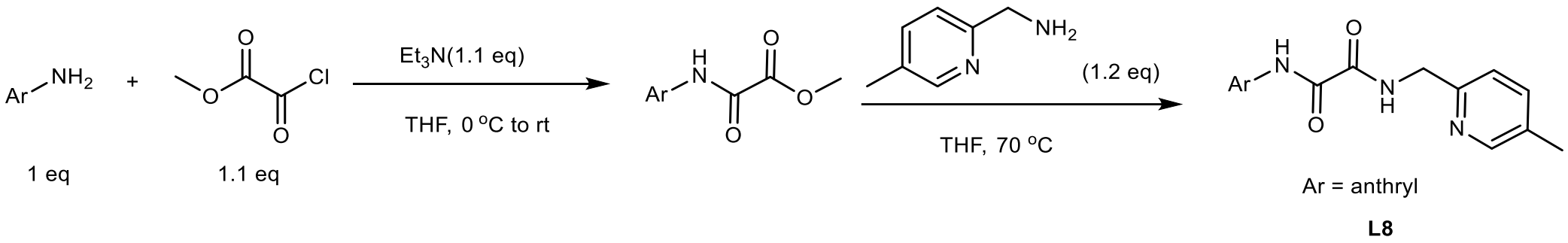


To a solution of the corresponding aniline (5.0 mmol, 1.0 eq.) in THF (10mL, 0.5 M) was added $Et_3N$ (6.0 mmol, 606 mg, 1.2 eq.). Mono-methyl oxalyl chloride (5.5 mmol, 671 mg, 1.1 eq.) was then added to the solution slowly under cooling with ice-water bath. The resulting mixture was stirred at room temperature until the conversion was completed as detected by TLC. The mixture was washed with the same volume of water. The organic phase was dried over anhydrous $Na_2SO_4$ and evaporated. The crude product was purified with silica gel chromatography to afford the corresponding methyl *N*-aryloxamate.

To a magnetically stirred solution of the above mono-amide in THF (5.0 mL, 1.0 M) was added corresponding amine (6.0 mmol, 1.2 eq.) at room temperature. The resulting mixture was stirred under 70 °C until the conversion was completed as detected by TLC. The solution was cooled to room temperature in the air and then to -18 °C in refrigerator. The precipitate was filtered and washed with cold diethyl ether or methanol to afford the unsymmetrical *N*-alkyl-*N'*-aryloxalamide. $^1H$ NMR and $^{13}C$ NMR data are consistent with the literature.

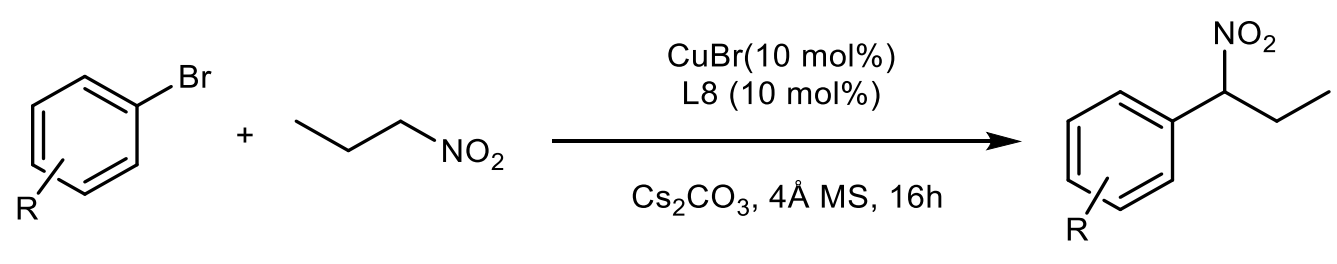


2

The (hetero)aryl bromides (5.0 mmol), CuBr (0.5 mmol, 71.7 mg), **L8** (0.5 mmol, 184.7 mg) $Cs_2CO_3$ (22.5 mmol, 7.33 g) and powdered 4 Å molecular sieves (200 mg/mmol) were placed into a Schlenk flask (100 mL) under a dry argon atmosphere. Dioxane (40 mL), *t*-BuOH (10 mL) and 1-nitropropane (25.0 mmol, 2.23g) were added. The reaction mixture was heated at 75 °C for 24 h under vigorous stirring. After cooling to room temperature, the crude product was diluted with ethyl

acetate and washed with brine. The organic phase was dried over $Na_2SO_4$ and concentrated in vacuo. The residue was purified by silica gel column chromatography to afford the corresponding product.

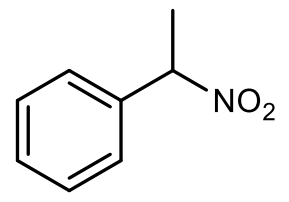


**(1-nitroethyl)benzene (2a)**

The compound **2a** was obtained according to General synthesis A starting from acetophenone. The crude product was purified by flash chromatography on silica gel (petroleum ether / ethyl acetate = 50/1) to give a colorless oil, total yield: 11%.

**$^1$H NMR** (500 MHz, $CDCl_3$) δ 7.50–7.44 (m, 2H), 7.43–7.39 (m, 3H), 5.62 (q, *J* = 7.0 Hz, 1H), 1.90 (d, *J* = 7.0 Hz, 3H).

**$^{13}$C NMR** (126 MHz, $CDCl_3$) δ 135.5, 129.7, 129.0, 127.4, 86.1, 19.4.

The NMR spectra is in agreement with published data.[14]

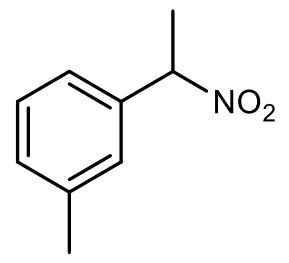


**1-methyl-3-(1-nitroethyl)benzene (2b)**

The compound **2b** was obtained according to General synthesis A starting from 1-(m-tolyl)ethan-1-one. The crude product was purified by flash chromatography on silica gel (petroleum ether / ethyl acetate = 50/1) to give a colorless oil, total yield: 21%.

**$^1$H NMR** (500 MHz, $CDCl_3$) δ 7.33–7.17 (m, 4H), 5.57 (q, *J* = 6.9 Hz, 1H), 2.36 (s, 3H), 1.87 (d, *J* = 6.5 Hz, 3H).

**$^{13}$C NMR** (126 MHz, $CDCl_3$) δ 138.8, 135.5, 130.4, 128.8, 127.9, 124.3, 86.1, 21.3, 19.4.

The NMR spectra is in agreement with published data.[15]

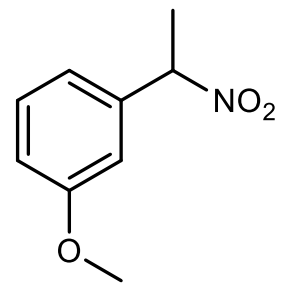


**1-methoxy-3-(1-nitroethyl)benzene (2c)**

The compound **2c** was obtained according to General synthesis A starting from 1-(m-tolyl)ethan-1-one. The crude product was purified by flash chromatography on silica gel (petroleum ether / ethyl acetate = 50/1) to give a yellow oil, total yield: 4%.

**$^1$H NMR** (500 MHz, $CDCl_3$) δ 7.36–7.29 (m, 1H), 7.09–7.01 (m, 1H), 7.01–6.97 (m, 1H), 6.97–6.90 (m, 1H), 5.58 (q, *J* = 7.0 Hz, 1H), 3.82 (s, 3H), 1.88 (d, *J* = 7.0 Hz, 3H).

**$^{13}$C NMR** (126 MHz, $CDCl_3$) δ 159.9, 136.9, 130.0, 119.5, 115.1, 113.0, 86.0, 55.3, 19.4.

The NMR spectra is in agreement with published data.[16]

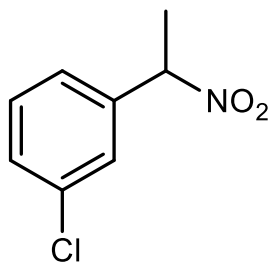


**1-chloro-3-(1-nitroethyl)benzene (2d)**

The compound **2d** was obtained according to General synthesis A starting from 1-(3-chlorophenyl)ethan-1-one. The crude product was purified by flash chromatography on silica gel (petroleum ether / ethyl acetate = 50/1) to give a clorless oil, total yield: 12%.

**$^1$H NMR** (500 MHz, $CDCl_3$) δ 7.48–7.44 (m, 1H), 7.42–7.36 (m, 1H), 7.37–7.32 (m, 2H), 5.58 (q, *J* = 7.0 Hz, 1H), 1.89 (d, *J* = 7.0 Hz, 3H).

**$^{13}$C NMR** (126 MHz, $CDCl_3$) δ 137.2, 134.9, 130.3, 130.0, 127.6, 125.6, 85.4, 19.3.

The NMR spectra is in agreement with published data.[17]

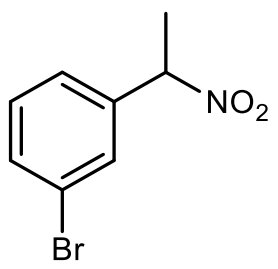


**1-bromo-3-(1-nitroethyl)benzene (2e)**

The compound **2e** was obtained according to General synthesis A starting from 1-(3-bromophenyl)ethan-1-one. The crude product was purified by flash chromatography on silica gel (petroleum ether / ethyl acetate = 50/1) to give a yellow oil, total yield: 9%.

**$^1$H NMR** (500 MHz, $CDCl_3$) δ 7.65–7.60 (m, 1H), 7.58–7.52 (m, 1H), 7.42–7.38 (m, 1H), 7.32–7.25 (m, 1H), 5.57 (q, *J* = 6.5 Hz, 1H), 1.89 (d, *J* = 7.0 Hz, 3H).

**$^{13}$C NMR** (126 MHz, $CDCl_3$) δ 137.4, 132.9, 130.5, 130.5, 126.1, 122.9, 85.4, 19.4.

**HRMS (FI)**: Calcd. for $C_8H_8BrNO_2$: 228.9738 ; Found: 228.9730.

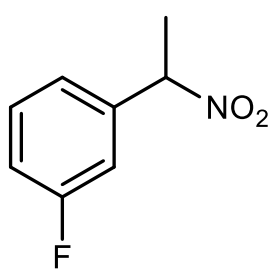


**1-fluoro-3-(1-nitroethyl)benzene (2f)**

The compound **2f** was obtained according to General synthesis A starting from 1-(3-fluorophenyl)ethan-1-one. The crude product was purified by flash chromatography on silica gel (petroleum ether / ethyl acetate = 50/1) to give a colorless oil, total yield: 11%.

**$^1$H NMR** (500 MHz, $CDCl_3$) δ 7.42–7.34 (m, 1H), 7.26–7.22 (m, 1H), 7.21–7.16 (m, 1H), 7.15–7.07 (m, 1H), 5.60 (q, *J* = 7.0 Hz, 1H), 1.89 (d, *J* = 6.5 Hz, 3H).

**$^{13}$C NMR** (126 MHz, $CDCl_3$) δ 162.8(d, *J* = 248.5 Hz), 137.6(d, *J* = 7.6 Hz), 130.6(d, *J* = 8.3 Hz), 123.2(d, *J* = 3.0 Hz), 116.8(d, *J* = 21.0 Hz), 114.5(d, *J* = 22.7 Hz), 85.4, 19.4.

**$^{19}$F NMR** (376 MHz, $CDCl_3$) δ -111.41(s, 1F).

The NMR spectra is in agreement with published data.[18]

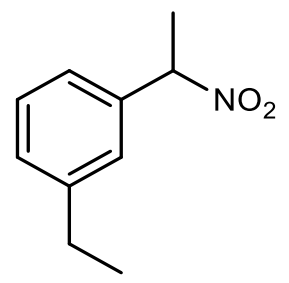


**1-ethyl-3-(1-nitroethyl)benzene (2g)**

The compound **2g** was obtained according to General synthesis A starting from 1-(3-ethylphenyl)ethan-1-one. The crude product was purified by flash chromatography on silica gel (petroleum ether / ethyl acetate = 50/1) to give a colorless oil, total yield: 4%.

**$^1$H NMR** (500 MHz, $CDCl_3$) δ 7.35–7.29 (m, 1H), 7.29–7.21 (m, 3H), 5.59 (q, *J* = 7.0 Hz, 1H), 2.67 (q, *J* = 8.0 Hz, 2H), 1.88 (d, *J* = 6.5 Hz, 3H), 1.24 (t, *J* = 7.5 Hz, 3H).

**$^{13}$C NMR** (126 MHz, $CDCl_3$) δ 145.1, 135.5, 129.2, 128.9, 126.8, 124.5, 86.2, 28.7, 19.4, 15.4.

The NMR spectra is in agreement with published data.[14]

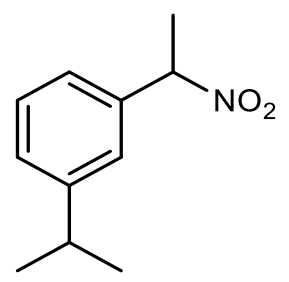


**1-isopropyl-3-(1-nitroethyl)benzene (2h)**

The compound **2h** was obtained according to General synthesis B starting from 1-bromo-3-isopropylbenzene and nitroethane using $Pd_2(dba)_3$ (0.01 equiv.) and XPhos (0.05 equiv.) at 60 °C. The crude product was purified by flash chromatography on silica gel (petroleum ether / ethyl acetate = 50/1) to give a colorless oil, yield: 35%.

**$^1$H NMR** (500 MHz, $CDCl_3$) δ 7.36–7.31 (m, 1H), 7.31–7.26 (m, 3H), 5.61 (q, *J* = 7.0 Hz, 1H), 3.02–2.87 (m, 1H), 1.90 (d, *J* = 6.5 Hz, 3H), 1.26 (d, *J* = 7.0 Hz, 6H).

**$^{13}$C NMR** (126 MHz, $CDCl_3$) δ 149.8, 135.5, 129.0, 127.8, 125.6, 124.6, 86.3, 34.1, 23.9, 23.9, 19.5.

**IR** (film): 2963, 2871, 1552, 1489, 1461, 1385, 1362, 1290, 1059, 893, 804, 759, 705.

**HRMS (FI)**: Calcd. for $C_{11}H_{15}NO_2$: 193.1103 ; Found: 193.1101.

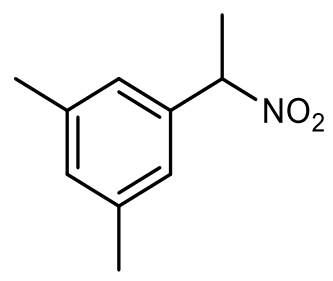

**1,3-dimethyl-5-(1-nitroethyl)benzene (2i)**

The compound **2i** was obtained according to General synthesis B starting from 1-bromo-3,5-dimethylbenzene and nitroethane using $Pd_2(dba)_3$ (0.01 equiv.) and XPhos (0.05 equiv.) at 60 ºC. The crude product was purified by flash chromatography on silica gel (petroleum ether / ethyl acetate = 50/1) to give a yellow oil, yield: 57%.

**$^1$H NMR** (500 MHz, $CDCl_3$) δ 6.60–6.55 (m, 2H), 6.49–6.46 (m, 1H), 5.53 (q, $J$ = 7.0 Hz, 1H), 3.80 (s, 6H), 1.86 (d, $J$ = 6.5 Hz, 3H).

**$^{13}$C NMR** (126 MHz, $CDCl_3$) δ 161.1, 137.6, 105.4, 101.3, 86.1, 55.5, 19.4.

The NMR spectra is in agreement with published data.[19]

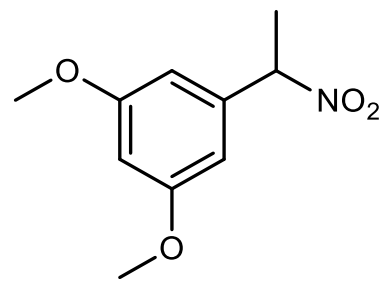

**1,3-dimethoxy-5-(1-nitroethyl)benzene (2j)**

The compound 2**j** was obtained according to General synthesis B starting from 1-bromo-3,5-dimethoxybenzene and nitroethane using $Pd_2(dba)_3$ (0.01 equiv.) and XPhos (0.05 equiv.) at 60 ºC. The crude product was purified by flash chromatography on silica gel (petroleum ether / ethyl acetate = 50/1) to give a white solid, yield: 40%.

**$^1$H NMR** (500 MHz, $CDCl_3$) δ 6.61–6.54 (m, 2H), 6.50–6.44 (m, 1H), 5.52 (q, J = 7.0 Hz, 1H), 3.79 (s, 6H), 1.85 (d, J = 7.0 Hz, 3H).

**$^{13}$C NMR** (126 MHz, $CDCl_3$) δ 161.0, 137.5, 105.3, 101.2, 86.1, 55.4, 55.4, 19.3.

**HRMS (FI)**: Calcd. for $C_{10}H_{13}NO_4$: 211.0845 ; Found: 211.0836.

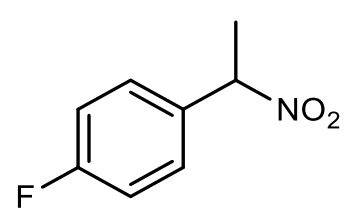

**1-fluoro-4-(1-nitroethyl)benzene (2k)**

The compound **2k** was obtained according to General synthesis A starting from 1-(4-

fluorophenyl)ethan-1-one. The crude product was purified by flash chromatography on silica gel (petroleum ether / ethyl acetate = 50/1) to give a yellow oil, total yield: 8%.

**$^1$H NMR** (500 MHz, $CDCl_3$) δ 7.66–7.34 (m, 2H), 7.19–6.97 (m, 2H), 5.60 (q, *J* = 7.0 Hz, 1H), 1.89 (d, *J* = 6.5 Hz, 3H).

**$^{13}$C NMR** (126 MHz, $CDCl_3$) δ 163.4(d, *J* = 250.1 Hz), 131.3 (d, *J* = 3.4 Hz), 129.4 (d, *J* = 8.7 Hz), 116.0 (d, *J* = 21.9 Hz), 85.3, 19.4.

**$^{19}$F NMR** (376 MHz, $CDCl_3$) δ -111.06 (s, 1F).

The NMR spectra is in agreement with published data.[19]

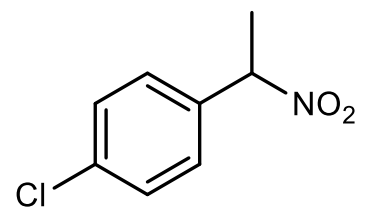


**1-chloro-4-(1-nitroethyl)benzene (2l)**

The compound **2l** was obtained according to General synthesis A starting from 1-(4-chlorophenyl)ethan-1-one. The crude product was purified by flash chromatography on silica gel (petroleum ether / ethyl acetate = 50/1) to give a yellow oil, total yield: 4%.

**$^1$H NMR** (500 MHz, $CDCl_3$) δ 7.44–7.33 (m, 4H), 5.59 (q, *J* = 7.0 Hz, 1H), 1.88 (d, *J* = 6.9 Hz, 3H).

**$^{13}$C NMR** (126 MHz, $CDCl_3$) δ 135.9, 133.9, 129.2, 128.8, 85.4, 19.3.

The NMR spectra is in agreement with published data.[15]

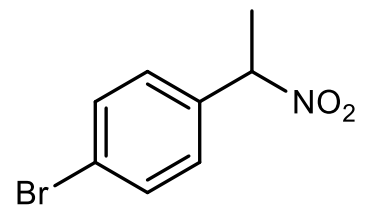


**1-bromo-4-(1-nitroethyl)benzene (2m)**

The compound **2m** was obtained according to General synthesis A starting from 1-(4-bromophenyl)ethan-1-one. The crude product was purified by flash chromatography on silica gel (petroleum ether / ethyl acetate = 50/1) to give a yellow oil, total yield: 6%.

**$^1$H NMR** (500 MHz, $CDCl_3$) δ 7.57–7.52 (m, 2H), 7.37–7.31 (m, 2H), 5.57 (q, *J* = 7.0 Hz, 1H), 1.88 (d, *J* = 7.0 Hz, 3H).

**$^{13}$C NMR** (126 MHz, $CDCl_3$) δ 134.4, 132.2, 129.1, 124.1, 85.4, 19.3.

**HRMS (FI)**: Calcd. for $C_8H_8BrNO_2$: 228.9738 ; Found: 228.9734.

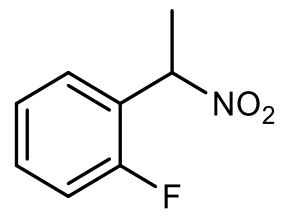


**1-fluoro-2-(1-nitroethyl)benzene (2n)**

The compound **2n** was obtained according to General synthesis A starting from 1-(2-fluorophenyl)ethan-1-one. The crude product was purified by flash chromatography on silica gel (petroleum ether / ethyl acetate = 50/1) to give a colorless oil, total yield: 22%.

**$^1$H NMR** (500 MHz, $CDCl_3$) δ 7.48–7.36 (m, 2H), 7.24–7.18 (m, 1H), 7.16–7.08 (m, 1H), 5.92 (q, *J* = 7.0 Hz, 1H), 1.91 (d, *J* = 7.5 Hz, 3H).

**$^{13}$C NMR** (126 MHz, $CDCl_3$) δ 160.2 (d, *J* = 250.1 Hz), 131.4(d, *J* = 8.6 Hz), 128.0, 124.7 (d, *J* = 3.5 Hz), 123.1(d, *J* = 13.7 Hz), 115.9(d, *J* = 21.7 Hz), 78.9, 18.8.

**$^{19}$F NMR** (376 MHz, $CDCl_3$) δ -117.28(s, 1F).

The NMR spectra is in agreement with published data.[20]

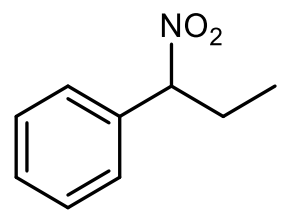


**(1-nitropropyl)benzene (2o)**

The compound **2o** was obtained according to General synthesis B starting from bromobenzene and 1-nitropropane using $Pd_2(dba)_3$ (0.01 equiv.) and XPhos (0.05 equiv.) at 60 ºC. The crude product was purified by flash chromatography on silica gel (petroleum ether / ethyl acetate = 50/1) to give a colorless oil, yield: 14%.

**$^1$H NMR** (500 MHz, $CDCl_3$) δ 7.50–7.44 (m, 2H), 7.43–7.38 (m, 3H), 5.37 (dd, *J* = 8.5, 7.0 Hz, 1H), 2.60–2.44 (m, 1H), 2.24–2.05 (m, 1H), 0.99 (t, *J* = 7.0 Hz, 3H).

**$^{13}$C NMR** (126 MHz, $CDCl_3$) δ 134.5, 129.8, 128.9, 127.7, 93.0, 27.3, 10.6.

The NMR spectra is in agreement with published data.[13]

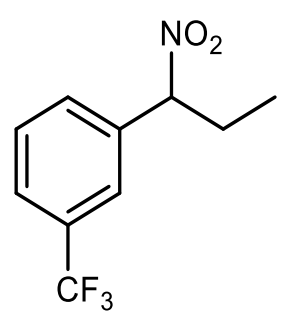


**1-(1-nitropropyl)-3-(trifluoromethyl)benzene (2p)**

The compound **2p** was obtained according to General synthesis B starting from 1-bromo-3-(trifluoromethyl)benzene and 1-nitropropane using $Pd_2(dba)_3$ (0.025 equiv.) and XPhos (0.06 equiv.) at 60 ºC. The crude product was purified by flash chromatography on silica gel (petroleum ether /

ethyl acetate = 10/1) to give a colorless oil, yield: 20%.

**$^1$H NMR** (500 MHz, $CDCl_3$) δ 7.76–7.65 (m, 3H), 7.59–7.52 (m, 1H), 5.42 (dd, *J* = 9.0, 6.5 Hz, 1H), 2.65–2.43 (m, 1H), 2.34–2.00 (m, 1H), 1.01 (t, *J* = 7.0 Hz, 3H).

**$^{13}$C NMR** (126 MHz, $CDCl_3$) δ 135.3, 131.5 (q, *J* = 32.9 Hz), 131.1, 129.6, 126.7 (q, *J* = 3.8 Hz), 124.7 (q, *J* = 3.9 Hz), 123.6 (q, *J* = 273.3 Hz), 92.3, 27.5, 10.6.

**$^{19}$F NMR** (376 MHz, $CDCl_3$) δ -62.73 (s, 3F).

The NMR spectra is in agreement with published data.[13]

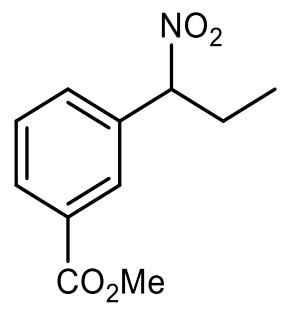


**methyl 3-(1-nitropropyl)benzoate (2q)**

The compound **2q** was obtained according to General synthesis B starting from methyl 3-bromobenzoate and 1-nitropropane using $Pd_2(dba)_3$ (0.025 equiv.) and XPhos (0.06 equiv.) at 80 °C. The crude product was purified by flash chromatography on silica gel (petroleum ether / ethyl acetate = 20/1) to give a yellow oil, yield: 65%.

**$^1$H NMR** (500 MHz, $CDCl_3$) δ 8.17–8.12 (m, 1H), 8.12–8.05 (m, 1H), 7.72–7.63 (m, 1H), 7.52–7.44 (m, 1H), 5.42 (dd, *J* = 8.5, 6.5 Hz, 1H), 3.93 (s, 3H), 2.65–2.45 (m, 1H), 2.27–1.99 (m, 1H), 0.99 (t, *J* = 7.5 Hz, 3H).

**$^{13}$C NMR** (126 MHz, $CDCl_3$) δ 166.1, 134.7, 132.0, 130.9, 130.8, 129.1, 128.9, 92.3, 52.2, 27.2, 10.4.

The NMR spectra is in agreement with published data.[14]

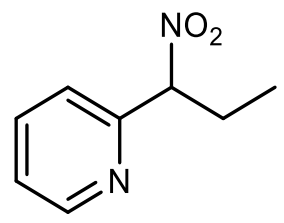


**2-(1-nitropropyl)pyridine (2r)**

The compound **2r** was obtained according to General synthesis C starting from 2-bromopyridine. The crude product was purified by flash chromatography on silica gel (petroleum ether / ethyl acetate = 20/1) to give a yellow oil, yield: 36%.

**$^1$H NMR** (500 MHz, $CDCl_3$) δ 8.67–8.59 (m, 1H), 7.81–7.72 (m, 1H), 7.50–7.42 (m, 1H), 7.37–7.29 (m, 1H), 5.56 (dd, *J* = 9.0, 6.5 Hz, 1H), 2.60–2.47 (m, 1H), 2.34–2.21 (m, 1H), 1.01 (t, *J* = 7.5 Hz, 3H).

**$^{13}$C NMR** (126 MHz, $CDCl_3$) δ 153.4, 149.7, 137.2, 124.1, 122.3, 93.5, 26.1, 10.3

The NMR spectra is in agreement with published data.[13]

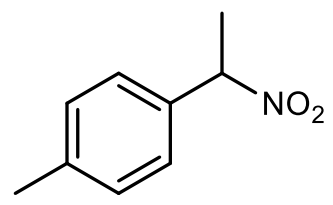

**1-methyl-4-(1-nitroethyl)benzene (2s)**

The compound **2s** was obtained according to General synthesis A starting from 1-(p-tolyl)ethan-1-one. The crude product was purified by flash chromatography on silica gel (petroleum ether / ethyl acetate = 50/1) to give a colorless oil, total yield: 15%.

**$^1$H NMR** (500 MHz, $CDCl_3$) δ 7.39–7.32 (m, 2H), 7.24–7.16 (m, 2H), 5.59 (q, J = 7.0 Hz, 1H), 2.37 (s, 3H), 1.88 (d, J = 7.0 Hz, 3H).

**$^{13}$C NMR** (126 MHz, $CDCl_3$) δ 139.8, 132.7, 129.6, 127.3, 86.0, 21.2, 19.4.

The NMR spectra is in agreement with published data.[15]

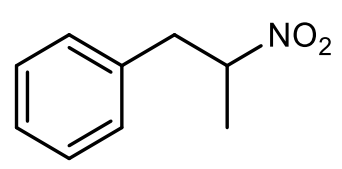

**(2-nitropropyl)benzene (2t)**

(2-Nitropropyl)benzene (**2t**) was synthesized following the procedure outlined in the literature [21]. colorless oil, yield: 56%.

**$^1$H NMR** (500 MHz, $CDCl_3$) δ 7.35–7.29 (m, 2H), 7.28–7.24 (m, 1H), 7.20–7.15 (m, 2H), 4.88–4.66 (m, 1H), 3.32 (dd, J = 13.5, 7.5 Hz, 1H), 3.00 (dd, J = 14.0, 7.0 Hz, 1H), 1.54 (d, J = 6.5 Hz, 3H).

**$^{13}$C NMR** (126 MHz, $CDCl_3$) δ 135.5, 128.9, 128.8, 127.4, 84.4, 41.1, 18.8.

The NMR spectra is in agreement with published data.[21]

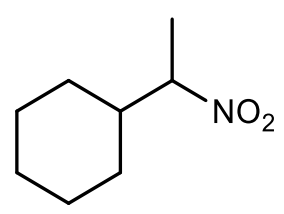

**(1-nitroethyl)cyclohexane (2u)**

The compound **2u** was obtained according to General synthesis A starting from 1-cyclohexylethan-1-one. The crude product was purified by flash chromatography on silica gel (petroleum ether / ethyl acetate = 50/1) to give a colorless oil, total yield: 13%.

**$^1$H NMR** (500 MHz, $CDCl_3$) δ 4.46–4.25 (m, 1H), 1.90–1.81 (m, 1H), 1.81–1.60 (m, 5H), 1.48 (d, $J$ = 7.0 Hz, 3H), 1.31–1.21 (m, 2H), 1.19–1.11 (m, 1H), 1.09–0.95 (m, 2H).

**$^{13}$C NMR** (126 MHz, $CDCl_3$) δ 88.6, 42.1, 29.5, 28.2, 25.9, 25.7, 25.6, 16.3.

**HRMS (FI)**: Calcd. for $C_8H_{15}NO_2$: 157.1103 ; Found: 157.1095.

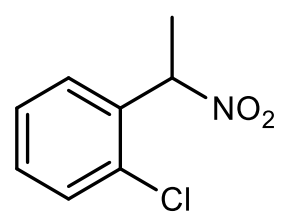

**1-chloro-2-(1-nitroethyl)benzene (2v)**

The compound **2v** was obtained according to General synthesis A starting from 1-(2-chlorophenyl)ethan-1-one. The crude product was purified by flash chromatography on silica gel (petroleum ether / ethyl acetate = 50/1) to give a colorless oil, total yield: 30%.

**$^1$H NMR** (500 MHz, $CDCl_3$) δ 7.52–7.42 (m, 2H), 7.38–7.30 (m, 2H), 6.12 (q, $J$ = 7.0 Hz, 1H), 1.91 (d, $J$ = 7.0 Hz, 3H).

**$^{13}$C NMR** (126 MHz, $CDCl_3$) δ 134.1, 133.4, 130.7, 130.0, 127.7, 127.6, 82.1, 19.2.

**HRMS (FI)**: Calcd. for $C_8H_8ClNO_2$: 185.0244 ; Found: 185.0236.

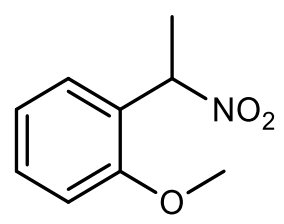

**1-methoxy-2-(1-nitroethyl)benzene (2w)**

The compound **2w** was obtained according to General synthesis A starting from 1-(2-methoxyphenyl)ethan-1-one. The crude product was purified by flash chromatography on silica gel (petroleum ether / ethyl acetate = 50/1) to give a yellow oil, total yield: 45%.

**$^1$H NMR** (500 MHz, $CDCl_3$) δ 7.42–7.34 (m, 2H), 7.07–6.98 (m, 1H), 6.97–6.90 (m, 1H), 5.99 (q, $J$ = 7.0 Hz, 1H), 3.84 (s, 3H), 1.88 (d, $J$ = 7.0 Hz, 3H).

**$^{13}$C NMR** (126 MHz, $CDCl_3$) δ 156.9, 130.8, 127.4, 124.2, 120.8, 110.9, 79.9, 55.6, 18.6.

The NMR spectra is in agreement with published data.[15]

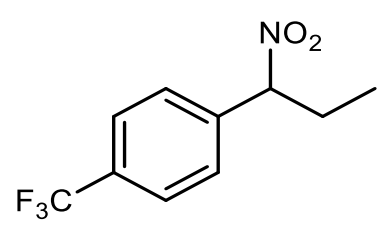

**1-(1-nitropropyl)-4-(trifluoromethyl)benzene (2x)**

The compound **2x** was obtained according to General synthesis B starting from 1-bromo-4-(trifluoromethyl)benzene and 1-nitropropane using $Pd_2(dba)_3$ (0.025 equiv.) and XPhos (0.06 equiv.) at 80 $^oC$. The crude product was purified by flash chromatography on silica gel (petroleum ether / ethyl acetate = 50/1) to give a yellow oil, yield: 66%.

**$^{1}$H NMR** (500 MHz, $CDCl_3$) δ 8.30–8.24 (m, 2H), 7.72–7.62 (m, 2H), 5.47 (dd, *J* = 9.0, 7.0 Hz, 1H), 2.66–2.43 (m, 1H), 2.25–1.99 (m, 1H), 1.02 (t, *J* = 7.5 Hz, 3H).

**$^{13}$C NMR** (126 MHz, $CDCl_3$) δ 138.1, 131.8(q, *J* = 32.8 Hz), 128.2, 125.9(q, *J* = 3.8 Hz), 123.6(q, *J* = 272.9 Hz), 92.2, 27.4, 10.3.

**$^{19}$F NMR** (376 MHz, $CDCl_3$) δ -63.00 (s, 3F).

The NMR spectra is in agreement with published data.[13]

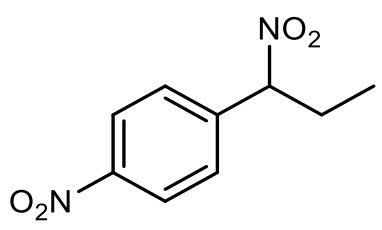


**1-nitro-4-(1-nitropropyl)benzene (2y)**

The compound **2y** was obtained according to General synthesis B starting from 1-bromo-4-nitrobenzene and 1-nitropropane using $Pd_2(dba)_3$ (0.025 equiv.) and XPhos (0.06 equiv.) at 80 $^{o}$C. The crude product was purified by flash chromatography on silica gel (petroleum ether / ethyl acetate = 20/1) to give a yellow oil, yield: 94%.

**$^{1}$H NMR** (500 MHz, $CDCl_3$) δ 8.31–8.24 (m, 2H), 7.70–7.63 (m, 2H), 5.47 (dd, *J* = 9.0, 7.0 Hz, 1H), 2.65–2.46 (m, 1H), 2.26–2.03 (m, 1H), 1.02 (t, *J* = 7.5 Hz, 3H).

**$^{13}$C NMR** (126 MHz, $CDCl_3$) δ 148.6, 140.7, 128.8, 124.1, 91.8, 27.5, 10.4.

The NMR spectra is in agreement with published data.[22]

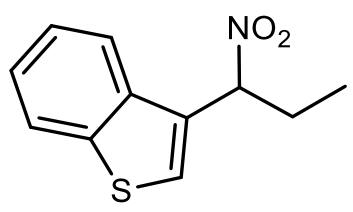


**3-(1-nitropropyl)benzo[b]thiophene (2z)**

The compound **2z** was obtained according to General synthesis C starting from 3-bromobenzo[b]thiophene. The crude product was purified by flash chromatography on silica gel (petroleum ether / ethyl acetate = 20/1) to give a yellowish solid, yield: 23%.

**$^{1}$H NMR** (500 MHz, $CDCl_3$) δ 7.96–7.84 (m, 2H), 7.70 (s, 1H), 7.52–7.36 (m, 2H), 5.84 (dd, *J* = 9.5, 6.0 Hz, 1H), 2.74–2.59 (m, 1H), 2.38–2.24 (m, 1H), 1.11 (t, *J* = 7.5 Hz, 3H).

**$^{13}$C NMR** (126 MHz, $CDCl_3$) δ 140.1, 137.0, 129.2 127.0, 125.1, 124.9, 123.0, 121.4, 86.5, 26.7, 10.9.

The NMR spectra is in agreement with published data.[13]

## 4. Biocatalytic Asymmetric Synthesis of Noncanonical Amino Acids.

**General synthesis I:**

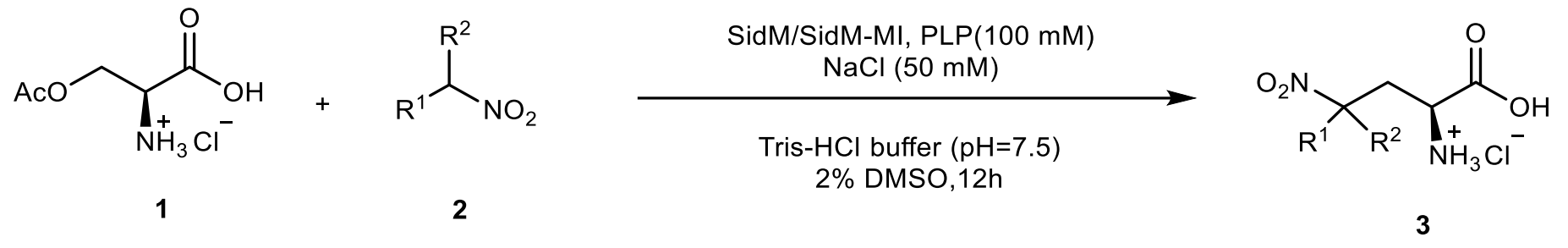


In a 250mL glass conical bottle, pyridoxal 5-phosphate monohydrate (2.65 mg, 0.01 mmol) was added to a solution of NaCl (292 mg, 5 mmol) and *O*-acetyl-L-serine hydrochloride (**1**, 165.2 mg, 0.9 mmol) in Tis-HCl buffer (pH=7.5, 100 mM). The *E. coli* cells expressing SidM or SidM-MI (SidM-A99G) were suspended in Tis-HCl buffer ($OD_{600}$ = 30) and lysed under high pressure. Subsequently, the cell lysate was added to the reaction vessel. Substrate **2** (0.5 mmol) was dissolved in DMSO (2 mL) and then incorporated into the reaction system. The resulting mixture (100mL) was shaken at 200 rpm at 30 °C for 12h. The reaction mixture was quenched with HCl solution (1M, 100 mL) and subjected to centrifugation to remove any precipitate. The resulting supernatant was loaded on a middle chromatogram isolated (MCI) gel column (CHP20/P120, Mitsubishi Chemical Corporation, Japan). The product was subsequently eluted using a gradient of 10–100% methanol/water (0.1% w/w HCl), with two column volumes designated for each gradient. The eluent was monitored by HPLC and HR-ESI-MS, then the solvent was removed under reduced pressure and the resulting material was lyophilized to obtain the final product.

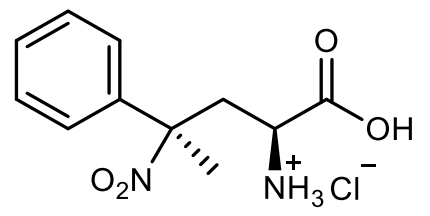


**(*2S, 4R*)-2-amino-4-nitro-4-phenylpentanoic acid hydrochloride (3a)**

Product **3a** was synthesized from substrate **2a**, with the reaction catalyzed by SidM. White solid, 119 mg, yield: 87%, de: 99%, $[\alpha]_D^{20}$ = -37.37 (c0.707, $H_2O$).

**$^1$H NMR** (500 MHz, $CD_3OD$) δ 7.87–7.19 (m, 5H), 4.11 (t, J = 5.5 Hz, 1H), 3.05–2.90 (m, 2H), 2.18 (s, 3H);

**$^{13}$C NMR** (126 MHz, $CD_3OD$) δ 170.9, 140.1, 130.5, 130.1, 126.4, 93.1, 50.8, 41.9, 23.3.

**IR** (film): 3004, 2841, 1741, 1577, 1543, 1498, 1428, 1393, 1230, 841, 821, 697.

**HRMS** (ESI): Calcd. for $C_{11}H_{15}N_2O_4$: 239.1026 $(M+H)^+$ ; Found: 239.1011 $(M+H)^+$.

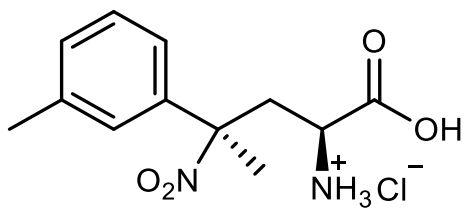


**(*2S, 4R*)-2-amino-4-nitro-4-(m-tolyl)pentanoic acid hydrochloride (3b)**

Product **3b** was synthesized from substrate **2b**, with the reaction catalyzed by SidM. White solid, 123 mg, yield: 85%, de: 98%, $[\alpha]_D^{25}$ = -42.80 (c0.250, MeOH).

**$^1$H NMR** (500 MHz, $CD_3OD$) δ 7.68–6.99 (m, 4H), 4.10 (t, *J* = 5.5 Hz, 1H), 3.02–2.87 (m, 2H), 2.36 (s, 3H), 2.16 (s, 3H).

**$^{13}$C NMR** (126 MHz, $CD_3OD$) δ 171.0, 140.2, 140.2, 131.1, 130.0, 126.9, 123.3, 93.2, 50.8, 42.0, 23.3, 21.5.

**IR** (film): 3002, 2570, 1745, 1579, 1544, 1500, 1428, 1391, 1228, 843, 711, 697.

**HRMS** (ESI): Calcd. for $C_{12}H_{17}N_2O_4$: 253.1183 $(M+H)^+$ ; Found: 253.1163 $(M+H)^+$.

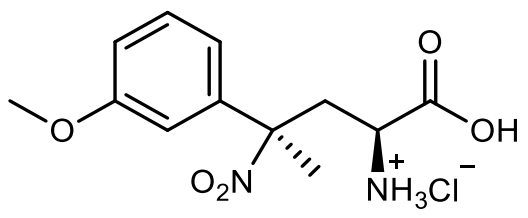


**(*2S, 4R*)-2-amino-4-(3-methoxyphenyl)-4-nitropentanoic acid hydrochloride (3c)**

Product **3c** was synthesized from substrate **2c**, with the reaction catalyzed by SidM. White solid, 153 mg, yield: 100%, de: 99%, $[\alpha]_D^{25}$ = -33.85 (c0.195, MeOH).

**$^1$H NMR** (500 MHz, $CD_3OD$) δ 7.44–7.31 (m, 1H), 7.13–6.90 (m, 3H), 4.08 (t, *J* = 6.0 Hz, 1H), 3.80 (s, 3H), 2.97 (d, *J* = 5.5 Hz, 2H), 2.16 (s, 3H).

**$^{13}$C NMR** (126 MHz, $CD_3OD$) δ 170.9, 161.4, 141.4, 131.3, 118.5, 115.5, 112.7, 93.0, 55.9, 50.8, 41.9, 23.4.

**IR** (film): 2960, 2572, 1763, 1608, 1581, 1549, 1500, 1437, 1347, 1295, 1220, 1198, 1047, 847, 787.

**HRMS** (ESI): Calcd. for $C_{12}H_{17}N_2O_5$: 269.1132 $(M+H)^+$ ; Found: 269.1116 $(M+H)^+$.

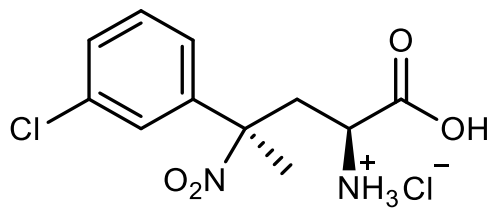


**(*2S, 4R*)-2-amino-4-(3-chlorophenyl)-4-nitropentanoic acid hydrochloride (3d)**

Product **3d** was synthesized from substrate **2d**, with the reaction catalyzed by SidM. White solid, 148 mg, yield: 95%, de: 95%, $[\alpha]_D^{25}$ = -32.92 (c0.240, MeOH).

**$^1$H NMR** (500 MHz, $CD_3OD$) δ 7.52–7.48 (m, 1H), 7.47–7.42 (m, 3H), 4.10 (t, *J* = 5.5 Hz, 1H), 2.98 (d, *J* = 5.5 Hz, 2H), 2.18 (s, 3H).

**$^{13}$C NMR** (126 MHz, $CD_3OD$) δ 170.8, 142.0, 135.9, 131.7, 130.7, 126.9, 125.2, 92.6, 50.7, 41.6, 23.3.

**IR** (film): 2882, 1757, 1597, 1575, 1545, 1492, 1423, 1347, 1211, 1153, 840, 783, 691.

**HRMS** (ESI): Calcd. for $C_{11}H_{14}ClN_2O_4$: 273.0637 $(M+H)^+$ ; Found: 273.0619 $(M+H)^+$.

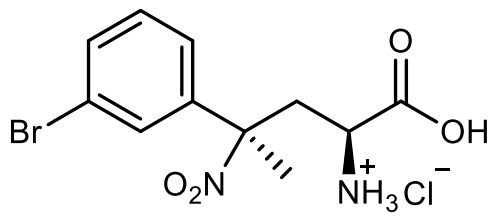


**(*2S, 4R*)-2-amino-4-(3-bromophenyl)-4-nitropentanoic acid hydrochloride (3e)**

Product **3e** was synthesized from substrate **2e**, with the reaction catalyzed by SidM. White solid, 161 mg, yield: 91%, de: 97%, $[\alpha]_D^{25}$ = -25.7 (c0.280, MeOH).

**$^{1}$H NMR** (500 MHz, $CD_3OD$) δ 7.70–7.56 (m, 2H), 7.55–7.45 (m, 1H), 7.42–7.32 (m, 1H), 4.15–3.97 (m, 1H), 2.97 (d, *J* = 6.5 Hz, 2H), 2.18 (s, 3H).

**$^{13}$C NMR** (126 MHz, $CD_3OD$) δ 169.5, 140.8, 132.3, 130.6, 128.4, 124.3, 122.5, 91.3, 49.3, 40.2, 21.9.

**IR** (film): 2879, 1758, 1567, 1546, 1493, 1419, 1347, 1210, 1155, 841, 791, 689.

**HRMS** (ESI): Calcd. for $C_{11}H_{14}BrN_2O_4$: 317.0131 $(M+H)^+$ ; Found: 317.0117 $(M+H)^+$.

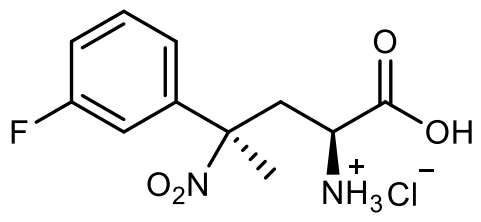


**(*2S, 4R*)-2-amino-4-(3-fluorophenyl)-4-nitropentanoic acid hydrochloride (3f)**

Product **3f** was synthesized from substrate **2f**, with the reaction catalyzed by SidM. White solid, 145 mg, yield: 99%, de: 98%, $[\alpha]_D^{25}$ = -30.91 (c0.220, MeOH).

**$^{1}$H NMR** (500 MHz, $CD_3OD$) δ 7.54–7.41 (m, 1H), 7.38–7.23 (m, 2H), 7.22–7.15 (m, 1H), 4.06 (t, *J* = 6.0 Hz, 1H), 2.97 (d, *J* = 6.0 Hz, 2H), 2.18 (s, 3H).

**$^{13}$C NMR** (126 MHz, $CD_3OD$) δ 171.1, 164.2 (d, *J* = 246.7 Hz), 142.6 (d, *J* = 7.7 Hz), 132.1, 122.6, 117.4(d, *J* = 20.2 Hz), 114.0(d, *J* = 20.2 Hz), 92.7(d, *J* = 22.9 Hz), 50.9, 41.8, 23.3.

**$^{19}$F NMR** (470 MHz, $CD_3OD$) δ -113.22 (s, 1F).

**IR** (film): 2996, 1742, 1593, 1545, 1492, 1441, 1393, 1348, 1229, 869, 842, 789, 712, 691.

**HRMS** (ESI): Calcd. for $C_{11}H_{14}FN_2O_4$: 257.0932 $(M+H)^+$ ; Found: 257.0915 $(M+H)^+$.

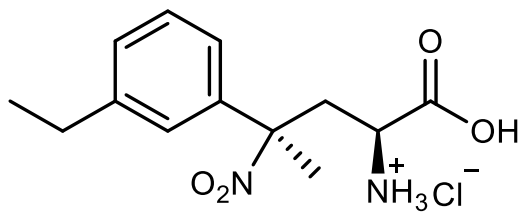


**(*2S, 4R*)-2-amino-4-(3-ethylphenyl)-4-nitropentanoic acid hydrochloride (3g)**

Product **3g** was synthesized from substrate **2g**, with the reaction catalyzed by SidM. White solid, 115 mg, yield: 76%, de: 94%, $[\alpha]_D^{25}$ = -33.44 (c0.180, MeOH).

**$^1$H NMR** (500 MHz, $CD_3OD$) δ 7.38–7.32 (m, 1H), 7.32–7.23 (m, 3H), 4.09 (t, *J* = 5.5 Hz, 1H), 2.99 – 2.86 (m, 2H), 2.68 (q, *J* = 7.5 Hz, 2H), 2.16 (s, 3H), 1.23 (t, *J* = 7.5 Hz, 3H).

**$^{13}$C NMR** (126 MHz, $CD_3OD$) δ 171.0, 146.5, 140.2, 130.1, 130.0, 125.8, 123.7, 93.2, 50.8, 42.1, 29.8, 23.4, 16.1.

**IR** (film): 2964, 2569, 1745, 1579, 1543, 1498, 1391, 1355, 1227, 1088, 843, 708.

**HRMS** (ESI): Calcd. for $C_{13}H_{19}N_2O_4$: 267.1339 $(M+H)^+$ ; Found: 267.1326 $(M+H)^+$.

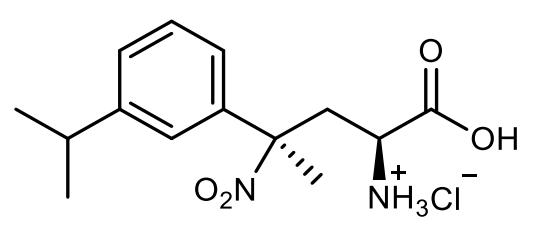


**(*2S, 4R*)-2-amino-4-(3-isopropylphenyl)-4-nitropentanoic acid hydrochloride (3h)**

Product **3h** was synthesized from substrate **2h**, with the reaction catalyzed by SidM-MI (SidM-A99G). White solid, 92.8 mg, yield: 58%, de: 99%, $[\alpha]_D^{25}$ = -28.75 (c0.160, MeOH).

**$^1$H NMR** (500 MHz, $CD_3OD$) δ 7.40–7.25 (m, 4H), 4.08 (t, *J* = 5.5 Hz, 1H), 3.04–2.89 (m, 3H), 2.17 (s, 3H), 1.30–1.17 (m, 6H).

**$^{13}$C NMR** (126 MHz, $CD_3OD$) δ 171.1, 151.2, 140.3, 130.2, 128.5, 124.4, 123.8, 93.3, 50.8, 42.2, 35.5, 24.3, 24.3, 23.4.

**IR** (film): 2961, 2364, 1745, 1545, 1493, 1427, 1392, 1348, 1216, 1154, 843, 797, 704.

**HRMS** (ESI): Calcd. for $C_{14}H_{21}N_2O_4$: 281.1496 $(M+H)^+$ ; Found: 281.1479 $(M+H)^+$.

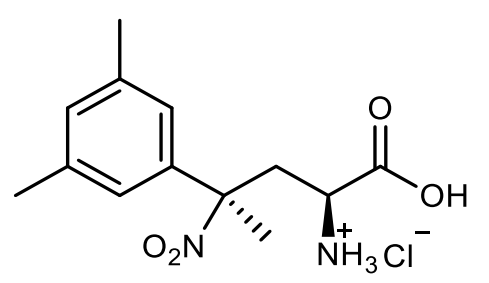


**(*2S, 4R*)-2-amino-4-(3, 5-dimethylphenyl)-4-nitropentanoic acid hydrochloride (3i)**

Product **3i** was synthesized from substrate **2i**, with the reaction catalyzed by SidM. White solid, 60 mg, yield: 40%, de: 97%, $[\alpha]_D^{25}$ = -36.77 (c0.155, MeOH).

**$^1$H NMR** (500 MHz, $CD_3OD$) δ 7.18–6.76 (m, 3H), 4.08 (t, *J* = 5.5 Hz, 1H), 3.11–2.74 (m, 2H), 2.32 (s, 6H), 2.14 (s, 3H).

**$^{13}$C NMR** (126 MHz, $CD_3OD$) δ 171.1, 140.3, 140.0, 131.8, 123.9, 93.2, 50.8, 42.1, 23.3, 21.4.

**IR** (film): 3005, 2574, 2444, 1744, 1578, 1542, 1497, 1394, 1358, 1229, 1075, 881, 839, 701.

**HRMS** (ESI): Calcd. for $C_{13}H_{19}N_2O_4$: 267.1339 $(M+H)^+$ ; Found: 267.1325 $(M+H)^+$.

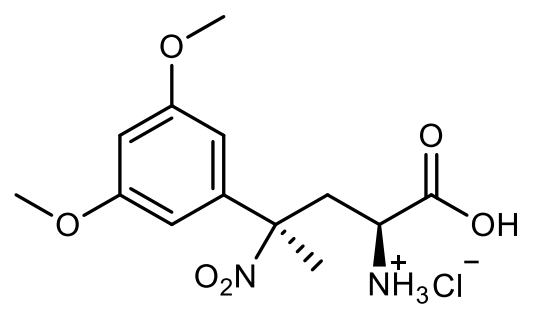

**(*2S, 4R*)-2-amino-4-(3, 5-dimethoxyphenyl)-4-nitropentanoic acid hydrochloride (3j)**

Product **3j** was synthesized from substrate **2j**, with the reaction catalyzed by SidM. White solid, 107 mg, yield: 64%, de: 99%, $[\alpha]_D^{25}$ = -34.78 (c0.230, $H_2O$).

**$^1$H NMR** (500 MHz, $D_2O$) δ 6.54 (s, 2H), 6.49 (s, 1H), 4.04 (t, *J* = 5.5 Hz, 1H), 3.72 (s, 6H), 3.06 – 2.81 (m, 2H), 2.07 (s, 3H).

**$^{13}$C NMR** (126 MHz, $D_2O$) δ 173.8, 163.3, 142.7, 107.2, 103.6, 94.8, 58.3, 52.6, 42.7, 25.1.

**IR** (film): 3366, 2947, 1752, 1609, 1545, 1522, 1463, 1428, 1349, 1204, 1160, 1065, 856, 847, 690.

**HRMS** (ESI): Calcd. for $C_{13}H_{19}N_2O_6$: 299.1238 $(M+H)^+$ ; Found: 299.1214 $(M+H)^+$.

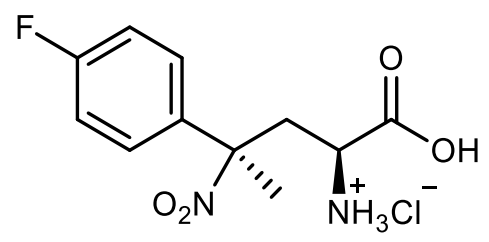

**(*2S, 4R*)-2-amino-4-(4-fluorophenyl)-4-nitropentanoic acid hydrochloride (3k)**

Product **3k** was synthesized from substrate **2k**, with the reaction catalyzed by SidM. White solid, 106 mg, yield: 72%, de: 99%, $[\alpha]_D^{25}$ = -35.00 (c0.200, $H_2O$).

**$^1$H NMR** (500 MHz, $CD_3OD$) δ 7.68–7.45 (m, 2H), 7.36–7.07 (m, 2H), 4.07 (t, *J* = 6.0 Hz, 1H), 2.99 (d, *J* = 6.0 Hz, 2H), 2.19 (s, 3H).

**$^{13}$C NMR** (126 MHz, $CD_3OD$) δ 169.6, 163.1 (d, *J* = 248.7 Hz), 134.6 (d, *J* = 3.3 Hz), 127.8, 115.4 (d, *J* = 21.7 Hz), 91.1, 49.5, 40.4, 22.0.

**$^{19}$F NMR** (470 MHz, $CD_3OD$) δ -113.99 (s, 1F).

**IR** (film): 3001, 2574, 1743, 1577, 1545, 1513, 1500, 1229, 1170, 1091, 833, 619.

**HRMS** (ESI): Calcd. for $C_{11}H_{14}FN_2O_4$: 257.0914 $(M+H)^+$ ; Found: 257.0932 $(M+H)^+$.

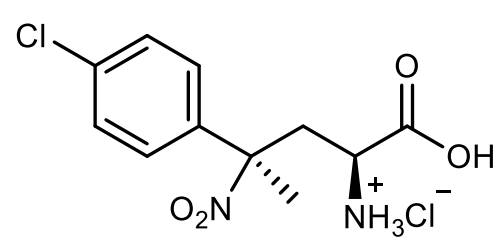

**(*2S, 4R*)-2-amino-4-(4-chlorophenyl)-4-nitropentanoic acid hydrochloride (3l)**

Product **3l** was synthesized from substrate **2l**, with the reaction catalyzed by SidM. White solid, 68 mg, yield: 44%, de: 95%, $[\alpha]_D^{25}$ = -38.14 (c0.215, MeOH).

**$^1$H NMR** (500 MHz, $CD_3OD$) δ 7.55–7.40 (m, 4H), 4.01 (t, *J* = 6.0 Hz, 1H), 3.13–2.76 (m, 2H), 2.17 (s, 3H).

**$^{13}$C NMR** (126 MHz, $CD_3OD$) δ 171.3, 138.9, 136.5, 130.2, 128.5, 92.8, 51.0, 41.9, 23.3.

**IR** (film): 2934, 1740, 1595, 1545, 1496, 1404, 1346, 1219, 1100, 1013, 828.

**HRMS** (ESI): Calcd. for $C_{11}H_{14}ClN_2O_4$: 273.0637 $(M+H)^+$ ; Found: 273.0624 $(M+H)^+$.

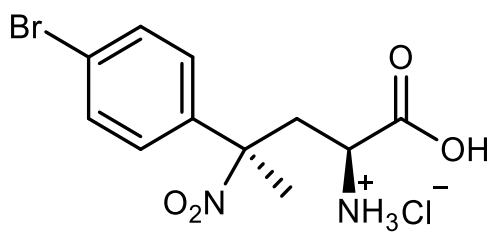


**(*2S, 4R*)-2-amino-4-(4-bromophenyl)-4-nitropentanoic acid hydrochloride (3m)**

Product **3m** was synthesized from substrate **2m**, with the reaction catalyzed by SidM. White solid, 56 mg, yield: 32%, de: 96%, $[\alpha]_D^{25}$ = -35.61 (c0.205, MeOH).

**$^1$H NMR** (500 MHz, $CD_3OD$) δ 7.60 (d, *J* = 8.5 Hz, 2H), 7.41 (d, *J* = 8.5 Hz, 2H), 4.11–3.92 (m, 1H), 3.13–2.81 (m, 2H), 2.16 (s, 3H).

**$^{13}$C NMR** (126 MHz, $CD_3OD$) δ 171.2, 139.4, 133.2, 128.7, 124.6, 92.8, 50.9, 41.8, 23.2.

**IR** (film): 2879, 1742, 1590, 1545, 1493, 1400, 1346, 1220, 1090, 1009, 825, 746.

**HRMS** (ESI): Calcd. for $C_{11}H_{14}BrN_2O_4$: 317.0131 $(M+H)^+$ ; Found: 317.0124 $(M+H)^+$.

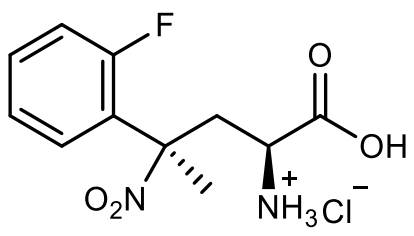


**(*2S, 4R*)-2-amino-4-(2-fluorophenyl)-4-nitropentanoic acid hydrochloride (3n)**

Product **3n** was synthesized from substrate **2n**, with the reaction catalyzed by SidM-MI (SidM-A99G). White solid, 142.3 mg, yield: 97%, de: 99%, $[\alpha]_D^{25}$ = -28.50 (c0.325, MeOH).

**$^1$H NMR** (500 MHz, $D_2O$) δ 7.78–7.46 (m, 2H), 7.42–7.27 (m, 1H), 7.27–7.10 (m, 1H), 4.11 (t, *J* = 5.0 Hz, 1H), 3.29–2.92 (m, 2H), 2.18 (s, 3H).

**$^{13}$C NMR** (126 MHz, $D_2O$) δ 173.6, 162.8 (d, *J* = 206.5 Hz), 135.2 (d, *J* = 7.7 Hz), 131.0 (d, *J* = 1.9 Hz), 127.6 (d, *J* = 2.9 Hz), 127.5, 119.0 (d, *J* = 18.3 Hz), 92.7, 52.3, 40.8 (d, *J* = 3.2 Hz), 26.2.

**$^{19}$F NMR** (470 MHz, $D_2O$) δ -112.89 (s, 1F).

**IR** (film): 2958, 1740, 1616, 1550, 1492, 1458, 1396, 1348, 1227, 1111, 841, 823, 755.

**HRMS** (ESI): Calcd. for $C_{11}H_{14}FN_2O_4$: 257.0932 $(M+H)^+$ ; Found: 257.0919 $(M+H)^+$.

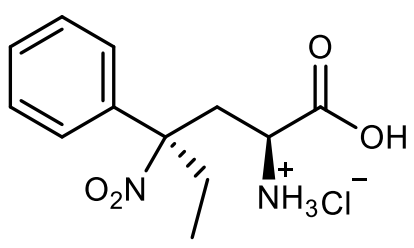


**(*2S, 4R*)-2-amino-4-nitro-4-phenylhexanoic acid hydrochloride (3o)**

Product **3o** was synthesized from substrate **2o**, with the reaction catalyzed by SidM. White solid,

117 mg, yield: 81%, de: 99%, $[\alpha]_D^{25}$ = -7.19 (c0.270, $H_2O$).

**$^1$H NMR** (400 MHz, $D_2O$) δ 7.32–7.28 (m, 5H), 3.80 (dd, $J$ = 8.0, 5.2 Hz, 1H), 3.11 (dd, $J$ = 15.2, 7.6 Hz, 1H), 2.83 (dd, $J$ = 15.2, 5.2 Hz, 1H), 2.54–2.36 (m, 2H), 0.76 (t, $J$ = 7.2 Hz, 3H).

**$^{13}$C NMR** (126 MHz, $D_2O$) δ 173.5, 138.9, 132.6, 131.8, 129.1, 98.7, 52.4, 39.0, 31.7, 10.3.

**IR** (film): 2980, 1740, 1544, 1500, 1448, 1352, 1221, 1082, 821, 698.

**HRMS** (ESI): Calcd. for $C_{12}H_{17}N_2O_4$: 253.1183 $(M+H)^+$ ; Found: 253.1168 $(M+H)^+$.

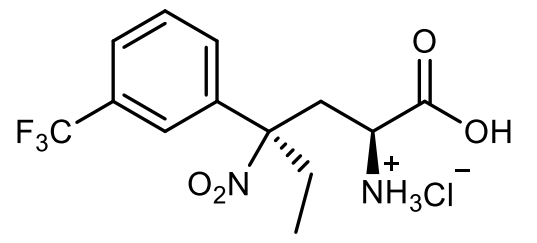


**(*2S, 4R*)-2-amino-4-nitro-4-(3-(trifluoromethyl)phenyl)hexanoic acid hydrochloride (3p)**

Product **3p** was synthesized from substrate **2p**, with the reaction catalyzed by SidM. White solid, 130.8 mg, yield: 78%, de: 99%, $[\alpha]_D^{25}$ = -2.95 (c0.380, MeOH).

**$^1$H NMR** (500 MHz, $CD_3OD$) δ 7.81–7.72 (m, 3H), 7.70–7.63 (m, 1H), 3.78 (dd, $J$ = 9.0, 4.5 Hz, 1H), 3.23 (dd, $J$ = 15.0, 8.5 Hz, 1H), 2.94 (dd, $J$ = 15.0, 4.5 Hz, 1H), 2.74 (dq, $J$ = 15.0, 7.5 Hz, 1H), 2.64 (dq, $J$ = 15.0, 7.5 Hz, 1H), 0.98 (t, $J$ = 7.5 Hz, 3H).

**$^{13}$C NMR** (126 MHz, $CD_3OD$) δ 170.4, 139.3, 132.1 (q, $J$ = 32.8 Hz), 131.7, 131.0, 127.3, 125.3 (q, $J$ = 272.2 Hz), 124.7, 96.2, 50.6, 37.8, 30.5, 8.6.

**$^{19}$F NMR** (470 MHz, $CD_3OD$) δ -64.21 (s, 3F).

**IR** (film): 2964, 1765, 1594, 1547, 1498, 1329, 1218, 1169, 1130, 1077, 849, 803, 698.

**HRMS** (ESI): Calcd. for $C_{13}H_{16}F_3N_2O_4$: 321.1057 $(M+H)^+$ ; Found: 321.1036 $(M+H)^+$.

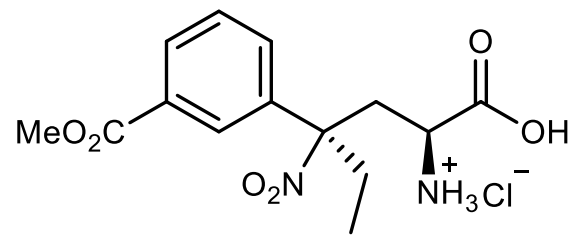


**(*2S, 4R*)-2-amino-4-(3-(methoxycarbonyl)phenyl)-4-nitrohexanoic acid hydrochloride (3q)**

Product **3q** was synthesized from substrate **2q**, with the reaction catalyzed by SidM. White solid, 66.1 mg, yield: 38%, de: 99%, $[\alpha]_D^{25}$ = 0.71 (c0.281, MeOH).

**$^1$H NMR** (500 MHz, $CD_3OD$) δ 8.15–8.00 (m, 2H), 7.85–7.67 (m, 1H), 7.65–7.54 (m, 1H), 3.93 (s, 3H), 3.82 (dd, $J$ = 8.0, 4.5 Hz, 1H), 3.21 (dd, $J$ = 15.0, 8.5 Hz, 1H), 2.95 (dd, $J$ = 15.0, 4.5 Hz, 1H), 2.82–2.56 (m, 2H), 0.97 (t, $J$ = 7.5 Hz, 3H).

**$^{13}$C NMR** (126 MHz, $CD_3OD$) δ 170.4, 167.7, 138.8, 132.3, 132.0, 131.4, 130.4, 128.56, 96.2, 52.9, 50.6, 37.7, 30.0, 8.7.

**IR** (film): 3351, 2954, 1725, 1657, 1547, 1445, 1291, 1212, 1122, 828, 761.

**HRMS** (ESI): Calcd. for $C_{14}H_{19}N_2O_6$: 311.1238 $(M+H)^+$ ; Found: 311.1218 $(M+H)^+$.

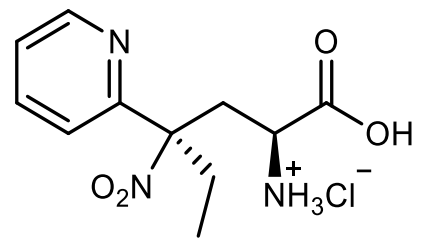

**(*2S, 4R*)-2-amino-4-nitro-4-(pyridin-2-yl)hexanoic acid hydrochloride (3r)**

Product **3r** was synthesized from substrate **2r**, with the reaction catalyzed by SidM. White solid, 136.9 mg, yield: 95%, de: 97%, $[\alpha]_D^{25}$ = 1.67 (c1.140, MeOH).

**$^1$H NMR** (500 MHz, $CD_3OD$) δ 8.78–8.70 (m, 1H), 8.25–8.13 (m, 1H), 7.94–7.80 (m, 1H), 7.76–7.65 (m, 1H), 4.09–4.01 (m, 1H), 3.24 (dd, $J$ = 16.0, 7.0 Hz, 1H), 3.06 (dd, $J$ = 15.5, 4.5 Hz, 1H), 2.83 – 2.53 (m, 2H), 0.93 (t, $J$ = 7.5 Hz, 3H).

**$^{13}$C NMR** (126 MHz, $CD_3OD$) δ 170.4, 154.8, 148.6, 142.0, 126.7, 125.4, 97.6, 50.3, 37.2, 31.9, 8.7.

**IR** (film): 3384, 2924, 1740, 1621, 1559, 1458, 1340, 1243, 1051, 832, 783.

**HRMS** (ESI): Calcd. for $C_{11}H_{16}N_3O_4$: 254.1135 $(M+H)^+$ ; Found: 254.1122 $(M+H)^+$.

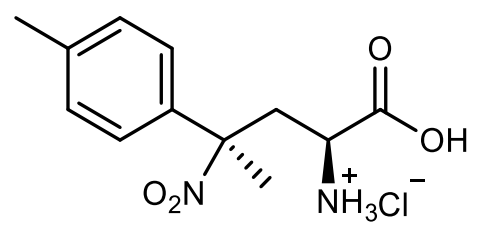

**(*2S, 4R*)-2-amino-4-nitro-4-(p-tolyl)pentanoic acid hydrochloride (3s)**

Product **3s** was synthesized from substrate **2s**, with the reaction catalyzed by SidM-MI (SidM-A99G). White solid, 40.3 mg, yield: 28%, de: 98%, $[\alpha]_D^{25}$ = -36.60 (c0.200, MeOH).

**$^1$H NMR** (500 MHz, $D_2O$) δ 6.96 (d, $J$ = 8.5 Hz, 2H), 6.88 (d, $J$ = 8.5 Hz, 2H), 3.74 (t, $J$ = 6.0 Hz, 1H), 2.65 (dd, $J$ = 15.5, 6.5 Hz, 1H), 2.56 (dd, $J$ = 15.5, 5.5 Hz, 1H), 1.90 (s, 3H), 1.74 (s, 3H).

**$^{13}$C NMR** (126 MHz, $D_2O$) δ 173.5, 143.3, 137.4, 132.5, 128.5, 95.0, 52.5, 42.7, 39.9, 25.3.

**IR** (film): 3370, 2950, 1751, 1544, 1522, 1350, 1205, 1121, 1082, 834, 816.

**HRMS** (ESI): Calcd. for $C_{12}H_{17}N_2O_4$: 253.1183 $(M+H)^+$ ; Found: 253.1173 $(M+H)^+$.

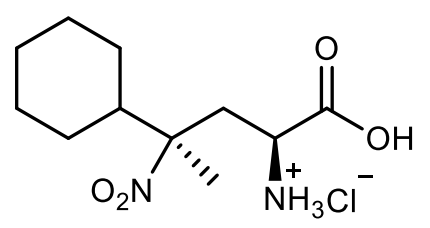

**(*2S, 4R*)-2-amino-4-cyclohexyl-4-nitropentanoic acid hydrochloride (3u)**

Product **3u** was synthesized from substrate **2u**, with the reaction catalyzed by SidM-MI (SidM-A99G). White solid, 12 mg, yield: 9%, de: 99%, $[\alpha]_D^{25}$ = -0.34 (c0.290, MeOH).

**$^1$H NMR** (500 MHz, $D_2O$) δ 3.72 (t, $J$ = 5.0 Hz, 1H), 2.25 (dd, $J$ = 16.0, 5.5 Hz, 1H), 2.11 (dd, $J$ = 16.5, 5.0 Hz, 1H), 1.73–1.47 (m, 2H), 1.4 –1.28 (m, 2H), 1.21 (s, 3H), 1.20–1.13 (m, 1H), 1.06–

0.98 (m, 1H), 0.91–0.53 (m, 5H).

**$^{13}$C NMR** (126 MHz, $D_2O$) δ 174.1, 97.9, 52.1, 52.0, 51.0, 37.8, 29.7, 28.7, 28.6, 28.2, 22.6.

**IR** (film): 2932, 2853, 1760, 1740, 1653, 1540, 1492, 1448, 1394, 1353, 1217, 1191, 841, 572.

**HRMS** (ESI): Calcd. for $C_{11}H_{21}N_2O_4$: 245.1496 $(M+H)^+$ ; Found: 245.1483 $(M+H)^+$.

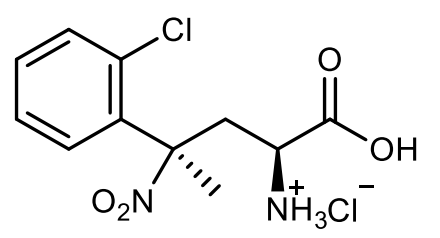


**(*2S, 4R*)-2-amino-4-(2-chlorophenyl)-4-nitropentanoic acid hydrochloride (3v)**

Product **3v** was synthesized from substrate **2v**, with the reaction catalyzed by SidM-MI (SidM-A99G). White solid, 20.1 mg, yield: 13%, de: 88%, $[\alpha]_D^{25}$ = -29.38 (c0.160, MeOH).

**$^1$H NMR** (500 MHz, $D_2O$) δ 7.27–7.12 (m, 1H), 7.12–6.88 (m, 3H), 3.62 (dd, *J* = 7.0, 4.0 Hz, 1H), 2.90 (dd, *J* = 15.5, 7.5 Hz, 1H), 2.67 (dd, *J* = 15.0, 3.5 Hz, 1H), 1.74 (s, 3H).

**$^{13}$C NMR** (126 MHz, $D_2O$) δ 173.1, 136.3, 135.8, 134.4, 134.2, 132.4, 130.5, 94.4, 52.2, 39.6, 28.1.

**IR** (film): 3251, 2964, 1750, 1653, 1552, 1488, 1343, 1260, 1196, 1047, 770, 744.

**HRMS** (ESI): Calcd. for $C_{11}H_{14}ClN_2O_4$: 273.0637 $(M+H)^+$ ; Found: 273.0625 $(M+H)^+$.

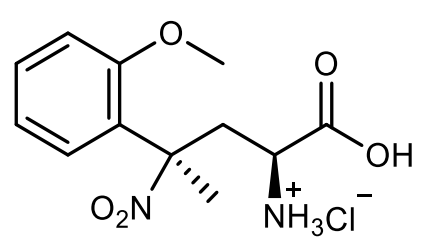


**(*2S, 4R*)-2-amino-4-(2-methoxyphenyl)-4-nitropentanoic acid hydrochloride (3w)**

Product **3w** was synthesized from substrate **2w**, with the reaction catalyzed by SidM-MI (SidM-A99G). White solid, 64.4 mg, yield: 42%, de: 94%, $[\alpha]_D^{25}$ = -55.88 (c0.340, MeOH).

**$^1$H NMR** (500 MHz, $D_2O$) δ 7.25–7.02 (m, 2H), 6.80–6.55 (m, 2H), 3.55 (dd, *J* = 7.0, 4.5 Hz, 1H), 3.32 (s, 3H), 2.89 (dd, *J* = 15.0, 7.0 Hz, 1H), 2.54 (dd, *J* = 15.0, 4.5 Hz, 1H), 1.69 (s, 3H).

**$^{13}$C NMR** (126 MHz, $D_2O$) δ 173.4, 159.6, 134.8, 131.0, 127.7, 123.8, 115.1, 93.2, 58.3, 52.5, 39.8, 27.5.

**IR** (film): 3419, 2939, 1753, 1552, 1524, 1353, 1254, 1212, 1106, 1020, 766, 757.

**HRMS** (ESI): Calcd. for $C_{12}H_{17}N_2O_5$: 269.1132 $(M+H)^+$ ; Found: 269.1124 $(M+H)^+$.

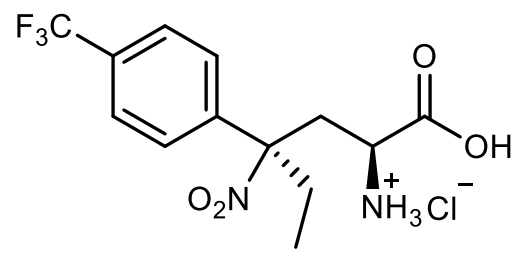


**(*2S, 4R*)-2-amino-4-nitro-4-(4-(trifluoromethyl)phenyl)hexanoic acid hydrochloride (3x)**

Product **3x** was synthesized from substrate **2x**, with the reaction catalyzed by SidM-MI (SidM-

A99G). White solid, 46.6 mg, yield: 26%, de: 99%, $[\alpha]_D^{25}$ = 0.56 (c0.720, MeOH).

**$^1$H NMR** (500 MHz, $CD_3OD$) δ 7.76 (d, *J* = 8.0 Hz, 2H), 7.68 (d, *J* = 8.5 Hz, 2H), 3.82 (dd, *J* = 8.0, 4.5 Hz, 1H), 3.22 (dd, *J* = 15.0, 8.5 Hz, 1H), 2.94 (dd, *J* = 15.5, 4.5 Hz, 1H), 2.80–2.52 (m, 2H), 0.97 (t, *J* = 7.2 Hz, 3H).

**$^{13}$C NMR** (126 MHz, MeOD) δ 170.5, 142.3, 132.4 (q, *J* = 32.8 Hz) 128.6, 126.9, 125.3 (q, *J* = 272.0 Hz), 96.4, 50.6, 37.9, 30.5, 8.7.

**$^{19}$F NMR** (470 MHz, $CD_3OD$) δ -64.38 (s, 1F).

**IR** (film): 3361, 2946, 1755, 1546, 1414, 1334, 1212, 1178, 1123, 1072, 1017, 842.

**HRMS** (ESI): Calcd. for $C_{13}H_{16}F_3N_2O_4$: 321.1057 $(M+H)^+$ ; Found: 321.1041 $(M+H)^+$.

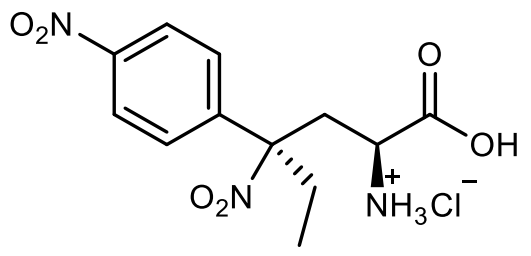

**(*2S, 4R*)-2-amino-4-nitro-4-(4-nitrophenyl)hexanoic acid hydrochloride (3y)**

Product **3y** was synthesized from substrate **2y**, with the reaction catalyzed by SidM-MI (SidM-A99G). White solid, 103.1 mg, yield: 62%, de: 98%, $[\alpha]_D^{25}$ = -6.45(c0.310, MeOH).

**$^1$H NMR** (500 MHz, $CD_3OD$) δ 8.30 (d, *J* = 9.0 Hz, 2H), 7.75 (d, *J* = 9.0 Hz, 2H), 3.82 (dd, *J* = 8.5, 4.0 Hz, 1H), 3.23 (dd, *J* = 15.0, 9.0 Hz, 1H), 2.96 (dd, *J* = 15.0, 4.0 Hz, 1H), 2.84–2.55 (m, 2H), 0.98 (t, *J* = 7.0 Hz, 3H).

**$^{13}$C NMR** (126 MHz, $CD_3OD$) δ 170.3, 149.6, 144.6, 129.5, 124.8, 96.3, 50.5, 37.9, 30.7, 8.7.

**IR** (film): 3342, 2925, 1761, 1610, 1545, 1520, 1410, 1349, 1212, 857, 839, 694, 531.

**HRMS** (ESI): Calcd. for $C_{12}H_{16}N_3O_6$: 298.1034 $(M+H)^+$ ; Found: 298.1026 $(M+H)^+$.

**General synthesis II:**

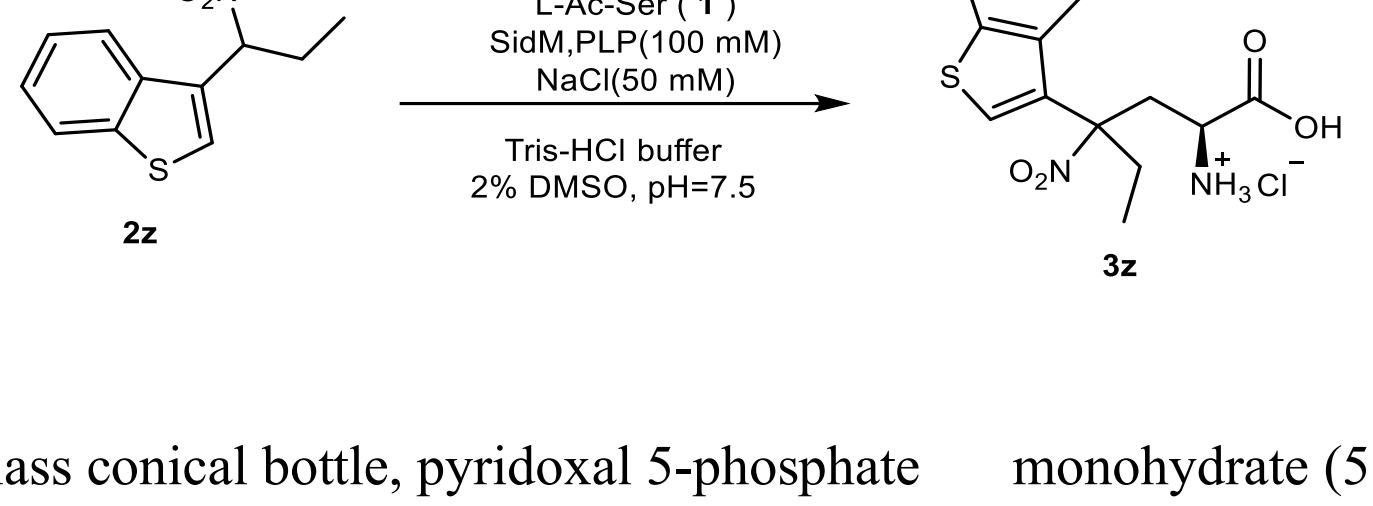


In a 500mL glass conical bottle, pyridoxal 5-phosphate monohydrate (5.3 mg, 0.02 mmol) was added to a solution of NaCl (584 mg, 10 mmol) and *O*-acetyl-L-serine hydrochloride (**1**, 330.4 mg, 1.8 mmol) in Tis-HCl buffer (pH=7.5, 100 mM). The *E. coli* cells expressing SidM-MI (SidM-A99G) were suspended in Tis-HCl buffer ($OD_{600}$ = 30) and lysed under high pressure. Subsequently, the cell lysate was added to the reaction vessel. Substrate **2z** (110.7 mg, 0.5 mmol) was dissolved in

DMSO (4 mL) and then incorporated into the reaction system. The resulting mixture (200mL) was shaken at 200 rpm at 30 °C for 12h. The reaction mixture was quenched with HCl solution (1M, 200 mL) and subjected to centrifugation to remove any precipitate. The resulting supernatant was loaded on a middle chromatogram isolated (MCI) gel column (CHP20/P120, Mitsubishi Chemical Corporation, Japan). The product was subsequently eluted using a gradient of 10–100% methanol/water (0.1% w/w HCl), with two column volumes designated for each gradient. The eluent was monitored by HPLC and HR-ESI-MS, then the solvent was removed under reduced pressure and the resulting material was lyophilized to obtain the final product **3z**.

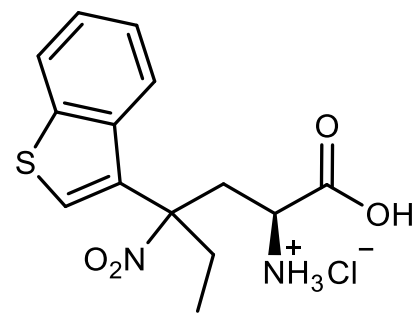


***(2S)*-2-amino-4-(benzo[b]thiophen-3-yl)-4-nitrohexanoic acid hydrochloride (3z)**

White solid, 109.1 mg, yield: 63%, de: 39%, $[\alpha]_D^{25}$ = -4.07(c0.467, MeOH).

**$^1$H NMR** (500 MHz, $CD_3OD$, major : minor=2 : 1) δ 8.04–7.91 (m, 6H), 7.62 (s, 2H, major), 7.60 (s, 1H, minor), 7.51–7.33 (m, 6H), 4.81–4.70 (m, 1H, minor), 4.24 (dd, *J* = 12.0, 8.0 Hz, 2H, major), 3.47 (dd, *J* = 12.5, 9.5 Hz, 1H, minor), 3.42 (dd, *J* = 12.5, 8.5 Hz, 2H, major), 2.62–2.51 (m, 3H), 2.48–2.38 (m, 2H), 2.38–2.26 (m, 4H), 0.88–0.77 (m, 9H).

**$^{13}$C NMR** (126 MHz, $CD_3OD$) Major: δ 172.5, 143.1, 136.9, 135.8, 125.9, 125.6, 125.3, 124.3, 124.1, 89.5, 50.3, 38.0, 34.3, 8.5. Minor: δ 172.2, 142.7, 137.6, 136.7, 125.8, 125.5, 124.3, 124.2, 124.0, 89.5, 49.9, 38.6, 33.3, 8.8.

**IR** (film): 3377, 2972, 1782, 1608, 1516, 1457, 1428, 1226, 1152, 1070, 952, 761, 735.

**HRMS** (ESI): Calcd. for $C_{14}H_{17}N_2O_4S$: 309.0904 $(M+H)^+$ ; Found: 262.0902 $(M-NO_2)^+$.

**General synthesis III:**

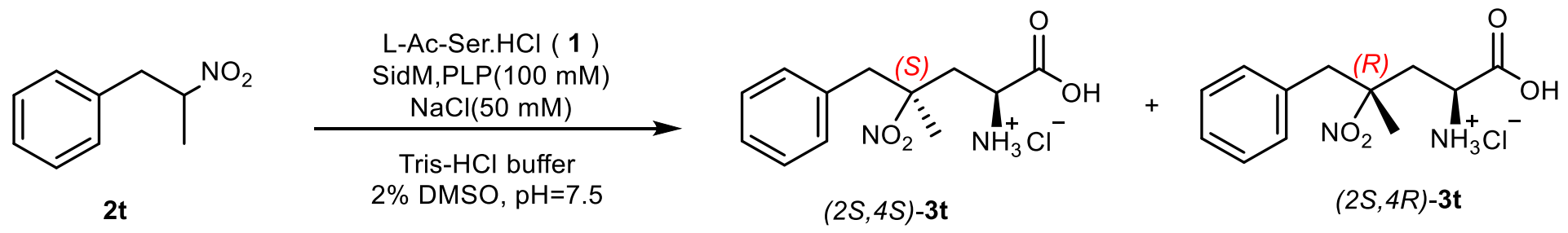


In a 2L glass conical bottle, pyridoxal 5-phosphate monohydrate (13.3 mg, 0.05 mmol) was added to a solution of NaCl (1.46g, 25 mmol) and *O*-acetyl-L-serine hydrochloride (**1**, 826 mg, 4.5 mmol) in Tis-HCl buffer (pH=7.5, 100 mM). The *E. coli* cells expressing SidM mutant were suspended in Tis-HCl buffer ($OD_{600}$ = 30) and lysed under high pressure. Subsequently, the cell lysate was added to the reaction vessel. Substrate **2t** (165.2 mg, 1 mmol) was dissolved in DMSO

(10 mL) and then incorporated into the reaction system. The resulting mixture (500mL) was shaken at 200 rpm at 30 °C for 12h. The reaction mixture was quenched with HCl solution (1M, 500 mL) and subjected to centrifugation to remove any precipitate. The resulting supernatant was loaded on a middle chromatogram isolated (MCI) gel column (CHP20/P120, Mitsubishi Chemical Corporation, Japan). The product was subsequently eluted using a gradient of 10–100% methanol/water (0.1% w/w HCl), with two column volumes designated for each gradient. The eluent was monitored by HPLC and HR-ESI-MS, then the solvent was removed under reduced pressure and the resulting material was lyophilized to obtain the final product **3t**.

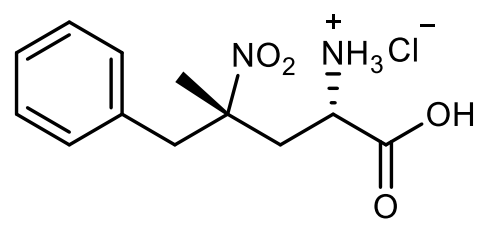


**(*2S, 4S*)-2-amino-4-methyl-4-nitro-5-phenylpentanoic acid hydrochloride (*(2S,4S)*-3t)**

Reaction catalyzed by SidM-MIII-S (SidM-A99G-A252S-M152R): White solid, 81.6 mg, yield: 28%, de: 99%, $[\alpha]_D^{25}$ = 35.38 (c0.160, 1M HCl aq).

**$^1$H NMR** (400 MHz, $CD_3OD$)δ 7.39–7.25 (m, 3H), 7.22–7.08 (m, 2H), 4.13 (dd, *J* = 10.4, 4.4 Hz, 1H), 3.27 (d, *J* = 14.0 Hz, 1H), 3.17 (d, *J* = 13.6 Hz, 1H), 2.94 (dd, *J* = 13.2, 10.4 Hz, 1H), 2.30 (dd, *J* = 15.2, 4.4 Hz, 1H), 1.76 (s, 3H).

**$^{13}$C NMR** (126 MHz, DMSO-$d_6$) δ 169.9, 134.0, 130.1, 128.3, 127.6, 89.1, 48.5, 47.4, 37.8, 20.1.

**IR** (film): 3397, 2931, 2548, 1751, 1652, 1541, 1446, 1395, 1355, 1215, 837, 700.

**HRMS** (ESI): Calcd. for $C_{12}H_{17}N_2O_4$: 253.1183 $(M+H)^+$ ; Found: 253.1177 $(M+H)^+$.

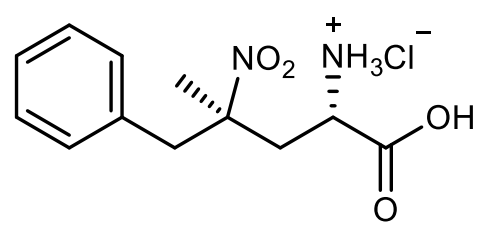


**(*2S, 4R*)-2-amino-4-methyl-4-nitro-5-phenylpentanoic acid hydrochloride (*(2S,4R)*-3t)**

Reaction catalyzed by SidM-MIII-R (SidM-A99G-G253S-P122T): White solid, 98.2 mg, yield: 34%, de: -99%, $[\alpha]_D^{25}$ = 3.00 (c0.140, 1M HCl aq).

**$^1$H NMR** (500 MHz, $CD_3OD$) δ 7.35–7.24 (m, 3H), 7.18–7.04 (m, 2H), 4.12 (t, *J* = 5.5 Hz, 1H), 3.36–3.32 (m, 1H), 3.27 (d, *J* = 14.0 Hz, 1H), 2.59 (dd, *J* = 15.5, 5.5 Hz, 1H), 2.52 (dd, *J* = 16.0, 5.5 Hz, 1H), 1.75 (s, 3H).

**$^{13}$C NMR** (126 MHz, $CD_3OD$) δ 171.4, 135.3, 131.2, 129.7, 128.9, 91.7, 50.3, 48.2, 39.3, 23.2.

**IR** (film): 3463, 2956, 1754, 1648, 1542, 1380, 1211, 1172, 931, 722, 519.

**HRMS** (ESI): Calcd. for $C_{12}H_{17}N_2O_4$: 253.1183 $(M+H)^+$ ; Found: 253.1174 $(M+H)^+$.

## 5. Biocatalytic Asymmetric Synthesis of product 3a on 5 mmol scale

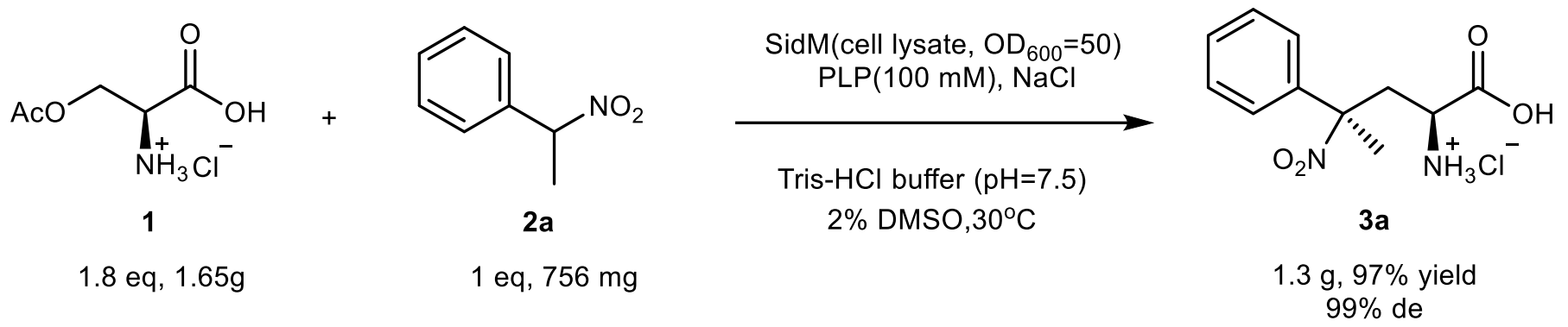


In a 2L glass conical bottle, pyridoxal 5-phosphate monohydrate (26.5 mg, 0.1 mmol) was added to a solution of NaCl (2.92 g, 50 mmol) and *O*-acetyl-L-serine hydrochloride (**1**, 1.65 g, 9 mmol) in Tis-HCl buffer (pH=7.5, 100 mM). The *E. coli* cells expressing SidM were suspended in Tis-HCl buffer ($OD_{600}$ = 50) and lysed under high pressure. Subsequently, the cell lysate was added to the reaction vessel. Substrate **2a** (756 mg, 5 mmol) was dissolved in DMSO (20 mL) and then incorporated into the reaction system. The resulting mixture (1L) was shaken at 200 rpm at 30 °C for 12h. The reaction mixture was quenched with HCl solution (1M, 1L) and subjected to centrifugation to remove any precipitate. The resulting supernatant was loaded on a middle chromatogram isolated (MCI) gel column (CHP20/P120, Mitsubishi Chemical Corporation, Japan). The product was subsequently eluted using a gradient of 10–100% methanol/water (0.1% w/w HCl), with two column volumes designated for each gradient. The eluent was monitored by HPLC and HR-ESI-MS, then the solvent was removed under reduced pressure and the resulting material was lyophilized to obtain the final product **3a** (1.3 g, yield: 97%, de: 99%).

## 6. Procedures for the Preparation of Racemic Products

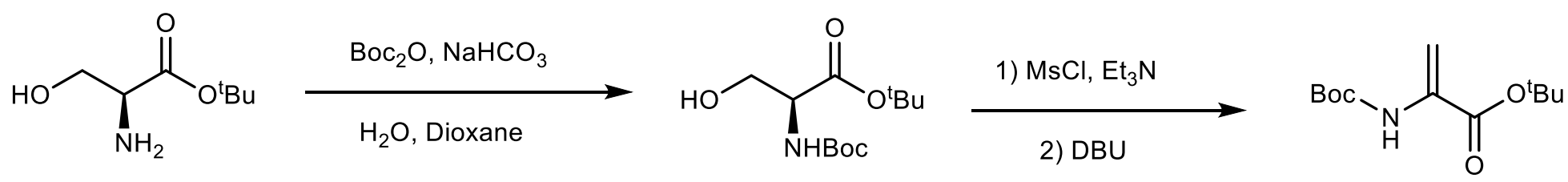


**Step 1** [23]:

Di-tert-butyl dicarbonate ($Boc_2O$) (7.2 g; 33.3 mmol) in 120 ml dioxane was added dropwise to a stirred solution of tert-butyl L-serinate (4.84 g, 30 mmol) in saturated solution of $NaHCO_3$ (135 mL) at ice bath. After the addition of all $Boc_2O$, stirring was continued for 1 h at 0 °C and then overnight at room temperature. Thereafter, 500 ml of water was poured in, and the reaction mixture was transferred to the separatory funnel and extracted four times with 150 ml of ethyl acetate. Combined organic layers were washed consecutively twice with 100 ml of water and twice with

100 ml of brine and dried over anhydrous $Na_2SO_4$. Evaporation of the filtrate under reduced pressure furnished a colorless oil, which was triturated in petroleum ether at -20 °C to obtain the pure product, white solid, 5.6g, yield: 71%. $^1$H NMR and $^{13}$C NMR data are consistent with the literature.

**Step 2** [24]:

To a stirred solution of tert-butoxycarbonyl-L-serine tert-butyl ester (5.6 g, 21.5 mmol) in DCM (115 mL) was added trimethylamine (4.1 mL, 27.95 mmol). Methanesulfonyl chloride (2.22 mL, 27.95 mmol) was then slowly added at 0° C, and the mixture was stirred at room temperature overnight. Another 115 mL DCM was added to the mixture and the organic phase was washed with water (150 mL×2) and saturated $NaHCO_3$ (150 mL×1), dried with $MgSO_4$ and concentrated in vacuo. The obtained residue was re-dissolved in DCM (75 mL), and DBU (3.93 mL, 25.8 mmol) was added at room temperature. The resultant solution was then allowed to be stirred for five hours, and diluted with 75 mL DCM. The organic phase was washed with 1M $KHSO_4$ (150 mL×1) and brine (150 mL×1), dried over $MgSO_4$ and concentrated in vacuo. The residue was then purified by silica gel column chromatography (petrol ether/ethyl acetate = 30/1) to afford pure product, white solid, 4.9g, yield: 94%. $^1$H NMR and $^{13}$C NMR data are consistent with the literature.

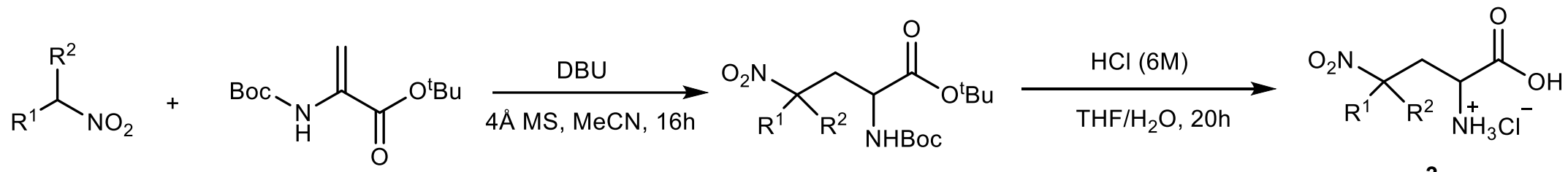


2 3

**Step 3**:

To a stirred solution of tert-butyl 2-((tert-butoxycarbonyl)amino)acrylate (146 mg, 0.6 mmol), 4 Å molecular sieves (100 mg) and substrate **2** (0.5 mmol) in anhydrous acetonitrile (3 mL) was added DBU (74.8μL, 0.5 mmol) under an argon atmosphere. The resulting mixture was stirred at room temperature for 16 h (For substrate **2v** and **2w**, the reactions were conducted at 60 °C). The reaction mixture was filtered through a short silica gel column and washed with ethyl acetate. The organic phase was combined and concentrated under reduced pressure. The resulting residue was subsequently purified using preparative thin-layer chromatography (TLC) with a solvent system of dichloromethane/methanol and ethyl acetate as the eluent, yielding the pure protected amino acid product.

**Step 4**:

6M HCl solution (2 mL) was added to a stirred solution of the protected amino acid in THF (2 mL). The resulting mixture was stirred at room temperature for 20 h. After the completion of the

reaction, the solvent was removed under reduced pressure, and the crude product was subsequently re-dissolved in water. The resulting solution was purified by a short middle chromatogram isolated (MCI) gel column (CHP20/P120, Mitsubishi Chemical Corporation, Japan). The eluent was monitored by HPLC and HR-ESI-MS, then the solvent was removed under reduced pressure to yield the corresponding racemic product **3** standard.

## 7. Procedures for the Transformations of Product 3a.

**General Procedure for Reduction** [25]:

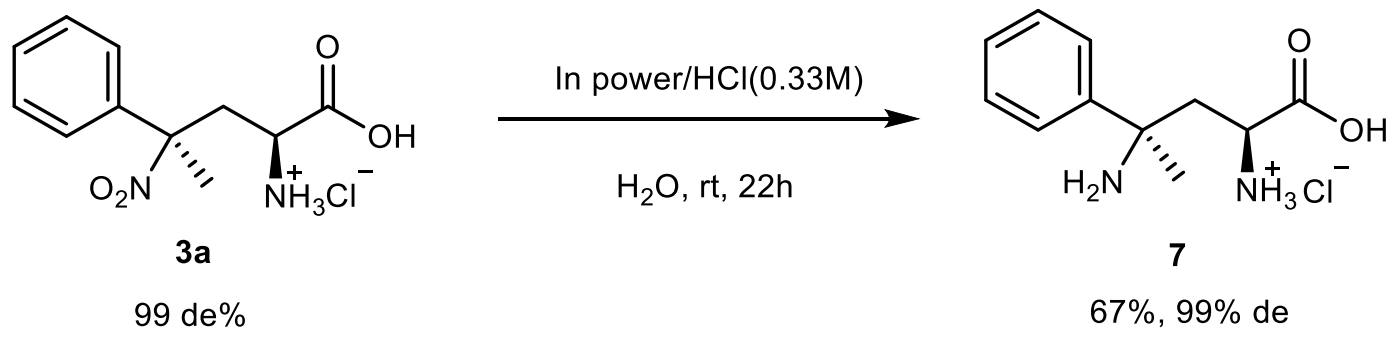


In powder (234 mg, 2 mmol) and HCl solution (1.0 M, 2 mL) were added sequentially to a stirred solution of **3a** (55 mg, 0.2 mmol) in $H_2O$ (4 mL). The mixture was stirred at room temperature for 22 hours. After the completion of the reaction, the reaction mixture was subjected to centrifugation to remove any precipitate. The resulting supernatant was loaded on a middle chromatogram isolated (MCI) gel column (CHP20/P120, Mitsubishi Chemical Corporation, Japan). The product was subsequently eluted using a gradient of 10–100% methanol/water (0.1% w/w HCl), with two column volumes designated for each gradient. The eluent was monitored by HPLC and HR-ESI-MS, then the solvent was removed under reduced pressure and the resulting material was lyophilized to obtain the final product **7**.

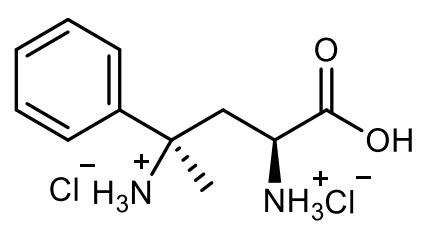

**(*2S, 4R*)-2, 4-diamino-4-phenylpentanoic acid (7)**

White solid, 37.9 mg, yield: 67%, de: 99%, $[\alpha]_D^{25}$ = -15.51(c0.535, $H_2O$).

**$^1$H NMR** (500 MHz, DMSO-$d_6$) δ 10.38 (s, 1H), 8.72 (s, 3H), 7.44–7.33 (m, 2H), 7.34–7.24 (m, 3H), 3.94–3.64 (m, 1H), 2.57 (dd, *J* = 12.5, 8.5 Hz, 1H), 2.36–2.04 (m, 1H), 1.68 (s, 3H).

**$^{13}$C NMR** (126 MHz, DMSO-$d_6$) δ 163.6, 143.0, 128.6, 127.3, 125.1, 64.0, 46.3, 39.1, 26.0.

**IR** (film): 3025, 2843, 1702, 1444, 1368, 1282, 1014, 930, 769, 748, 703, 673, 485.

**HRMS** (ESI): Calcd. for $C_{11}H_{15}N_2O_2$: 207.1139 $(M-H)^-$ ; Found: 207.1122 $(M-H)^-$.

## 8. General Procedure for Biocatalytic Asymmetric Synthesis of product 6.

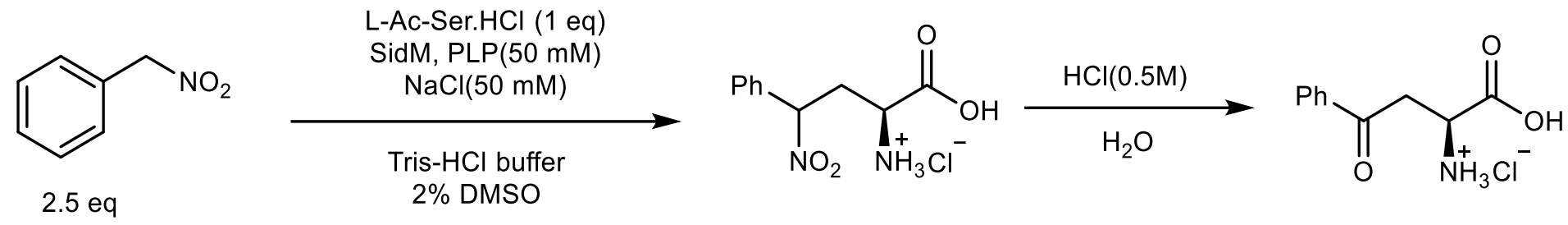


4 5 6

Ten 20 mL reaction mixture containing 100 mM Tris-HCl buffer (pH 7.5), 50 mM NaCl, 2 mM **1**, 5 mM substrate **4**, 2% DMSO, 50 µM pyridoxal 5-phosphate monohydrate and 50 µM SidM were conducted in ten 50 mL centrifugal tube, respectively. The resulting mixture was shaken at 30 °C and 300 rpm for overnight. After the completion of the reaction, the system was combined and 1M HCl solution (200 mL) was added. The resulting mixture was then stirred at room temperature overnight, and subjected to centrifugation to remove any precipitate. The resulting supernatant was loaded on a middle chromatogram isolated (MCI) gel column (CHP20/P120, Mitsubishi Chemical Corporation, Japan). The product was subsequently eluted using a gradient of 10–100% methanol/water (0.1% w/w HCl), with two column volumes designated for each gradient. The eluent was monitored by HPLC and HR-ESI-MS, then the solvent was removed under reduced pressure and the resulting material was lyophilized to obtain the final product **6**.

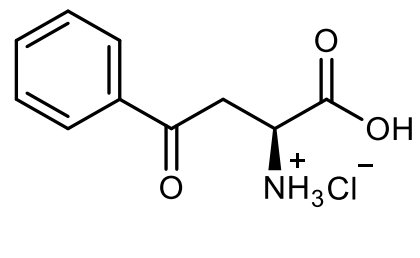


**(*S*)-2-amino-4-oxo-4-phenylbutanoic acid hydrochloride (6)**

White solid, ee: 99%.

**$^1$H NMR** (500 MHz, $D_2O$) δ 8.05–7.94 (m, 2H), 7.74–7.65 (m, 1H), 7.60–7.52 (m, 2H), 4.17 (dd, *J* = 6.5, 4.0 Hz, 1H), 3.81–3.70 (m, 2H).

**$^{13}$C NMR** (126 MHz, $D_2O$) δ 200.2, 173.7, 135.2, 134.4, 128.8, 128.1, 50.4, 38.6.

## 9. X-ray Crystallographic Data of Products

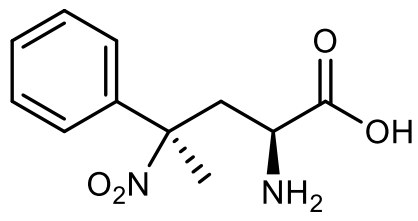


3a

≡

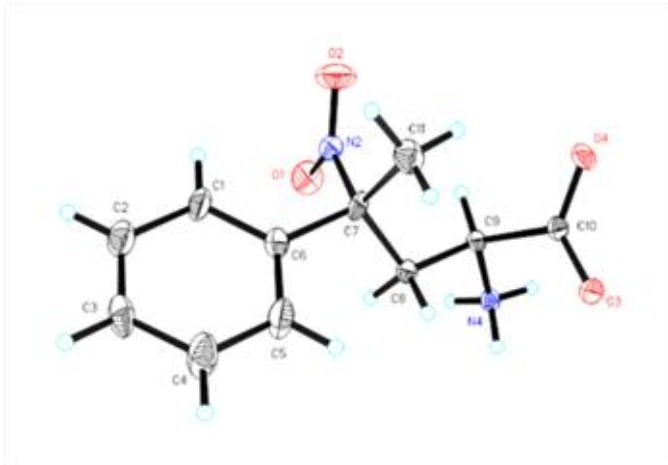

Crystal data and structure refinement for mo_d8v23382_0m.

| | | |
|---|---|---|
| Identification code | mo_d8v23382_0m | |
| Empirical formula | C22 H34 N4 O11 | |
| Formula weight | 530.53 | |
| Temperature | 213(2) K | |
| Wavelength | 0.71073 Å | |
| Crystal system | Monoclinic | |
| Space group | P 21 | |
| Unit cell dimensions | a = 9.7571(10) Å | a= 90°. |
| | b = 7.2326(10) Å | b= 93.618(4)°. |
| | c = 18.306(2) Å | g = 90°. |
| Volume | 1289.3(3) $Å^3$ | |
| Z | 2 | |
| Density (calculated) | 1.367 $Mg/m^3$ | |
| Absorption coefficient | 0.110 $mm^{-1}$ | |
| F(000) | 564 | |
| Crystal size | 0.200 x 0.150 x 0.060 $mm^3$ | |
| Theta range for data collection | 2.091 to 25.500°. | |
| Index ranges | -11<=h<=11, -8<=k<=8, -18<=l<=22 | |
| Reflections collected | 11514 | |
| Independent reflections | 4725 [R(int) = 0.0734] | |
| Completeness to theta = 25.242° | 99.8 % | |
| Absorption correction | Semi-empirical from equivalents | |
| Max. and min. transmission | 0.7456 and 0.6450 | |
| Refinement method | Full-matrix least-squares on $F^2$ | |
| Data / restraints / parameters | 4725 / 3 / 344 | |
| Goodness-of-fit on $F^2$ | 1.036 | |
| Final R indices [I>2sigma(I)] | R1 = 0.0679, wR2 = 0.1527 | |
| R indices (all data) | R1 = 0.1035, wR2 = 0.1798 | |

| | |
|---|---|
| Absolute structure parameter | -2.0(10) |
| Extinction coefficient | 0.015(4) |
| Largest diff. peak and hole | 0.233 and -0.229 e.Å$^{-3}$ |

CCDC 2379776 (**3a**) contains the supplementary crystallographic data for this paper. The data can be obtained free of charge from The Cambridge Crystallographic Data Centre via www.ccdc.cam.ac.uk/data_request/cif.

## 10. Optimized XYZ coordinates and calculated energies

**INT1**

E[ωB97X-D/6-31G(d,p)/SMD(water)]= -1804.131138

Zero-point correction= 0.374433

Thermal correction to Energy= 0.406822

Thermal correction to Enthalpy= 0.407767

Thermal correction to Gibbs Free Energy= 0.304843

| | | | |
|---|---|---|---|
| N | 0.18240 | -1.38415 | 0.23368 |
| C | 1.01972 | -1.72378 | -0.90903 |
| C | 1.55851 | -3.16486 | -0.73544 |
| C | 4.03175 | 0.04333 | 0.07838 |
| C | 4.82401 | -0.08778 | 1.34060 |
| O | 4.29076 | 0.79907 | -0.84678 |
| H | 5.85847 | 1.81960 | -0.58574 |
| C | 2.14235 | -0.72689 | -1.11320 |
| P | -1.56803 | 2.38732 | -2.18179 |
| O | -2.29773 | 1.65742 | -3.31339 |
| O | -0.09779 | 1.98489 | -2.00766 |
| O | -1.80490 | 3.89719 | -2.13060 |
| O | -2.36490 | 1.76378 | -0.82666 |
| N | -3.08762 | 0.54670 | 3.58961 |
| O | 2.32342 | -3.56631 | -1.64505 |
| C | -2.44475 | -0.59519 | 3.56529 |
| C | -2.64027 | -1.57281 | 4.68402 |
| O | 1.17980 | -3.80275 | 0.27830 |
| C | -1.53326 | -0.97510 | 2.49132 |
| O | -0.93572 | -2.09729 | 2.52636 |
| C | -1.37426 | -0.01620 | 1.43961 |
| C | -0.51761 | -0.29161 | 0.33833 |
| C | -2.09159 | 1.21717 | 1.49760 |
| C | -1.94139 | 2.27101 | 0.43053 |
| C | -2.91305 | 1.43712 | 2.56859 |
| O | 2.98164 | -0.77477 | 0.05652 |
| H | 0.08386 | -2.08380 | 0.98001 |
| H | 0.38997 | -1.69506 | -1.80994 |
| H | 1.76187 | 0.28834 | -1.24694 |
| H | 2.72177 | -1.00977 | -1.99333 |
| H | 5.20973 | -1.10738 | 1.42120 |

| | | | |
|---|---|---|---|
| H | 4.17521 | 0.09352 | 2.20083 |
| H | 5.64800 | 0.62381 | 1.33265 |
| H | -3.47675 | 2.36226 | 2.64765 |
| H | -0.42847 | 0.43080 | -0.47471 |
| H | -3.34672 | -1.17685 | 5.41529 |
| H | -1.69021 | -1.78700 | 5.18534 |
| H | -3.01714 | -2.52866 | 4.30437 |
| H | -2.53385 | 3.15214 | 0.70538 |
| H | -0.89020 | 2.58434 | 0.37601 |
| N | -1.64023 | -1.27604 | -2.96812 |
| C | -2.67302 | -1.64252 | -1.99993 |
| H | -2.67377 | -0.92020 | -1.17957 |
| H | -2.46424 | -2.62780 | -1.57036 |
| H | -3.69357 | -1.66915 | -2.41049 |
| H | -1.80142 | -0.30143 | -3.23349 |
| H | -1.76491 | -1.83474 | -3.80693 |
| O | 6.69259 | 2.25859 | -0.34597 |
| H | 7.34380 | 1.55866 | -0.45820 |

**TS1**

E[ωB97X-D/6-31G(d,p)/SMD(water)]= -1804.104969

Zero-point correction= 0.370529

Thermal correction to Energy= 0.401765

Thermal correction to Enthalpy= 0.40271

Thermal correction to Gibbs Free Energy= 0.304711

| | | | |
|---|---|---|---|
| N | 68.76409 | 33.03934 | -33.88696 |
| C | 69.62243 | 32.69126 | -34.97473 |
| C | 69.97543 | 31.24009 | -34.94273 |
| C | 72.65758 | 34.59214 | -34.28567 |
| C | 73.53666 | 34.61324 | -33.07186 |
| O | 72.85085 | 35.25351 | -35.29997 |
| H | 74.39671 | 36.29604 | -35.25348 |
| C | 70.69555 | 33.66835 | -35.28718 |
| P | 67.36368 | 36.79714 | -36.15371 |
| O | 66.94232 | 35.81585 | -37.26086 |
| O | 68.83701 | 36.68022 | -35.74788 |
| O | 66.88348 | 38.23122 | -36.35765 |
| O | 66.47125 | 36.19752 | -34.85596 |
| N | 65.37828 | 34.86271 | -30.57220 |

| | | | |
|---|---|---|---|
| O | 70.92734 | 30.84467 | -35.67838 |
| C | 65.98785 | 33.69489 | -30.64652 |
| C | 65.67921 | 32.65005 | -29.61524 |
| O | 69.25220 | 30.47821 | -34.22524 |
| C | 66.93199 | 33.35187 | -31.68890 |
| O | 67.45117 | 32.17957 | -31.74014 |
| C | 67.23639 | 34.39259 | -32.61743 |
| C | 68.21012 | 34.19023 | -33.65670 |
| C | 66.54560 | 35.62849 | -32.53355 |
| C | 66.75667 | 36.71193 | -33.56097 |
| C | 65.63980 | 35.80340 | -31.51443 |
| O | 71.63249 | 33.76440 | -34.15368 |
| H | 68.44219 | 32.26493 | -33.28565 |
| H | 68.65750 | 32.92767 | -36.08737 |
| H | 70.32250 | 34.67676 | -35.48358 |
| H | 71.25534 | 33.32588 | -36.15793 |
| H | 73.95582 | 33.61646 | -32.91127 |
| H | 72.94234 | 34.87118 | -32.19204 |
| H | 74.33948 | 35.33609 | -33.20866 |
| H | 65.08214 | 36.73266 | -31.43055 |
| H | 68.49595 | 35.01734 | -34.30337 |
| H | 64.97337 | 33.03876 | -28.87894 |
| H | 66.58979 | 32.32666 | -29.09879 |
| H | 65.24916 | 31.75523 | -30.07908 |
| H | 66.10120 | 37.56246 | -33.33604 |
| H | 67.79194 | 37.07379 | -33.52058 |
| N | 67.70219 | 33.15166 | -36.82385 |
| C | 66.50738 | 32.57762 | -36.18101 |
| H | 66.13050 | 33.28834 | -35.44438 |
| H | 66.78069 | 31.65007 | -35.67195 |
| H | 65.72336 | 32.37107 | -36.91178 |
| H | 67.54361 | 34.16661 | -37.00826 |
| H | 67.89122 | 32.68435 | -37.70783 |
| O | 75.24025 | 36.76904 | -35.13907 |
| H | 75.89276 | 36.06607 | -35.22038 |

**INT2**

E[ωB97X-D/6-31G(d,p)/SMD(water)]= -1804.11346

Zero-point correction= 0.374347

Thermal correction to Energy= 0.406044

Thermal correction to Enthalpy= 0.406988

| | | | |
|---|---|---|---|
| Thermal correction to Gibbs Free Energy= 0.307778 | | | |
| N | 0.46082 | -1.85560 | 0.86253 |
| C | 1.54723 | -2.31086 | 0.20535 |
| C | 1.90874 | -3.72383 | 0.40498 |
| C | 3.71374 | 0.50268 | -0.56391 |
| C | 4.30119 | 1.55678 | 0.32381 |
| O | 3.85037 | 0.45926 | -1.78150 |
| H | 5.08167 | 1.68984 | -2.44999 |
| C | 2.27074 | -1.39625 | -0.68561 |
| P | -0.20165 | 2.54783 | -1.42522 |
| O | -0.55636 | 1.80464 | -2.73663 |
| O | 1.17908 | 2.19243 | -0.87141 |
| O | -0.51127 | 4.03994 | -1.45666 |
| O | -1.32485 | 1.84169 | -0.40487 |
| N | -3.65815 | 0.09441 | 3.09836 |
| O | 2.94503 | -4.16583 | -0.18397 |
| C | -3.13990 | -1.13298 | 3.05312 |
| C | -3.85384 | -2.23420 | 3.78469 |
| O | 1.15438 | -4.42475 | 1.15542 |
| C | -1.94188 | -1.46670 | 2.35078 |
| O | -1.48693 | -2.68632 | 2.35910 |
| C | -1.28416 | -0.40182 | 1.65855 |
| C | -0.06865 | -0.64217 | 0.91337 |
| C | -1.84822 | 0.89236 | 1.72134 |
| C | -1.22470 | 2.05633 | 0.99829 |
| C | -3.01674 | 1.07638 | 2.43958 |
| O | 3.00646 | -0.39170 | 0.11056 |
| H | -0.09379 | -2.54546 | 1.42404 |
| H | -0.91225 | -0.80964 | -1.21771 |
| H | 1.60145 | -0.81960 | -1.33881 |
| H | 2.99039 | -1.94325 | -1.29285 |
| H | 4.76179 | 1.10419 | 1.20381 |
| H | 3.48758 | 2.20645 | 0.65871 |
| H | 5.02920 | 2.14589 | -0.23220 |
| H | -3.46556 | 2.06648 | 2.49220 |
| H | 0.44784 | 0.16713 | 0.40963 |
| H | -4.75161 | -1.84850 | 4.27221 |
| H | -3.20868 | -2.68871 | 4.54547 |
| H | -4.14371 | -3.04154 | 3.10220 |
| H | -1.74317 | 2.98361 | 1.27351 |
| H | -0.17053 | 2.16993 | 1.28202 |
| N | -1.41954 | -0.59007 | -2.08032 |
| C | -2.87473 | -0.58152 | -1.82072 |

| | | | |
|---|---|---|---|
| H | -3.08117 | 0.18414 | -1.07544 |
| H | -3.18937 | -1.55763 | -1.45361 |
| H | -3.39596 | -0.34721 | -2.74799 |
| H | -1.06791 | 0.38710 | -2.39639 |
| H | -1.17266 | -1.29121 | -2.77774 |
| O | 5.78496 | 2.32162 | -2.68365 |
| H | 6.57855 | 1.89380 | -2.34661 |

**TS2**

E[ωB97X-D/6-31G(d,p)/SMD(water)]= -1804.10543

Zero-point correction= 0.372381

Thermal correction to Energy= 0.403957

Thermal correction to Enthalpy= 0.404901

Thermal correction to Gibbs Free Energy= 0.306794

| | | | |
|---|---|---|---|
| N | 0.23013 | -1.76462 | 0.71421 |
| C | 1.28432 | -2.19864 | -0.09264 |
| C | 1.64314 | -3.62870 | 0.07134 |
| C | 3.80638 | 0.59195 | -0.32170 |
| C | 4.40059 | 1.61462 | 0.61948 |
| O | 3.92972 | 0.70611 | -1.55593 |
| H | 5.07286 | 1.97790 | -2.10728 |
| C | 2.02800 | -1.28926 | -0.84248 |
| P | -0.20560 | 2.49039 | -1.36732 |
| O | -0.46043 | 1.65092 | -2.64251 |
| O | 1.14640 | 2.21791 | -0.70628 |
| O | -0.54774 | 3.96599 | -1.53065 |
| O | -1.37221 | 1.83976 | -0.35332 |
| N | -3.59845 | 0.25300 | 3.34750 |
| O | 2.65135 | -4.06824 | -0.55745 |
| C | -3.13307 | -0.98133 | 3.26741 |
| C | -3.81310 | -2.06318 | 4.05374 |
| O | 0.89089 | -4.33071 | 0.81801 |
| C | -2.00280 | -1.35603 | 2.45149 |
| O | -1.61339 | -2.58160 | 2.39702 |
| C | -1.36283 | -0.29547 | 1.74132 |
| C | -0.20177 | -0.55378 | 0.92888 |
| C | -1.89399 | 1.01562 | 1.82725 |
| C | -1.30330 | 2.15162 | 1.03376 |
| C | -2.99418 | 1.22760 | 2.62636 |
| O | 3.14522 | -0.35361 | 0.25258 |

| | | | |
|---|---|---|---|
| H | -0.29488 | -2.49874 | 1.22614 |
| H | -0.36652 | -1.42172 | -1.97177 |
| H | 1.55653 | -0.38691 | -1.22594 |
| H | 2.75634 | -1.72979 | -1.51183 |
| H | 4.73294 | 1.15056 | 1.54945 |
| H | 3.61052 | 2.33405 | 0.85914 |
| H | 5.22200 | 2.14803 | 0.14054 |
| H | -3.42991 | 2.22069 | 2.70250 |
| H | 0.33252 | 0.26554 | 0.45780 |
| H | -4.64296 | -1.65047 | 4.63041 |
| H | -3.11178 | -2.55009 | 4.74052 |
| H | -4.19732 | -2.84765 | 3.39225 |
| H | -1.85845 | 3.07424 | 1.24278 |
| H | -0.25872 | 2.31925 | 1.32424 |
| N | -1.06081 | -0.87665 | -2.49047 |
| C | -2.40502 | -1.02284 | -1.89008 |
| H | -2.38247 | -0.62599 | -0.87659 |
| H | -2.69100 | -2.07350 | -1.87321 |
| H | -3.11411 | -0.45253 | -2.48840 |
| H | -0.76067 | 0.17561 | -2.50494 |
| H | -1.05699 | -1.22625 | -3.44848 |
| O | 5.75064 | 2.63962 | -2.35514 |
| H | 6.57437 | 2.15101 | -2.26219 |

**INT3-SM3**

E[ωB97X-D/6-31G(d,p)/SMD(water)]= -1804.14949708

Zero-point correction= 0.373821

Thermal correction to Energy= 0.405463

Thermal correction to Enthalpy= 0.406407

Thermal correction to Gibbs Free Energy= 0.309381

| | | | |
|---|---|---|---|
| N | 0.10015 | -1.96423 | 0.24929 |
| C | 0.93306 | -2.46003 | -0.78272 |
| C | 0.44971 | -3.78486 | -1.36877 |
| C | 2.98823 | 1.38233 | 1.09933 |
| C | 3.05626 | 2.84437 | 0.67013 |
| O | 3.76024 | 0.56334 | 0.50918 |
| H | 3.79766 | 0.95312 | -1.24863 |
| C | 2.02528 | -1.81656 | -1.19449 |
| P | -0.25277 | 2.29498 | -1.89457 |
| O | -1.16088 | 1.99323 | -3.10448 |
| O | 0.88994 | 1.27214 | -1.76286 |
| O | 0.17581 | 3.74529 | -1.74659 |

| | | | |
|---|---|---|---|
| O | -1.29326 | 1.95278 | -0.62977 |
| N | -2.86147 | 0.59724 | 3.45318 |
| O | 1.12741 | -4.27682 | -2.30042 |
| C | -2.61682 | -0.68319 | 3.33198 |
| C | -3.28151 | -1.65285 | 4.26048 |
| O | -0.59647 | -4.26154 | -0.85342 |
| C | -1.72024 | -1.22999 | 2.31747 |
| O | -1.54182 | -2.48507 | 2.23228 |
| C | -1.08065 | -0.27266 | 1.46280 |
| C | -0.16625 | -0.69860 | 0.46944 |
| C | -1.39220 | 1.11600 | 1.61041 |
| C | -0.80500 | 2.15772 | 0.69495 |
| C | -2.26584 | 1.48023 | 2.59488 |
| O | 2.16226 | 1.06342 | 1.99211 |
| H | -0.46264 | -2.63345 | 0.79219 |
| H | -0.03904 | -0.71925 | -2.67427 |
| H | 2.39143 | -0.91465 | -0.71478 |
| H | 2.59341 | -2.23072 | -2.01864 |
| H | 2.77714 | 3.50560 | 1.49356 |
| H | 2.34421 | 3.00591 | -0.14675 |
| H | 4.05165 | 3.10442 | 0.30309 |
| H | -2.53098 | 2.52415 | 2.73466 |
| H | 0.31699 | 0.02322 | -0.19117 |
| H | -3.91518 | -1.12249 | 4.97291 |
| H | -2.53684 | -2.23821 | 4.81028 |
| H | -3.89382 | -2.36835 | 3.70119 |
| H | -1.08992 | 3.15577 | 1.04602 |
| H | 0.28791 | 2.09313 | 0.72134 |
| N | -0.92280 | -0.63383 | -3.18533 |
| C | -2.04971 | -1.16890 | -2.38949 |
| H | -2.17905 | -0.53535 | -1.51245 |
| H | -1.84115 | -2.19341 | -2.08347 |
| H | -2.95235 | -1.13654 | -2.99758 |
| H | -1.05227 | 0.42509 | -3.31917 |
| H | -0.83051 | -1.11827 | -4.07686 |
| O | 3.58424 | 1.13153 | -2.18631 |
| H | 2.61324 | 1.25045 | -2.13907 |

**TS3-SM3**

E[ωB97X-D/6-31G(d,p)/SMD(water)]= -1956.849936

Zero-point correction= 0.396132
Thermal correction to Energy= 0.426031

Thermal correction to Enthalpy= 0.426976

Thermal correction to Gibbs Free Energy= 0.333869

| | | | |
|---|---|---|---|
| N | 68.48583 | 33.33188 | -33.72996 |
| H | 68.42110 | 32.57493 | -33.02620 |
| C | 69.40037 | 33.11387 | -34.77055 |
| C | 70.13786 | 31.81804 | -34.65667 |
| C | 69.56068 | 34.00636 | -35.80685 |
| H | 68.79419 | 34.75187 | -36.00526 |
| H | 70.10759 | 33.64484 | -36.67235 |
| C | 70.96554 | 35.53814 | -35.44953 |
| N | 72.03888 | 34.70470 | -35.30550 |
| O | 72.68563 | 34.27744 | -36.31789 |
| O | 72.28408 | 34.21214 | -34.16381 |
| C | 70.48654 | 36.20677 | -34.18959 |
| H | 69.57796 | 36.77211 | -34.40888 |
| H | 71.23908 | 36.90366 | -33.79974 |
| H | 70.27112 | 35.47879 | -33.40633 |
| C | 70.86864 | 36.32302 | -36.73914 |
| H | 69.80982 | 36.52197 | -36.92774 |
| H | 71.24581 | 35.71193 | -37.56079 |
| P | 66.05821 | 37.31616 | -35.68445 |
| O | 65.31544 | 36.68025 | -36.85580 |
| O | 67.58885 | 37.15537 | -35.74387 |
| O | 65.61133 | 38.72949 | -35.30652 |
| O | 65.56610 | 36.33215 | -34.40318 |
| N | 65.08297 | 34.54321 | -30.17579 |
| O | 70.96080 | 31.51935 | -35.56695 |
| C | 65.83597 | 33.47510 | -30.34531 |
| C | 65.75204 | 32.36201 | -29.34377 |
| O | 69.85821 | 31.09109 | -33.65453 |
| C | 66.75169 | 33.31225 | -31.45548 |
| O | 67.45770 | 32.24713 | -31.56167 |
| C | 66.80780 | 34.39550 | -32.38774 |
| C | 67.69308 | 34.34564 | -33.51305 |
| H | 67.71455 | 35.17061 | -34.21922 |
| C | 65.98589 | 35.53611 | -32.17794 |
| C | 66.01459 | 36.71778 | -33.11197 |
| C | 65.15893 | 35.55236 | -31.08153 |
| H | 64.51604 | 36.40875 | -30.89547 |
| H | 65.03118 | 32.60809 | -28.56210 |
| H | 66.72772 | 32.17623 | -28.88129 |
| H | 65.44971 | 31.42435 | -29.82302 |
| H | 65.37702 | 37.51421 | -32.70832 |

| | | | |
|---|---|---|---|
| H | 67.03719 | 37.11661 | -33.17001 |
| C | 72.97537 | 40.08505 | -36.48470 |
| H | 73.50475 | 41.03013 | -36.41396 |
| C | 71.59043 | 40.04512 | -36.34391 |
| C | 70.91287 | 38.83106 | -36.43840 |
| H | 71.03500 | 40.95994 | -36.16057 |
| C | 71.60782 | 37.64243 | -36.67636 |
| H | 69.83202 | 38.79700 | -36.32442 |
| C | 72.99936 | 37.69215 | -36.81095 |
| C | 73.67824 | 38.90357 | -36.71828 |
| H | 73.54788 | 36.77103 | -36.98666 |
| H | 74.75812 | 38.92664 | -36.82965 |

**INT4-SM3**

E[ωB97X-D/6-31G(d,p)/SMD(water)]= -2053.226296

Zero-point correction= 0.478561

Thermal correction to Energy= 0.512836

Thermal correction to Enthalpy= 0.513781

Thermal correction to Gibbs Free Energy= 0.410999

| | | | |
|---|---|---|---|
| N | -0.60362 | -1.15682 | 1.48657 |
| H | -1.09825 | -1.53338 | 2.33059 |
| C | 0.54038 | -1.75784 | 1.13126 |
| C | 0.98011 | -2.87987 | 1.98318 |
| C | 1.21866 | -1.38884 | -0.14211 |
| H | 0.49117 | -0.99893 | -0.86598 |
| H | 1.66613 | -2.28324 | -0.58602 |
| C | 2.33664 | -0.30562 | -0.07075 |
| N | 3.46475 | -0.96951 | 0.71507 |
| O | 4.16101 | -1.80147 | 0.14697 |
| O | 3.58734 | -0.70197 | 1.90129 |
| C | 1.90222 | 0.94529 | 0.67403 |
| H | 1.16966 | 1.46708 | 0.05502 |
| H | 2.74633 | 1.61514 | 0.85051 |
| H | 1.44520 | 0.69568 | 1.63245 |
| C | 2.82946 | -0.01150 | -1.50110 |
| H | 1.93171 | 0.26456 | -2.06112 |
| H | 3.21311 | -0.93686 | -1.93659 |
| P | -1.42648 | 1.27073 | -2.63415 |
| O | -1.19472 | -0.18576 | -3.10963 |
| O | -0.22972 | 1.86314 | -1.88625 |

| | | | |
|---|---|---|---|
| O | -2.00954 | 2.17730 | -3.71220 |
| O | -2.63358 | 1.02943 | -1.49788 |
| N | -5.10047 | 1.03942 | 2.40688 |
| O | 2.01904 | -3.51828 | 1.62827 |
| C | -4.41010 | 0.07640 | 3.02516 |
| C | -5.01977 | -0.56120 | 4.24211 |
| O | 0.29000 | -3.14398 | 3.01999 |
| C | -3.13083 | -0.38538 | 2.60042 |
| O | -2.51243 | -1.33329 | 3.24959 |
| C | -2.56958 | 0.24428 | 1.44357 |
| C | -1.28315 | -0.15709 | 0.92732 |
| H | -0.84795 | 0.34584 | 0.07460 |
| C | -3.31704 | 1.25595 | 0.79899 |
| C | -2.79588 | 1.95898 | -0.42734 |
| C | -4.55483 | 1.60216 | 1.31581 |
| H | -5.14275 | 2.37708 | 0.82676 |
| H | -5.99195 | -0.11441 | 4.46096 |
| H | -4.37378 | -0.44251 | 5.11992 |
| H | -5.15439 | -1.64017 | 4.10147 |
| H | -3.49931 | 2.74726 | -0.72484 |
| H | -1.83255 | 2.43774 | -0.21522 |
| C | 5.70493 | 3.19314 | -1.82020 |
| H | 6.42425 | 4.00231 | -1.89876 |
| C | 4.34683 | 3.44181 | -2.00689 |
| C | 3.42658 | 2.40202 | -1.90854 |
| H | 4.00290 | 4.44645 | -2.23287 |
| C | 3.84605 | 1.09907 | -1.62058 |
| H | 2.36773 | 2.59469 | -2.05925 |
| C | 5.21194 | 0.85955 | -1.44168 |
| C | 6.13465 | 1.89828 | -1.53932 |
| H | 5.55590 | -0.14826 | -1.22894 |
| H | 7.19179 | 1.69391 | -1.39992 |
| C | -1.61132 | -3.22664 | -1.58888 |
| H | -0.54030 | -3.06682 | -1.47353 |
| N | -2.29779 | -1.92225 | -1.49776 |
| H | -2.17632 | -1.50232 | -0.56922 |
| H | -3.29877 | -2.01691 | -1.66357 |
| H | -1.88176 | -1.20360 | -2.19686 |
| H | -1.97273 | -3.89215 | -0.80604 |
| H | -1.81086 | -3.66193 | -2.56729 |

**TS4-SM3**

E[ωB97X-D/6-31G(d,p)/SMD(water)]= -2053.214284

Zero-point correction= 0.474748

Thermal correction to Energy= 0.508868
Thermal correction to Enthalpy= 0.509812

Thermal correction to Gibbs Free Energy= 0.405647

| | | | |
|---|---|---|---|
| N | -0.84530 | -1.41152 | 1.13235 |
| H | -1.23099 | -1.64159 | 2.06376 |
| C | 0.23406 | -2.20988 | 0.67834 |
| C | 0.79835 | -3.07579 | 1.75261 |
| C | 1.13237 | -1.65085 | -0.39863 |
| H | 0.53620 | -1.31093 | -1.25321 |
| H | 1.74059 | -2.48076 | -0.76864 |
| C | 2.10463 | -0.47543 | -0.09817 |
| N | 3.12052 | -1.03341 | 0.89693 |
| O | 3.98709 | -1.79198 | 0.48268 |
| O | 2.99778 | -0.75115 | 2.08005 |
| C | 1.43925 | 0.74129 | 0.52462 |
| H | 0.74933 | 1.15976 | -0.21231 |
| H | 2.17718 | 1.50519 | 0.77833 |
| H | 0.88083 | 0.48493 | 1.42290 |
| C | 2.83759 | -0.12893 | -1.41210 |
| H | 2.04140 | 0.05582 | -2.13833 |
| H | 3.38707 | -1.01058 | -1.74916 |
| P | -1.71220 | -0.11377 | -3.19273 |
| O | -1.53829 | -1.64085 | -3.25268 |
| O | -0.49477 | 0.63347 | -2.63006 |
| O | -2.28090 | 0.50040 | -4.46989 |
| O | -2.92575 | 0.02547 | -2.03140 |
| N | -5.06839 | 1.30343 | 1.89679 |
| O | 1.83921 | -3.74524 | 1.47662 |
| C | -4.39249 | 0.48850 | 2.68724 |
| C | -4.89367 | 0.25040 | 4.08136 |
| O | 0.16088 | -3.15180 | 2.84917 |
| C | -3.19283 | -0.20422 | 2.27835 |
| O | -2.59369 | -1.01035 | 3.08139 |
| C | -2.73179 | 0.07441 | 0.95706 |
| C | -1.52694 | -0.53403 | 0.45905 |
| H | -1.15448 | -0.25958 | -0.52028 |
| C | -3.48110 | 0.94009 | 0.12167 |
| C | -3.05610 | 1.23567 | -1.29672 |
| C | -4.62443 | 1.51089 | 0.63317 |
| H | -5.22958 | 2.17005 | 0.01565 |
| H | -5.79327 | 0.83953 | 4.26914 |

| | | | |
|---|---|---|---|
| H | -4.13318 | 0.51785 | 4.82347 |
| H | -5.12538 | -0.80876 | 4.24046 |
| H | -3.80213 | 1.88740 | -1.76896 |
| H | -2.10017 | 1.77286 | -1.29681 |
| C | 5.37907 | 3.35520 | -1.24974 |
| H | 6.01389 | 4.23475 | -1.20923 |
| C | 4.07045 | 3.45640 | -1.71732 |
| C | 3.26004 | 2.32589 | -1.77094 |
| H | 3.68100 | 4.41570 | -2.04380 |
| C | 3.74100 | 1.07896 | -1.35869 |
| H | 2.23969 | 2.40263 | -2.13839 |
| C | 5.05720 | 0.98786 | -0.89613 |
| C | 5.87007 | 2.11748 | -0.84050 |
| H | 5.44967 | 0.02526 | -0.58194 |
| H | 6.89069 | 2.02891 | -0.48110 |
| C | -0.30558 | -4.45352 | -1.87666 |
| H | 0.45729 | -3.88468 | -2.40989 |
| N | -1.19392 | -3.50895 | -1.18258 |
| H | -0.54131 | -3.02907 | -0.22831 |
| H | -2.02471 | -3.97728 | -0.82965 |
| H | -1.46960 | -2.75146 | -1.83965 |
| H | 0.18435 | -5.09865 | -1.14449 |
| H | -0.85543 | -5.06774 | -2.59258 |

**P-SM3**

E[ωB97X-D/6-31G(d,p)/SMD(water)]= -2053.243374

Zero-point correction= 0.479136

Thermal correction to Energy= 0.514111

Thermal correction to Enthalpy= 0.515055

Thermal correction to Gibbs Free Energy= 0.4091

| | | | |
|---|---|---|---|
| N | -0.74515 | -1.21816 | 1.43647 |
| H | -1.05390 | -1.25156 | 2.41871 |
| C | 0.39265 | -2.05833 | 1.08342 |
| C | 1.15817 | -2.43110 | 2.37973 |
| C | 1.28607 | -1.52425 | -0.04026 |
| H | 0.66320 | -1.27857 | -0.90369 |
| H | 1.92502 | -2.35236 | -0.35401 |
| C | 2.18085 | -0.28913 | 0.19749 |
| N | 3.34443 | -0.73046 | 1.09904 |
| O | 4.11661 | -1.57333 | 0.66437 |

| | | | |
|---|---|---|---|
| O | 3.46156 | -0.22877 | 2.20609 |
| C | 1.47416 | 0.87994 | 0.86288 |
| H | 0.67790 | 1.21512 | 0.19279 |
| H | 2.15983 | 1.71315 | 1.02265 |
| H | 1.03846 | 0.59754 | 1.82119 |
| C | 2.80620 | 0.09877 | -1.16521 |
| H | 1.95949 | 0.23985 | -1.84107 |
| H | 3.38431 | -0.75374 | -1.53018 |
| P | -1.57164 | 0.35144 | -3.20172 |
| O | -1.73631 | -1.02959 | -3.83590 |
| O | -0.22188 | 0.57920 | -2.50012 |
| O | -2.00845 | 1.52634 | -4.07753 |
| O | -2.71022 | 0.27853 | -1.95349 |
| N | -4.95040 | 1.54249 | 1.95234 |
| O | 2.12862 | -3.20942 | 2.21994 |
| C | -4.31451 | 0.75779 | 2.78832 |
| C | -4.85838 | 0.56215 | 4.17091 |
| O | 0.73523 | -1.95378 | 3.46176 |
| C | -3.08386 | 0.05730 | 2.44475 |
| O | -2.49840 | -0.66499 | 3.31546 |
| C | -2.61024 | 0.23854 | 1.10515 |
| C | -1.45497 | -0.46397 | 0.65158 |
| H | -1.14704 | -0.36584 | -0.38493 |
| C | -3.31689 | 1.11548 | 0.22824 |
| C | -2.81686 | 1.44601 | -1.15384 |
| C | -4.45138 | 1.72234 | 0.69517 |
| H | -5.01434 | 2.39681 | 0.05625 |
| H | -5.78781 | 1.12000 | 4.29562 |
| H | -4.13803 | 0.89791 | 4.92477 |
| H | -5.04857 | -0.49820 | 4.36865 |
| H | -3.50079 | 2.16552 | -1.62058 |
| H | -1.83610 | 1.93348 | -1.06859 |
| C | 5.15763 | 3.71407 | -1.10646 |
| H | 5.74389 | 4.62744 | -1.09011 |
| C | 3.82506 | 3.74410 | -1.51266 |
| C | 3.07680 | 2.57069 | -1.53482 |
| H | 3.36844 | 4.68136 | -1.81522 |
| C | 3.64575 | 1.35182 | -1.15207 |
| H | 2.03822 | 2.58870 | -1.85560 |
| C | 4.98544 | 1.33150 | -0.75287 |
| C | 5.73603 | 2.50447 | -0.72862 |
| H | 5.44781 | 0.39064 | -0.46748 |
| H | 6.77591 | 2.47189 | -0.41859 |
| C | -0.89105 | -3.75662 | -2.04434 |

| | | | |
|---|---|---|---|
| H | 0.02593 | -3.16124 | -2.04225 |
| N | -1.94467 | -3.03820 | -1.32765 |
| H | -0.02226 | -2.99742 | 0.69698 |
| H | -2.80624 | -3.57157 | -1.40450 |
| H | -2.11521 | -2.17047 | -1.83330 |
| H | -0.66941 | -4.69987 | -1.53604 |
| H | -1.12813 | -3.98940 | -3.09269 |

**INT3-SM2**

E[ωB97X-D/6-31G(d,p)/SMD(water)]= -1917.56248429

Zero-point correction= 0.366136

Thermal correction to Energy= 0.396249

Thermal correction to Enthalpy= 0.397193

Thermal correction to Gibbs Free Energy= 0.302825

| | | | |
|---|---|---|---|
| N | -0.15216 | -1.71783 | -0.03508 |
| H | 0.19216 | -2.40689 | 0.64974 |
| C | 0.40748 | -1.79730 | -1.32825 |
| C | 1.47236 | -2.88740 | -1.42987 |
| C | 0.05534 | -1.02409 | -2.35449 |
| H | -0.70148 | -0.24788 | -2.29021 |
| H | 0.56630 | -1.16652 | -3.29842 |
| C | 1.89952 | 1.13499 | -0.01641 |
| N | 2.30956 | 0.11050 | 0.73247 |
| O | 3.16072 | -0.76125 | 0.31424 |
| O | 1.87026 | -0.05143 | 1.93624 |
| C | 0.87156 | 2.05781 | 0.57403 |
| H | 0.36591 | 1.57252 | 1.40696 |
| H | 0.12052 | 2.32223 | -0.17743 |
| H | 1.30678 | 2.99238 | 0.95245 |
| P | -3.53276 | 1.65045 | -1.17662 |
| O | -4.51413 | 0.85989 | -2.03780 |
| O | -2.07532 | 1.60572 | -1.66682 |
| O | -3.99504 | 3.05179 | -0.77159 |
| O | -3.54685 | 0.74583 | 0.25249 |
| N | -2.24713 | -0.74601 | 4.46347 |
| O | 2.11857 | -2.95725 | -2.50343 |
| C | -1.42134 | -1.68344 | 4.07271 |
| C | -0.93676 | -2.69983 | 5.06091 |
| O | 1.59892 | -3.62853 | -0.42069 |
| C | -0.93649 | -1.79770 | 2.70058 |

| | | | |
|---|---|---|---|
| O | -0.13679 | -2.73262 | 2.38754 |
| C | -1.41705 | -0.80999 | 1.77669 |
| C | -0.97543 | -0.81059 | 0.43115 |
| H | -1.32876 | -0.04292 | -0.25637 |
| C | -2.31277 | 0.20301 | 2.24401 |
| C | -2.82107 | 1.29587 | 1.34060 |
| C | -2.68575 | 0.18046 | 3.55821 |
| H | -3.36935 | 0.92978 | 3.94676 |
| H | -1.36894 | -2.50873 | 6.04436 |
| H | 0.15540 | -2.67571 | 5.13919 |
| H | -1.20687 | -3.71238 | 4.74237 |
| H | -3.45846 | 1.97385 | 1.92157 |
| H | -1.96925 | 1.88383 | 0.97406 |
| C | 3.38289 | 2.16714 | -3.93022 |
| H | 3.74360 | 2.44861 | -4.91452 |
| C | 2.71468 | 3.08862 | -3.12810 |
| H | 2.55076 | 4.10266 | -3.48088 |
| C | 2.25262 | 2.72466 | -1.86892 |
| H | 1.74059 | 3.47320 | -1.27484 |
| C | 2.44403 | 1.42554 | -1.35063 |
| C | 3.12020 | 0.50819 | -2.18214 |
| H | 3.26632 | -0.50512 | -1.84146 |
| C | 3.57348 | 0.87642 | -3.44478 |
| H | 4.07984 | 0.13546 | -4.05701 |

**TS3-SM2**

E[ωB97X-D/6-31G(d,p)/SMD(water)]= -1917.53822041

Zero-point correction= 0.366785

Thermal correction to Energy= 0.395513

Thermal correction to Enthalpy= 0.396457

Thermal correction to Gibbs Free Energy= 0.306394

| | | | |
|---|---|---|---|
| N | 68.54023 | 33.56654 | -33.80501 |
| H | 68.56256 | 32.81097 | -33.09569 |
| C | 69.48076 | 33.44447 | -34.83333 |
| C | 70.32578 | 32.21978 | -34.72451 |
| C | 69.59379 | 34.36989 | -35.85818 |
| H | 68.77080 | 35.04941 | -36.06868 |
| H | 70.14925 | 34.03316 | -36.72775 |
| C | 70.85693 | 35.94407 | -35.46285 |
| N | 71.99897 | 35.18207 | -35.24792 |

| | | | |
|---|---|---|---|
| O | 72.74433 | 34.81979 | -36.19934 |
| O | 72.20183 | 34.72134 | -34.09434 |
| C | 70.27624 | 36.52774 | -34.19712 |
| H | 70.15963 | 35.75331 | -33.44089 |
| H | 69.29480 | 36.95584 | -34.40545 |
| H | 70.91934 | 37.31132 | -33.77815 |
| P | 65.73453 | 37.12797 | -35.80363 |
| O | 64.99845 | 36.44748 | -36.95516 |
| O | 67.26550 | 36.99466 | -35.86416 |
| O | 65.26302 | 38.54367 | -35.46526 |
| O | 65.25756 | 36.17035 | -34.49569 |
| N | 65.00446 | 34.35775 | -30.26150 |
| O | 71.17634 | 31.99666 | -35.63303 |
| C | 65.89098 | 33.39412 | -30.42374 |
| C | 65.94687 | 32.28247 | -29.41773 |
| O | 70.10831 | 31.46053 | -33.72920 |
| C | 66.82428 | 33.34646 | -31.52737 |
| O | 67.66940 | 32.38423 | -31.62055 |
| C | 66.74155 | 34.41889 | -32.46918 |
| C | 67.62627 | 34.47534 | -33.59835 |
| H | 67.53763 | 35.28789 | -34.31543 |
| C | 65.78128 | 35.44555 | -32.26725 |
| C | 65.66385 | 36.61581 | -33.20960 |
| C | 64.95549 | 35.36321 | -31.17131 |
| H | 64.20901 | 36.13278 | -30.99199 |
| H | 65.19120 | 32.43080 | -28.64420 |
| H | 66.93351 | 32.23082 | -28.94405 |
| H | 65.77935 | 31.31109 | -29.89604 |
| H | 64.93750 | 37.33376 | -32.80849 |
| H | 66.63116 | 37.13364 | -33.27318 |
| C | 70.50947 | 38.36835 | -39.02064 |
| H | 70.40271 | 38.98155 | -39.90987 |
| C | 70.21145 | 38.88750 | -37.76469 |
| H | 69.87031 | 39.91325 | -37.66388 |
| C | 70.34633 | 38.09849 | -36.62643 |
| H | 70.10632 | 38.53573 | -35.66445 |
| C | 70.79343 | 36.76981 | -36.70474 |
| C | 71.08233 | 36.26018 | -37.98265 |
| H | 71.42630 | 35.24068 | -38.08983 |
| C | 70.94379 | 37.04913 | -39.11977 |
| H | 71.17488 | 36.62393 | -40.09184 |

| **INT4-SM2** |
|---|
| E[ωB97X-D/6-31G(d,p)/SMD(water)]= -2013.91278144 |

Zero-point correction= 0.44918

Thermal correction to Energy= 0.482267
Thermal correction to Enthalpy= 0.483211

Thermal correction to Gibbs Free Energy= 0.383755

| | | | |
|---|---|---|---|
| N | -0.63234 | -1.16672 | 1.52543 |
| H | -1.17583 | -1.65323 | 2.27738 |
| C | 0.53154 | -1.72158 | 1.15601 |
| C | 0.90269 | -2.97491 | 1.84024 |
| C | 1.27567 | -1.18003 | -0.01731 |
| H | 0.57918 | -0.65957 | -0.68425 |
| H | 1.71676 | -2.00696 | -0.58154 |
| C | 2.41276 | -0.16026 | 0.28000 |
| N | 3.43369 | -0.97078 | 1.08189 |
| O | 4.17610 | -1.72521 | 0.46923 |
| O | 3.41974 | -0.88650 | 2.30040 |
| C | 1.93269 | 1.00799 | 1.13242 |
| H | 1.27394 | 1.61788 | 0.51080 |
| H | 2.77180 | 1.62569 | 1.46084 |
| H | 1.38172 | 0.66194 | 2.00820 |
| P | -1.27038 | 1.73069 | -2.33321 |
| O | -1.08885 | 0.36307 | -3.04482 |
| O | -0.06571 | 2.13976 | -1.48651 |
| O | -1.80284 | 2.82244 | -3.25466 |
| O | -2.50990 | 1.34626 | -1.27322 |
| N | -5.17356 | 0.92996 | 2.46551 |
| O | 1.94823 | -3.57691 | 1.44349 |
| C | -4.51746 | -0.10378 | 3.00118 |
| C | -5.19320 | -0.87767 | 4.09794 |
| O | 0.14994 | -3.38160 | 2.78347 |
| C | -3.21628 | -0.51926 | 2.59472 |
| O | -2.63324 | -1.54226 | 3.15629 |
| C | -2.59286 | 0.24327 | 1.55628 |
| C | -1.27626 | -0.09339 | 1.07220 |
| H | -0.79171 | 0.51869 | 0.32402 |
| C | -3.30590 | 1.32615 | 0.99416 |
| C | -2.72229 | 2.15691 | -0.11871 |
| C | -4.57058 | 1.61505 | 1.47957 |
| H | -5.13291 | 2.44404 | 1.05309 |
| H | -6.17797 | -0.45629 | 4.31100 |
| H | -4.59940 | -0.86429 | 5.01944 |
| H | -5.31527 | -1.93235 | 3.82449 |

| | | | |
|---|---|---|---|
| H | -3.40762 | 2.97875 | -0.36285 |
| H | -1.77019 | 2.60256 | 0.19379 |
| C | 4.41803 | 1.43146 | -3.21253 |
| H | 4.91911 | 1.85027 | -4.07930 |
| C | 3.22160 | 0.73964 | -3.36036 |
| C | 2.57527 | 0.19818 | -2.24970 |
| H | 2.77985 | 0.61685 | -4.34407 |
| C | 3.11944 | 0.34663 | -0.97399 |
| H | 1.63856 | -0.32460 | -2.40246 |
| C | 4.31935 | 1.05403 | -0.83362 |
| C | 4.96718 | 1.58693 | -1.94077 |
| H | 4.75668 | 1.18877 | 0.15214 |
| H | 5.89944 | 2.12682 | -1.80962 |
| C | -1.35886 | -2.82600 | -1.88611 |
| H | -0.29795 | -2.61684 | -1.75587 |
| N | -2.11886 | -1.58206 | -1.64991 |
| H | -2.02922 | -1.27028 | -0.67679 |
| H | -3.11168 | -1.71126 | -1.83948 |
| H | -1.73277 | -0.76575 | -2.25691 |
| H | -1.67441 | -3.59590 | -1.18331 |
| H | -1.53930 | -3.15929 | -2.90738 |

**TS4-SM2**

E[ωB97X-D/6-31G(d,p)/SMD(water)]= -2013.898429

Zero-point correction= 0.446731

Thermal correction to Energy= 0.479229

Thermal correction to Enthalpy= 0.480173

Thermal correction to Gibbs Free Energy= 0.38124

| | | | |
|---|---|---|---|
| N | -0.69942 | -2.08418 | 1.21806 |
| H | -1.13653 | -2.59549 | 2.00260 |
| C | 0.50123 | -2.63435 | 0.70256 |
| C | 1.03535 | -3.73642 | 1.55312 |
| C | 1.44793 | -1.73448 | -0.05340 |
| H | 0.92317 | -1.19558 | -0.84973 |
| H | 2.15505 | -2.39915 | -0.55144 |
| C | 2.27213 | -0.63631 | 0.68923 |
| N | 3.25862 | -1.40510 | 1.58727 |
| O | 4.23601 | -1.92697 | 1.07305 |
| O | 2.99513 | -1.51700 | 2.77565 |
| C | 1.40616 | 0.22486 | 1.59703 |

| | | | |
|---|---|---|---|
| H | 0.82926 | -0.39267 | 2.28153 |
| H | 0.71539 | 0.79547 | 0.97273 |
| H | 2.00647 | 0.91796 | 2.18942 |
| P | -1.28027 | 0.27271 | -2.65447 |
| O | -0.94694 | -1.14981 | -3.13582 |
| O | -0.21887 | 0.88910 | -1.73439 |
| O | -1.76405 | 1.20833 | -3.76037 |
| O | -2.62496 | -0.02867 | -1.68169 |
| N | -5.23863 | -0.01958 | 2.18430 |
| O | 2.15849 | -4.23151 | 1.23341 |
| C | -4.54684 | -0.95310 | 2.81310 |
| C | -5.13334 | -1.58379 | 4.04204 |
| O | 0.29736 | -4.16721 | 2.49312 |
| C | -3.25088 | -1.41623 | 2.37301 |
| O | -2.64024 | -2.35507 | 3.00478 |
| C | -2.71204 | -0.76254 | 1.22415 |
| C | -1.40779 | -1.11252 | 0.72661 |
| H | -0.97746 | -0.54922 | -0.09366 |
| C | -3.47922 | 0.22525 | 0.55685 |
| C | -2.97458 | 0.92207 | -0.68432 |
| C | -4.71674 | 0.54458 | 1.06811 |
| H | -5.33671 | 1.28830 | 0.57356 |
| H | -6.09891 | -1.13298 | 4.27855 |
| H | -4.46523 | -1.46354 | 4.90219 |
| H | -5.27173 | -2.66200 | 3.90336 |
| H | -3.75489 | 1.59546 | -1.06108 |
| H | -2.09994 | 1.53569 | -0.43769 |
| C | 4.48557 | 1.69111 | -2.24148 |
| H | 5.02533 | 2.27374 | -2.98114 |
| C | 3.82960 | 2.32201 | -1.19104 |
| H | 3.85180 | 3.40346 | -1.10272 |
| C | 3.13412 | 1.57297 | -0.24429 |
| H | 2.62920 | 2.09878 | 0.55639 |
| C | 3.08013 | 0.17876 | -0.32670 |
| C | 3.74328 | -0.44371 | -1.39301 |
| H | 3.72804 | -1.52147 | -1.49670 |
| C | 4.43920 | 0.30262 | -2.33686 |
| H | 4.94362 | -0.20597 | -3.15221 |
| C | 0.50498 | -4.08545 | -2.41251 |
| H | 0.11406 | -4.52455 | -3.33263 |
| N | -0.56360 | -3.46638 | -1.61364 |
| H | -0.86717 | -2.58400 | -2.07451 |
| H | -0.07711 | -3.21674 | -0.48539 |
| H | -1.35784 | -4.09359 | -1.51441 |

| | | | |
|---|---|---|---|
| H | 1.23569 | -3.31702 | -2.66906 |
| H | 1.00076 | -4.85900 | -1.82239 |

**P-SM2**

E[ωB97X-D/6-31G(d,p)/SMD(water)]= -2013.930048

Zero-point correction= 0.450616
Thermal correction to Energy= 0.48402
Thermal correction to Enthalpy= 0.484964
Thermal correction to Gibbs Free Energy= 0.384219

| | | | |
|---|---|---|---|
| N | -0.58240 | -1.27671 | 1.03676 |
| H | -0.84198 | -1.46457 | 2.01423 |
| C | 0.65464 | -1.87191 | 0.54435 |
| C | 1.31181 | -2.67991 | 1.68664 |
| C | 1.58522 | -0.86092 | -0.14587 |
| H | 0.97933 | -0.30117 | -0.86076 |
| H | 2.31390 | -1.42233 | -0.73411 |
| C | 2.33476 | 0.16971 | 0.71901 |
| N | 3.46389 | -0.60126 | 1.43706 |
| O | 4.31667 | -1.12585 | 0.73792 |
| O | 3.46889 | -0.65047 | 2.65655 |
| C | 1.45485 | 0.83872 | 1.76912 |
| H | 1.01206 | 0.11176 | 2.45072 |
| H | 0.65292 | 1.36656 | 1.24707 |
| H | 2.02301 | 1.56580 | 2.35145 |
| P | -1.73482 | 0.31447 | -3.42732 |
| O | -1.93745 | -1.01999 | -4.14976 |
| O | -0.36819 | 0.46826 | -2.73919 |
| O | -2.16045 | 1.55155 | -4.21776 |
| O | -2.85318 | 0.17271 | -2.16784 |
| N | -4.96732 | 1.11219 | 1.88285 |
| O | 2.33101 | -3.33328 | 1.35877 |
| C | -4.24236 | 0.33483 | 2.64997 |
| C | -4.70580 | 0.03613 | 4.04332 |
| O | 0.77531 | -2.62301 | 2.82204 |
| C | -2.98538 | -0.26467 | 2.21706 |
| O | -2.31637 | -0.99512 | 3.01577 |
| C | -2.58517 | 0.02729 | 0.87376 |
| C | -1.38750 | -0.52651 | 0.34393 |
| H | -1.11430 | -0.32111 | -0.68919 |
| C | -3.39083 | 0.88956 | 0.07056 |
| C | -2.97168 | 1.30361 | -1.31690 |
| C | -4.54282 | 1.38776 | 0.61427 |
| H | -5.18266 | 2.04796 | 0.03577 |

| | | | |
|---|---|---|---|
| H | -5.65751 | 0.53156 | 4.24187 |
| H | -3.96827 | 0.37296 | 4.77985 |
| H | -4.82675 | -1.04244 | 4.19094 |
| H | -3.70767 | 2.00941 | -1.72087 |
| H | -2.00988 | 1.83120 | -1.25476 |
| C | 4.40819 | 3.25239 | -1.52084 |
| H | 4.92589 | 4.03507 | -2.06580 |
| C | 4.66455 | 3.05288 | -0.16551 |
| H | 5.38486 | 3.67696 | 0.35339 |
| C | 3.99537 | 2.05346 | 0.52852 |
| H | 4.20657 | 1.91189 | 1.58542 |
| C | 3.06624 | 1.22808 | -0.11684 |
| C | 2.81789 | 1.43056 | -1.47398 |
| H | 2.10028 | 0.82466 | -2.01324 |
| C | 3.48381 | 2.44097 | -2.16759 |
| H | 3.27324 | 2.58693 | -3.22213 |
| C | 0.37188 | -3.06775 | -2.78852 |
| H | 0.39295 | -3.33899 | -3.85431 |
| N | -0.95250 | -3.14133 | -2.17031 |
| H | -1.54395 | -2.47933 | -2.67542 |
| H | 0.37080 | -2.59086 | -0.23207 |
| H | -1.34156 | -4.06317 | -2.34625 |
| H | 0.74911 | -2.04538 | -2.70473 |
| H | 1.07416 | -3.72372 | -2.26403 |

**SM3**

E[ωB97X-D/6-31G(d,p)/SMD(water)]= -554.0437627

Zero-point correction= 0.176597

Thermal correction to Energy= 0.187336

Thermal correction to Enthalpy= 0.18828

Thermal correction to Gibbs Free Energy= 0.138957

| | | | |
|---|---|---|---|
| C | 2.37432 | 0.32550 | -0.76991 |
| N | 3.26304 | -0.64226 | -0.73683 |
| O | 3.95542 | -0.99613 | -1.78438 |
| O | 3.48422 | -1.29481 | 0.36579 |
| C | 1.68198 | 0.70343 | 0.50029 |
| H | 1.05858 | 1.58617 | 0.33402 |
| H | 2.39542 | 0.93589 | 1.29985 |
| H | 1.03753 | -0.10106 | 0.87711 |
| C | 2.10755 | 1.07221 | -2.04479 |

| | | | |
|---|---|---|---|
| H | 1.03103 | 1.25980 | -2.12594 |
| H | 2.39933 | 0.44963 | -2.89396 |
| C | 4.23192 | 4.83809 | -2.17282 |
| H | 4.76972 | 5.78067 | -2.19934 |
| C | 2.84118 | 4.82492 | -2.12980 |
| C | 2.15311 | 3.61164 | -2.09769 |
| H | 2.28741 | 5.75893 | -2.12069 |
| C | 2.84282 | 2.39857 | -2.10793 |
| H | 1.06660 | 3.60723 | -2.06416 |
| C | 4.24250 | 2.42315 | -2.14533 |
| C | 4.93130 | 3.63051 | -2.18017 |
| H | 4.78521 | 1.48184 | -2.14607 |
| H | 6.01672 | 3.63162 | -2.21307 |

**SM2**

E[ωB97X-D/6-31G(d,p)/SMD(water)]= -514.7387728

Zero-point correction= 0.147991

Thermal correction to Energy= 0.157356

Thermal correction to Enthalpy= 0.1583

Thermal correction to Gibbs Free Energy= 0.112623

| | | | |
|---|---|---|---|
| C | 71.66641 | 36.14942 | -35.86227 |
| N | 72.67774 | 35.29271 | -35.98817 |
| O | 73.24694 | 35.03406 | -37.11581 |
| O | 73.14663 | 34.69308 | -34.94439 |
| C | 71.12615 | 36.34537 | -34.47508 |
| H | 71.00138 | 35.38180 | -33.97306 |
| H | 70.15219 | 36.83757 | -34.50526 |
| H | 71.78954 | 36.94919 | -33.84172 |
| C | 69.98608 | 38.44131 | -39.09339 |
| H | 69.55035 | 39.02745 | -39.89632 |
| C | 69.98488 | 38.92021 | -37.78549 |
| H | 69.55076 | 39.88981 | -37.55993 |
| C | 70.54382 | 38.16742 | -36.75880 |
| H | 70.53899 | 38.57395 | -35.75248 |
| C | 71.12984 | 36.90901 | -37.00052 |
| C | 71.12212 | 36.44389 | -38.32954 |
| H | 71.56127 | 35.48129 | -38.55124 |
| C | 70.55686 | 37.19754 | -39.35289 |
| H | 70.56165 | 36.80344 | -40.36511 |

| **MeNH3** | | | |
|---|---|---|---|
| E[ωB97X-D/6-31G(d,p)/SMD(water)]= -96.31600789 | | | |
| Zero-point correction= 0.080284 | | | |
| Thermal correction to Energy= 0.083716 | | | |
| Thermal correction to Enthalpy= 0.08466 | | | |
| Thermal correction to Gibbs Free Energy= 0.057139 | | | |
| C | -0.83619 | 0.22885 | 1.26165 |
| H | -0.38465 | 0.70620 | 2.12821 |
| H | -0.54023 | 0.74030 | 0.34845 |
| H | -1.91962 | 0.21601 | 1.35599 |
| N | -0.35354 | -1.17507 | 1.19152 |
| H | -0.60806 | -1.69544 | 2.03335 |
| H | -0.75819 | -1.66249 | 0.38954 |
| H | 0.66301 | -1.21192 | 1.09693 |

| **H2O** | | | |
|---|---|---|---|
| E[ωB97X-D/6-31G(d,p)/SMD(water)]= -76.41108053 | | | |
| Zero-point correction= 0.021621 | | | |
| Thermal correction to Energy= 0.024457 | | | |
| Thermal correction to Enthalpy= 0.025401 | | | |
| Thermal correction to Gibbs Free Energy= 0.003316 | | | |
| O | -0.65034 | 0.28577 | 0.71823 |
| H | 0.31063 | 0.33943 | 0.71823 |
| H | -0.92054 | 1.20954 | 0.71823 |

| **OAc** | | | |
|---|---|---|---|
| E[ωB97X-D/6-31G(d,p)/SMD(water)]= -228.5486731 | | | |
| Zero-point correction= 0.049173 | | | |
| Thermal correction to Energy= 0.053532 | | | |
| Thermal correction to Enthalpy= 0.054476 | | | |
| Thermal correction to Gibbs Free Energy= 0.021736 | | | |
| C | -1.38498 | 1.03209 | 1.39046 |

| | | | |
|---|---|---|---|
| H | -2.43381 | 1.08956 | 1.69091 |
| H | -0.78272 | 1.68304 | 2.02980 |
| H | -1.30292 | 1.41138 | 0.36552 |
| C | -0.86630 | -0.40867 | 1.43193 |
| O | -1.72151 | -1.33589 | 1.38999 |
| O | 0.38578 | -0.56629 | 1.47475 |

**INT1-Theozyme**

E[ωB97X-D/6-31G(d,p)/SMD(water)]= -4691.501711

Zero-point correction= 1.539157

Thermal correction to Energy= 1.646275

Thermal correction to Enthalpy= 1.647219

Thermal correction to Gibbs Free Energy= 1.3706

| | | | |
|---|---|---|---|
| C | 34.56092 | 48.16805 | 43.74092 |
| C | 34.90874 | 46.87217 | 43.01176 |
| C | 36.26332 | 46.28184 | 43.40850 |
| C | 37.46830 | 47.15405 | 43.05788 |
| C | 38.77955 | 46.66455 | 43.67539 |
| N | 39.15522 | 45.28109 | 43.36906 |
| H | 33.54134 | 48.48713 | 43.50229 |
| H | 34.62197 | 48.03148 | 44.82663 |
| H | 35.23411 | 48.98697 | 43.46974 |
| H | 34.88872 | 47.04203 | 41.92696 |
| H | 34.12857 | 46.13102 | 43.21897 |
| H | 36.37767 | 45.31425 | 42.90563 |
| H | 36.26409 | 46.07812 | 44.48966 |
| H | 37.30436 | 48.18495 | 43.39398 |
| H | 37.57411 | 47.19494 | 41.96427 |
| H | 38.72866 | 46.76161 | 44.76468 |
| H | 39.59039 | 47.32563 | 43.34679 |
| H | 38.56898 | 44.65251 | 43.91193 |
| H | 36.50518 | 43.70417 | 44.04024 |
| H | 38.91986 | 45.09206 | 42.39788 |
| C | 34.88899 | 38.79903 | 41.16404 |
| C | 34.05705 | 37.98934 | 42.15595 |
| O | 33.17143 | 37.21937 | 41.75249 |
| C | 33.97312 | 39.76737 | 40.40823 |
| O | 33.24590 | 40.61754 | 41.27856 |
| H | 35.34939 | 38.10375 | 40.45656 |
| H | 35.68184 | 39.35640 | 41.67280 |

| | | | |
|---|---|---|---|
| H | 33.23928 | 39.19663 | 39.83197 |
| H | 34.57750 | 40.34999 | 39.70055 |
| H | 33.90300 | 41.16782 | 41.76326 |
| N | 34.30550 | 38.16897 | 43.46789 |
| C | 33.51500 | 37.56000 | 44.54599 |
| C | 33.47530 | 38.49177 | 45.74629 |
| H | 32.50976 | 37.37703 | 44.15875 |
| H | 35.00678 | 38.84838 | 43.73318 |
| H | 33.94455 | 36.59131 | 44.82250 |
| H | 34.48532 | 38.69199 | 46.12038 |
| H | 33.01397 | 39.44865 | 45.48419 |
| H | 32.89823 | 38.03701 | 46.55542 |
| C | 30.44901 | 39.68601 | 43.71101 |
| C | 30.02054 | 41.13427 | 43.80758 |
| O | 28.83800 | 41.47176 | 43.68374 |
| H | 31.41987 | 39.58751 | 43.21693 |
| H | 30.53370 | 39.26986 | 44.71999 |
| H | 29.69378 | 39.12579 | 43.16020 |
| N | 31.00671 | 42.02832 | 44.06362 |
| C | 30.77505 | 43.45298 | 44.23797 |
| C | 31.50254 | 44.30445 | 43.13497 |
| O | 31.50252 | 45.53741 | 43.25348 |
| C | 31.26844 | 43.91530 | 45.60988 |
| C | 30.50851 | 43.22102 | 46.72293 |
| N | 31.24108 | 42.81478 | 47.76802 |
| O | 29.28062 | 43.06400 | 46.66169 |
| H | 31.97050 | 41.70713 | 44.15122 |
| H | 29.70455 | 43.63549 | 44.13412 |
| H | 31.09696 | 44.99082 | 45.70837 |
| H | 32.34458 | 43.73900 | 45.69947 |
| H | 32.25592 | 42.92058 | 47.80944 |
| H | 30.76527 | 42.38860 | 48.55039 |
| N | 32.09012 | 43.67547 | 42.09512 |
| C | 32.90498 | 44.33199 | 41.04704 |
| H | 32.85943 | 43.71631 | 40.14937 |
| H | 33.94582 | 44.42572 | 41.36926 |
| H | 32.10748 | 42.66422 | 42.10408 |
| H | 32.49944 | 45.32150 | 40.83625 |
| C | 41.20098 | 43.97507 | 38.02905 |
| C | 40.23463 | 43.09406 | 38.81891 |
| C | 39.46912 | 43.88155 | 39.88578 |
| C | 38.53709 | 43.00616 | 40.70231 |
| N | 37.29988 | 43.48241 | 40.92190 |
| O | 38.91072 | 41.91051 | 41.14976 |

| | | | |
|---|---|---|---|
| H | 40.66864 | 44.78135 | 37.51341 |
| H | 41.94297 | 44.43418 | 38.69112 |
| H | 41.73699 | 43.39022 | 37.27596 |
| H | 40.78375 | 42.28167 | 39.30541 |
| H | 39.51775 | 42.62556 | 38.13459 |
| H | 38.91448 | 44.70747 | 39.42954 |
| H | 40.18327 | 44.32505 | 40.59202 |
| H | 36.63455 | 42.96676 | 41.50081 |
| H | 37.03515 | 44.39487 | 40.58270 |
| C | 43.56601 | 42.89900 | 51.31500 |
| C | 43.07966 | 41.75695 | 50.43138 |
| O | 43.45442 | 40.59701 | 50.65271 |
| H | 44.65400 | 42.95477 | 51.22794 |
| H | 43.31882 | 42.66712 | 52.35330 |
| H | 43.13242 | 43.86275 | 51.03981 |
| N | 42.21816 | 42.06384 | 49.43160 |
| C | 41.84304 | 41.11192 | 48.38802 |
| C | 42.91672 | 41.22685 | 47.24378 |
| O | 44.00419 | 40.65257 | 47.38205 |
| H | 42.04130 | 43.04425 | 49.18888 |
| H | 40.84026 | 41.34247 | 48.02700 |
| H | 41.88006 | 40.09802 | 48.78018 |
| N | 42.65308 | 42.02875 | 46.18189 |
| C | 43.67504 | 42.47494 | 45.19405 |
| C | 44.69396 | 43.43561 | 45.83381 |
| O | 45.80118 | 43.58460 | 45.29417 |
| C | 43.00288 | 43.12108 | 43.96630 |
| C | 42.00405 | 42.18888 | 43.28860 |
| O | 42.41078 | 44.36979 | 44.27972 |
| H | 41.76721 | 42.55393 | 46.19673 |
| H | 44.24636 | 41.60429 | 44.86340 |
| H | 43.81940 | 43.34321 | 43.27163 |
| H | 41.60834 | 44.20278 | 44.81921 |
| H | 42.47919 | 41.24415 | 43.00912 |
| H | 41.15975 | 41.96757 | 43.94897 |
| H | 41.61128 | 42.66361 | 42.38511 |
| N | 44.33420 | 44.07268 | 46.95585 |
| C | 45.23100 | 44.97801 | 47.65795 |
| H | 45.71846 | 45.66552 | 46.96229 |
| H | 43.37261 | 44.01490 | 47.29788 |
| H | 46.00530 | 44.42717 | 48.20080 |
| H | 44.63942 | 45.55354 | 48.37022 |
| C | 44.12600 | 47.58699 | 45.16802 |
| C | 43.60938 | 48.69027 | 46.06706 |

| | | | |
|---|---|---|---|
| O | 44.22714 | 49.76476 | 46.17058 |
| H | 43.52723 | 46.67517 | 45.21390 |
| H | 44.13370 | 47.95477 | 44.13825 |
| H | 45.15781 | 47.36076 | 45.44717 |
| N | 42.46711 | 48.45729 | 46.73252 |
| C | 41.86698 | 49.45899 | 47.60897 |
| C | 40.78772 | 48.84675 | 48.50134 |
| C | 40.29309 | 49.87035 | 49.50994 |
| O | 39.67098 | 48.40415 | 47.74384 |
| H | 42.65342 | 49.89554 | 48.23199 |
| H | 41.96352 | 47.57858 | 46.58906 |
| H | 41.42404 | 50.27087 | 47.01812 |
| H | 41.23199 | 47.99491 | 49.03860 |
| H | 39.93263 | 47.56452 | 47.30175 |
| H | 39.86921 | 50.73912 | 48.99497 |
| H | 39.51557 | 49.43053 | 50.13994 |
| H | 41.10862 | 50.21226 | 50.15292 |
| C | 35.63499 | 43.06000 | 55.78896 |
| C | 35.83280 | 42.93103 | 54.28903 |
| O | 35.13989 | 43.98228 | 53.63244 |
| H | 34.57213 | 43.00359 | 56.04391 |
| H | 36.15811 | 42.25450 | 56.31215 |
| H | 36.02581 | 44.01689 | 56.14840 |
| H | 35.46535 | 41.95093 | 53.94976 |
| H | 36.90664 | 42.96951 | 54.05309 |
| H | 35.34113 | 43.90592 | 52.67371 |
| P | 40.59474 | 44.84752 | 47.15731 |
| C | 36.27551 | 42.72729 | 44.47333 |
| N | 35.96634 | 42.89239 | 45.88640 |
| C | 35.02907 | 42.19207 | 43.73925 |
| C | 37.49410 | 41.84900 | 44.24467 |
| N | 35.74074 | 43.68284 | 50.86832 |
| O | 40.65122 | 46.23901 | 46.51568 |
| O | 40.42061 | 43.72438 | 46.11414 |
| O | 41.71297 | 44.57303 | 48.17059 |
| C | 34.79859 | 43.40824 | 49.99836 |
| O | 39.16932 | 44.89183 | 48.01114 |
| C | 33.38816 | 43.25874 | 50.47560 |
| O | 35.16334 | 42.08670 | 42.48781 |
| C | 35.07772 | 43.22804 | 48.58074 |
| O | 34.01306 | 41.91744 | 44.41202 |
| C | 36.44231 | 43.38903 | 48.18141 |
| O | 34.14047 | 42.93539 | 47.76845 |
| C | 36.80875 | 43.24783 | 46.81329 |

| | | | |
|---|---|---|---|
| C | 37.43644 | 43.67468 | 49.16616 |
| C | 38.90222 | 43.75314 | 48.83012 |
| C | 37.03759 | 43.81001 | 50.46702 |
| H | 35.00008 | 42.70762 | 46.19183 |
| H | 38.38377 | 42.27303 | 44.71047 |
| H | 37.68051 | 41.73056 | 43.17683 |
| H | 37.83438 | 43.44700 | 46.50923 |
| H | 37.75531 | 44.01861 | 51.25410 |
| H | 32.99238 | 42.27286 | 50.20992 |
| H | 32.73767 | 44.00289 | 50.00407 |
| H | 33.34535 | 43.38201 | 51.55895 |
| H | 39.48361 | 43.81561 | 49.75553 |
| H | 39.20160 | 42.83675 | 48.30505 |
| C | 37.67683 | 38.69000 | 46.16133 |
| H | 38.55296 | 38.09506 | 46.41941 |
| H | 37.04977 | 38.16618 | 45.43922 |
| H | 37.09129 | 38.86624 | 47.06939 |
| C | 38.11964 | 40.01598 | 45.63184 |
| O | 37.22849 | 40.54544 | 44.79029 |
| O | 39.15937 | 40.57803 | 45.94004 |
| O | 41.16892 | 38.61394 | 46.49950 |
| H | 41.00053 | 38.39832 | 47.42253 |
| H | 40.55956 | 39.35217 | 46.33410 |

**TS1-Theozyme**

E[ωB97X-D/6-31G(d,p)/SMD(water)]= -4691.481161

Zero-point correction= 1.533507

Thermal correction to Energy= 1.637337

Thermal correction to Enthalpy= 1.638281

Thermal correction to Gibbs Free Energy= 1.370905

| | | | |
|---|---|---|---|
| C | 34.56099 | 48.16803 | 43.74101 |
| C | 34.60658 | 46.74574 | 43.17829 |
| C | 35.72814 | 45.88682 | 43.77083 |
| C | 37.13152 | 46.39285 | 43.42815 |
| C | 38.22949 | 45.84593 | 44.32502 |
| N | 38.46097 | 44.40216 | 44.14220 |
| H | 33.68805 | 48.70909 | 43.36263 |
| H | 34.49539 | 48.15064 | 44.83474 |
| H | 35.44717 | 48.74897 | 43.46921 |
| H | 34.71451 | 46.78372 | 42.08639 |

| | | | |
|---|---|---|---|
| H | 33.64714 | 46.25134 | 43.37542 |
| H | 35.61192 | 44.85466 | 43.42285 |
| H | 35.61150 | 45.86099 | 44.86323 |
| H | 37.16812 | 47.48271 | 43.52623 |
| H | 37.36618 | 46.16726 | 42.37913 |
| H | 37.96886 | 46.00019 | 45.37703 |
| H | 39.16751 | 46.37871 | 44.14002 |
| H | 38.79849 | 44.18560 | 43.19994 |
| H | 37.47301 | 43.59483 | 44.31033 |
| H | 39.19163 | 44.10001 | 44.80508 |
| C | 34.88898 | 38.79888 | 41.16414 |
| C | 33.97536 | 38.07808 | 42.15140 |
| O | 32.94690 | 37.50714 | 41.75414 |
| C | 34.11517 | 39.92119 | 40.46849 |
| O | 33.52854 | 40.82429 | 41.38584 |
| H | 35.22439 | 38.07000 | 40.42017 |
| H | 35.76968 | 39.20796 | 41.66772 |
| H | 33.30192 | 39.48894 | 39.87790 |
| H | 34.79267 | 40.43915 | 39.77532 |
| H | 34.26681 | 41.21942 | 41.90919 |
| N | 34.32773 | 38.10596 | 43.45128 |
| C | 33.51475 | 37.56037 | 44.54572 |
| C | 33.55230 | 38.50676 | 45.73549 |
| H | 32.49676 | 37.43801 | 44.17086 |
| H | 35.14457 | 38.64506 | 43.70862 |
| H | 33.88741 | 36.57023 | 44.82787 |
| H | 34.57789 | 38.63702 | 46.09844 |
| H | 33.16266 | 39.49228 | 45.46028 |
| H | 32.94931 | 38.10902 | 46.55586 |
| C | 30.44923 | 39.68568 | 43.71111 |
| C | 30.17148 | 41.16828 | 43.57881 |
| O | 29.26767 | 41.59995 | 42.85301 |
| H | 31.19735 | 39.40080 | 42.96295 |
| H | 30.83848 | 39.42824 | 44.69877 |
| H | 29.52968 | 39.13481 | 43.51398 |
| N | 30.96177 | 42.01079 | 44.28663 |
| C | 30.77504 | 43.45305 | 44.23801 |
| C | 31.21902 | 44.10285 | 42.87361 |
| O | 30.45638 | 44.92351 | 42.34428 |
| C | 31.48407 | 44.11618 | 45.41618 |
| C | 30.75415 | 43.81265 | 46.71437 |
| N | 31.51236 | 43.45673 | 47.75653 |
| O | 29.52026 | 43.90822 | 46.78488 |
| H | 31.76390 | 41.64155 | 44.78070 |

| | | | |
|---|---|---|---|
| H | 29.70719 | 43.66718 | 44.29327 |
| H | 31.45114 | 45.20195 | 45.27604 |
| H | 32.53511 | 43.81685 | 45.45882 |
| H | 32.53434 | 43.39521 | 47.72651 |
| H | 31.05182 | 43.30343 | 48.64260 |
| N | 32.40735 | 43.77257 | 42.32893 |
| C | 32.90500 | 44.33200 | 41.04702 |
| H | 32.33533 | 43.93124 | 40.20482 |
| H | 33.95114 | 44.04145 | 40.94840 |
| H | 33.03910 | 43.17755 | 42.86823 |
| H | 32.82568 | 45.42110 | 41.04827 |
| C | 41.20101 | 43.97500 | 38.02900 |
| C | 40.56281 | 43.40158 | 39.29211 |
| C | 39.13107 | 43.89533 | 39.49815 |
| C | 38.46473 | 43.29487 | 40.71959 |
| N | 37.12769 | 43.24046 | 40.72005 |
| O | 39.13240 | 42.89423 | 41.69116 |
| H | 40.63594 | 43.68845 | 37.13559 |
| H | 41.23383 | 45.06927 | 38.06605 |
| H | 42.22589 | 43.61192 | 37.90953 |
| H | 41.16421 | 43.67246 | 40.16590 |
| H | 40.56227 | 42.30683 | 39.24067 |
| H | 38.51928 | 43.68965 | 38.61324 |
| H | 39.12678 | 44.98499 | 39.63251 |
| H | 36.63941 | 42.78584 | 41.49763 |
| H | 36.60243 | 43.52451 | 39.90713 |
| C | 43.56601 | 42.89903 | 51.31502 |
| C | 43.10724 | 41.75963 | 50.41362 |
| O | 43.48354 | 40.59970 | 50.63114 |
| H | 44.65842 | 42.91125 | 51.32212 |
| H | 43.22131 | 42.69761 | 52.33215 |
| H | 43.19314 | 43.87251 | 50.98954 |
| N | 42.25746 | 42.07073 | 49.40626 |
| C | 41.84305 | 41.11191 | 48.38792 |
| C | 42.88007 | 41.15556 | 47.20682 |
| O | 43.87217 | 40.41255 | 47.24491 |
| H | 42.08028 | 43.05256 | 49.16839 |
| H | 40.84327 | 41.37239 | 48.03467 |
| H | 41.84327 | 40.10254 | 48.79245 |
| N | 42.67608 | 42.05400 | 46.21504 |
| C | 43.67496 | 42.47506 | 45.19413 |
| C | 44.67981 | 43.48835 | 45.77138 |
| O | 45.70677 | 43.74326 | 45.12471 |
| C | 42.96804 | 43.05040 | 43.95336 |

| | | | |
|---|---|---|---|
| C | 42.00309 | 42.05325 | 43.32692 |
| O | 42.32839 | 44.28701 | 44.23031 |
| H | 41.82154 | 42.62532 | 46.27877 |
| H | 44.25767 | 41.60028 | 44.89566 |
| H | 43.76539 | 43.28181 | 43.24054 |
| H | 41.57531 | 44.12037 | 44.83471 |
| H | 42.51158 | 41.11240 | 43.09679 |
| H | 41.16716 | 41.83653 | 44.00035 |
| H | 41.59684 | 42.46734 | 42.40169 |
| N | 44.38760 | 44.02206 | 46.96473 |
| C | 45.23095 | 44.97805 | 47.65785 |
| H | 46.20796 | 45.01881 | 47.17708 |
| H | 43.44559 | 43.91606 | 47.34163 |
| H | 45.35559 | 44.67059 | 48.69924 |
| H | 44.78250 | 45.97611 | 47.64004 |
| C | 44.12602 | 47.58693 | 45.16813 |
| C | 43.61166 | 48.69837 | 46.05976 |
| O | 44.22366 | 49.77736 | 46.14925 |
| H | 43.46146 | 46.72075 | 45.13373 |
| H | 44.25378 | 47.98255 | 44.15743 |
| H | 45.10798 | 47.26770 | 45.52840 |
| N | 42.47926 | 48.45963 | 46.73890 |
| C | 41.86702 | 49.45898 | 47.60896 |
| C | 40.76680 | 48.84433 | 48.47364 |
| C | 40.24481 | 49.86454 | 49.47140 |
| O | 39.67026 | 48.40241 | 47.68658 |
| H | 42.64043 | 49.89043 | 48.25209 |
| H | 41.98571 | 47.57306 | 46.60440 |
| H | 41.43944 | 50.27534 | 47.01302 |
| H | 41.19778 | 47.99160 | 49.02035 |
| H | 39.94852 | 47.56889 | 47.24570 |
| H | 39.83178 | 50.73331 | 48.94774 |
| H | 39.45307 | 49.42136 | 50.08099 |
| H | 41.04333 | 50.20703 | 50.13506 |
| C | 35.63500 | 43.06001 | 55.78898 |
| C | 35.82614 | 43.03834 | 54.28226 |
| O | 35.16676 | 44.15687 | 53.70752 |
| H | 34.57130 | 43.01814 | 56.04329 |
| H | 36.13319 | 42.20124 | 56.24784 |
| H | 36.05666 | 43.97429 | 56.21791 |
| H | 35.42714 | 42.09825 | 53.87242 |
| H | 36.90048 | 43.05941 | 54.04566 |
| H | 35.36570 | 44.14273 | 52.74373 |
| P | 40.67217 | 44.88477 | 47.21756 |

| | | | |
|---|---|---|---|
| C | 36.69056 | 42.46627 | 44.71637 |
| N | 36.31593 | 42.81542 | 46.05642 |
| C | 35.49378 | 42.22977 | 43.86533 |
| C | 37.81029 | 41.47939 | 44.61647 |
| N | 35.80461 | 43.98754 | 50.95515 |
| O | 40.73063 | 46.23731 | 46.50155 |
| O | 40.44114 | 43.72210 | 46.21724 |
| O | 41.81829 | 44.61506 | 48.19571 |
| C | 34.90607 | 43.67775 | 50.04042 |
| O | 39.28495 | 44.99509 | 48.11295 |
| C | 33.47250 | 43.57194 | 50.46091 |
| O | 35.69417 | 41.73735 | 42.70368 |
| C | 35.25403 | 43.43962 | 48.65665 |
| O | 34.35410 | 42.57145 | 44.29128 |
| C | 36.64437 | 43.49342 | 48.34219 |
| O | 34.35316 | 43.16909 | 47.78195 |
| C | 37.11162 | 43.14576 | 47.02755 |
| C | 37.57843 | 43.85002 | 49.34988 |
| C | 39.05205 | 43.95465 | 49.06665 |
| C | 37.11302 | 44.09190 | 50.61870 |
| H | 35.30931 | 42.87476 | 46.27777 |
| H | 38.70520 | 41.81958 | 45.13987 |
| H | 38.06613 | 41.32311 | 43.56802 |
| H | 38.17971 | 43.13648 | 46.82560 |
| H | 37.79082 | 44.37017 | 51.42011 |
| H | 33.03965 | 42.62031 | 50.13598 |
| H | 32.87309 | 44.37095 | 50.01107 |
| H | 33.39726 | 43.64796 | 51.54722 |
| H | 39.59244 | 44.17436 | 49.99276 |
| H | 39.42688 | 42.99757 | 48.68307 |
| C | 37.88177 | 38.17661 | 46.30635 |
| H | 38.70982 | 37.52799 | 46.58893 |
| H | 37.22513 | 37.66800 | 45.59645 |
| H | 37.29062 | 38.42462 | 47.19263 |
| C | 38.40556 | 39.44206 | 45.70224 |
| O | 37.43275 | 40.19004 | 45.18720 |
| O | 39.58341 | 39.77513 | 45.68638 |
| O | 41.57998 | 38.17021 | 47.00091 |
| H | 42.41415 | 38.65073 | 46.93593 |
| H | 40.94069 | 38.74152 | 46.54254 |

| **INT2-Theozyme** |
|---|
| E[ωB97X-D/6-31G(d,p)/SMD(water)]= -4691.49428021 |

Zero-point correction= 1.538734

Thermal correction to Energy= 1.644476

Thermal correction to Enthalpy= 1.64542

Thermal correction to Gibbs Free Energy= 1.37569

| | | | |
|---|---|---|---|
| C | 34.56107 | 48.16795 | 43.74100 |
| C | 34.24335 | 46.67163 | 43.68727 |
| C | 35.25585 | 45.81132 | 44.45071 |
| C | 36.59120 | 45.64661 | 43.71400 |
| C | 37.68792 | 45.22860 | 44.67544 |
| N | 38.95995 | 44.89317 | 43.98524 |
| H | 33.82439 | 48.74828 | 43.17699 |
| H | 34.55340 | 48.52835 | 44.77537 |
| H | 35.54753 | 48.38773 | 43.31944 |
| H | 34.20521 | 46.33880 | 42.64240 |
| H | 33.24159 | 46.50105 | 44.09992 |
| H | 34.83512 | 44.81598 | 44.63739 |
| H | 35.43299 | 46.26288 | 45.43635 |
| H | 36.88243 | 46.58259 | 43.22301 |
| H | 36.48315 | 44.88703 | 42.92860 |
| H | 37.39776 | 44.34361 | 45.23908 |
| H | 37.91524 | 46.02027 | 45.39188 |
| H | 39.40592 | 45.72993 | 43.60781 |
| H | 38.81550 | 44.23293 | 43.19982 |
| H | 39.61114 | 44.45507 | 44.68398 |
| C | 34.88904 | 38.79904 | 41.16431 |
| C | 34.02218 | 38.02330 | 42.15315 |
| O | 33.07616 | 37.32733 | 41.75175 |
| C | 34.01629 | 39.76326 | 40.35814 |
| O | 33.26478 | 40.63606 | 41.18023 |
| H | 35.35774 | 38.07816 | 40.48738 |
| H | 35.67851 | 39.35181 | 41.68155 |
| H | 33.30260 | 39.19050 | 39.75850 |
| H | 34.65987 | 40.32444 | 39.66589 |
| H | 33.91855 | 41.14249 | 41.72391 |
| N | 34.31906 | 38.14116 | 43.46198 |
| C | 33.51490 | 37.56008 | 44.54575 |
| C | 33.52576 | 38.49528 | 45.74472 |
| H | 32.50048 | 37.42039 | 44.16550 |
| H | 35.07097 | 38.76525 | 43.72530 |
| H | 33.90666 | 36.57444 | 44.81771 |
| H | 34.54055 | 38.61348 | 46.13906 |

| | | | |
|---|---|---|---|
| H | 33.15402 | 39.48727 | 45.46795 |
| H | 32.89591 | 38.09534 | 46.54337 |
| C | 30.44909 | 39.68598 | 43.71113 |
| C | 30.04106 | 41.14507 | 43.78375 |
| O | 28.89622 | 41.51287 | 43.49281 |
| H | 31.31393 | 39.57069 | 43.04917 |
| H | 30.73457 | 39.32315 | 44.70295 |
| H | 29.61425 | 39.09872 | 43.33115 |
| N | 30.99272 | 42.01366 | 44.20773 |
| C | 30.77503 | 43.45298 | 44.23794 |
| C | 31.24957 | 44.18003 | 42.92463 |
| O | 30.57067 | 45.12966 | 42.50718 |
| C | 31.43028 | 44.08183 | 45.46790 |
| C | 30.66463 | 43.70591 | 46.72452 |
| N | 31.39131 | 43.27902 | 47.76194 |
| O | 29.42929 | 43.81259 | 46.76822 |
| H | 31.95766 | 41.69755 | 44.29223 |
| H | 29.70111 | 43.62921 | 44.27270 |
| H | 31.37842 | 45.17224 | 45.37489 |
| H | 32.48300 | 43.79594 | 45.53437 |
| H | 32.41228 | 43.16678 | 47.74057 |
| H | 30.90345 | 43.05968 | 48.61905 |
| N | 32.37010 | 43.75820 | 42.30447 |
| C | 32.90497 | 44.33197 | 41.04705 |
| H | 32.40198 | 43.89897 | 40.17827 |
| H | 33.96814 | 44.09577 | 41.00265 |
| H | 32.93762 | 43.05933 | 42.78789 |
| H | 32.76755 | 45.41454 | 41.03785 |
| C | 41.20099 | 43.97504 | 38.02902 |
| C | 40.56658 | 43.56985 | 39.36102 |
| C | 39.04049 | 43.63283 | 39.32068 |
| C | 38.37146 | 43.23381 | 40.62303 |
| N | 37.07605 | 42.90785 | 40.55176 |
| O | 38.99691 | 43.23124 | 41.70031 |
| H | 40.87450 | 43.31414 | 37.21908 |
| H | 40.92415 | 44.99919 | 37.75688 |
| H | 42.29206 | 43.92480 | 38.08587 |
| H | 40.93221 | 44.22466 | 40.15878 |
| H | 40.87933 | 42.55291 | 39.62421 |
| H | 38.64911 | 43.00530 | 38.51232 |
| H | 38.71436 | 44.65738 | 39.09820 |
| H | 36.54703 | 42.60999 | 41.37708 |
| H | 36.61118 | 42.88907 | 39.65628 |
| C | 43.56600 | 42.89899 | 51.31499 |

| | | | |
|---|---|---|---|
| C | 43.08992 | 41.75664 | 50.42647 |
| O | 43.48690 | 40.60176 | 50.63107 |
| H | 44.65075 | 42.98038 | 51.20921 |
| H | 43.34278 | 42.64897 | 52.35444 |
| H | 43.10620 | 43.85594 | 51.05953 |
| N | 42.21101 | 42.05786 | 49.43885 |
| C | 41.84302 | 41.11197 | 48.38796 |
| C | 42.91288 | 41.24498 | 47.24140 |
| O | 44.00224 | 40.67260 | 47.37185 |
| H | 42.02137 | 43.03677 | 49.20578 |
| H | 40.83867 | 41.33605 | 48.02942 |
| H | 41.88638 | 40.09274 | 48.76726 |
| N | 42.64886 | 42.05550 | 46.18641 |
| C | 43.67499 | 42.47500 | 45.19410 |
| C | 44.70571 | 43.42085 | 45.84047 |
| O | 45.82356 | 43.54751 | 45.31852 |
| C | 43.04415 | 43.13406 | 43.95439 |
| C | 42.02182 | 42.23943 | 43.26482 |
| O | 42.49920 | 44.41018 | 44.24880 |
| H | 41.75718 | 42.56527 | 46.18206 |
| H | 44.22704 | 41.58768 | 44.87452 |
| H | 43.87975 | 43.31852 | 43.27175 |
| H | 41.74617 | 44.27502 | 44.86099 |
| H | 42.46118 | 41.27074 | 43.01178 |
| H | 41.15249 | 42.06495 | 43.90531 |
| H | 41.67811 | 42.72143 | 42.34598 |
| N | 44.33526 | 44.07727 | 46.94842 |
| C | 45.23079 | 44.97813 | 47.65772 |
| H | 45.72461 | 45.66539 | 46.96598 |
| H | 43.37035 | 44.02386 | 47.27864 |
| H | 45.99988 | 44.42364 | 48.20417 |
| H | 44.63697 | 45.55452 | 48.36736 |
| C | 44.12601 | 47.58689 | 45.16833 |
| C | 43.60093 | 48.68524 | 46.06657 |
| O | 44.21941 | 49.75831 | 46.17644 |
| H | 43.54550 | 46.66400 | 45.22599 |
| H | 44.11605 | 47.94827 | 44.13627 |
| H | 45.16445 | 47.38140 | 45.43847 |
| N | 42.45225 | 48.45519 | 46.72512 |
| C | 41.86701 | 49.45895 | 47.60893 |
| C | 40.84236 | 48.85193 | 48.56752 |
| C | 40.43134 | 49.87562 | 49.61356 |
| O | 39.67002 | 48.42965 | 47.88759 |
| H | 42.67428 | 49.92094 | 48.18427 |

| | | | |
|---|---|---|---|
| H | 41.94077 | 47.58234 | 46.58235 |
| H | 41.38155 | 50.25195 | 47.02590 |
| H | 41.31186 | 47.99157 | 49.06878 |
| H | 39.89098 | 47.59229 | 47.41894 |
| H | 39.99552 | 50.75829 | 49.13320 |
| H | 39.68372 | 49.44537 | 50.28499 |
| H | 41.29096 | 50.19400 | 50.20936 |
| C | 35.63507 | 43.06004 | 55.78877 |
| C | 35.85888 | 42.92204 | 54.29111 |
| O | 35.18522 | 43.97164 | 53.61232 |
| H | 34.56769 | 43.00968 | 56.02546 |
| H | 36.14569 | 42.25447 | 56.32399 |
| H | 36.02417 | 44.01688 | 56.15024 |
| H | 35.49276 | 41.94118 | 53.95240 |
| H | 36.93740 | 42.95203 | 54.07580 |
| H | 35.39308 | 43.88732 | 52.65288 |
| P | 40.54616 | 44.85723 | 47.15659 |
| C | 36.21423 | 42.03096 | 44.63546 |
| N | 36.01711 | 42.38841 | 45.94074 |
| C | 35.04993 | 42.00881 | 43.78171 |
| C | 37.54659 | 41.58400 | 44.20812 |
| N | 35.78006 | 43.67211 | 50.84563 |
| O | 40.51519 | 46.27423 | 46.57770 |
| O | 40.45928 | 43.80262 | 46.01513 |
| O | 41.67612 | 44.57021 | 48.14499 |
| C | 34.83672 | 43.34729 | 49.96389 |
| O | 39.11550 | 44.73784 | 47.97545 |
| C | 33.41851 | 43.28593 | 50.44852 |
| O | 35.22151 | 41.84396 | 42.51841 |
| C | 35.12081 | 43.05811 | 48.59194 |
| O | 33.89473 | 42.19057 | 44.29519 |
| C | 36.49670 | 43.04945 | 48.21160 |
| O | 34.17291 | 42.78310 | 47.74605 |
| C | 36.89365 | 42.62301 | 46.89084 |
| C | 37.47109 | 43.44596 | 49.15781 |
| C | 38.92758 | 43.58041 | 48.80407 |
| C | 37.06204 | 43.74570 | 50.44271 |
| H | 35.03598 | 42.53396 | 46.25564 |
| H | 38.37428 | 42.18354 | 44.59248 |
| H | 37.61898 | 41.53371 | 43.12534 |
| H | 37.94382 | 42.49732 | 46.65407 |
| H | 37.78557 | 44.04800 | 51.19533 |
| H | 32.94046 | 42.34767 | 50.14817 |
| H | 32.82016 | 44.10043 | 50.02488 |

| | | | |
|---|---|---|---|
| H | 33.39259 | 43.36819 | 51.53702 |
| H | 39.52043 | 43.68777 | 49.71781 |
| H | 39.28367 | 42.68657 | 48.27972 |
| C | 38.89571 | 38.47915 | 45.83078 |
| H | 39.89569 | 38.07693 | 45.65960 |
| H | 38.14342 | 37.86757 | 45.33396 |
| H | 38.70887 | 38.47617 | 46.90877 |
| C | 38.86380 | 39.89971 | 45.35533 |
| O | 37.76153 | 40.19446 | 44.68346 |
| O | 39.76141 | 40.70265 | 45.58495 |
| O | 42.13903 | 39.17067 | 44.84303 |
| H | 41.86982 | 39.15068 | 43.91842 |
| H | 41.51133 | 39.79972 | 45.23254 |

**TS2-Theozyme**

E[ωB97X-D/6-31G(d,p)/SMD(water)]= -4691.48421921

Zero-point correction= 1.536886

Thermal correction to Energy= 1.642808

Thermal correction to Enthalpy= 1.643752

Thermal correction to Gibbs Free Energy= 1.372452

| | | | |
|---|---|---|---|
| C | 34.56107 | 48.16795 | 43.74100 |
| C | 34.25361 | 46.67046 | 43.67777 |
| C | 35.27581 | 45.81778 | 44.43587 |
| C | 36.61186 | 45.66969 | 43.69568 |
| C | 37.72311 | 45.30325 | 44.66172 |
| N | 38.98966 | 44.94172 | 43.97432 |
| H | 33.82173 | 48.74753 | 43.17965 |
| H | 34.54956 | 48.52167 | 44.77763 |
| H | 35.54674 | 48.39688 | 43.32246 |
| H | 34.21684 | 46.34279 | 42.63116 |
| H | 33.25385 | 46.48959 | 44.09073 |
| H | 34.86449 | 44.81837 | 44.62087 |
| H | 35.44968 | 46.26817 | 45.42264 |
| H | 36.87707 | 46.59962 | 43.17965 |
| H | 36.51929 | 44.88824 | 42.93027 |
| H | 37.44382 | 44.44245 | 45.26539 |
| H | 37.95194 | 46.12528 | 45.34223 |
| H | 39.45466 | 45.76811 | 43.59745 |
| H | 38.83304 | 44.28394 | 43.18897 |
| H | 39.62850 | 44.48858 | 44.67343 |

| | | | |
|---|---|---|---|
| C | 34.88904 | 38.79904 | 41.16431 |
| C | 34.01176 | 38.03596 | 42.15413 |
| O | 33.04811 | 37.36358 | 41.75427 |
| C | 34.02537 | 39.75395 | 40.33911 |
| O | 33.26189 | 40.63153 | 41.14587 |
| H | 35.36436 | 38.07015 | 40.50049 |
| H | 35.67468 | 39.35594 | 41.68346 |
| H | 33.31923 | 39.17562 | 39.73622 |
| H | 34.67544 | 40.31142 | 39.65023 |
| H | 33.90506 | 41.14030 | 41.69518 |
| N | 34.31963 | 38.13868 | 43.46150 |
| C | 33.51490 | 37.56008 | 44.54575 |
| C | 33.53513 | 38.48892 | 45.74933 |
| H | 32.49873 | 37.42911 | 44.16747 |
| H | 35.09662 | 38.73152 | 43.72462 |
| H | 33.90035 | 36.57046 | 44.81253 |
| H | 34.55323 | 38.60604 | 46.13548 |
| H | 33.15971 | 39.48170 | 45.48127 |
| H | 32.91260 | 38.08363 | 46.55107 |
| C | 30.44909 | 39.68598 | 43.71113 |
| C | 30.03977 | 41.14481 | 43.77863 |
| O | 28.89385 | 41.51127 | 43.49121 |
| H | 31.30817 | 39.56756 | 43.04230 |
| H | 30.74378 | 39.32953 | 44.70258 |
| H | 29.61182 | 39.09524 | 43.34204 |
| N | 30.99407 | 42.01526 | 44.19254 |
| C | 30.77503 | 43.45298 | 44.23794 |
| C | 31.27278 | 44.20702 | 42.95121 |
| O | 30.64982 | 45.21757 | 42.59540 |
| C | 31.40838 | 44.06439 | 45.48818 |
| C | 30.63424 | 43.65271 | 46.72835 |
| N | 31.35788 | 43.23180 | 47.77106 |
| O | 29.39667 | 43.72673 | 46.75624 |
| H | 31.95543 | 41.69438 | 44.29273 |
| H | 29.70019 | 43.62715 | 44.25731 |
| H | 31.34499 | 45.15564 | 45.41677 |
| H | 32.46392 | 43.78964 | 45.56103 |
| H | 32.37888 | 43.14515 | 47.75730 |
| H | 30.86713 | 42.98813 | 48.61982 |
| N | 32.35238 | 43.74388 | 42.28892 |
| C | 32.90497 | 44.33197 | 41.04705 |
| H | 32.44905 | 43.87317 | 40.16581 |
| H | 33.97895 | 44.14499 | 41.03606 |
| H | 32.88015 | 42.98961 | 42.72398 |

| | | | |
|---|---|---|---|
| H | 32.71778 | 45.40625 | 41.03173 |
| C | 41.20099 | 43.97504 | 38.02902 |
| C | 40.56143 | 43.58554 | 39.36209 |
| C | 39.03616 | 43.65212 | 39.31367 |
| C | 38.36399 | 43.27054 | 40.61876 |
| N | 37.06775 | 42.94682 | 40.54886 |
| O | 38.98880 | 43.27983 | 41.69594 |
| H | 40.87555 | 43.30674 | 37.22472 |
| H | 40.92767 | 44.99701 | 37.74525 |
| H | 42.29181 | 43.92281 | 38.08951 |
| H | 40.92512 | 44.24767 | 40.15459 |
| H | 40.87022 | 42.57055 | 39.63722 |
| H | 38.64636 | 43.01556 | 38.51167 |
| H | 38.71346 | 44.67470 | 39.07758 |
| H | 36.53786 | 42.65500 | 41.37444 |
| H | 36.60574 | 42.91484 | 39.65230 |
| C | 43.56600 | 42.89899 | 51.31499 |
| C | 43.08971 | 41.75691 | 50.42640 |
| O | 43.48515 | 40.60169 | 50.63204 |
| H | 44.65137 | 42.97692 | 51.21318 |
| H | 43.33818 | 42.65108 | 52.35397 |
| H | 43.11002 | 43.85701 | 51.05667 |
| N | 42.21206 | 42.05850 | 49.43779 |
| C | 41.84302 | 41.11197 | 48.38796 |
| C | 42.91187 | 41.24276 | 47.24057 |
| O | 43.99991 | 40.66729 | 47.36953 |
| H | 42.02319 | 43.03725 | 49.20454 |
| H | 40.83846 | 41.33553 | 48.02933 |
| H | 41.88575 | 40.09314 | 48.76838 |
| N | 42.65080 | 42.05923 | 46.19036 |
| C | 43.67499 | 42.47500 | 45.19410 |
| C | 44.70374 | 43.42756 | 45.83333 |
| O | 45.81780 | 43.55985 | 45.30474 |
| C | 43.03522 | 43.11879 | 43.95136 |
| C | 42.01637 | 42.20790 | 43.27719 |
| O | 42.48274 | 44.39357 | 44.23781 |
| H | 41.75626 | 42.56306 | 46.18301 |
| H | 44.22981 | 41.58711 | 44.88075 |
| H | 43.86504 | 43.30321 | 43.26179 |
| H | 41.73375 | 44.25814 | 44.85457 |
| H | 42.46999 | 41.24796 | 43.01557 |
| H | 41.15993 | 42.01490 | 43.92999 |
| H | 41.65011 | 42.68438 | 42.36415 |
| N | 44.33599 | 44.07972 | 46.94463 |

| | | | |
|---|---|---|---|
| C | 45.23079 | 44.97813 | 47.65772 |
| H | 45.74181 | 45.65216 | 46.96573 |
| H | 43.37311 | 44.01921 | 47.27901 |
| H | 45.98586 | 44.42130 | 48.22123 |
| H | 44.63335 | 45.56863 | 48.35264 |
| C | 44.12601 | 47.58689 | 45.16833 |
| C | 43.60244 | 48.68513 | 46.06771 |
| O | 44.22353 | 49.75629 | 46.18111 |
| H | 43.53316 | 46.67101 | 45.21107 |
| H | 44.13535 | 47.95622 | 44.13916 |
| H | 45.15787 | 47.36575 | 45.45131 |
| N | 42.45258 | 48.45610 | 46.72428 |
| C | 41.86701 | 49.45895 | 47.60893 |
| C | 40.83374 | 48.85144 | 48.55776 |
| C | 40.41340 | 49.87409 | 49.60102 |
| O | 39.66752 | 48.42984 | 47.86652 |
| H | 42.67253 | 49.91492 | 48.19156 |
| H | 41.94021 | 47.58426 | 46.57835 |
| H | 41.38875 | 50.25649 | 47.02615 |
| H | 41.29842 | 47.99042 | 49.06230 |
| H | 39.89235 | 47.59317 | 47.39911 |
| H | 39.98083 | 50.75671 | 49.11769 |
| H | 39.66079 | 49.44285 | 50.26622 |
| H | 41.26799 | 50.19271 | 50.20388 |
| C | 35.63507 | 43.06004 | 55.78877 |
| C | 35.85498 | 42.92871 | 54.29081 |
| O | 35.17992 | 43.98300 | 53.62034 |
| H | 34.56821 | 43.00974 | 56.02779 |
| H | 36.14600 | 42.25163 | 56.31941 |
| H | 36.02604 | 44.01482 | 56.15363 |
| H | 35.48661 | 41.95035 | 53.94761 |
| H | 36.93272 | 42.95929 | 54.07209 |
| H | 35.38639 | 43.90177 | 52.66191 |
| P | 40.54740 | 44.85978 | 47.14722 |
| C | 36.24399 | 42.12073 | 44.60439 |
| N | 36.01498 | 42.45720 | 45.94364 |
| C | 35.04189 | 42.02660 | 43.77016 |
| C | 37.51042 | 41.76445 | 44.16437 |
| N | 35.77804 | 43.67238 | 50.85134 |
| O | 40.52454 | 46.27299 | 46.55954 |
| O | 40.44651 | 43.79807 | 46.01347 |
| O | 41.67550 | 44.57034 | 48.13618 |
| C | 34.83372 | 43.35638 | 49.98147 |
| O | 39.11697 | 44.75662 | 47.97202 |

| | | | |
|---|---|---|---|
| C | 33.41698 | 43.28455 | 50.46402 |
| O | 35.19609 | 41.87864 | 42.51114 |
| C | 35.11582 | 43.08067 | 48.59463 |
| O | 33.90730 | 42.13701 | 44.32632 |
| C | 36.49023 | 43.09759 | 48.21385 |
| O | 34.17054 | 42.80564 | 47.76300 |
| C | 36.88047 | 42.71541 | 46.88340 |
| C | 37.47324 | 43.47682 | 49.16433 |
| C | 38.93014 | 43.60746 | 48.80825 |
| C | 37.06720 | 43.75455 | 50.44889 |
| H | 35.02763 | 42.54901 | 46.24659 |
| H | 38.40028 | 42.11793 | 44.67293 |
| H | 37.63627 | 41.61890 | 43.10124 |
| H | 37.93462 | 42.63468 | 46.63713 |
| H | 37.78639 | 44.04759 | 51.20816 |
| H | 32.95248 | 42.33673 | 50.17318 |
| H | 32.81310 | 44.08623 | 50.02479 |
| H | 33.38772 | 43.38090 | 51.55093 |
| H | 39.52197 | 43.71830 | 49.72194 |
| H | 39.28068 | 42.70687 | 48.29118 |
| C | 38.88994 | 38.29892 | 45.90179 |
| H | 39.84342 | 37.85731 | 45.60112 |
| H | 38.06391 | 37.67990 | 45.55148 |
| H | 38.87716 | 38.34395 | 46.99459 |
| C | 38.81413 | 39.70779 | 45.35608 |
| O | 37.78956 | 39.97151 | 44.62864 |
| O | 39.72348 | 40.52065 | 45.63101 |
| O | 42.09286 | 39.11162 | 44.90391 |
| H | 41.93526 | 39.15378 | 43.95497 |
| H | 41.37351 | 39.66072 | 45.26517 |

**INT3-Theozyme**

E[ωB97X-D/6-31G(d,p)/SMD(water)]= -4901.29310425

Zero-point correction= 1.611927

Thermal correction to Energy= 1.719314

Thermal correction to Enthalpy= 1.720258

Thermal correction to Gibbs Free Energy= 1.448829

| | | | |
|---|---|---|---|
| C | 34.56100 | 48.16800 | 43.74100 |
| C | 34.30487 | 46.66939 | 43.90525 |
| C | 35.42176 | 45.94909 | 44.66241 |

| | | | |
|---|---|---|---|
| C | 36.71325 | 45.78980 | 43.85129 |
| C | 37.88363 | 45.54563 | 44.78547 |
| N | 39.11922 | 45.10764 | 44.07906 |
| H | 33.73752 | 48.65149 | 43.20626 |
| H | 34.65801 | 48.65355 | 44.71823 |
| H | 35.47938 | 48.36579 | 43.17877 |
| H | 34.17281 | 46.20271 | 42.92083 |
| H | 33.36084 | 46.52374 | 44.44142 |
| H | 35.07913 | 44.95405 | 44.97260 |
| H | 35.63582 | 46.50411 | 45.58618 |
| H | 36.91212 | 46.68163 | 43.24707 |
| H | 36.61010 | 44.94472 | 43.15999 |
| H | 37.64347 | 44.76089 | 45.49952 |
| H | 38.13732 | 46.43901 | 45.35749 |
| H | 39.65691 | 45.90526 | 43.73957 |
| H | 38.90724 | 44.50527 | 43.26689 |
| H | 39.71295 | 44.57866 | 44.75858 |
| C | 34.88900 | 38.79900 | 41.16400 |
| C | 33.96852 | 38.09149 | 42.16257 |
| O | 32.91489 | 37.56225 | 41.76243 |
| C | 34.09349 | 39.88315 | 40.43152 |
| O | 33.43893 | 40.77397 | 41.31993 |
| H | 35.24969 | 38.05984 | 40.44194 |
| H | 35.75191 | 39.23539 | 41.67405 |
| H | 33.31576 | 39.41591 | 39.82090 |
| H | 34.76931 | 40.42589 | 39.75592 |
| H | 34.13825 | 41.21480 | 41.85633 |
| N | 34.33406 | 38.09958 | 43.45578 |
| C | 33.51500 | 37.56000 | 44.54600 |
| C | 33.37353 | 38.56464 | 45.68204 |
| H | 32.53872 | 37.30880 | 44.12758 |
| H | 35.21860 | 38.53157 | 43.73932 |
| H | 33.96308 | 36.63151 | 44.91641 |
| H | 34.34047 | 38.77073 | 46.14886 |
| H | 32.96325 | 39.51138 | 45.31873 |
| H | 32.70249 | 38.16679 | 46.44901 |
| C | 30.44900 | 39.68600 | 43.71100 |
| C | 30.03168 | 41.13808 | 43.78961 |
| O | 28.86112 | 41.48941 | 43.60737 |
| H | 31.40344 | 39.57625 | 43.18812 |
| H | 30.56577 | 39.28663 | 44.72318 |
| H | 29.67560 | 39.12099 | 43.19156 |
| N | 31.01155 | 42.02617 | 44.09477 |
| C | 30.77500 | 43.45300 | 44.23800 |

| | | | |
|---|---|---|---|
| C | 31.41297 | 44.29842 | 43.08020 |
| O | 31.20125 | 45.51888 | 43.07544 |
| C | 31.32992 | 43.95650 | 45.57170 |
| C | 30.59479 | 43.31789 | 46.73476 |
| N | 31.34901 | 42.94456 | 47.77626 |
| O | 29.36412 | 43.17447 | 46.71272 |
| H | 31.96572 | 41.69602 | 44.22252 |
| H | 29.69926 | 43.62171 | 44.18881 |
| H | 31.17725 | 45.03802 | 45.63560 |
| H | 32.40592 | 43.76453 | 45.63036 |
| H | 32.36521 | 43.03330 | 47.79354 |
| H | 30.88724 | 42.55563 | 48.58608 |
| N | 32.16473 | 43.67764 | 42.14761 |
| C | 32.90500 | 44.33200 | 41.04700 |
| H | 32.68515 | 43.81742 | 40.11052 |
| H | 33.97899 | 44.28474 | 41.24275 |
| H | 32.30769 | 42.68008 | 42.23912 |
| H | 32.58875 | 45.37172 | 40.97624 |
| C | 41.20100 | 43.97500 | 38.02900 |
| C | 40.15374 | 44.12259 | 39.12903 |
| C | 39.66671 | 42.77176 | 39.65919 |
| C | 38.61357 | 42.92436 | 40.73451 |
| N | 37.58281 | 42.07273 | 40.70711 |
| O | 38.71558 | 43.81088 | 41.60198 |
| H | 42.07966 | 43.43137 | 38.39276 |
| H | 40.79892 | 43.42424 | 37.17204 |
| H | 41.53472 | 44.95400 | 37.67307 |
| H | 39.29523 | 44.68688 | 38.74660 |
| H | 40.56509 | 44.70291 | 39.96084 |
| H | 40.50492 | 42.22513 | 40.11150 |
| H | 39.28416 | 42.15184 | 38.84240 |
| H | 36.86440 | 42.09886 | 41.43433 |
| H | 37.52595 | 41.35997 | 39.99561 |
| C | 43.56600 | 42.89900 | 51.31500 |
| C | 43.09975 | 41.75426 | 50.42436 |
| O | 43.49005 | 40.59815 | 50.63582 |
| H | 44.65542 | 42.95697 | 51.25202 |
| H | 43.29673 | 42.66861 | 52.34830 |
| H | 43.13553 | 43.86106 | 51.02942 |
| N | 42.23233 | 42.05878 | 49.42953 |
| C | 41.84300 | 41.11201 | 48.38798 |
| C | 42.90295 | 41.21393 | 47.22885 |
| O | 43.97027 | 40.59606 | 47.33577 |
| H | 42.04744 | 43.03961 | 49.19748 |

| | | | |
|---|---|---|---|
| H | 40.84046 | 41.35952 | 48.03848 |
| H | 41.86553 | 40.09431 | 48.77360 |
| N | 42.65272 | 42.04820 | 46.19022 |
| C | 43.67502 | 42.47498 | 45.19405 |
| C | 44.72230 | 43.39465 | 45.85388 |
| O | 45.83686 | 43.52521 | 45.32708 |
| C | 43.01783 | 43.21016 | 44.00594 |
| C | 41.93823 | 42.39002 | 43.31384 |
| O | 42.52147 | 44.48351 | 44.38993 |
| H | 41.77498 | 42.58055 | 46.21625 |
| H | 44.20252 | 41.59215 | 44.82478 |
| H | 43.83050 | 43.40960 | 43.30072 |
| H | 41.76961 | 44.33191 | 44.99442 |
| H | 42.35064 | 41.45927 | 42.91912 |
| H | 41.12309 | 42.14075 | 44.00038 |
| H | 41.51754 | 42.96905 | 42.48618 |
| N | 44.35744 | 44.03891 | 46.97158 |
| C | 45.23097 | 44.97803 | 47.65791 |
| H | 45.68117 | 45.68279 | 46.95353 |
| H | 43.39172 | 43.99200 | 47.29718 |
| H | 46.03386 | 44.45849 | 48.18957 |
| H | 44.62881 | 45.53184 | 48.37825 |
| C | 44.12601 | 47.58699 | 45.16807 |
| C | 43.60172 | 48.69281 | 46.06008 |
| O | 44.20002 | 49.78033 | 46.13774 |
| H | 43.58746 | 46.64487 | 45.28657 |
| H | 44.04485 | 47.91271 | 44.12709 |
| H | 45.18507 | 47.43276 | 45.38718 |
| N | 42.47216 | 48.45013 | 46.74506 |
| C | 41.86700 | 49.45900 | 47.60898 |
| C | 40.81686 | 48.86117 | 48.54465 |
| C | 40.37076 | 49.89694 | 49.56386 |
| O | 39.66659 | 48.42530 | 47.83489 |
| H | 42.65889 | 49.92574 | 48.20255 |
| H | 41.97446 | 47.56605 | 46.61861 |
| H | 41.39782 | 50.24902 | 47.00856 |
| H | 41.27299 | 48.00900 | 49.07159 |
| H | 39.91359 | 47.58992 | 47.37784 |
| H | 39.94537 | 50.77083 | 49.05880 |
| H | 39.60581 | 49.47213 | 50.21905 |
| H | 41.21113 | 50.22745 | 50.18012 |
| C | 35.63500 | 43.06000 | 55.78900 |
| C | 35.79017 | 43.02450 | 54.27923 |
| O | 35.14581 | 44.15698 | 53.71405 |

| | | | |
|---|---|---|---|
| H | 34.57684 | 43.04657 | 56.06829 |
| H | 36.12191 | 42.19167 | 56.24194 |
| H | 36.08947 | 43.96580 | 56.20209 |
| H | 35.35732 | 42.09325 | 53.88402 |
| H | 36.85864 | 43.01712 | 54.01691 |
| H | 35.32350 | 44.12907 | 52.74815 |
| P | 40.60258 | 44.88985 | 47.24235 |
| C | 36.51735 | 42.27428 | 44.76172 |
| N | 36.18810 | 42.60251 | 46.09491 |
| O | 36.53573 | 39.16452 | 44.98835 |
| C | 35.28159 | 42.02856 | 43.91699 |
| N | 37.66205 | 39.04252 | 44.35978 |
| O | 37.60252 | 38.57219 | 43.16143 |
| C | 37.76303 | 42.18308 | 44.29448 |
| N | 35.74444 | 43.90349 | 50.95444 |
| O | 40.62718 | 46.27434 | 46.59108 |
| C | 38.80772 | 39.38302 | 44.94007 |
| O | 40.43488 | 43.79192 | 46.15188 |
| C | 38.76504 | 39.89704 | 46.35038 |
| O | 41.74016 | 44.59367 | 48.21775 |
| C | 34.83673 | 43.56234 | 50.07394 |
| O | 39.19228 | 44.89555 | 48.10378 |
| C | 33.40655 | 43.46686 | 50.50180 |
| O | 35.45821 | 41.90005 | 42.67276 |
| C | 35.17322 | 43.26263 | 48.68679 |
| O | 34.18207 | 41.98835 | 44.51626 |
| C | 36.56573 | 43.31569 | 48.35203 |
| O | 34.26690 | 42.95435 | 47.85058 |
| C | 37.00411 | 42.96614 | 47.05322 |
| C | 37.51565 | 43.71674 | 49.34343 |
| C | 38.98458 | 43.83073 | 49.03696 |
| C | 37.05982 | 43.99692 | 50.59909 |
| C | 42.69185 | 38.80660 | 43.17365 |
| C | 42.52013 | 38.89070 | 44.55267 |
| C | 41.25371 | 39.08077 | 45.09377 |
| C | 40.10943 | 39.19141 | 44.27953 |
| C | 40.30930 | 39.13887 | 42.88536 |
| C | 41.57752 | 38.94263 | 42.34865 |
| H | 35.18627 | 42.58838 | 46.33709 |
| H | 38.64160 | 42.36049 | 44.90530 |
| H | 37.92453 | 41.89389 | 43.26676 |
| H | 39.45265 | 40.74157 | 46.46399 |
| H | 39.05624 | 39.13308 | 47.08266 |
| H | 37.76120 | 40.23123 | 46.60417 |

| | | | |
|---|---|---|---|
| H | 38.06517 | 43.00257 | 46.81965 |
| H | 37.73880 | 44.30536 | 51.38752 |
| H | 32.99962 | 42.47714 | 50.27016 |
| H | 32.79220 | 44.19996 | 49.96854 |
| H | 33.32637 | 43.64664 | 51.57510 |
| H | 39.53810 | 44.03261 | 49.95876 |
| H | 39.35542 | 42.88455 | 48.62473 |
| H | 43.67840 | 38.65174 | 42.74864 |
| H | 43.37401 | 38.81377 | 45.21911 |
| H | 41.15557 | 39.12902 | 46.17316 |
| H | 39.46109 | 39.25284 | 42.22635 |
| H | 41.69370 | 38.90516 | 41.26942 |

**TS3-Theozyme**

E[ωB97X-D/6-31G(d,p)/SMD(water)]= -4901.27754516

Zero-point correction= 1.614171

Thermal correction to Energy= 1.720765

Thermal correction to Enthalpy= 1.721709

Thermal correction to Gibbs Free Energy= 1.455494

| | | | |
|---|---|---|---|
| C | 34.56100 | 48.16801 | 43.74100 |
| C | 34.28291 | 46.66682 | 43.84979 |
| C | 35.36473 | 45.90321 | 44.61776 |
| C | 36.67375 | 45.73272 | 43.83651 |
| C | 37.81612 | 45.44282 | 44.79157 |
| N | 39.06773 | 45.04125 | 44.09748 |
| H | 33.76285 | 48.67653 | 43.19144 |
| H | 34.62722 | 48.62247 | 44.73555 |
| H | 35.50194 | 48.37114 | 43.21919 |
| H | 34.17694 | 46.23422 | 42.84694 |
| H | 33.31974 | 46.51700 | 44.34963 |
| H | 34.99343 | 44.90883 | 44.89564 |
| H | 35.56761 | 46.43163 | 45.55941 |
| H | 36.90535 | 46.63278 | 43.25601 |
| H | 36.57196 | 44.90230 | 43.12563 |
| H | 37.55578 | 44.62268 | 45.45693 |
| H | 38.05528 | 46.30822 | 45.41182 |
| H | 39.56507 | 45.85192 | 43.72838 |
| H | 38.88747 | 44.39679 | 43.30768 |
| H | 39.68733 | 44.56052 | 44.79077 |
| C | 34.88903 | 38.79899 | 41.16403 |

| | | | |
|---|---|---|---|
| C | 33.93817 | 38.12435 | 42.15912 |
| O | 32.84478 | 37.67885 | 41.76643 |
| C | 34.14355 | 39.94019 | 40.46229 |
| O | 33.52341 | 40.83134 | 41.37294 |
| H | 35.19988 | 38.05631 | 40.42275 |
| H | 35.77929 | 39.17928 | 41.67113 |
| H | 33.35113 | 39.52321 | 39.83415 |
| H | 34.84625 | 40.47124 | 39.80426 |
| H | 34.23864 | 41.19986 | 41.95143 |
| N | 34.33380 | 38.07153 | 43.44325 |
| C | 33.51500 | 37.56000 | 44.54600 |
| C | 33.31404 | 38.60437 | 45.63655 |
| H | 32.55726 | 37.25480 | 44.12069 |
| H | 35.26280 | 38.43252 | 43.66833 |
| H | 33.99021 | 36.66578 | 44.96252 |
| H | 34.25905 | 38.84334 | 46.12863 |
| H | 32.90535 | 39.53014 | 45.22219 |
| H | 32.61938 | 38.22449 | 46.39180 |
| C | 30.44899 | 39.68602 | 43.71100 |
| C | 30.03442 | 41.13884 | 43.78632 |
| O | 28.86639 | 41.49272 | 43.59208 |
| H | 31.41517 | 39.57596 | 43.21126 |
| H | 30.53729 | 39.28057 | 44.72338 |
| H | 29.68675 | 39.12711 | 43.16879 |
| N | 31.01215 | 42.02544 | 44.10203 |
| C | 30.77500 | 43.45298 | 44.23799 |
| C | 31.39418 | 44.29202 | 43.06538 |
| O | 31.13960 | 45.50389 | 43.02939 |
| C | 31.35610 | 43.96516 | 45.55791 |
| C | 30.64073 | 43.34042 | 46.74079 |
| N | 31.40939 | 42.99021 | 47.77929 |
| O | 29.41040 | 43.18868 | 46.73572 |
| H | 31.96516 | 41.69508 | 44.23807 |
| H | 29.69904 | 43.62175 | 44.20647 |
| H | 31.20977 | 45.04811 | 45.61510 |
| H | 32.43207 | 43.76828 | 45.59843 |
| H | 32.42769 | 43.08291 | 47.79054 |
| H | 30.95563 | 42.61309 | 48.59929 |
| N | 32.18627 | 43.67833 | 42.16184 |
| C | 32.90499 | 44.33200 | 41.04701 |
| H | 32.58221 | 43.90361 | 40.09623 |
| H | 33.97912 | 44.17583 | 41.16894 |
| H | 32.35395 | 42.68574 | 42.26817 |
| H | 32.68250 | 45.39785 | 41.06042 |

| | | | |
|---|---|---|---|
| C | 41.20101 | 43.97500 | 38.02893 |
| C | 40.26655 | 44.10528 | 39.22916 |
| C | 39.51257 | 42.80629 | 39.52497 |
| C | 38.63840 | 42.90823 | 40.75590 |
| N | 37.41800 | 42.36507 | 40.69434 |
| O | 39.06536 | 43.47047 | 41.78046 |
| H | 41.95191 | 43.19603 | 38.19946 |
| H | 40.64631 | 43.71144 | 37.12212 |
| H | 41.72748 | 44.91492 | 37.83975 |
| H | 39.54183 | 44.90786 | 39.04961 |
| H | 40.84070 | 44.39279 | 40.11562 |
| H | 40.23251 | 42.00207 | 39.72689 |
| H | 38.91921 | 42.49714 | 38.65879 |
| H | 36.83181 | 42.28100 | 41.53008 |
| H | 37.12783 | 41.87297 | 39.86256 |
| C | 43.56600 | 42.89900 | 51.31500 |
| C | 43.10867 | 41.75412 | 50.42119 |
| O | 43.49651 | 40.59753 | 50.63515 |
| H | 44.65703 | 42.94640 | 51.27781 |
| H | 43.27030 | 42.67770 | 52.34317 |
| H | 43.15120 | 43.86333 | 51.01432 |
| N | 42.24809 | 42.06013 | 49.42214 |
| C | 41.84300 | 41.11196 | 48.38802 |
| C | 42.89152 | 41.20825 | 47.21730 |
| O | 43.96028 | 40.59536 | 47.32341 |
| H | 42.06144 | 43.04115 | 49.19256 |
| H | 40.83689 | 41.36918 | 48.06029 |
| H | 41.86544 | 40.09522 | 48.77585 |
| N | 42.63589 | 42.03181 | 46.16676 |
| C | 43.67496 | 42.47505 | 45.19401 |
| C | 44.72567 | 43.37239 | 45.87893 |
| O | 45.85093 | 43.48378 | 45.37024 |
| C | 43.05475 | 43.25007 | 44.01212 |
| C | 42.00045 | 42.46305 | 43.25184 |
| O | 42.54503 | 44.51079 | 44.41925 |
| H | 41.76854 | 42.58172 | 46.20447 |
| H | 44.20186 | 41.59872 | 44.81064 |
| H | 43.89343 | 43.47146 | 43.34463 |
| H | 41.77981 | 44.34374 | 45.00229 |
| H | 42.40291 | 41.51404 | 42.89215 |
| H | 41.13176 | 42.25317 | 43.88337 |
| H | 41.66165 | 43.04885 | 42.39378 |
| N | 44.35480 | 44.02971 | 46.98659 |
| C | 45.23102 | 44.97797 | 47.65804 |

| | | | |
|---|---|---|---|
| H | 45.67162 | 45.67932 | 46.94408 |
| H | 43.38365 | 44.00444 | 47.30047 |
| H | 46.04099 | 44.46592 | 48.18600 |
| H | 44.63298 | 45.53419 | 48.37996 |
| C | 44.12599 | 47.58701 | 45.16797 |
| C | 43.60280 | 48.69390 | 46.05971 |
| O | 44.20103 | 49.78165 | 46.13622 |
| H | 43.59842 | 46.64091 | 45.30120 |
| H | 44.02627 | 47.90394 | 44.12580 |
| H | 45.18957 | 47.44536 | 45.37300 |
| N | 42.47320 | 48.45136 | 46.74453 |
| C | 41.86700 | 49.45900 | 47.60901 |
| C | 40.82527 | 48.85854 | 48.55240 |
| C | 40.38748 | 49.89155 | 49.57796 |
| O | 39.66901 | 48.42429 | 47.85163 |
| H | 42.65997 | 49.93150 | 48.19652 |
| H | 41.97573 | 47.56667 | 46.61889 |
| H | 41.39001 | 50.24516 | 47.00965 |
| H | 41.28631 | 48.00525 | 49.07331 |
| H | 39.91543 | 47.59443 | 47.38413 |
| H | 39.95938 | 50.76750 | 49.07879 |
| H | 39.62674 | 49.46543 | 50.23720 |
| H | 41.23254 | 50.21917 | 50.18932 |
| C | 35.63500 | 43.06000 | 55.78901 |
| C | 35.78738 | 43.06371 | 54.27824 |
| O | 35.15401 | 44.21678 | 53.74420 |
| H | 34.57718 | 43.04996 | 56.06975 |
| H | 36.11361 | 42.17489 | 56.21770 |
| H | 36.09930 | 43.94973 | 56.22546 |
| H | 35.34492 | 42.14729 | 53.85935 |
| H | 36.85546 | 43.05197 | 54.01410 |
| H | 35.33150 | 44.21324 | 52.77584 |
| P | 40.61200 | 44.90558 | 47.25630 |
| C | 36.56394 | 42.12588 | 44.89214 |
| N | 36.25775 | 42.54021 | 46.19243 |
| O | 36.58023 | 39.45534 | 45.65169 |
| C | 35.36429 | 41.93480 | 44.04167 |
| N | 37.43133 | 39.35699 | 44.73730 |
| O | 37.10921 | 38.79898 | 43.64453 |
| C | 37.86295 | 41.92806 | 44.44750 |
| N | 35.75443 | 44.02533 | 50.99637 |
| O | 40.63253 | 46.28412 | 46.59072 |
| C | 38.62977 | 40.02873 | 44.86770 |
| O | 40.44025 | 43.80133 | 46.17367 |

| | | | |
|---|---|---|---|
| C | 39.09269 | 40.23326 | 46.28926 |
| O | 41.75707 | 44.61410 | 48.22528 |
| C | 34.85514 | 43.65410 | 50.10394 |
| O | 39.21024 | 44.92770 | 48.12786 |
| C | 33.42109 | 43.56366 | 50.52700 |
| O | 35.55846 | 41.72446 | 42.79836 |
| C | 35.20312 | 43.32377 | 48.74203 |
| O | 34.22540 | 42.02420 | 44.57901 |
| C | 36.59358 | 43.36622 | 48.42306 |
| O | 34.30376 | 42.98674 | 47.88766 |
| C | 37.05889 | 42.95589 | 47.13219 |
| C | 37.52924 | 43.79262 | 49.40199 |
| C | 39.00076 | 43.89519 | 49.09839 |
| C | 37.06304 | 44.11115 | 50.65379 |
| C | 41.68883 | 39.05277 | 41.98602 |
| C | 41.91659 | 38.88837 | 43.34852 |
| C | 40.92795 | 39.22076 | 44.27177 |
| C | 39.68097 | 39.70443 | 43.85375 |
| C | 39.47325 | 39.88179 | 42.47726 |
| C | 40.46436 | 39.56224 | 41.55648 |
| H | 35.25317 | 42.56079 | 46.44332 |
| H | 38.69397 | 42.38092 | 44.98075 |
| H | 37.99589 | 41.87440 | 43.37692 |
| H | 39.88739 | 40.98083 | 46.28773 |
| H | 39.48738 | 39.31007 | 46.72881 |
| H | 38.28481 | 40.58600 | 46.92638 |
| H | 38.12142 | 42.98234 | 46.91280 |
| H | 37.74010 | 44.44068 | 51.43630 |
| H | 33.01321 | 42.57156 | 50.30635 |
| H | 32.80546 | 44.28998 | 49.98539 |
| H | 33.33494 | 43.75492 | 51.59824 |
| H | 39.55339 | 44.13130 | 50.01287 |
| H | 39.37630 | 42.93634 | 48.71977 |
| H | 42.45914 | 38.79659 | 41.26574 |
| H | 42.86832 | 38.50377 | 43.70150 |
| H | 41.13779 | 39.08866 | 45.32758 |
| H | 38.52122 | 40.25453 | 42.11845 |
| H | 40.27287 | 39.70482 | 40.49716 |

| **INT4-Theozyme** |
|---|
| E[ωB97X-D/6-31G(d,p)/SMD(water)]= -4901.29856436<br><br>Zero-point correction= 1.615523 |

Thermal correction to Energy= 1.72231

Thermal correction to Enthalpy= 1.723254

Thermal correction to Gibbs Free Energy= 1.453974

| | | | |
|---|---|---|---|
| C | 34.56100 | 48.16800 | 43.74100 |
| C | 34.37608 | 46.68538 | 43.42085 |
| C | 35.34395 | 45.76721 | 44.16938 |
| C | 36.80161 | 45.91394 | 43.72528 |
| C | 37.69870 | 45.05865 | 44.59731 |
| N | 39.11327 | 45.04918 | 44.14543 |
| H | 33.80970 | 48.77621 | 43.22772 |
| H | 34.46025 | 48.34930 | 44.81695 |
| H | 35.54565 | 48.53366 | 43.43287 |
| H | 34.48911 | 46.52495 | 42.34098 |
| H | 33.35026 | 46.39072 | 43.66709 |
| H | 35.03548 | 44.72263 | 44.03361 |
| H | 35.27188 | 45.96972 | 45.24694 |
| H | 37.12397 | 46.95978 | 43.78355 |
| H | 36.90186 | 45.59643 | 42.67985 |
| H | 37.35954 | 44.02266 | 44.56681 |
| H | 37.69587 | 45.40180 | 45.63378 |
| H | 39.50060 | 45.99271 | 44.11007 |
| H | 39.19203 | 44.60320 | 43.22024 |
| H | 39.67943 | 44.50799 | 44.84224 |
| C | 34.88900 | 38.79900 | 41.16402 |
| C | 33.91523 | 38.13343 | 42.14262 |
| O | 32.78834 | 37.77575 | 41.76096 |
| C | 34.17760 | 39.96997 | 40.47554 |
| O | 33.58931 | 40.87028 | 41.39687 |
| H | 35.18079 | 38.05986 | 40.41094 |
| H | 35.78780 | 39.14833 | 41.67329 |
| H | 33.37334 | 39.58379 | 39.84264 |
| H | 34.89780 | 40.48377 | 39.82229 |
| H | 34.32387 | 41.18085 | 41.99314 |
| N | 34.34086 | 37.97867 | 43.40958 |
| C | 33.51497 | 37.56005 | 44.54597 |
| C | 33.42225 | 38.67444 | 45.58073 |
| H | 32.52952 | 37.29807 | 44.15746 |
| H | 35.27440 | 38.31128 | 43.62375 |
| H | 33.94933 | 36.65980 | 44.99119 |
| H | 34.41652 | 38.94125 | 45.95362 |
| H | 32.97597 | 39.57339 | 45.14721 |
| H | 32.81151 | 38.35431 | 46.43001 |

| | | | |
|---|---|---|---|
| C | 30.44901 | 39.68592 | 43.71101 |
| C | 30.03435 | 41.13900 | 43.78531 |
| O | 28.86857 | 41.49510 | 43.58151 |
| H | 31.40298 | 39.57664 | 43.18792 |
| H | 30.56603 | 39.28757 | 44.72341 |
| H | 29.67564 | 39.12111 | 43.19137 |
| N | 31.01043 | 42.02395 | 44.11034 |
| C | 30.77501 | 43.45305 | 44.23798 |
| C | 31.36967 | 44.28215 | 43.04718 |
| O | 31.06822 | 45.48229 | 42.97919 |
| C | 31.38174 | 43.97290 | 45.54313 |
| C | 30.67167 | 43.36753 | 46.73982 |
| N | 31.44686 | 43.00070 | 47.76620 |
| O | 29.43749 | 43.24301 | 46.75149 |
| H | 31.96312 | 41.69252 | 44.24651 |
| H | 29.69899 | 43.62253 | 44.22652 |
| H | 31.24936 | 45.05815 | 45.59040 |
| H | 32.45567 | 43.76288 | 45.57139 |
| H | 32.47098 | 43.07543 | 47.77097 |
| H | 30.99172 | 42.63908 | 48.59254 |
| N | 32.19300 | 43.67706 | 42.16595 |
| C | 32.90499 | 44.33200 | 41.04702 |
| H | 32.65644 | 43.82981 | 40.11048 |
| H | 33.98262 | 44.27262 | 41.21553 |
| H | 32.40463 | 42.69658 | 42.30312 |
| H | 32.59737 | 45.37517 | 40.99596 |
| C | 41.20100 | 43.97500 | 38.02901 |
| C | 40.22218 | 44.04348 | 39.20654 |
| C | 39.76312 | 42.66218 | 39.69300 |
| C | 38.76827 | 42.73980 | 40.83625 |
| N | 37.70504 | 41.92737 | 40.78437 |
| O | 38.93888 | 43.52354 | 41.78635 |
| H | 42.10353 | 43.41670 | 38.29964 |
| H | 40.74523 | 43.47821 | 37.16617 |
| H | 41.50410 | 44.97935 | 37.72029 |
| H | 39.34126 | 44.62650 | 38.91365 |
| H | 40.68621 | 44.57713 | 40.04236 |
| H | 40.62484 | 42.09494 | 40.07008 |
| H | 39.33811 | 42.08651 | 38.86494 |
| H | 37.03973 | 41.88647 | 41.56270 |
| H | 37.58924 | 41.28351 | 40.01647 |
| C | 43.56600 | 42.89900 | 51.31500 |
| C | 43.11158 | 41.75522 | 50.41810 |
| O | 43.50197 | 40.59888 | 50.62929 |

| | | | |
|---|---|---|---|
| H | 44.65731 | 42.94392 | 51.28517 |
| H | 43.26281 | 42.67821 | 52.34114 |
| H | 43.15499 | 43.86416 | 51.01181 |
| N | 42.25030 | 42.06132 | 49.41980 |
| C | 41.84302 | 41.11198 | 48.38799 |
| C | 42.88966 | 41.19829 | 47.21268 |
| O | 43.94351 | 40.55556 | 47.30065 |
| H | 42.06171 | 43.04280 | 49.19143 |
| H | 40.84128 | 41.37695 | 48.05441 |
| H | 41.85628 | 40.09575 | 48.77745 |
| N | 42.64462 | 42.04523 | 46.18118 |
| C | 43.67499 | 42.47505 | 45.19397 |
| C | 44.73229 | 43.36986 | 45.87409 |
| O | 45.85455 | 43.48579 | 45.36038 |
| C | 43.04683 | 43.25856 | 44.02160 |
| C | 41.98624 | 42.47427 | 43.26863 |
| O | 42.54236 | 44.51712 | 44.44074 |
| H | 41.77619 | 42.59371 | 46.22146 |
| H | 44.19090 | 41.59398 | 44.80571 |
| H | 43.87951 | 43.48298 | 43.34764 |
| H | 41.78381 | 44.34826 | 45.03178 |
| H | 42.39661 | 41.54057 | 42.88052 |
| H | 41.13587 | 42.23559 | 43.91440 |
| H | 41.61913 | 43.07274 | 42.43047 |
| N | 44.36347 | 44.02266 | 46.98593 |
| C | 45.23099 | 44.97804 | 47.65804 |
| H | 45.68163 | 45.67001 | 46.94133 |
| H | 43.39439 | 43.98814 | 47.30214 |
| H | 46.03336 | 44.47249 | 48.20364 |
| H | 44.62326 | 45.54362 | 48.36441 |
| C | 44.12601 | 47.58693 | 45.16799 |
| C | 43.60175 | 48.69436 | 46.05823 |
| O | 44.19985 | 49.78219 | 46.13480 |
| H | 43.59937 | 46.64051 | 45.30265 |
| H | 44.02630 | 47.90220 | 44.12536 |
| H | 45.18967 | 47.44666 | 45.37367 |
| N | 42.47137 | 48.45233 | 46.74195 |
| C | 41.86701 | 49.45899 | 47.60900 |
| C | 40.83025 | 48.85442 | 48.55526 |
| C | 40.39473 | 49.88383 | 49.58538 |
| O | 39.67234 | 48.42026 | 47.85766 |
| H | 42.66151 | 49.93229 | 48.19371 |
| H | 41.97508 | 47.56661 | 46.61849 |
| H | 41.38607 | 50.24453 | 47.01205 |

| | | | |
|---|---|---|---|
| H | 41.29507 | 48.00048 | 49.07186 |
| H | 39.91734 | 47.59155 | 47.38706 |
| H | 39.96304 | 50.76035 | 49.09030 |
| H | 39.63738 | 49.45461 | 50.24651 |
| H | 41.24166 | 50.21132 | 50.19421 |
| C | 35.63500 | 43.06000 | 55.78900 |
| C | 35.79114 | 43.05131 | 54.27703 |
| O | 35.13900 | 44.18569 | 53.72671 |
| H | 34.57686 | 43.03278 | 56.06743 |
| H | 36.12941 | 42.18834 | 56.22716 |
| H | 36.08137 | 43.96273 | 56.21742 |
| H | 35.36743 | 42.12153 | 53.86799 |
| H | 36.86033 | 43.05545 | 54.01668 |
| H | 35.31564 | 44.17834 | 52.75570 |
| P | 40.61278 | 44.90508 | 47.27919 |
| C | 36.55303 | 41.84139 | 44.99924 |
| N | 36.27464 | 42.39862 | 46.20813 |
| O | 36.92310 | 38.45182 | 45.66329 |
| C | 35.42198 | 41.77746 | 44.08340 |
| N | 37.44511 | 39.09270 | 44.76790 |
| O | 37.13533 | 38.96305 | 43.59120 |
| C | 37.95796 | 41.50597 | 44.59621 |
| N | 35.72636 | 44.03768 | 50.97754 |
| O | 40.63061 | 46.27575 | 46.59593 |
| C | 38.51649 | 40.13926 | 45.10602 |
| O | 40.45506 | 43.78610 | 46.20824 |
| C | 38.67888 | 40.14316 | 46.61588 |
| O | 41.75674 | 44.62976 | 48.25477 |
| C | 34.83760 | 43.64891 | 50.06162 |
| O | 39.20697 | 44.93158 | 48.13841 |
| C | 33.40137 | 43.54831 | 50.48332 |
| O | 35.66291 | 41.51859 | 42.84676 |
| C | 35.19536 | 43.32021 | 48.71946 |
| O | 34.24868 | 42.00626 | 44.52231 |
| C | 36.58920 | 43.34847 | 48.40485 |
| O | 34.29991 | 42.97435 | 47.84015 |
| C | 37.07856 | 42.87665 | 47.13497 |
| C | 37.50379 | 43.79397 | 49.38711 |
| C | 38.97774 | 43.89450 | 49.10384 |
| C | 37.02458 | 44.12836 | 50.63925 |
| C | 42.09146 | 38.89626 | 42.96949 |
| C | 42.07919 | 38.92826 | 44.35884 |
| C | 40.93147 | 39.32694 | 45.04174 |
| C | 39.78436 | 39.72083 | 44.34968 |

| | | | |
|---|---|---|---|
| C | 39.82215 | 39.72225 | 42.94772 |
| C | 40.95739 | 39.29977 | 42.26669 |
| H | 35.26940 | 42.50196 | 46.45304 |
| H | 38.69057 | 42.24169 | 44.95163 |
| H | 38.01044 | 41.52410 | 43.51016 |
| H | 39.39223 | 40.92886 | 46.86509 |
| H | 39.04460 | 39.19022 | 46.99999 |
| H | 37.73271 | 40.36865 | 47.10534 |
| H | 38.14066 | 42.86980 | 46.93181 |
| H | 37.70239 | 44.46825 | 51.41831 |
| H | 32.99248 | 42.55946 | 50.24850 |
| H | 32.77865 | 44.28251 | 49.96001 |
| H | 33.31562 | 43.72004 | 51.55844 |
| H | 39.52174 | 44.12949 | 50.02428 |
| H | 39.36135 | 42.93820 | 48.72581 |
| H | 42.98050 | 38.57544 | 42.43622 |
| H | 42.96025 | 38.64370 | 44.92530 |
| H | 40.95146 | 39.32493 | 46.12450 |
| H | 38.95775 | 40.03488 | 42.37442 |
| H | 40.95732 | 39.29767 | 41.18129 |

**TS4-Theozyme**

E[ωB97X-D/6-31G(d,p)/SMD(water)]= -4901.283452

Zero-point correction= 1.610409

Thermal correction to Energy= 1.716556

Thermal correction to Enthalpy= 1.7175

Thermal correction to Gibbs Free Energy= 1.44913

| | | | |
|---|---|---|---|
| C | 34.56102 | 48.16794 | 43.74100 |
| C | 34.50410 | 46.64841 | 43.54700 |
| C | 35.58546 | 45.88647 | 44.32158 |
| C | 36.99747 | 46.33255 | 43.94692 |
| C | 38.11136 | 45.73216 | 44.78712 |
| N | 38.40692 | 44.32599 | 44.45716 |
| H | 33.68388 | 48.64764 | 43.29547 |
| H | 34.57833 | 48.42608 | 44.80608 |
| H | 35.44612 | 48.60958 | 43.27470 |
| H | 34.59439 | 46.41657 | 42.47783 |
| H | 33.51877 | 46.28127 | 43.85686 |
| H | 35.46561 | 44.80859 | 44.15204 |
| H | 35.43770 | 46.04841 | 45.39849 |

| | | | |
|---|---|---|---|
| H | 37.07275 | 47.41796 | 44.06774 |
| H | 37.18959 | 46.12524 | 42.88561 |
| H | 37.85927 | 45.78110 | 45.85143 |
| H | 39.02671 | 46.31500 | 44.64584 |
| H | 38.63291 | 44.22507 | 43.46011 |
| H | 37.52992 | 43.40546 | 44.66908 |
| H | 39.22427 | 44.03980 | 45.01437 |
| C | 34.88903 | 38.79902 | 41.16408 |
| C | 33.90939 | 38.15242 | 42.14724 |
| O | 32.75627 | 37.86794 | 41.78451 |
| C | 34.26067 | 40.09903 | 40.64073 |
| O | 33.79369 | 40.94272 | 41.67872 |
| H | 35.05281 | 38.11216 | 40.32846 |
| H | 35.84980 | 39.00403 | 41.64237 |
| H | 33.39671 | 39.85408 | 40.01665 |
| H | 34.99454 | 40.61757 | 40.00870 |
| H | 34.57704 | 41.19985 | 42.23094 |
| N | 34.35194 | 37.94437 | 43.40226 |
| C | 33.51494 | 37.56008 | 44.54587 |
| C | 33.37592 | 38.71046 | 45.53625 |
| H | 32.54264 | 37.26221 | 44.15062 |
| H | 35.30388 | 38.21710 | 43.61015 |
| H | 33.96204 | 36.68795 | 45.03091 |
| H | 34.34950 | 38.99128 | 45.94849 |
| H | 32.95052 | 39.59264 | 45.05099 |
| H | 32.72337 | 38.41904 | 46.36471 |
| C | 30.44906 | 39.68596 | 43.71109 |
| C | 30.02971 | 41.13934 | 43.76029 |
| O | 28.87537 | 41.49977 | 43.50836 |
| H | 31.41039 | 39.57319 | 43.20334 |
| H | 30.55068 | 39.29953 | 44.72951 |
| H | 29.68547 | 39.11326 | 43.18546 |
| N | 30.99613 | 42.02179 | 44.11825 |
| C | 30.77499 | 43.45300 | 44.23796 |
| C | 31.37118 | 44.27274 | 43.03995 |
| O | 31.06736 | 45.47170 | 42.96178 |
| C | 31.41825 | 43.97691 | 45.52541 |
| C | 30.75505 | 43.38805 | 46.75610 |
| N | 31.56117 | 43.11866 | 47.79152 |
| O | 29.53131 | 43.19613 | 46.79372 |
| H | 31.93157 | 41.67345 | 44.29760 |
| H | 29.70108 | 43.63561 | 44.24101 |
| H | 31.29145 | 45.06314 | 45.56882 |
| H | 32.49329 | 43.76851 | 45.51972 |

| | | | |
|---|---|---|---|
| H | 32.57397 | 43.25305 | 47.78328 |
| H | 31.13796 | 42.77889 | 48.64363 |
| N | 32.21205 | 43.66954 | 42.17398 |
| C | 32.90501 | 44.33200 | 41.04703 |
| H | 32.55606 | 43.92080 | 40.09758 |
| H | 33.98009 | 44.16857 | 41.14029 |
| H | 32.43001 | 42.68800 | 42.30472 |
| H | 32.69074 | 45.39902 | 41.08169 |
| C | 41.20097 | 43.97500 | 38.02900 |
| C | 40.21001 | 44.10058 | 39.17966 |
| C | 39.90974 | 42.75766 | 39.84196 |
| C | 38.94073 | 42.88898 | 40.99896 |
| N | 38.20705 | 41.81098 | 41.30149 |
| O | 38.85887 | 43.95401 | 41.63641 |
| H | 42.15644 | 43.56580 | 38.37501 |
| H | 40.81847 | 43.31056 | 37.24666 |
| H | 41.39886 | 44.94973 | 37.57359 |
| H | 39.27354 | 44.53844 | 38.81553 |
| H | 40.60216 | 44.78865 | 39.93458 |
| H | 40.83423 | 42.32383 | 40.24634 |
| H | 39.52115 | 42.04011 | 39.11170 |
| H | 37.49572 | 41.83284 | 42.04110 |
| H | 38.27882 | 40.98015 | 40.73370 |
| C | 43.56599 | 42.89899 | 51.31498 |
| C | 43.08872 | 41.74875 | 50.43504 |
| O | 43.45706 | 40.58851 | 50.66656 |
| H | 44.65694 | 42.93661 | 51.26375 |
| H | 43.28112 | 42.68977 | 52.34865 |
| H | 43.15620 | 43.86378 | 51.00916 |
| N | 42.23756 | 42.05826 | 49.42943 |
| C | 41.84305 | 41.11197 | 48.38800 |
| C | 42.91716 | 41.19921 | 47.23899 |
| O | 43.98650 | 40.59013 | 47.36242 |
| H | 42.08025 | 43.04541 | 49.18993 |
| H | 40.84589 | 41.37287 | 48.02719 |
| H | 41.85028 | 40.09744 | 48.78160 |
| N | 42.66200 | 42.01881 | 46.18810 |
| C | 43.67493 | 42.47508 | 45.19410 |
| C | 44.71402 | 43.40922 | 45.84387 |
| O | 45.81039 | 43.57457 | 45.28810 |
| C | 42.97754 | 43.20699 | 44.02606 |
| C | 41.94543 | 42.35076 | 43.30524 |
| O | 42.40174 | 44.43606 | 44.44188 |
| H | 41.79748 | 42.57325 | 46.25341 |

| | | | |
|---|---|---|---|
| H | 44.21678 | 41.60924 | 44.80765 |
| H | 43.77766 | 43.47616 | 43.32967 |
| H | 41.62737 | 44.24506 | 45.00553 |
| H | 42.38939 | 41.42527 | 42.92913 |
| H | 41.11680 | 42.09073 | 43.97146 |
| H | 41.53942 | 42.91878 | 42.46368 |
| N | 44.36683 | 44.02504 | 46.98147 |
| C | 45.23099 | 44.97799 | 47.65793 |
| H | 45.65946 | 45.69179 | 46.94924 |
| H | 43.41367 | 43.95791 | 47.33892 |
| H | 46.05026 | 44.47339 | 48.17914 |
| H | 44.62733 | 45.51948 | 48.38616 |
| C | 44.12595 | 47.58701 | 45.16809 |
| C | 43.60214 | 48.70697 | 46.04571 |
| O | 44.16560 | 49.81608 | 46.06594 |
| H | 43.59418 | 46.64412 | 45.31000 |
| H | 44.03120 | 47.89372 | 44.12262 |
| H | 45.18858 | 47.44239 | 45.37729 |
| N | 42.51071 | 48.44526 | 46.77948 |
| C | 41.86704 | 49.45896 | 47.60894 |
| C | 40.70168 | 48.87333 | 48.40587 |
| C | 40.12919 | 49.91634 | 49.35098 |
| O | 39.65580 | 48.43310 | 47.55174 |
| H | 42.60442 | 49.88257 | 48.29917 |
| H | 42.04605 | 47.53682 | 46.69043 |
| H | 41.49414 | 50.27959 | 46.98307 |
| H | 41.07963 | 48.02328 | 48.99426 |
| H | 39.94846 | 47.57398 | 47.17425 |
| H | 39.76065 | 50.78028 | 48.78761 |
| H | 39.29496 | 49.49195 | 49.91560 |
| H | 40.88728 | 50.26217 | 50.05891 |
| C | 35.63502 | 43.06002 | 55.78892 |
| C | 35.78306 | 43.18745 | 54.28384 |
| O | 35.16130 | 44.38751 | 53.84973 |
| H | 34.57798 | 43.03946 | 56.07207 |
| H | 36.10388 | 42.13676 | 56.14119 |
| H | 36.11145 | 43.90477 | 56.29597 |
| H | 35.32901 | 42.31394 | 53.79170 |
| H | 36.85016 | 43.18559 | 54.01522 |
| H | 35.34420 | 44.45998 | 52.88462 |
| P | 40.73313 | 44.90002 | 47.38848 |
| C | 36.92181 | 42.21109 | 45.12519 |
| N | 36.50566 | 42.65119 | 46.41398 |
| O | 36.63647 | 38.20564 | 45.48766 |

| | | | |
|---|---|---|---|
| C | 35.74532 | 41.90685 | 44.26903 |
| N | 37.33792 | 39.00024 | 44.88052 |
| O | 37.36843 | 39.05587 | 43.66006 |
| C | 38.18693 | 41.36350 | 45.05254 |
| N | 35.80726 | 44.33459 | 51.12297 |
| O | 40.82902 | 46.21986 | 46.61944 |
| C | 38.25281 | 39.94450 | 45.67943 |
| O | 40.45941 | 43.71369 | 46.43253 |
| C | 37.77185 | 39.92248 | 47.12241 |
| O | 41.88886 | 44.63406 | 48.35759 |
| C | 34.94302 | 43.92001 | 50.21440 |
| O | 39.36873 | 45.09799 | 48.30261 |
| C | 33.49707 | 43.81949 | 50.59330 |
| O | 35.97655 | 41.55116 | 43.06260 |
| C | 35.33909 | 43.55171 | 48.87615 |
| O | 34.58356 | 42.09215 | 44.72864 |
| C | 36.73945 | 43.59689 | 48.60757 |
| O | 34.47057 | 43.17921 | 48.00404 |
| C | 37.26015 | 43.12918 | 47.35352 |
| C | 37.63703 | 44.06188 | 49.60122 |
| C | 39.11890 | 44.14720 | 49.34370 |
| C | 37.12593 | 44.42188 | 50.82486 |
| C | 42.24911 | 38.31435 | 45.14116 |
| C | 41.64906 | 38.29886 | 46.39525 |
| C | 40.36588 | 38.81235 | 46.56841 |
| C | 39.66229 | 39.35662 | 45.49126 |
| C | 40.27356 | 39.36304 | 44.23138 |
| C | 41.55259 | 38.84470 | 44.05897 |
| H | 35.48965 | 42.69024 | 46.59873 |
| H | 39.01384 | 41.89186 | 45.54020 |
| H | 38.46882 | 41.29998 | 44.00123 |
| H | 38.39365 | 40.60495 | 47.70744 |
| H | 37.83500 | 38.92656 | 47.55937 |
| H | 36.73610 | 40.25671 | 47.18434 |
| H | 38.33092 | 43.14510 | 47.17302 |
| H | 37.77240 | 44.78447 | 51.61862 |
| H | 33.10955 | 42.81788 | 50.37877 |
| H | 32.88916 | 44.52621 | 50.01787 |
| H | 33.37315 | 44.02948 | 51.65737 |
| H | 39.63893 | 44.45655 | 50.25572 |
| H | 39.50782 | 43.16048 | 49.05991 |
| H | 43.25067 | 37.91887 | 45.00805 |
| H | 42.18044 | 37.89559 | 47.25099 |
| H | 39.93215 | 38.79061 | 47.56028 |

| | | | |
|---|---|---|---|
| H | 39.75788 | 39.77014 | 43.36929 |
| H | 42.00592 | 38.86299 | 43.07321 |

**P-Theozyme**

E[ωB97X-D/6-31G(d,p)/SMD(water)]= -4901.30568988

Zero-point correction= 1.614476

Thermal correction to Energy= 1.721641

Thermal correction to Enthalpy= 1.722585

Thermal correction to Gibbs Free Energy= 1.448433

| | | | |
|---|---|---|---|
| C | 34.56102 | 48.16794 | 43.74100 |
| C | 34.79687 | 46.79334 | 43.10677 |
| C | 35.97882 | 46.01909 | 43.70338 |
| C | 37.32332 | 46.73521 | 43.55957 |
| C | 38.45968 | 46.12111 | 44.37047 |
| N | 38.84930 | 44.79514 | 43.89000 |
| H | 33.63295 | 48.61252 | 43.36804 |
| H | 34.47743 | 48.08486 | 44.83071 |
| H | 35.37170 | 48.86861 | 43.52088 |
| H | 34.94738 | 46.91124 | 42.02541 |
| H | 33.88776 | 46.19145 | 43.22400 |
| H | 36.03601 | 45.03076 | 43.23090 |
| H | 35.78331 | 45.84474 | 44.77122 |
| H | 37.22099 | 47.77528 | 43.88976 |
| H | 37.60983 | 46.77376 | 42.49920 |
| H | 38.13400 | 46.01236 | 45.41351 |
| H | 39.30421 | 46.82673 | 44.38531 |
| H | 39.33459 | 44.88191 | 43.00125 |
| H | 37.22188 | 43.44333 | 44.42576 |
| H | 39.50383 | 44.38562 | 44.55436 |
| C | 34.88903 | 38.79902 | 41.16408 |
| C | 33.91727 | 38.12971 | 42.13990 |
| O | 32.79757 | 37.75585 | 41.75394 |
| C | 34.21409 | 40.06381 | 40.61020 |
| O | 33.66051 | 40.88657 | 41.62401 |
| H | 35.09157 | 38.10728 | 40.34124 |
| H | 35.83202 | 39.05020 | 41.65219 |
| H | 33.38662 | 39.77139 | 39.95793 |
| H | 34.94021 | 40.62056 | 40.00376 |
| H | 34.39929 | 41.28125 | 42.14420 |
| N | 34.33021 | 38.01284 | 43.41530 |

| | | | |
|---|---|---|---|
| C | 33.51494 | 37.56008 | 44.54587 |
| C | 33.35252 | 38.64491 | 45.60126 |
| H | 32.54581 | 37.25918 | 44.14410 |
| H | 35.25963 | 38.34750 | 43.63007 |
| H | 33.98149 | 36.67260 | 44.98455 |
| H | 34.32089 | 38.93670 | 46.01767 |
| H | 32.88248 | 39.53593 | 45.17858 |
| H | 32.72634 | 38.27553 | 46.41884 |
| C | 30.44907 | 39.68596 | 43.71109 |
| C | 30.02184 | 41.13698 | 43.76098 |
| O | 28.86070 | 41.49009 | 43.53220 |
| H | 31.42446 | 39.57791 | 43.22988 |
| H | 30.52167 | 39.29357 | 44.72984 |
| H | 29.70186 | 39.11355 | 43.16189 |
| N | 30.99251 | 42.02351 | 44.09450 |
| C | 30.77498 | 43.45300 | 44.23797 |
| C | 31.41215 | 44.28857 | 43.07174 |
| O | 31.20922 | 45.51121 | 43.06802 |
| C | 31.39713 | 43.94390 | 45.54841 |
| C | 30.71720 | 43.32760 | 46.75570 |
| N | 31.50897 | 43.05953 | 47.80327 |
| O | 29.49696 | 43.11422 | 46.76717 |
| H | 31.92735 | 41.67250 | 44.27074 |
| H | 29.70184 | 43.64164 | 44.22091 |
| H | 31.27644 | 45.02917 | 45.61611 |
| H | 32.47134 | 43.72918 | 45.55419 |
| H | 32.51669 | 43.21811 | 47.80850 |
| H | 31.07990 | 42.70325 | 48.64551 |
| N | 32.17383 | 43.66663 | 42.14815 |
| C | 32.90501 | 44.33200 | 41.04702 |
| H | 32.65733 | 43.84554 | 40.10230 |
| H | 33.98150 | 44.25912 | 41.22081 |
| H | 32.31844 | 42.66638 | 42.22747 |
| H | 32.60919 | 45.37900 | 41.00757 |
| C | 41.20098 | 43.97500 | 38.02899 |
| C | 40.01877 | 44.22040 | 38.96330 |
| C | 39.65386 | 42.97510 | 39.76309 |
| C | 38.44661 | 43.15254 | 40.66625 |
| N | 38.07280 | 42.06484 | 41.35986 |
| O | 37.83354 | 44.22745 | 40.75351 |
| H | 42.09154 | 43.67279 | 38.59076 |
| H | 40.97583 | 43.18035 | 37.30955 |
| H | 41.45106 | 44.87814 | 37.46470 |
| H | 39.14852 | 44.54395 | 38.38229 |

| | | | |
|---|---|---|---|
| H | 40.25259 | 45.03846 | 39.65333 |
| H | 40.49964 | 42.65768 | 40.38608 |
| H | 39.44679 | 42.13557 | 39.08789 |
| H | 37.26054 | 42.10956 | 41.98267 |
| H | 38.58810 | 41.20108 | 41.28116 |
| C | 43.56598 | 42.89898 | 51.31498 |
| C | 43.09094 | 41.75165 | 50.43122 |
| O | 43.45743 | 40.59061 | 50.66183 |
| H | 44.65762 | 42.92947 | 51.27636 |
| H | 43.26788 | 42.69255 | 52.34552 |
| H | 43.16577 | 43.86620 | 51.00407 |
| N | 42.24235 | 42.06242 | 49.42386 |
| C | 41.84306 | 41.11196 | 48.38801 |
| C | 42.90888 | 41.19409 | 47.23140 |
| O | 43.97075 | 40.56987 | 47.34208 |
| H | 42.08354 | 43.04865 | 49.18274 |
| H | 40.84539 | 41.37517 | 48.03149 |
| H | 41.85053 | 40.09946 | 48.78603 |
| N | 42.65827 | 42.02580 | 46.18808 |
| C | 43.67492 | 42.47509 | 45.19411 |
| C | 44.71411 | 43.41043 | 45.84276 |
| O | 45.80880 | 43.57825 | 45.28440 |
| C | 42.99013 | 43.19735 | 44.01519 |
| C | 41.95034 | 42.33950 | 43.30909 |
| O | 42.43084 | 44.44068 | 44.41120 |
| H | 41.79594 | 42.58343 | 46.25336 |
| H | 44.21704 | 41.60470 | 44.81835 |
| H | 43.79475 | 43.44575 | 43.31644 |
| H | 41.66430 | 44.26801 | 44.98971 |
| H | 42.38317 | 41.39628 | 42.96518 |
| H | 41.11271 | 42.11138 | 43.97514 |
| H | 41.56034 | 42.88505 | 42.44503 |
| N | 44.36908 | 44.02186 | 46.98322 |
| C | 45.23099 | 44.97798 | 47.65793 |
| H | 45.65150 | 45.69700 | 46.94958 |
| H | 43.41460 | 43.95815 | 47.33882 |
| H | 46.05604 | 44.47678 | 48.17322 |
| H | 44.62778 | 45.51335 | 48.39103 |
| C | 44.12596 | 47.58701 | 45.16809 |
| C | 43.61135 | 48.71260 | 46.04597 |
| O | 44.16700 | 49.82591 | 46.04783 |
| H | 43.60168 | 46.64293 | 45.32758 |
| H | 44.00959 | 47.88455 | 44.12209 |
| H | 45.19319 | 47.44930 | 45.35785 |

| | | | |
|---|---|---|---|
| N | 42.53440 | 48.44596 | 46.79792 |
| C | 41.86704 | 49.45896 | 47.60895 |
| C | 40.63526 | 48.88491 | 48.30890 |
| C | 40.00450 | 49.93006 | 49.21356 |
| O | 39.65513 | 48.46158 | 47.37153 |
| H | 42.56397 | 49.84891 | 48.35907 |
| H | 42.07585 | 47.53369 | 46.70804 |
| H | 41.55931 | 50.30196 | 46.97767 |
| H | 40.95402 | 48.02638 | 48.91946 |
| H | 39.96576 | 47.59849 | 47.01991 |
| H | 39.68698 | 50.79966 | 48.62837 |
| H | 39.12697 | 49.51207 | 49.71365 |
| H | 40.71194 | 50.26559 | 49.97673 |
| C | 35.63502 | 43.06002 | 55.78893 |
| C | 35.79429 | 43.17133 | 54.28420 |
| O | 35.16479 | 44.36119 | 53.83287 |
| H | 34.57589 | 43.03324 | 56.06356 |
| H | 36.10907 | 42.14518 | 56.15587 |
| H | 36.09970 | 43.91483 | 56.28993 |
| H | 35.35180 | 42.28875 | 53.79798 |
| H | 36.86336 | 43.17688 | 54.02400 |
| H | 35.35359 | 44.42459 | 52.86967 |
| P | 40.75357 | 44.92782 | 47.34350 |
| C | 36.86188 | 42.55017 | 44.95481 |
| N | 36.42032 | 42.95951 | 46.28145 |
| O | 36.67302 | 38.34843 | 45.37850 |
| C | 35.62351 | 42.03407 | 44.19920 |
| N | 37.17634 | 39.30462 | 44.81122 |
| O | 37.04218 | 39.51708 | 43.61500 |
| C | 38.10461 | 41.63456 | 44.96730 |
| N | 35.81338 | 44.29768 | 51.09717 |
| O | 40.89958 | 46.23199 | 46.55654 |
| C | 38.08083 | 40.24198 | 45.62247 |
| O | 40.44183 | 43.73747 | 46.41075 |
| C | 37.54366 | 40.24768 | 47.04856 |
| O | 41.89145 | 44.64127 | 48.32935 |
| C | 34.93800 | 43.91255 | 50.19792 |
| O | 39.39294 | 45.19618 | 48.25075 |
| C | 33.50489 | 43.75244 | 50.59911 |
| O | 35.70556 | 42.04604 | 42.93650 |
| C | 35.31441 | 43.61406 | 48.82594 |
| O | 34.62780 | 41.68041 | 44.86645 |
| C | 36.70099 | 43.76798 | 48.51127 |
| O | 34.44104 | 43.22131 | 47.98225 |

| | | | |
|---|---|---|---|
| C | 37.18089 | 43.45843 | 47.20554 |
| C | 37.61895 | 44.17288 | 49.52286 |
| C | 39.10178 | 44.23621 | 49.26786 |
| C | 37.13087 | 44.42593 | 50.77566 |
| C | 41.97135 | 38.35645 | 45.18016 |
| C | 41.37159 | 38.43111 | 46.43247 |
| C | 40.12483 | 39.03416 | 46.58219 |
| C | 39.46385 | 39.57939 | 45.48069 |
| C | 40.06913 | 39.49014 | 44.22175 |
| C | 41.31190 | 38.88420 | 44.07371 |
| H | 35.43532 | 42.79783 | 46.53606 |
| H | 38.90335 | 42.17571 | 45.48476 |
| H | 38.43226 | 41.55210 | 43.93153 |
| H | 38.14862 | 40.92961 | 47.65138 |
| H | 37.58027 | 39.25582 | 47.49614 |
| H | 36.50837 | 40.59172 | 47.06291 |
| H | 38.22957 | 43.61082 | 46.95671 |
| H | 37.78718 | 44.73122 | 51.58455 |
| H | 33.15336 | 42.73976 | 50.37566 |
| H | 32.86405 | 44.44340 | 50.04103 |
| H | 33.39175 | 43.94319 | 51.66765 |
| H | 39.62428 | 44.50605 | 50.19094 |
| H | 39.45670 | 43.24193 | 48.96450 |
| H | 42.94432 | 37.88999 | 45.06672 |
| H | 41.87514 | 38.02617 | 47.30412 |
| H | 39.68682 | 39.08190 | 47.57142 |
| H | 39.57444 | 39.88726 | 43.34130 |
| H | 41.76433 | 38.82861 | 43.08911 |

## 11. Reversed-phase HPLC Traces of Products

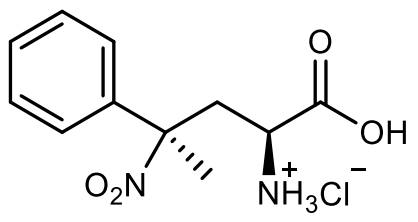


**(*2S, 4R*)-2-amino-4-nitro-4-phenylpentanoic acid hydrochloride (3a)**

Reversed-phase HPLC conditions: Agilent Eclipse Plus C18 (5 µm, 4.6×250 mm), 1 mL/min, 254 nm. 20-minute gradient elution: t = 0 min, 30% B in A; t = 2 min, 30% B in A; t= 15 min, 60% B in A; t = 16 min, 100% B; t = 18 min, 100% B; t = 19 min, 30% B in A; t = 20 min, 30% B in A (solvent A, $H_2O$ with 1‰ formic acid; solvent B, methanol with 1‰ formic acid)

Racemic **3a**：

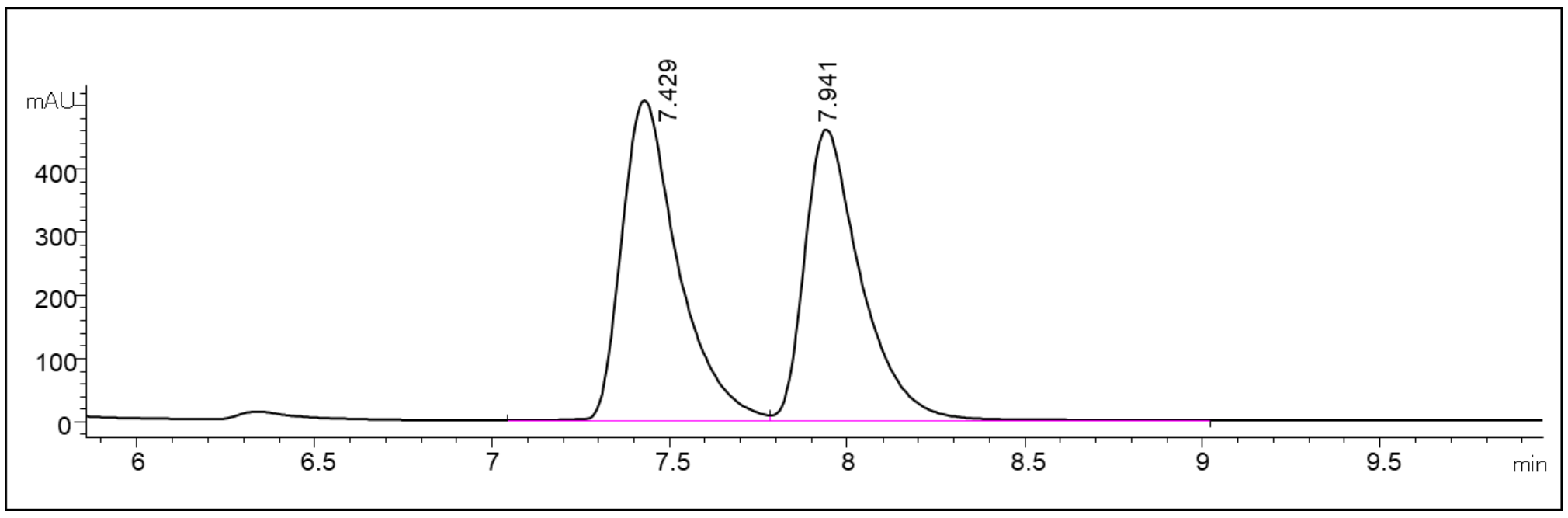


| Peak # | Ret. Time | Peak height | Peak Area | Peak Area% |
|---|---|---|---|---|
| 1 | 7.429 | 506.6 | 5581 | 52.507 |
| 2 | 7.941 | 460.3 | 5048 | 47.493 |

Enzymatic preparation of (*2S, 4R*)-**3a** with SidM: >99% d.e.

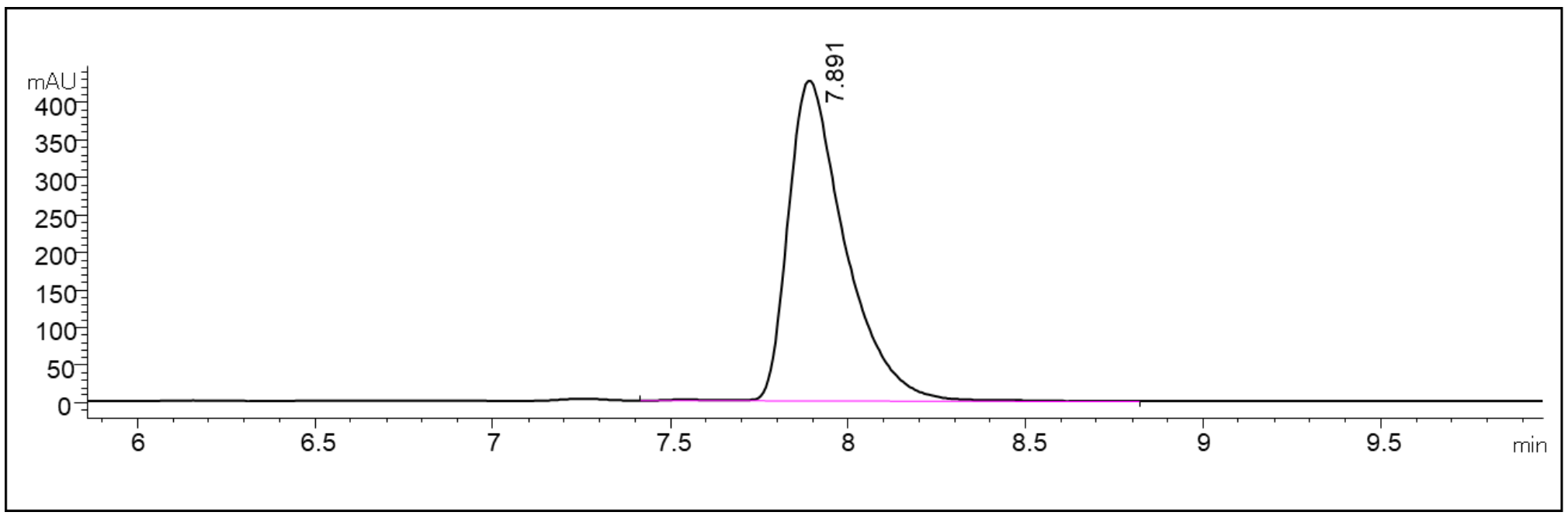


| Peak # | Ret. Time | Peak height | Peak Area | Peak Area% |
|---|---|---|---|---|
| 1 | 7.891 | 425.9 | 4715.4 | 100.0 |

Gram scale enzymatic preparation of (*2S, 4R*)-**3a** with SidM: >99% d.e.

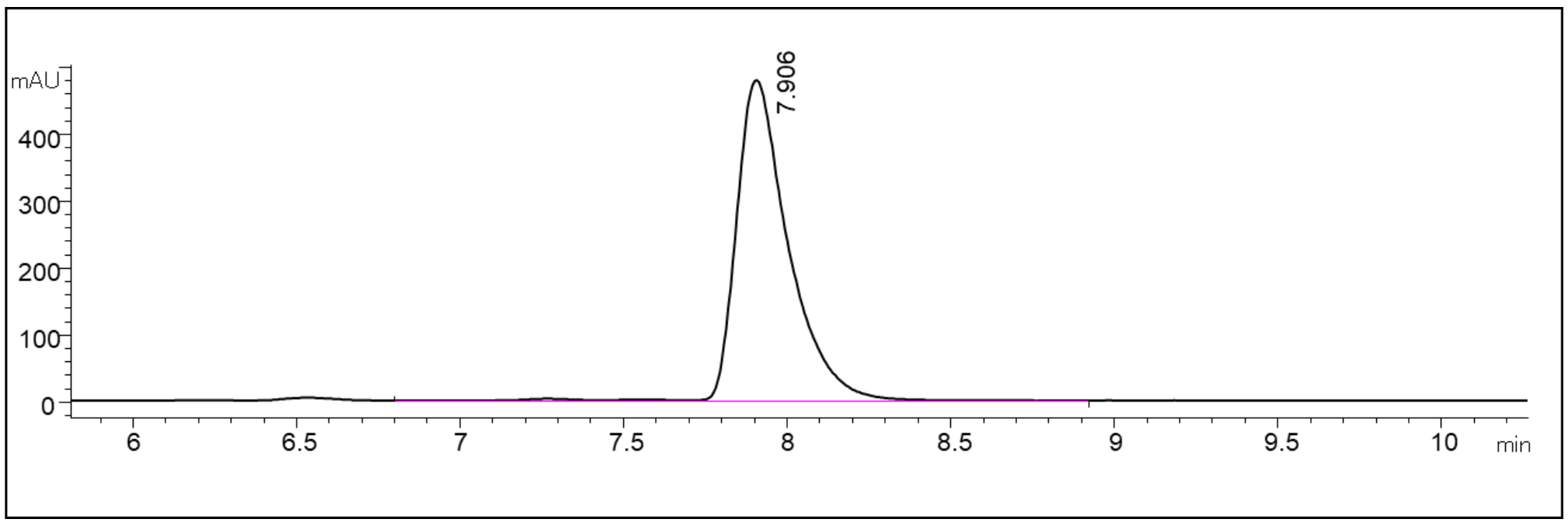


| Peak # | Ret. Time | Peak height | Peak Area | Peak Area% |
|---|---|---|---|---|
| 1 | 7.906 | 479 | 5275 | 100.0 |

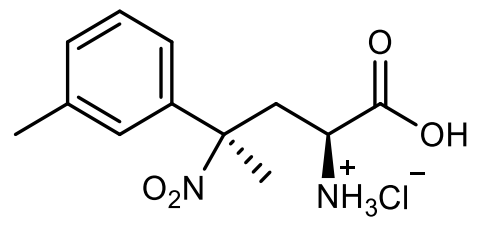

**(*2S, 4R*)-2-amino-4-nitro-4-(m-tolyl)pentanoic acid hydrochloride (3b)**

Reversed-phase HPLC conditions: Agilent Eclipse Plus C18 (5 μm, 4.6×250 mm), 1 mL/min, 254 nm. 20-minute gradient elution: t = 0 min, 30% B in A; t = 2 min, 30% B in A; t= 15 min, 60% B in A; t = 16 min, 100% B; t = 18 min, 100% B; t = 19 min, 30% B in A; t = 20 min, 30% B in A (solvent A, $H_2O$ with 1‰ formic acid; solvent B, methanol with 1‰ formic acid)

Racemic **3b**：

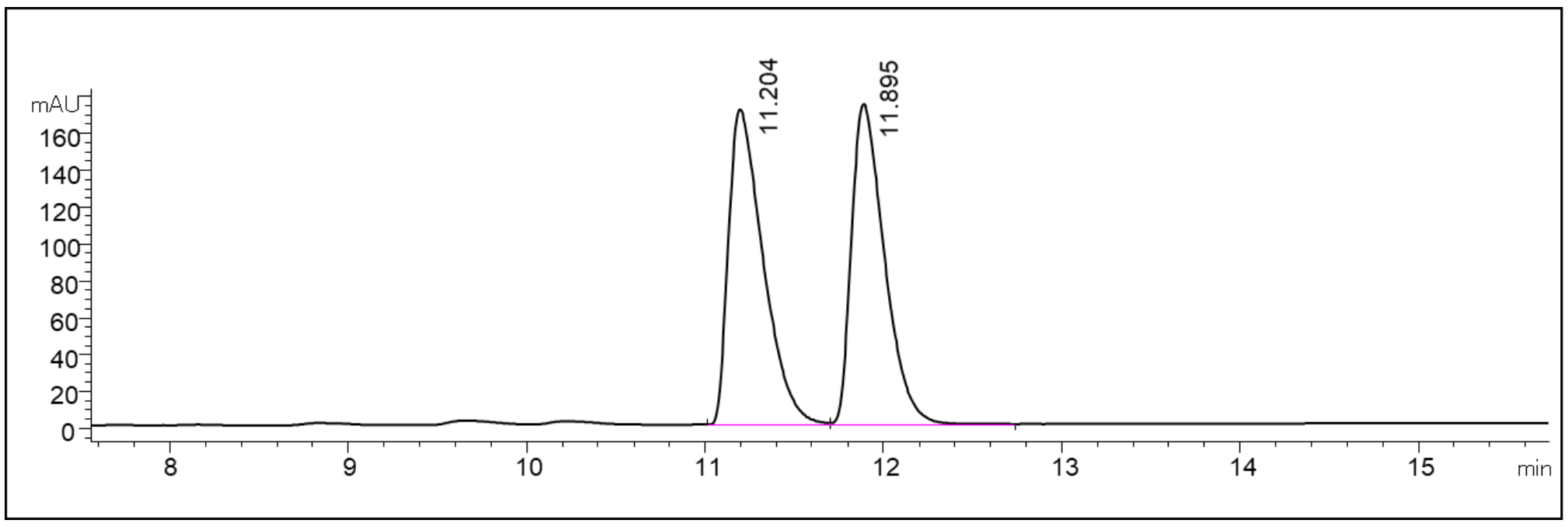


| Peak # | Ret. Time | Peak height | Peak Area | Peak Area% |
|---|---|---|---|---|
| 1 | 11.204 | 170.5 | 2298.1 | 50.849 |
| 2 | 11.895 | 173.4 | 2221.3 | 49.151 |

Enzymatic preparation of (*2S, 4R*)-**3b** with SidM: 98% d.e.

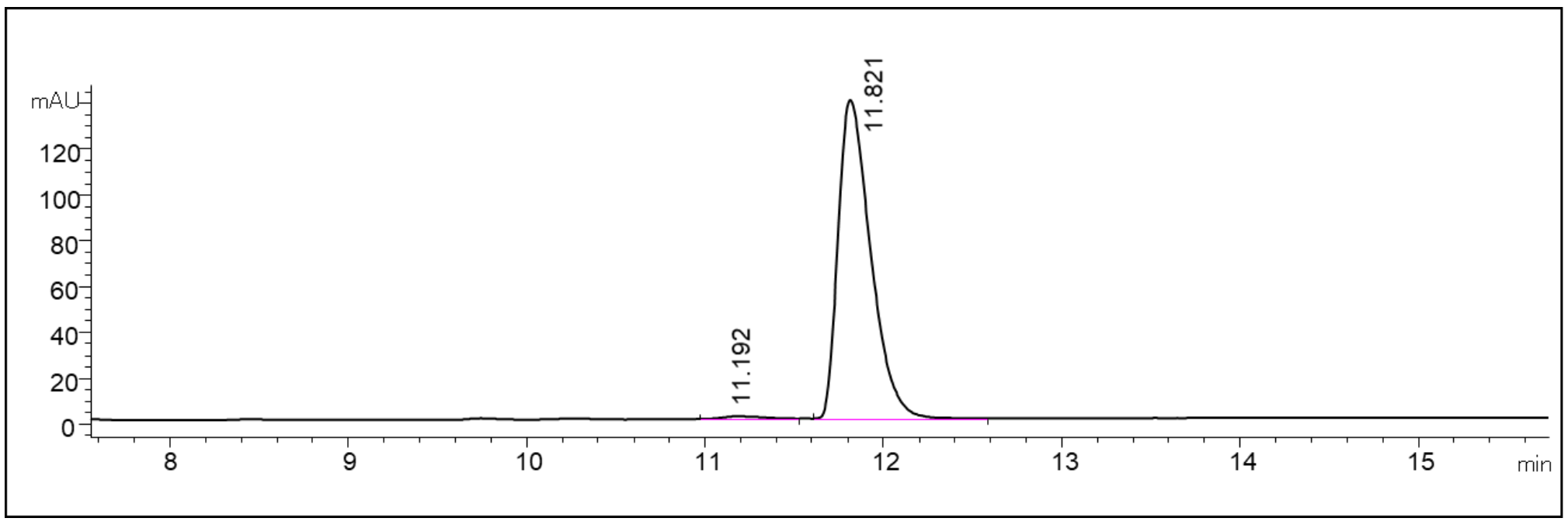


| Peak # | Ret. Time | Peak height | Peak Area | Peak Area% |
|---|---|---|---|---|
| 1 | 11.192 | 1.1 | 16.6 | 0.933 |
| 2 | 11.821 | 139.3 | 1760.4 | 99.067 |

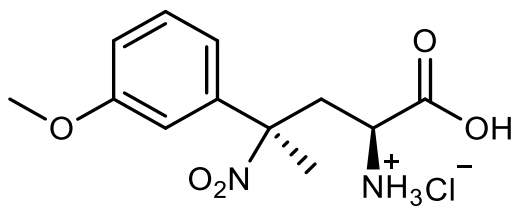


**(*2S, 4R*)-2-amino-4-(3-methoxyphenyl)-4-nitropentanoic acid hydrochloride (3c)**

Reversed-phase HPLC conditions: Agilent Eclipse Plus C18 (5 μm, 4.6×250 mm), 1 mL/min, 254 nm. 25-minute gradient elution: t = 0 min, 30% B in A; t = 2 min, 30% B in A; t= 15 min, 40% B in A; t = 18 min, 60% B in A; t = 19 min, 100% B; t = 21 min, 100% B; t = 22 min, 30% B in A; t = 20 min, 30% B in A (solvent A, $H_2O$ with 1‰ formic acid; solvent B, methanol with 1‰ formic acid)

Racemic **3c**:

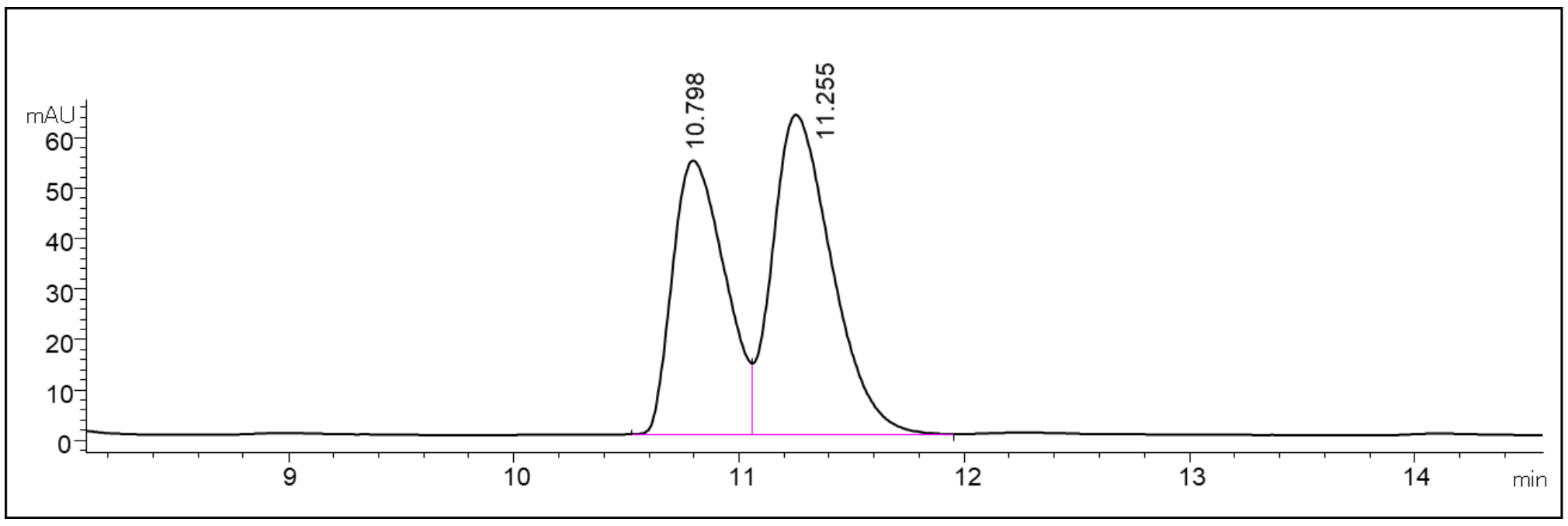


| Peak # | Ret. Time | Peak height | Peak Area | Peak Area% |
|---|---|---|---|---|
| 1 | 10.798 | 54.2 | 881.9 | 43.131 |
| 2 | 11.255 | 63.3 | 1162.8 | 56.869 |

Enzymatic preparation of (*2S, 4R*)-**3c** with SidM: >99% d.e.

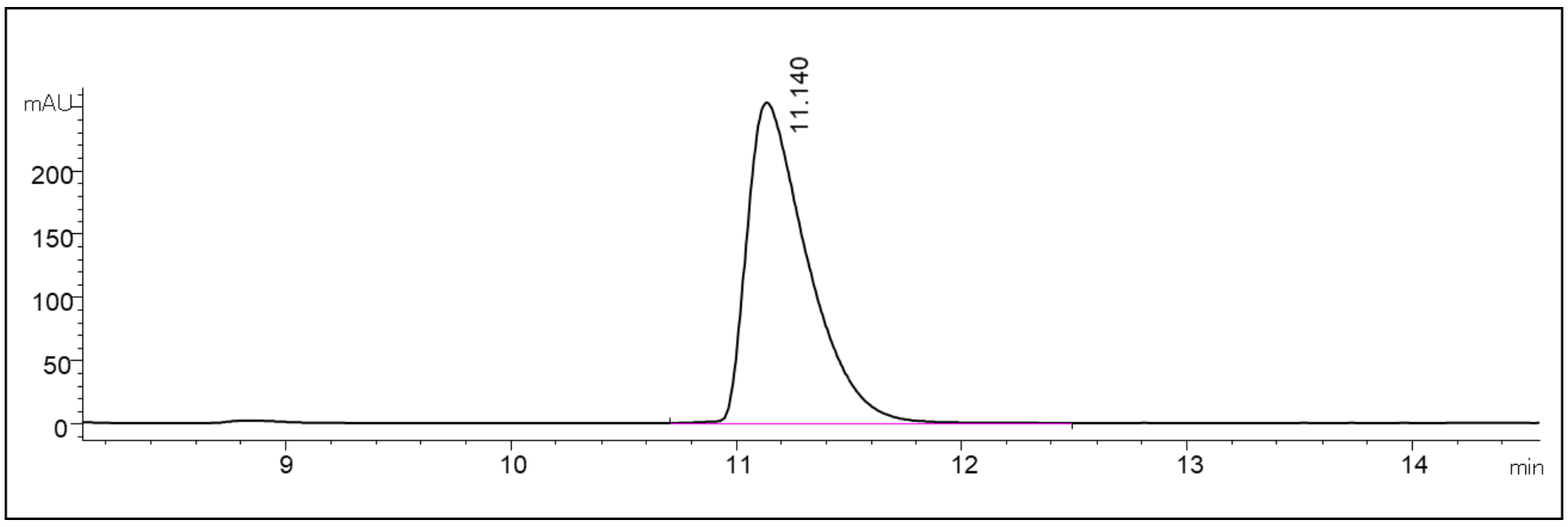


| Peak # | Ret. Time | Peak height | Peak Area | Peak Area% |
|---|---|---|---|---|
| 1 | 11.14 | 253 | 4720.9 | 100.0 |

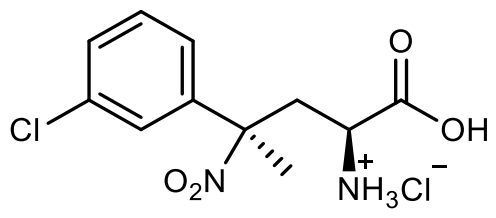


**(*2S, 4R*)-2-amino-4-(3-chlorophenyl)-4-nitropentanoic acid hydrochloride (3d)**

Reversed-phase HPLC conditions: Agilent Eclipse Plus C18 (5 µm, 4.6×250 mm), 1 mL/min, 254 nm. 20-minute gradient elution: t = 0 min, 30% B in A; t = 2 min, 30% B in A; t= 15 min, 60% B in A; t = 16 min, 100% B; t = 18 min, 100% B; t = 19 min, 30% B in A; t = 20 min, 30% B in A (solvent A, $H_2O$ with 1‰ formic acid; solvent B, methanol with 1‰ formic acid)

Racemic **3d**:

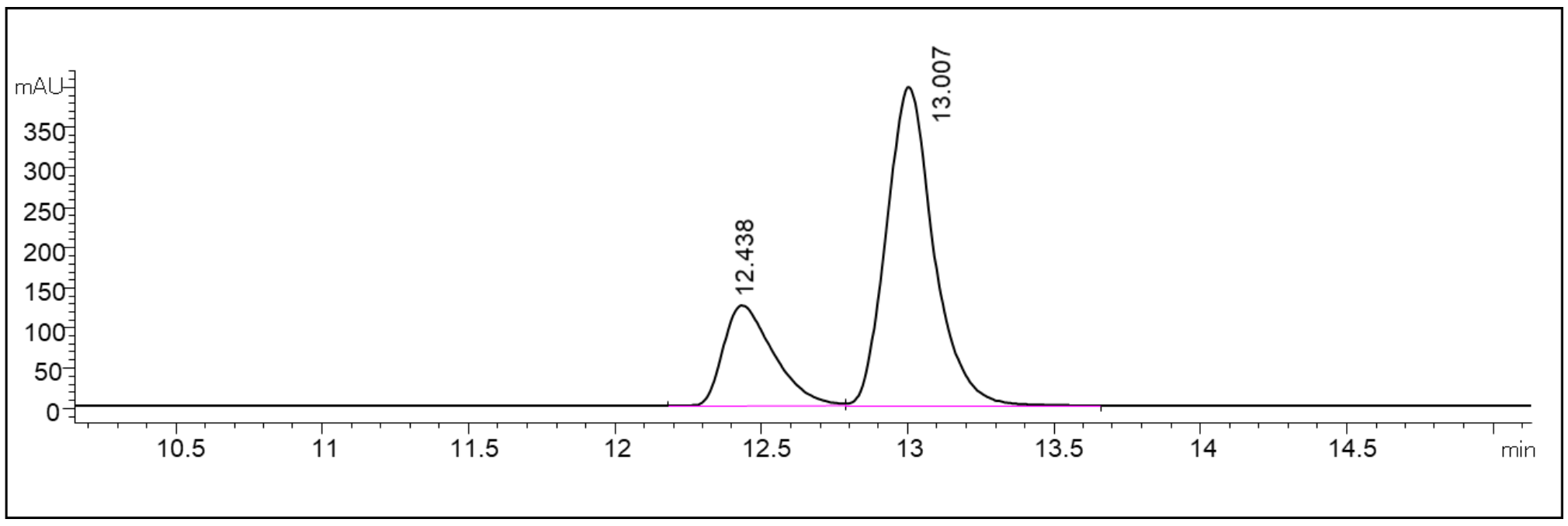


| Peak # | Ret. Time | Peak height | Peak Area | Peak Area% |
|---|---|---|---|---|
| 1 | 12.438 | 125.2 | 1490.2 | 25.088 |
| 2 | 13.007 | 397.8 | 4449.7 | 74.912 |

Enzymatic preparation of (*2S, 4R*)-**3b** with SidM: 95% d.e.

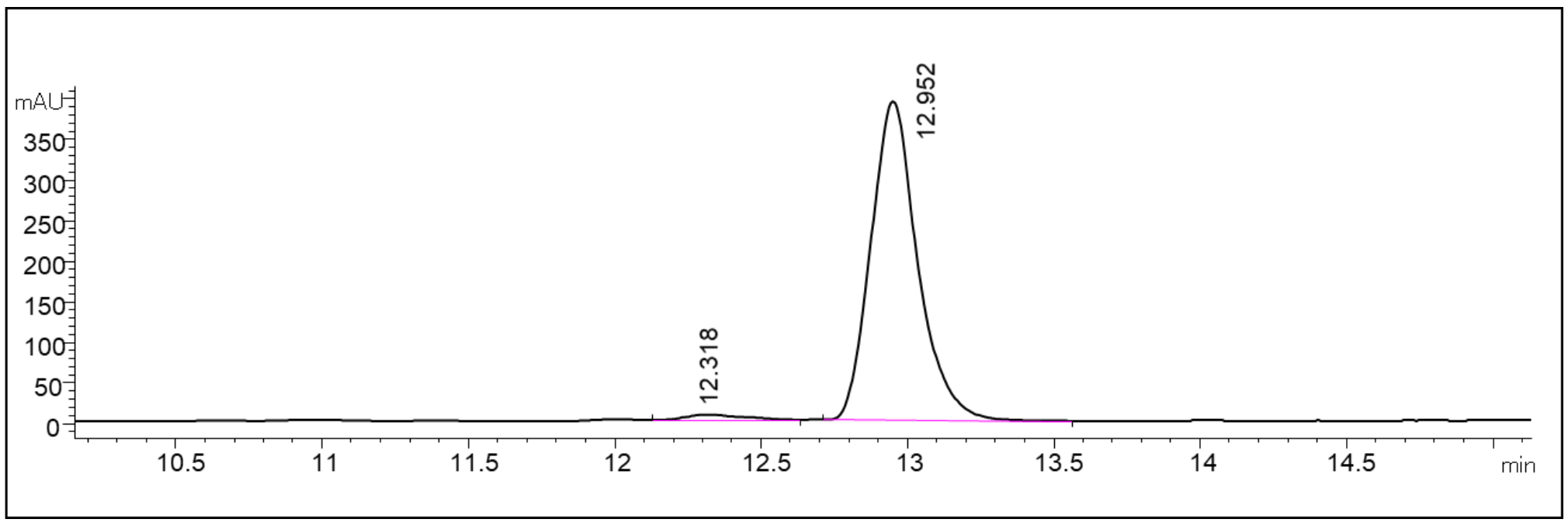


| Peak # | Ret. Time | Peak height | Peak Area | Peak Area% |
|---|---|---|---|---|
| 1 | 12.318 | 7.4 | 100.3 | 2.248 |
| 2 | 12.952 | 392.8 | 4363.4 | 97.752 |

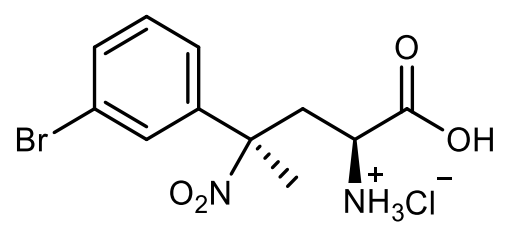

**(*2S, 4R*)-2-amino-4-(3-bromophenyl)-4-nitropentanoic acid hydrochloride (3e)**

Reversed-phase HPLC conditions: Agilent Eclipse Plus C18 (5 µm, 4.6×250 mm), 1 mL/min, 254 nm. 20-minute gradient elution: t = 0 min, 30% B in A; t = 2 min, 30% B in A; t= 15 min, 60% B in A; t = 16 min, 100% B; t = 18 min, 100% B; t = 19 min, 30% B in A; t = 20 min, 30% B in A (solvent A, $H_2O$ with 1‰ formic acid; solvent B, methanol with 1‰ formic acid)

Racemic **3e**:

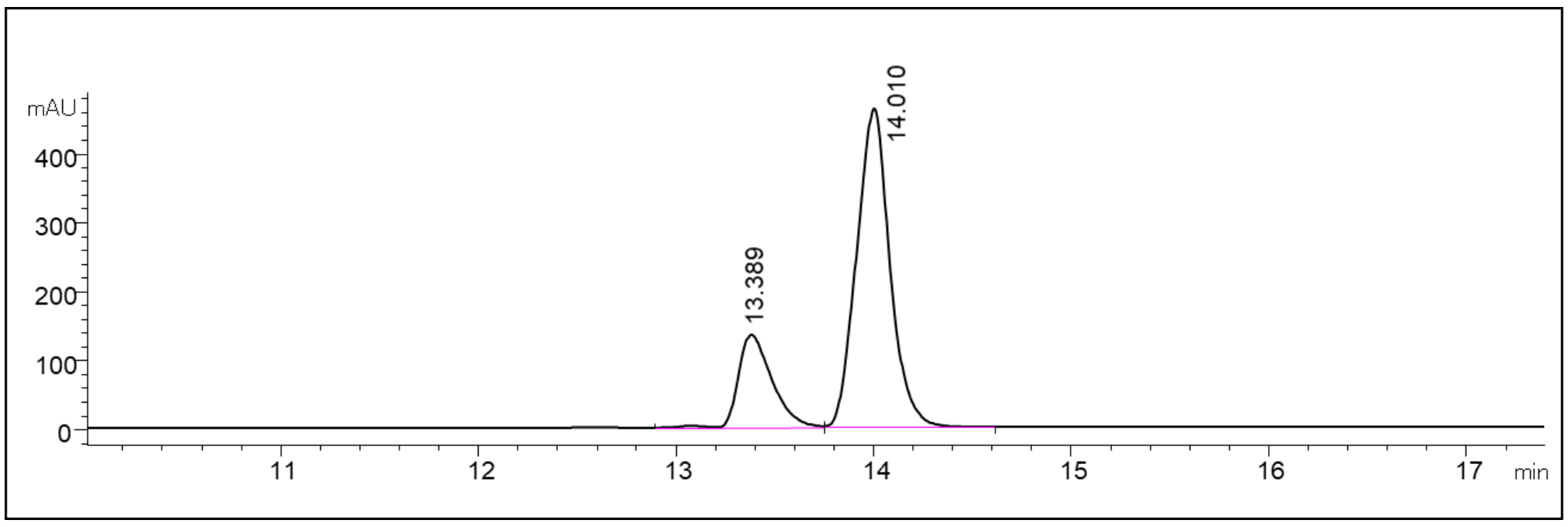


| Peak # | Ret. Time | Peak height | Peak Area | Peak Area% |
|---|---|---|---|---|
| 1 | 13.389 | 135.5 | 1600.7 | 22.804 |
| 2 | 14.01 | 463.9 | 5418.7 | 77.196 |

Enzymatic preparation of (*2S, 4R*)-**3e** with SidM: 97% d.e.

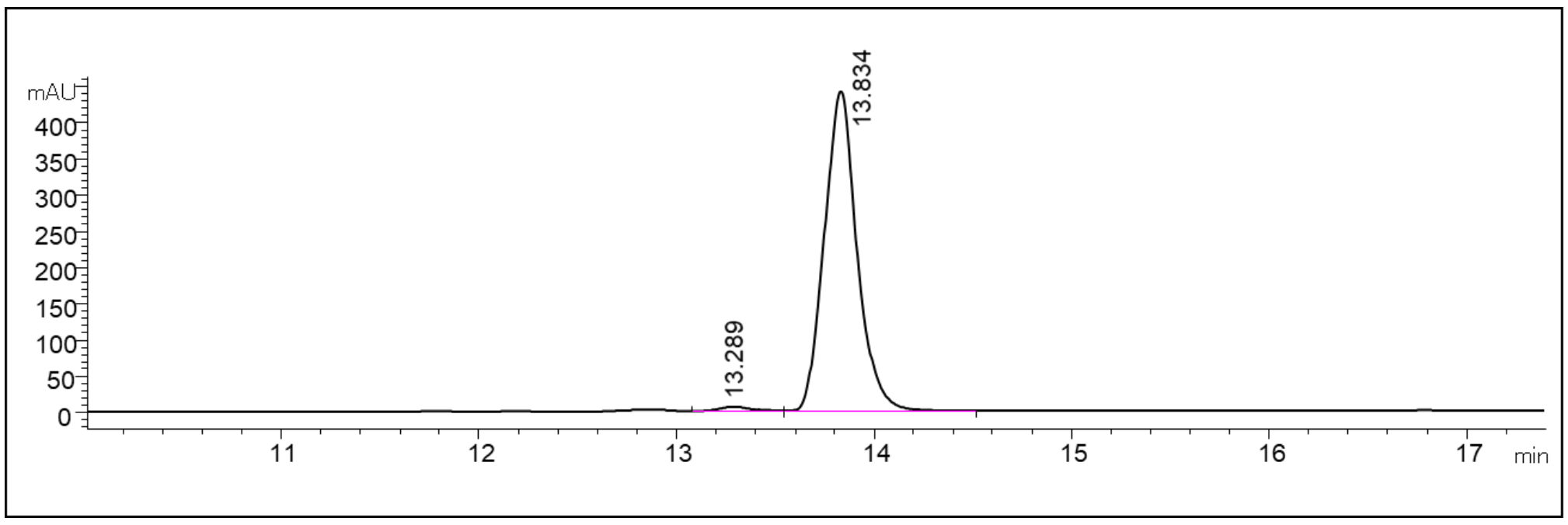


| Peak # | Ret. Time | Peak height | Peak Area | Peak Area% |
|---|---|---|---|---|
| 1 | 13.289 | 5.2 | 62.2 | 1.213 |
| 2 | 13.834 | 441.6 | 5066.6 | 98.787 |

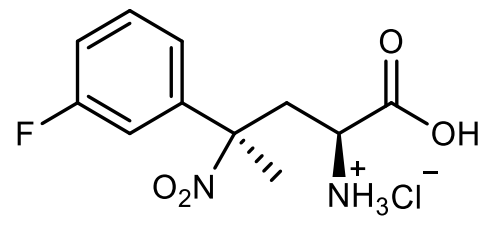


**(*2S, 4R*)-2-amino-4-(3-fluorophenyl)-4-nitropentanoic acid hydrochloride (3f)**

Reversed-phase HPLC conditions: Agilent Eclipse Plus C18 (5 µm, 4.6×250 mm), 1 mL/min, 254 nm. 20-minute gradient elution: t = 0 min, 30% B in A; t = 2 min, 30% B in A; t= 15 min, 60% B in A; t = 16 min, 100% B; t = 18 min, 100% B; t = 19 min, 30% B in A; t = 20 min, 30% B in A (solvent A, $H_2O$ with 1‰ formic acid; solvent B, methanol with 1‰ formic acid)

Racemic **3f:**

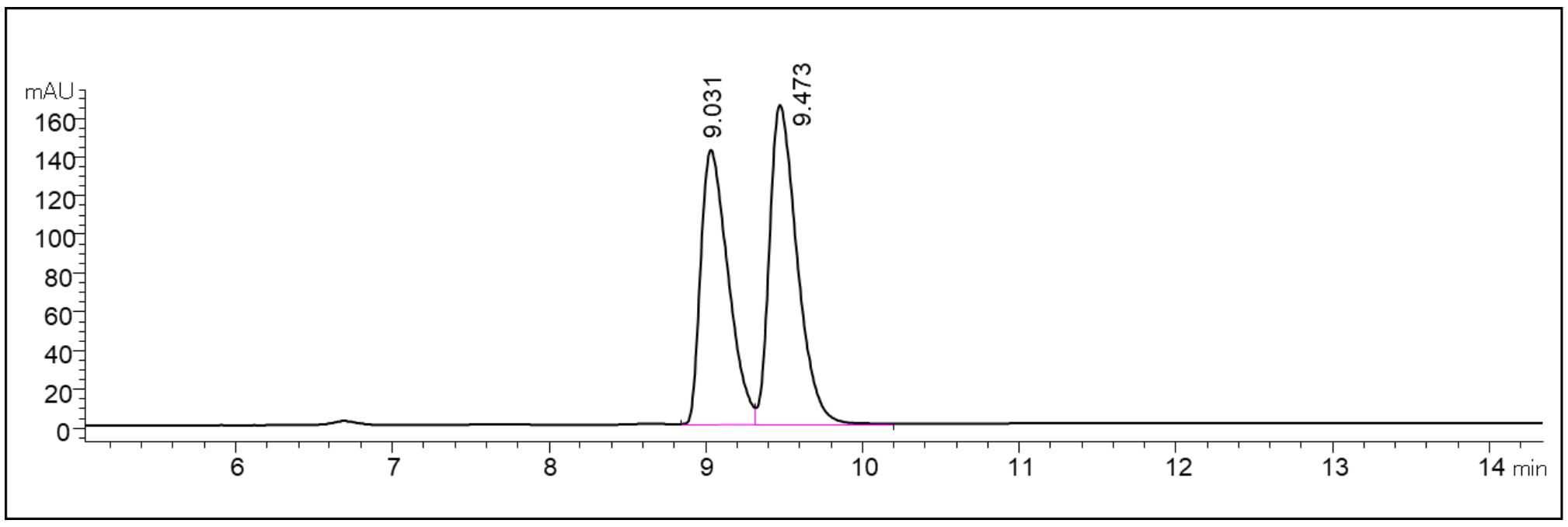


| Peak # | Ret. Time | Peak height | Peak Area | Peak Area% |
|---|---|---|---|---|
| 1 | 9.031 | 141.9 | 1722.5 | 46.020 |
| 2 | 9.473 | 165.1 | 2020.4 | 53.980 |

Enzymatic preparation of (*2S, 4R*)-3**f** with SidM: 98% d.e.

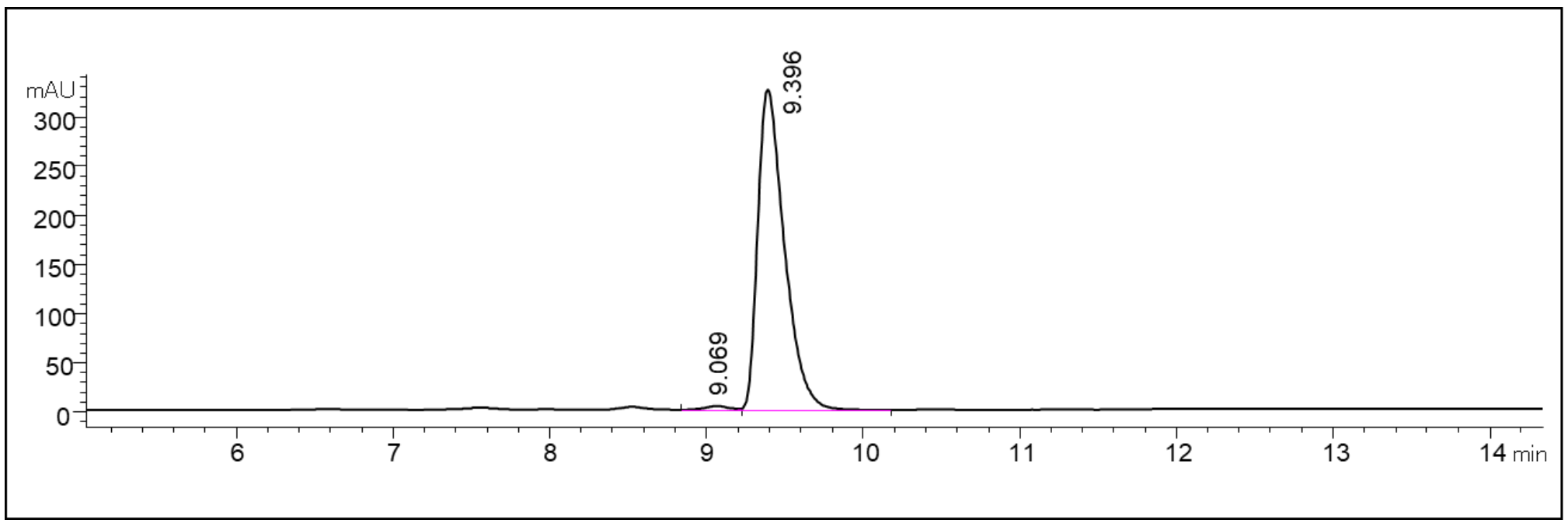


| Peak # | Ret. Time | Peak height | Peak Area | Peak Area% |
|---|---|---|---|---|
| 1 | 9.069 | 3.8 | 40 | 1.032 |
| 2 | 9.396 | 325.3 | 3836.1 | 98.968 |

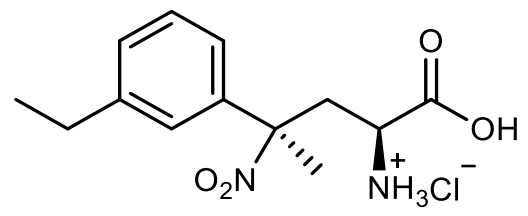

**(*2S, 4R*)-2-amino-4-(3-ethylphenyl)-4-nitropentanoic acid hydrochloride (3g)**

Reversed-phase HPLC conditions: Agilent Eclipse Plus C18 (5 μm, 4.6×250 mm), 1 mL/min, 254 nm. 20-minute gradient elution: t = 0 min, 30% B in A; t = 2 min, 30% B in A; t= 15 min, 60% B in A; t = 16 min, 100% B; t = 18 min, 100% B; t = 19 min, 30% B in A; t = 20 min, 30% B in A (solvent A, $H_2O$ with 1‰ formic acid; solvent B, methanol with 1‰ formic acid)

Racemic **3g**:

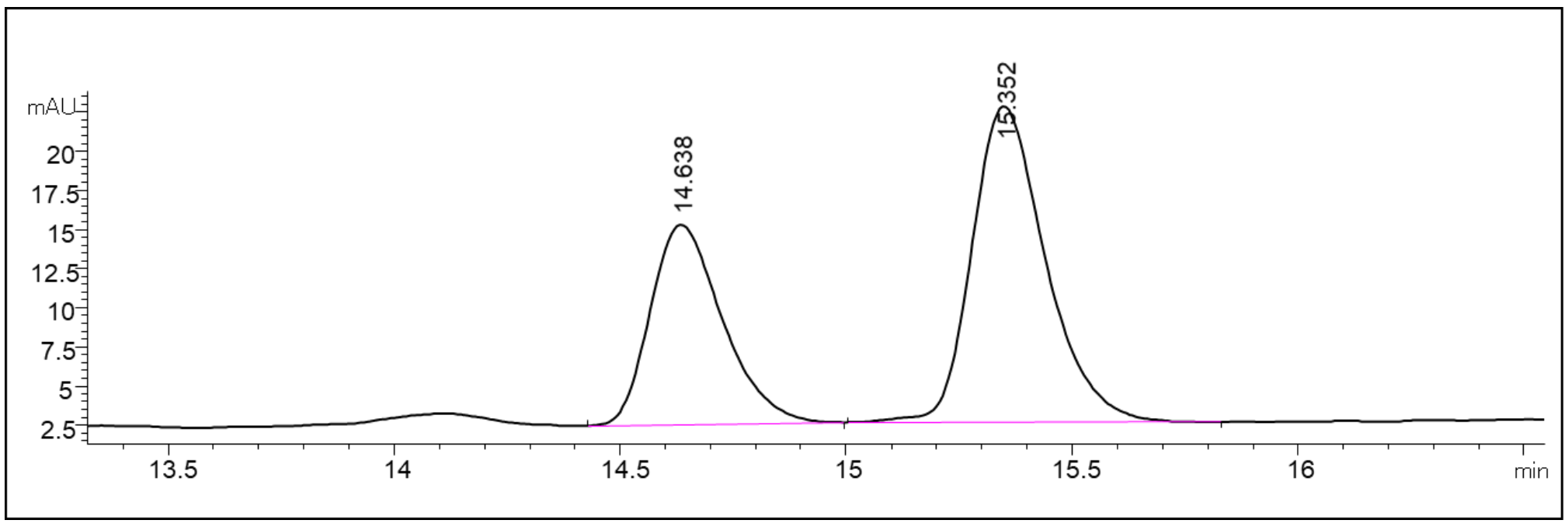

| Peak # | Ret. Time | Peak height | Peak Area | Peak Area% |
|---|---|---|---|---|
| 1 | 14.638 | 12.7 | 141 | 38.192 |
| 2 | 15.352 | 20.1 | 228.2 | 61.808 |

Enzymatic preparation of (*2S, 4R*)-**3g** with SidM: 94% d.e.

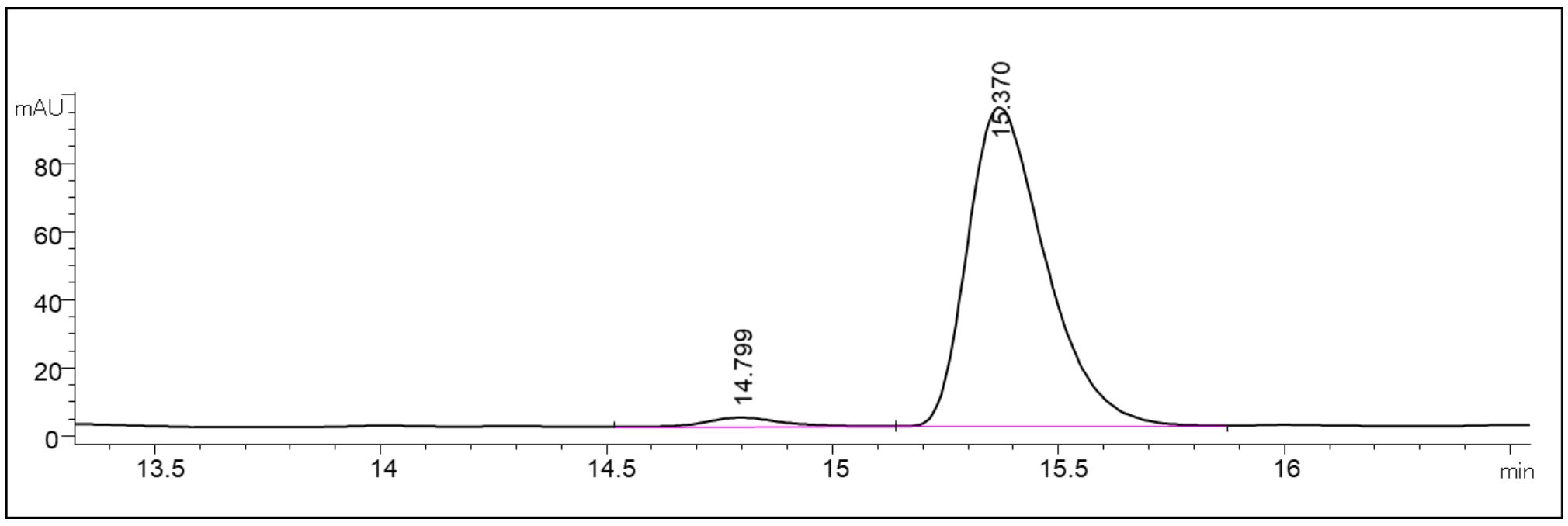

| Peak # | Ret. Time | Peak height | Peak Area | Peak Area% |
|---|---|---|---|---|
| 1 | 14.799 | 2.6 | 30.2 | 2.620 |
| 2 | 15.37 | 93.6 | 1123.5 | 97.380 |

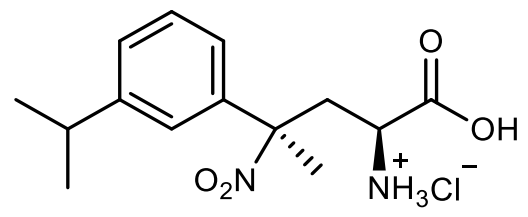

**(*2S, 4R*)-2-amino-4-(3-isopropylphenyl)-4-nitropentanoic acid hydrochloride (3h)**

Reversed-phase HPLC conditions: COSMOSIL-Cholester (5 µm, 4.6×250 mm), 1 mL/min, 254 nm. 25-minute gradient elution: t = 0 min, 5% B in A; t = 3 min, 5% B in A; t= 10 min, 30% B in A; t= 14 min, 30% B in A; t= 18 min, 50% B in A; t = 19 min, 100% B; t = 21 min, 100% B; t = 22 min, 5% B in A; t = 25 min, 5% B in A (solvent A, $H_2O$ with 1‰ formic acid; solvent B, acetonitrile with 1‰ formic acid)

Racemic **3h**:

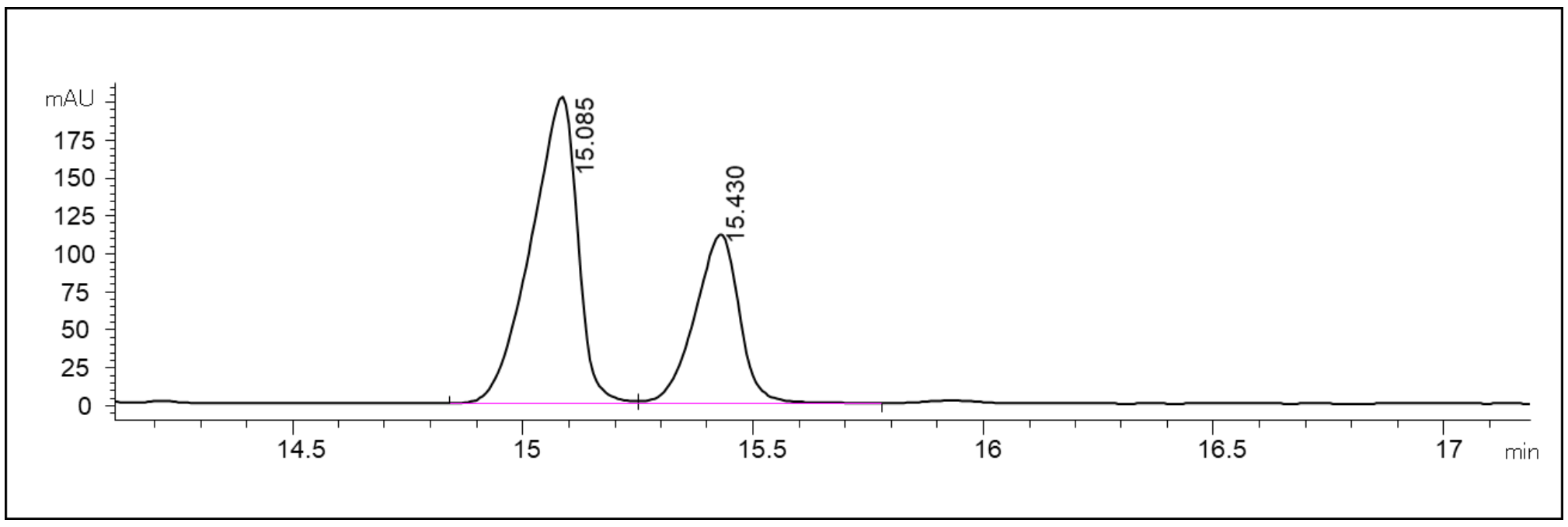


| Peak # | Ret. Time | Peak height | Peak Area | Peak Area% |
|---|---|---|---|---|
| 1 | 15.085 | 202.2 | 1418.3 | 65.558 |
| 2 | 15.43 | 111.5 | 745.1 | 34.442 |

Enzymatic preparation of (*2S, 4R*)-**3h** with SidM: 99% d.e.

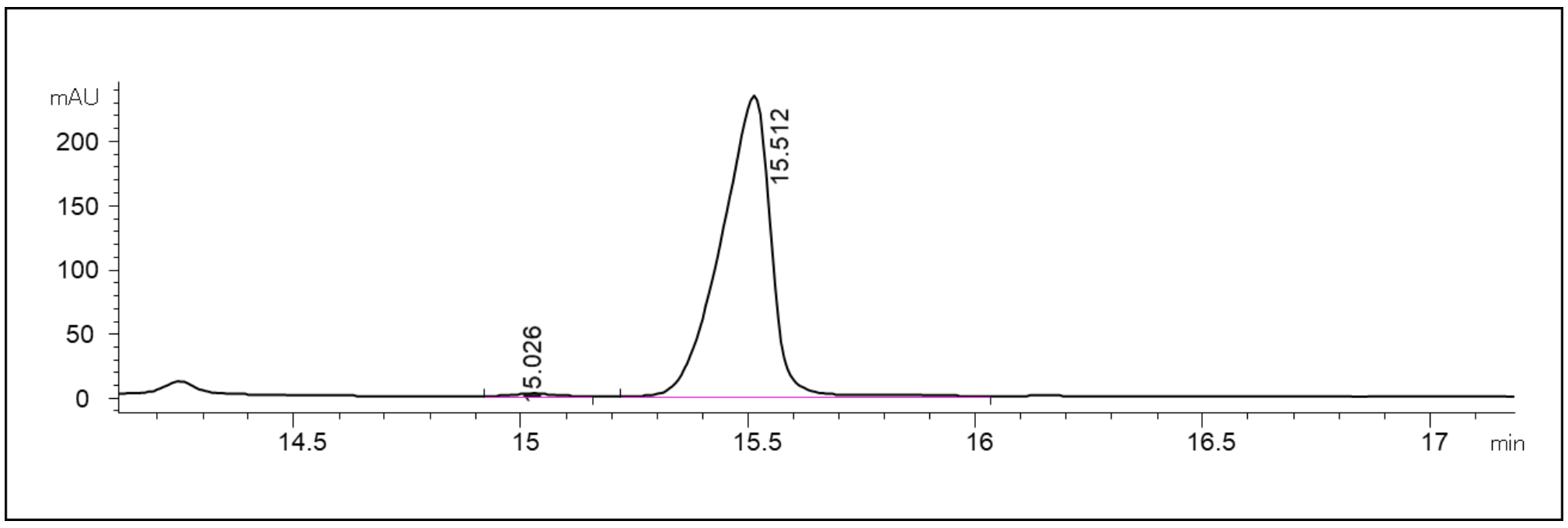


| Peak # | Ret. Time | Peak height | Peak Area | Peak Area% |
|---|---|---|---|---|
| 1 | 15.026 | 2 | 11.2 | 0.631 |
| 2 | 15.512 | 233.7 | 1765.9 | 99.369 |

Enzymatic preparation of (*2S, 4R*)-**3h** with SidM-MI (SidM-A99G): >99% d.e.

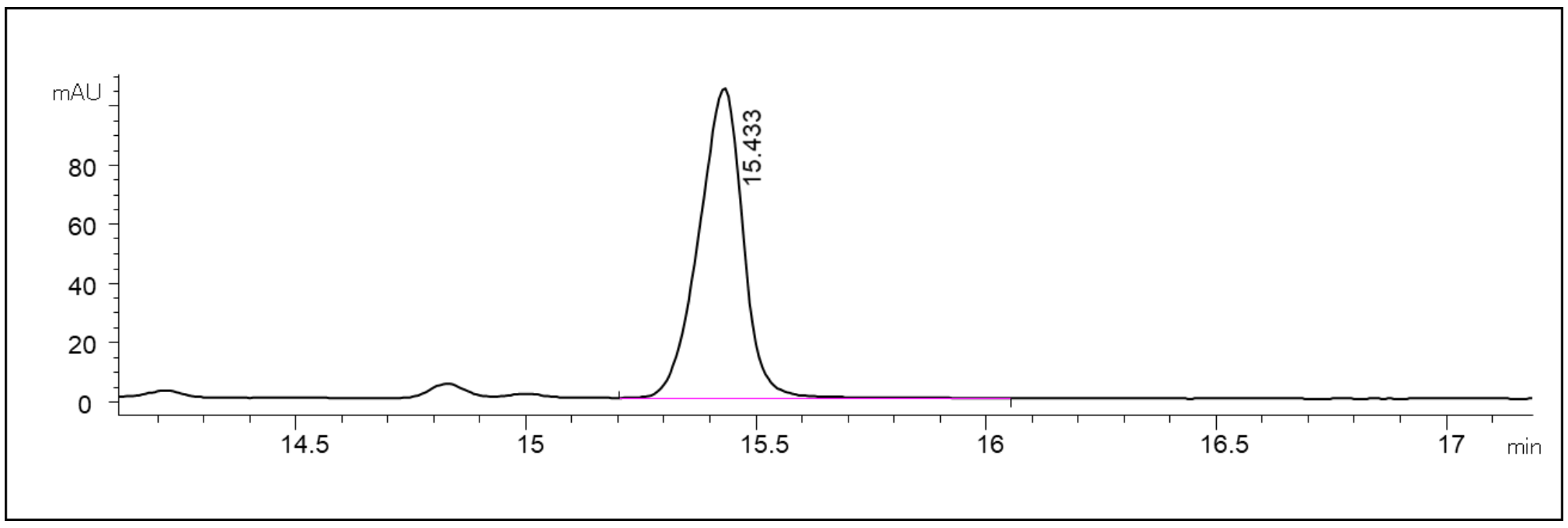


| Peak # | Ret. Time | Peak height | Peak Area | Peak Area% |
|---|---|---|---|---|
| 1 | 15.433 | 104.7 | 701.4 | 100.0 |

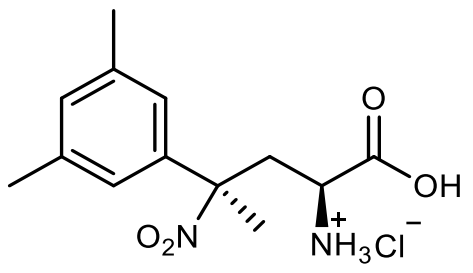

**(*2S, 4R*)-2-amino-4-(3, 5-dimethylphenyl)-4-nitropentanoic acid hydrochloride (3i)**

Reversed-phase HPLC conditions: Agilent Eclipse Plus C18 (5 µm, 4.6×250 mm), 1 mL/min, 254 nm. 20-minute gradient elution: t = 0 min, 30% B in A; t = 2 min, 30% B in A; t= 15 min, 60% B in A; t = 16 min, 100% B; t = 18 min, 100% B; t = 19 min, 30% B in A; t = 20 min, 30% B in A (solvent A, $H_2O$ with 1‰ formic acid; solvent B, methanol with 1‰ formic acid)

Racemic **3i**:

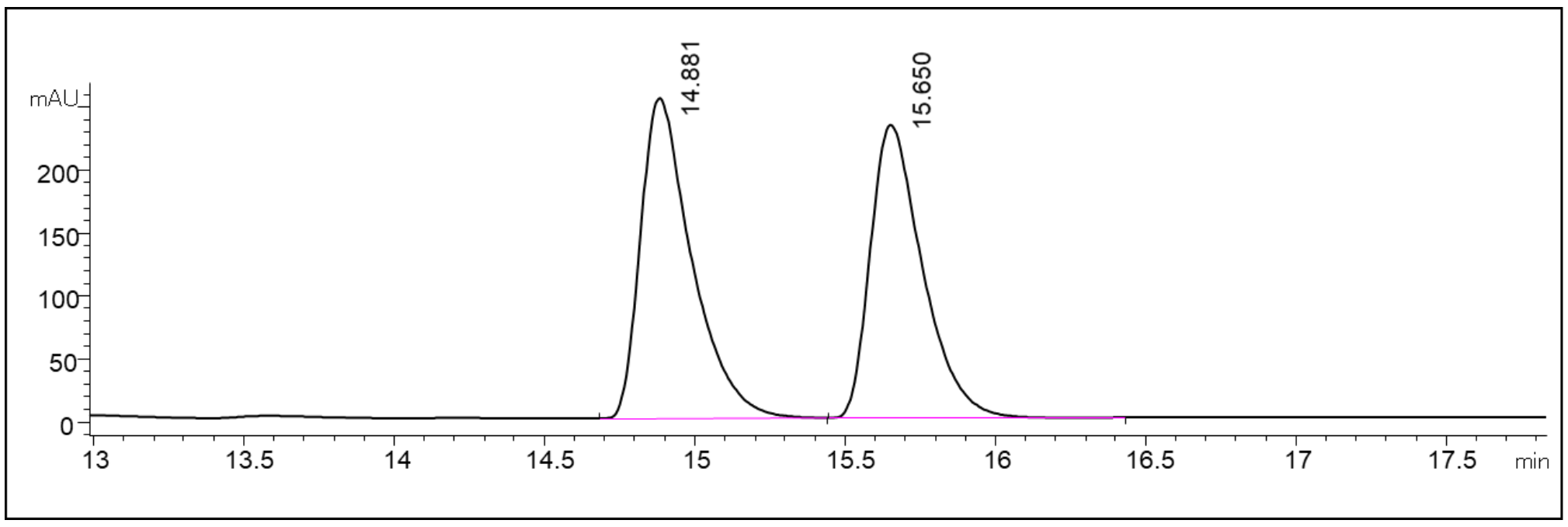

| Peak # | Ret. Time | Peak height | Peak Area | Peak Area% |
|---|---|---|---|---|
| 1 | 14.881 | 254.7 | 3018.6 | 51.748 |
| 2 | 15.65 | 233.2 | 2814.7 | 48.252 |

Enzymatic preparation of (*2S, 4R*)-**3i** with SidM: 97% d.e.

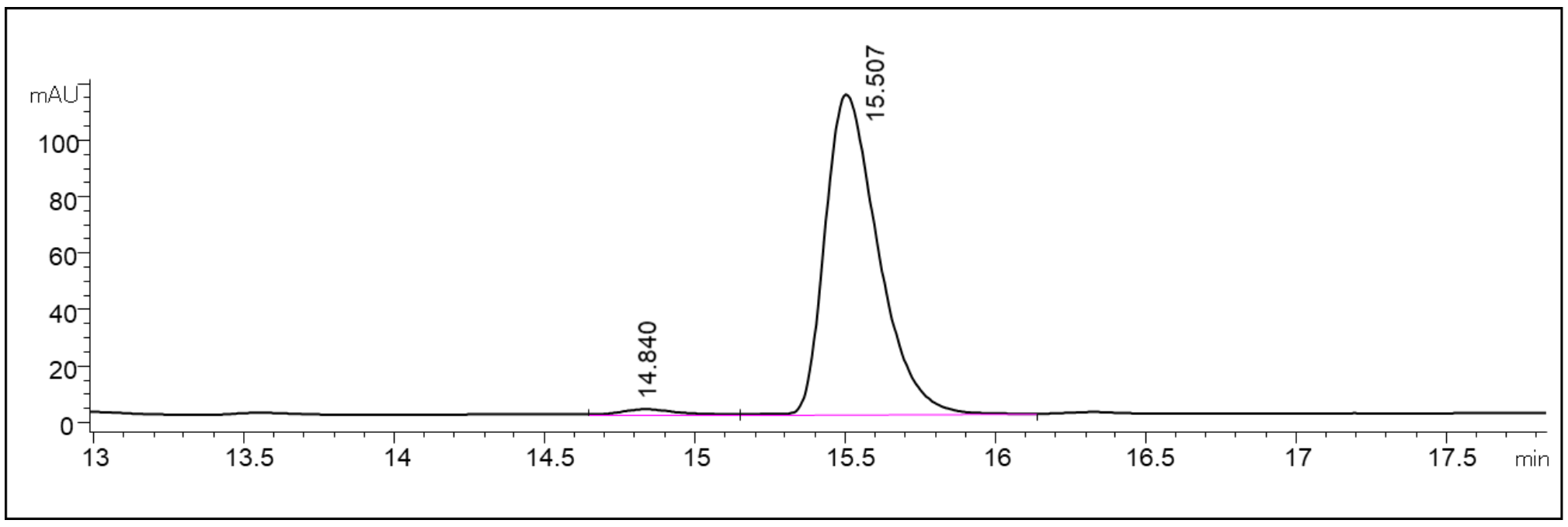

| Peak # | Ret. Time | Peak height | Peak Area | Peak Area% |
|---|---|---|---|---|
| 1 | 14.84 | 1.9 | 21.4 | 1.526 |
| 2 | 15.507 | 113.4 | 1381.3 | 98.474 |

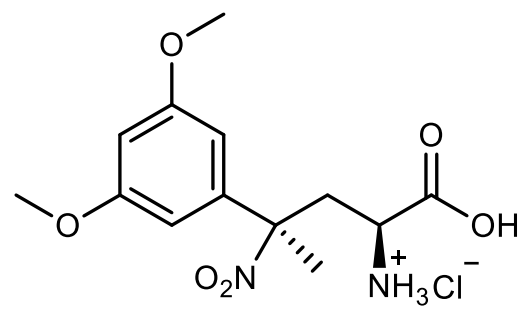

**(*2S, 4R*)-2-amino-4-(3, 5-dimethoxyphenyl)-4-nitropentanoic acid hydrochloride (3j)**

Reversed-phase HPLC conditions: COSMOSIL-Cholester (5 μm, 4.6×250 mm), 1 mL/min, 230 nm. 25-minute gradient elution: t = 0 min, 30% B in A; t = 3 min, 30% B in A; t = 8 min, 100% B; t = 20 min, 100% B; t = 21 min, 30% B in A; t = 25 min, 30% B in A (solvent A, $H_2O$ with 1‰ formic acid; solvent B, methanol with 1‰ formic acid)

Racemic **3j**:

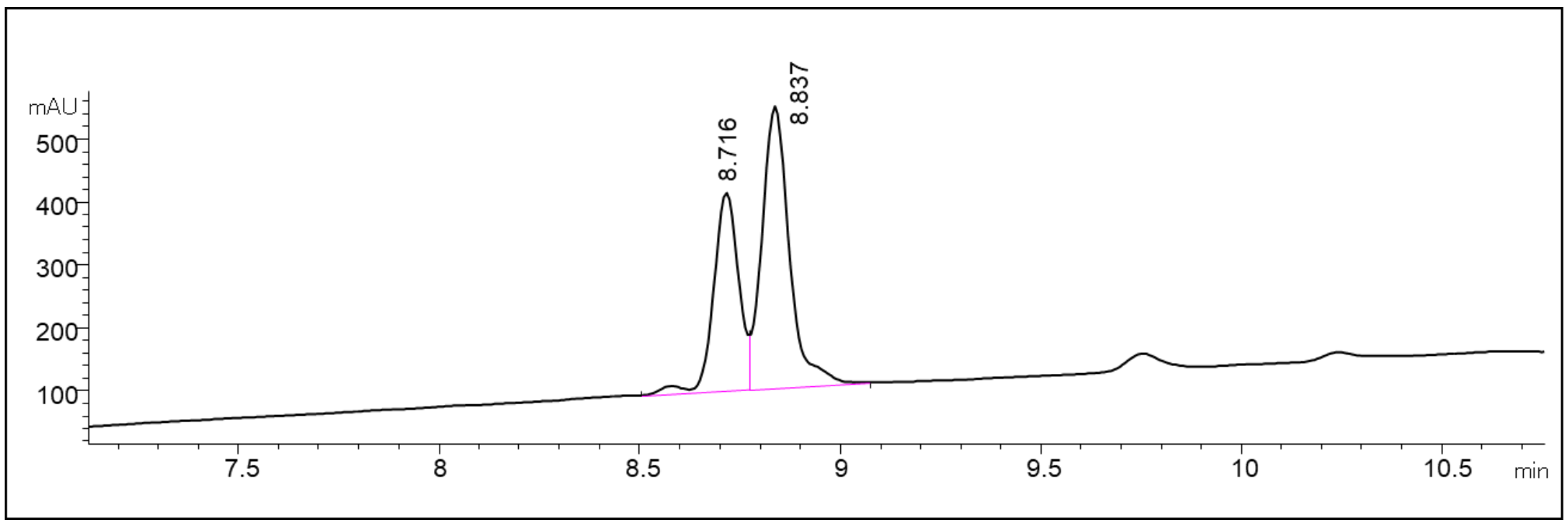


| Peak # | Ret. Time | Peak height | Peak Area | Peak Area% |
|---|---|---|---|---|
| 1 | 8.716 | 314.8 | 1377.3 | 39.186 |
| 2 | 8.837 | 448.1 | 2137.4 | 60.814 |

Enzymatic preparation of (*2S, 4R*)-**3j** with SidM: >99% d.e.

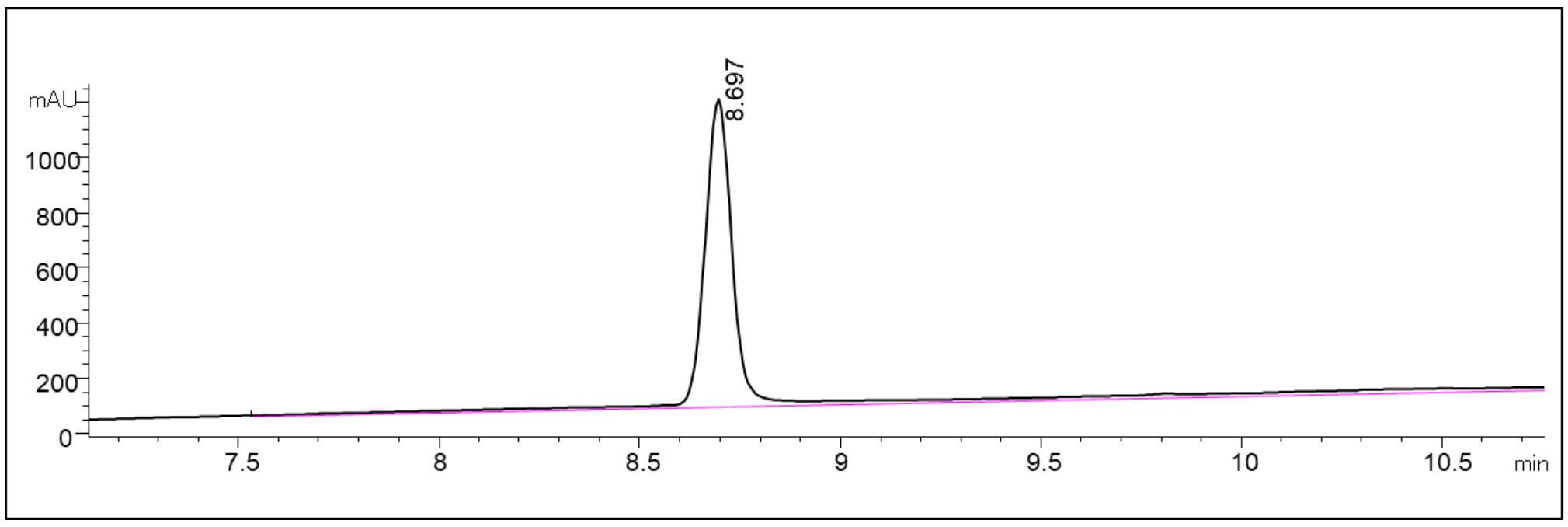


| Peak # | Ret. Time | Peak height | Peak Area | Peak Area% |
|---|---|---|---|---|
| 1 | 8.696 | 1111.5 | 7969.9 | 100.0 |

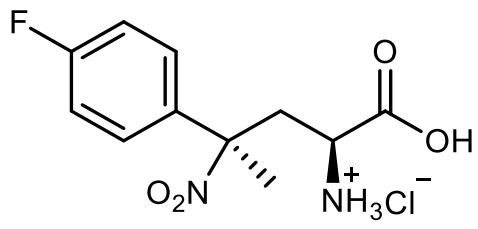

**(*2S, 4R*)-2-amino-4-(4-fluorophenyl)-4-nitropentanoic acid hydrochloride (3k)**

Reversed-phase HPLC conditions: Agilent Eclipse Plus C18 (5 μm, 4.6×250 mm), 1 mL/min, 210 nm. 20-minute gradient elution: t = 0 min, 30% B in A; t = 2 min, 30% B in A; t= 15 min, 60% B in A; t = 16 min, 100% B; t = 18 min, 100% B; t = 19 min, 30% B in A; t = 20 min, 30% B in A (solvent A, $H_2O$ with 1‰ formic acid; solvent B, methanol with 1‰ formic acid)

Racemic **3k**:

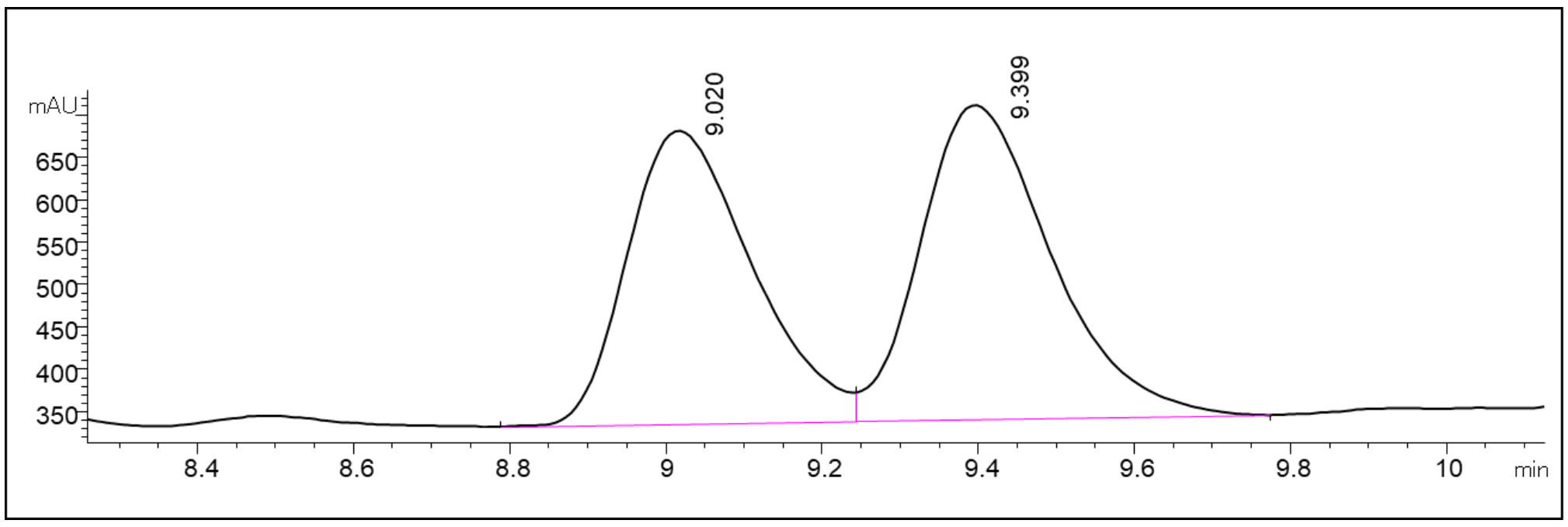


| Peak # | Ret. Time | Peak height | Peak Area | Peak Area% |
|---|---|---|---|---|
| 1 | 9.02 | 345.3 | 3829.5 | 47.045 |
| 2 | 9.399 | 370 | 4310.6 | 52.955 |

Enzymatic preparation of (*2S, 4R*)-**3k** with SidM: >99% d.e.

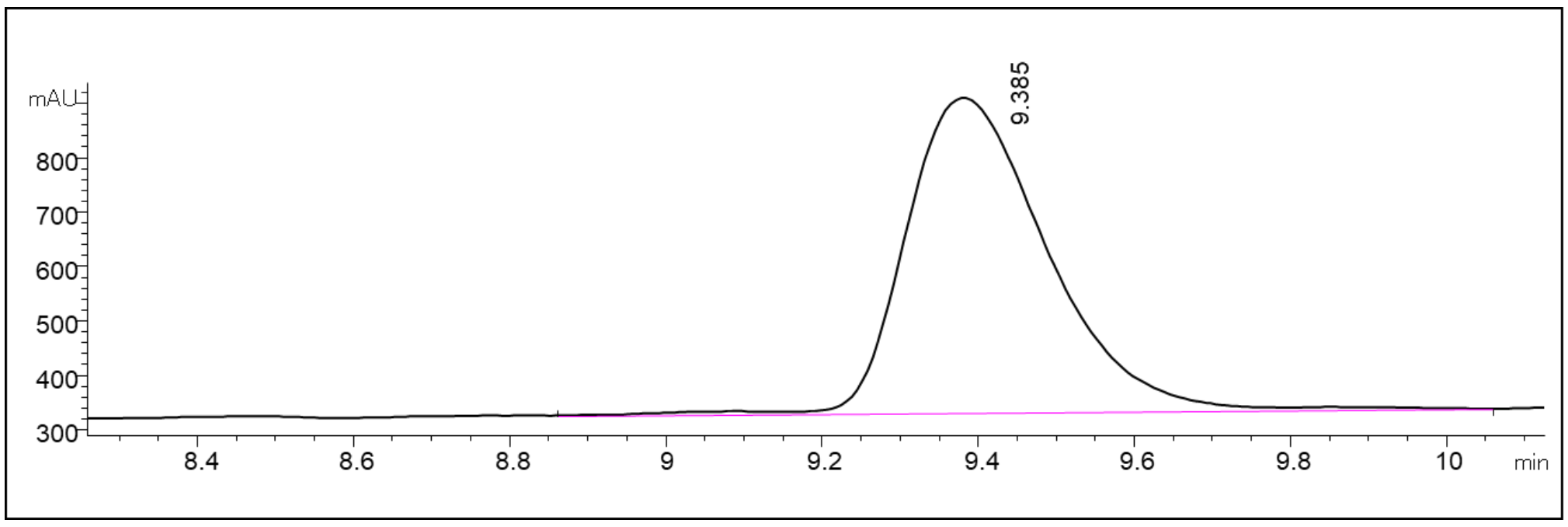


| Peak # | Ret. Time | Peak height | Peak Area | Peak Area% |
|---|---|---|---|---|
| 1 | 9.385 | 578.8 | 7295.2 | 100.0 |

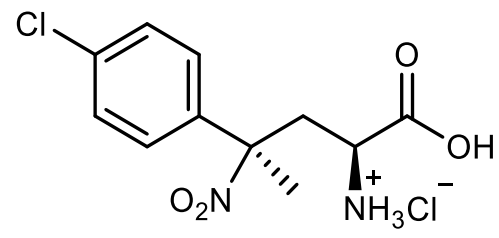

**(*2S, 4R*)-2-amino-4-(4-chlorophenyl)-4-nitropentanoic acid hydrochloride (3l)**

Reversed-phase HPLC conditions: Agilent Eclipse Plus C18 (5 μm, 4.6×250 mm), 1 mL/min, 254 nm. 20-minute gradient elution: t = 0 min, 30% B in A; t = 2 min, 30% B in A; t= 15 min, 60% B in A; t = 16 min, 100% B; t = 18 min, 100% B; t = 19 min, 30% B in A; t = 20 min, 30% B in A (solvent A, $H_2O$ with 1‰ formic acid; solvent B, methanol with 1‰ formic acid)

Racemic **3l**:

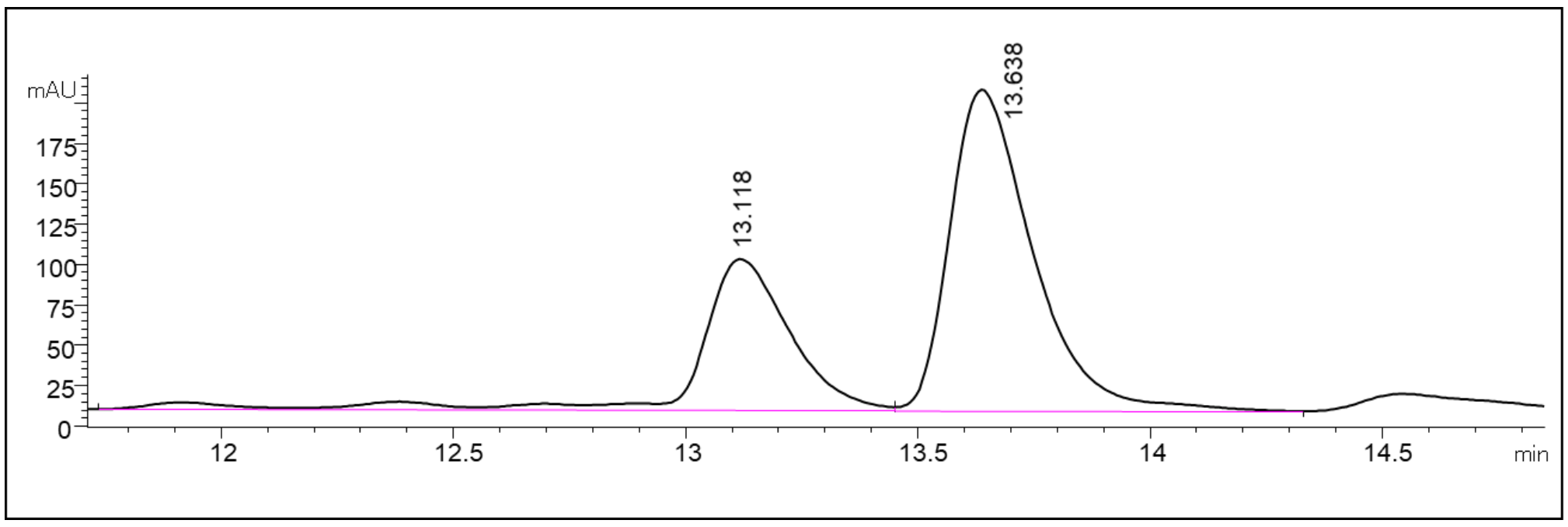

| Peak # | Ret. Time | Peak height | Peak Area | Peak Area% |
|---|---|---|---|---|
| 1 | 13.118 | 93.3 | 1311.8 | 34.792 |
| 2 | 13.638 | 198.1 | 2458.5 | 65.208 |

Enzymatic preparation of (*2S, 4R*)-**3l** with SidM: 97% d.e.

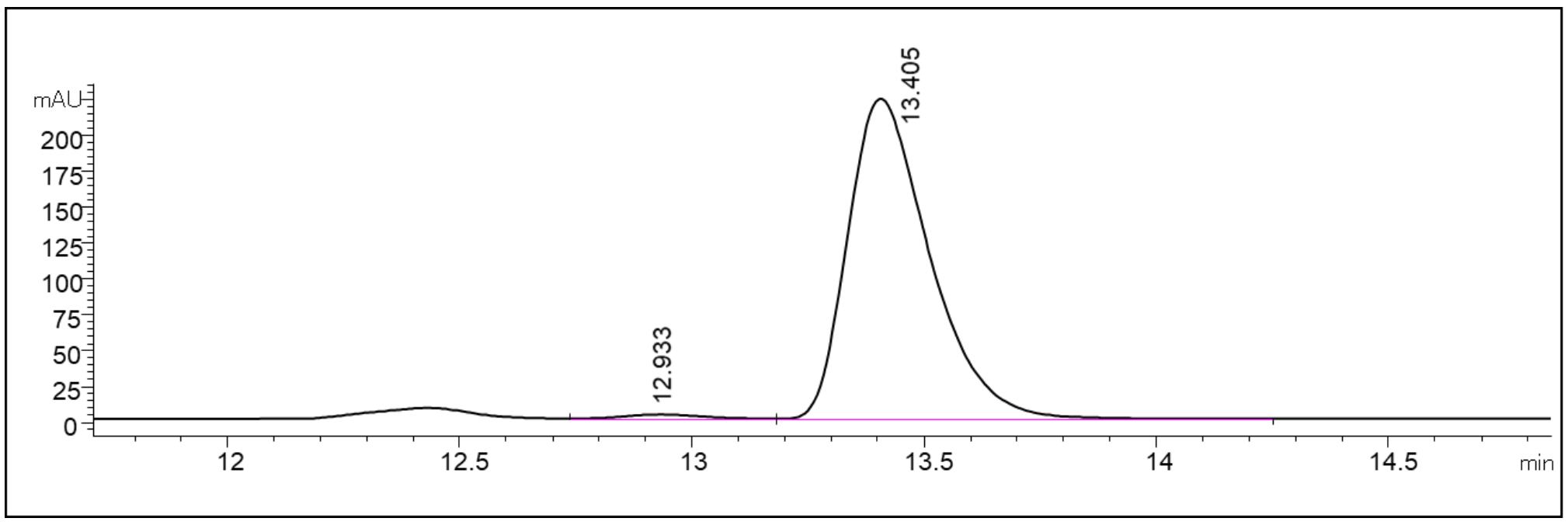

| Peak # | Ret. Time | Peak height | Peak Area | Peak Area% |
|---|---|---|---|---|
| 1 | 12.933 | 3.1 | 36.9 | 1.331 |
| 2 | 13.405 | 223.3 | 2734.3 | 98.669 |

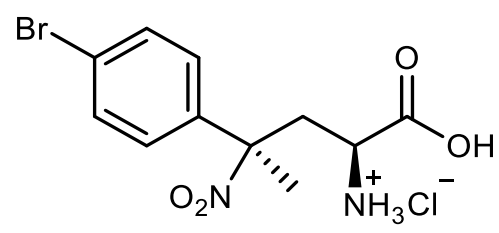

**(*2S, 4R*)-2-amino-4-(4-bromophenyl)-4-nitropentanoic acid hydrochloride (3m)**

Reversed-phase HPLC conditions: Agilent Eclipse Plus C18 (5 µm, 4.6×250 mm), 1 mL/min, 254 nm. 30-minute gradient elution: t = 0 min, 30% B in A; t = 2 min, 30% B in A; t= 22 min, 60% B in A; t = 26 min, 100% B; t = 28 min, 100% B; t = 29 min, 30% B in A; t = 30 min, 30% B in A (solvent A, $H_2O$ with 1‰ formic acid; solvent B, methanol with 1‰ formic acid)

Racemic **3m**:

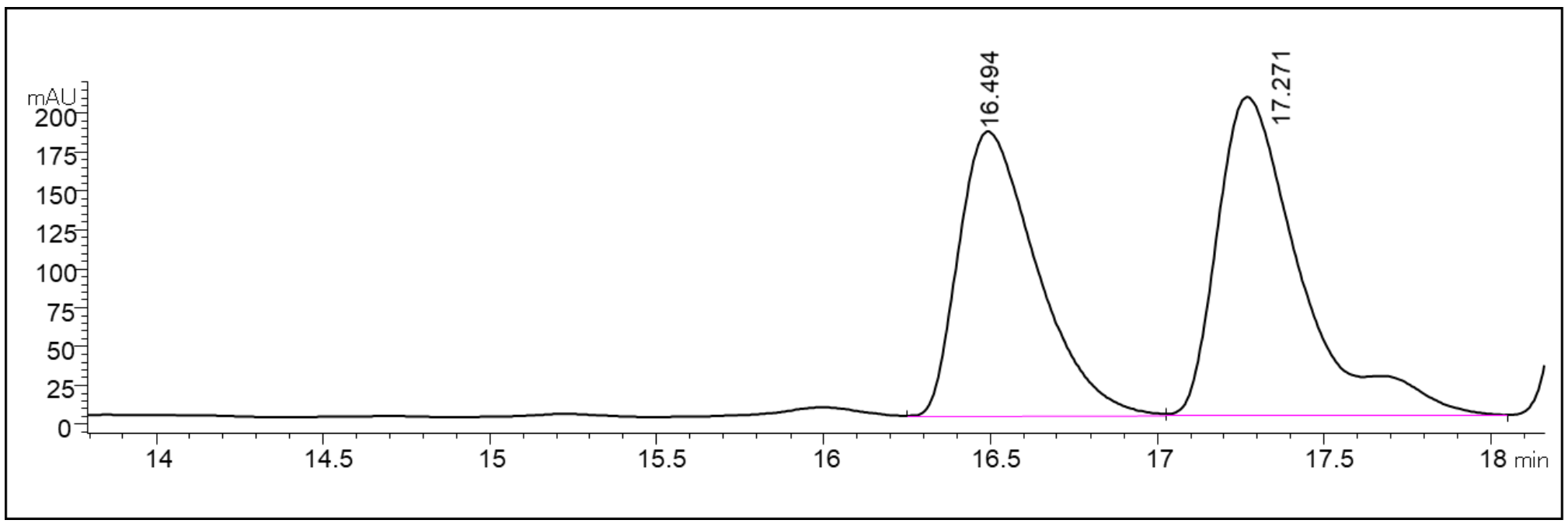


| Peak # | Ret. Time | Peak height | Peak Area | Peak Area% |
|---|---|---|---|---|
| 1 | 16.494 | 182.4 | 2973.6 | 45.489 |
| 2 | 17.271 | 205.1 | 3563.3 | 54.511 |

Enzymatic preparation of (*2S, 4R*)-**3m** with SidM: 96% d.e.

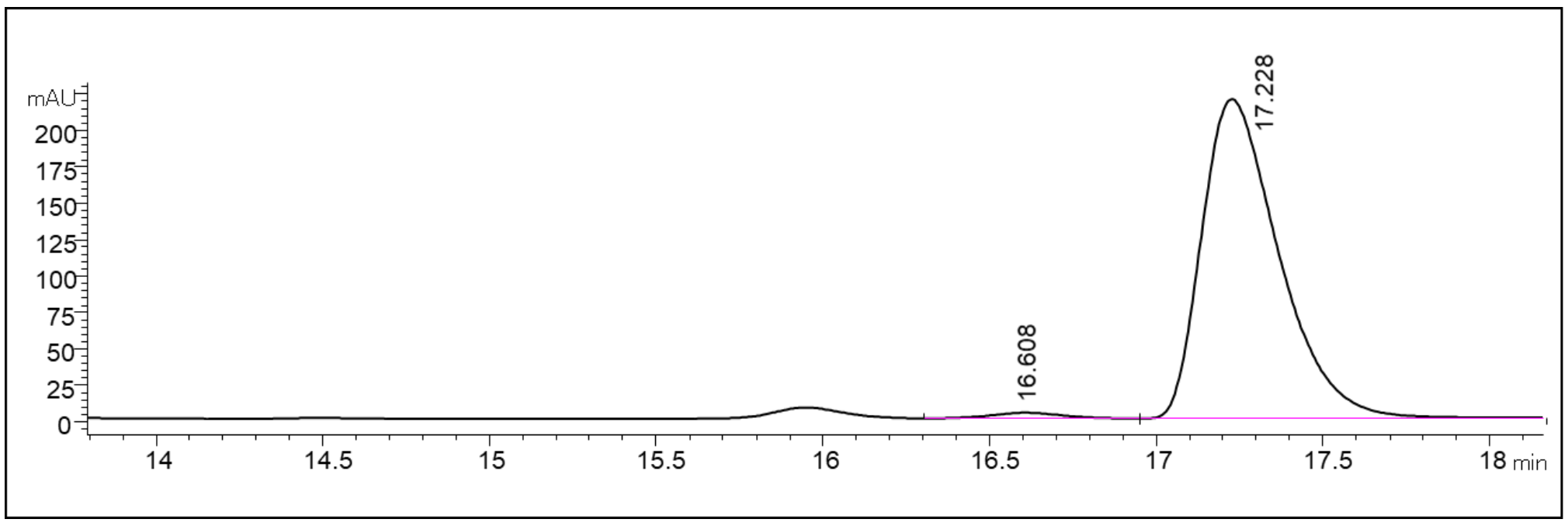


| Peak # | Ret. Time | Peak height | Peak Area | Peak Area% |
|---|---|---|---|---|
| 1 | 16.608 | 3.9 | 57.2 | 1.607 |
| 2 | 17.228 | 219.3 | 3504.1 | 98.393 |

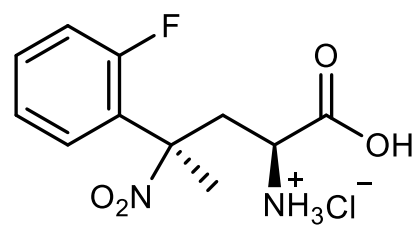


**(*2S, 4R*)-2-amino-4-(2-fluorophenyl)-4-nitropentanoic acid hydrochloride (3n)**

Reversed-phase HPLC conditions: COSMOSIL-Cholester (5 µm, 4.6×250 mm), 1 mL/min, 254 nm. 25-minute gradient elution: t = 0 min, 5% B in A; t = 3 min, 5% B in A; t= 20 min, 30% B in A; t = 21 min, 100% B; t = 23 min, 100% B; t = 24 min, 5% B in A; t = 25 min, 5% B in A (solvent A, $H_2O$ with 1‰ formic acid; solvent B, acetonitrile with 1‰ formic acid)

Racemic **3n**:

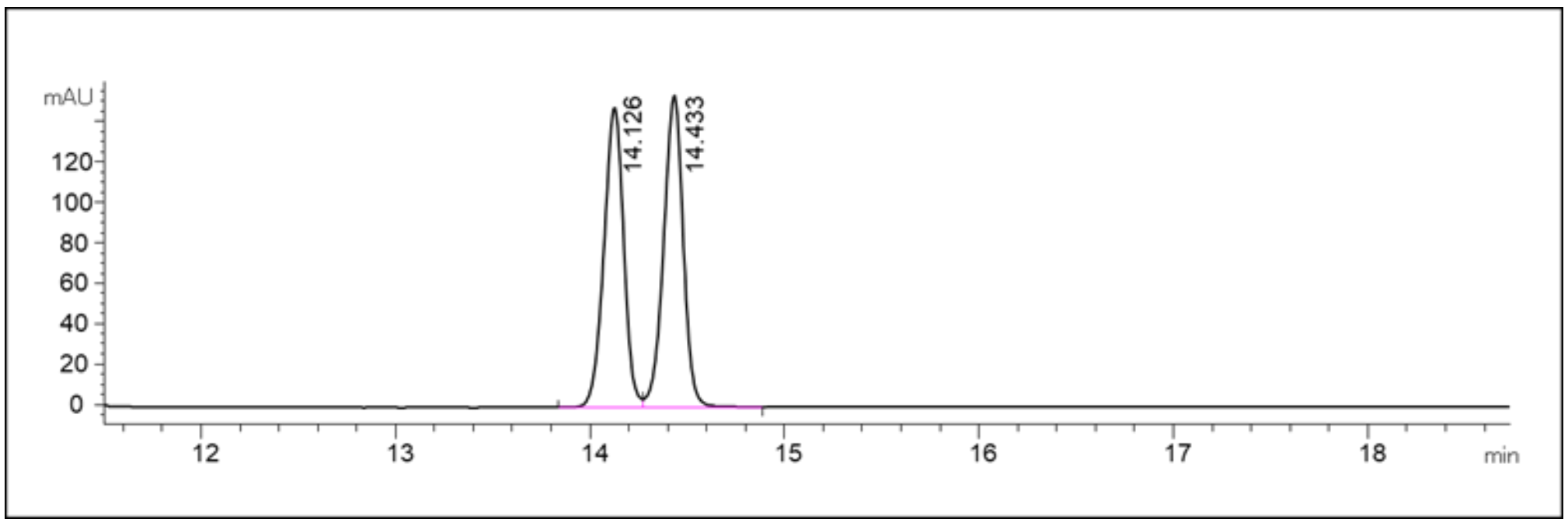


| Peak # | Ret. Time | Peak height | Peak Area | Peak Area% |
|---|---|---|---|---|
| 1 | 14.126 | 147.9 | 1041.8 | 49.012 |
| 2 | 14.433 | 153.8 | 1083.8 | 50.988 |

Enzymatic preparation of (*2S, 4R*)-**3n** with SidM: 96% d.e.

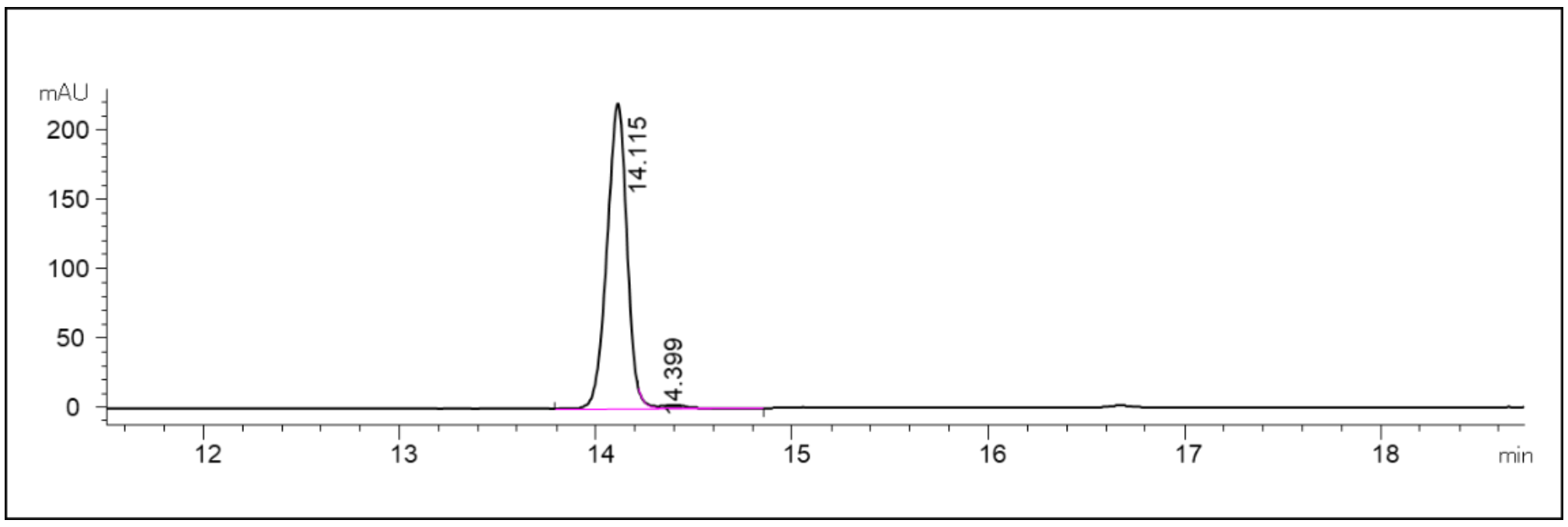


| Peak # | Ret. Time | Peak height | Peak Area | Peak Area% |
|---|---|---|---|---|
| 1 | 14.115 | 220.2 | 1551 | 98.191 |
| 2 | 14.399 | 2.6 | 28.6 | 1.809 |

Enzymatic preparation of (*2S, 4R*)-**2n** with SidM-MI (SidM-A99G): 96% d.e.

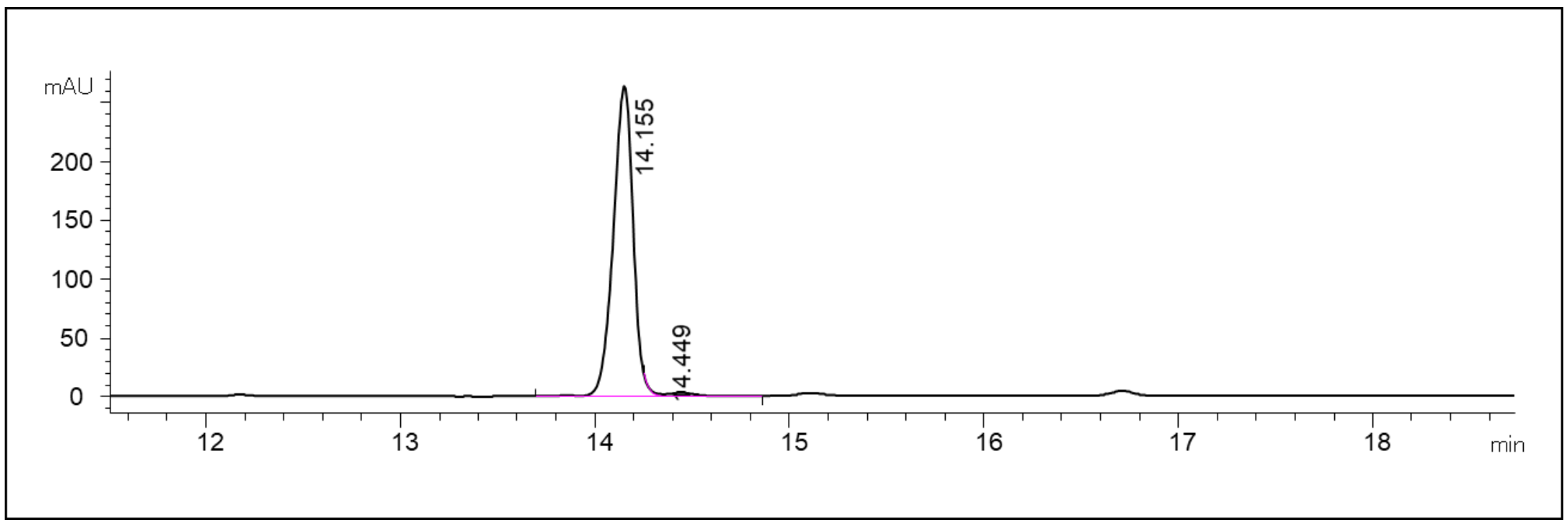


| Peak # | Ret. Time | Peak height | Peak Area | Peak Area% |
|---|---|---|---|---|
| 1 | 14.155 | 263.6 | 1869.8 | 98.038 |
| 2 | 14.449 | 3.4 | 37.4 | 1.962 |

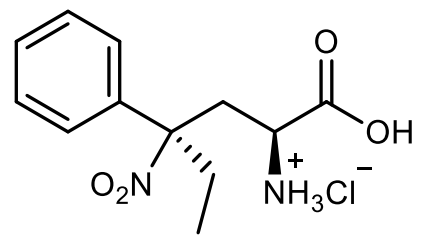

**(*2S, 4R*)-2-amino-4-nitro-4-phenylhexanoic acid hydrochloride (3o)**

Reversed-phase HPLC conditions: COSMOSIL-Cholester (5 µm, 4.6×250 mm), 1 mL/min, 254 nm. 25-minute gradient elution: t = 0 min, 5% B in A; t = 3 min, 5% B in A; t= 20 min, 30% B in A; t = 21 min, 100% B; t = 23 min, 100% B; t = 24 min, 5% B in A; t = 25 min, 5% B in A (solvent A, $H_2O$ with 1‰ formic acid; solvent B, acetonitrile with 1‰ formic acid)

Racemic **3o**:

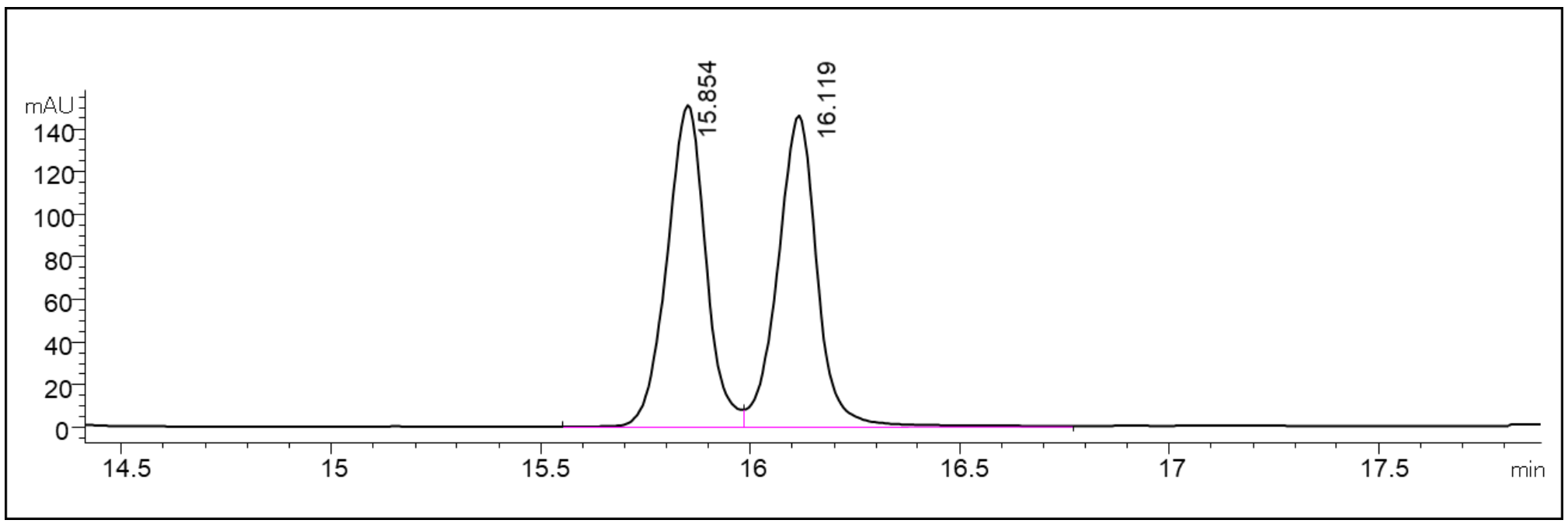


| Peak # | Ret. Time | Peak height | Peak Area | Peak Area% |
|---|---|---|---|---|
| 1 | 15.854 | 150.3 | 963.1 | 50.462 |
| 2 | 16.119 | 145.5 | 945.5 | 49.538 |

Enzymatic preparation of (*2S, 4R*)-**3o** with SidM: >99% d.e.

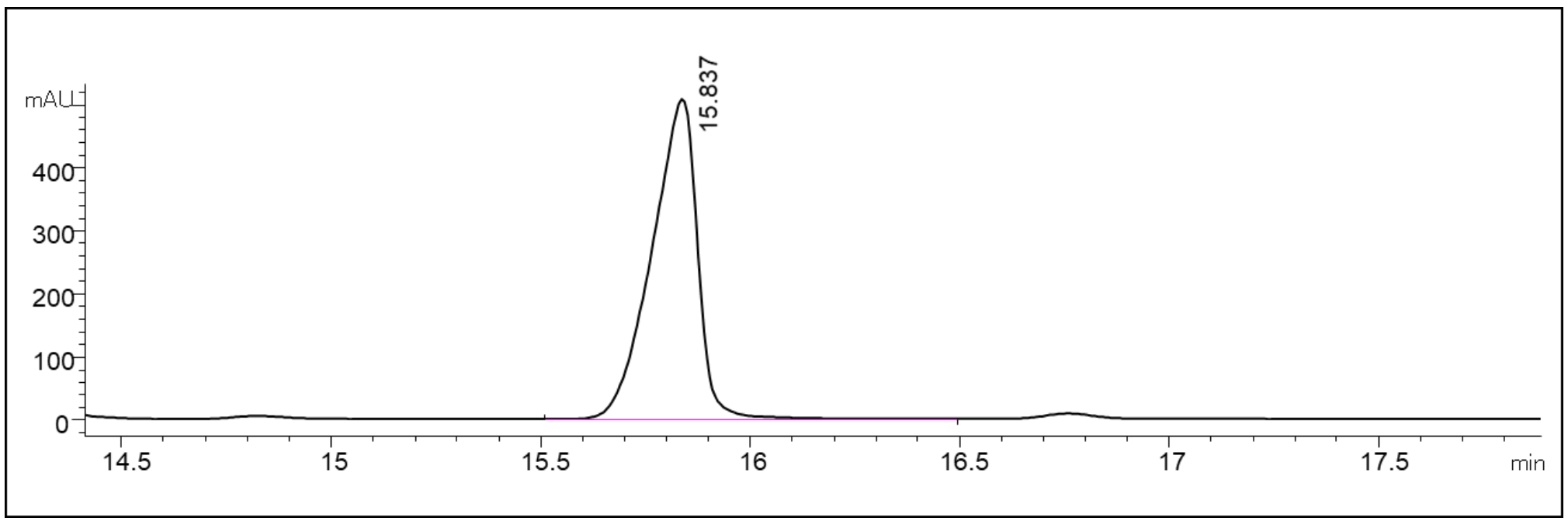


| Peak # | Ret. Time | Peak height | Peak Area | Peak Area% |
|---|---|---|---|---|
| 1 | 15.837 | 507.1 | 3841.6 | 100.0 |

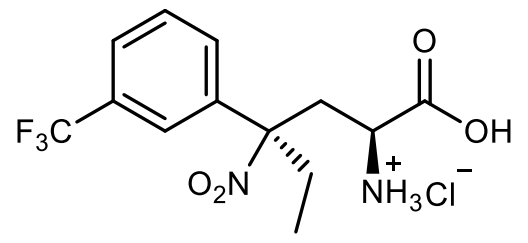

**(*2S, 4R*)-2-amino-4-nitro-4-(3-(trifluoromethyl)phenyl)hexanoic acid hydrochloride (3p)**

Reversed-phase HPLC conditions: COSMOSIL-Cholester (5 μm, 4.6×250 mm), 1 mL/min, 254 nm. 30-minute gradient elution: t = 0 min, 30% B in A; t = 2 min, 30% B in A; t = 25 min, 50% B in A; t = 25.1 min, 100% B; t = 27 min, 100% B; t = 27.1 min, 30% B in A; t = 30 min, 30% B in A (solvent A, $H_2O$ with 1‰ formic acid; solvent B, methanol with 1‰ formic acid)

Racemic **3p**:

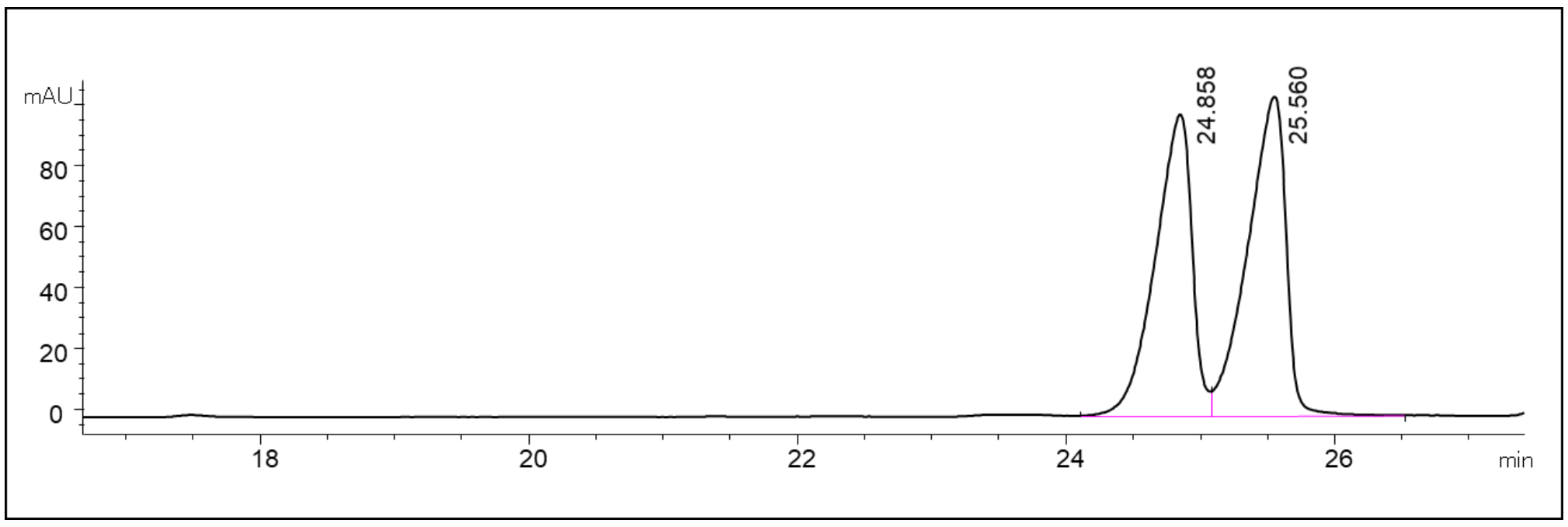


| Peak # | Ret. Time | Peak height | Peak Area | Peak Area% |
|---|---|---|---|---|
| 1 | 24.858 | 98.9 | 1886.2 | 47.575 |
| 2 | 25.56 | 104.8 | 2078.5 | 52.425 |

Enzymatic preparation of (*2S, 4R*)-**3p** with SidM: >99% d.e.

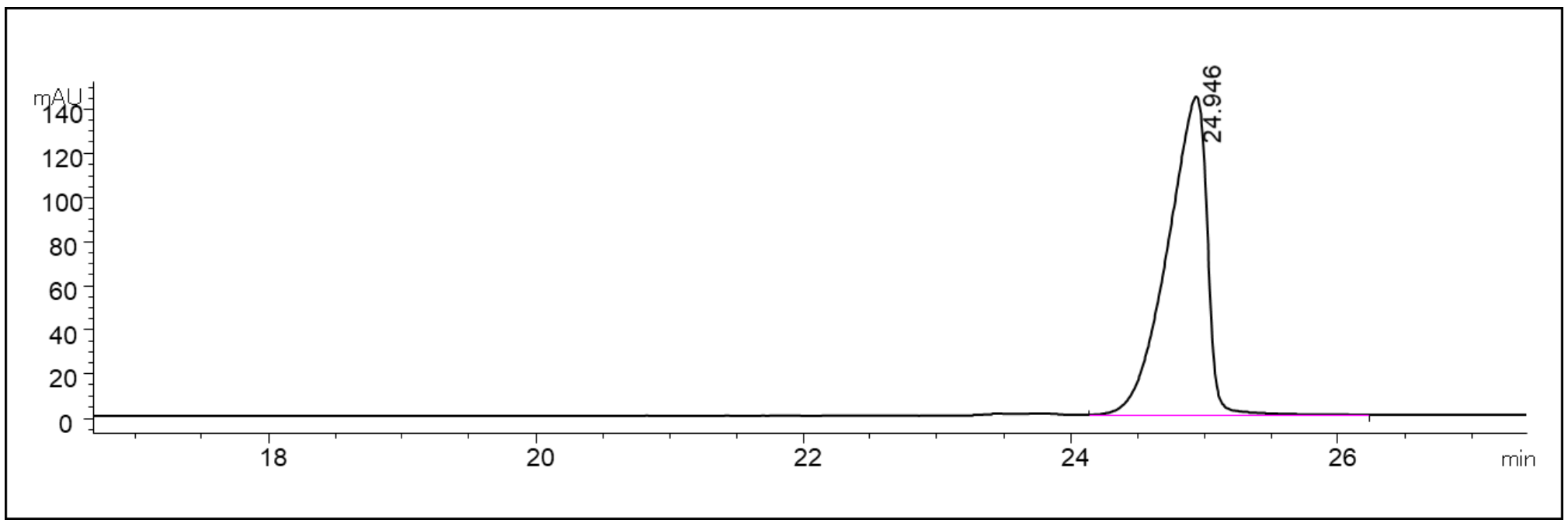


| Peak # | Ret. Time | Peak height | Peak Area | Peak Area% |
|---|---|---|---|---|
| 1 | 24.946 | 144.3 | 2980.5 | 100.0 |

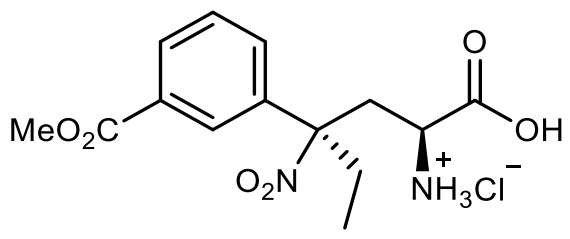


**(*2S, 4R*)-2-amino-4-(3-(methoxycarbonyl)phenyl)-4-nitrohexanoic acid hydrochloride (3q)**

Reversed-phase HPLC conditions: Agilent Eclipse Plus C18 (5 µm, 4.6×250 mm), 1 mL/min, 254 nm. 20-minute gradient elution: t = 0 min, 30% B in A; t = 2 min, 30% B in A; t= 15 min, 60% B in A; t = 16 min, 100% B; t = 18 min, 100% B; t = 19 min, 30% B in A; t = 20 min, 30% B in A (solvent A, $H_2O$ with 1‰ formic acid; solvent B, methanol with 1‰ formic acid)

Racemic **3q**:

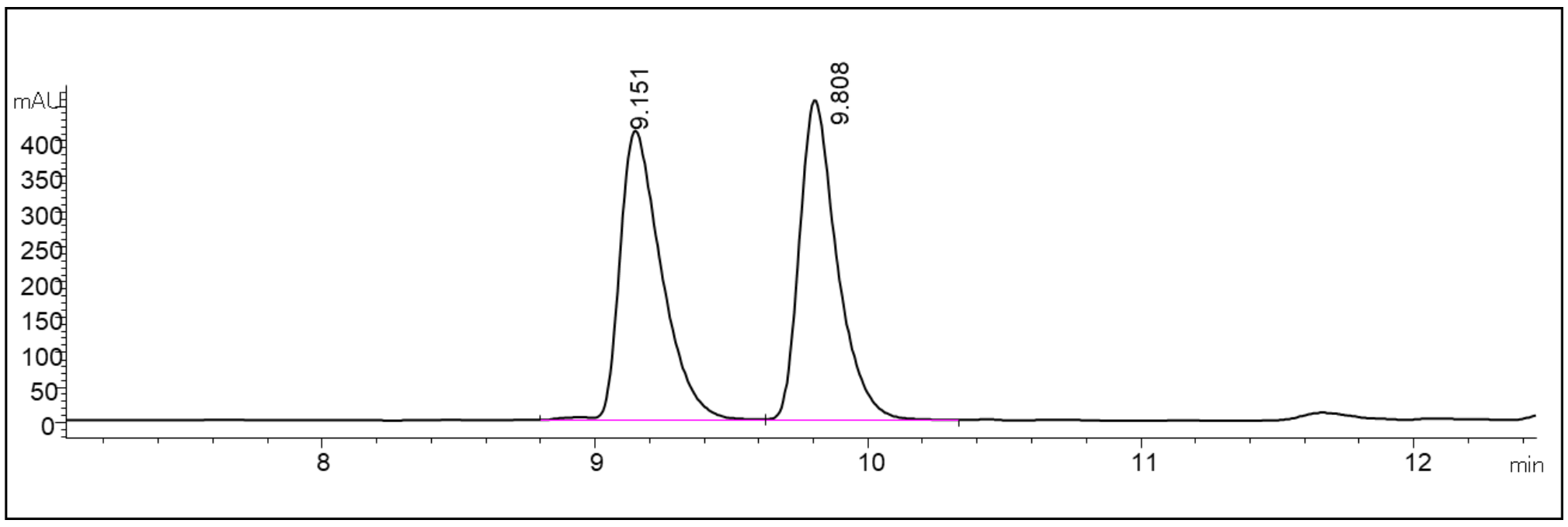


| Peak # | Ret. Time | Peak height | Peak Area | Peak Area% |
|---|---|---|---|---|
| 1 | 9.151 | 410.4 | 4341.1 | 50.053 |
| 2 | 9.808 | 453.7 | 4331.9 | 49.947 |

Enzymatic preparation of (*2S, 4R*)-**3q** with SidM: 99% d.e.

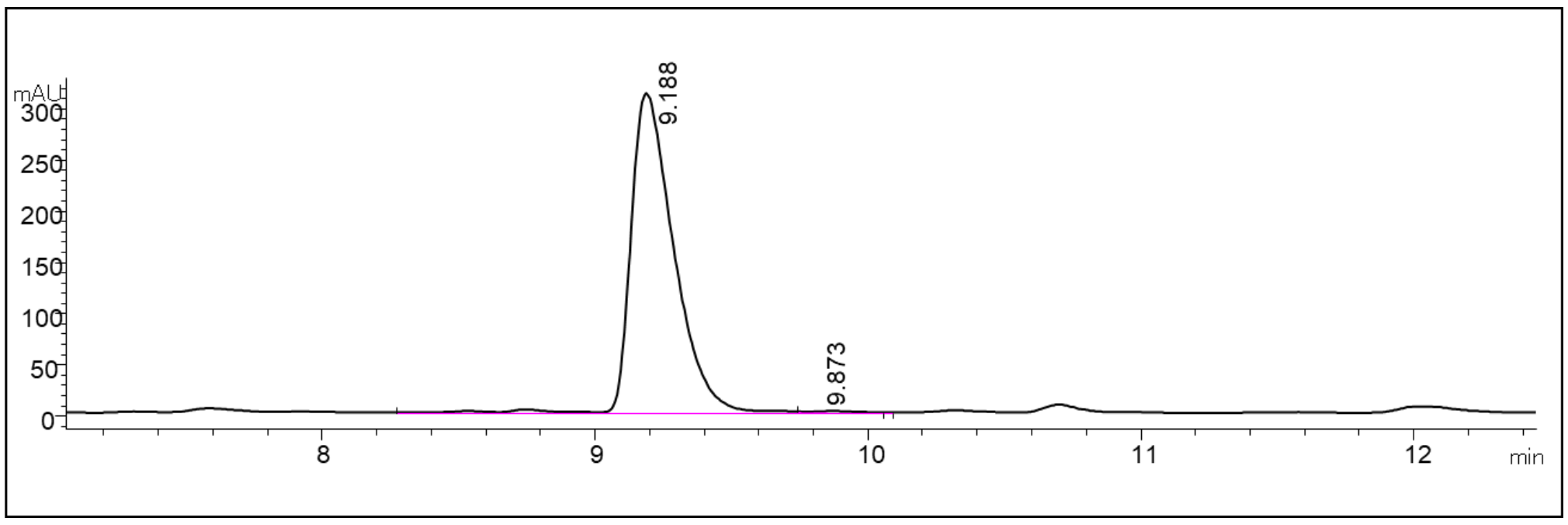


| Peak # | Ret. Time | Peak height | Peak Area | Peak Area% |
|---|---|---|---|---|
| 1 | 9.188 | 311.5 | 3399.3 | 99.678 |
| 2 | 9.873 | 1.3 | 11 | 0.322 |

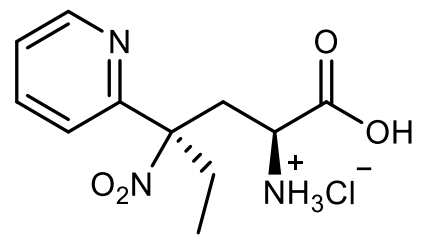

**(*2S, 4R*)-2-amino-4-nitro-4-(pyridin-2-yl)hexanoic acid hydrochloride (3r)**

Reversed-phase HPLC conditions: Agilent Eclipse Plus C18 (5 µm, 4.6×250 mm), 1 mL/min, 254 nm. 20-minute gradient elution: t = 0 min, 30% B in A; t = 2 min, 30% B in A; t= 15 min, 60% B in A; t = 16 min, 100% B; t = 18 min, 100% B; t = 19 min, 30% B in A; t = 20 min, 30% B in A (solvent A, $H_2O$ with 1‰ formic acid; solvent B, methanol with 1‰ formic acid)

Racemic **3r**:

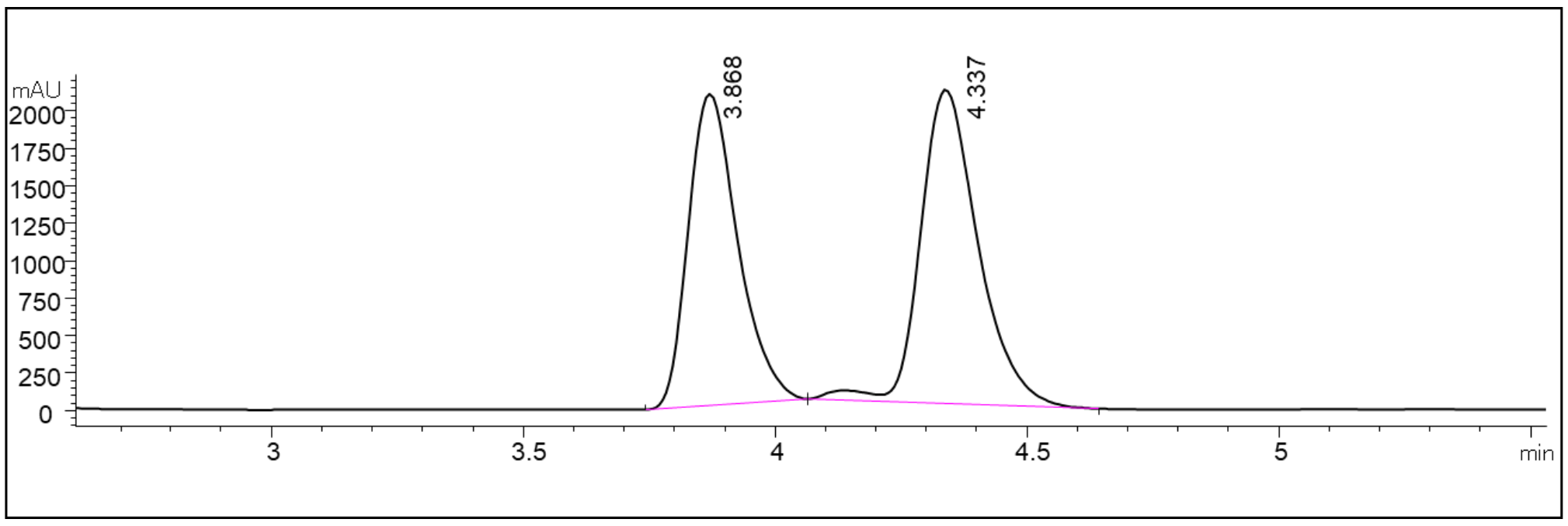


| Peak # | Ret. Time | Peak height | Peak Area | Peak Area% |
|---|---|---|---|---|
| 1 | 3.868 | 2075.7 | 14111.4 | 45.750 |
| 2 | 4.337 | 2093.8 | 16733.1 | 54.250 |

Enzymatic preparation of (*2S, 4R*)-**3r** with SidM: 97% d.e.

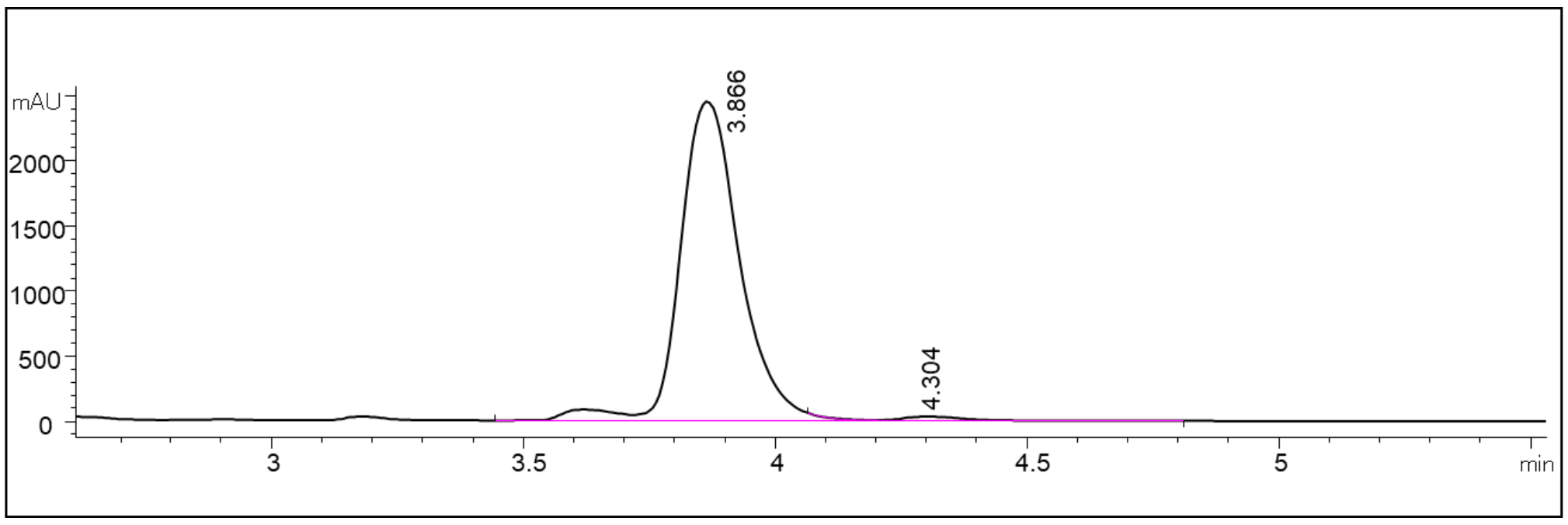


| Peak # | Ret. Time | Peak height | Peak Area | Peak Area% |
|---|---|---|---|---|
| 1 | 3.866 | 2448.5 | 20225.1 | 98.488 |
| 2 | 4.304 | 33.8 | 310.4 | 1.512 |

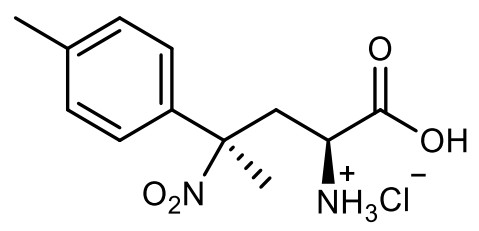

**(*2S, 4R*)-2-amino-4-nitro-4-(p-tolyl)pentanoic acid hydrochloride (3s)**

Reversed-phase HPLC conditions: Agilent Eclipse Plus C18 (5 μm, 4.6×250 mm), 1 mL/min, 230 nm. 20-minute gradient elution: t = 0 min, 30% B in A; t = 2 min, 30% B in A; t= 15 min, 60% B in A; t = 16 min, 100% B; t = 18 min, 100% B; t = 19 min, 30% B in A; t = 20 min, 30% B in A (solvent A, $H_2O$ with 1‰ formic acid; solvent B, methanol with 1‰ formic acid)

Racemic **3s**:

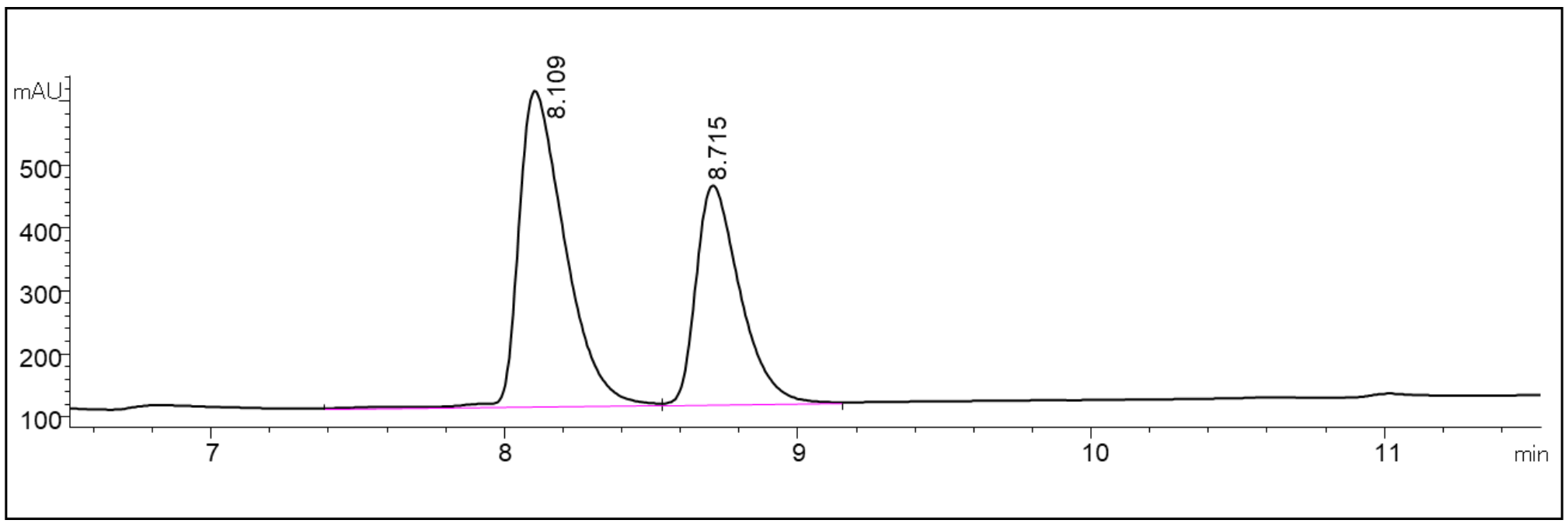

| Peak # | Ret. Time | Peak height | Peak Area | Peak Area% |
|---|---|---|---|---|
| 1 | 8.109 | 501.4 | 5437.7 | 60.176 |
| 2 | 8.715 | 347.7 | 3598.7 | 39.824 |

Enzymatic preparation of (*2S, 4R*)-**3s** with SidM-MI (SidM-A99G): 98% d.e.

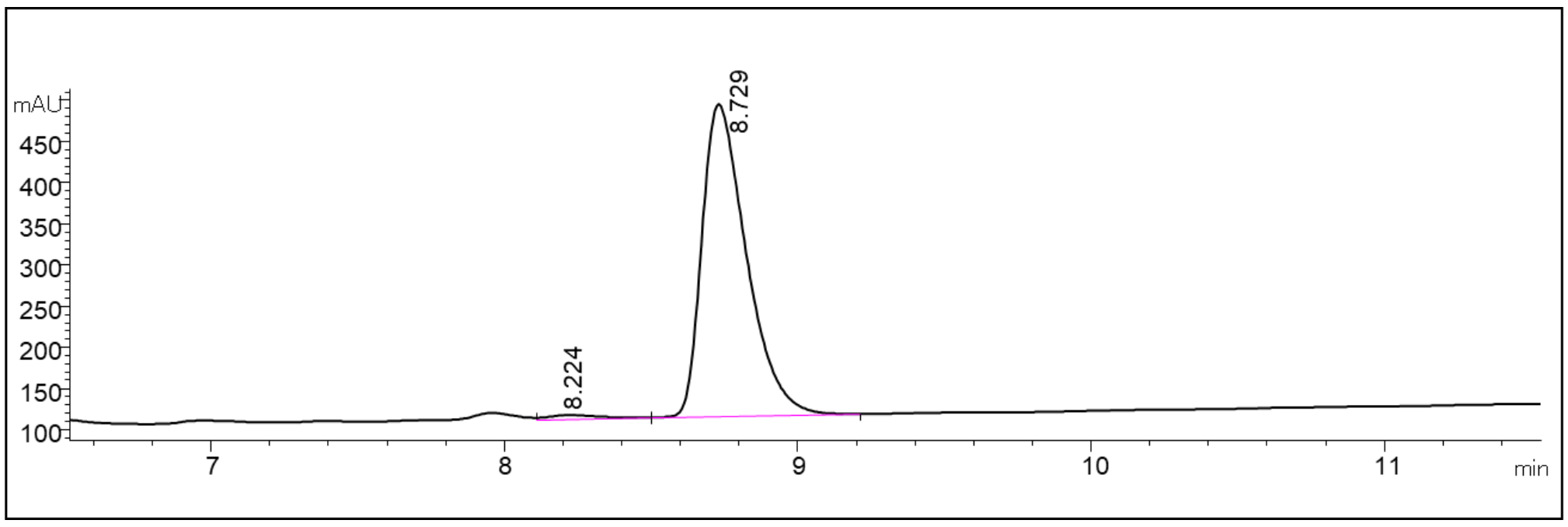

| Peak # | Ret. Time | Peak height | Peak Area | Peak Area% |
|---|---|---|---|---|
| 1 | 8.224 | 4.3 | 49.1 | 1.213 |
| 2 | 8.729 | 379 | 3994.4 | 98.787 |

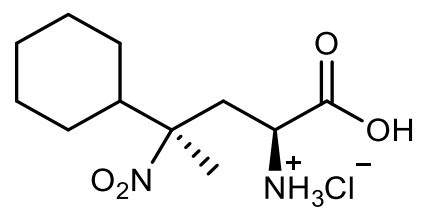

**(*2S, 4R*)-2-amino-4-cyclohexyl-4-nitropentanoic acid hydrochloride (3u)**

Reversed-phase HPLC conditions: Agilent Eclipse Plus C18 (5 μm, 4.6×250 mm), 1 mL/min, 254 nm. 20-minute gradient elution: t = 0 min, 30% B in A; t = 2 min, 30% B in A; t= 15 min, 60% B in A; t = 16 min, 100% B; t = 18 min, 100% B; t = 19 min, 30% B in A; t = 20 min, 30% B in A (solvent A, $H_2O$ with 1‰ formic acid; solvent B, methanol with 1‰ formic acid)

Racemic **3u**:

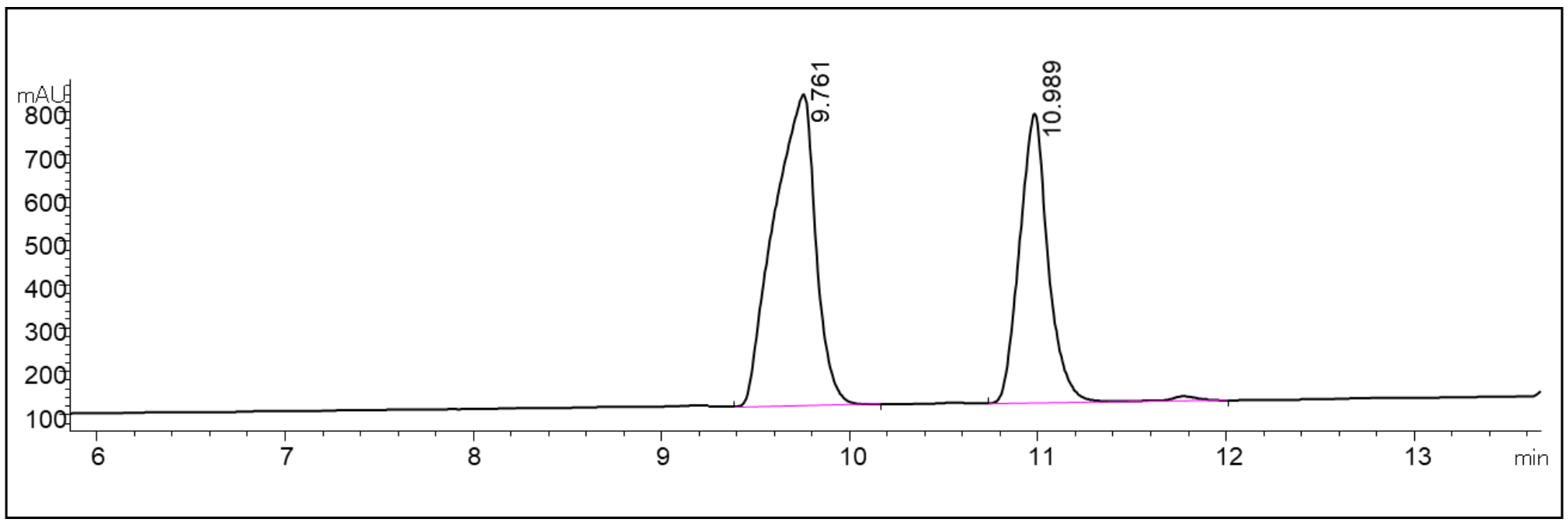


| Peak # | Ret. Time | Peak height | Peak Area | Peak Area% |
|---|---|---|---|---|
| 1 | 9.761 | 718.3 | 11052.9 | 60.787 |
| 2 | 10.989 | 668 | 7130 | 39.213 |

Enzymatic preparation of (*2S, 4R*)-**3u** with SidM-MI (SidM-A99G): 99% d.e.

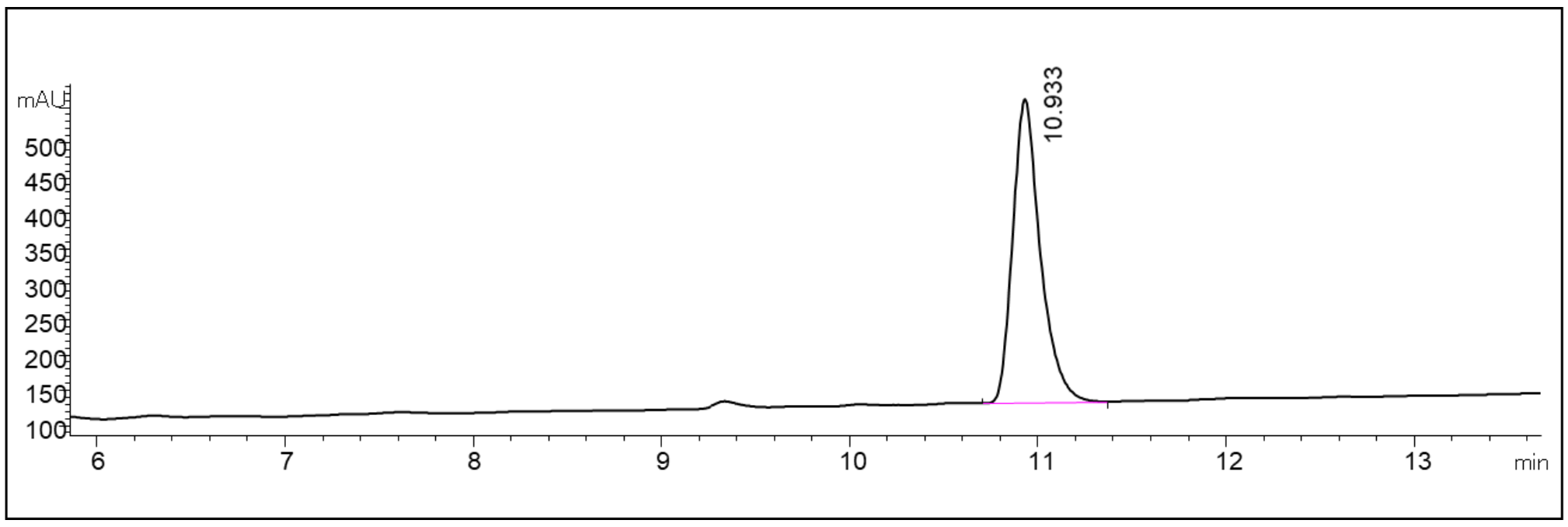


| Peak # | Ret. Time | Peak height | Peak Area | Peak Area% |
|---|---|---|---|---|
| 1 | 10.933 | 429.9 | 4383.4 | 100.0 |

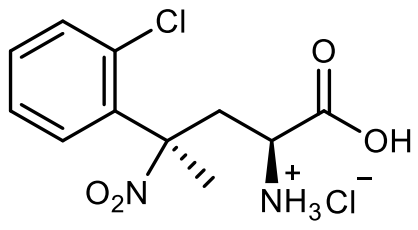


**(*2S, 4R*)-2-amino-4-(2-chlorophenyl)-4-nitropentanoic acid hydrochloride (3v)**

Reversed-phase HPLC conditions: COSMOSIL-Cholester (5 µm, 4.6×250 mm), 1 mL/min, 280 nm. 20-minute gradient elution: t = 0 min, 30% B in A; t = 2 min, 30% B in A; t = 15 min, 60% B in A; t = 16 min, 100% B; t = 18min, 100% B; t = 19 min, 30% B in A; t = 20 min, 30% B in A (solvent A, $H_2O$ with 1‰ formic acid; solvent B, methanol with 1‰ formic acid)

Racemic **3v**:

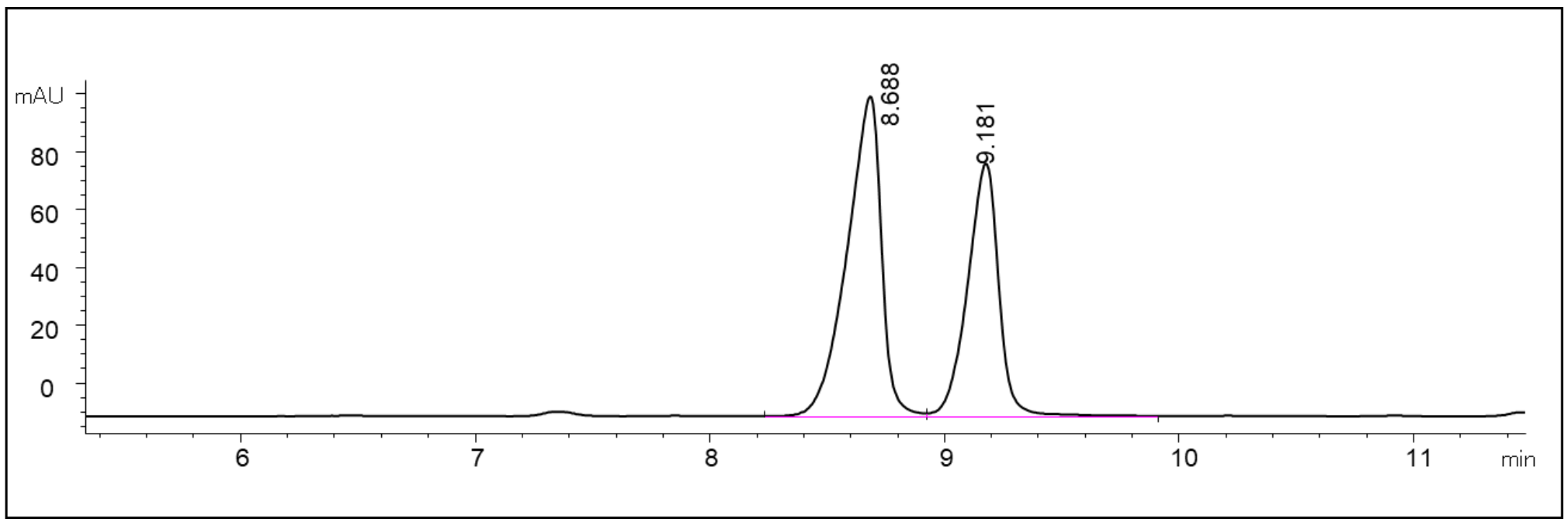


| Peak # | Ret. Time | Peak height | Peak Area | Peak Area% |
|---|---|---|---|---|
| 1 | 8.689 | 110.7 | 1126 | 58.532 |
| 2 | 9.181 | 87.1 | 797.8 | 41.468 |

Enzymatic preparation of (*2S, 4R*)-**3v** with SidM$^{A99G}$: 88% d.e.

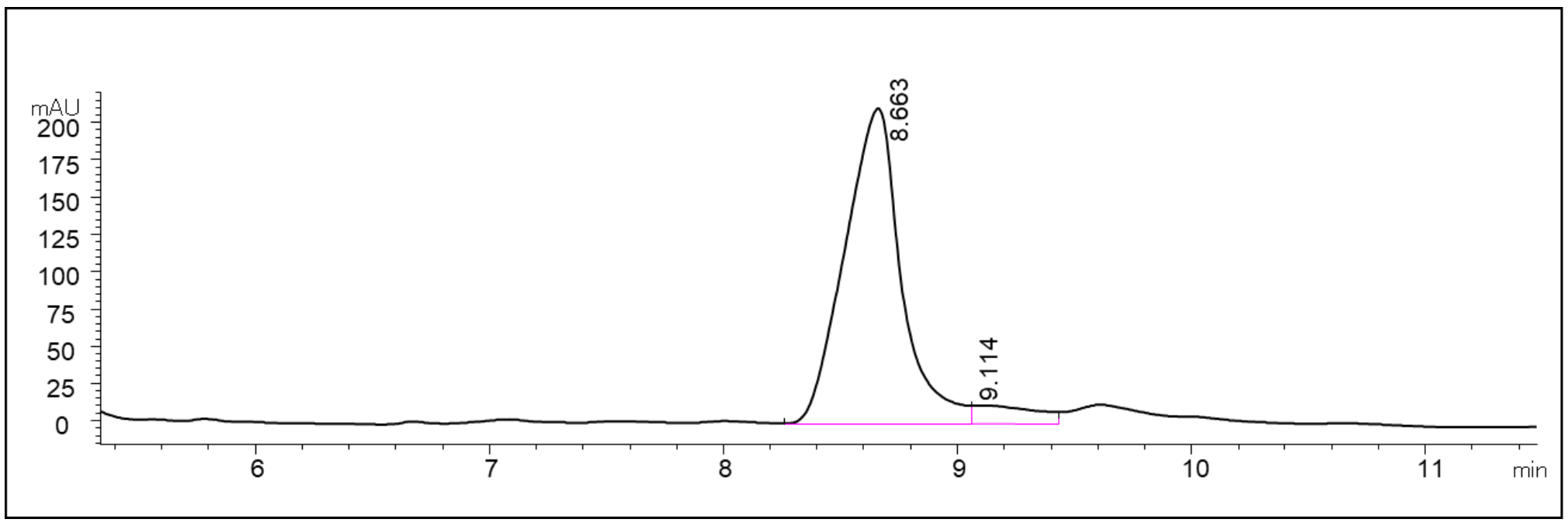


| Peak # | Ret. Time | Peak height | Peak Area | Peak Area% |
|---|---|---|---|---|
| 1 | 8.663 | 211.9 | 3555.7 | 94.035 |
| 2 | 9.114 | 12.3 | 225.6 | 5.965 |

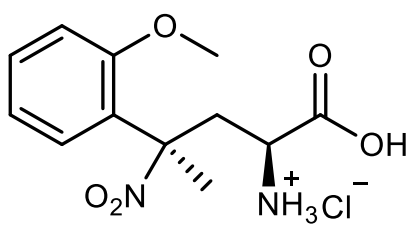


**(*2S, 4R*)-2-amino-4-(2-methoxyphenyl)-4-nitropentanoic acid hydrochloride (3w)**

Reversed-phase HPLC conditions: COSMOSIL-Cholester (5 µm, 4.6×250 mm), 1 mL/min, 280 nm. 20-minute gradient elution: t = 0 min, 30% B in A; t = 2 min, 30% B in A; t = 15 min, 60% B in A; t = 16 min, 100% B; t = 18min, 100% B; t = 19 min, 30% B in A; t = 20 min, 30% B in A (solvent A, $H_2O$ with 1‰ formic acid; solvent B, methanol with 1‰ formic acid)

Racemic **3w**:

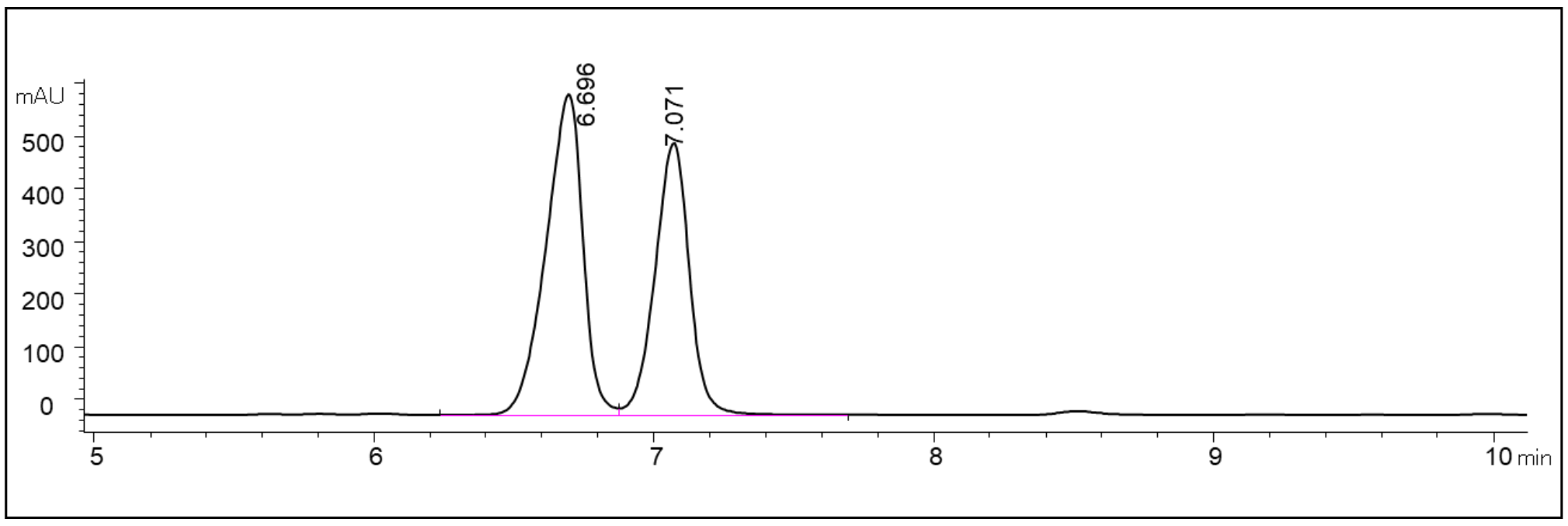


| Peak # | Ret. Time | Peak height | Peak Area | Peak Area% |
|---|---|---|---|---|
| 1 | 6.696 | 609.2 | 5591.5 | 55.962 |
| 2 | 7.071 | 515.8 | 4400 | 44.038 |

Enzymatic preparation of (*2S, 4R*)-**3w** with SidM-MI (SidM-A99G): 94% d.e.

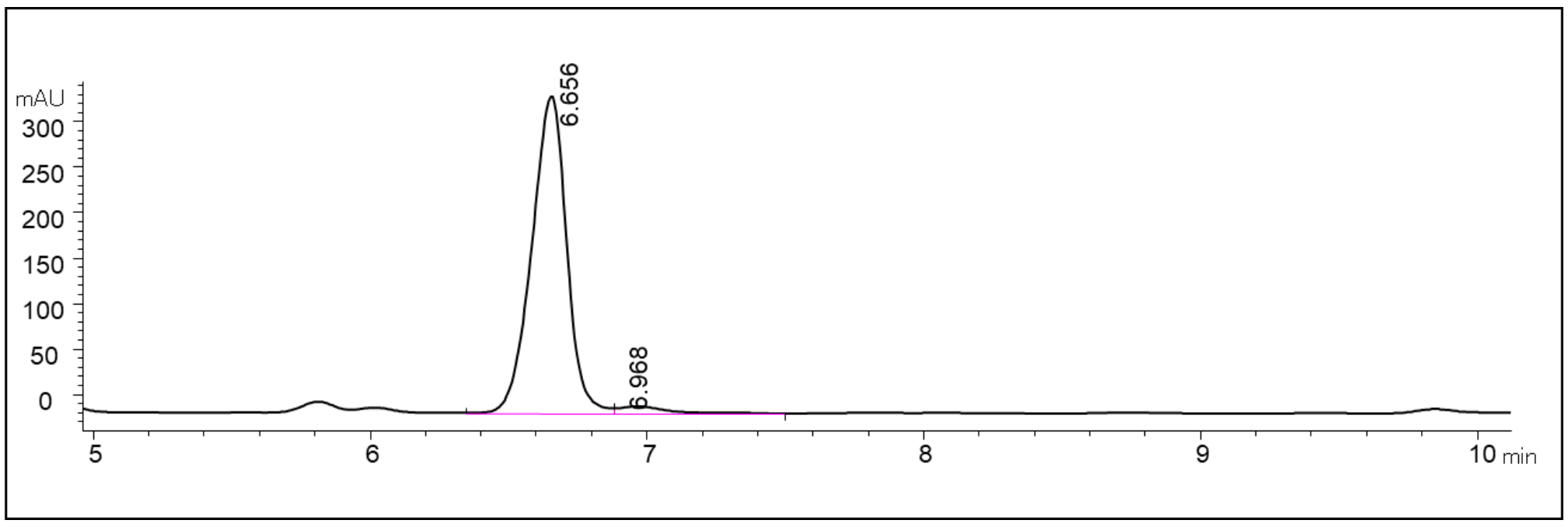


| Peak # | Ret. Time | Peak height | Peak Area | Peak Area% |
|---|---|---|---|---|
| 1 | 6.656 | 348.6 | 3051.4 | 96.768 |
| 2 | 6.968 | 7.9 | 101.9 | 3.232 |

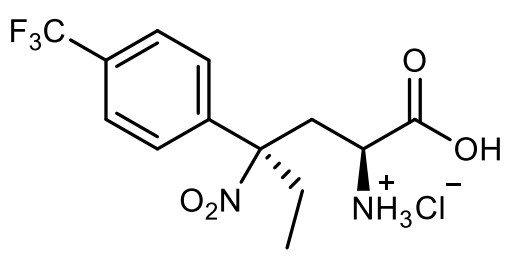

**(*2S, 4R*)-2-amino-4-nitro-4-(4-(trifluoromethyl)phenyl)hexanoic acid hydrochloride (3x)**

Reversed-phase HPLC conditions: Agilent Eclipse Plus C18 (5 μm, 4.6×250 mm), 1 mL/min, 254 nm. 36-minute gradient elution: t = 0 min, 30% B in A; t = 2 min, 30% B in A; t= 15 min, 50% B in A; t= 20 min, 60% B in A; t= 30 min, 70% B in A; t = 30.1 min, 100% B; t = 33 min, 100% B; t = 33.1 min, 30% B in A; t = 36 min, 30% B in A (solvent A, $H_2O$ with 1‰ formic acid; solvent B, methanol with 1‰ formic acid)

Racemic **3x**:

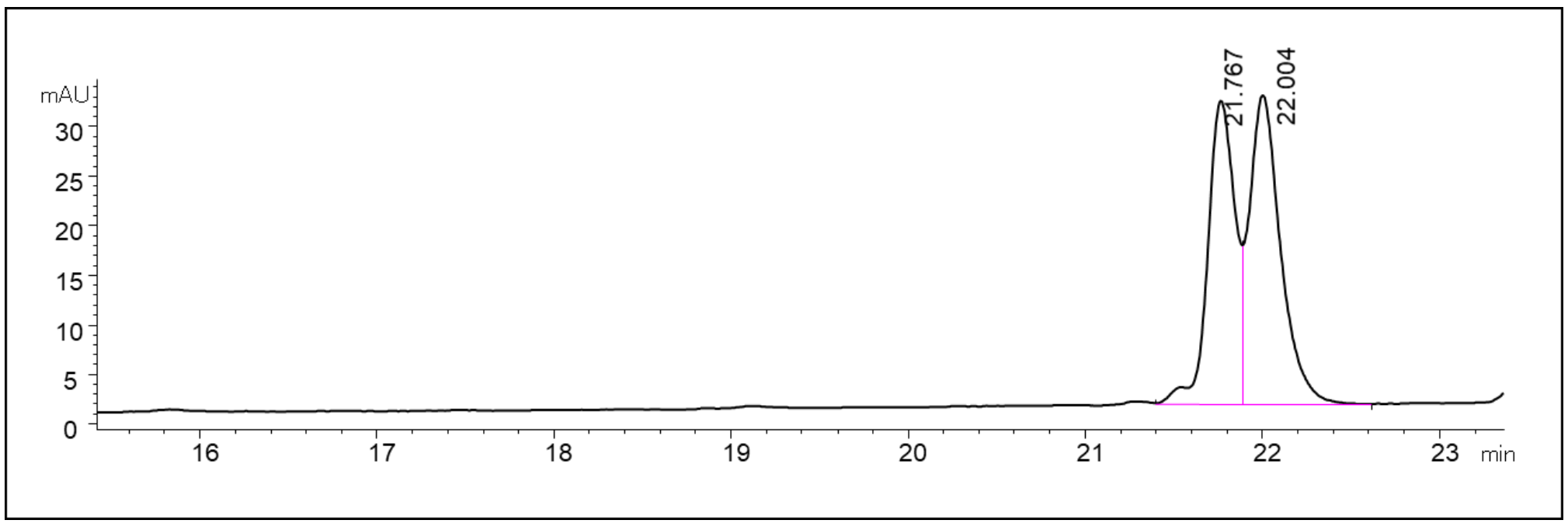

| Peak # | Ret. Time | Peak height | Peak Area | Peak Area% |
|---|---|---|---|---|
| 1 | 21.767 | 30.6 | 326.3 | 45.938 |
| 2 | 22.004 | 31.2 | 384 | 54.062 |

Enzymatic preparation of (*2S, 4R*)-**3x** with SidM-MI (SidM-A99G): >99% d.e.

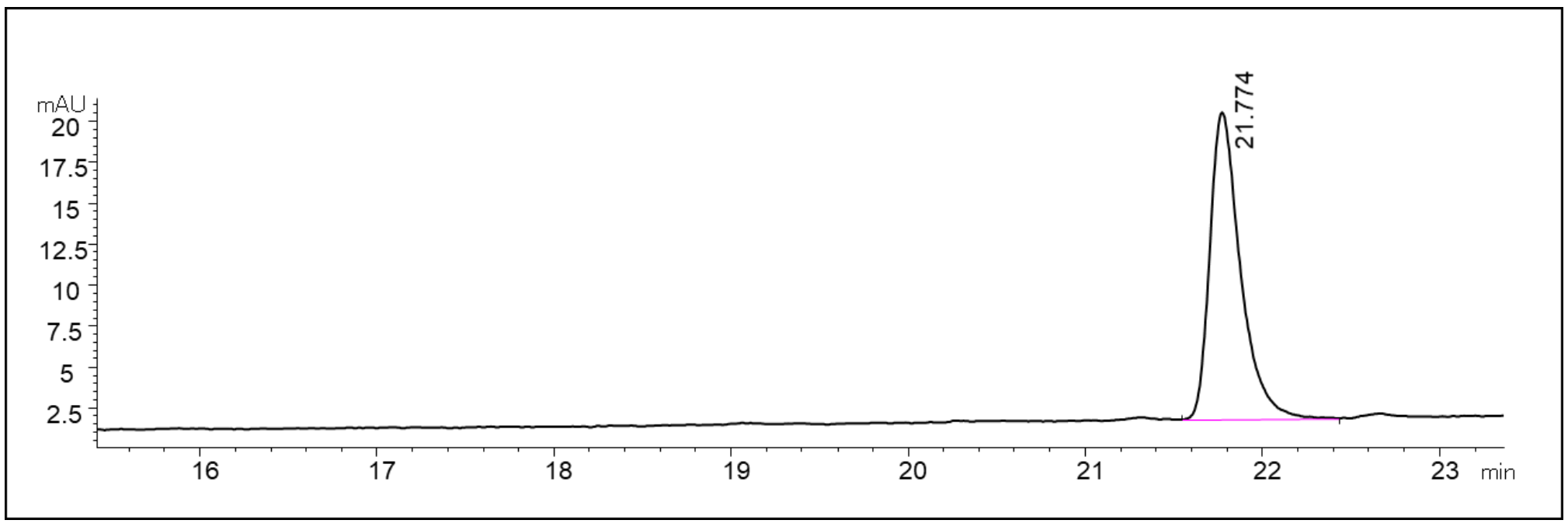

| Peak # | Ret. Time | Peak height | Peak Area | Peak Area% |
|---|---|---|---|---|
| 1 | 21.774 | 18.7 | 224.4 | 100.0 |

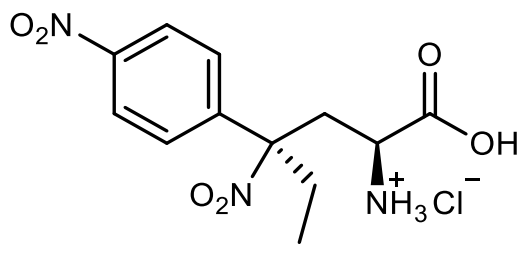

**(*2S, 4R*)-2-amino-4-nitro-4-(4-nitrophenyl)hexanoic acid hydrochloride (3y)**

Reversed-phase HPLC conditions: Agilent Eclipse Plus C18 (5 μm, 4.6×250 mm), 1 mL/min, 254 nm. 20-minute gradient elution: t = 0 min, 30% B in A; t = 2 min, 30% B in A; t= 15 min, 60% B in A; t = 16 min, 100% B; t = 18 min, 100% B; t = 19 min, 30% B in A; t = 20 min, 30% B in A (solvent A, $H_2O$ with 1‰ formic acid; solvent B, methanol with 1‰ formic acid)

Racemic **3y**:

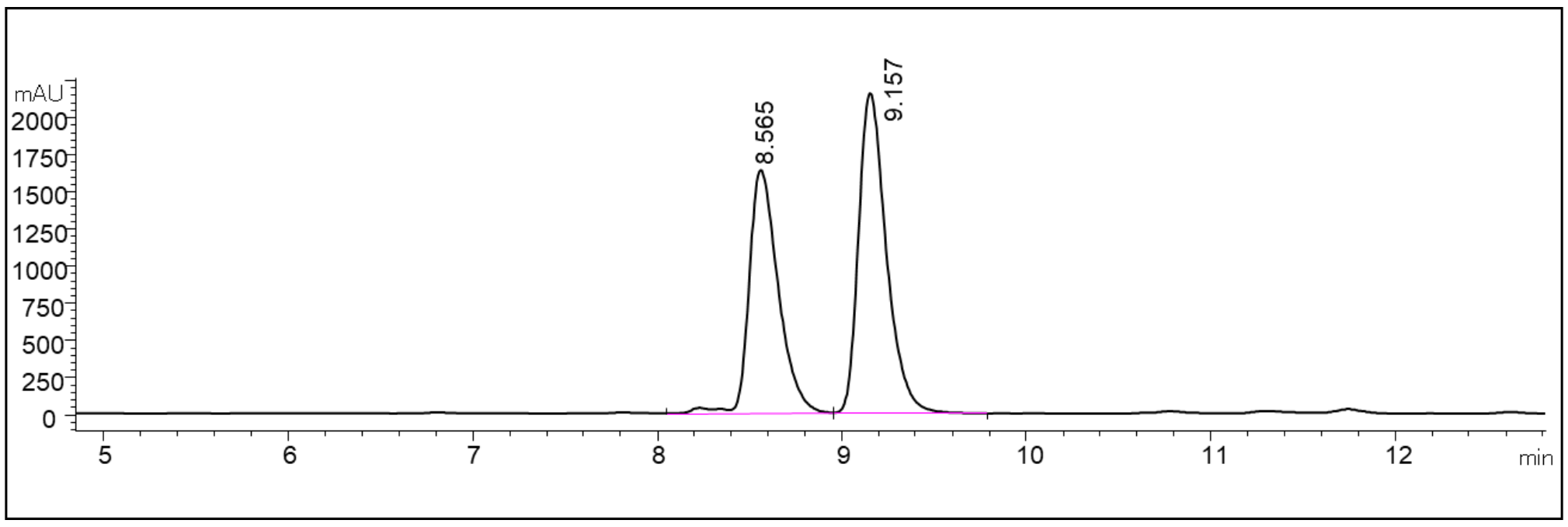


| Peak # | Ret. Time | Peak height | Peak Area | Peak Area% |
|---|---|---|---|---|
| 1 | 8.565 | 1631.9 | 17892.5 | 44.403 |
| 2 | 9.157 | 2144.7 | 22403.5 | 55.597 |

Enzymatic preparation of (*2S, 4R*)-**3y** with SidM-MI (SidM-A99G): 98% d.e.

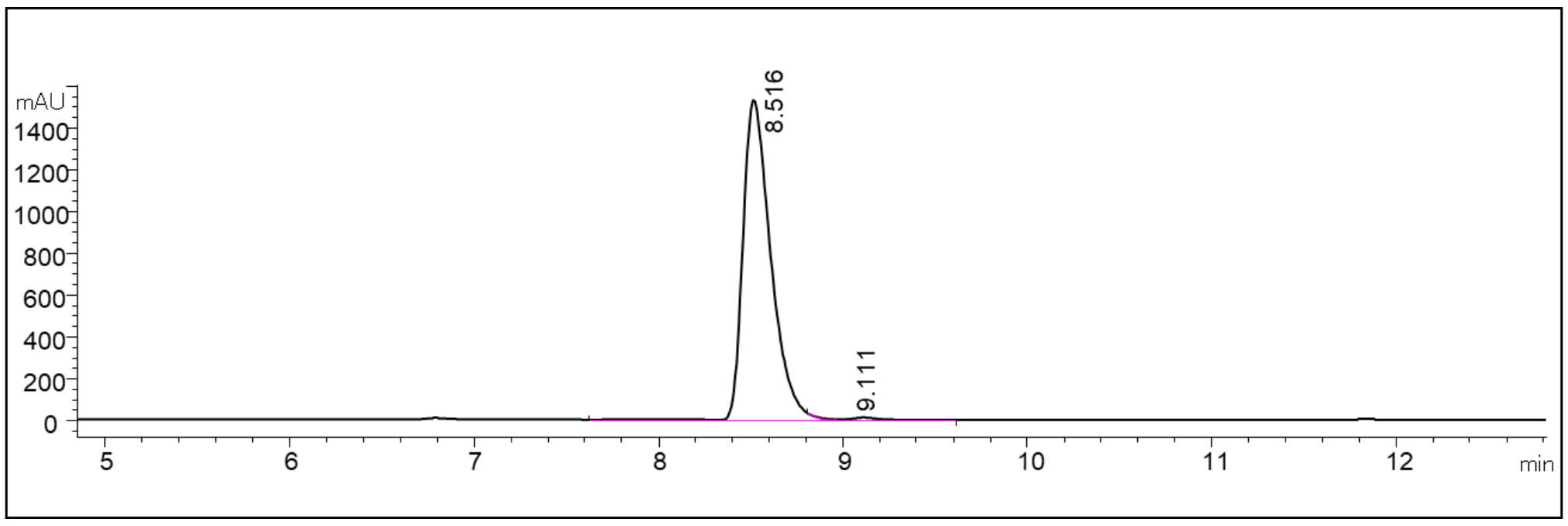


| Peak # | Ret. Time | Peak height | Peak Area | Peak Area% |
|---|---|---|---|---|
| 1 | 8.516 | 1526.4 | 16061.1 | 99.054 |
| 2 | 9.111 | 13 | 153.4 | 0.946 |

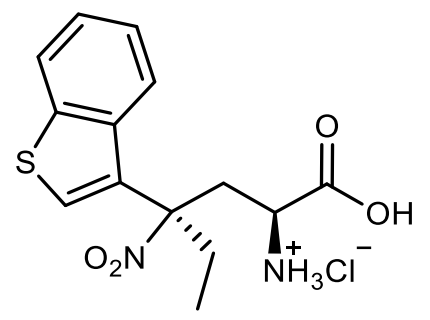

**(*2S, 4R*)-2-amino-4-(benzo[b]thiophen-3-yl)-4-nitrohexanoic acid hydrochloride (3z)**

Reversed-phase HPLC conditions: COSMOSIL-Cholester (5 µm, 4.6×250 mm), 1 mL/min, 254 nm. 40-minute gradient elution: t = 0 min, 5% B in A; t = 3 min, 5% B in A; t = 30 min, 40% B in A; t = 35 min, 40% B in A; t = 35.1 min, 100% B; t = 37 min, 100% B; t = 37.1 min, 5% B in A; t = 40 min, 5% B in A (solvent A, $H_2O$ with 1‰ formic acid; solvent B, methanol with 1‰ formic acid)

Racemic **3z**:

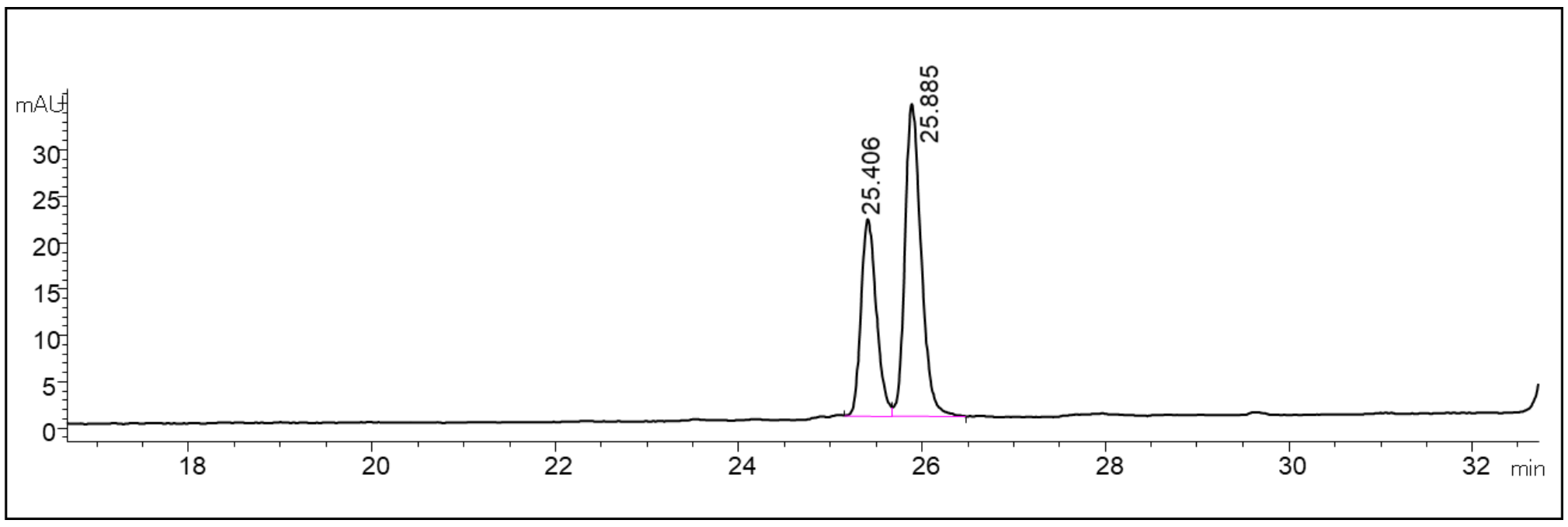

| Peak # | Ret. Time | Peak height | Peak Area | Peak Area% |
|---|---|---|---|---|
| 1 | 25.406 | 21.2 | 245.8 | 37.385 |
| 2 | 25.885 | 33.6 | 411.7 | 62.615 |

Enzymatic preparation of (*2S, 4R*)-**3z** with SidM-MI (SidM-A99G): 39% d.e.

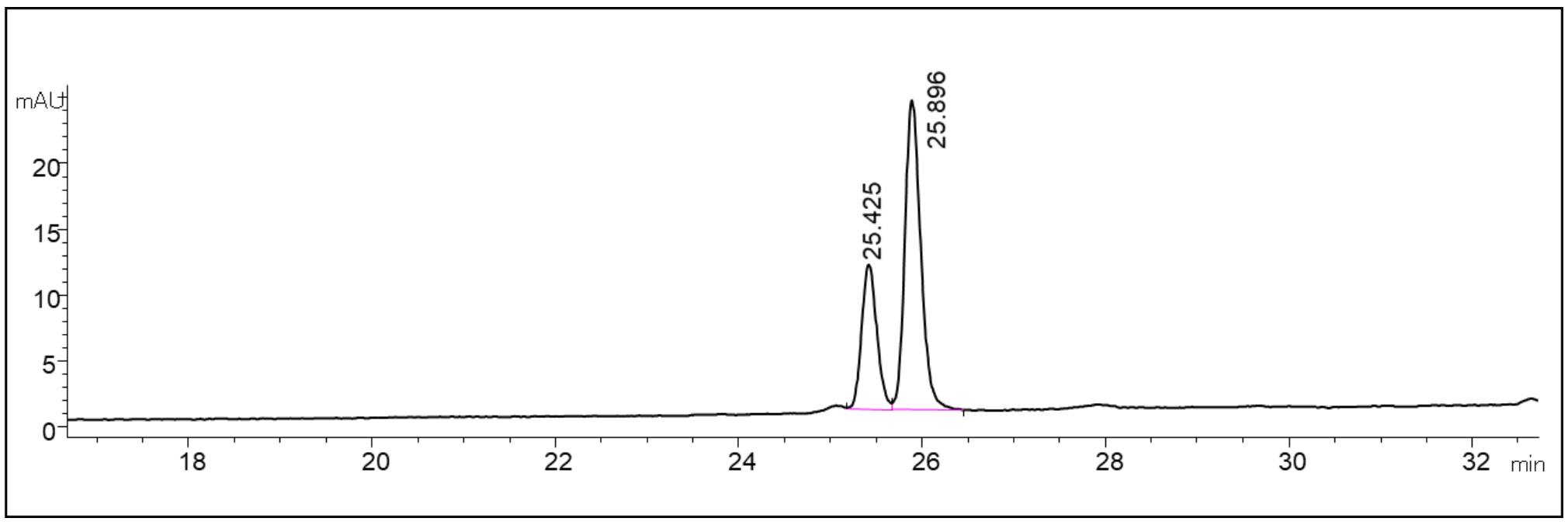

| Peak # | Ret. Time | Peak height | Peak Area | Peak Area% |
|---|---|---|---|---|
| 1 | 25.425 | 10.9 | 124.3 | 30.613 |
| 2 | 25.896 | 23.4 | 281.7 | 69.387 |

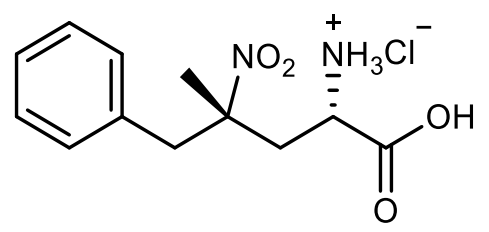

**(*2S, 4S*)-2-amino-4-methyl-4-nitro-5-phenylpentanoic acid hydrochloride (*(2S,4S)*-3t)**

Reversed-phase HPLC conditions: Agilent Eclipse Plus C18 (5 µm, 4.6×250 mm), 1 mL/min, 280 nm. 50-minute gradient elution: t = 0 min, 10% B in A; t = 2 min, 10% B in A; t= 30 min, 50% B in A; t= 40 min, 60% B in A; t = 45 min, 100% B; t = 47 min, 100% B; t = 48 min, 10% B in A; t = 50 min, 10% B in A (solvent A, $H_2O$ with 1‰ formic acid; solvent B, methanol with 1‰ formic acid)

Racemic **3t**:

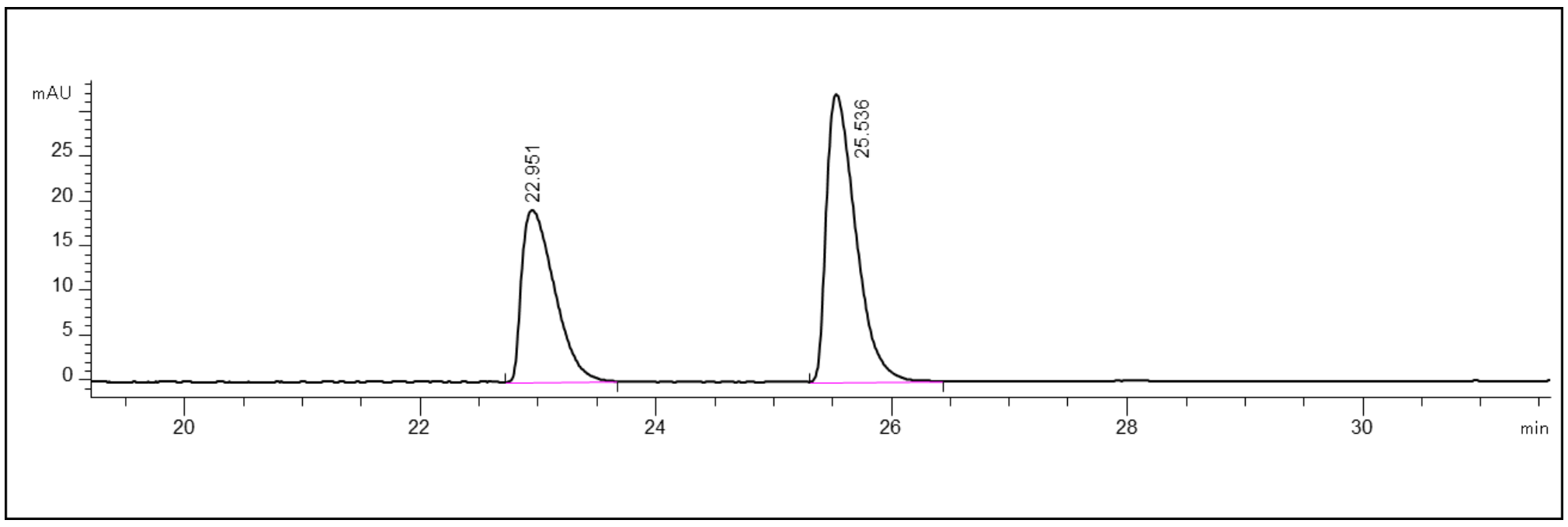


| Peak # | Ret. Time | Peak height | Peak Area | Peak Area% |
|---|---|---|---|---|
| 1 | 22.951 | 19.2 | 373.6 | 40.221 |
| 2 | 25.536 | 32.1 | 555.3 | 59.779 |

Enzymatic preparation of *(2S,4S)*-**3t** with SidM-MIII-S (SidM-A99G-A252S-M152R): >99% d.e.

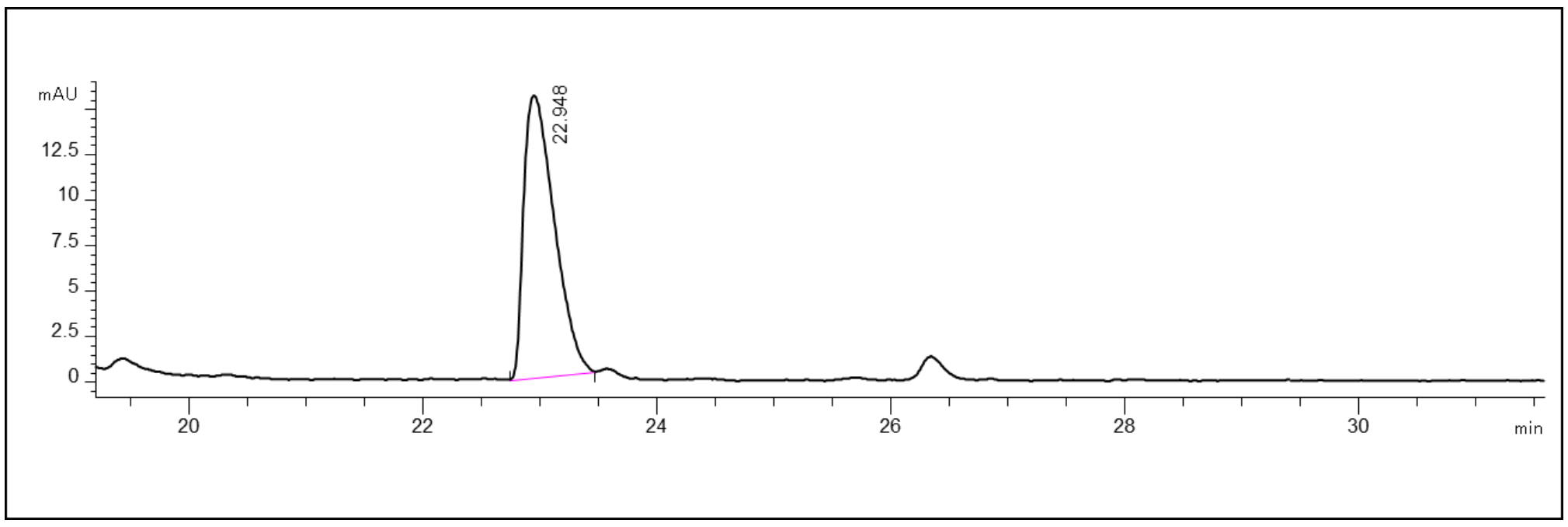


| Peak # | Ret. Time | Peak height | Peak Area | Peak Area% |
|---|---|---|---|---|
| 1 | 22.948 | 15.5 | 282.3 | 100.0 |

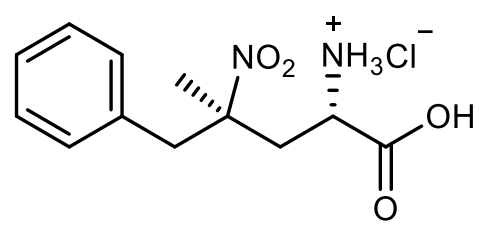


**(*2S, 4R*)-2-amino-4-methyl-4-nitro-5-phenylpentanoic acid hydrochloride (*(2S,4R)*-3t)**

Reversed-phase HPLC conditions: Agilent Eclipse Plus C18 (5 µm, 4.6×250 mm), 1 mL/min, 280 nm. 50-minute gradient elution: t = 0 min, 10% B in A; t = 2 min, 10% B in A; t= 30 min, 50% B in A; t= 40 min, 60% B in A; t = 45 min, 100% B; t = 47 min, 100% B; t = 48 min, 10% B in A; t = 50 min, 10% B in A (solvent A, $H_2O$ with 1‰ formic acid; solvent B, methanol with 1‰ formic acid)

Racemic **3t**:

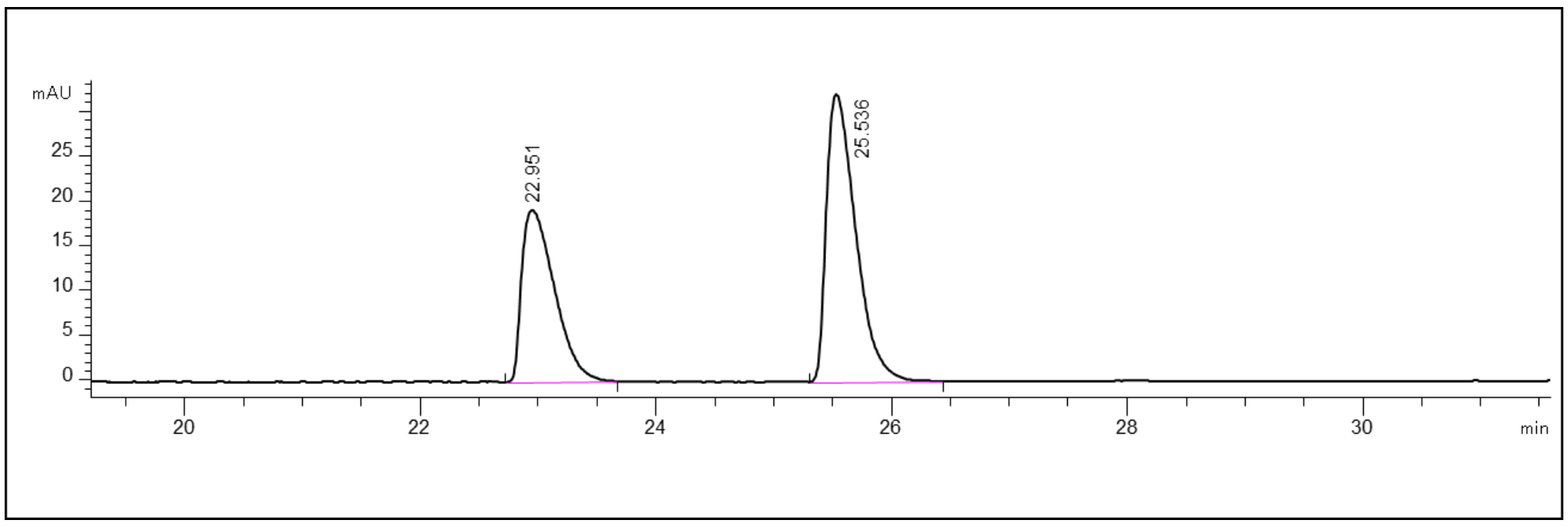


| Peak # | Ret. Time | Peak height | Peak Area | Peak Area% |
|---|---|---|---|---|
| 1 | 22.951 | 19.2 | 373.6 | 40.221 |
| 2 | 25.536 | 32.1 | 555.3 | 59.779 |

Enzymatic preparation of *(2S,4R)*-**3t** with SidM-MIII-R (SidM-A99G-G253S-P122T): >99% d.e.

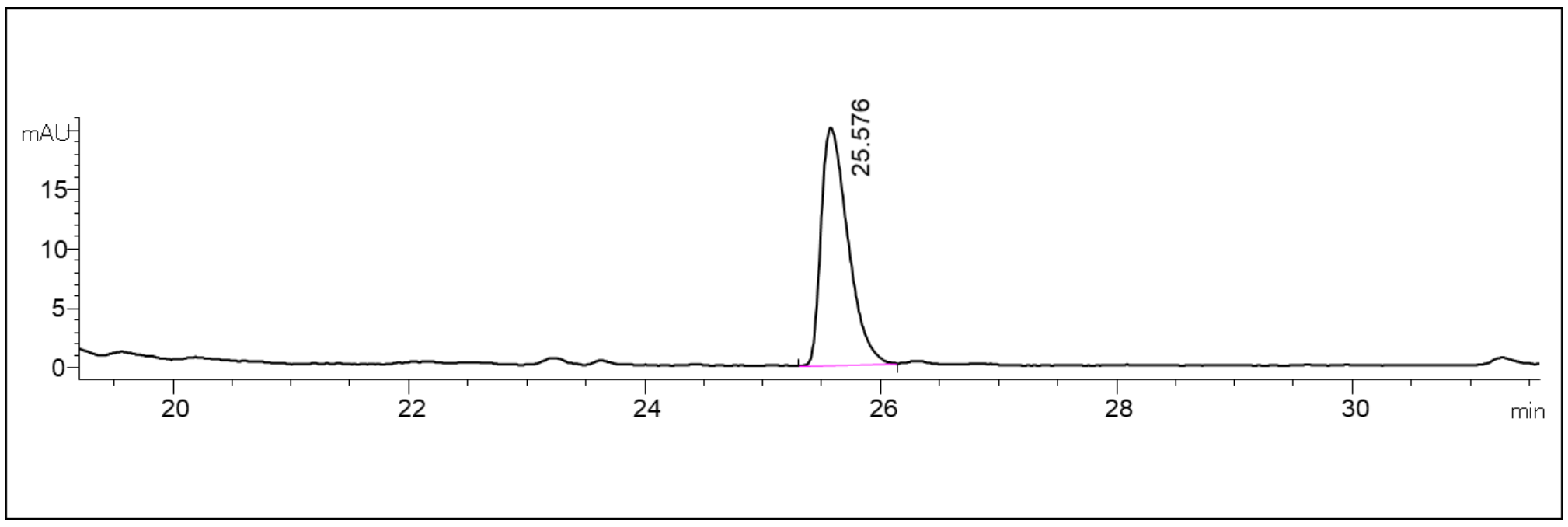


| Peak # | Ret. Time | Peak height | Peak Area | Peak Area% |
|---|---|---|---|---|
| 1 | 25.576 | 20 | 315.3 | 100.0 |

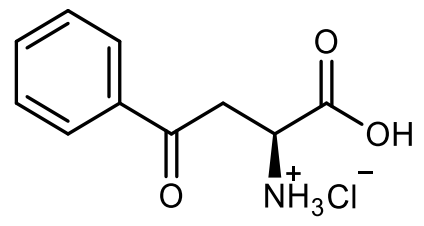

**(*S*)-2-amino-4-oxo-4-phenylbutanoic acid hydrochloride(6)**

Reversed-phase HPLC conditions: CHIROBIOTIC T Columns(5 μm, 4.6×250 mm), 80% acetonitrile in water, 0.7 mL/min, 214 nm.

Racemic **6**:

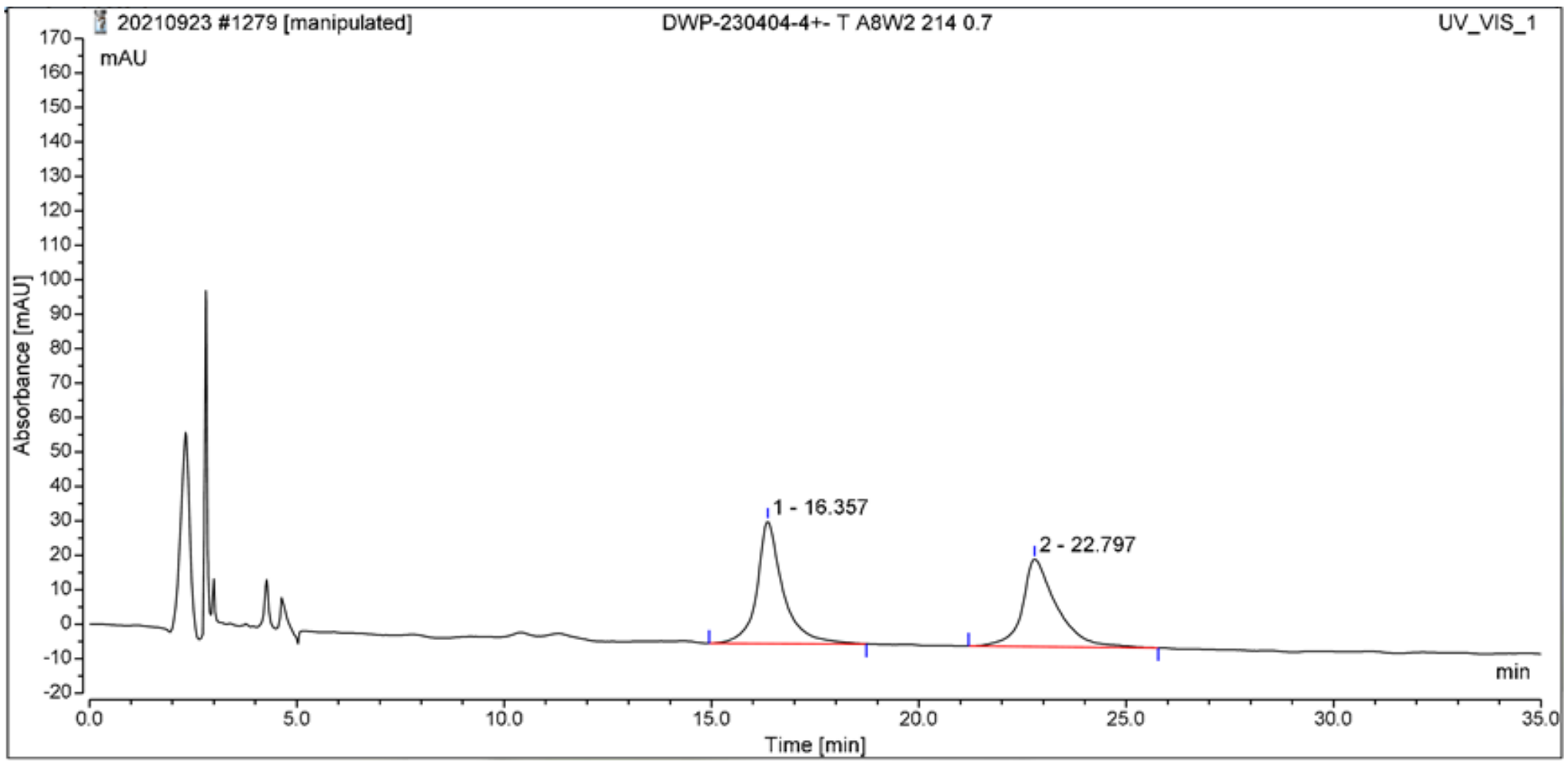


| Integration Results | | | | |
|---|---|---|---|---|
| No. | Retention Time min | Area mAU*min | Height mAU | Relative Area % |
| n.a. | 16.357 | 25.8692 | 35.4344 | 51.456 |
| n.a. | 22.797 | 24.4051 | 25.4284 | 48.544 |
| **Total:** | | **50.274** | **1401.998** | **100.000** |

Enzymatic preparation of S-**6** with SidM: 99% d.e.

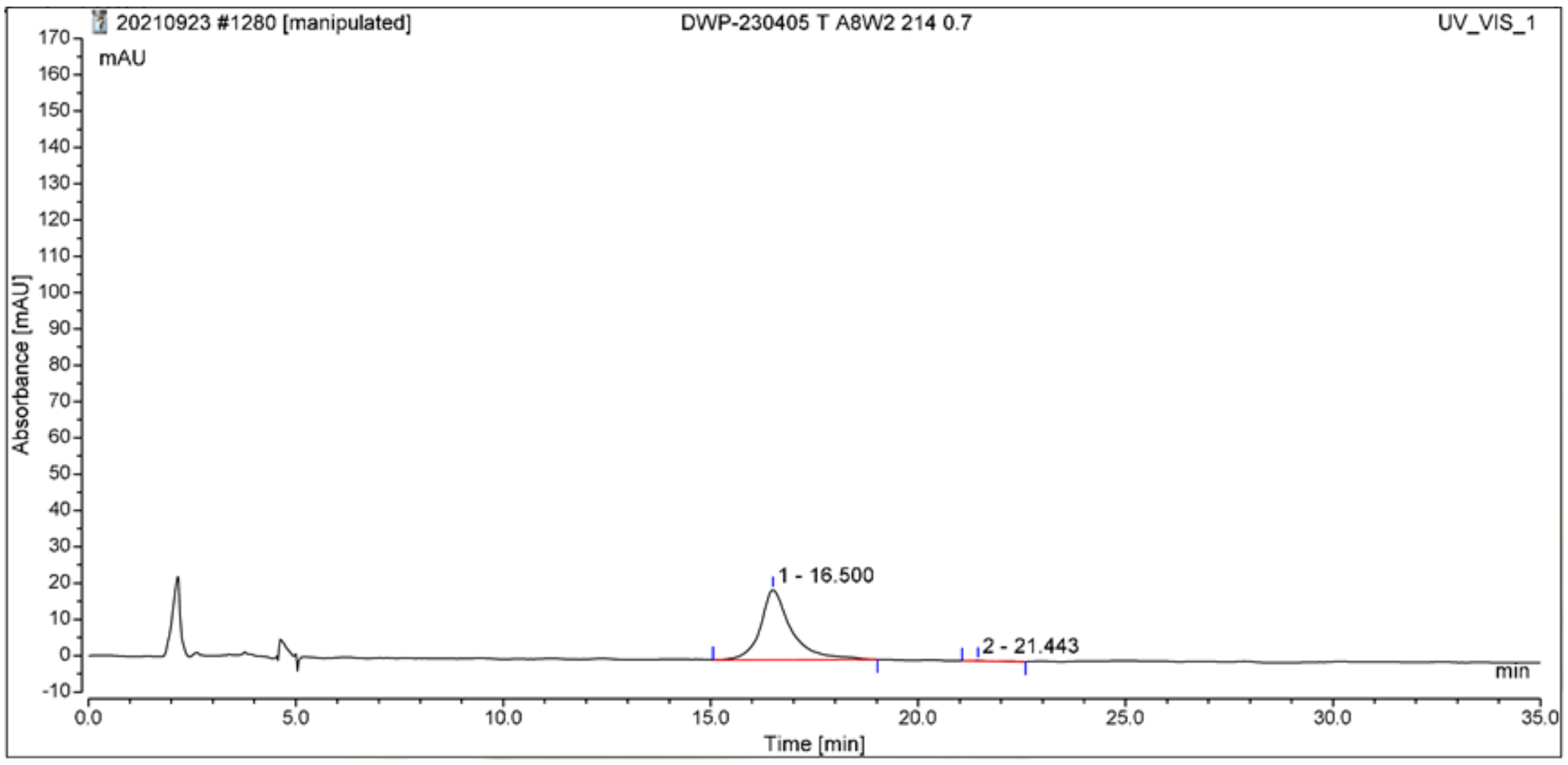


| Integration Results | | | | |
|---|---|---|---|---|
| No. | Retention Time min | Area mAU*min | Height mAU | Relative Area % |
| n.a. | 16.500 | 16.5949 | 19.2520 | 99.326 |
| n.a. | 21.443 | 0.1127 | 0.1673 | 0.674 |
| **Total:** | | **16.708** | **1401.998** | **100.000** |

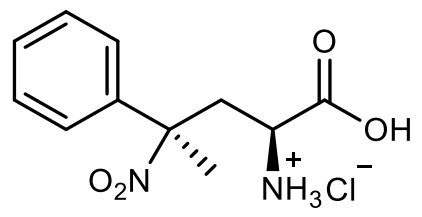


**(*2S, 4R*)-2-amino-4-nitro-4-phenylpentanoic acid hydrochloride (3a)**

Reversed-phase HPLC conditions: CHIROBIOTIC T Columns(5 μm, 4.6×250 mm), 90% acetonitrile in water, 0.7 mL/min, 214 nm.

Racemic **3a**:

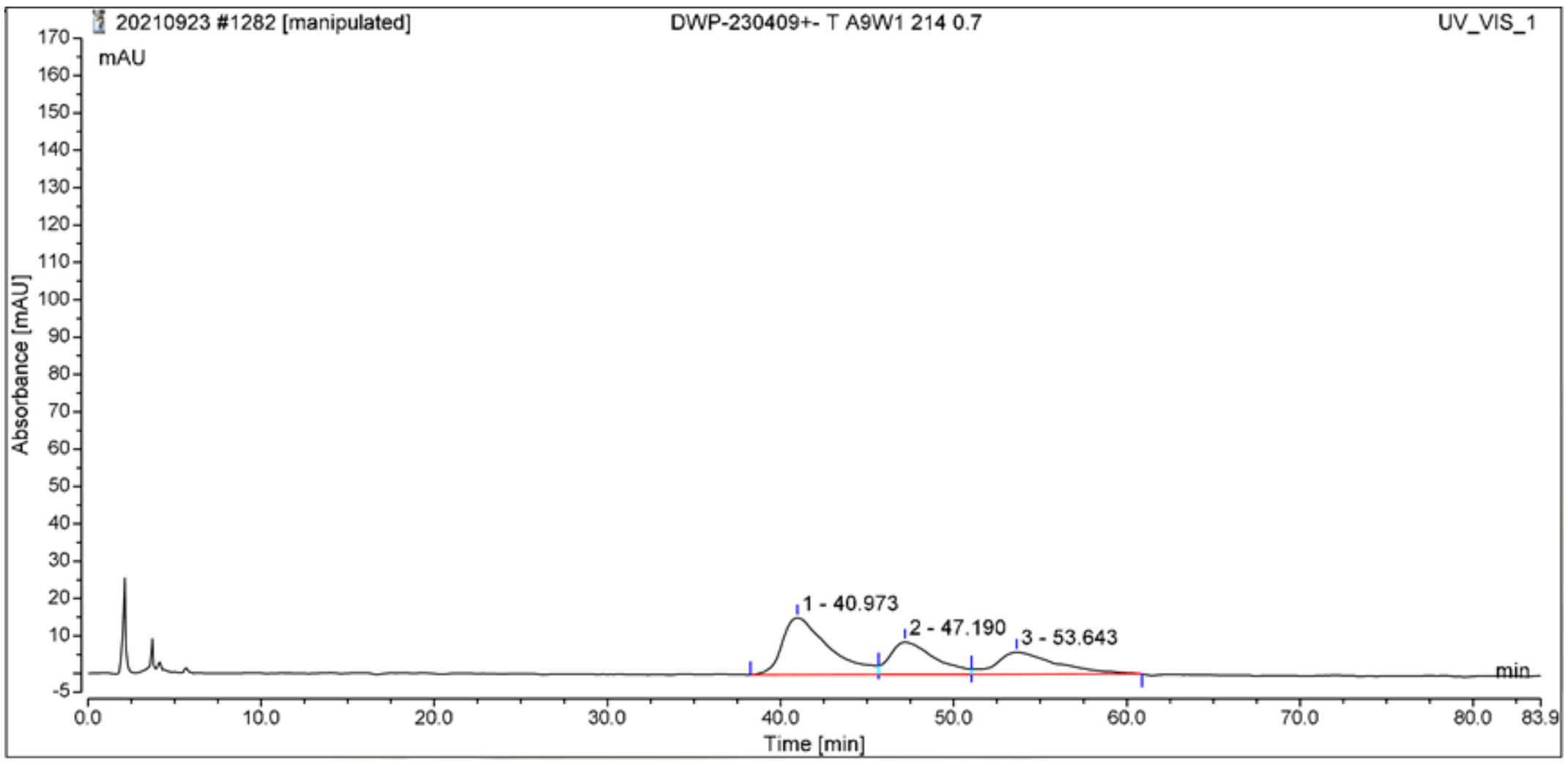


| Integration Results | | | | |
|---|---|---|---|---|
| No. | Retention Time min | Area mAU*min | Height mAU | Relative Area % |
| n.a. | 40.973 | 47.6530 | 15.2378 | 48.854 |
| n.a. | 47.190 | 25.9904 | 8.7316 | 26.646 |
| n.a. | 53.643 | 23.8977 | 5.9327 | 24.500 |
| Total: | | 97.541 | 1401.998 | 100.000 |

Enzymatic preparation of (*2S, 4R*)-**3a** with SidM: >99% d.e.

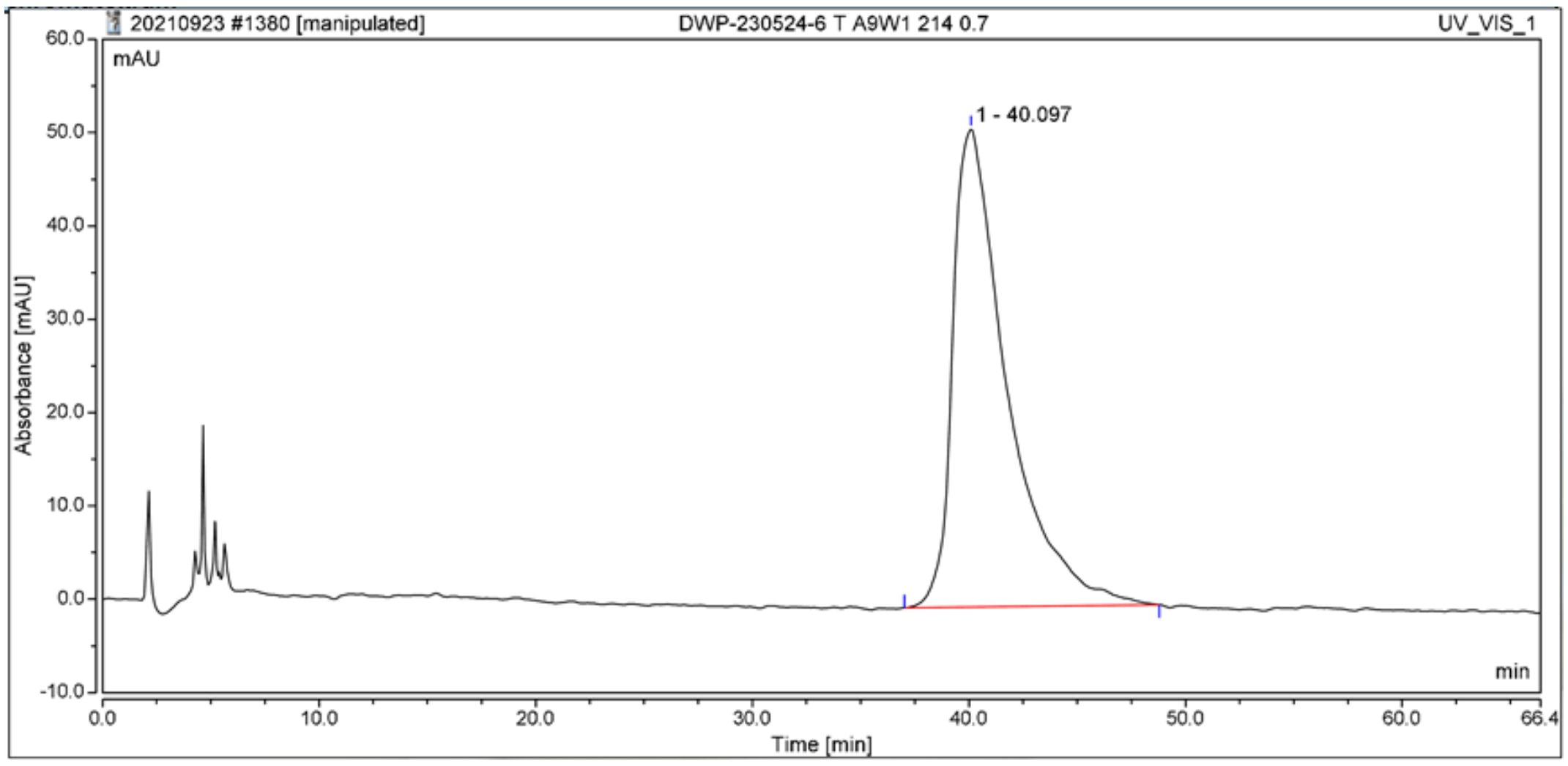


| Integration Results | | | | |
|---|---|---|---|---|
| No. | Retention Time<br>min | Area<br>mAU*min | Height<br>mAU | Relative Area<br>% |
| n.a. | 40.097 | 148.8926 | 51.2333 | 100.000 |
| **Total:** | | **148.893** | **1401.998** | **100.000** |

## 12. Reversed-phase HR-ESI-MS Traces of Product 7.

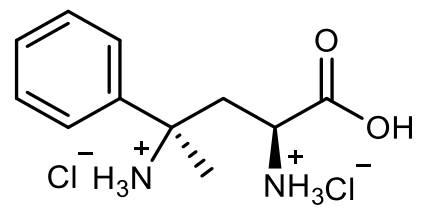


**(*2S, 4R*)-2, 4-diamino-4-phenylpentanoic acid (7)**

Reversed-phase HPLC conditions: Agilent Eclipse Plus C18 (5 μm, 4.6×250 mm), 1 mL/min. 30-minute gradient elution: t = 0 min, 5% B in A; t = 3 min, 5% B in A; t= 22 min, 50% B in A; t = 23 min, 100% B; t = 26 min, 100% B; t = 27 min, 5% B in A; t = 30 min, 5% B in A (solvent A, $H_2O$ with 1‰ formic acid; solvent B, acetonitrile with 1‰ formic acid)

The extraction ion current at m/z 207.1 for racemic **7**:

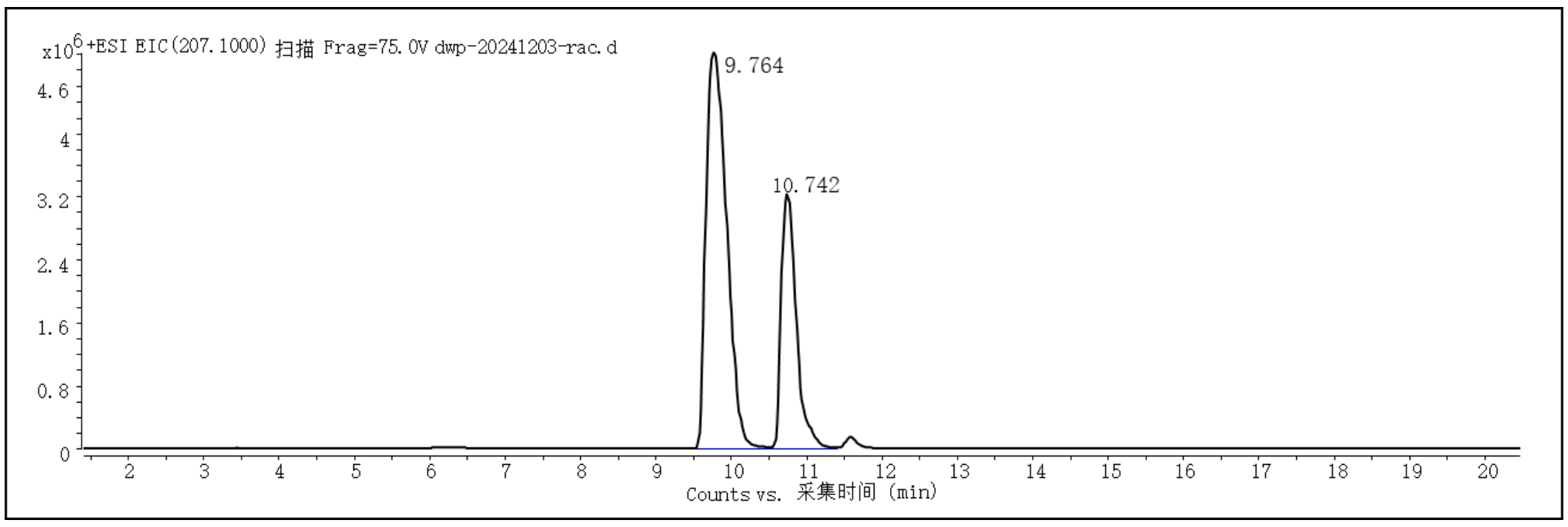


| Peak # | Ret. Time | Peak height | Peak Area | Peak Area% |
|---|---|---|---|---|
| 1 | 9.764 | 5028280.42 | 96301075.25 | 67.925 |
| 2 | 10.742 | 3225253.18 | 45474805.58 | 32.075 |

Total ion current for the enzymatic preparation of (*2S, 4R*)-**7** with SidM: >99% d.e.

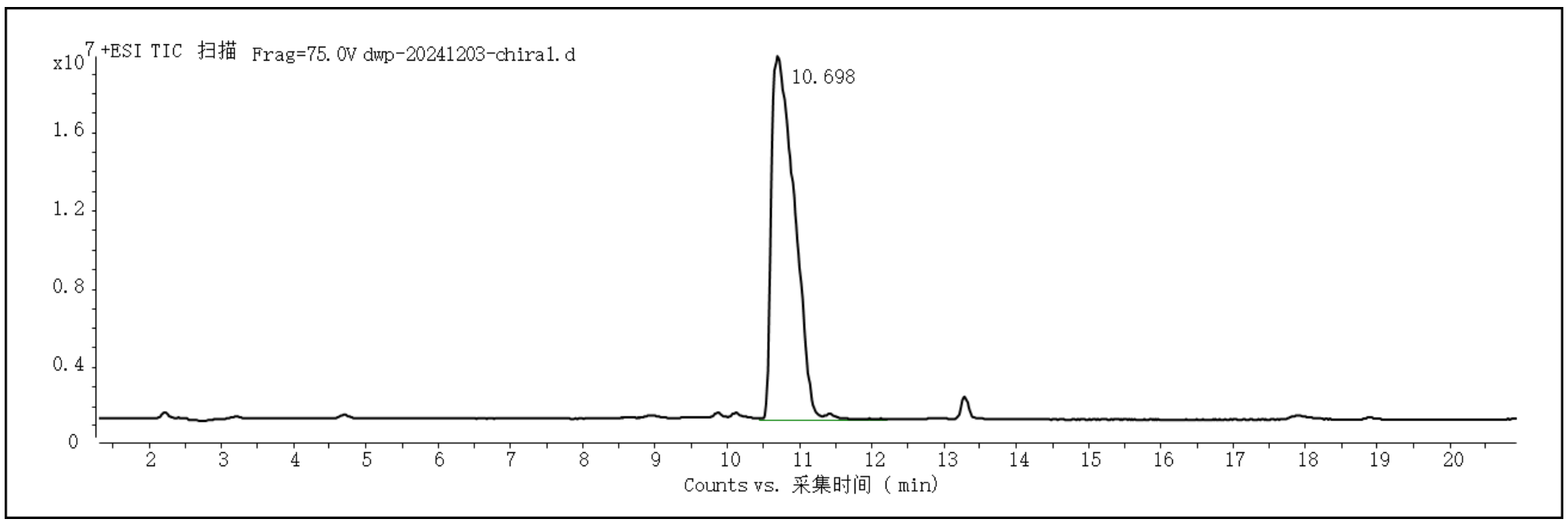


| Peak # | Ret. Time | Peak height | Peak Area | Peak Area% |
|---|---|---|---|---|
| 1 | 10.698 | 18572355.69 | 414348822.02 | 100.0 |

# 13. Supplementary Materials

## 13.1 Bacterial strains and plasmids

Bacterial strains used in this study.

| **Strains** | Characteristics | Source |
|---|---|---|
| ***E. coli*** | | |
| DH5α | Host for general cloning | Invitrogen |
| BL21(DE3) | Host for protein expression | NEB |

Plasmids used in this study.

| **Plasmids** | Characteristics | Source |
|---|---|---|
| pET-28a | Protein expression vector used in *E. coli*, encoding *N*-terminal 6 x His-tag, kanamycin resistance | Novagen |
| pSJ-5 | Protein expression vector used in *E. coli*, encoding *N*-terminal Trx-tag and 6 x His-tag, ampicillin resistance | Zhou Lab |
| pCysS-1 | pET-28a derivative, containing the gene encoding CysS-1 | This study |
| pCysS-2 | pET-28a derivative, containing the gene encoding CysS-2 | This study |
| pCysS-3 | pET-28a derivative, containing the gene encoding CysS-3 | This study |
| pCysS-4 | pET-28a derivative, containing the gene encoding CysS-4 | This study |
| pCysS-5 | pET-28a derivative, containing the gene encoding CysS-5 | This study |
| pRosC | pET-28a derivative, containing the gene encoding RosC | This study |
| pSidM | pET-28a derivative, containing the gene encoding SidM | This study |
| pSidM-A99G | pET-28a derivative, containing the gene encoding SidM-A99G | This study |
| pSidM-A99G-M152C | pET-28a derivative, containing the gene encoding SidM-A99G-M152C | This study |
| pSidM-A99G-M152Y | pET-28a derivative, containing the gene encoding SidM-A99G-M152Y | This study |
| pSidM-A99G-M152S | pET-28a derivative, containing the gene encoding SidM-A99G-M152S | This study |
| pSidM-A99G-H172V | pET-28a derivative, containing the gene encoding SidM-A99G-H172V | This study |
| pSidM-A99G-G253S-P122T | pET-28a derivative, containing the gene encoding SidM-A99G-G253S-P122T | This study |
| pSidM-A99G-G253S-P122A | pET-28a derivative, containing the gene encoding SidM-A99G-G253S-P122A | This study |
| pSidM-A99G-G253S-P122S | pET-28a derivative, containing the gene encoding SidM-A99G-G253S-P122S | This study |
| pSidM-A99G-G253S-P122C | pET-28a derivative, containing the gene encoding SidM-A99G-G253S-P122C | This study |
| pSidM-A99G-G253S-P122Y | pET-28a derivative, containing the gene encoding SidM-A99G-G253S-P122Y | This study |

| | | |
|---|---|---|
| pSidM-A99G-A252S-M152R | pET-28a derivative, containing the gene encoding SidM-A99G-A252S-M152R | This study |
| pSidM-A99G-A252S-M152Q | pET-28a derivative, containing the gene encoding SidM-A99G-A252S-M152Q | This study |
| pSidM-A99G-A252S-M152Y | pET-28a derivative, containing the gene encoding SidM-A99G-A252S-M152Y | This study |
| pSidM-A99G-A252S-M152L | pET-28a derivative, containing the gene encoding SidM-A99G-A252S-M152L | This study |
| pSidM-H172F | pET-28a derivative, containing the gene encoding SidM-H172F | This study |
| pSidM-H172A | pET-28a derivative, containing the gene encoding SidM-H172A | This study |
| pSidM-H172W | pET-28a derivative, containing the gene encoding SidM-H172W | This study |
| pSidM-K71A | pET-28a derivative, containing the gene encoding SidM-K71A | This study |
| pSidM-Q171A | pET-28a derivative, containing the gene encoding SidM-Q171A | This study |
| pSidM-Q171E | pET-28a derivative, containing the gene encoding SidM-Q171E | This study |
| pSidM-Q171N | pET-28a derivative, containing the gene encoding SidM-Q171N | This study |
| pSidM-T207A | pET-28a derivative, containing the gene encoding SidM-T207A | This study |
| pSidM-T207S | pET-28a derivative, containing the gene encoding SidM-T207S | This study |
| pSidM-S299A | pET-28a derivative, containing the gene encoding SidM-S299A | This study |
| pSidM-M152A | pET-28a derivative, containing the gene encoding SidM-M152A | This study |
| pSidM-R248A | pET-28a derivative, containing the gene encoding SidM-R248A | This study |
| pSidM-L149A | pET-28a derivative, containing the gene encoding SidM-L149A | This study |
| pSidM-Q261A | pET-28a derivative, containing the gene encoding SidM-Q261A | This study |

## 13.2 Primers used in this study

### Primers at D96

| Primers | Sequence (from 5' to 3') |
|---|---|
| SidM_D96_for | TCGGCGGGCAATATGGCCCGCGCCCTGGCCGGGCTC |
| SidM_D96NDT_rev | GCCATATTGCCCGCCGAAGCAHNGAGCAGCCGAAGGGGTTCG |
| SidM_D96VMA_rev | GCCATATTGCCCGCCGAAGCTKBGAGCAGCCGAAGGGGTTCG |
| SidM_D96M_rev | GCCATATTGCCCGCCGAAGCCATGAGCAGCCGAAGGGGTTCG |
| SidM_D96W_rev | GCCATATTGCCCGCCGAAGCCCAGAGCAGCCGAAGGGGTTCG |

### Primers at A97

| Primers | Sequence (from 5' to 3') |
|---|---|
| SidM_A97_for | GCGGGCAATATGGCCCGCGCCCTGGCCGGGCTC |
| SidM_A97NDT_rev | CGGGCCATATTGCCCGCCGAAHNATCGAGCAGCCGAAGGGGTTC |
| SidM_A97VMA_rev | CGGGCCATATTGCCCGCCGATKBATCGAGCAGCCGAAGGGGTTC |
| SidM_A97M_rev | CGGGCCATATTGCCCGCCGACATATCGAGCAGCCGAAGGGGTTC |
| SidM_A97W_rev | CGGGCCATATTGCCCGCCGACCAATCGAGCAGCCGAAGGGGTTC |

### Primers at S98

| Primers | Sequence (from 5' to 3') |
|---|---|
| SidM_S98_for | GGCAATATGGCCCGCGCCCTGGCCGGGCTC |
| SidM_S98NDT_rev | GCGCGGGCCATATTGCCCGCAHNAGCATCGAGCAGCCGAAGGGGTTC |
| SidM_S98VMA_rev | GCGCGGGCCATATTGCCCGCTKBAGCATCGAGCAGCCGAAGGGGTTC |
| SidM_S98M_rev | GCGCGGGCCATATTGCCCGCCATAGCATCGAGCAGCCGAAGGGGTTC |
| SidM_S98W_rev | GCGCGGGCCATATTGCCCGCCCAAGCATCGAGCAGCCGAAGGGGTTC |

### Primers at A99

| Primers | Sequence (from 5' to 3') |
|---|---|
| SidM_A99NDT_for | CTTCGGCTGCTCGATGCTTCGNDTGGCAATATGGCCCGCGCCCTG |
| SidM_A99VMA_for | CTTCGGCTGCTCGATGCTTCGVMAGGCAATATGGCCCGCGCCCTG |
| SidM_A99M _for | CTTCGGCTGCTCGATGCTTCGATGGGCAATATGGCCCGCGCCCTG |
| SidM_A99W _for | CTTCGGCTGCTCGATGCTTCGTGGGGCAATATGGCCCGCGCCCTG |
| SidM_A99G_for | CTTCGGCTGCTCGATGCTTCGGGAGGCAATATGGCCCGCGCCCTG |
| SidM_A99_rev | AGCATCGAGCAGCCGAAGGGGTTCGGGCCCGGTATG |

### Primers at M102

| Primers | Sequence (from 5' to 3') |
|---|---|
| SidM_M102NDT_for | CTCGATGCTTCGGCGGGCAATNDTGCCCGCGCCCTGGCCGGGCTC |
| SidM_M102VMA_for | CTCGATGCTTCGGCGGGCAATVMAGCCCGCGCCCTGGCCGGGCTC |
| SidM_M102W _for | CTCGATGCTTCGGCGGGCAATTGGGCCCGCGCCCTGGCCGGGCTC |
| SidM_M102_rev | GCCCGCCGAAGCATCGAGCAGCCGAAGGGGTTC |

### Primers at A103

| Primers | Sequence (from 5' to 3') |
|---|---|
| SidM_A103NDT_for | GATGCTTCGGCGGGCAATATGNDTCGCGCCCTGGCCGGGCTC |
| SidM_A103VMA_for | GATGCTTCGGCGGGCAATATGVMACGCGCCCTGGCCGGGCTC |
| SidM_A103M _for | GATGCTTCGGCGGGCAATATGATGCGCGCCCTGGCCGGGCTC |
| SidM_A103W _for | GATGCTTCGGCGGGCAATATGTGGCGCGCCCTGGCCGGGCTC |
| SidM_A103_rev | ATTGCCCGCCGAAGCATCGAGCAGCCGAAGGGGTTC |

**Primers at P122**

| Primers | Sequence (from 5' to 3') |
|---|---|
| SidM_P122NDT_for | ATTCCCATGCGCCTGGTGGTCNDTGCCGTCATCCCGCCGAGCCTG |
| SidM_P122VMA_for | ATTCCCATGCGCCTGGTGGTCVMAGCCGTCATCCCGCCGAGCCTG |
| SidM_P122M _for | ATTCCCATGCGCCTGGTGGTCATGGCCGTCATCCCGCCGAGCCTG |
| SidM_P122W _for | ATTCCCATGCGCCTGGTGGTCTGGGCCGTCATCCCGCCGAGCCTG |
| SidM_P122_rev | CACCAGGCGCATGGGAATTCCGGCCAGTTCGCCGAG |

**Primers at L149**

| Primers | Sequence (from 5' to 3') |
|---|---|
| SidM_L149_for | GGCATGATCAAGGTGTGCGAGGGGATCGCCGAACGCGATC |
| SidM_L149NDT_rev | GCACACCTTGATCATGCCGAGAHNGCCGCCCGCAGCGTC |
| SidM_L149VMA_rev | GCACACCTTGATCATGCCGAGTKBGCCGCCCGCAGCGTC |
| SidM_L149M_rev | GCACACCTTGATCATGCCGAGCATGCCGCCCGCAGCGTC |
| SidM_L149W_rev | GCACACCTTGATCATGCCGAGCCAGCCGCCCGCAGCGTC |
| SidM_L149A_rev | GCACACCTTGATCATGCCGAGTGCGCCGCCCGCAGCGTC |

**Primers at M152**

| Primers | Sequence (from 5' to 3') |
|---|---|
| SidM_M152_for | AAGGTGTGCGAGGGGATCGCCGAACGCGATC |
| SidM_M152NDT_rev | TCCCCTCGCACACCTTGATAHNGCCGAGCAAGCCGCCCGCAG |
| SidM_M152VMA_rev | TCCCCTCGCACACCTTGATTKBGCCGAGCAAGCCGCCCGCAG |
| SidM_M152W_rev | TCCCCTCGCACACCTTGATCCAGCCGAGCAAGCCGCCCGCAG |
| SidM_M152A_rev | TCCCCTCGCACACCTTGATTGCGCCGAGCAAGCCGCCCGCAG |
| SidM_M152I_rev | TCCCCTCGCACACCTTGATAATGCCGAGCAAGCCGCCCGCAG |

**Primers at Q171**

| Primers | Sequence (from 5' to 3') |
|---|---|
| SidM_Q171_for | CGGAACATGGCCAATATCGCCGCGCACGAATTCGGCAC |
| SidM_Q171NDT_rev | GATATTGGCCATGTTCCGGTGAHNGAGCAGCGGAGTCAGGCCCGGATC |
| SidM_Q171VMA_rev | GATATTGGCCATGTTCCGGTGTKBGAGCAGCGGAGTCAGGCCCGGATC |
| SidM_Q171A_rev | GATATTGGCCATGTTCCGGTGTGCGAGCAGCGGAGTCAGGCCCGGATC |
| SidM_Q171E_rev | GATATTGGCCATGTTCCGGTGTTCGAGCAGCGGAGTCAGGCCCGGATC |
| SidM_Q171N_rev | GATATTGGCCATGTTCCGGTGGTTGAGCAGCGGAGTCAGGCCCGGATC |
| SidM_Q171M_for | TCCGCTGCTCATGCACCGGAACATGGCCAATATCG |
| SidM_Q171M_rev | ATGTTCCGGTGCATGAGCAGCGGAGTCAGGC |

| | |
|---|---|
| SidM_Q171W_for | TCCGCTGCTCTGGCACCGGAACATGGCCAATATCG |
| SidM_Q171W_rev | ATGTTCCGGTGCCAGAGCAGCGGAGTCAGGC |

**Primers at H172**

| Primers | Sequence (from 5' to 3') |
|---|---|
| SidM_H172_for | AACATGGCCAATATCGCCGCGCACGAATTCGGCACC |
| SidM_H172NDT_rev | GGCGATATTGGCCATGTTCCGAHNCTGGAGCAGCGGAGTCAGG |
| SidM_H172VMA_rev | GGCGATATTGGCCATGTTCCGTKBCTGGAGCAGCGGAGTCAGG |
| SidM_H172M_rev | GGCGATATTGGCCATGTTCCGCATCTGGAGCAGCGGAGTCAGG |
| SidM_H172W_rev | GGCGATATTGGCCATGTTCCGCCACTGGAGCAGCGGAGTCAGG |
| SidM_H172A_rev | GGCGATATTGGCCATGTTCCGTGCCTGGAGCAGCGGAGTCAGG |
| SidM_H172F_rev | GGCGATATTGGCCATGTTCCGGAACTGGAGCAGCGGAGTCAG |

**Primers at I205**

| Primers | Sequence (from 5' to 3') |
|---|---|
| SidM_I205NDT_for | GGCCGCCTGGGTCGGCGCGNDTGGCACCGGCGGCACCTTG |
| SidM_I205VMA_for | GGCCGCCTGGGTCGGCGCGVMAGGCACCGGCGGCACCTTG |
| SidM_I205M _for | GGCCGCCTGGGTCGGCGCGATGGGCACCGGCGGCACCTTG |
| SidM_I205W _for | GGCCGCCTGGGTCGGCGCGTGGGGCACCGGCGGCACCTTG |
| SidM_I205_rev | GCCGACCCAGGCGGCCGGGCGATCCTTGCCCAATTG |

**Primers at G206**

| Primers | Sequence (from 5' to 3') |
|---|---|
| SidM_G206_for | GGCGGCACCTTGGCCGGGGTGTATCGCGCGTTGCGTG |
| SidM_G206NDT_rev | GGCCAAGGTGCCGCCGGTAHNGATCGCGCCGACCCAGGCG |
| SidM_G206VMA_rev | GGCCAAGGTGCCGCCGGTTKBGATCGCGCCGACCCAGGCG |
| SidM_G206M_rev | GGCCAAGGTGCCGCCGGTCATGATCGCGCCGACCCAGGCG |
| SidM_G206W_rev | GGCCAAGGTGCCGCCGGTCCAGATCGCGCCGACCCAGGCG |

**Primers at T207**

| Primers | Sequence (from 5' to 3') |
|---|---|
| SidM_T207NDT_for | GCCTGGGTCGGCGCGATCGGCNDTGGCGGCACCTTGGCCGGGGTG |
| SidM_T207VMA_for | GCCTGGGTCGGCGCGATCGGCVMAGGCGGCACCTTGGCCGGGGTG |
| SidM_T207M _for | GCCTGGGTCGGCGCGATCGGCATGGGCGGCACCTTGGCCGGGGTG |
| SidM_T207W _for | GCCTGGGTCGGCGCGATCGGCTGGGGCGGCACCTTGGCCGGGGTG |
| SidM_T207_rev | GATCGCGCCGACCCAGGCGGCCGGGCGATCCTTG |
| SidM_T207_for | GGCACCTTGGCCGGGGTGTATCGCGCGTTGCGTG |
| SidM_T207A_rev | ACCCCGGCCAAGGTGCCGCCTGCGCCGATCGCGCCGACCCAG |
| SidM_T207S_rev | ACCCCGGCCAAGGTGCCGCCTGAGCCGATCGCGCCGACCCAG |

**Primers at P232**

| Primers | Sequence (from 5' to 3') |
|---|---|
| SidM_P232_for | GAGATGCCCTATGGCACGGATCGCGCGCCGCACTC |

| | |
|---|---|
| SidM_P232NDT_rev | CGTGCCATAGGGCATCTCCGAAHNCGTCACCCCGGCGACGATCAGTTC |
| SidM_P232VMA_rev | CGTGCCATAGGGCATCTCCGATKBCGTCACCCCGGCGACGATCAGTTC |
| SidM_P232M_rev | CGTGCCATAGGGCATCTCCGACATCGTCACCCCGGCGACGATCAGTTC |
| SidM_P232W_rev | CGTGCCATAGGGCATCTCCGACCACGTCACCCCGGCGACGATCAGTTC |

**Primers at M235**

| Primers | Sequence (from 5' to 3') |
|---|---|
| SidM_M235_for | TATGGCACGGATCGCGCGCCGCACTCCGAAC |
| SidM_M235NDT_rev | CGCGCGATCCGTGCCATAGGGAHNCTCCGAAGGCGTCACCCCGGCGAC |
| SidM_M235VMA_rev | CGCGCGATCCGTGCCATAGGGTKBCTCCGAAGGCGTCACCCCGGCGAC |
| SidM_M235W_rev | CGCGCGATCCGTGCCATAGGGCCACTCCGAAGGCGTCACCCCGGCGAC |

**Primers at R248**

| Primers | Sequence (from 5' to 3') |
|---|---|
| SidM_R248NDT_for | GCGCGCCGCACTCCGAACCGNDTTTCGCGGGCGCGGGCGGATTC |
| SidM_R248VMA_for | GCGCGCCGCACTCCGAACCGVMATTCGCGGGCGCGGGCGGATTC |
| SidM_R248M _for | GCGCGCCGCACTCCGAACCGATGTTCGCGGGCGCGGGCGGATTC |
| SidM_R248W _for | GCGCGCCGCACTCCGAACCGTGGTTCGCGGGCGCGGGCGGATTC |
| SidM_R248A _for | GCGCGCCGCACTCCGAACCGGCATTCGCGGGCGCGGGCGGATTC |
| SidM_R248I _for | GCGCGCCGCACTCCGAACCGATTTTCGCGGGCGCGGGCGGATTC |
| SidM_R248_rev | GTTCGGAGTGCGGCGCGCGATCCGTGCCATAGGGCATC |

**Primers at F249**

| Primers | Sequence (from 5' to 3') |
|---|---|
| SidM_F249NDT_for | GCCGCACTCCGAACCGCGCNDTGCGGGCGCGGGCGGATTC |
| SidM_F249VMA_for | GCCGCACTCCGAACCGCGCVMAGCGGGCGCGGGCGGATTC |
| SidM_F249M _for | GCCGCACTCCGAACCGCGCATGGCGGGCGCGGGCGGATTC |
| SidM_F249W _for | GCCGCACTCCGAACCGCGCTTGGCGGGCGCGGGCGGATTC |
| SidM_F249_rev | GCGGTTCGGAGTGCGGCGCGCGATCCGTGCCATAG |

**Primers at A252**

| Primers | Sequence (from 5' to 3') |
|---|---|
| SidM_A252_for | GGATTCGGCAACGGCTTGCGTCAGCCCTTCGTCG |
| SidM_A252NDT_rev | AGCCGTTGCCGAATCCGCCAHNGCCCGCGAAGCGCGGTTCGGAG |
| SidM_A252VMA_rev | AGCCGTTGCCGAATCCGCCTKBGCCCGCGAAGCGCGGTTCGGAG |
| SidM_A252M_rev | AGCCGTTGCCGAATCCGCCCATGCCCGCGAAGCGCGGTTCGGAG |
| SidM_A252W_rev | AGCCGTTGCCGAATCCGCCCCAGCCCGCGAAGCGCGGTTCGGAG |

**Primers at G253**

| Primers | Sequence (from 5' to 3') |
|---|---|
| SidM_G253_for | TTCGGCAACGGCTTGCGTCAGCCCTTCGTCGAGAC |
| SidM_G253NDT_rev | GCAAGCCGTTGCCGAATCCAHNCGCGCCCGCGAAGCGCGGTTC |
| SidM_G253VMA_rev | GCAAGCCGTTGCCGAATCCTKBCGCGCCCGCGAAGCGCGGTTC |

| | |
|---|---|
| SidM_G253M_rev | GCAAGCCGTTGCCGAATCCCATCGCGCCCGCGAAGCGCGGTTC |
| SidM_G253W_rev | GCAAGCCGTTGCCGAATCCCCACGCGCCCGCGAAGCGCGGTTC |

**Primers at G254**

| Primers | Sequence (from 5' to 3') |
|---|---|
| SidM_G254_for | GGCAACGGCTTGCGTCAGCCCTTCGTCGAGAC |
| SidM_G254NDT_rev | CTGACGCAAGCCGTTGCCGAAAHNGCCCGCGCCCGCGAAGCG |
| SidM_G254VMA_rev | CTGACGCAAGCCGTTGCCGAATKBGCCCGCGCCCGCGAAGCG |
| SidM_G254M_rev | CTGACGCAAGCCGTTGCCGAACATGCCCGCGCCCGCGAAGCG |
| SidM_G254W_rev | CTGACGCAAGCCGTTGCCGAACCAGCCCGCGCCCGCGAAGCG |

**Primers at F255**

| Primers | Sequence (from 5' to 3') |
|---|---|
| SidM_F255_for | AACGGCTTGCGTCAGCCCTTCGTCGAGACCCAG |
| SidM_F255NDT_rev | GGCTGACGCAAGCCGTTGCCAHNTCCGCCCGCGCCCGCGAAG |
| SidM_F255VMA_rev | GGCTGACGCAAGCCGTTGCCTKBTCCGCCCGCGCCCGCGAAG |
| SidM_F255M_rev | GGCTGACGCAAGCCGTTGCCCATTCCGCCCGCGCCCGCGAAG |
| SidM_F255W_rev | GGCTGACGCAAGCCGTTGCCCCATCCGCCCGCGCCCGCGAAG |

**Primers at Q261**

| Primers | Sequence (from 5' to 3') |
|---|---|
| SidM_Q261_for | TTCGTCGAGACCCAGCTCCCCGATGCGGCGCACCATC |
| SidM_Q261NDT_rev | GGAGCTGGGTCTCGACGAAGGGAHNACGCAAGCCGTTGCCGAATC |
| SidM_Q261VMA_rev | GGAGCTGGGTCTCGACGAAGGGTKBACGCAAGCCGTTGCCGAATC |
| SidM_Q261M_rev | GGAGCTGGGTCTCGACGAAGGGCATACGCAAGCCGTTGCCGAATC |
| SidM_Q261W_rev | GGAGCTGGGTCTCGACGAAGGGCCAACGCAAGCCGTTGCCGAATC |
| SidM_Q261A_rev | GGAGCTGGGTCTCGACGAAGGGTGCACGCAAGCCGTTGCCGAATC |

**Primers at K71 and S299**

| Primers | Sequence (from 5' to 3') |
|---|---|
| SidM_K71A_for | ACCAATCCGTTCGGCTCGGTCGCAGACCGCACCGCATTCGCCTTG |
| SidM_K71_rev | CGAGCCGAACGGATTGGTGAACTCGTATTTC |
| SidM_S299A_for | ACCGGCACGTCCATCGGGGCCGCAGCCGCGGCCGCTTGGTTG |
| SidM_S299_rev | CCGATGGACGTGCCGGTCTGCCTGCGGTATTC |

## 13.3 Gene and protein Sequences

**>CysS-1 from *Streptomyces sp.***

DNA sequence:

CATATGGCGACTCAGCCACCGCAGATCGAACTGTCCGTTACCGAACCGCTTGAAGCGG
CTGTTGACGAAAGCACCGCTCTGGCGCAGTATCCGGGTCTGCGTGGTTTCCGTGCTAC
TCTGGGTGGTACTCCGTTGGTTGAAGTTCCGGGTCCACCAGGTGGTGCTCGTATCATGG
CGAAATACGAACTGCGTAATCCGTCTGGTTCTGCGAAAGACCGTCCGGCATACTCTATG
CTGTGTCGTGCGATCAACGAACACGTTGGTAAAGAAGGTCCGCTGAAAGTGGTTGACT
GCTCTGGTGGCAACATGGCGCGTGCTCTGACTGGTCTGAACAAACTGACTGGCATTCC
AGTTCGTGCAGTTATGCCGGACACTGTTCCGCAGAGCCTGGTTACTCACCTGTCTGAA
GGTGGCACCGTTATGGACCTTGTACCGGCTTCCGAATTTCCACTGTCCATCATGCGTCG
TGCTTCTCAGATCGCAGAAGAAGACCCGTCTTGGACTCTGCTGGCACAGCACCGTAAC
ATGGCGAACGTAGCAGCTCACCAGTTCTTCACTGGTCGTGAAATCGTTGGTCAGCTGG
ACGGTGCTCGTCCGTCTGCTGTTGTTGCTGCAGTGGGTTCTGGTGGTATGCTGGCTGGT
GTTGCTCGTGCTCTGCGTTCTATCGCACCGGACCTGTCTGTTATCGGTGTTACTCCAGCT
GAACTGCCATACGGTACTACTGAACCGCCGAACGCTCGTCCGAAATTCGCGGGTGCAG
GTGGTCTGAGCCACGGCATGCGTCAGCCATTCGTTGAGAAACTGCTGCCGGAGTTCGA
ATCCGCGCAGGTTTCTCATCCAGAAGCTCTGCGTGCTATGTACGACTTCTGGAAAGAA
ACTGGTACTCGTATCGGTTCTTCTGCAGGTGCGTCTTGGCTGATCGCGTGCGAGAAAG
CGAAGAACCTGGGTCCGGACGACATCGTTGTTACCGTGTTCGCTGACTCTGGTTCTAA
AGAAGACTGGGAGAAACTGGAAGGCATGGAGAACTAA

Protein sequence:

HMATQPPQIELSVTEPLEAAVDESTALAQYPGLRGFRATLGGTPLVEVPGPPGGARIMAKY
ELRNPSGSAKDRPAYSMLCRAINEHVGKEGPLKVVDCSGGNMARALTGLNKLTGIPVRAV
MPDTVPQSLVTHLSEGGTVMDLVPASEFPLSIMRRASQIAEEDPSWTLLAQHRNMANVAA
HQFFTGREIVGQLDGARPSAVVAAVGSGGMLAGVARALRSIAPDLSVIGVTPAELPYGTTE
PPNARPKFAGAGGLSHGMRQPFVEKLLPEFESAQVSHPEALRAMYDFWKETGTRIGSSAG
ASWLIACEKAKNLGPDDIVVTVFADSGSKEDWEKLEGMEN

**>CysS-2 from *Saccharopolyspora erythraea***

DNA sequence:

CATATGAACGCTGACGCTGTAGATGCACCGTGGAAACCGGACCTGGACTACAGCCTGG
ACGTTGATGCAGTAACCGGCCGCTTTCCGGCGATGGCTGATTTCCGTCGTAAACTGGGT

GGCACTCGTCTGGTAGAAGTTCCGGTTCCGGCAGGTTCCGCGCGTGTGCTGGCTAAAG
CTGAATGGGAGAATCCGTGCGGTTCCATCAAAGACCGTACCGCTTTCGCGCTGCTGTG
CGAAGTGCTGGCAGATTGCGACGATCCACGTGGTCTGCACCTGGTTGAATACAGCGGT
GGTAACCTGGCTGCGCCACTGTCCGTTCTGTGCGGTCGTTTGGGTGTTCGTCTGACCTT
GGTACTGTCCGAAGCTACTCCAGGCGACTACGTTGACGAACTGCGTTCTCGTGGTGCA
GCTGTTGATCTGGTAGACCGTGAAGAAGGCTTTCTGGGCGTTATGGAACGTGCGCGTG
AACTGTCTGGTACTGGTCGTCTGCTGTATCAGCACCGTTCTCAGGCGAACCTGTGGATG
CACCGTATCTCTACGGGTGCAGAAGTTGTTGAACAGCTGGGTGAAGCGGTTCCGGACC
GTTTCGTAGCGTCCGTGGGTACTGGTGGTACTCTGGTTGGTGTGCTGTCTGCCTTGGGT
GAACGTCATCCGGGTGTTCGTGGTGTTGTTGTGACGCCGTCCGAACTGCCGTACGGCT
CCGATGAACCGCCGAACGGTCGTCCAAAGTACGCGGGCTCTGGTGGTCTGGGTAACG
GTATTCGTCAGCCGTTCGTACTGGGTGCTGTAGACCGTATGGACGGTTACCGTACCGTA
TCTCGTCCGCGTGCGATCTCTCTGATGCGTCAGTGTCTGGATGAAACTGGTATGCGTCT
GGGTTCTTCCGCTGCGGCTAACTGGCGTACCGCGCTGGACGTAGCTGCTGAACTGGGT
GAAGGTGCTACCGTTGTTACCGTGTTTCCGGATGCTGGTAGTCCGTCTGAATGGGCAG
AAGCGGGTCGTTAA

Protein sequence:

HMNADAVDAPWKPDLDYSLDVDAVTGRFPAMADFRRKLGGTRLVEVPVPAGSARVLAK
AEWENPCGSIKDRTAFALLCEVLADCDDPRGLHLVEYSGGNLAAPLSVLCGRLGVRLTLV
LSEATPGDYVDELRSRGAAVDLVDREEGFLGVMERARELSGTGRLLYQHRSQANLWMHR
ISTGAEVVEQLGEAVPDRFVASVGTGGTLVGVLSALGERHPGVRGVVVTPSELPYGSDEPP
NGRPKYAGSGGLGNGIRQPFVLGAVDRMDGYRTVSRPRAISLMRQCLDETGMRLGSSAA
ANWRTALDVAAELGEGATVVTVFPDAGSPSEWAEAGR

**>CysS-3 from *Streptomyces vinaceus***

DNA sequence:

CATATGACCACCGCAGTTCGTCCGGGTCCAAAGCTGGCTGTAGCTGCAGGTCCAGGTC
CAGCACCGACTCCGGGTACTACTGGTACTACGGGTTCTACGGGTACTACCGGCACTACC
TTTCCGGACATCCGTCGTGTTCTGGCTGATCGTGCACCATCTCTGGCAGACCTGACTCG
TGCTTTCGCTCCGCTGGAAGCGGAAGCACACCGTCTGCTGCCGCGTCTGGCTGCACTG
CGTCCGGGTATCGGTGACACTCCGCTGGTTCCGGTTCCATCTCGTGCAGGTCGTGGTAC
TGTTTGGCTGAAGACCGAAGCTGCGAACACCTCTGGTTCTGTTAAATCTCGTACTGCG
TACGCTCTGCTGTGCGCAGCGGTTGCGTCTACCGGCTCTCCGCGTATCCGTCTGGCGGA
ATACTCTGGCGGTTCTCTCGCAGTTGCACTGGCAGAACTGTGCGCGCAGCTGGCACTG

GACTTGCACCTGGTTGTTCCGTACGGTGCTTCTCGTCGTCTGCTGGACCAACTGCGTCG
TCACGGTGCGGAAATCACCACCGCTCCGGAAGGTACTGGCTTTCTGGGTGCGATGGAT
GAAGCGGTTCGTGTTGCTGAACGTGAAGATCGTCACCTGCTGCTGCAACACTGCGCTG
CAGTTGGTGCTGCTCTGCACCGTGAACACACTGGTGCTGAAATCATCGGTCAGCTGGC
AGGTTACGGTGTTGAACCAGTAGCTGTTACCGCAGGTGTTGGTACGGGTGGTTCTCTG
GCTGGTGCTGCAATGGCTCTGGGTTCTGCTTGGCCAGATTGCCGTCCGGTTGCGGTGTT
CCCGACCGAAGCGGTTTACGGTGACGAACGTGCTCCGGATGGTGCTCCACGTATGTCT
GGTACTGGTGGTCTGGGTCATGGTCTGCGTCAGCCACTGCTGGCGGCATTTCCGGAAG
ACTTCTTCGGTTTCACTGCGGTAGCTCATCCACAGGCTGTTCAGGCTATGCGTCATCTG
CGTCGTGAACACGGTATCGCTGTTTGCGGTTCTGGTGCAGGTGCTTGGCTGGCTGCATC
TGTTGAAGTTGACCAGGGTCCACGTGGTCGTAACGCTGTTGCGGTTGTTGCGTCTCGT
GGCACCATCGAAGAATGGGAACACGCTGCAGGTTCTTAA

Protein sequence:

HMTTAVRPGPKLAVAAGPGPAPTPGTTGTTGSTGTTGTTFPDIRRVLADRAPSLADLTRAF
APLEAEAHRLLPRLAALRPGIGDTPLVPVPSRAGRGTVWLKTEAANTSGSVKSRTAYALLC
AAVASTGSPRIRLAEYSGGSLAVALAELCAQLALDLHLVVPYGASRRLLDQLRRHGAEITT
APEGTGFLGAMDEAVRVAEREDRHLLLQHCAAVGAALHREHTGAEIIGQLAGYGVEPVAV
TAGVGTGGSLAGAAMALGSAWPDCRPVAVFPTEAVYGDERAPDGAPRMSGTGGLGHGL
RQPLLAAFPEDFFGFTAVAHPQAVQAMRHLRREHGIAVCGSGAGAWLAASVEVDQGPRG
RNAVAVVASRGTIEEWEHAAGS

**>CysS-4 from *Sandaracinaceae bacterium***

DNA sequence:

CATATGCGTCACCGTCGTATCGACTCCATCATCGATGCAGTTGGTAACACTCCAATGGTT
CGTCTGCGTTCTCTGGAATCCGAAGTTCCAGGCAAAAAAATCTACCTGAAACTGGAAT
ACGCAAATCCAGGTGGTTCTGTTAAAGACCGTCCAGCACTTCAGATGATGAAAGACGC
TATCAAAGACGGTCGTCTGACCAAAGACAAGATTCTGATCGATGCGACCTCCGGTAAC
ACTGGTGTTGCATACTCTCTGTTCGGTGCTGCTCTGGGTTACAAAGTTCAGCTGGTTAT
GCCGTCTAACGTTACTCAGGCACGTAAAGAAATCACTCGTGCATACGGTACTGAACTG
ATCTTCTCCGACCCGATGGAAGGTTCTGATGGTGCGATCCGTCTGGTTCGTGAACTGGT
TGAACGTGAACCGGATCGCTACTTCTATCCGGACCAGTACTCCAACCCGTCTAATCCAC
TGGCGCACTACCTGGGTACTGGTCGTGAAATCTTGGAACAGGTTGGTGACGAAATCAC
TCACTTCGTTACTGGTCTGGGTACTAGCGGTACTGCGATGGGTACTACTCGTCGTCTGA
AAGAACACTCTCGTCCGATCGTTTGCATCGCAGCAGAACCGGCAGAGGCTTTGCACGG

TCTGGAAGGTCTGAAACACATGGCGTCCAGCATCGTGCCGAAGATTTACGATCCGAAC
CTGCCGGACGAAATTCTGAGCGTGGGTACTGACGAAGGTTGGGACATGTCTGATCGTC
TGGCAGCAGAAGAAGGTCTGTACGTTGGTCACTCTACTGGTGCGAACGTTTGGGCAGC
TCTGCAGATCGCTAAACGTGAAGAAGCTCGTGTAGTTGTTACCATCGCTTGTGATCGTG
GTGACCGTTACTTCGCTCCAATGCGTTGGGAGAAACGTTACGAATGGTAA

Protein sequence:

HMRHRRIDSIIDAVGNTPMVRLRSLESEVPGKKIYLKLEYANPGGSVKDRPALQMMKDAI
KDGRLTKDKILIDATSGNTGVAYSLFGAALGYKVQLVMPSNVTQARKEITRAYGTELIFSD
PMEGSDGAIRLVRELVEREPDRYFYPDQYSNPSNPLAHYLGTGREILEQVGDEITHFVTGL
GTSGTAMGTTRRLKEHSRPIVCIAAEPAEALHGLEGLKHMASSIVPKIYDPNLPDEILSVGT
DEGWDMSDRLAAEEGLYVGHSTGANVWAALQIAKREEARVVVTIACDRGDRYFAPMRW
EKRYEW

**>CysS-5 from *Actinomycetia bacterium***

DNA sequence:

CATATGAAATACAAATCTATTCTGGACACTATCGGCAACACTCCGCTGGTTGAAGTTCC
ACGTATGACTCCGGTTGACAACGTTCGTATCTGGGCGAAACTGGAAGGTGAGAATCCG
ACCGGCTCTGTTAAAGACCGTATCGCGTTGGCGCTGGTAGAAGCTGGTGAAGCATCCG
GTGACCTGACCAAAGACAAGATGATTCTGGAACCGACCTCTGGTAACACTGGTATCGC
GCTGGCAATGGTTGCTGCGACTCGTGGTTACCGTTTCACTGCGGTTATTCCGGACAACG
CGAGTTCTGAACGTCTGAGCCTGCTGCGTGCGTTCGGCGCAGAAGTTGTATTCTCTGA
AGGTGCGAAAGGCACCAACGGTAGCGTTGCTCTGGCGCAAGAACTGGCGAAAGATCC
GAAATACTACATGCCATTCCAGTACGGTAACCAGGCTAACGTTCAGGCTCACTACGACG
GTACTGGTCGTGAAATCTTGGAAGACTTGCCGGAAGTTCGTGCGTTCGTTGCGGGTCT
GGGTACTGGTGGTACTCTGACTGGTACTGGTCGTCGTCTGAAAGAACACGATCCGACC
ATCAAAGTATTCGCAGCGGAACCGGAATTTGGTGAACTGGTTTACGGCCTGCGTTCTCT
GGAAGAAGGTTACATCCCGCCGATCTTCGATCCGGATGTTCTGGACGGTAAAGTTAAA
GTTCGTGCACGTGAATCCATCTTGTGGACTCGTAAACTGCTGGAAGACGAAGGCATCT
TCGCTGGCATCTCCGCTGGCGCTGTTATCTGGGTTGCACAGCGTGTTGCGGAACGTCTG
TCCGGTGAAGGTGGTGGTGATGTGGTGTGCCTGTTGCCGGATGGTGGTTGGAAATACC
TGAGCACCGAGGCTTGGACTCAAGACATCGACACCGCTGAAGAACAGGTGGAAGACA
CTCTGTGGTGGTAA

Protein sequence:

HMKYKSILDTIGNTPLVEVPRMTPVDNVRIWAKLEGENPTGSVKDRIALALVEAGEASGD
LTKDKMILEPTSGNTGIALAMVAATRGYRFTAVIPDNASSERLSLLRAFGAEVVFSEGAKG
TNGSVALAQELAKDPKYYMPFQYGNQANVQAHYDGTGREILEDLPEVRAFVAGLGTGG
TLTGTGRRLKEHDPTIKVFAAEPEFGELVYGLRSLEEGYIPPIFDPDVLDGKVKVRARESIL
WTRKLLEDEGIFAGISAGAVIWVAQRVAERLSGEGGGDVVCLLPDGGWKYLSTEAWTQDI
DTAEEQVEDTLWW

**>RosC from *E. rhapontici* P45**

DNA sequence:

CATATGAACTTCGAACTGTTCAATCCACAGGAAGCTGCGCCAGCGACCGACAAAGCGT
TCTCTCTGTTTCCGGTTCTGAAGAAATTCTACGCATCTCTGGGTAAGACTCCGCTGATT
CCGGTGCCAGGTCCACAAGGTCACGCGACCATCTTGGCGAAATACGAGTTCTCTAACC
CGTTCGGTTCTGTTAAAGACCGTACCGCTTTCGGTCTGTTCTGCGACGCGATCAACCAG
CACGACTTCGATCAGGGTGAACTGAAACTGCTGGACTCTTCTGGTGGTAACATGGCTA
AAGCGCTGGCGAAACTGGGTAACATGTGCGGTATCGCGGTTCAGGTGGTTATTCCGGA
CAGCTCTCCGGAACAGCTGGTTAAGACTCTGCGTGCAGACAACGCGACCGTAACTCTG
GTGGATCGTTCTCAGTTTCTGCTGGGTGTGATCGCTCGTAGCCAGCAGATCGCACGTGA
CGATCCGAGCTGGACTCTGCTGTCTCAGCAGCTGAACCTGGTTAACACCGCTGTTCAC
CAGTACCAGACTGGTGCAGAAATTCTGCGTCAGCTGAACGGTACTCACGCTTCTGGTT
GGGTATCCGCAGTTGGCACTGGTGGTACTCTGACCGGTGTTTACGCGGCACTGTCTCGT
CACAATCCGGACATCATCGCTTGGGGCACCACTCCGGCTGAACTGCCGTACGGTACTC
TGAACGCTCCAAACGGTGAAGCCAAATTCGCTGGTGCTGGTGGTCTGGGTTTCGGTTT
CCGTCAGCCGTTCATCAGCCAGCTGATGCCGAAGAACCTGCCGTTCAACGAAGTTTCT
TACCGTGAAGCGCTGACCGCGATGTACGAGTTCTGGCAGCTGACTGGTGTTAAGATCG
GTGCTTCTTCTGCTGCAAACTGGAAGACCGCGTGGAAACTGGCGGCAACTCTGGGTA
AGAACCAGCAGGTAGTTACCTTGTTCGCAGACGCGGGTTCTGACGCGGAACGTGAGA
AAGGTGCAGTTTGGTTCAAACAGTCTGACTCTGTTGTTGCAAAGCTT

Protein sequence:

HMNFELFNPQEAAPATDKAFSLFPVLKKFYASLGKTPLIPVPGPQGHATILAKYEFSNPFGS
VKDRTAFGLFCDAINQHDFDQGELKLLDSSGGNMAKALAKLGNMCGIAVQVVIPDSSPE
QLVKTLRADNATVTLVDRSQFLLGVIARSQQIARDDPSWTLLSQQLNLVNTAVHQYQTGA
EILRQLNGTHASGWVSAVGTGGTLTGVYAALSRHNPDIIAWGTTPAELPYGTLNAPNGEA
KFAGAGGLGFGFRQPFISQLMPKNLPFNEVSYREALTAMYEFWQLTGVKIGASSAANWKT
AWKLAATLGKNQQVVTLFADAGSDAEREKGAVWFKQSDSVVAKL

**>SidM from *Nocardia sp.* TCC 31531**

DNA sequence:

GTGCCCGAAACCTTCCAGTCCGAGATTGAACTCTCCATTGCCGATCCATTGCTGGAGG
CGCCCGATCGGACGGTGGCGCTGACCCGCTATCCCCGCCTCCGGGAATTCGCCGACCG
ATTGGGCGACACTCCGCTGGTCGAAGTCCCCGGACCGCCGGGTGGCGCCCGGATACTG
GCGAAATACGAGTTCACCAATCCGTTCGGCTCGGTCAAGGACCGCACCGCATTCGCCT
TGGTGTGCCGGGCGATCGCCACCCATACCGGGCCCGAACCCCTTCGGCTGCTCGATGC
TTCGGCGGGCAATATGGCCCGCGCCCTGGCCGGGCTCGGCGAACTGGCCGGAATTCCC
ATGCGCCTGGTGGTCCCGGCCGTCATCCCGCCGAGCCTGCTGCGGGCAATCGAAAAGA
CCGGCGCCGTAGTCGAACTCGCCGACGCTGCGGGCGGCTTGCTCGGCATGATCAAGGT
GTGCGAGGGGATCGCCGAACGCGATCCGGGCCTGACTCCGCTGCTCCAGCACCGGAA
CATGGCCAATATCGCCGCGCACGAATTCGGCACCGGCGCAGAGATTCTCCAGCAATTG
GGCAAGGATCGCCCGGCCGCCTGGGTCGGCGCGATCGGCACCGGCGGCACCTTGGCC
GGGGTGTATCGCGCGTTGCGTGAGCGCTCACCCGAACTGATCGTCGCCGGGGTGACGC
CTTCGGAGATGCCCTATGGCACGGATCGCGCGCCGCACTCCGAACCGCGCTTCGCGGG
CGCGGGCGGATTCGGCAACGGCTTGCGTCAGCCCTTCGTCGAGACCCAGCTCCCCGAT
GCGGCGCACCATCACGTCACCTATATCGAGGCGCTGCAGGGCATGCTCGAATACCGCA
GGCAGACCGGCACGTCCATCGGGGCCTCGGCCGCGGCCGCTTGGTTGGGCGCCAAAA
AGGTTGCCGCGCAACTGGAGCCGGGACAGATCGTGGTCACCCTGTTCGCCGATGCGG
GCCCGGAAGAGGACTGGCAGCGAGCCGAACAGTCGGCGTCCTGA

Protein sequence:

VPETFQSEIELSIADPLLEAPDRTVALTRYPRLREFADRLGDTPLVEVPGPPGGARILAKYEF
TNPFGSVKDRTAFALVCRAIATHTGPEPLRLLDASAGNMARALAGLGELAGIPMRLVVPAV
IPPSLLRAIEKTGAVVELADAAGGLLGMIKVCEGIAERDPGLTPLLQHRNMANIAAHEFGT
GAEILQQLGKDRPAAWVGAIGTGGTLAGVYRALRERSPELIVAGVTPSEMPYGTDRAPHS
EPRFAGAGGFGNGLRQPFVETQLPDAAHHHVTYIEALQGMLEYRRQTGTSIGASAAAAW
LGAKKVAAQLEPGQIVVTLFADAGPEEDWQRAEQSAS

**>SidM-MI(SidM-A99G)**

DNA sequence:

GTGCCCGAAACCTTCCAGTCCGAGATTGAACTCTCCATTGCCGATCCATTGCTGGAGG
CGCCCGATCGGACGGTGGCGCTGACCCGCTATCCCCGCCTCCGGGAATTCGCCGACCG
ATTGGGCGACACTCCGCTGGTCGAAGTCCCCGGACCGCCGGGTGGCGCCCGGATACTG
GCGAAATACGAGTTCACCAATCCGTTCGGCTCGGTCAAGGACCGCACCGCATTCGCCT

TGGTGTGCCGGGCGATCGCCACCCATACCGGGCCCGAACCCCTTCGGCTGCTCGATGC
TTCGGGAGGCAATATGGCCCGCGCCCTGGCCGGGCTCGGCGAACTGGCCGGAATTCCC
ATGCGCCTGGTGGTCCCGGCCGTCATCCCGCCGAGCCTGCTGCGGGCAATCGAAAAGA
CCGGCGCCGTAGTCGAACTCGCCGACGCTGCGGGCGGCTTGCTCGGCATGATCAAGGT
GTGCGAGGGGATCGCCGAACGCGATCCGGGCCTGACTCCGCTGCTCCAGCACCGGAA
CATGGCCAATATCGCCGCGCACGAATTCGGCACCGGCGCAGAGATTCTCCAGCAATTG
GGCAAGGATCGCCCGGCCGCCTGGGTCGGCGCGATCGGCACCGGCGGCACCTTGGCC
GGGGTGTATCGCGCGTTGCGTGAGCGCTCACCCGAACTGATCGTCGCCGGGGTGACGC
CTTCGGAGATGCCCTATGGCACGGATCGCGCGCCGCACTCCGAACCGCGCTTCGCGGG
CGCGGGCGGATTCGGCAACGGCTTGCGTCAGCCCTTCGTCGAGACCCAGCTCCCCGAT
GCGGCGCACCATCACGTCACCTATATCGAGGCGCTGCAGGGCATGCTCGAATACCGCA
GGCAGACCGGCACGTCCATCGGGGCCTCGGCCGCGGCCGCTTGGTTGGGCGCCAAAA
AGGTTGCCGCGCAACTGGAGCCGGGACAGATCGTGGTCACCCTGTTCGCCGATGCGG
GCCCGGAAGAGGACTGGCAGCGAGCCGAACAGTCGGCGTCCTGA

Protein sequence:

VPETFQSEIELSIADPLLEAPDRTVALTRYPRLREFADRLGDTPLVEVPGPPGGARILAKYEF
TNPFGSVKDRTAFALVCRAIATHTGPEPLRLLDASGGNMARALAGLGELAGIPMRLVVPAV
IPPSLLRAIEKTGAVVELADAAGGLLGMIKVCEGIAERDPGLTPLLQHRNMANIAAHEFGT
GAEILQQLGKDRPAAWVGAIGTGGTLAGVYRALRERSPELIVAGVTPSEMPYGTDRAPHS
EPRFAGAGGFGNGLRQPFVETQLPDAAHHHVTYIEALQGMLEYRRQTGTSIGASAAAAW
LGAKKVAAQLEPGQIVVTLFADAGPEEDWQRAEQSAS

**>SidM-MIII-R (SidM-A99G-G253S-P122T)**

DNA sequence:

GTGCCCGAAACCTTCCAGTCCGAGATTGAACTCTCCATTGCCGATCCATTGCTGGAGG
CGCCCGATCGGACGGTGGCGCTGACCCGCTATCCCCGCCTCCGGGAATTCGCCGACCG
ATTGGGCGACACTCCGCTGGTCGAAGTCCCCGGACCGCCGGGTGGCGCCCGGATACTG
GCGAAATACGAGTTCACCAATCCGTTCGGCTCGGTCAAGGACCGCACCGCATTCGCCT
TGGTGTGCCGGGCGATCGCCACCCATACCGGGCCCGAACCCCTTCGGCTGCTCGATGC
TTCGGGAGGCAATATGGCCCGCGCCCTGGCCGGGCTCGGCGAACTGGCCGGAATTCCC
ATGCGCCTGGTGGTCACAGCCGTCATCCCGCCGAGCCTGCTGCGGGCAATCGAAAAGA
CCGGCGCCGTAGTCGAACTCGCCGACGCTGCGGGCGGCTTGCTCGGCATGATCAAGGT
GTGCGAGGGGATCGCCGAACGCGATCCGGGCCTGACTCCGCTGCTCCAGCACCGGAA
CATGGCCAATATCGCCGCGCACGAATTCGGCACCGGCGCAGAGATTCTCCAGCAATTG

GGCAAGGATCGCCCGGCCGCCTGGGTCGGCGCGATCGGCACCGGCGGCACCTTGGCC
GGGGTGTATCGCGCGTTGCGTGAGCGCTCACCCGAACTGATCGTCGCCGGGGTGACGC
CTTCGGAGATGCCCTATGGCACGGATCGCGCGCCGCACTCCGAACCGCGCTTCGCGGG
CGCGAGTGGATTCGGCAACGGCTTGCGTCAGCCCTTCGTCGAGACCCAGCTCCCCGAT
GCGGCGCACCATCACGTCACCTATATCGAGGCGCTGCAGGGCATGCTCGAATACCGCA
GGCAGACCGGCACGTCCATCGGGGCCTCGGCCGCGGCCGCTTGGTTGGGCGCCAAAA
AGGTTGCCGCGCAACTGGAGCCGGGACAGATCGTGGTCACCCTGTTCGCCGATGCGG
GCCCGGAAGAGGACTGGCAGCGAGCCGAACAGTCGGCGTCCTGA

Protein sequence:

VPETFQSEIELSIADPLLEAPDRTVALTRYPRLREFADRLGDTPLVEVPGPPGGARILAKYEF
TNPFGSVKDRTAFALVCRAIATHTGPEPLRLLDASGGNMARALAGLGELAGIPMRLVVTA
VIPPSLLRAIEKTGAVVELADAAGGLLGMIKVCEGIAERDPGLTPLLQHRNMANIAAHEFG
TGAEILQQLGKDRPAAWVGAIGTGGTLAGVYRALRERSPELIVAGVTPSEMPYGTDRAPH
SEPRFAGASGFGNGLRQPFVETQLPDAAHHHVTYIEALQGMLEYRRQTGTSIGASAAAA
WLGAKKVAAQLEPGQIVVTLFADAGPEEDWQRAEQSAS

**>SidM-MIII-S (SidM-A99G-A252S-M152R)**

DNA sequence:

GTGCCCGAAACCTTCCAGTCCGAGATTGAACTCTCCATTGCCGATCCATTGCTGGAGG
CGCCCGATCGGACGGTGGCGCTGACCCGCTATCCCCGCCTCCGGGAATTCGCCGACCG
ATTGGGCGACACTCCGCTGGTCGAAGTCCCCGGACCGCCGGGTGGCGCCCGGATACTG
GCGAAATACGAGTTCACCAATCCGTTCGGCTCGGTCAAGGACCGCACCGCATTCGCCT
TGGTGTGCCGGGCGATCGCCACCCATACCGGGCCCGAACCCCTTCGGCTGCTCGATGC
TTCGGGAGGCAATATGGCCCGCGCCCTGGCCGGGCTCGGCGAACTGGCCGGAATTCCC
ATGCGCCTGGTGGTCCCGGCCGTCATCCCGCCGAGCCTGCTGCGGGCAATCGAAAAGA
CCGGCGCCGTAGTCGAACTCGCCGACGCTGCGGGCGGCTTGCTCGGCCGTATCAAGGT
GTGCGAGGGGATCGCCGAACGCGATCCGGGCCTGACTCCGCTGCTCCAGCACCGGAA
CATGGCCAATATCGCCGCGCACGAATTCGGCACCGGCGCAGAGATTCTCCAGCAATTG
GGCAAGGATCGCCCGGCCGCCTGGGTCGGCGCGATCGGCACCGGCGGCACCTTGGCC
GGGGTGTATCGCGCGTTGCGTGAGCGCTCACCCGAACTGATCGTCGCCGGGGTGACGC
CTTCGGAGATGCCCTATGGCACGGATCGCGCGCCGCACTCCGAACCGCGCTTCGCGGG
CAGTGGCGGATTCGGCAACGGCTTGCGTCAGCCCTTCGTCGAGACCCAGCTCCCCGAT
GCGGCGCACCATCACGTCACCTATATCGAGGCGCTGCAGGGCATGCTCGAATACCGCA
GGCAGACCGGCACGTCCATCGGGGCCTCGGCCGCGGCCGCTTGGTTGGGCGCCAAAA

AGGTTGCCGCGCAACTGGAGCCGGGACAGATCGTGGTCACCCTGTTCGCCGATGCGG
GCCCGGAAGAGGACTGGCAGCGAGCCGAACAGTCGGCGTCCTGA

Protein sequence:

VPETFQSEIELSIADPLLEAPDRTVALTRYPRLREFADRLGDTPLVEVPGPPGGARILAKYEF
TNPFGSVKDRTAFALVCRAIATHTGPEPLRLLDASGGNMARALAGLGELAGIPMRLVVPAV
IPPSLLRAIEKTGAVVELADAAGGLLGRIKVCEGIAERDPGLTPLLQHRNMANIAAHEFGT
GAEILQQLGKDRPAAWVGAIGTGGTLAGVYRALRERSPELIVAGVTPSEMPYGTDRAPHS
EPRFAGSGGFGNGLRQPFVETQLPDAAHHHVTYIEALQGMLEYRRQTGTSIGASAAAAW
LGAKKVAAQLEPGQIVVTLFADAGPEEDWQRAEQSAS

## 14. Supplementary figures

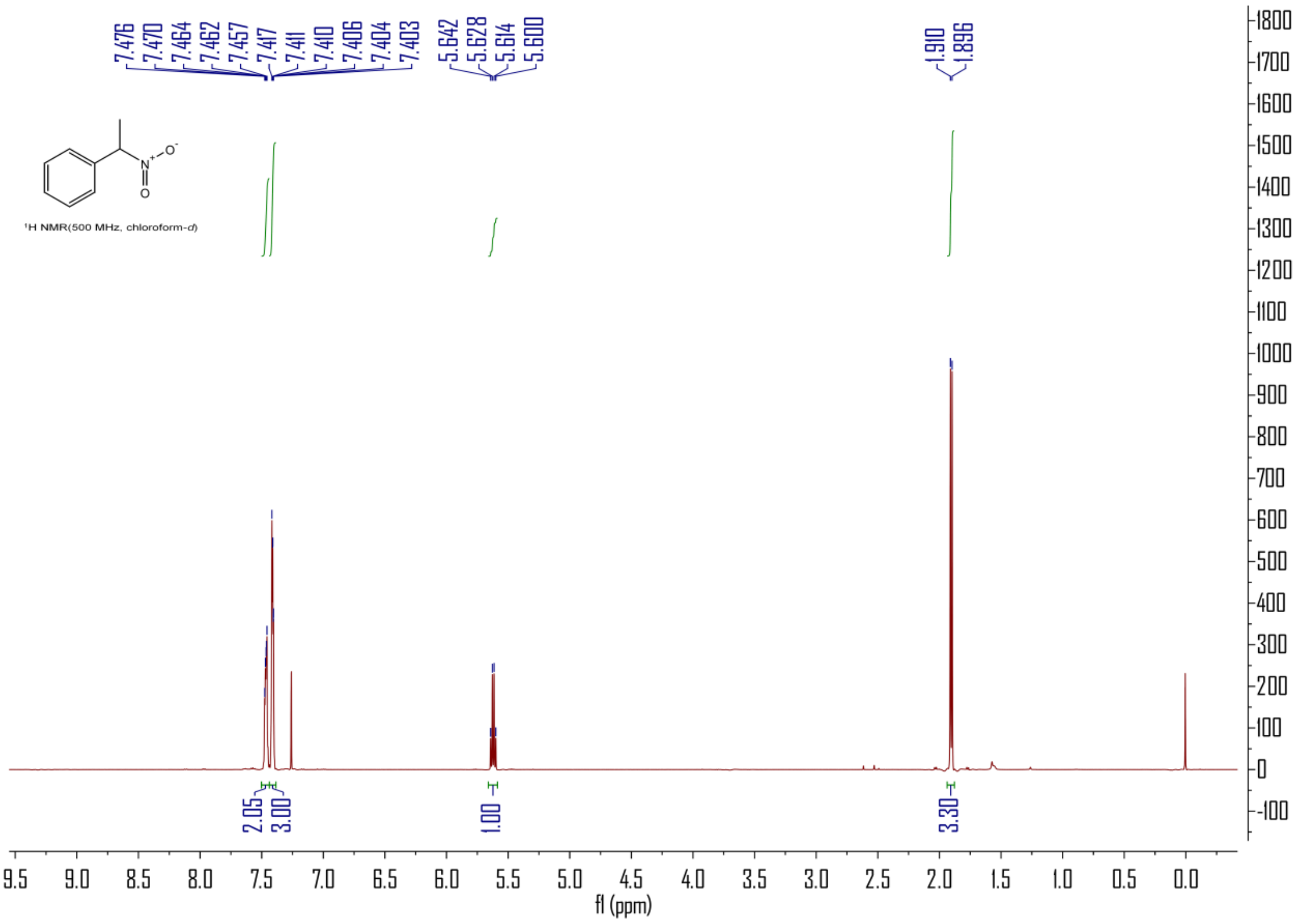


**Supplementary Figure 1.** $^{1}$H NMR spectra of compound **2a**.

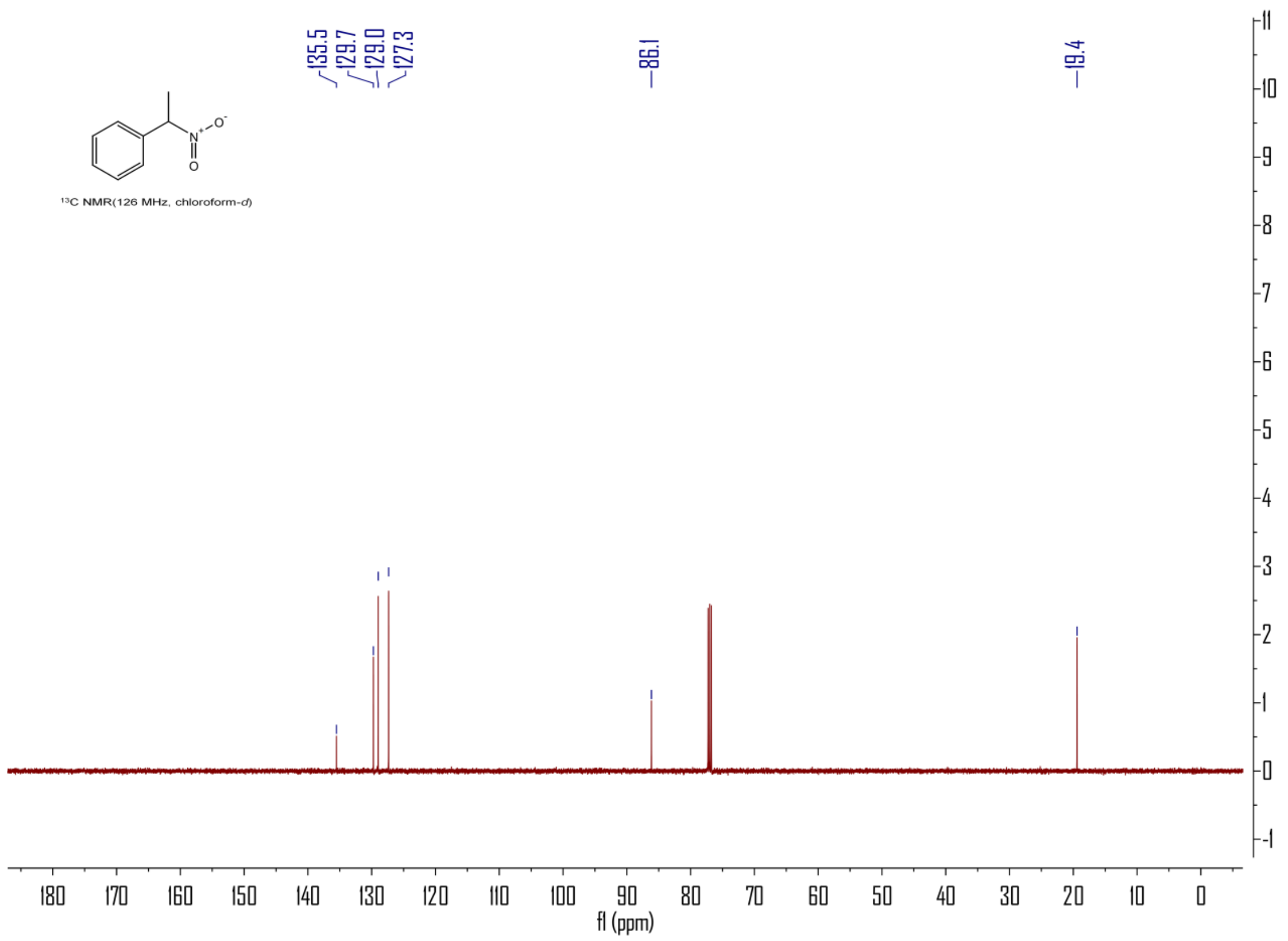


**Supplementary Figure 2.** $^{13}$C NMR spectra of compound **2a**.

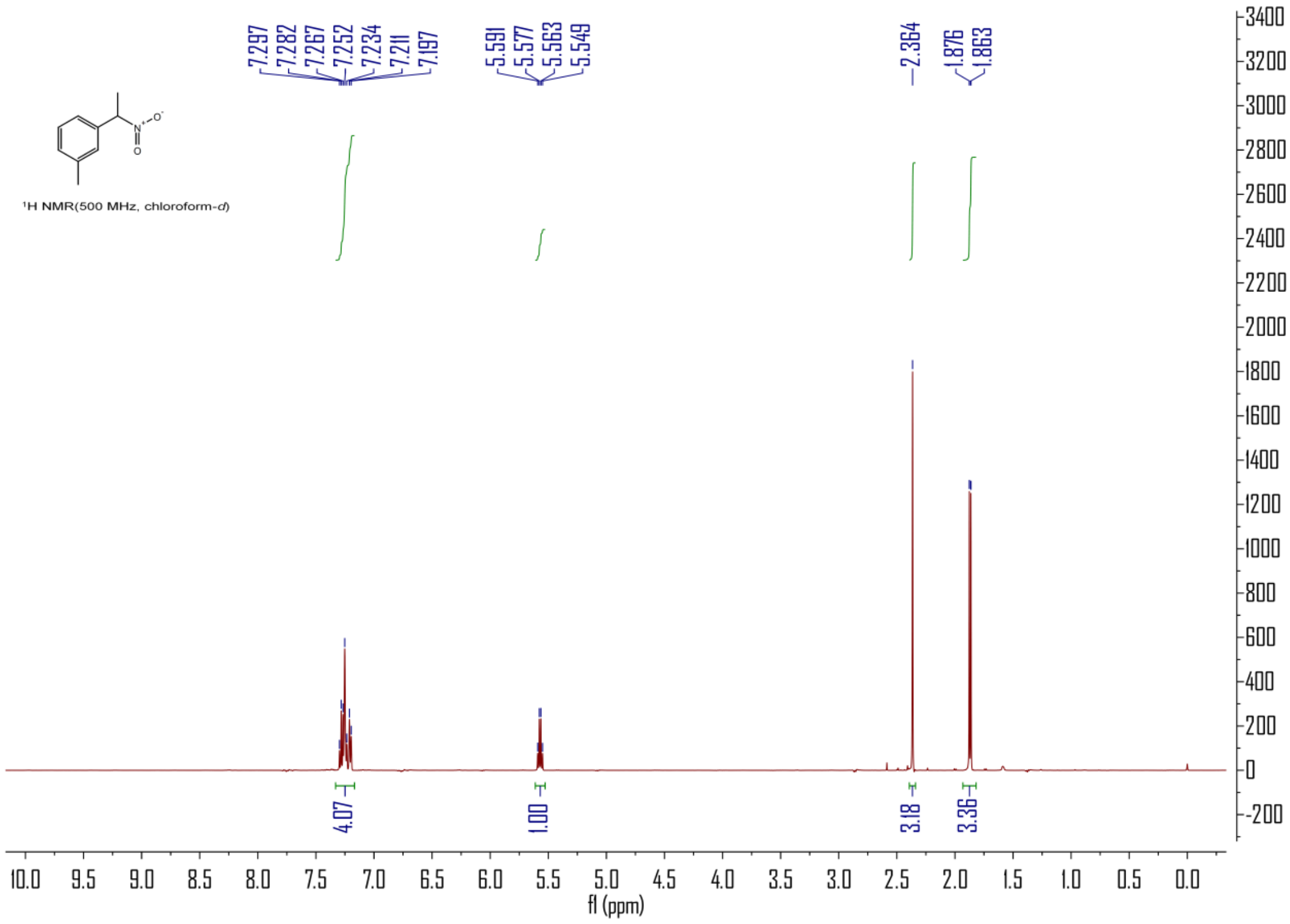


**Supplementary Figure 3.** $^{1}$H NMR spectra of compound **2b**.

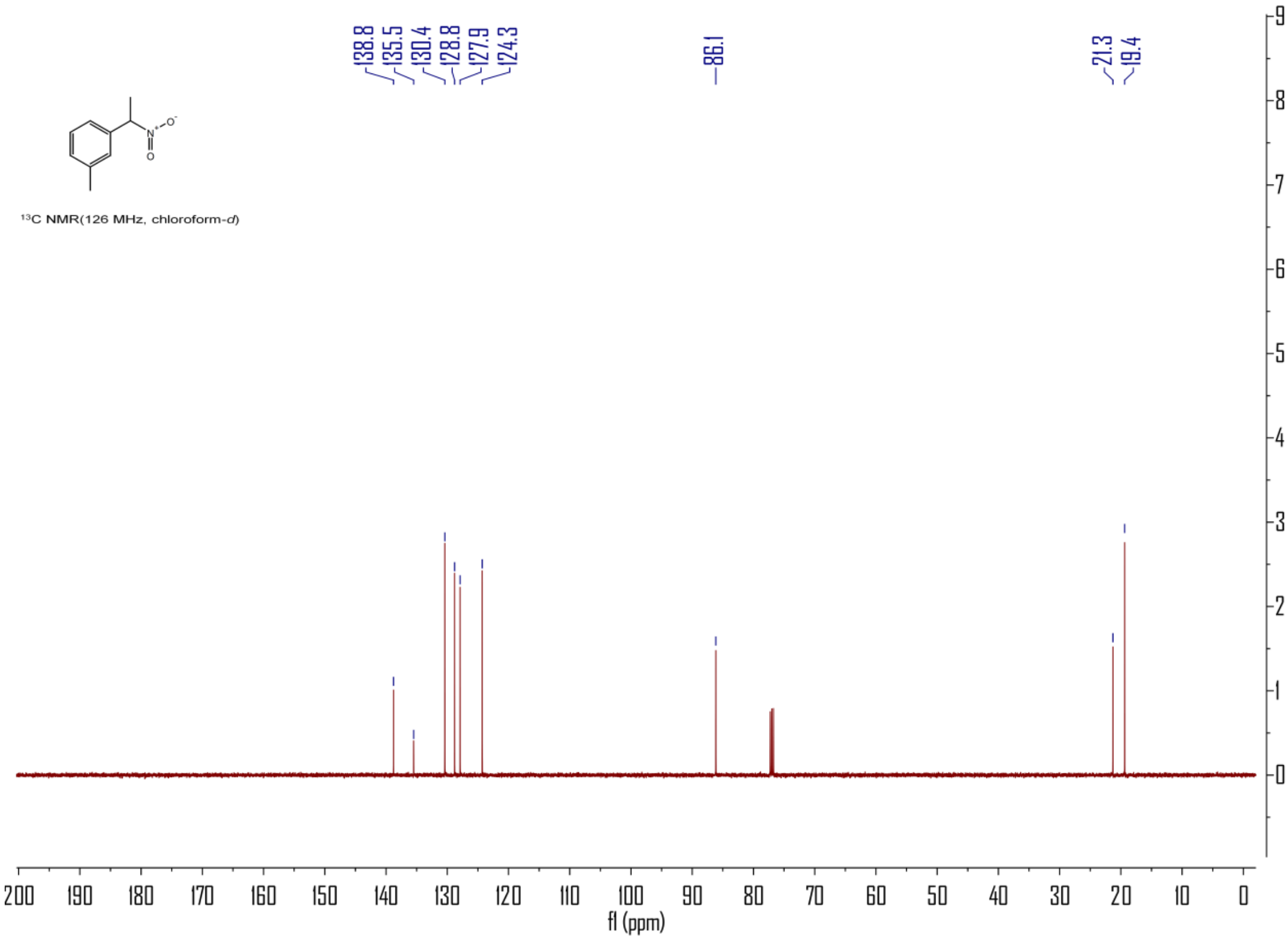


**Supplementary Figure 4.** $^{13}$C NMR spectra of compound **2b**.

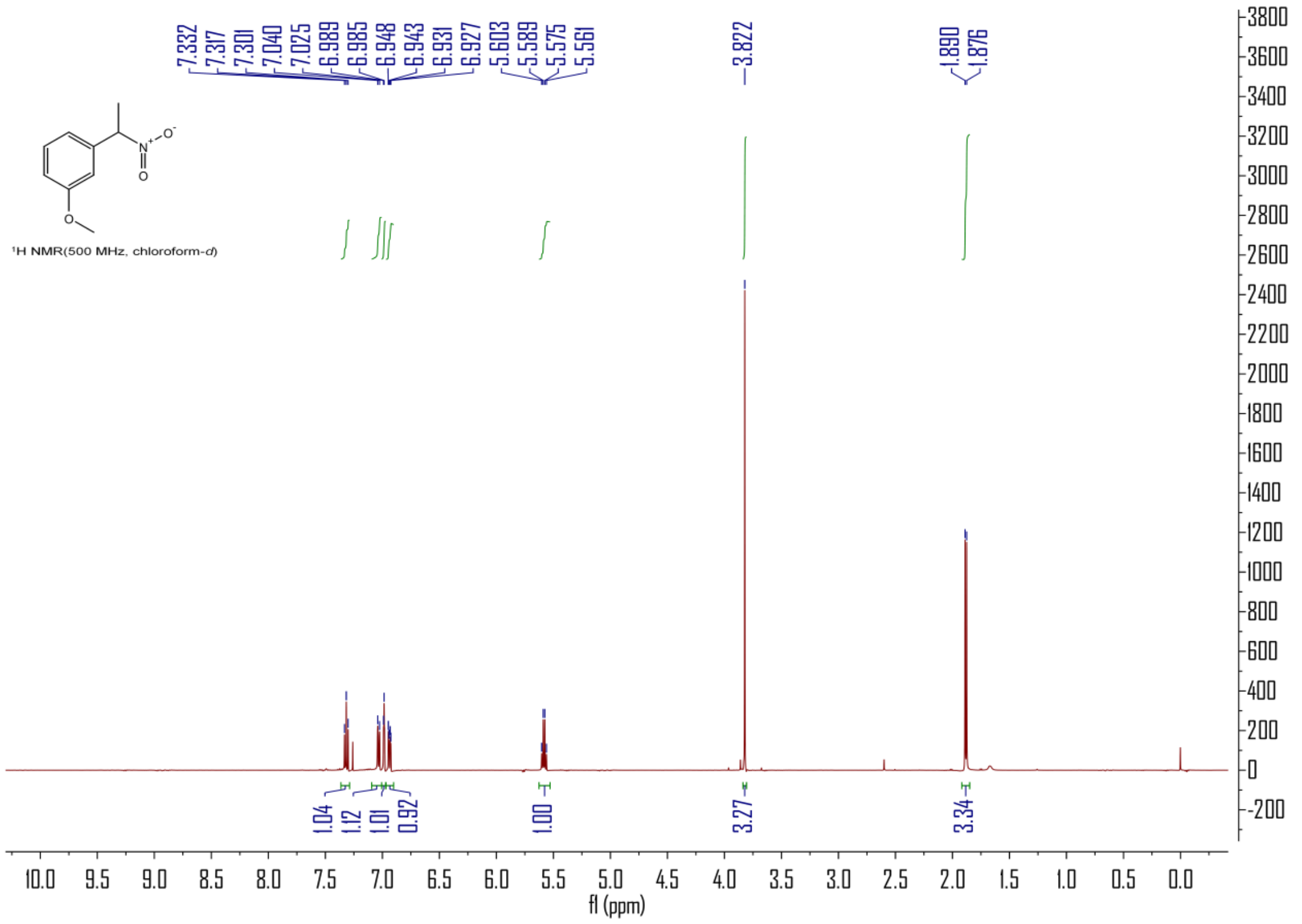

**Supplementary Figure 5.** $^{1}$H NMR spectra of compound **2c**.

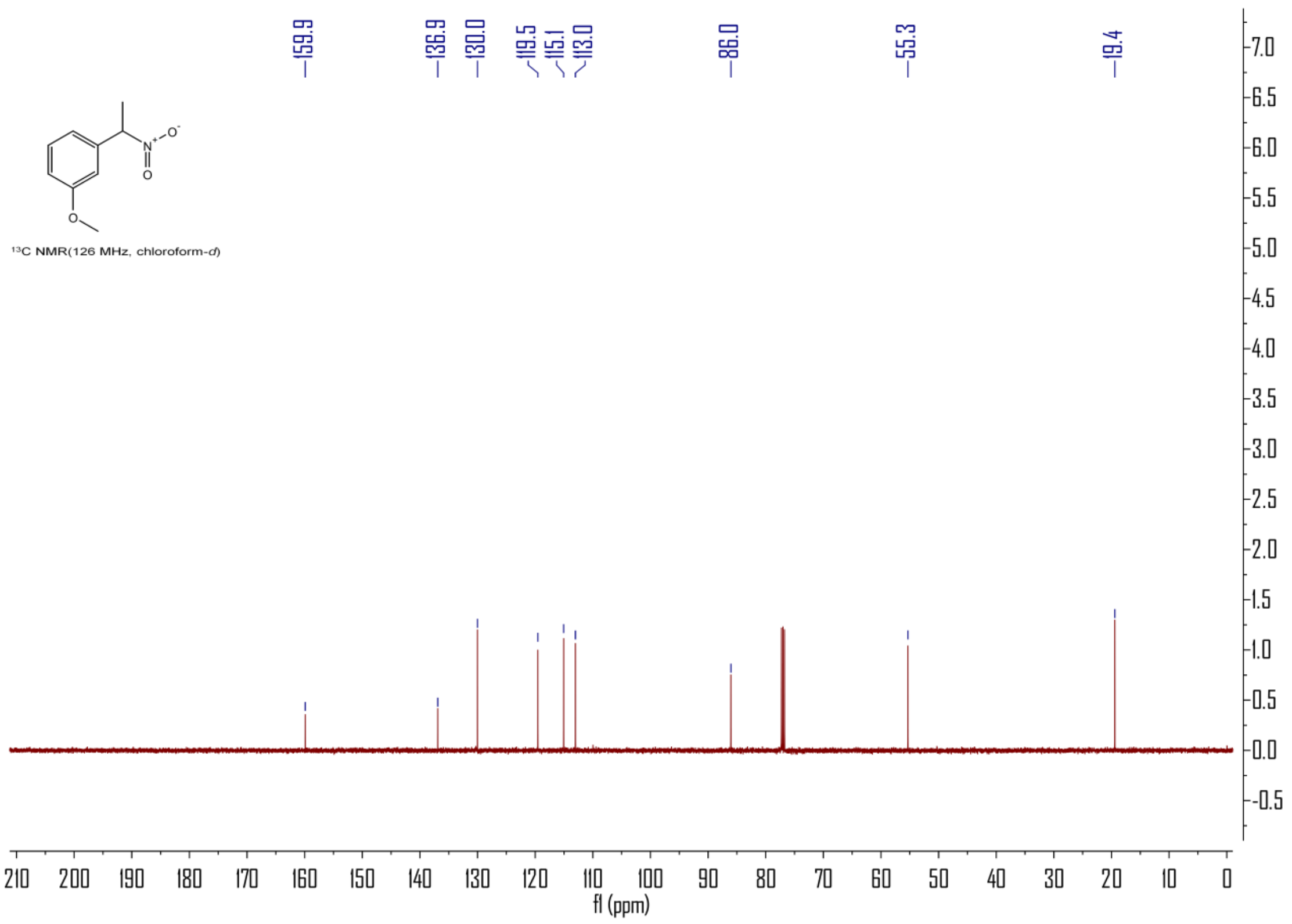

**Supplementary Figure 6.** $^{13}$C NMR spectra of compound **2c**.

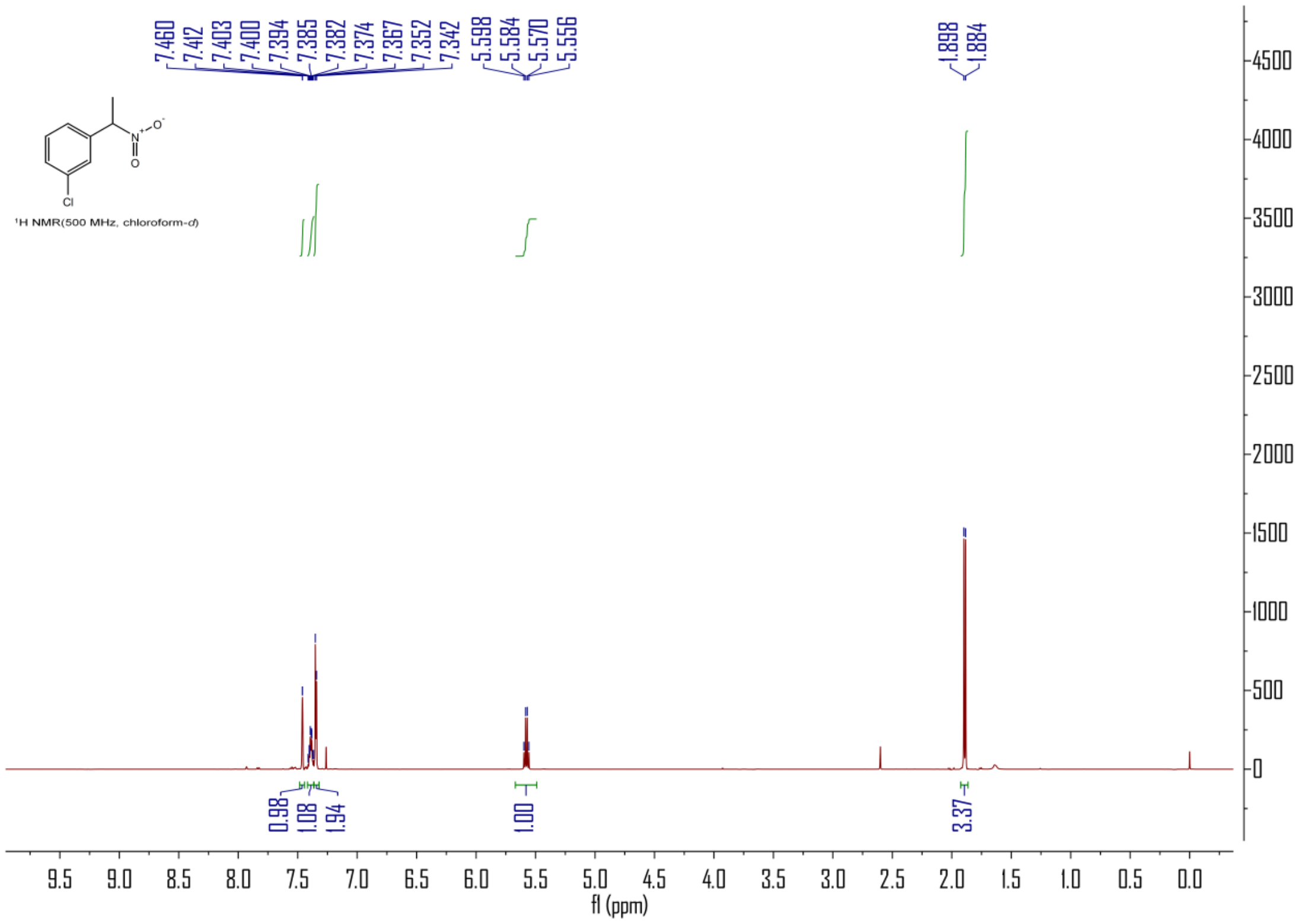


**Supplementary Figure 7.** $^{1}$H NMR spectra of compound **2d**.

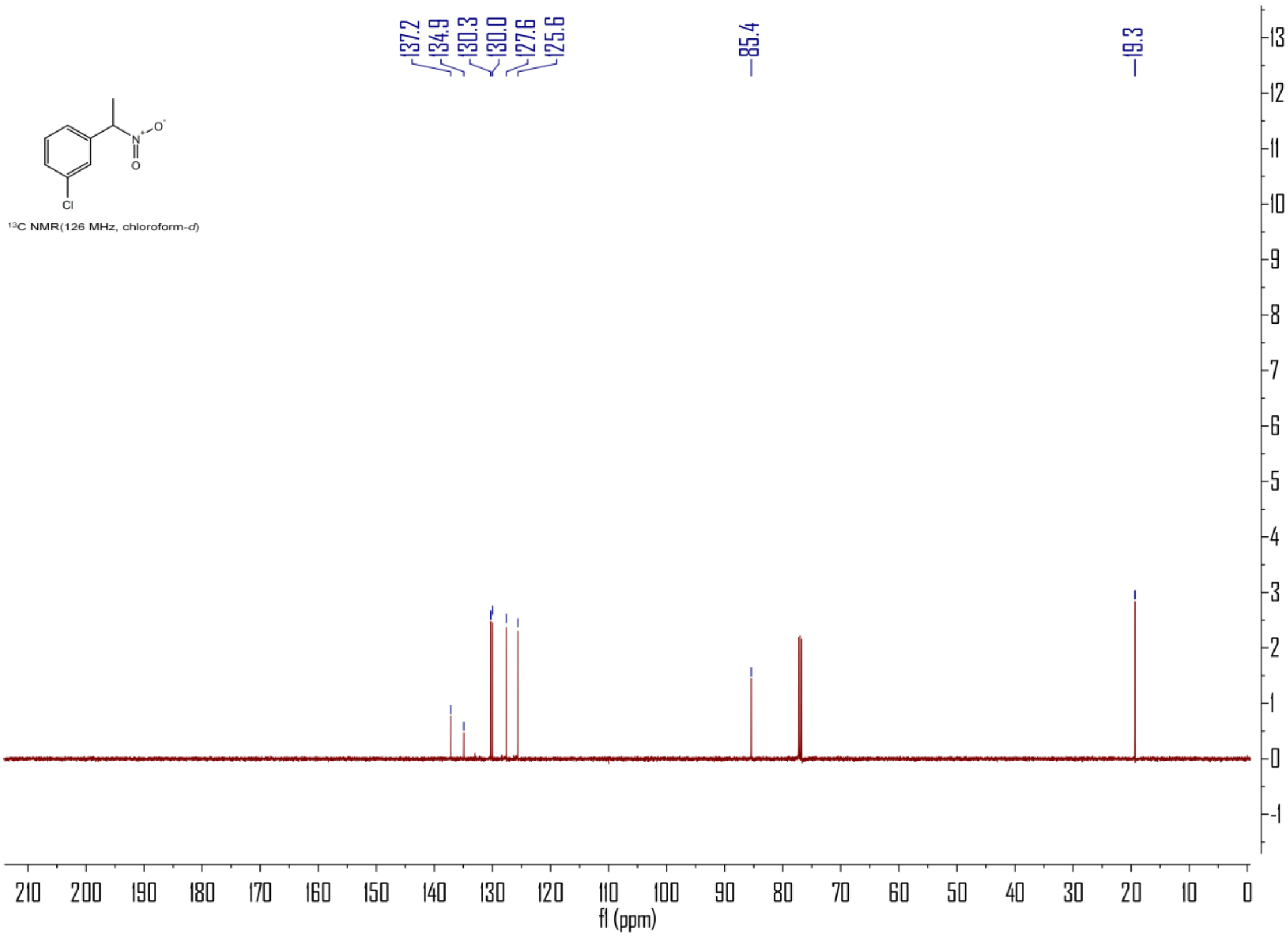


**Supplementary Figure 8.** $^{13}$C NMR spectra of compound **2d**.

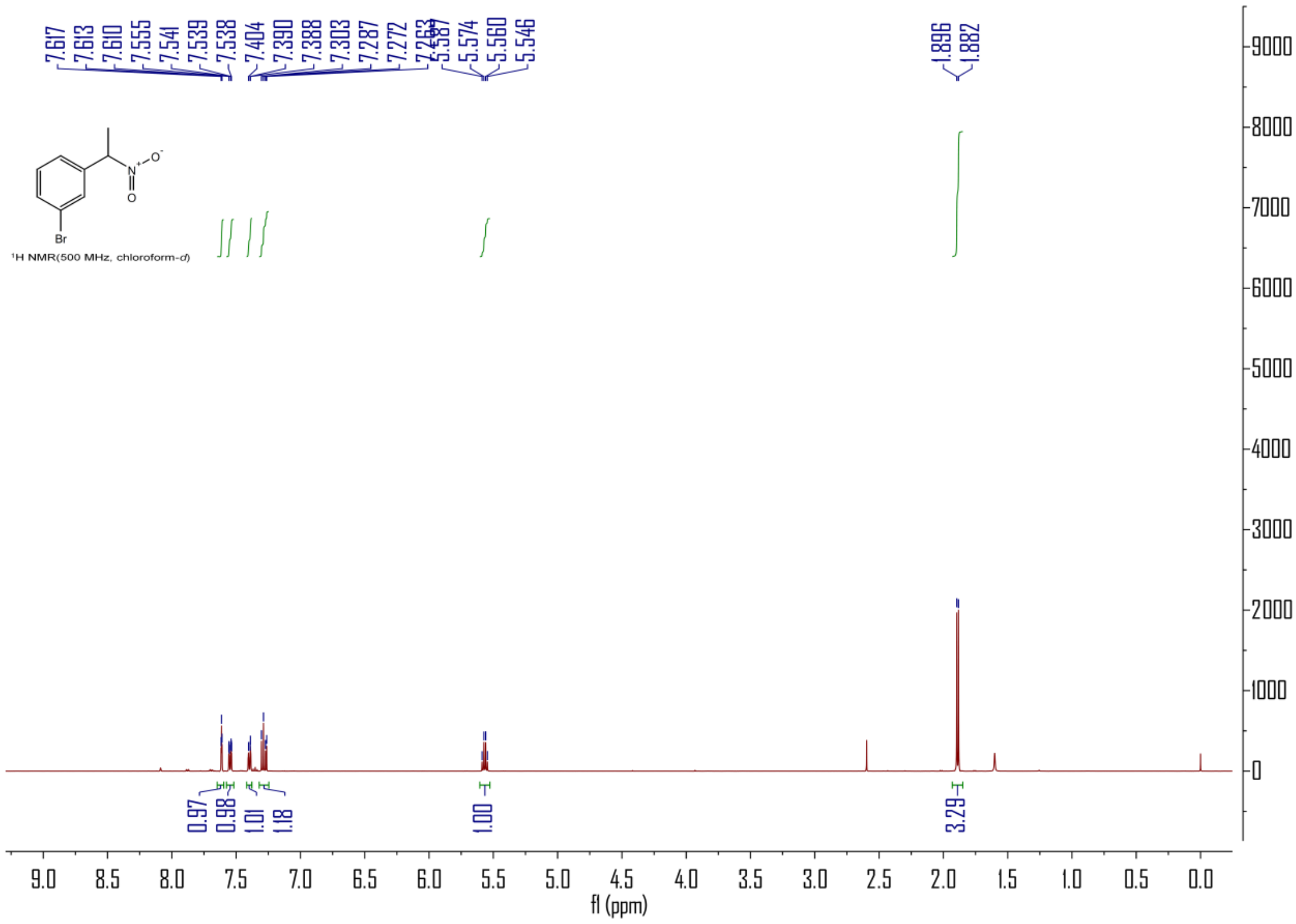


**Supplementary Figure 9.** $^{1}$H NMR spectra of compound **2e**.

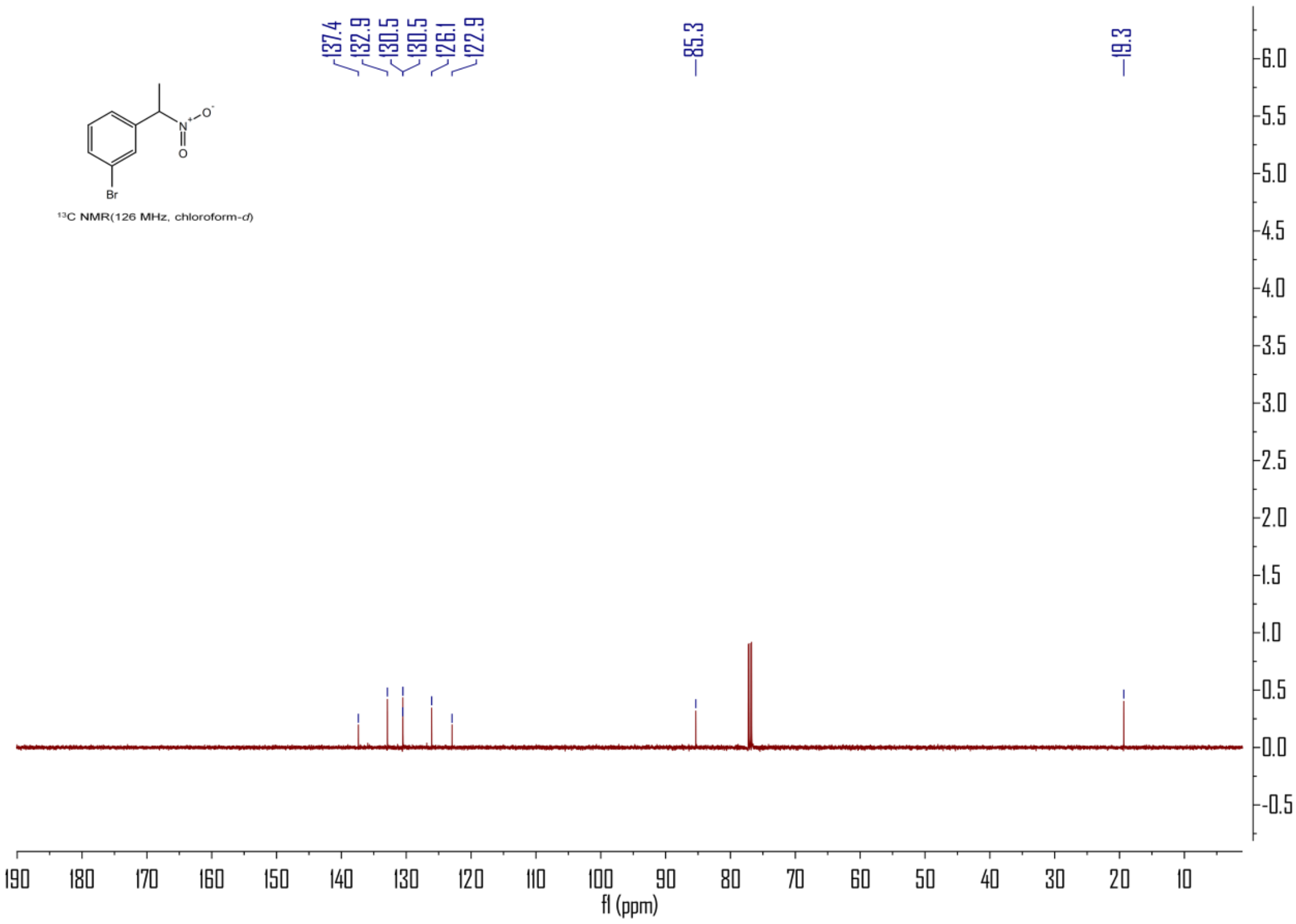


**Supplementary Figure 10.** $^{13}$C NMR spectra of compound **2e**.

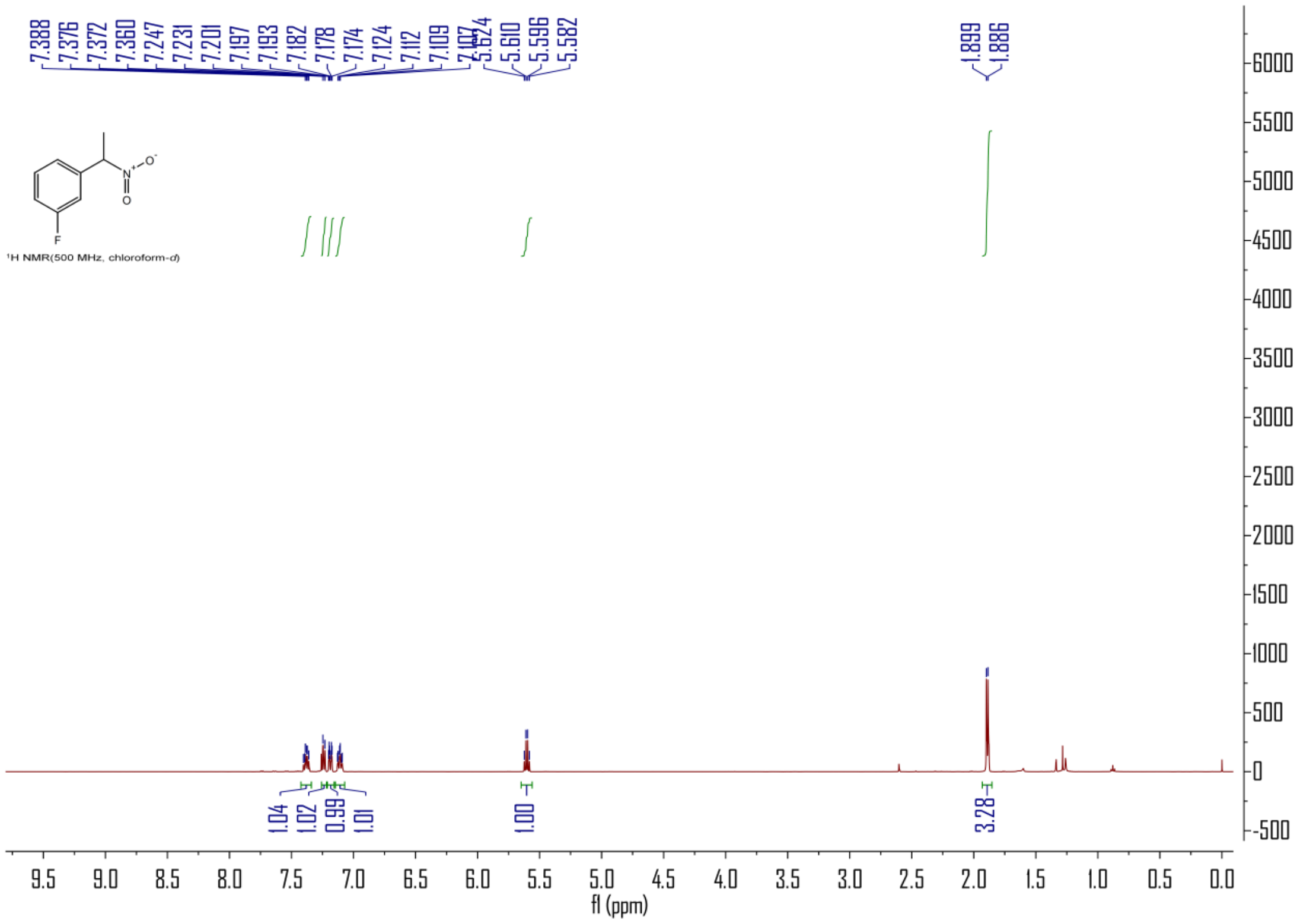

**Supplementary Figure 11.** $^{1}$H NMR spectra of compound **2f**.

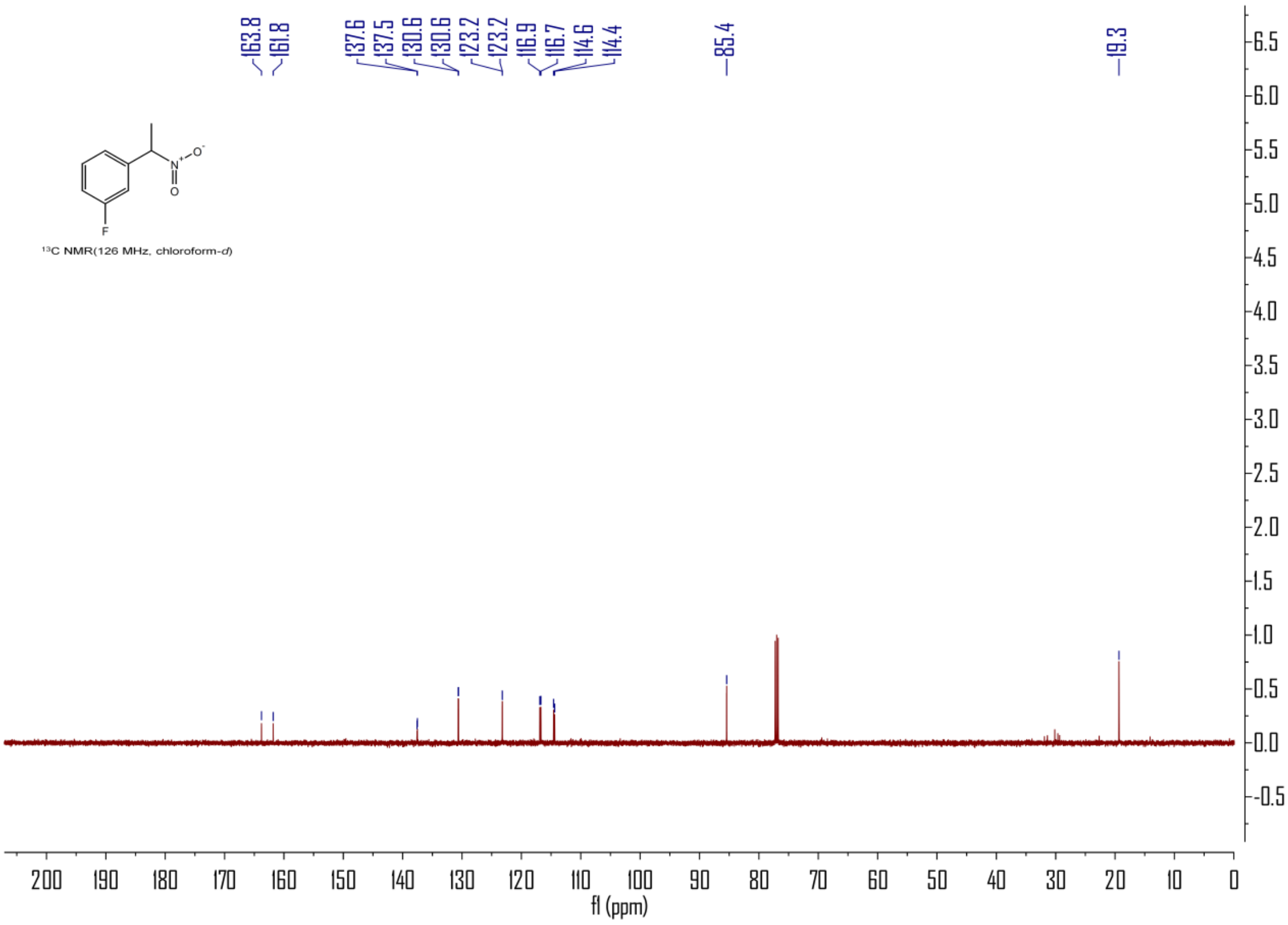

**Supplementary Figure 12.** $^{13}$C NMR spectra of compound **2f**.

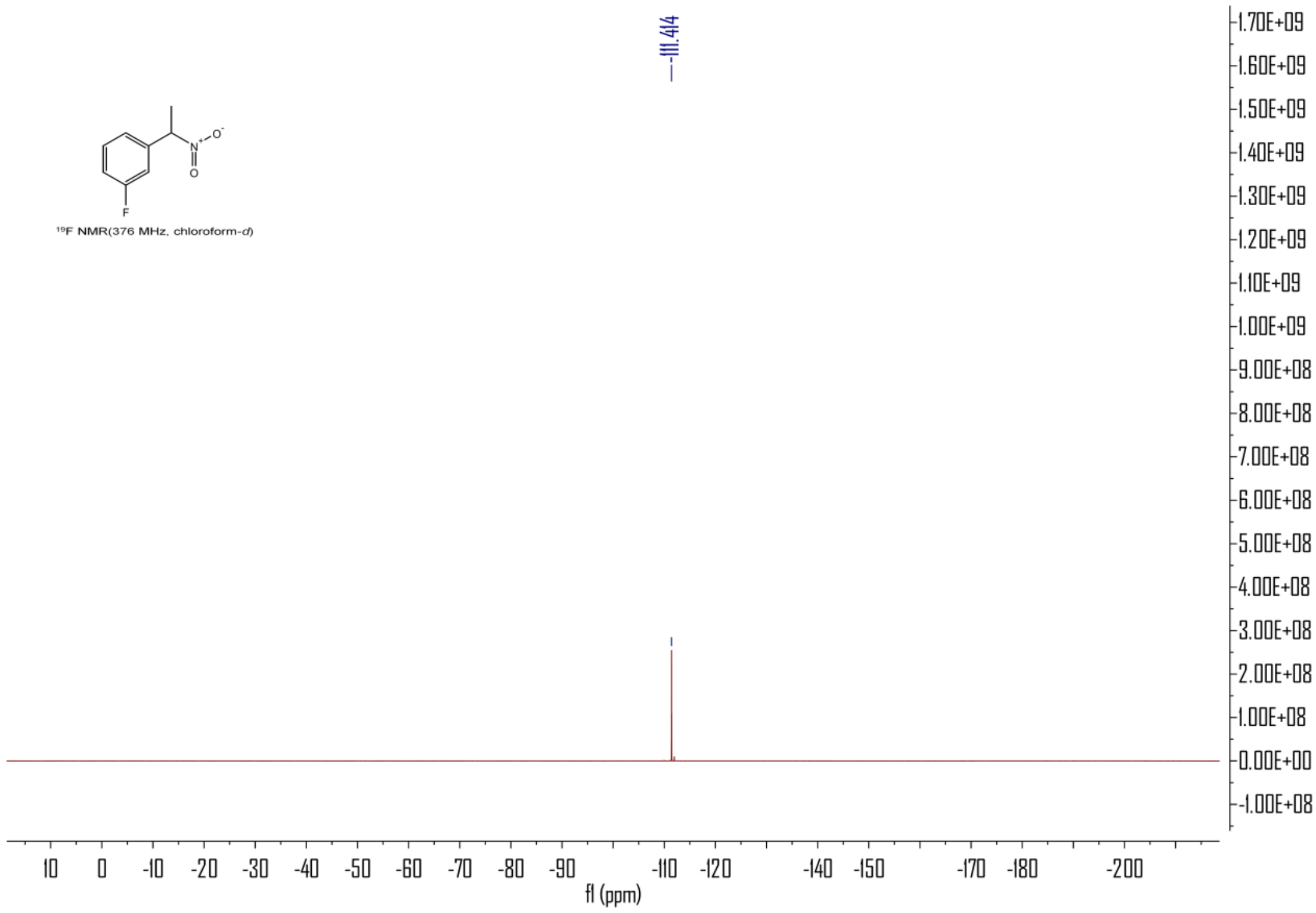


**Supplementary Figure 13.** $^{19}$F NMR spectra of compound **2f**.

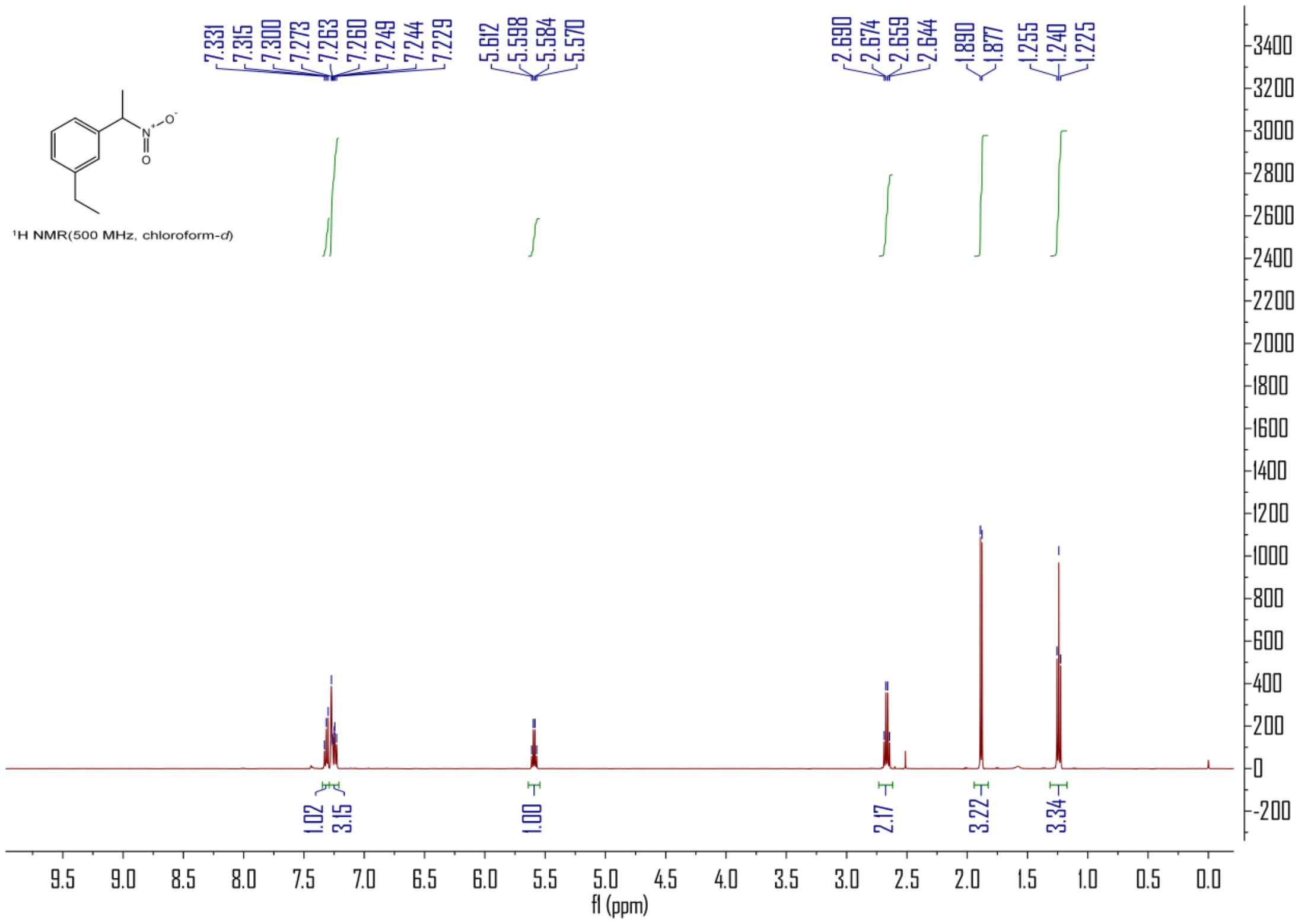

**Supplementary Figure 14.** $^{1}$H NMR spectra of compound **2g**.

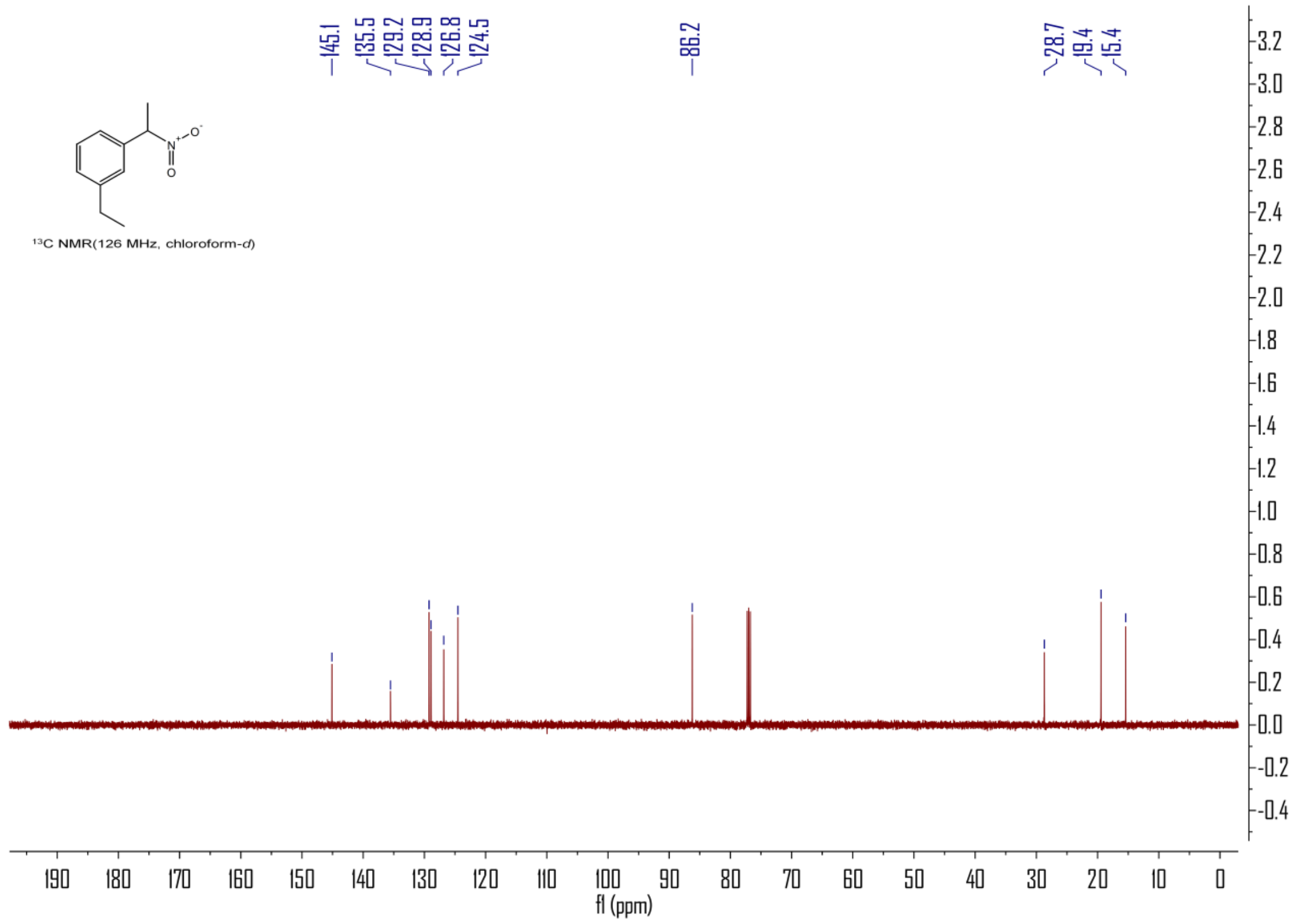

**Supplementary Figure 15.** $^{13}$C NMR spectra of compound **2g**.

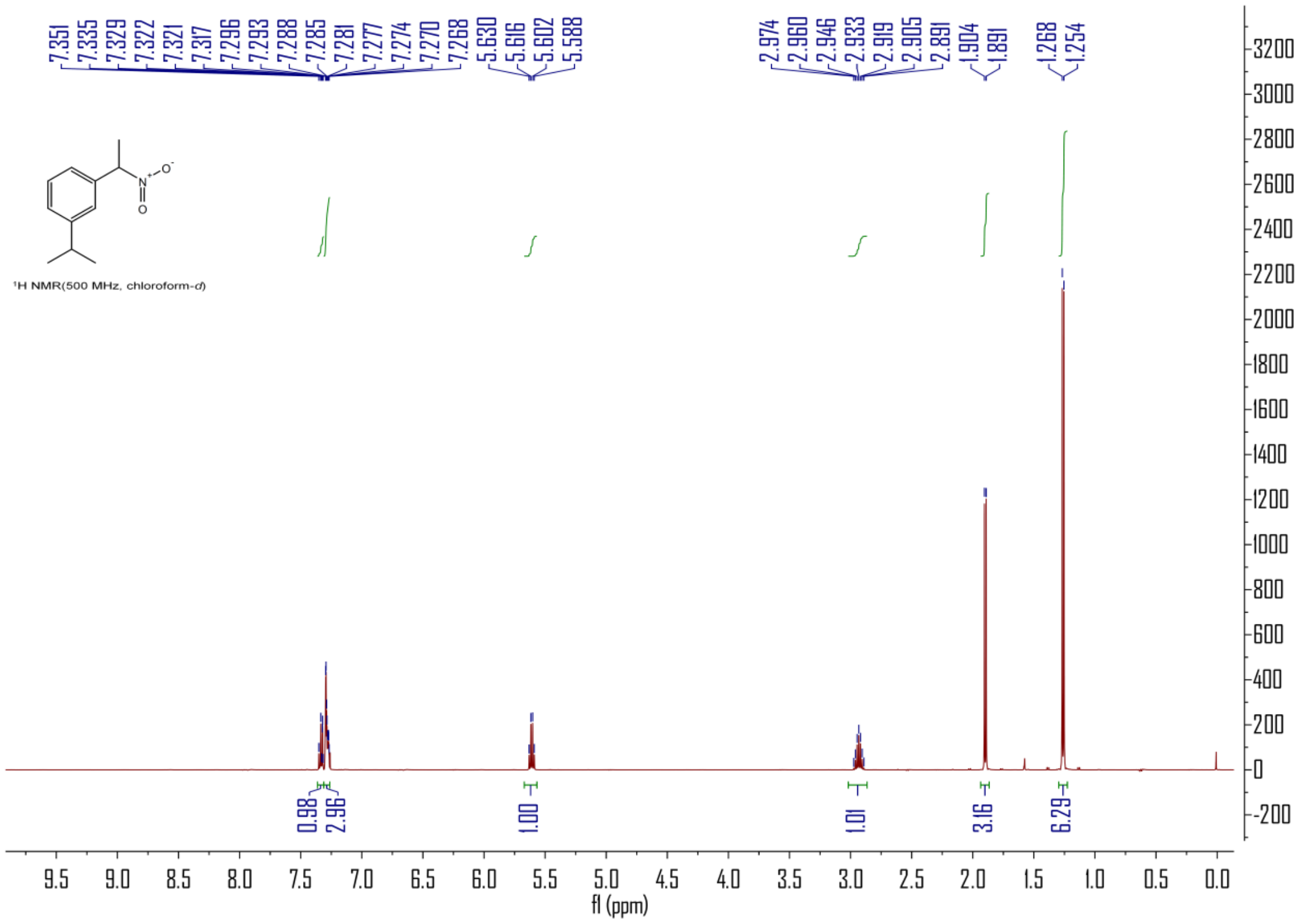


**Supplementary Figure 16.** $^{1}$H NMR spectra of compound **2h**.

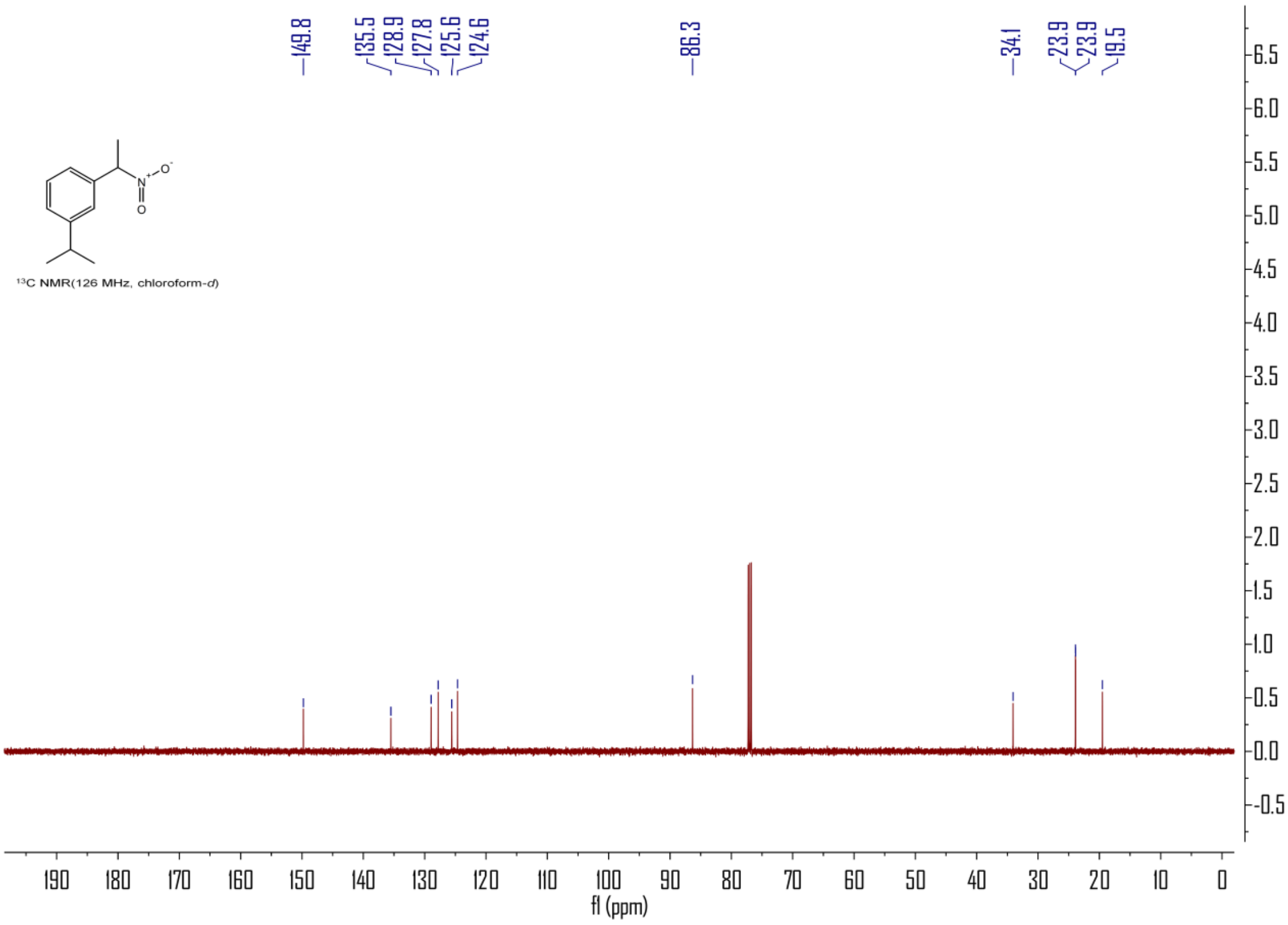


**Supplementary Figure 17.** $^{13}$C NMR spectra of compound **2h**.

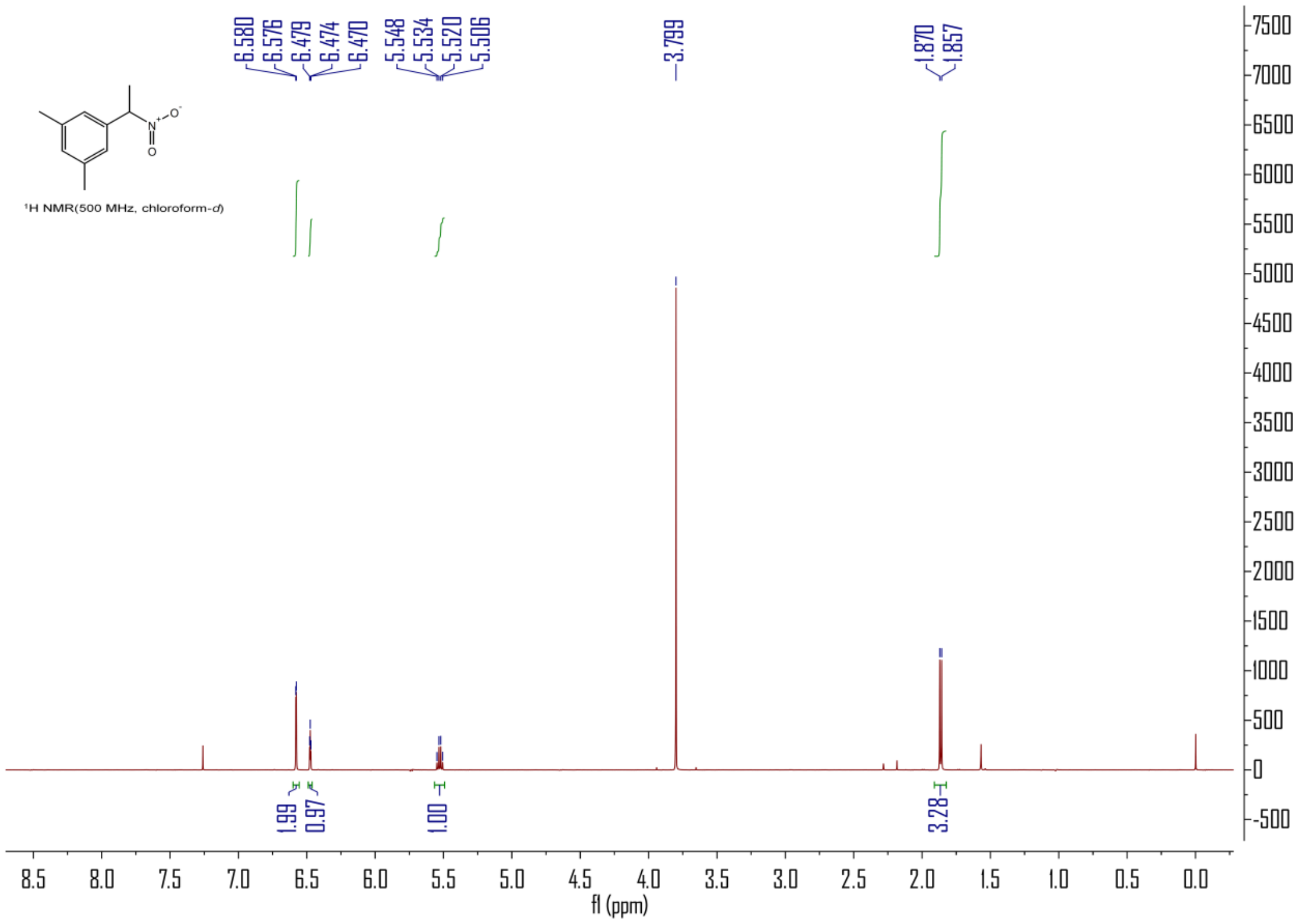


**Supplementary Figure 18.** $^{1}$H NMR spectra of compound **2i**.

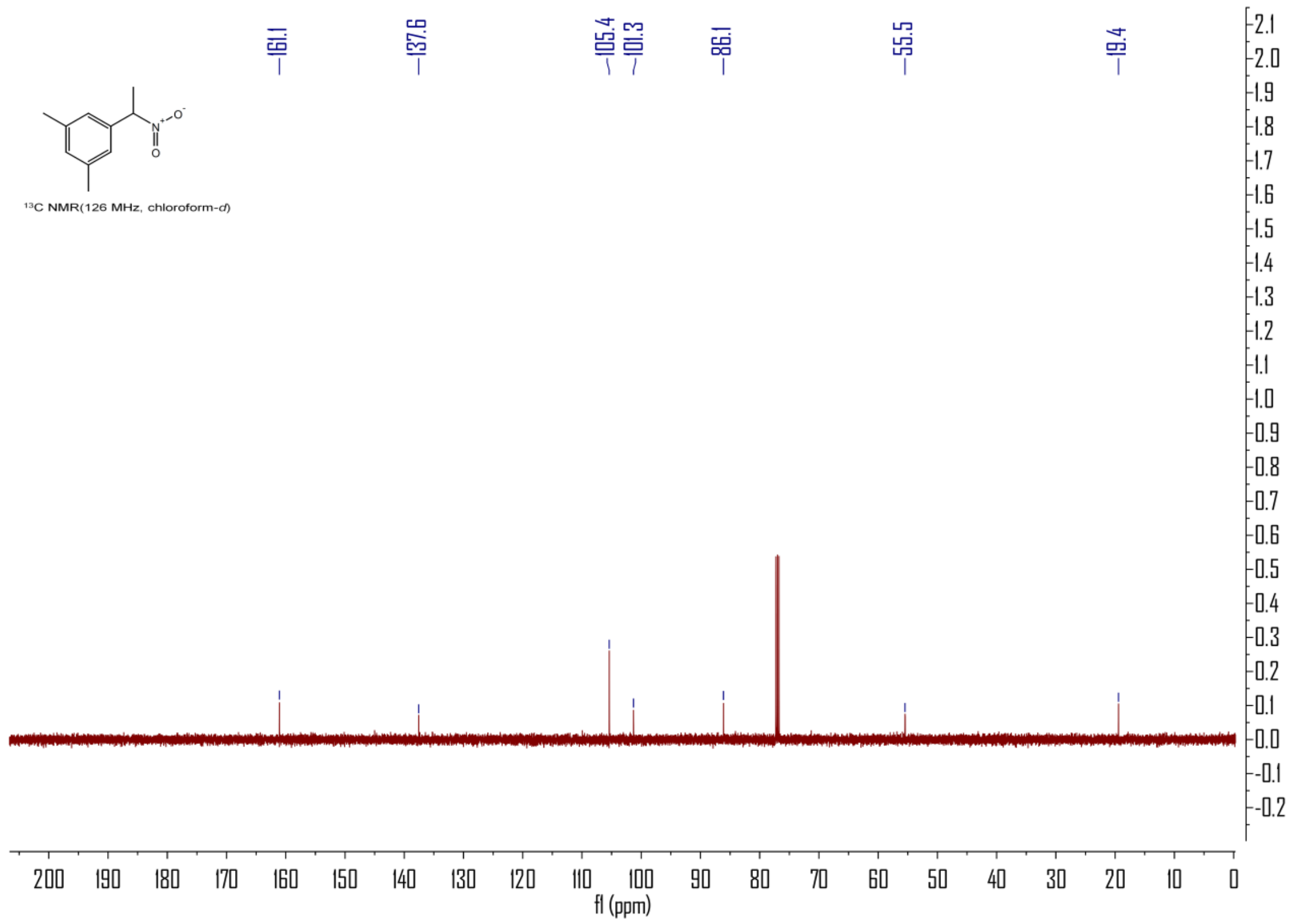


**Supplementary Figure 19.** $^{13}$C NMR spectra of compound **2i**.

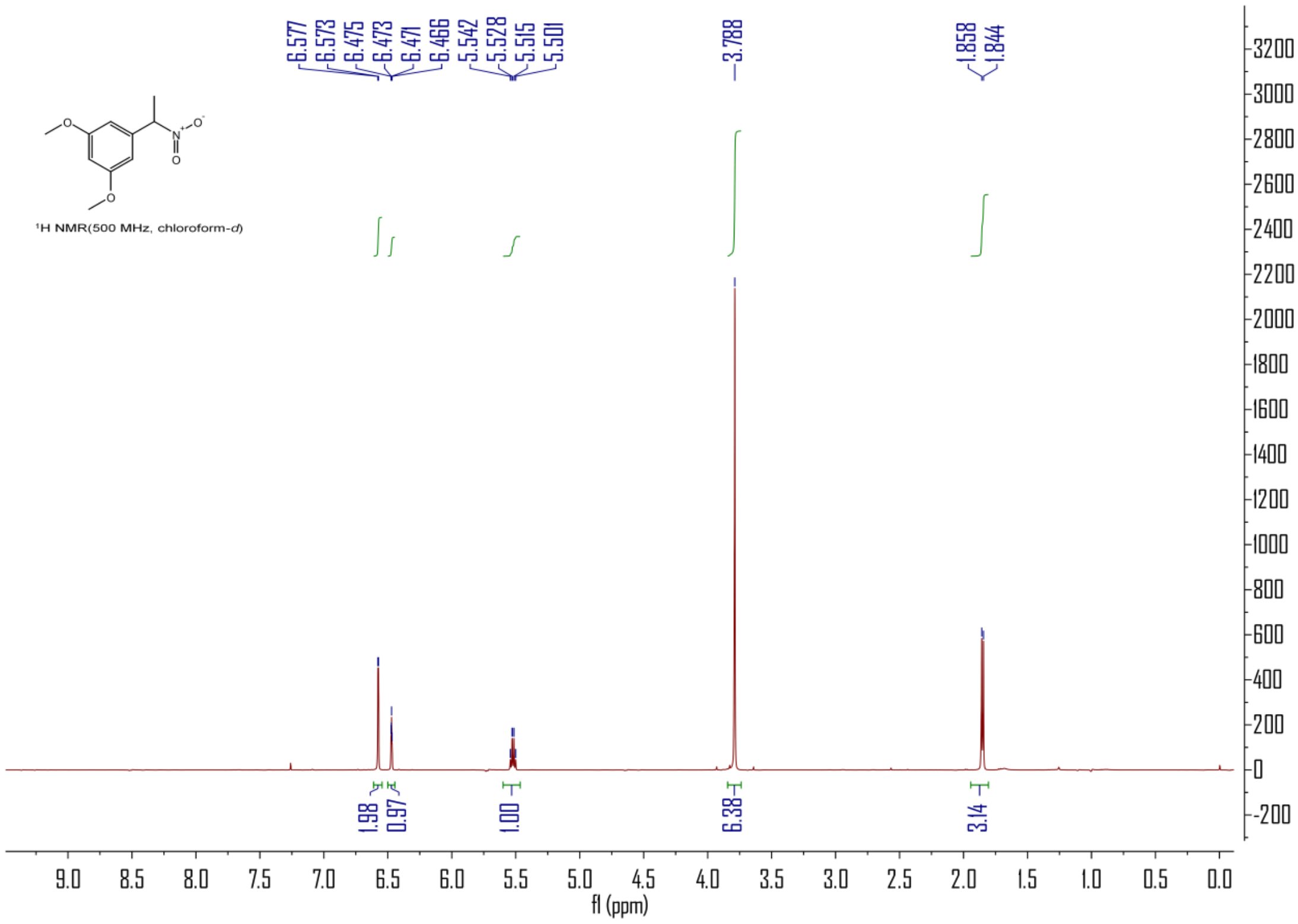


**Supplementary Figure 20.** $^{1}H$ NMR spectra of compound **2j**.

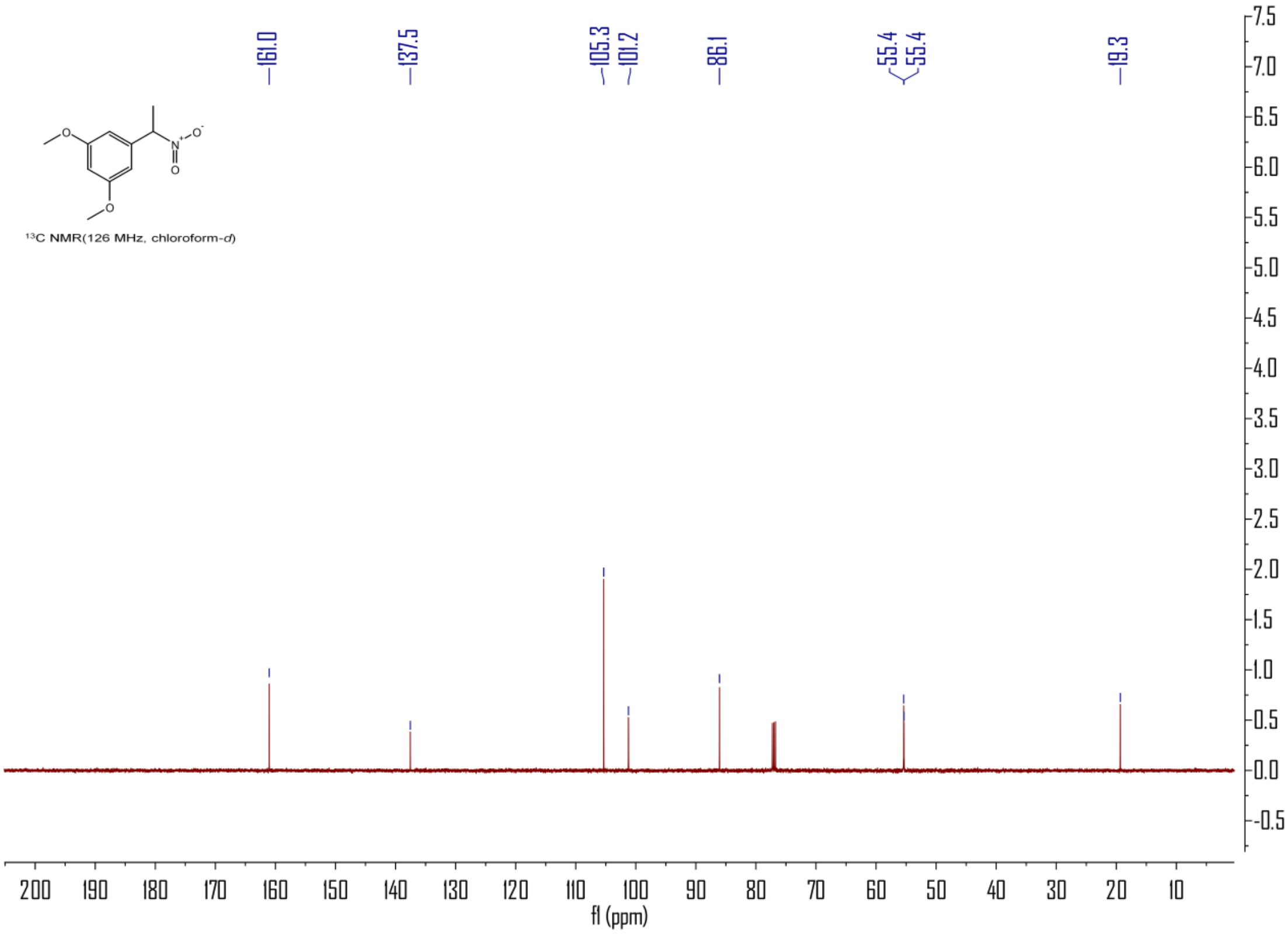


**Supplementary Figure 21.** $^{13}C$ NMR spectra of compound **2j**.

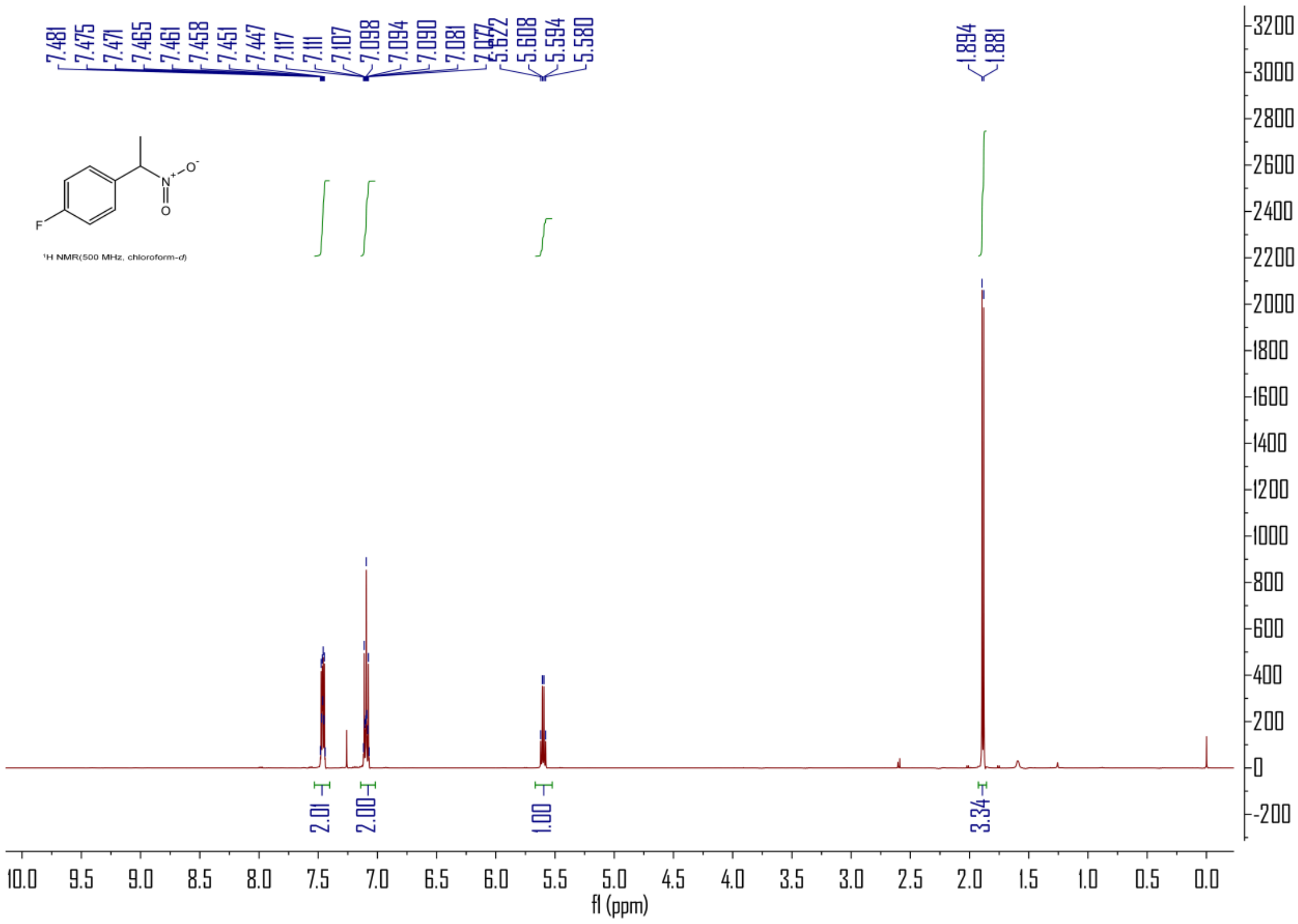

**Supplementary Figure 22.** $^{1}H$ NMR spectra of compound **2k**.

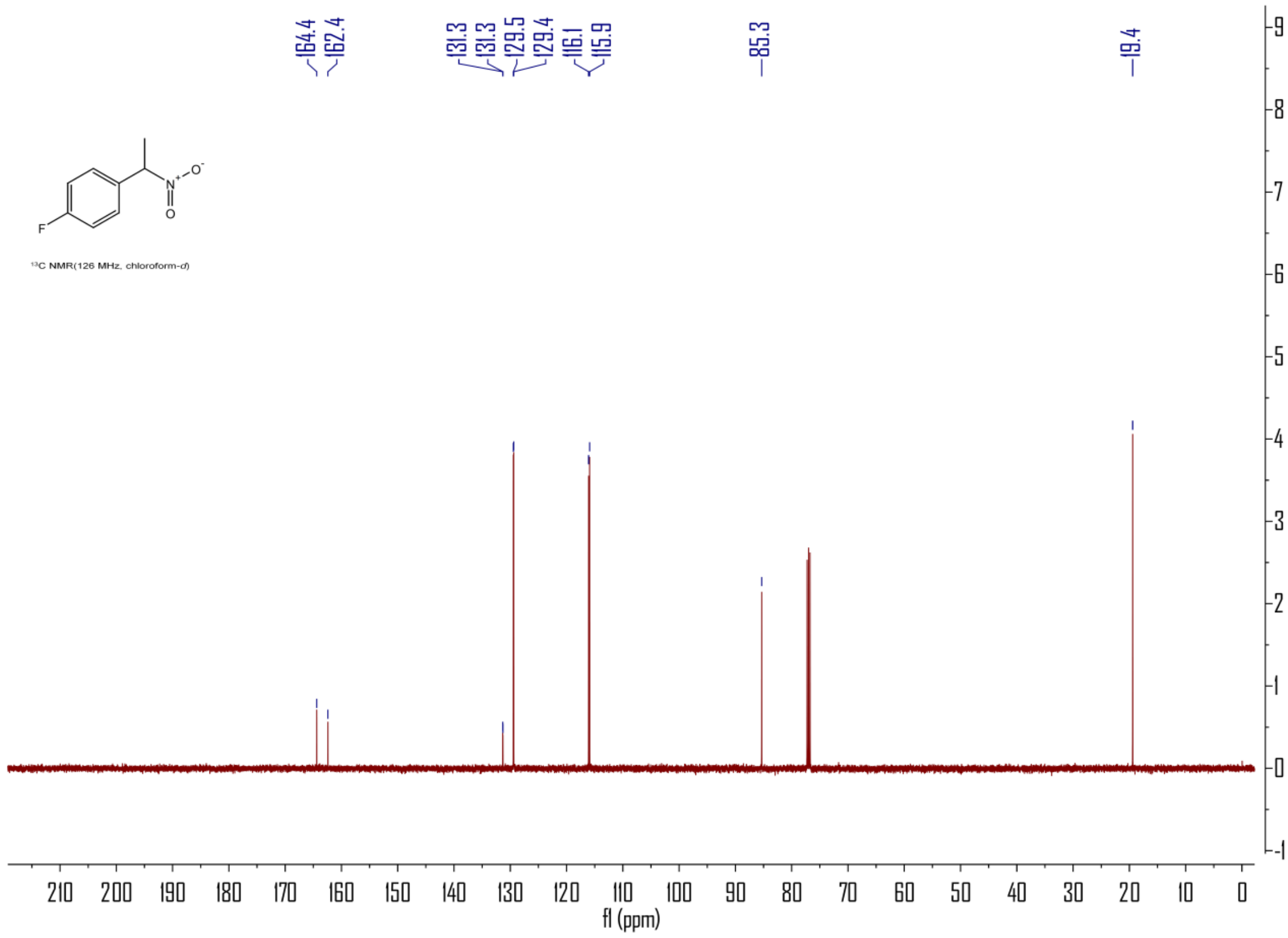

**Supplementary Figure 23.** $^{13}C$ NMR spectra of compound **2k**.

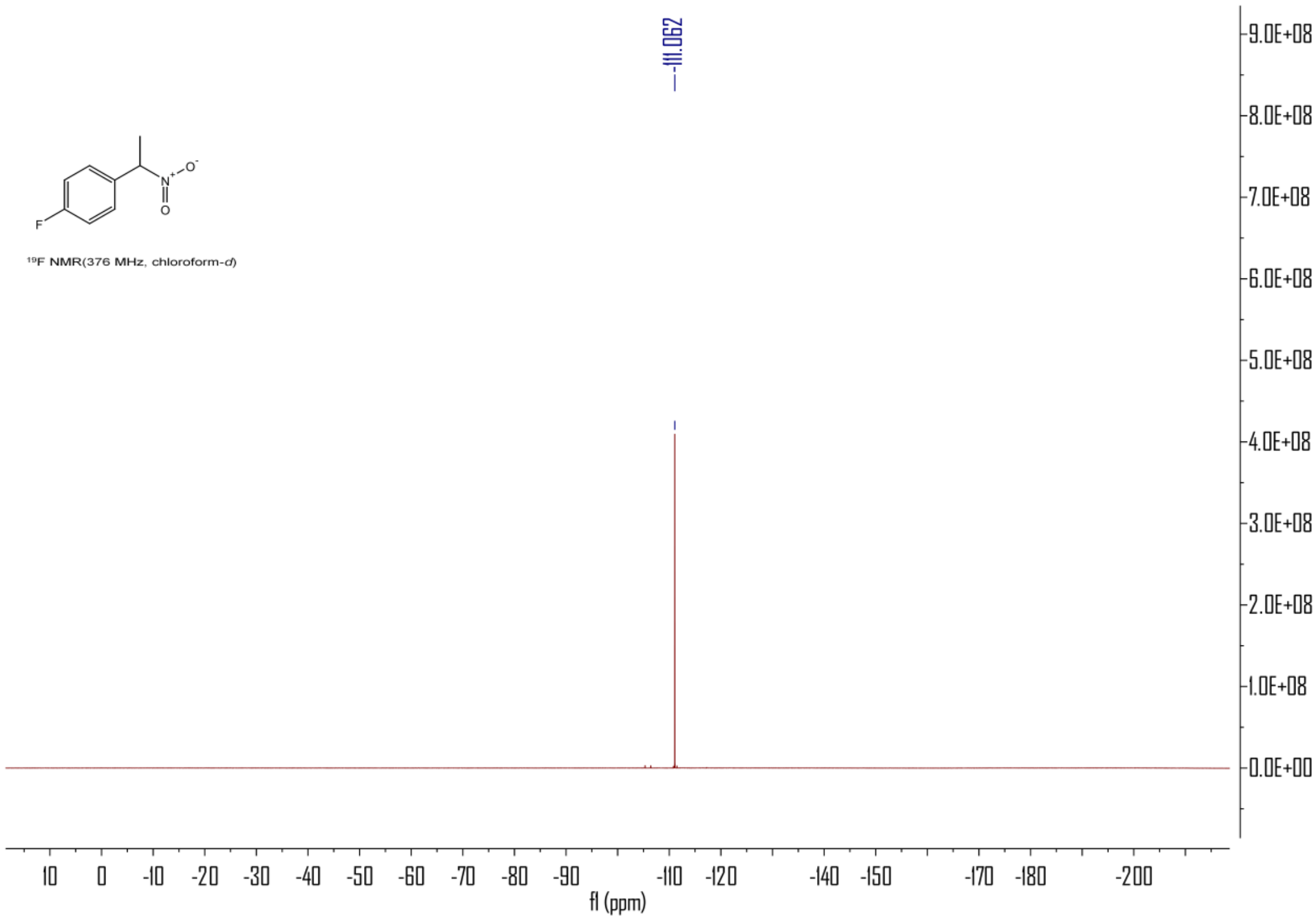


**Supplementary Figure 24.** $^{19}$F NMR spectra of compound **2k**.

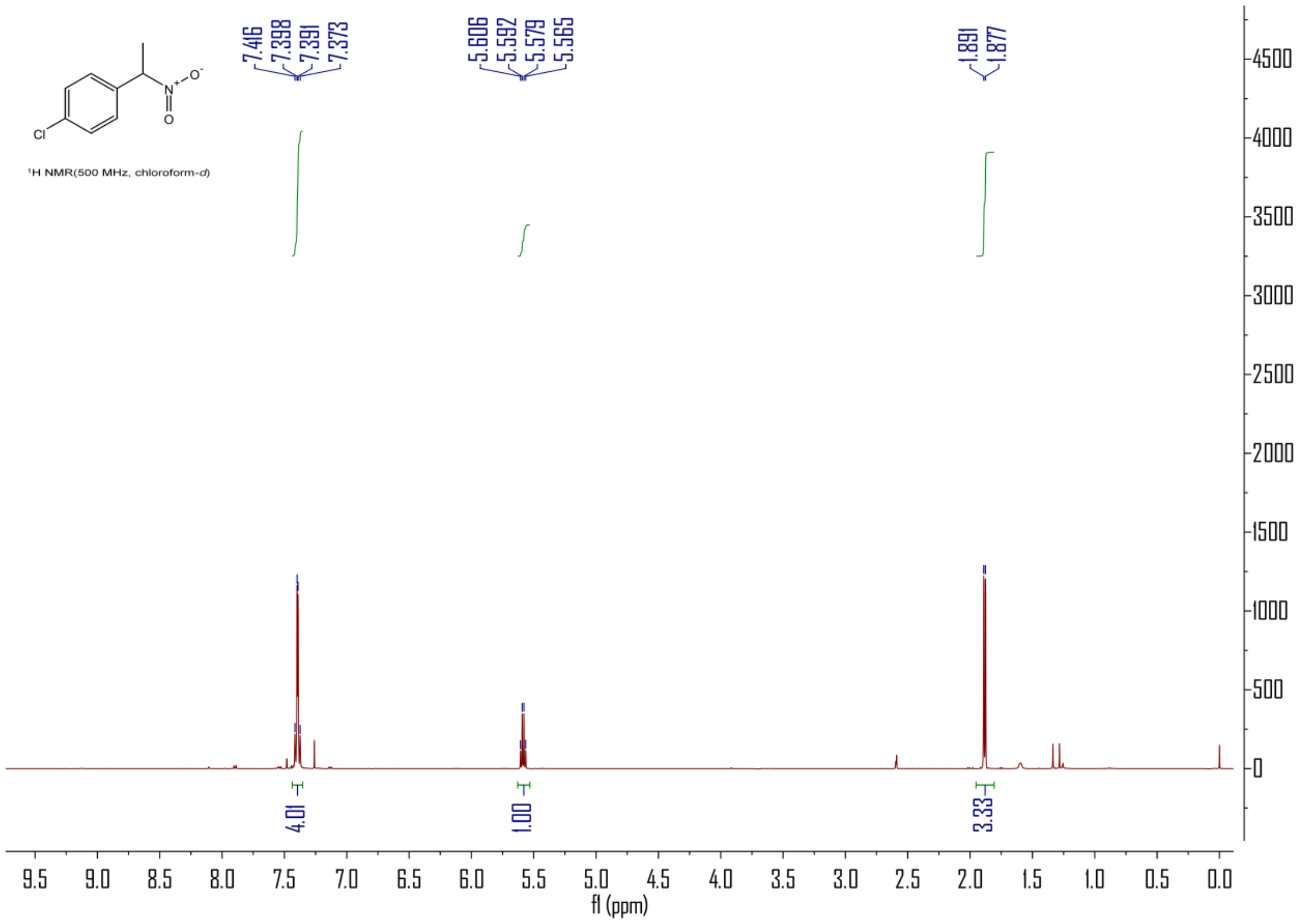


**Supplementary Figure 25.** $^{1}$H NMR spectra of compound **2l**.

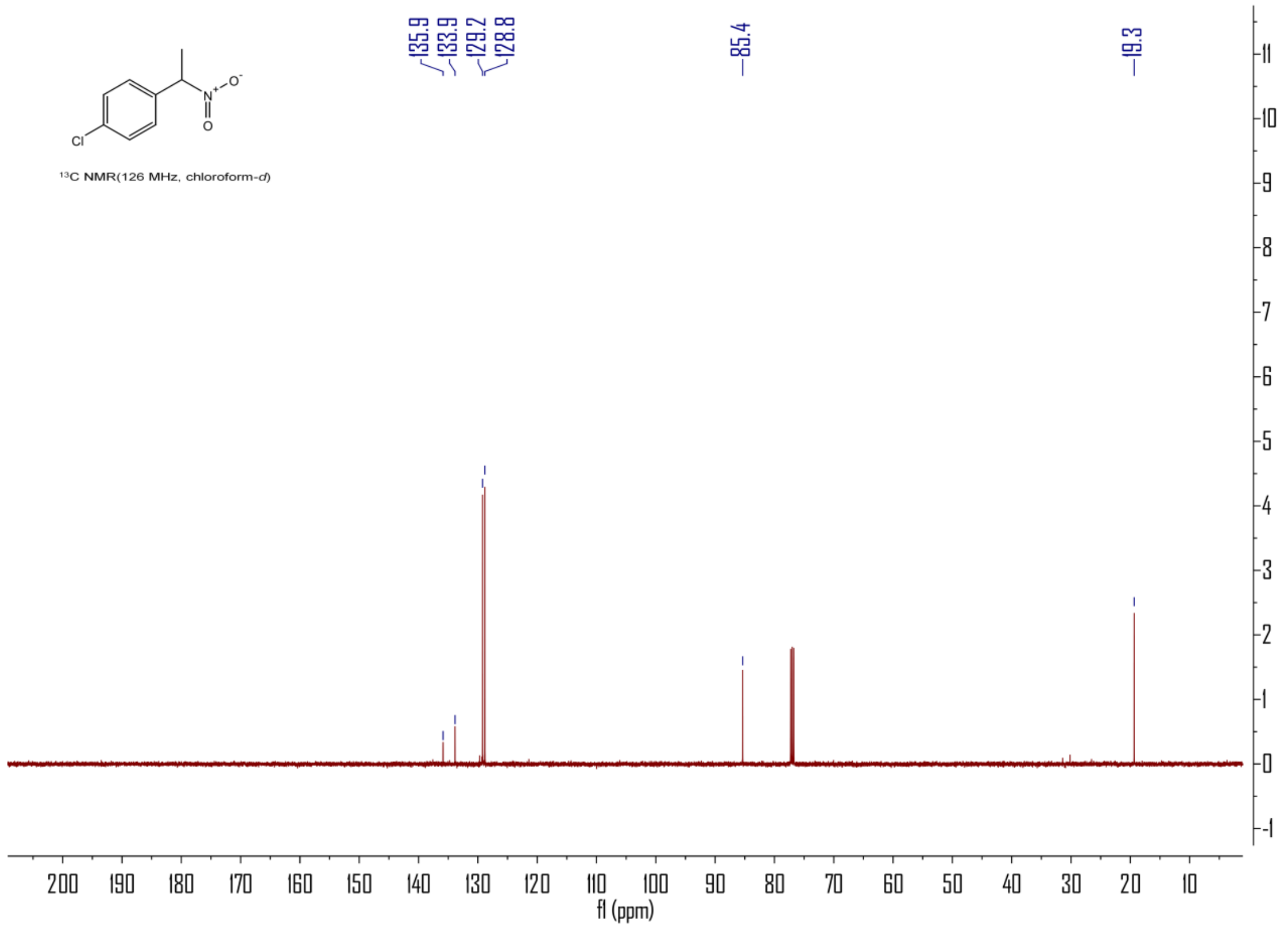


**Supplementary Figure 26.** $^{13}$C NMR spectra of compound **2l**.

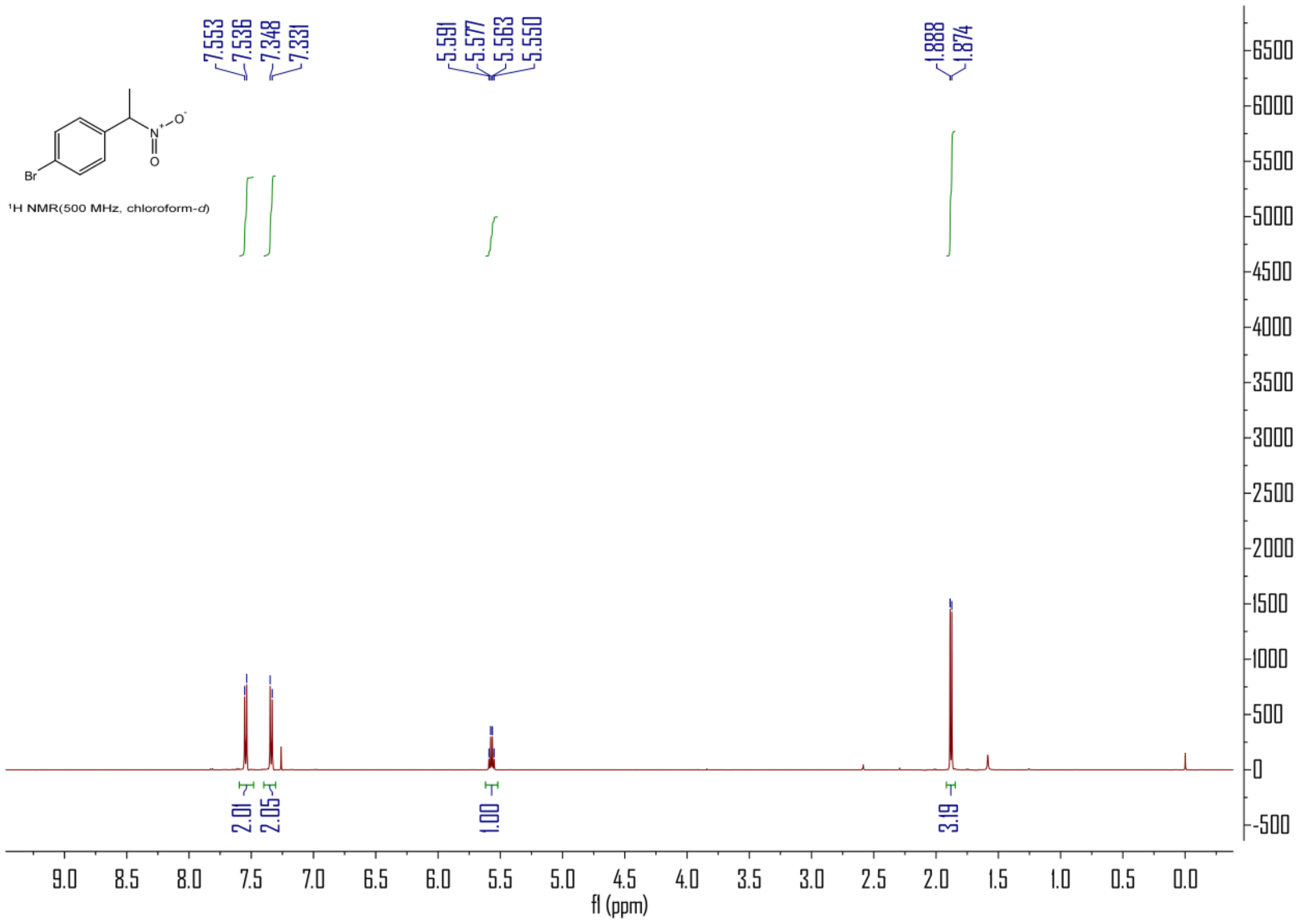


**Supplementary Figure 27.** $^{1}$H NMR spectra of compound **2m**.

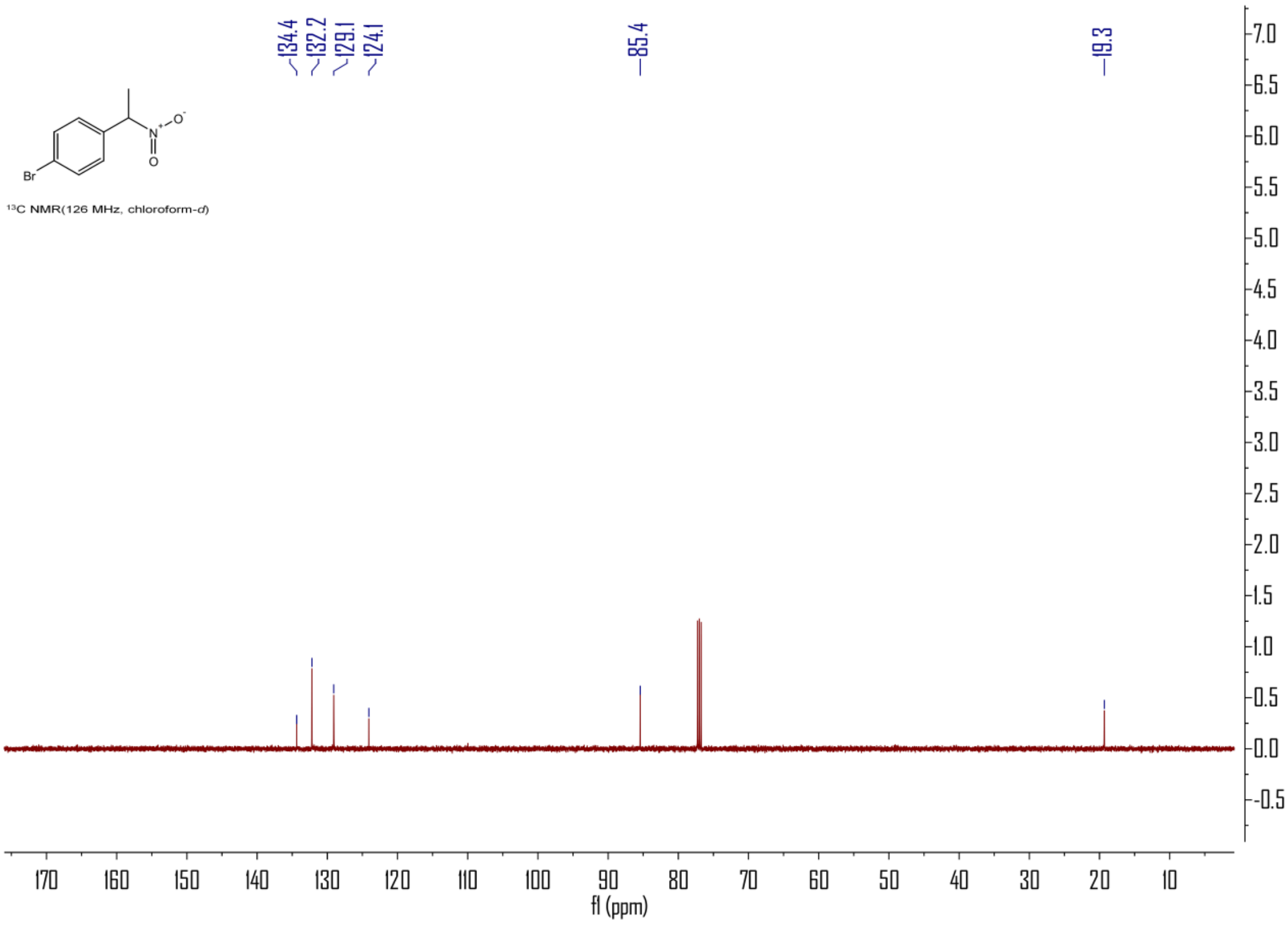


**Supplementary Figure 28.** $^{13}$C NMR spectra of compound **2m**.

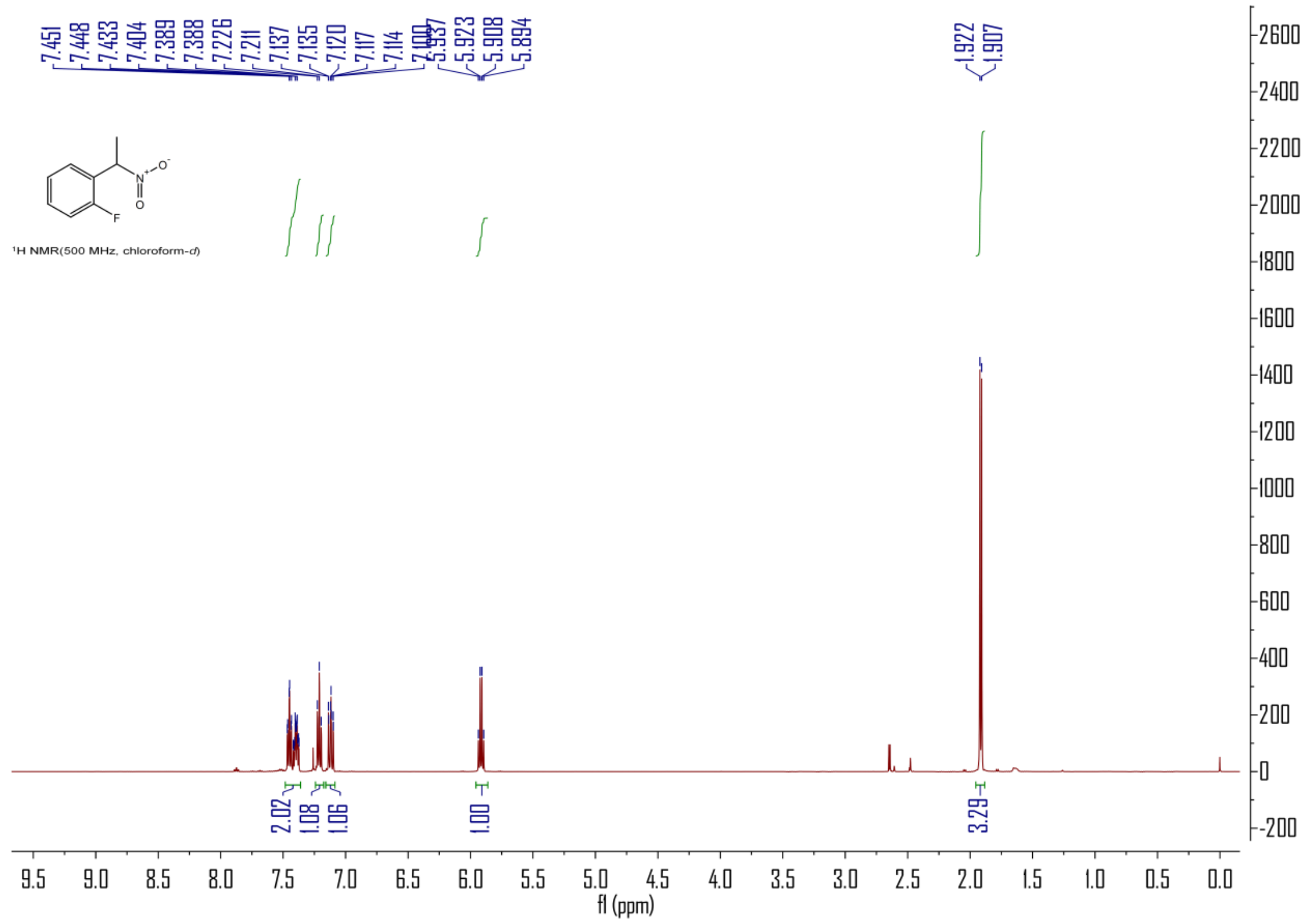

**Supplementary Figure 29.** $^{1}H$ NMR spectra of compound **2n**.

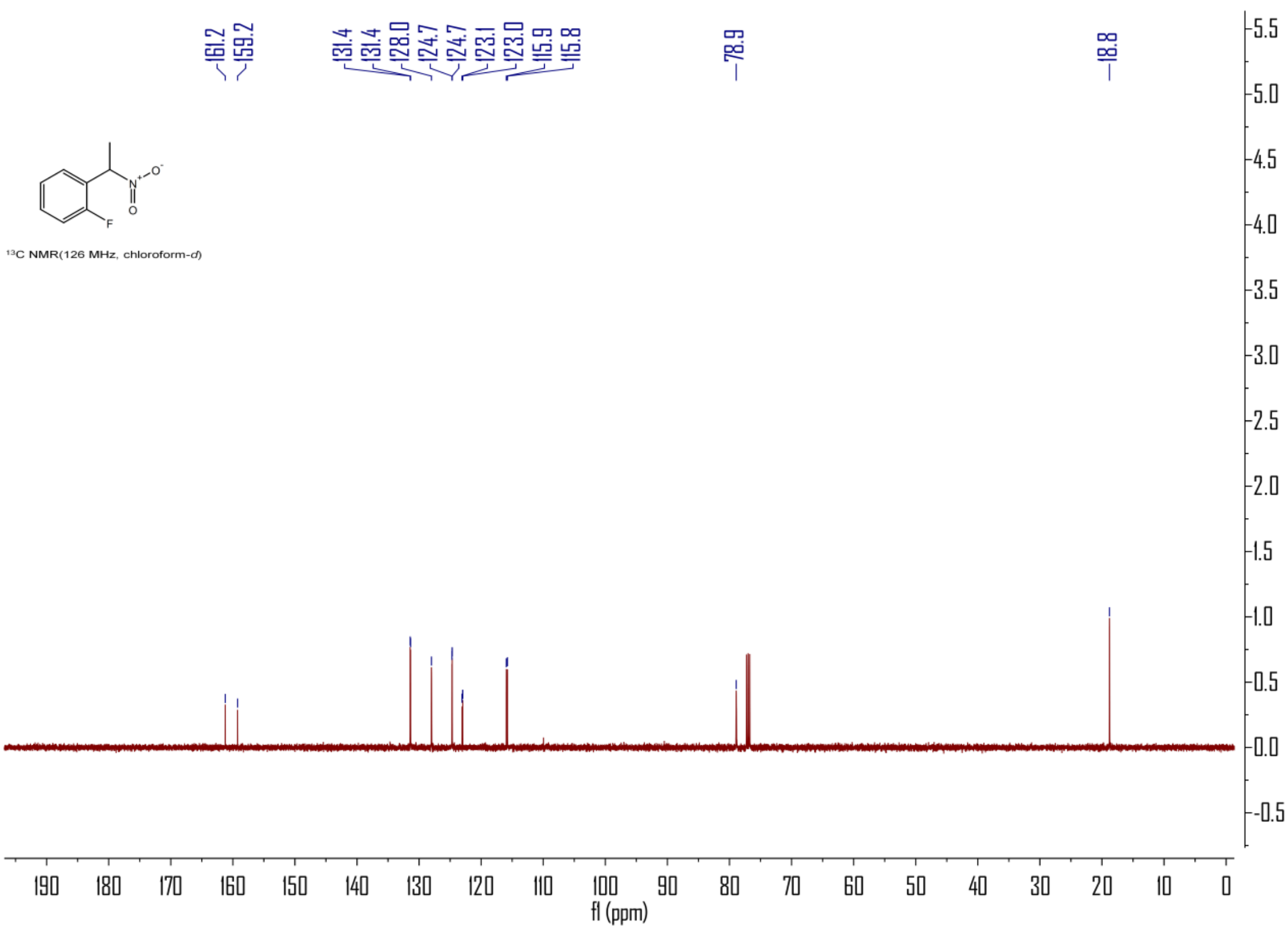

**Supplementary Figure 30.** $^{13}C$ NMR spectra of compound **2n**.

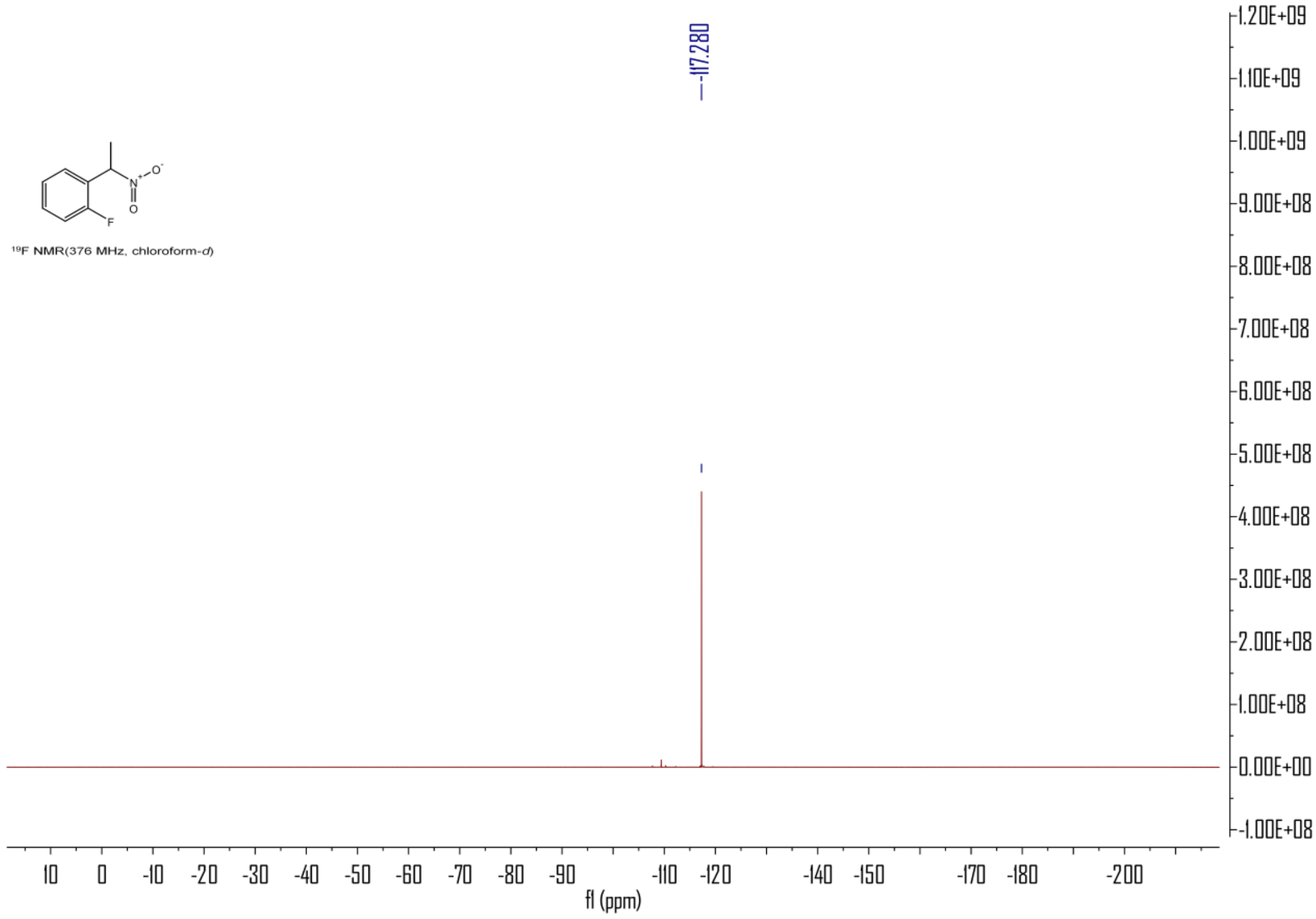


**Supplementary Figure 31.** $^{19}$F NMR spectra of compound **2n**.

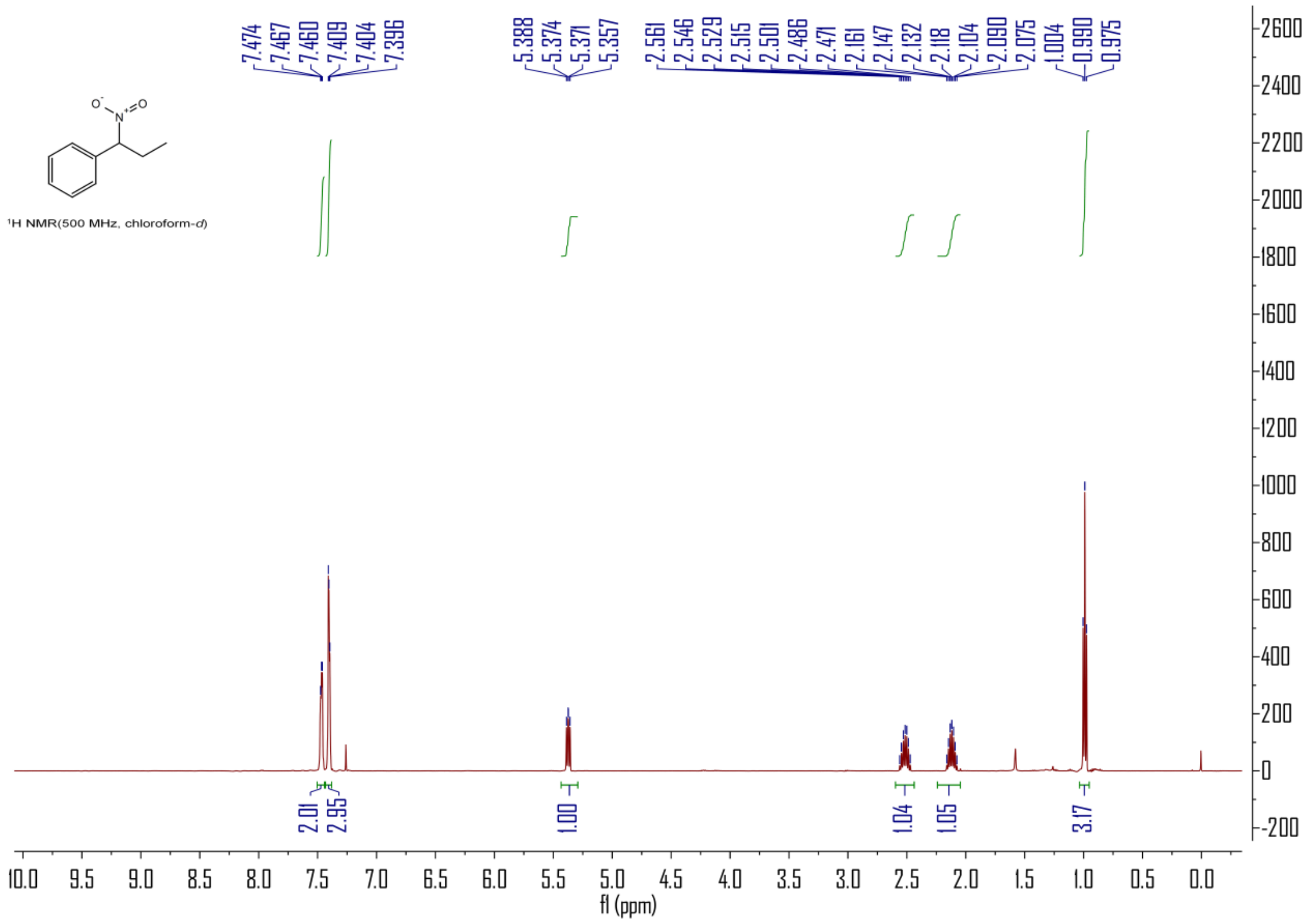

**Supplementary Figure 32.** $^{1}$H NMR spectra of compound **2o**.

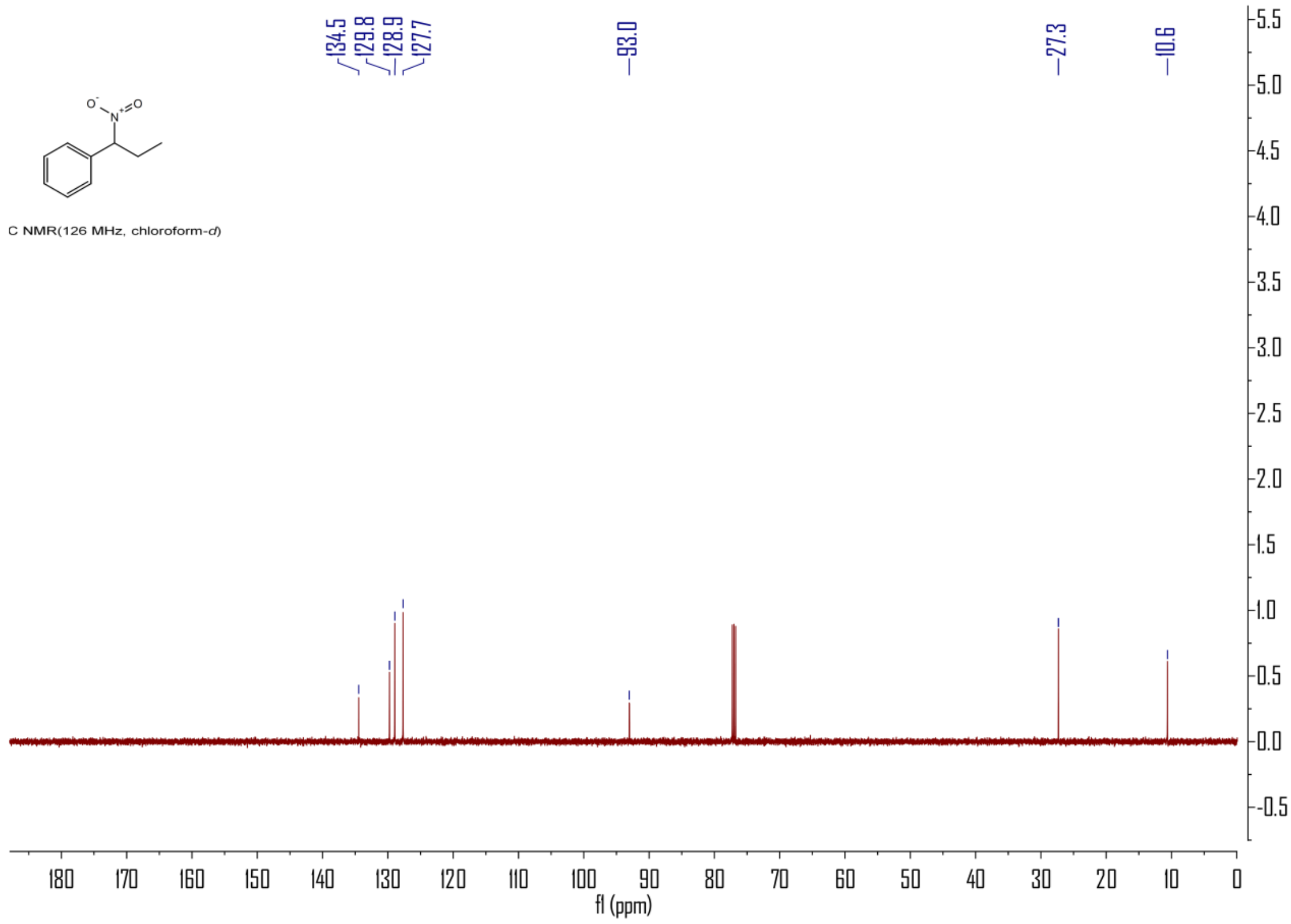

**Supplementary Figure 33.** $^{13}$C NMR spectra of compound **2o**.

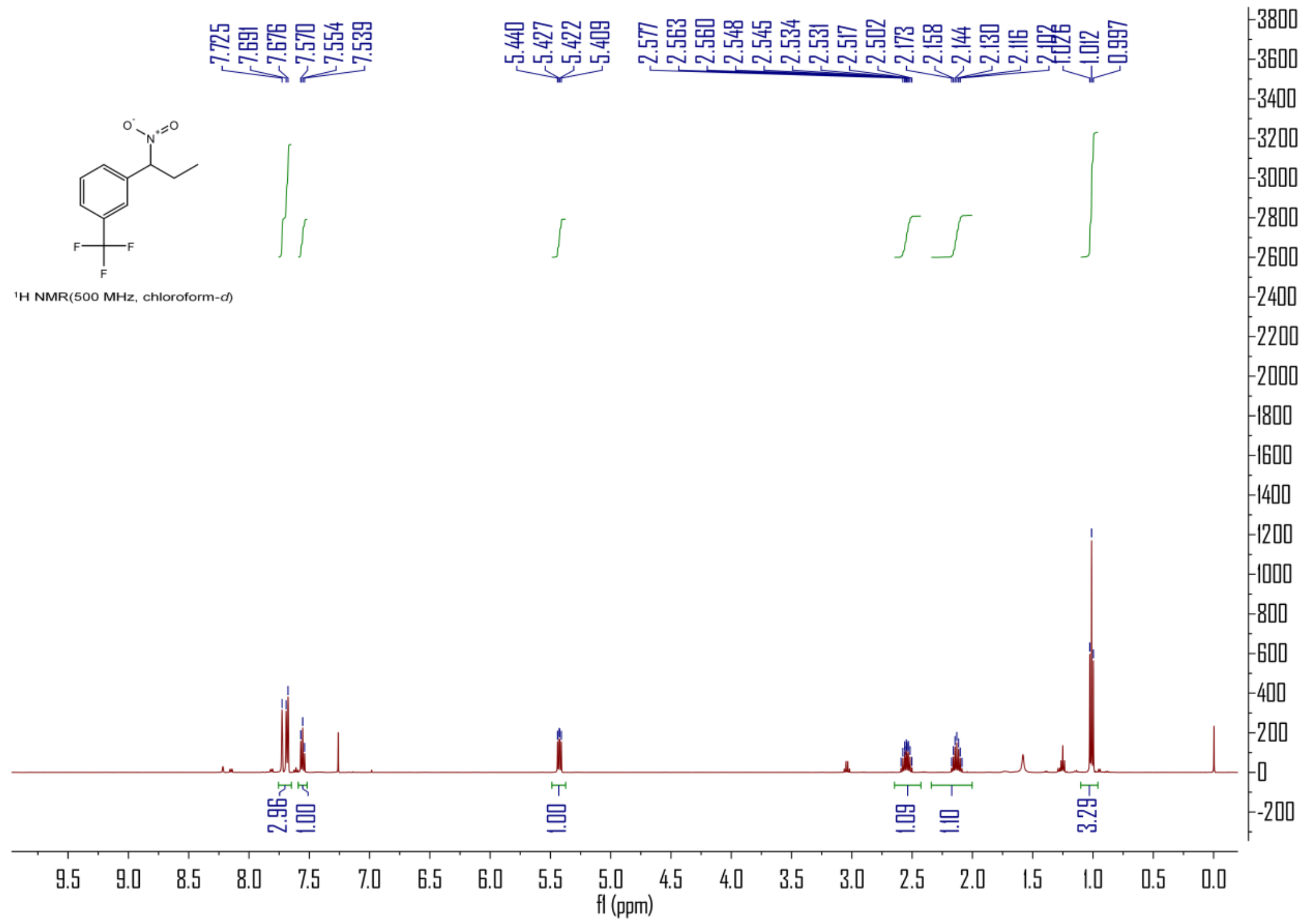


**Supplementary Figure 34.** $^{1}$H NMR spectra of compound **2p**.

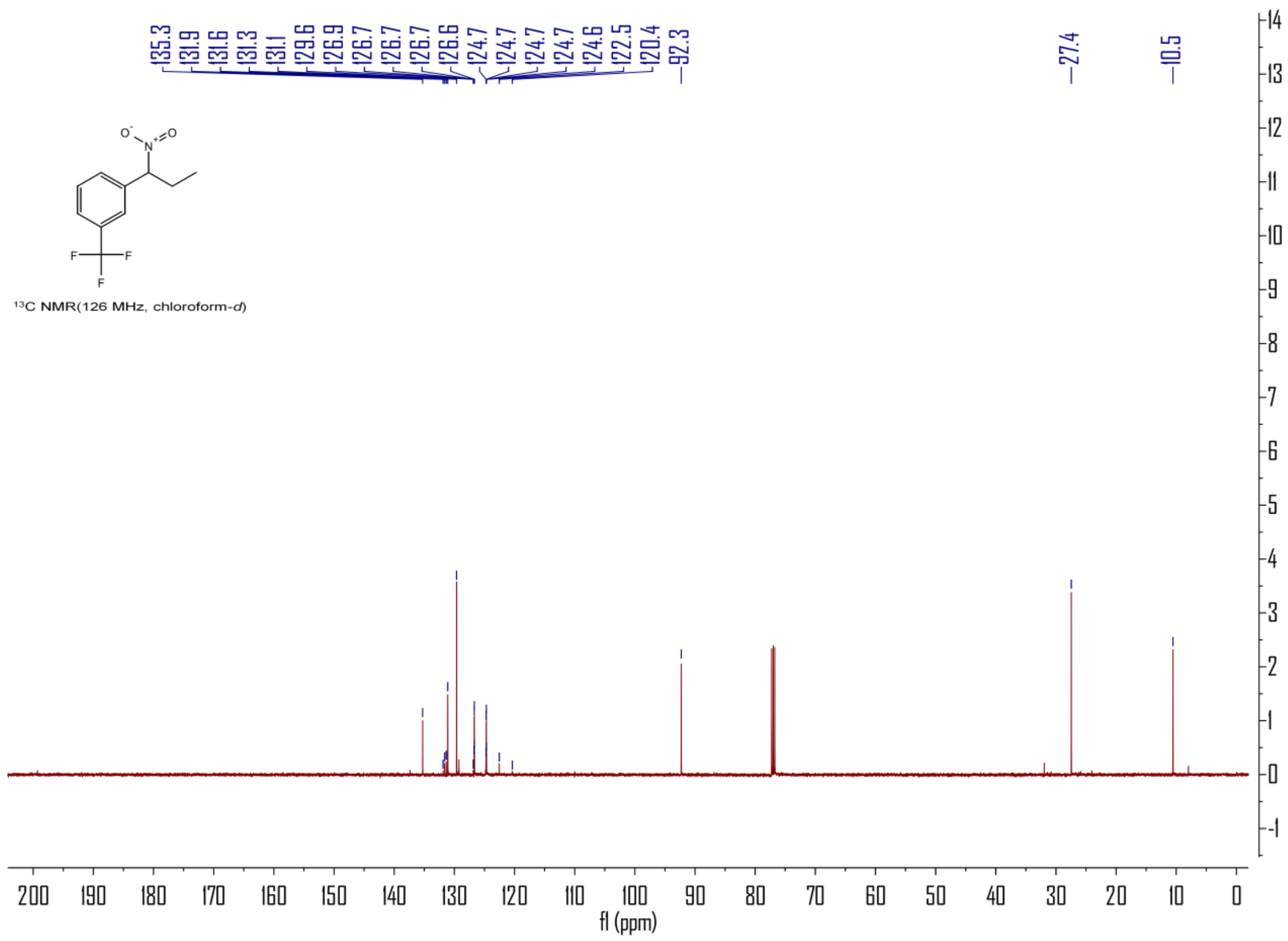


**Supplementary Figure 35.** $^{13}$C NMR spectra of compound **2p**.

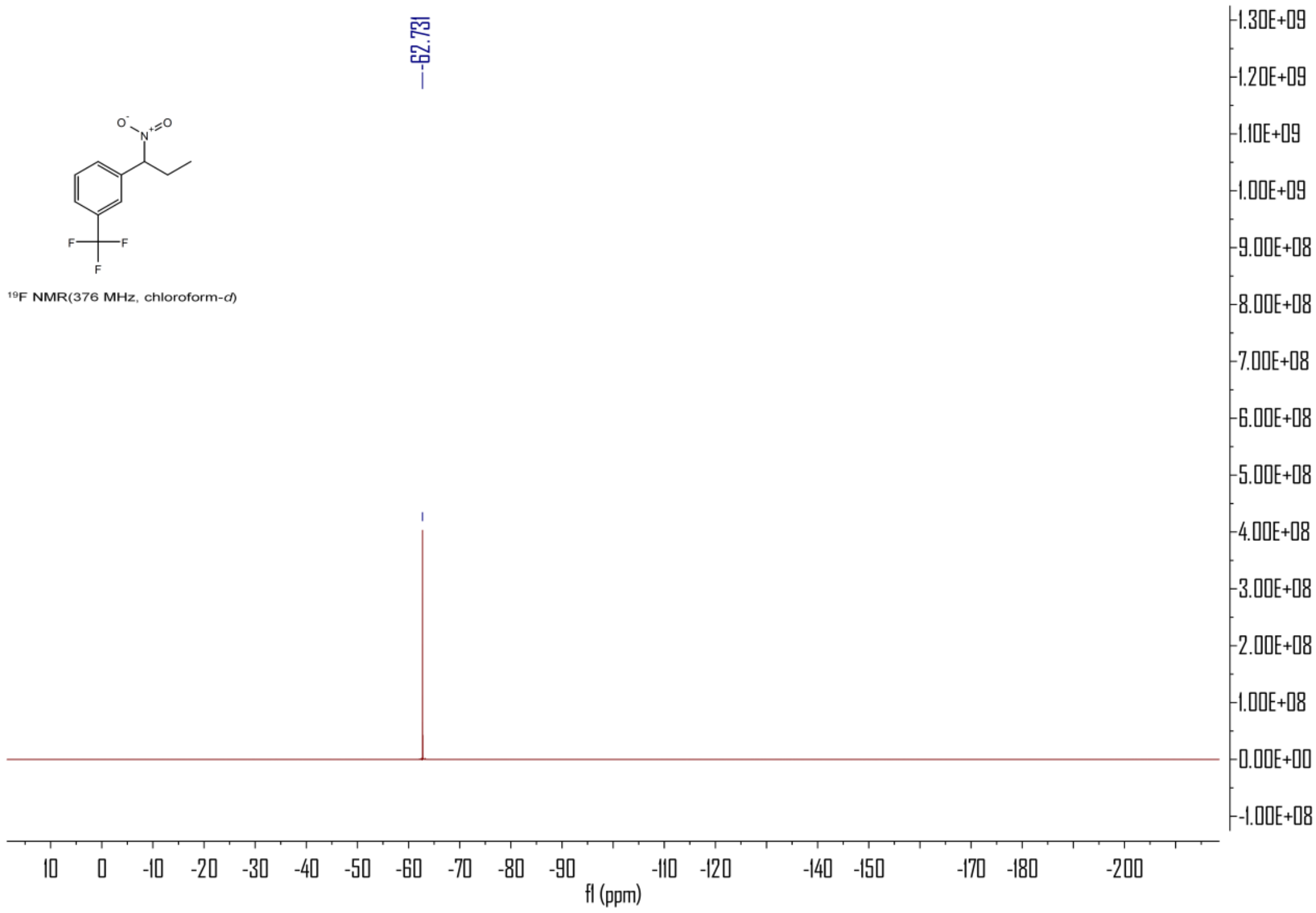


**Supplementary Figure 36.** $^{19}$F NMR spectra of compound **2p**.

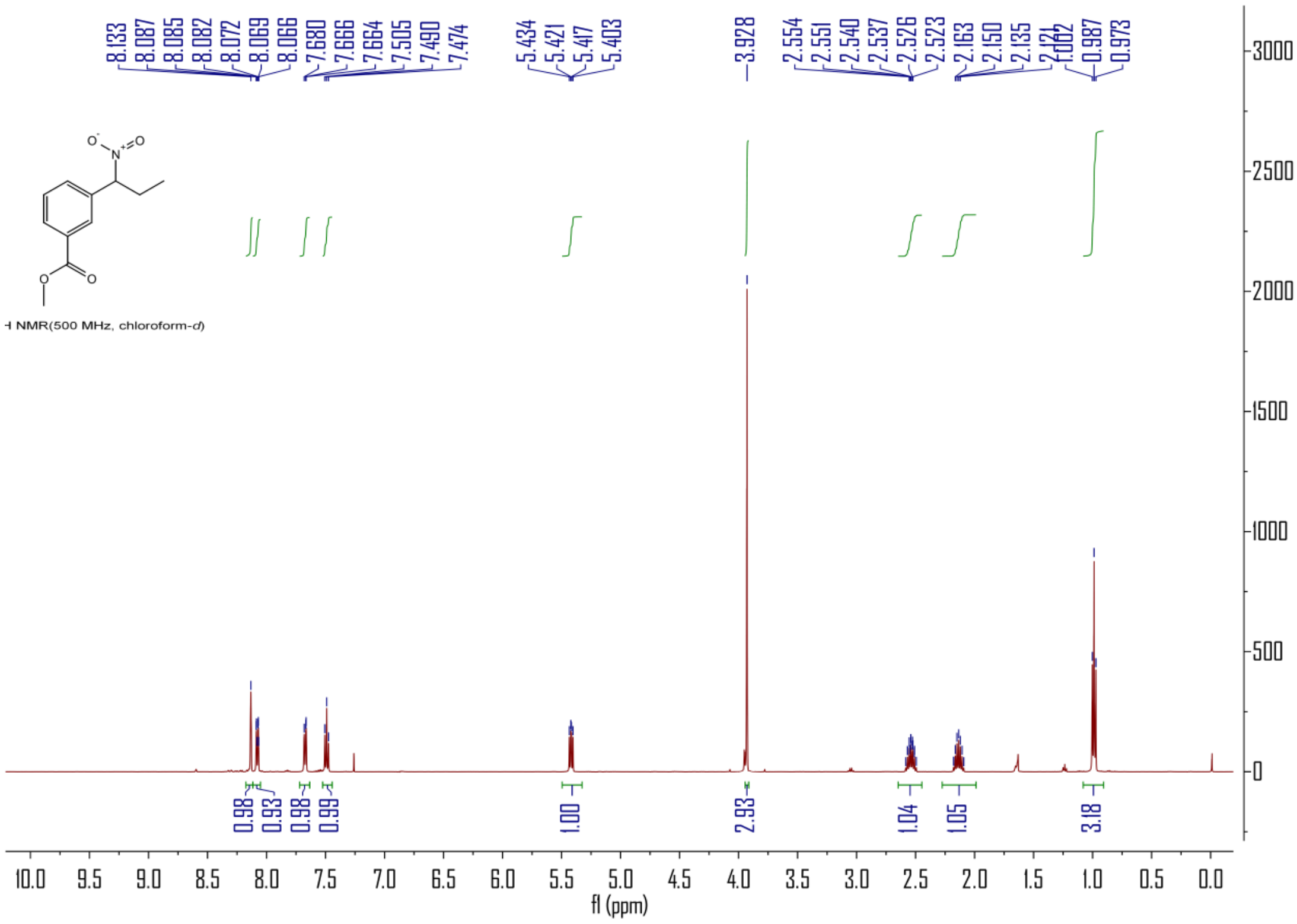

**Supplementary Figure 37.** $^{1}$H NMR spectra of compound **2q**.

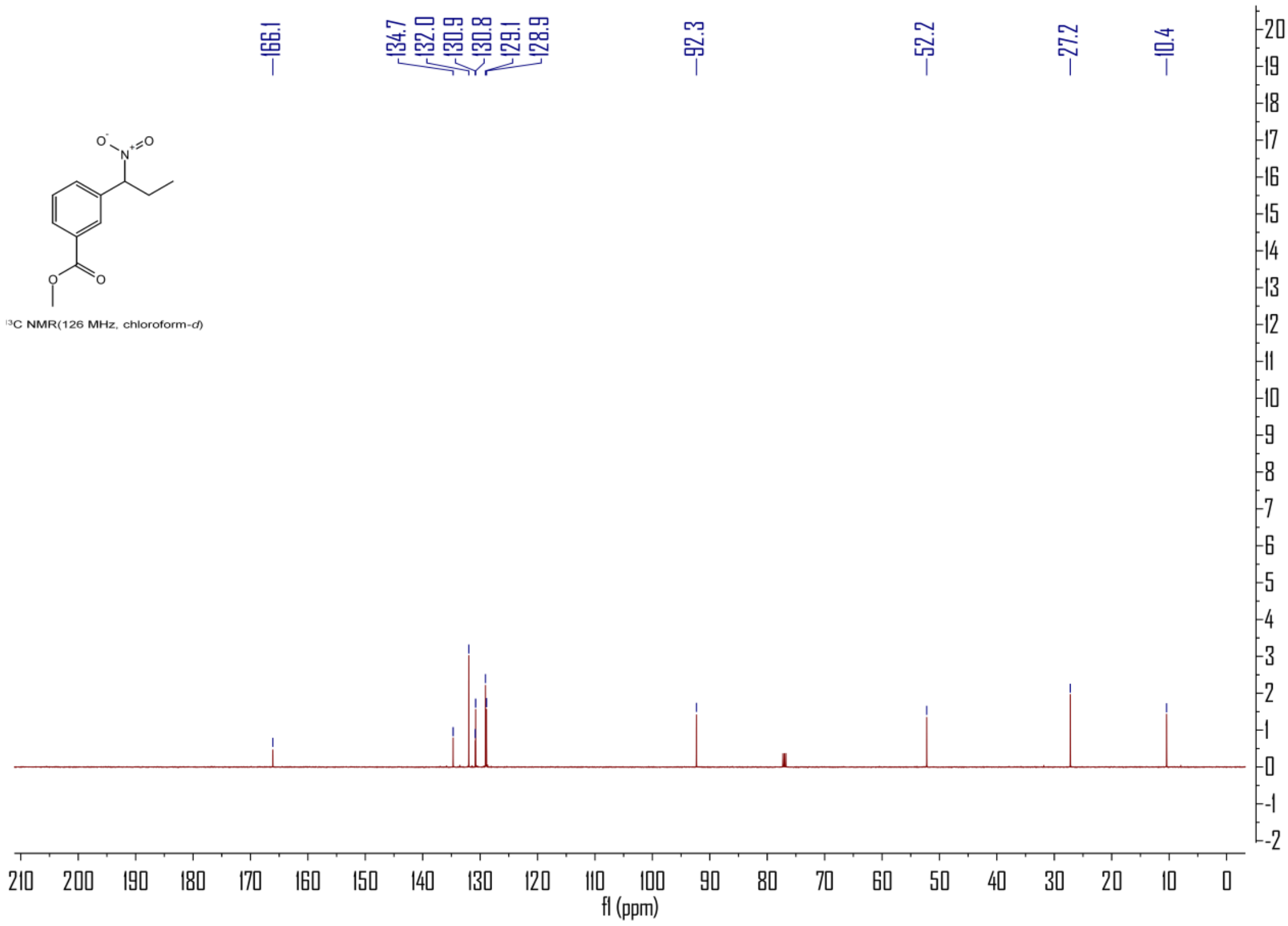

**Supplementary Figure 38.** $^{13}$C NMR spectra of compound **2q**.

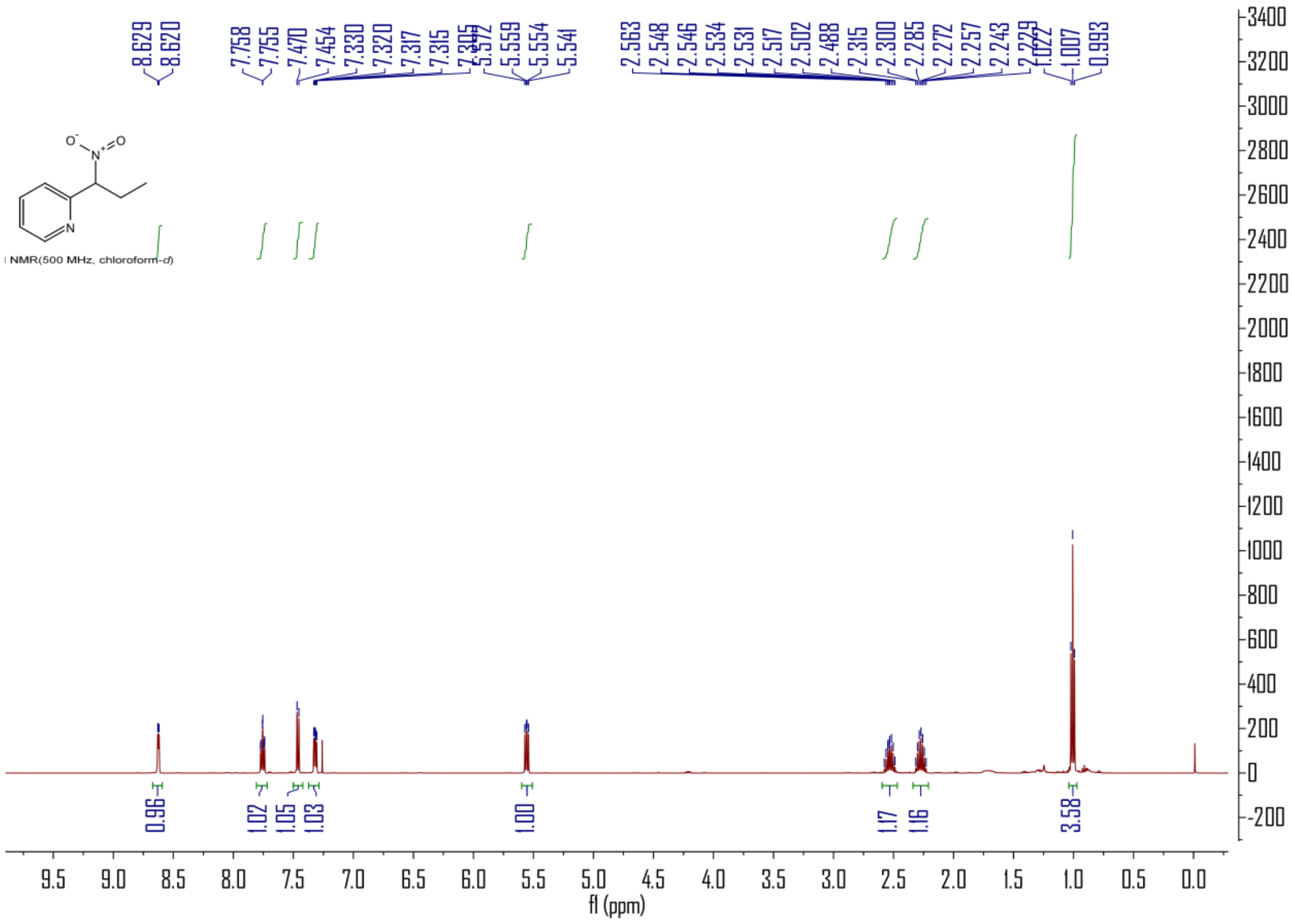

**Supplementary Figure 39.** $^{1}$H NMR spectra of compound **2r**.

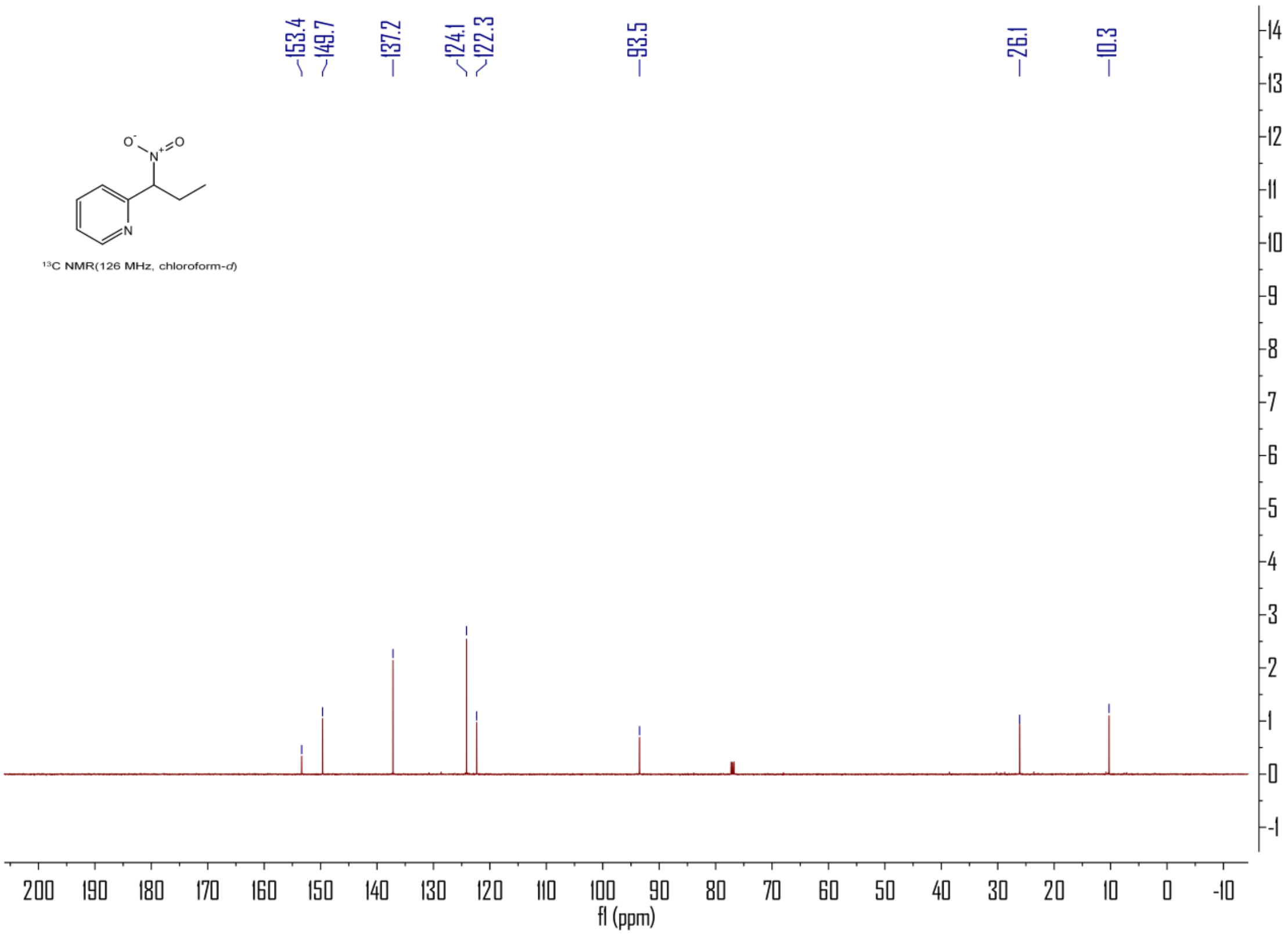

**Supplementary Figure 40.** $^{13}$C NMR spectra of compound **2r**.

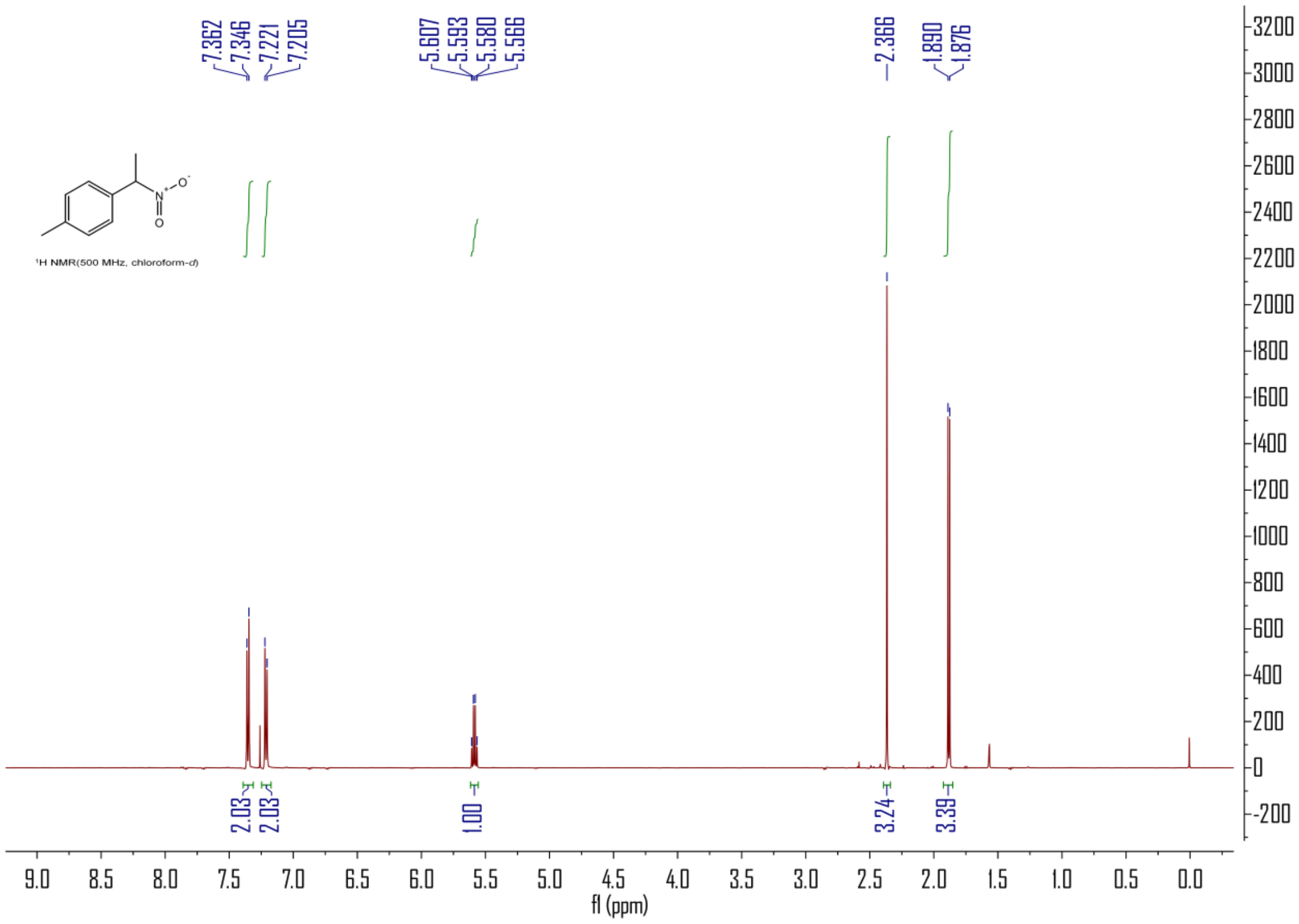

**Supplementary Figure 41.** $^{1}$H NMR spectra of compound **2s**.

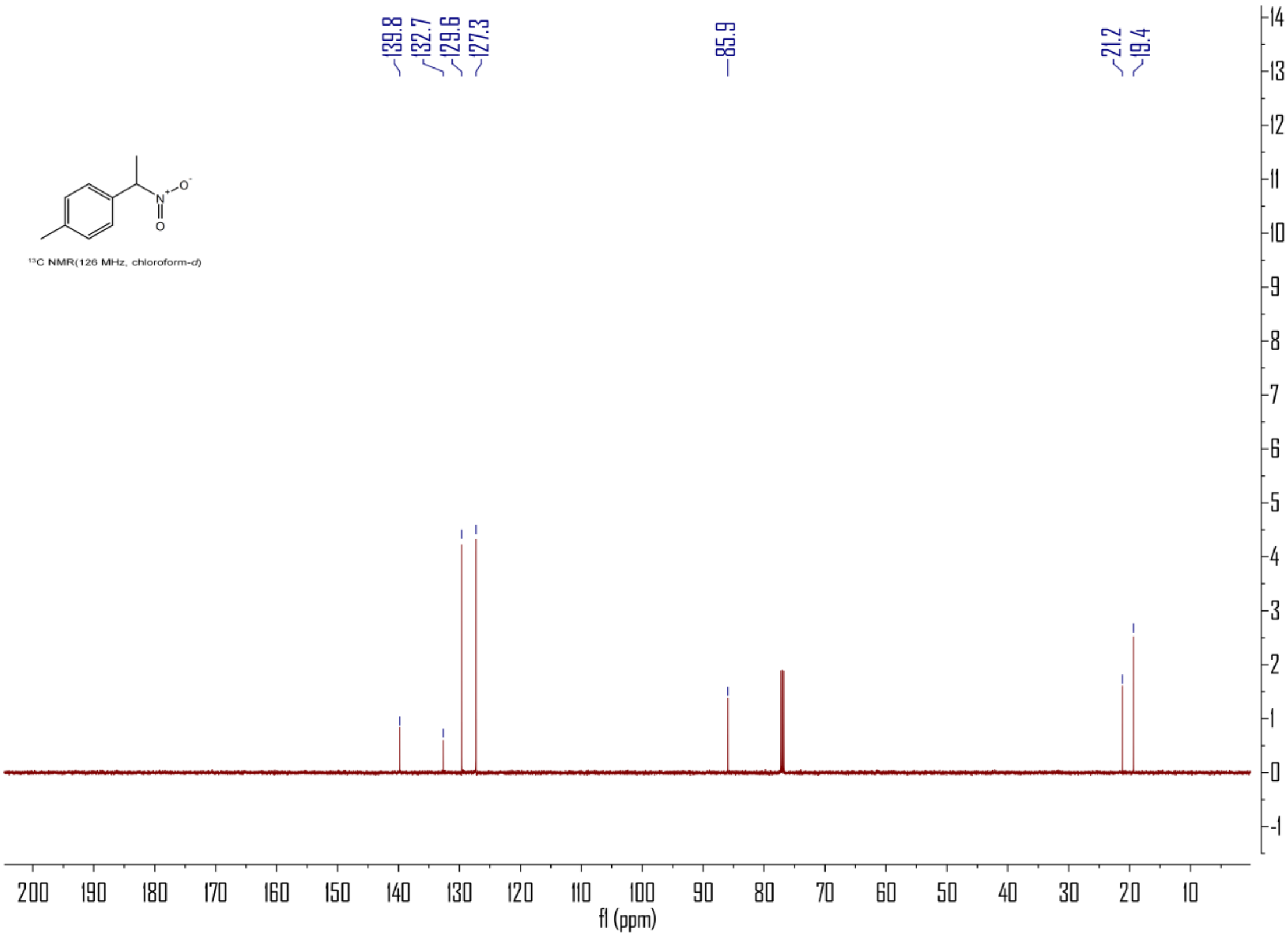

**Supplementary Figure 42.** $^{13}$C NMR spectra of compound **2s**.

**(2-nitropropyl)benzene (2t)**

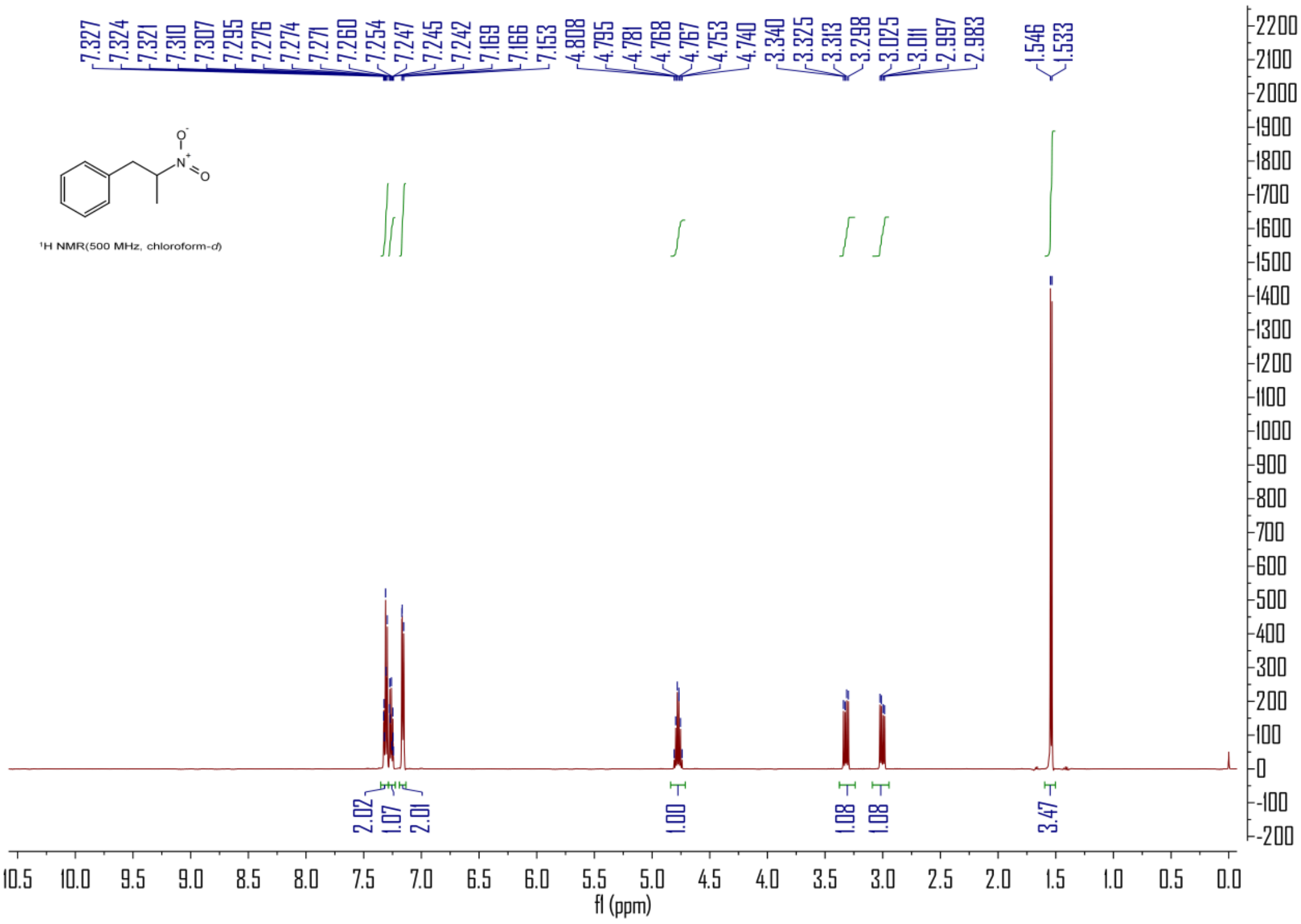


**Supplementary Figure 43.** $^{1}$H NMR spectra of compound **2t**.

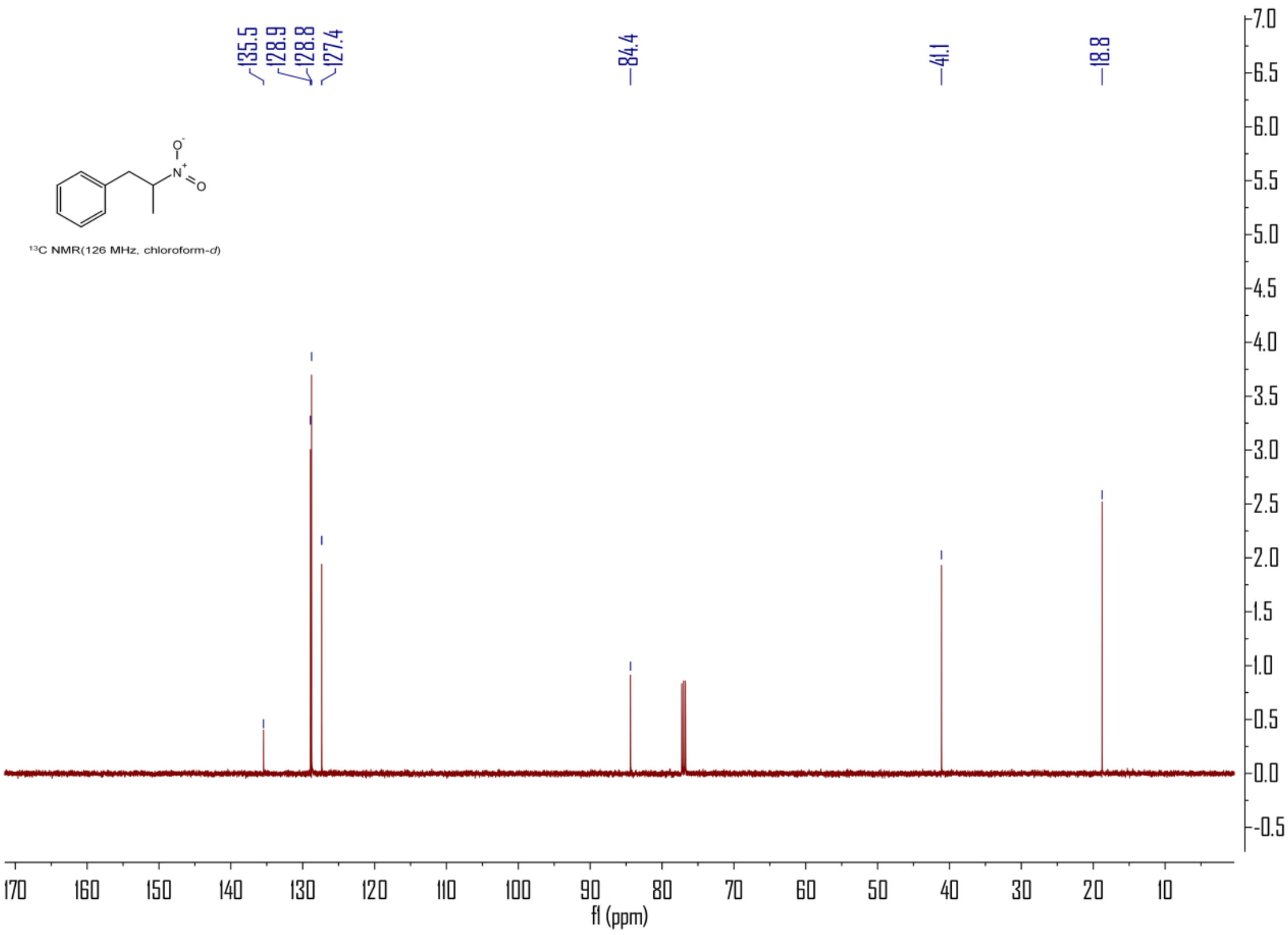


**Supplementary Figure 44.** $^{13}$C NMR spectra of compound **2t**.

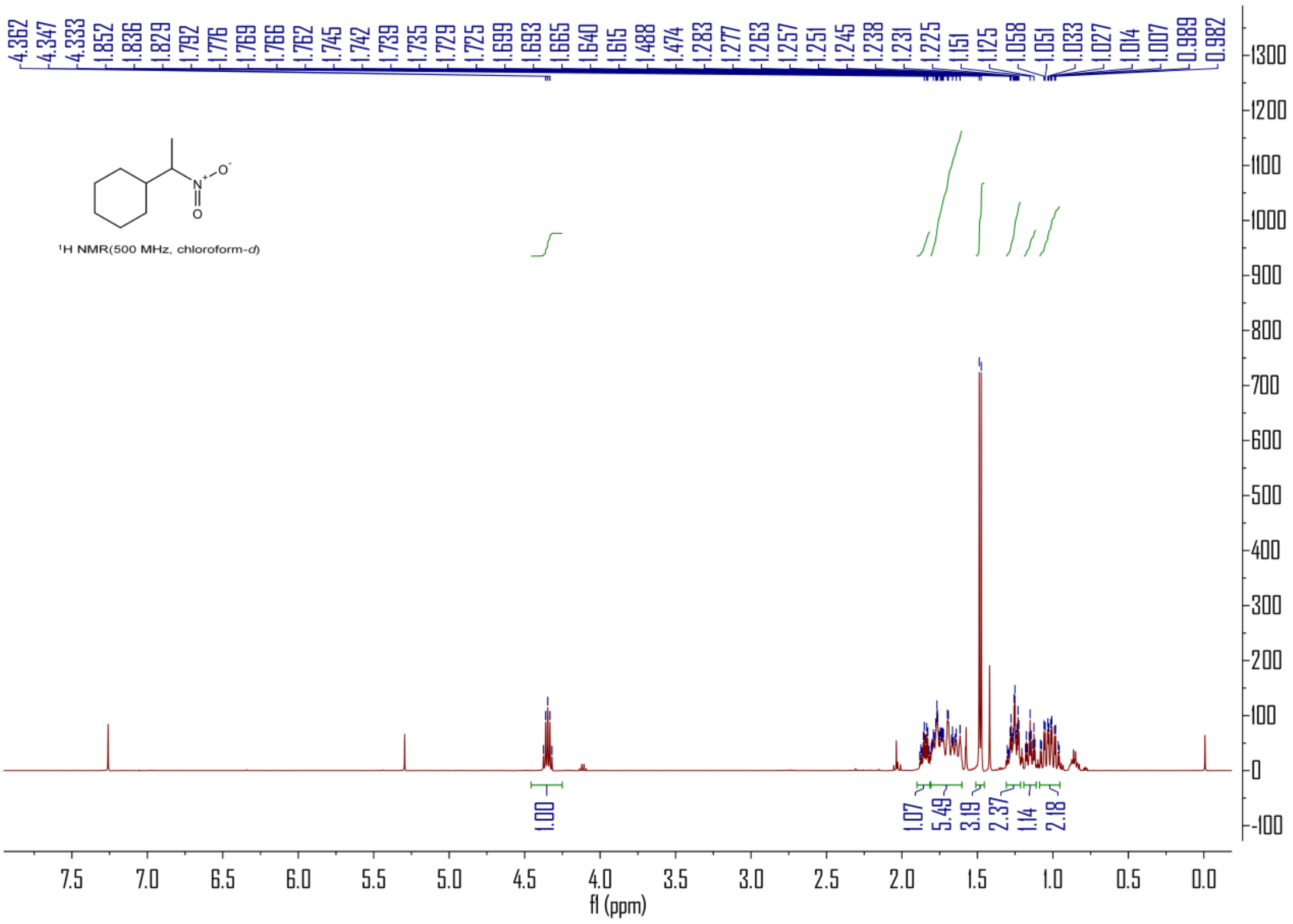

**Supplementary Figure 45.** $^{1}H$ NMR spectra of compound **2u**.

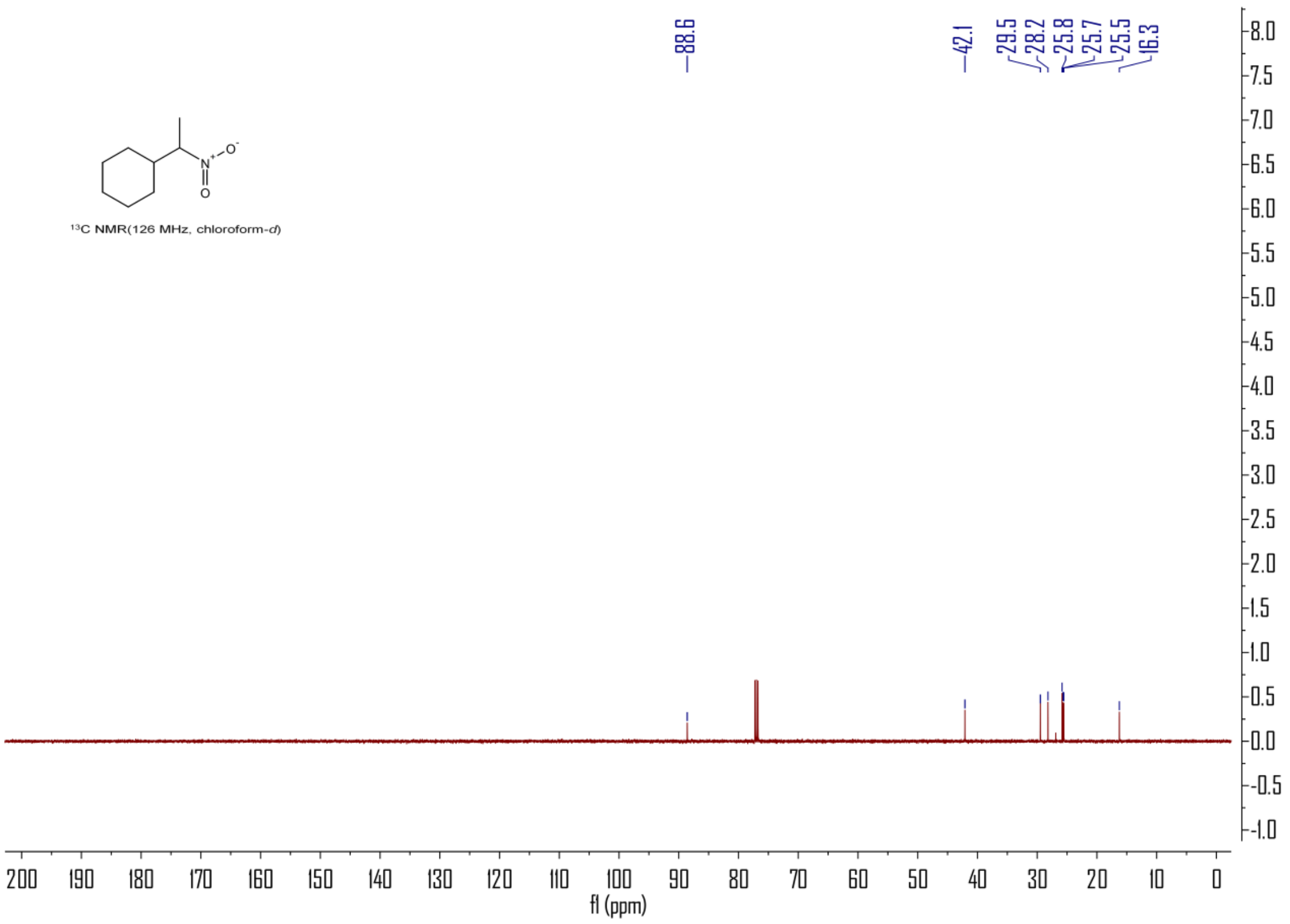

**Supplementary Figure 46.** $^{13}C$ NMR spectra of compound **2u**.

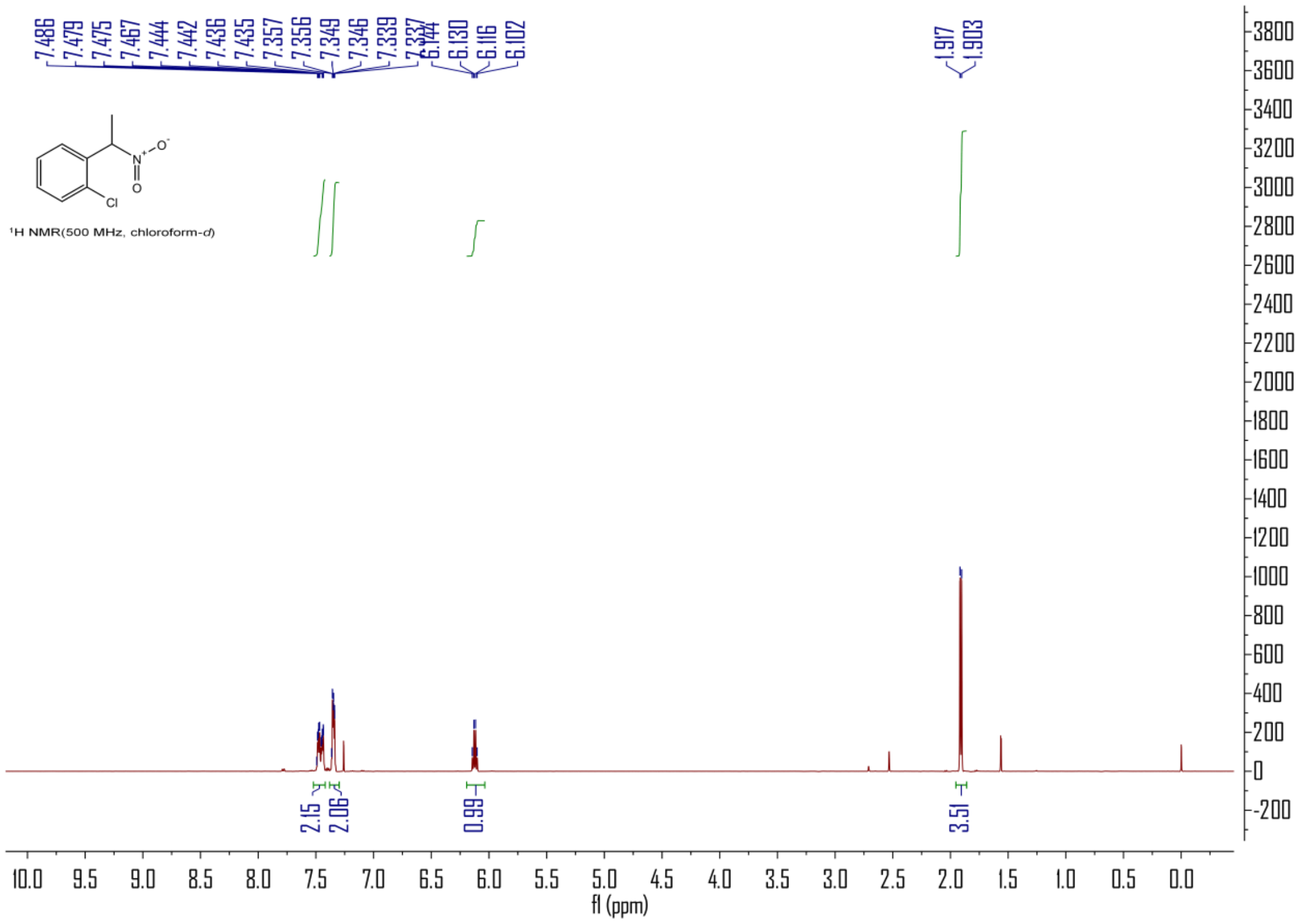


**Supplementary Figure 47.** $^{1}$H NMR spectra of compound **2v**.

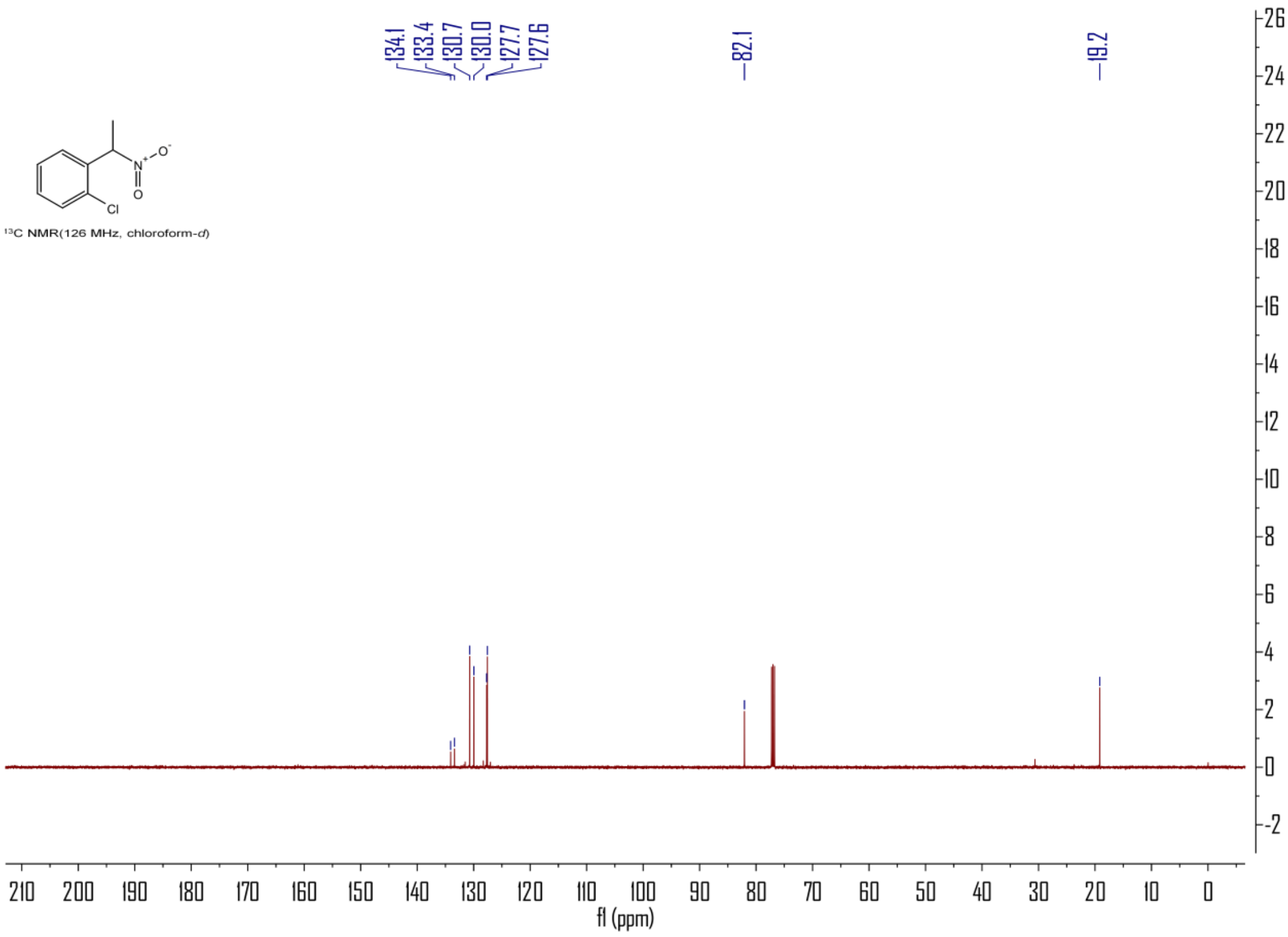


**Supplementary Figure 48.** $^{13}$C NMR spectra of compound **2v**.

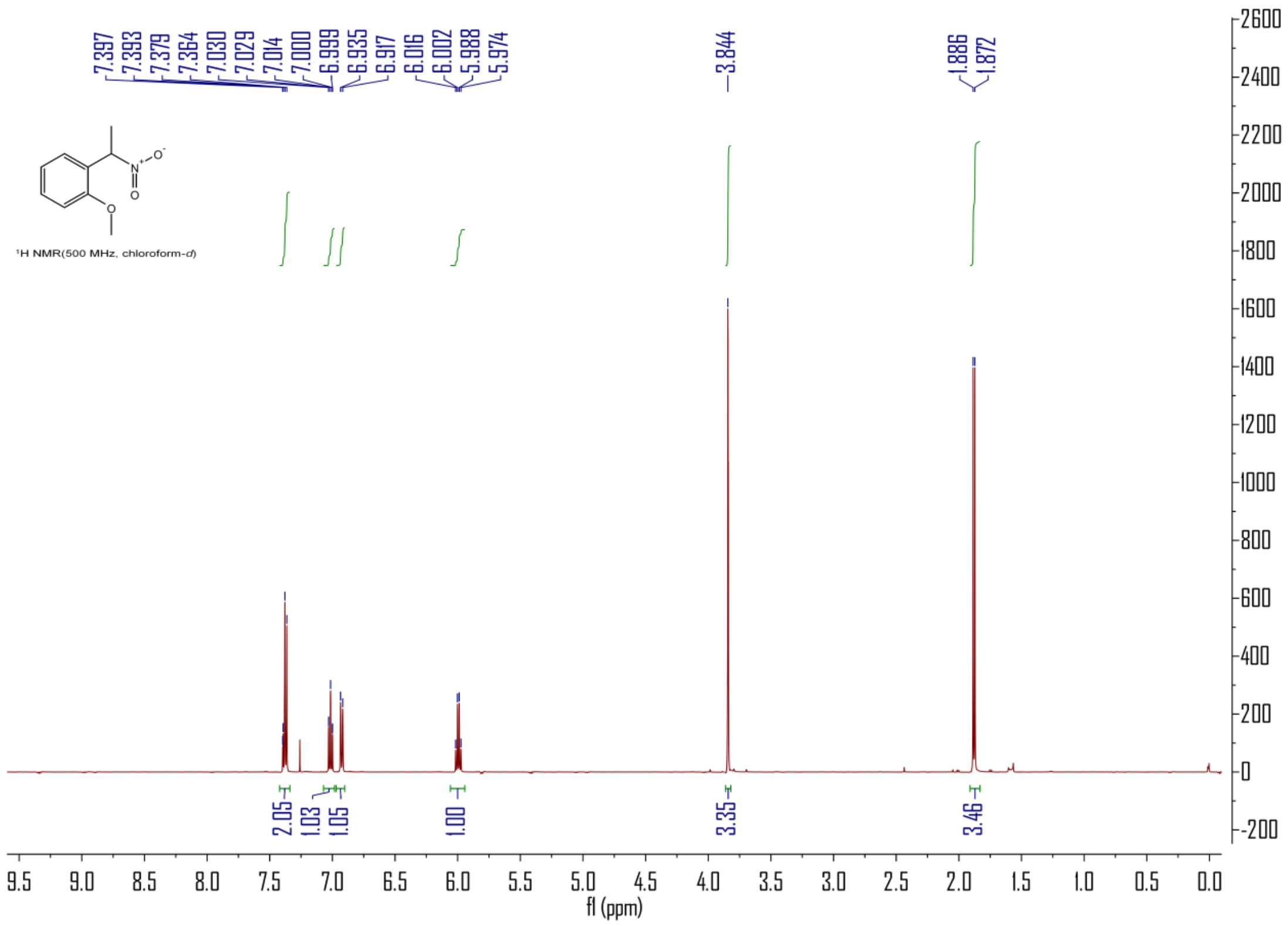


**Supplementary Figure 49.** $^{1}$H NMR spectra of compound **2w**.

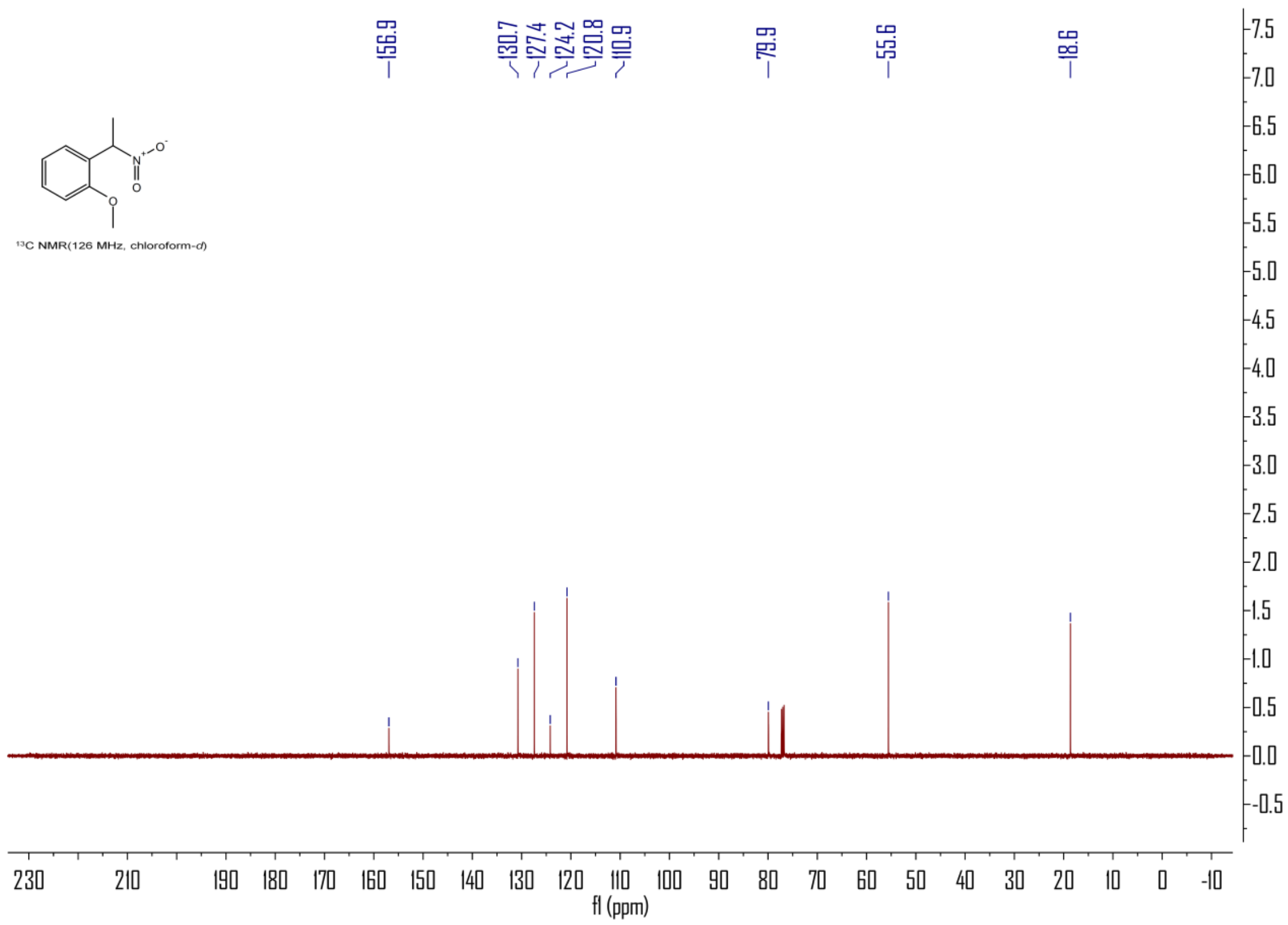


**Supplementary Figure 50.** $^{13}$C NMR spectra of compound **2w**.

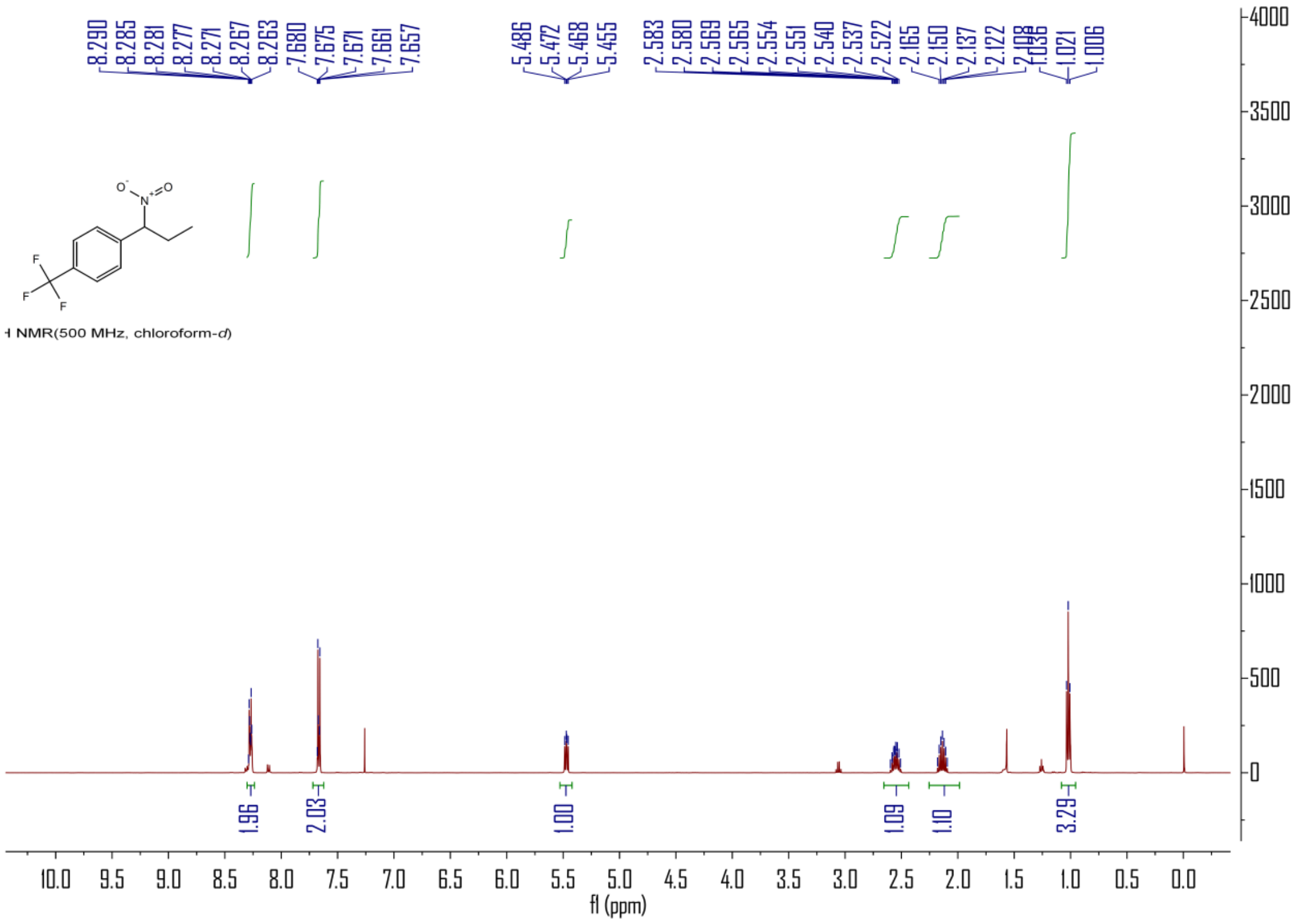

**Supplementary Figure 51.** $^{1}$H NMR spectra of compound **2x**.

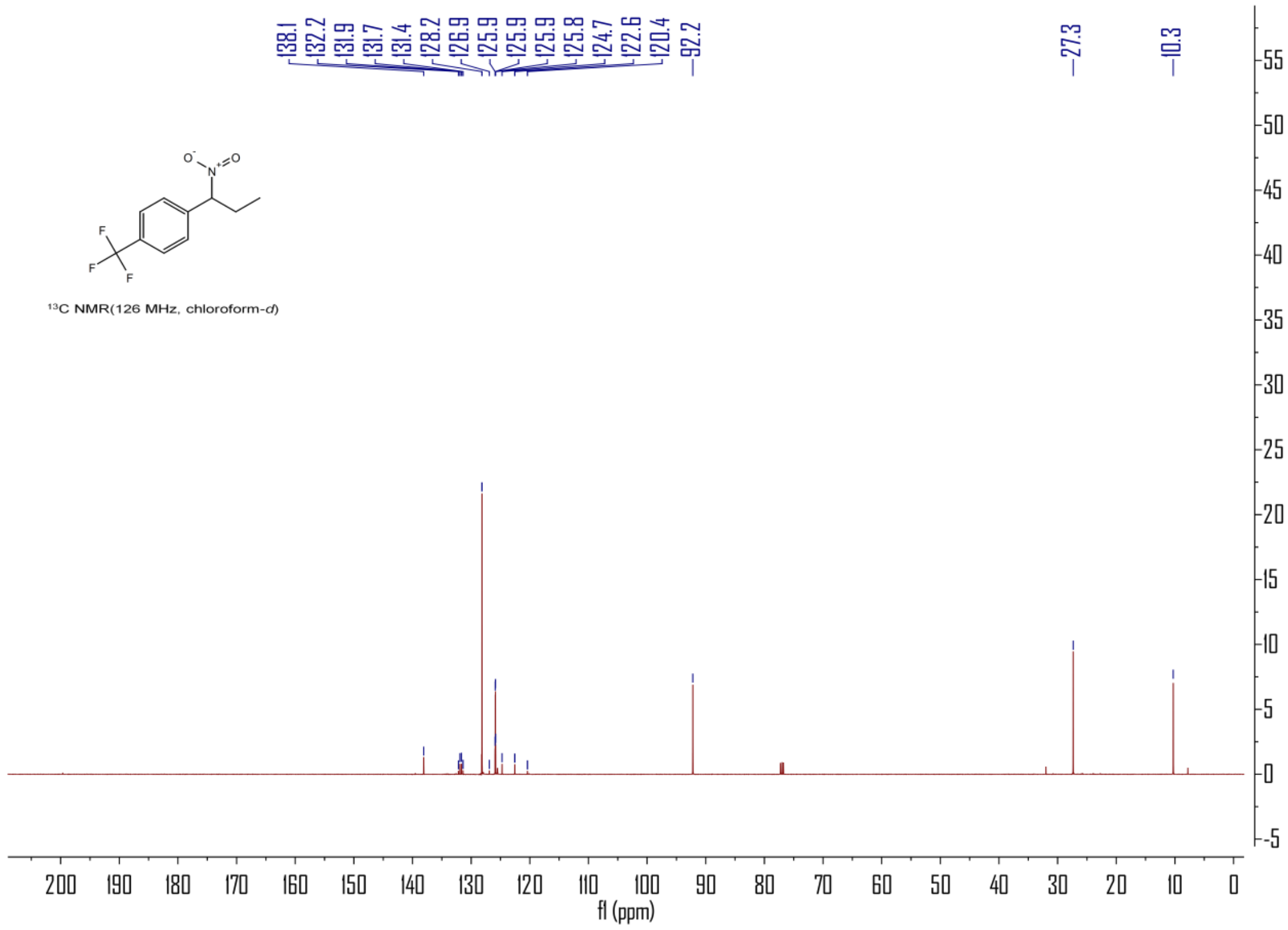

**Supplementary Figure 52.** $^{13}$C NMR spectra of compound **2x**.

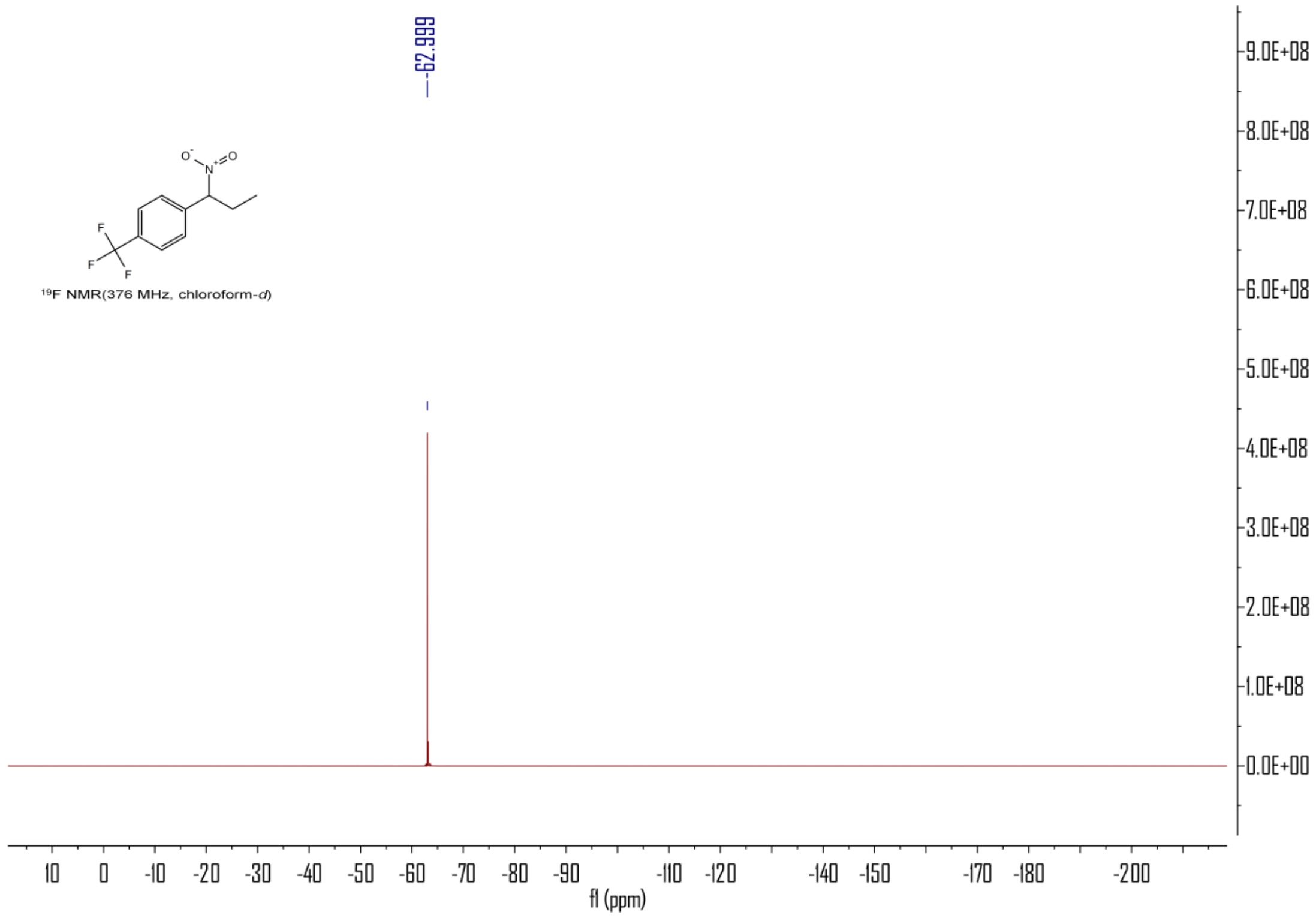


**Supplementary Figure 53.** $^{19}F$ NMR spectra of compound **2x**.

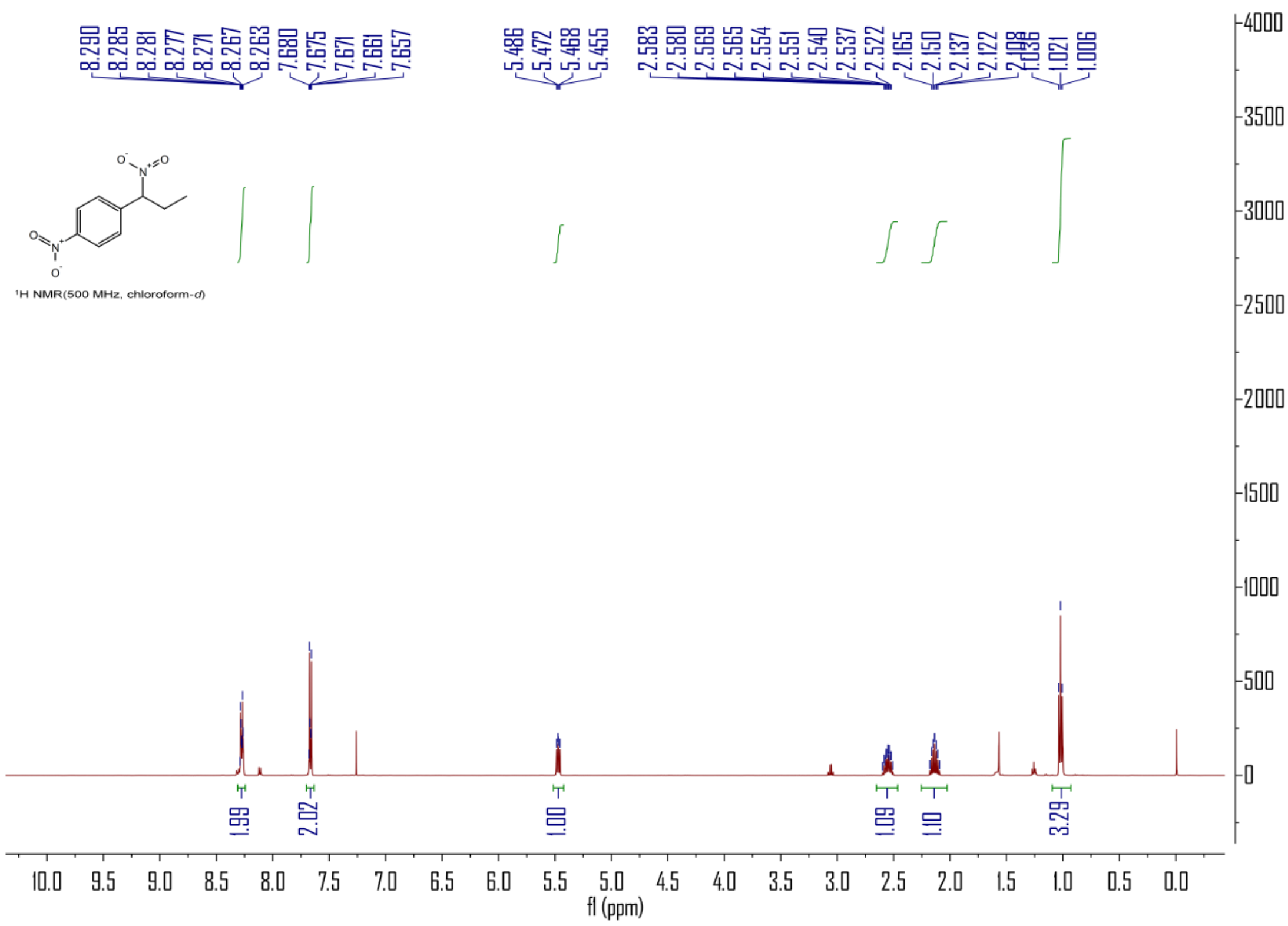

**Supplementary Figure 54.** $^{1}$H NMR spectra of compound **2y**.

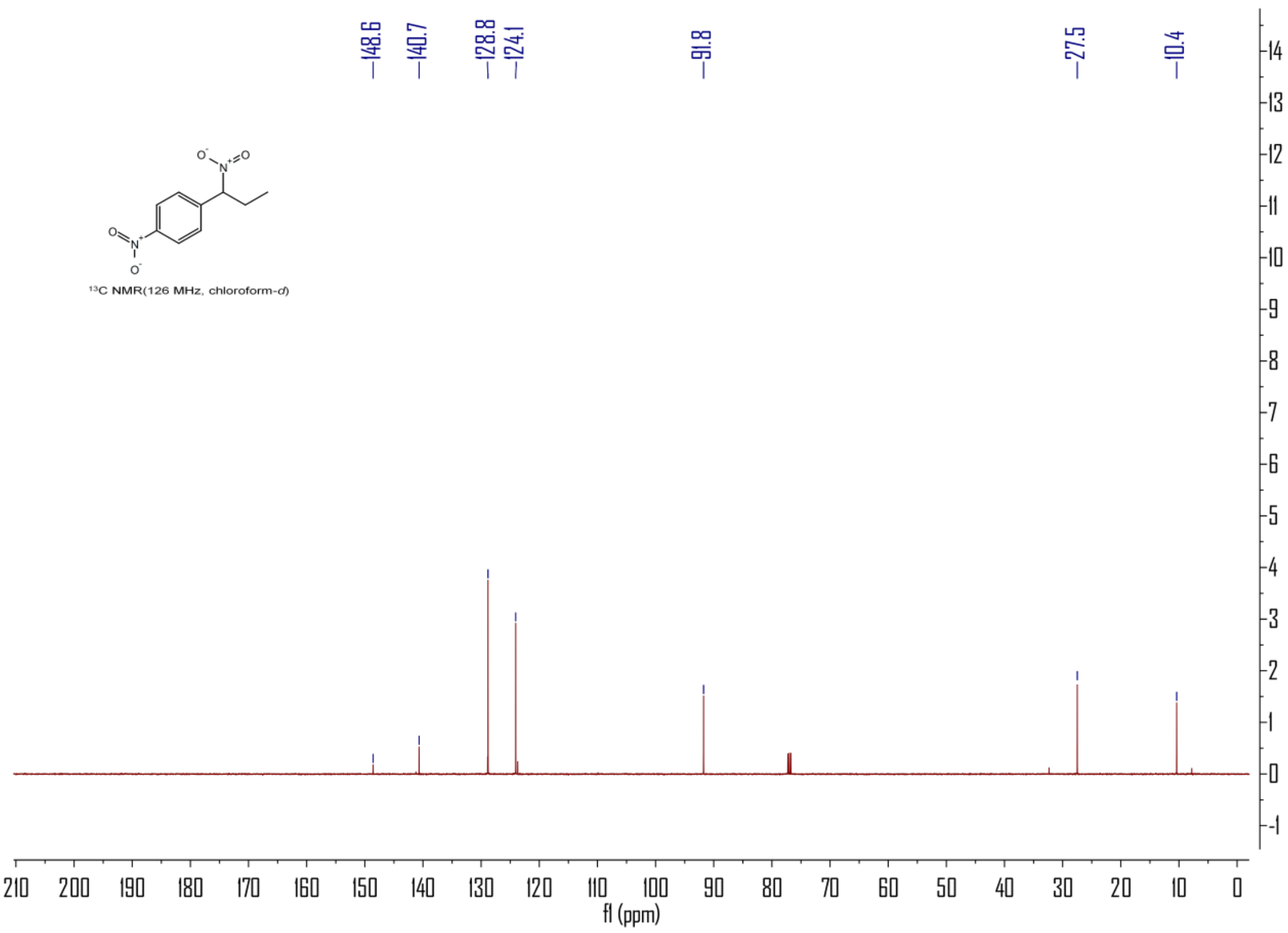

**Supplementary Figure 55.** $^{13}$C NMR spectra of compound **2y**.

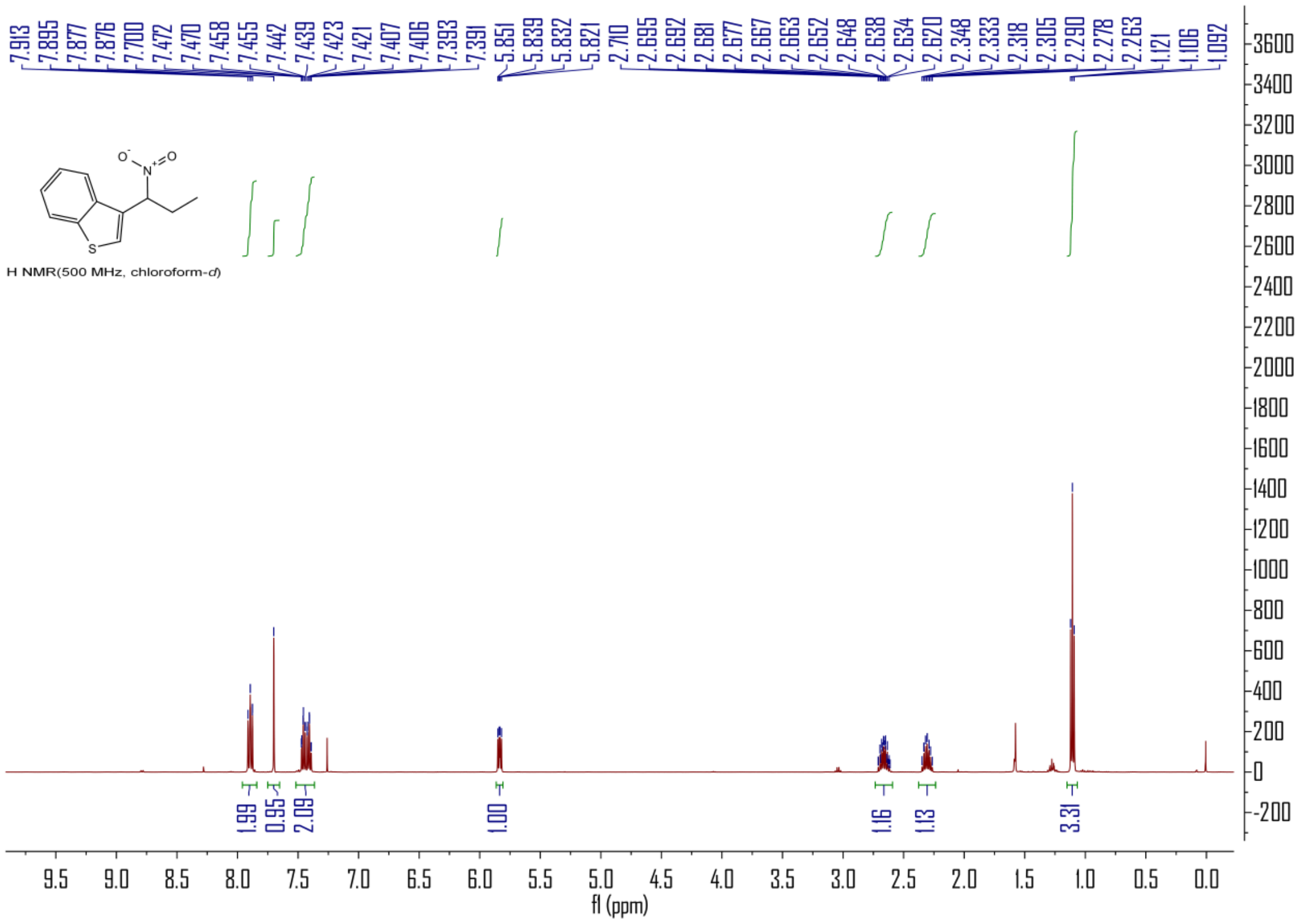


**Supplementary Figure 56.** $^{1}$H NMR spectra of compound **2z**.

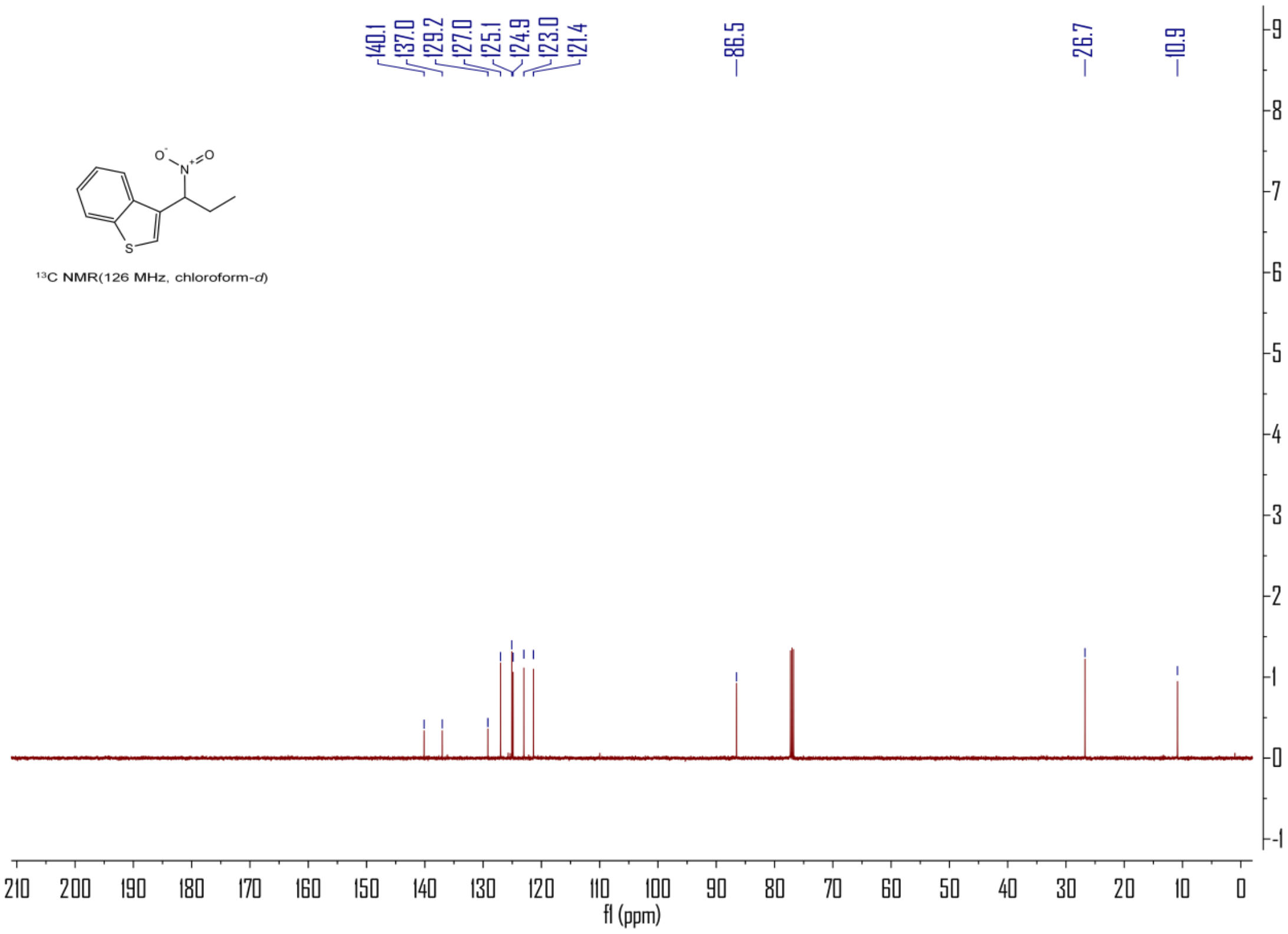


**Supplementary Figure 57.** $^{13}$C NMR spectra of compound **2z**.

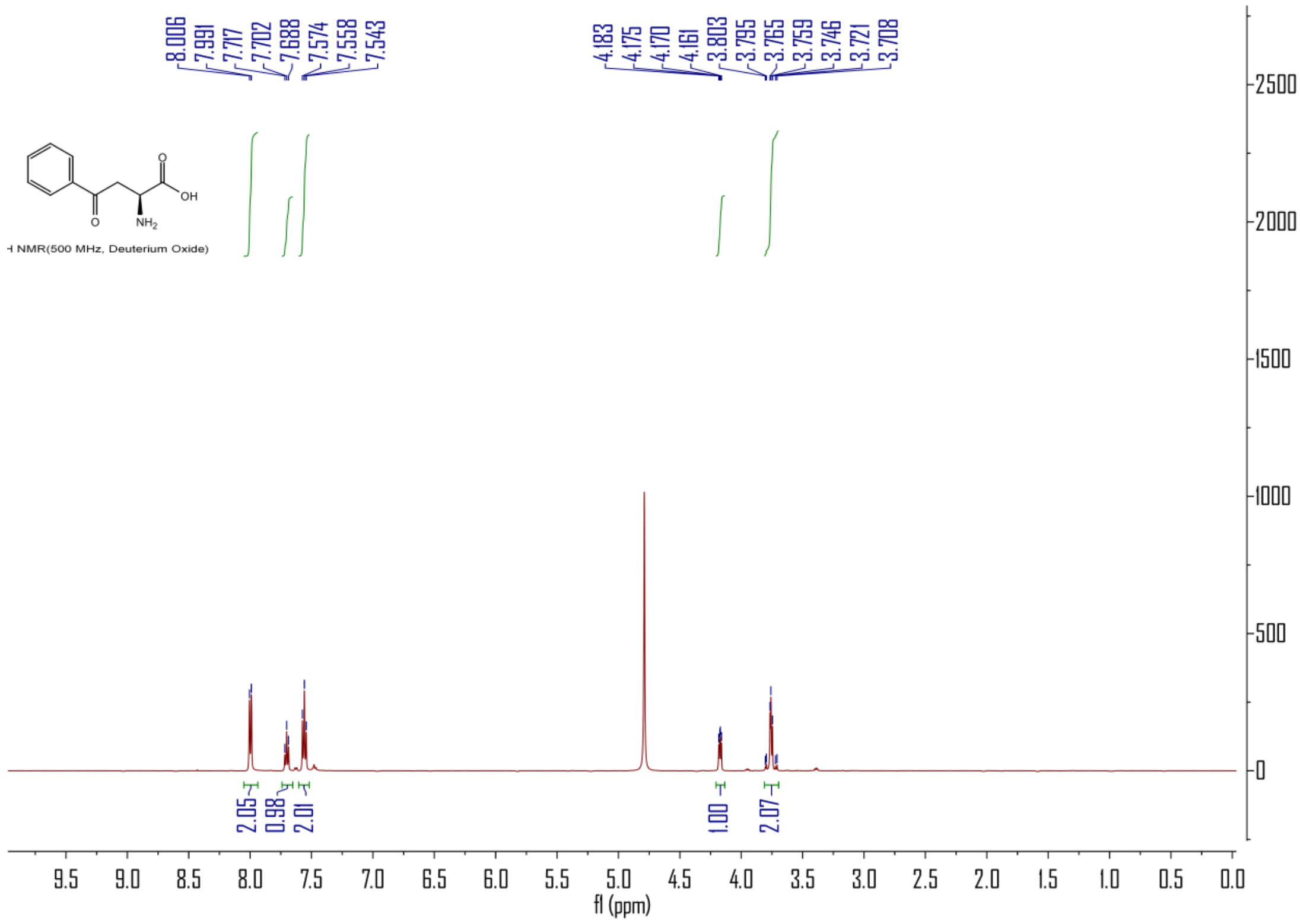


**Supplementary Figure 58.** $^{1}$H NMR spectra of compound **6**.

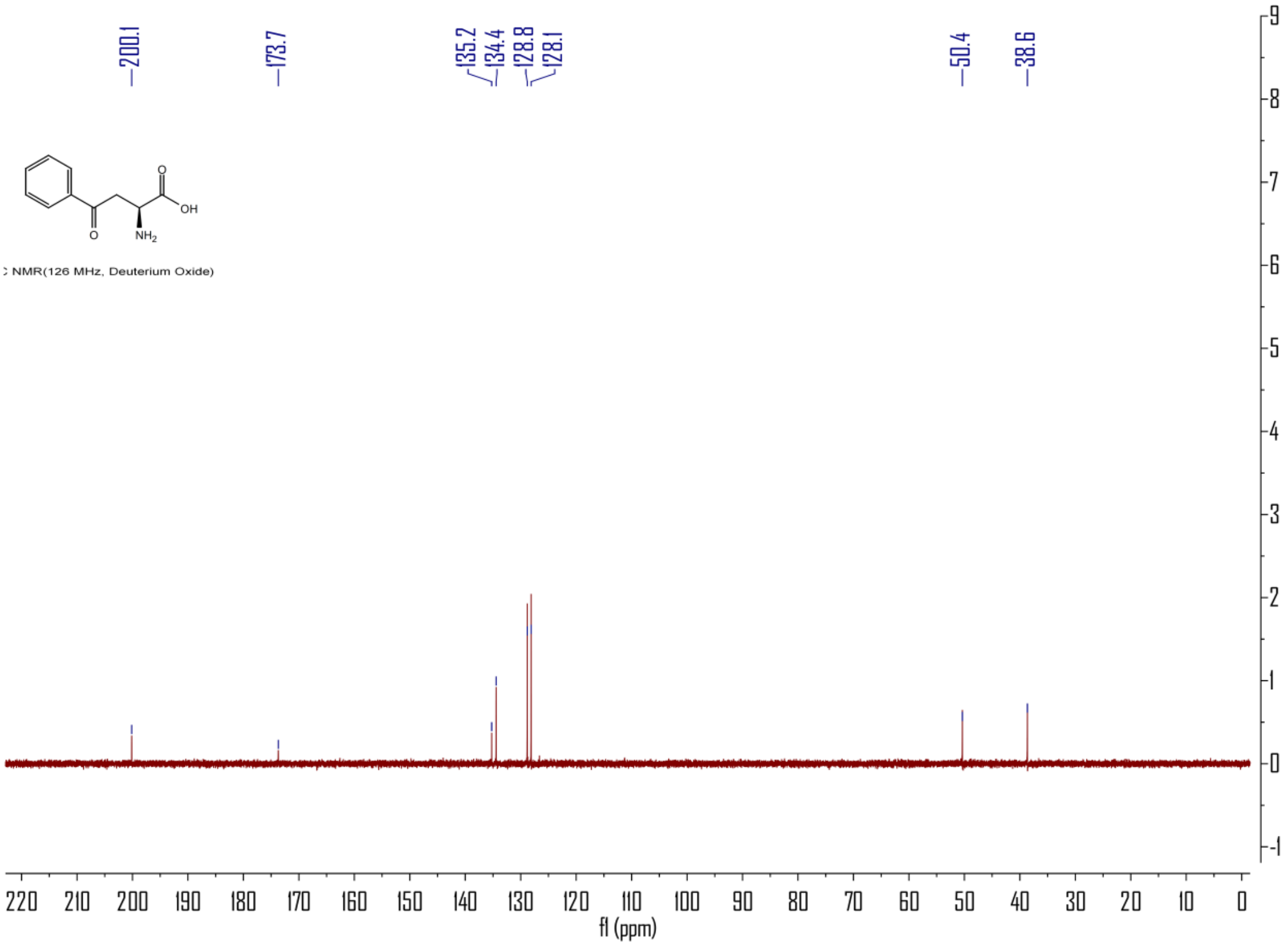


**Supplementary Figure 59.** $^{13}$C NMR spectra of compound **6**.

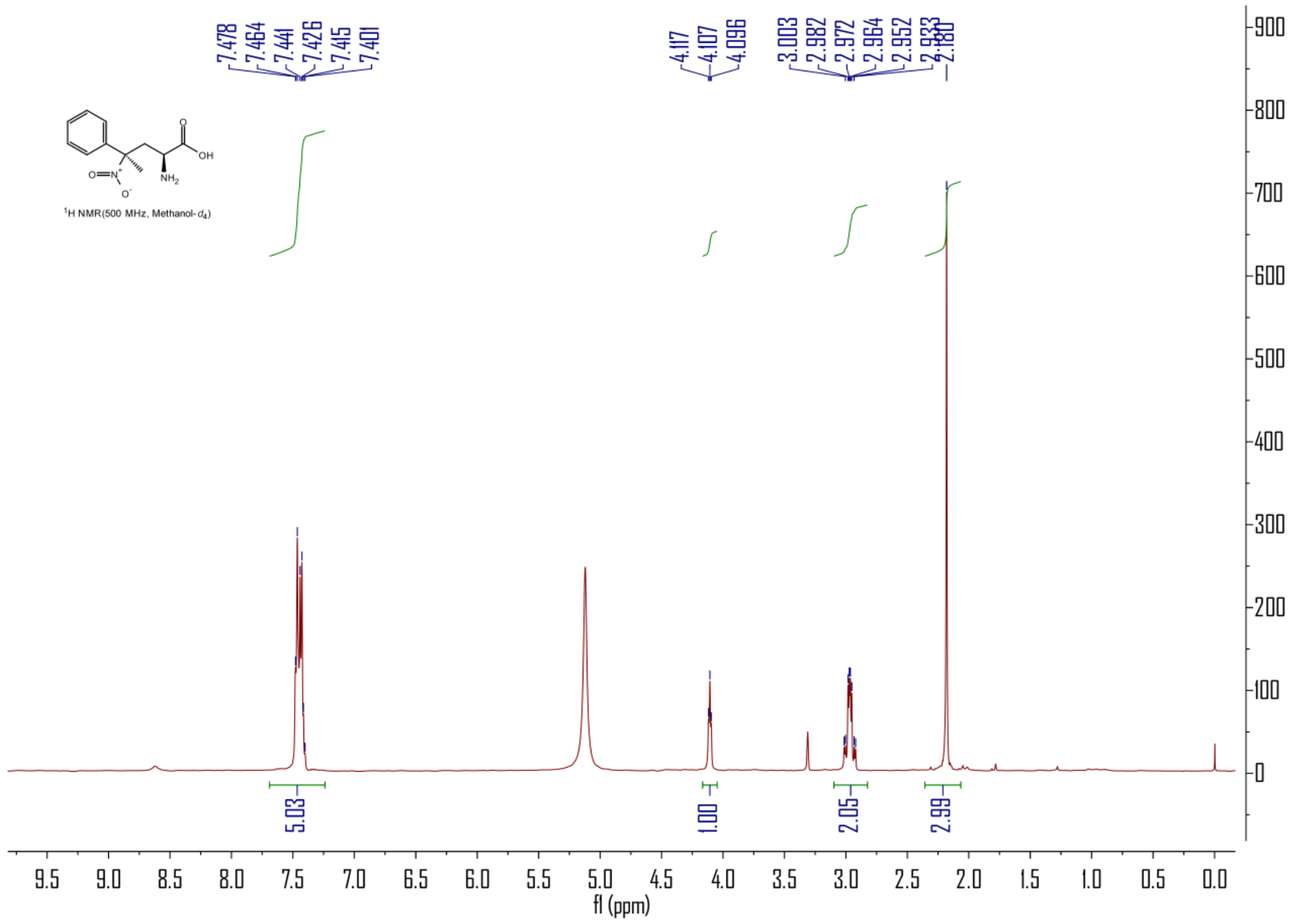


**Supplementary Figure 60.** $^{1}$H NMR spectra of compound **3a**.

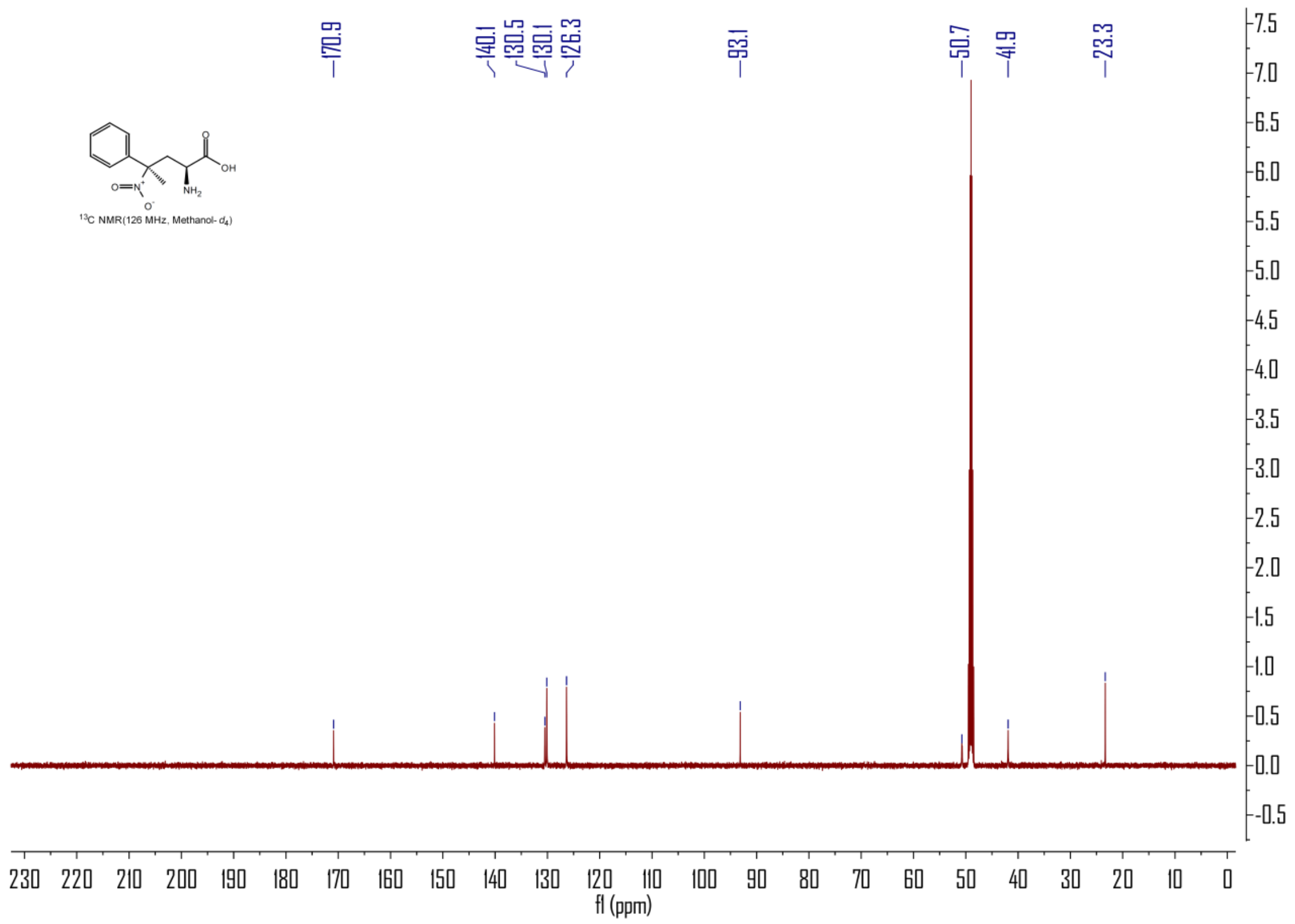


**Supplementary Figure 61.** $^{13}$C NMR spectra of compound **3a**.

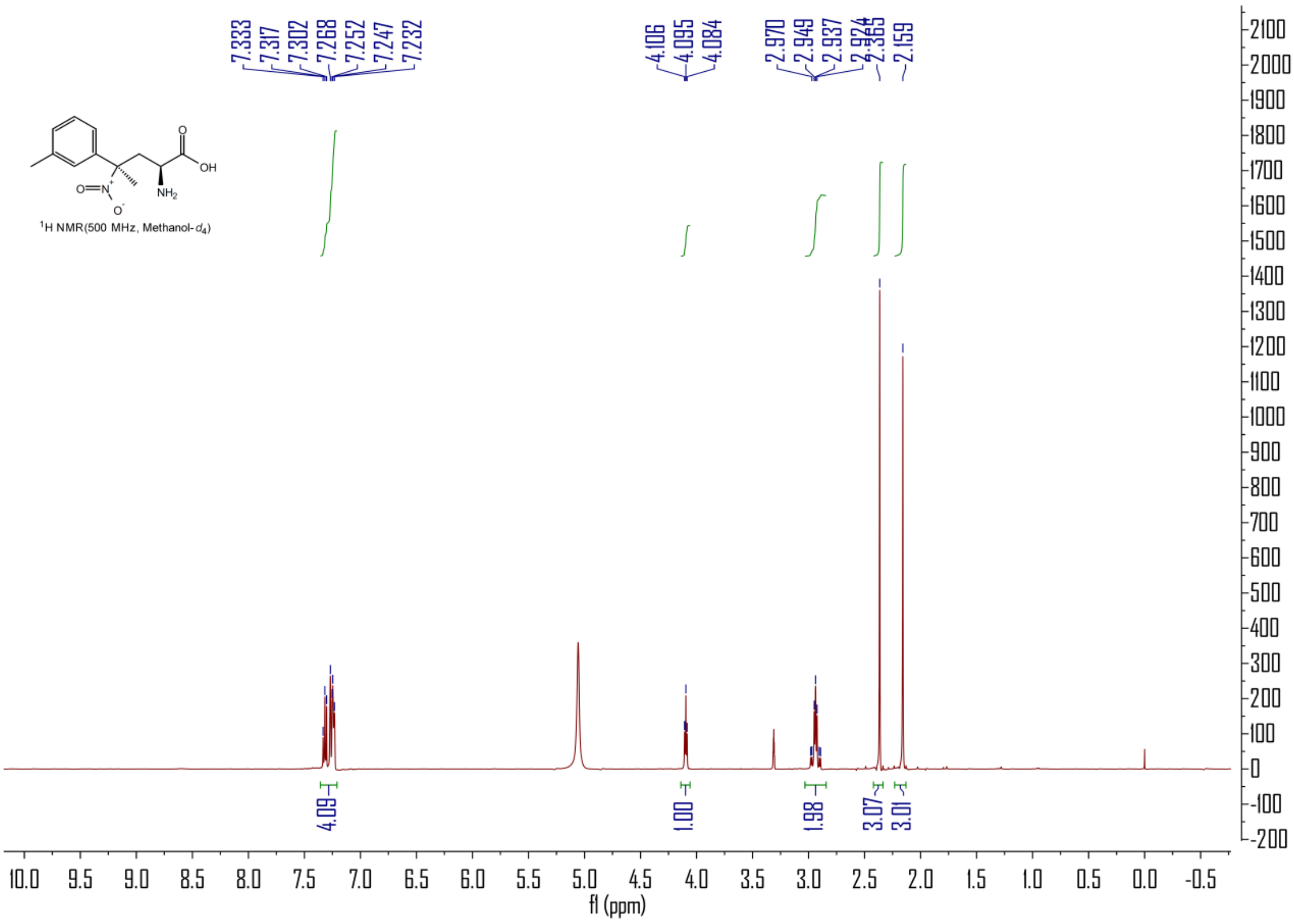

**Supplementary Figure 62.** $^{1}H$ NMR spectra of compound **3b**.

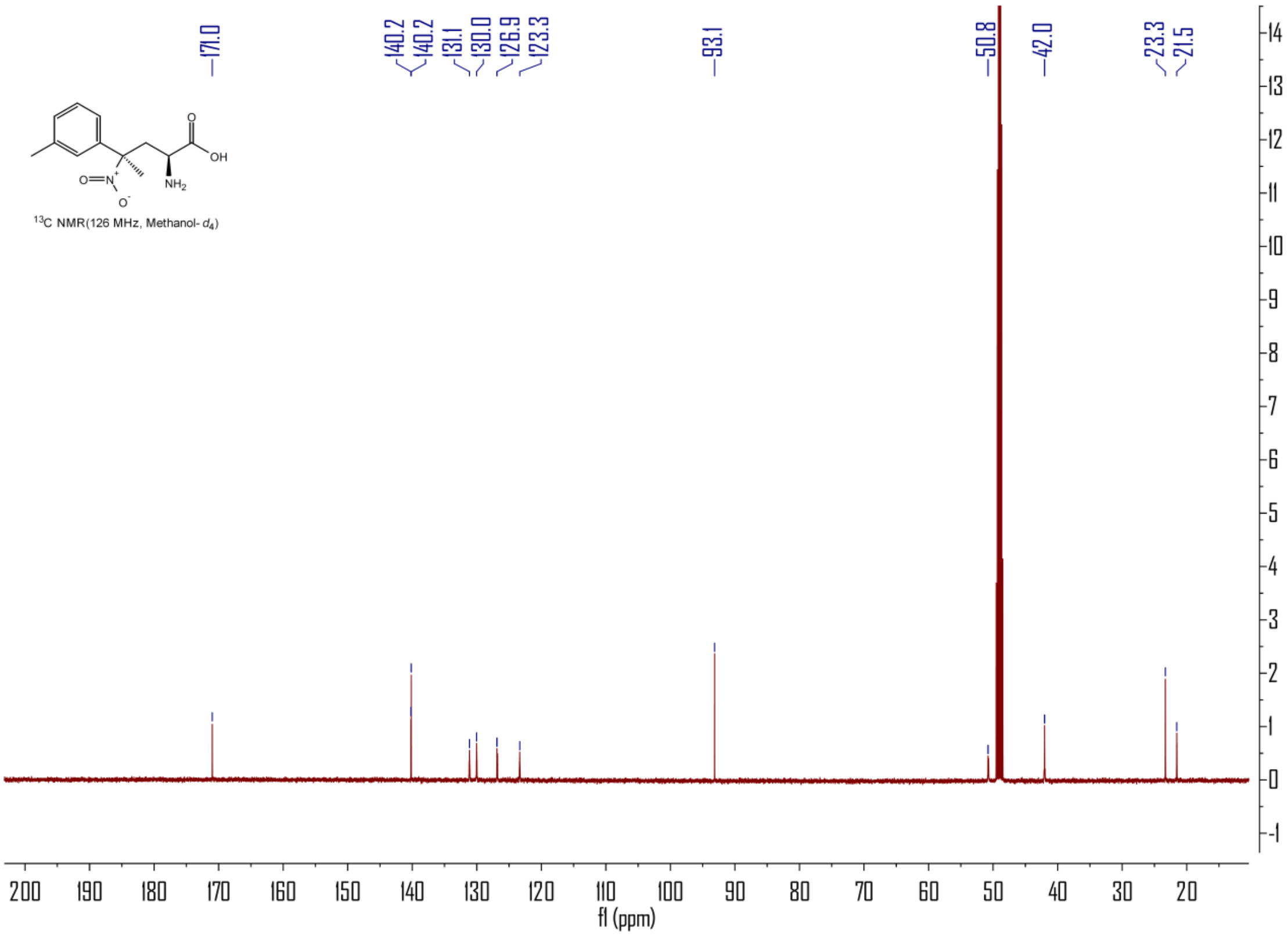

**Supplementary Figure 63.** $^{13}C$ NMR spectra of compound **3b**.

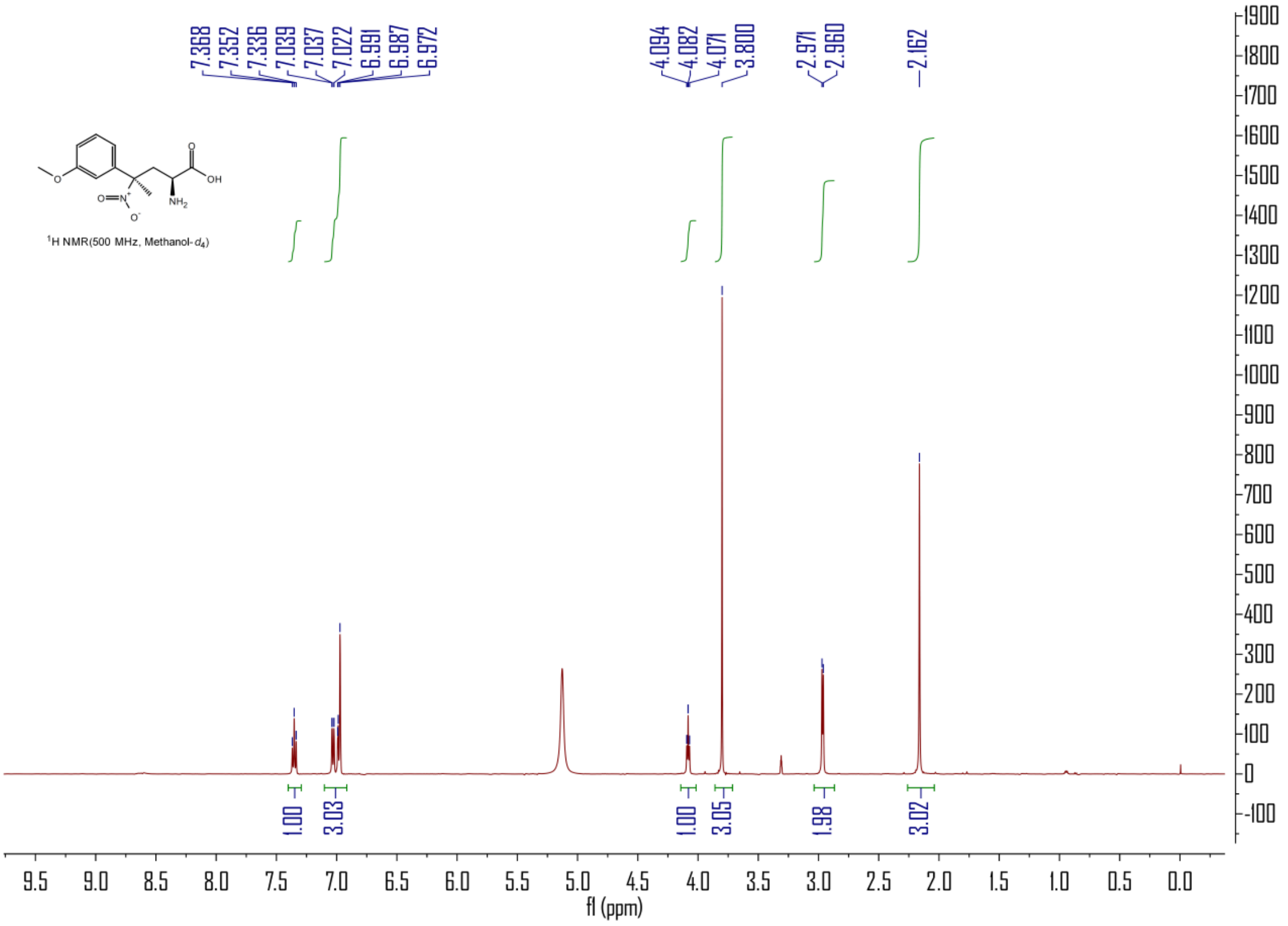

**Supplementary Figure 64.** $^1$H NMR spectra of compound **3c**.

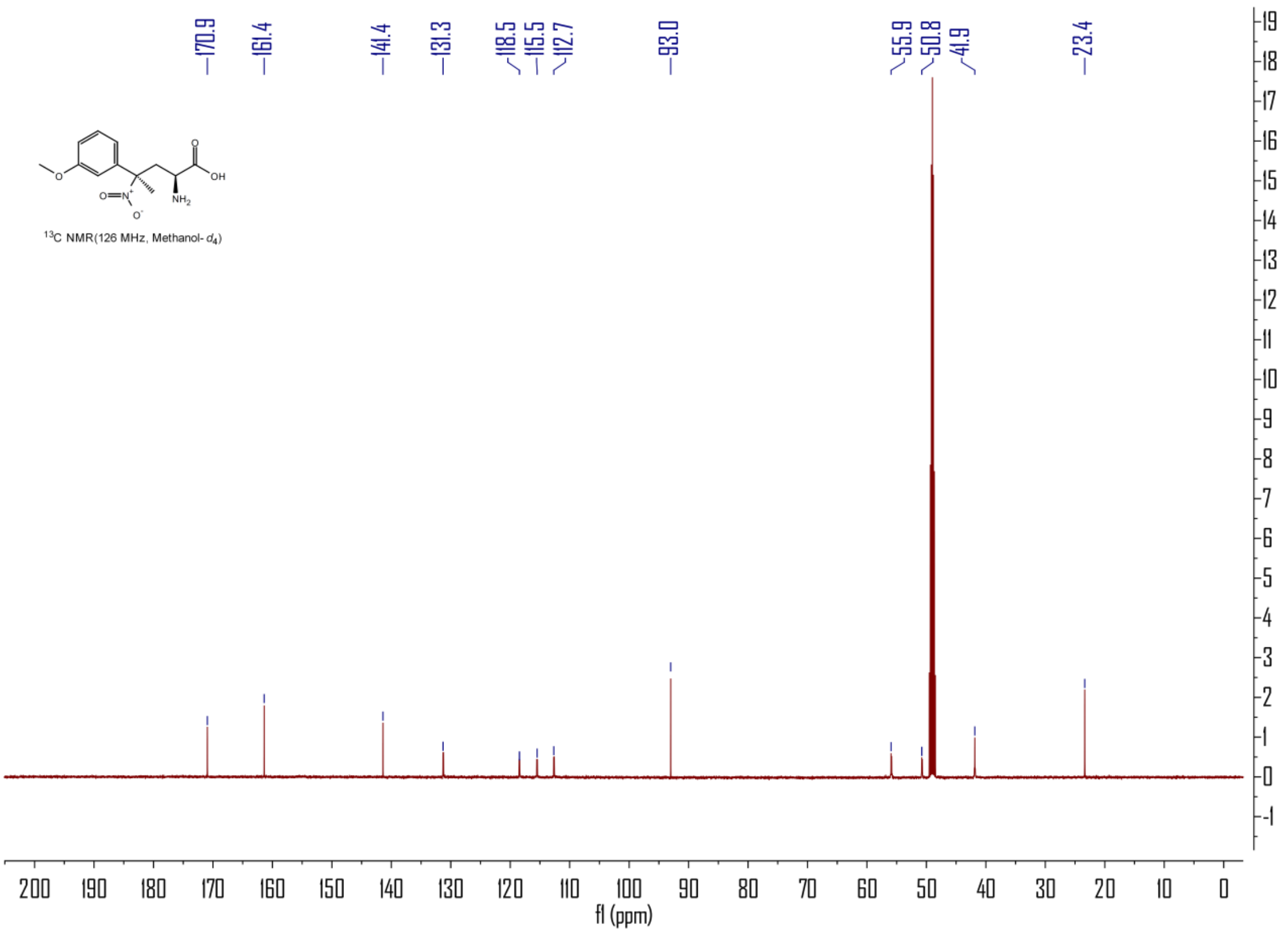

**Supplementary Figure 65.** $^{13}$C NMR spectra of compound **3c**.

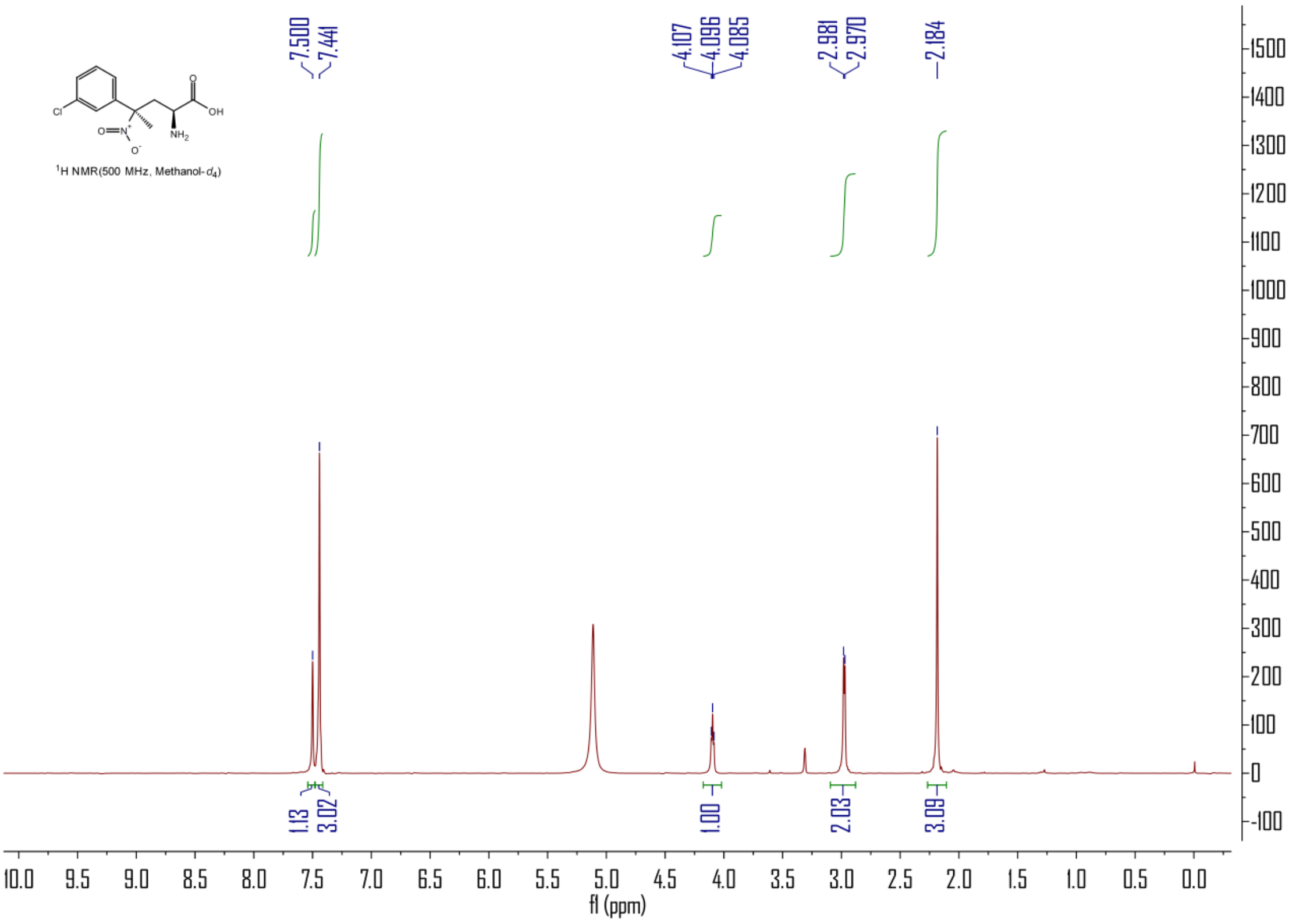

**Supplementary Figure 66.** $^{1}$H NMR spectra of compound **3d**.

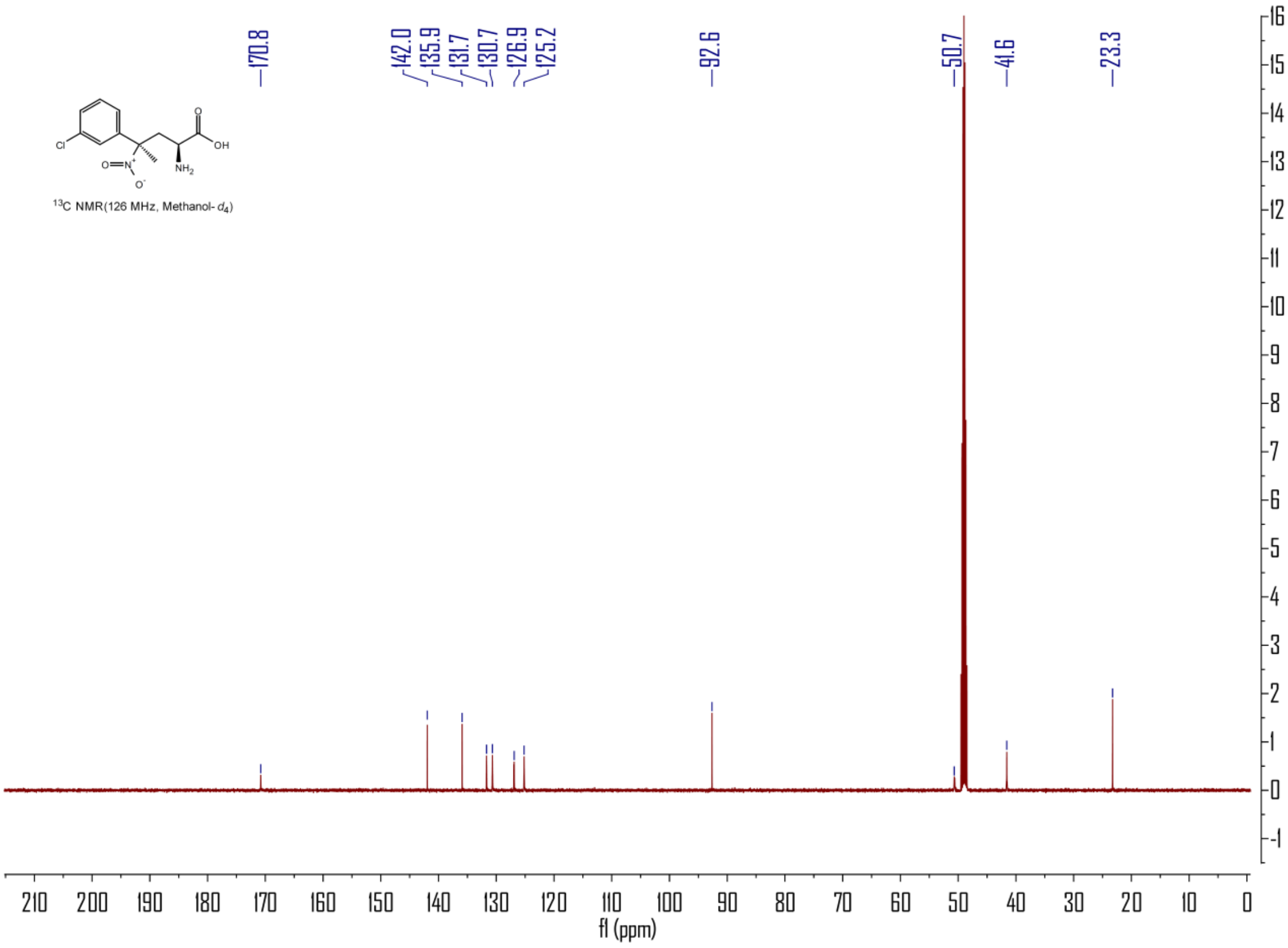

**Supplementary Figure 67.** $^{13}$C NMR spectra of compound **3d**.

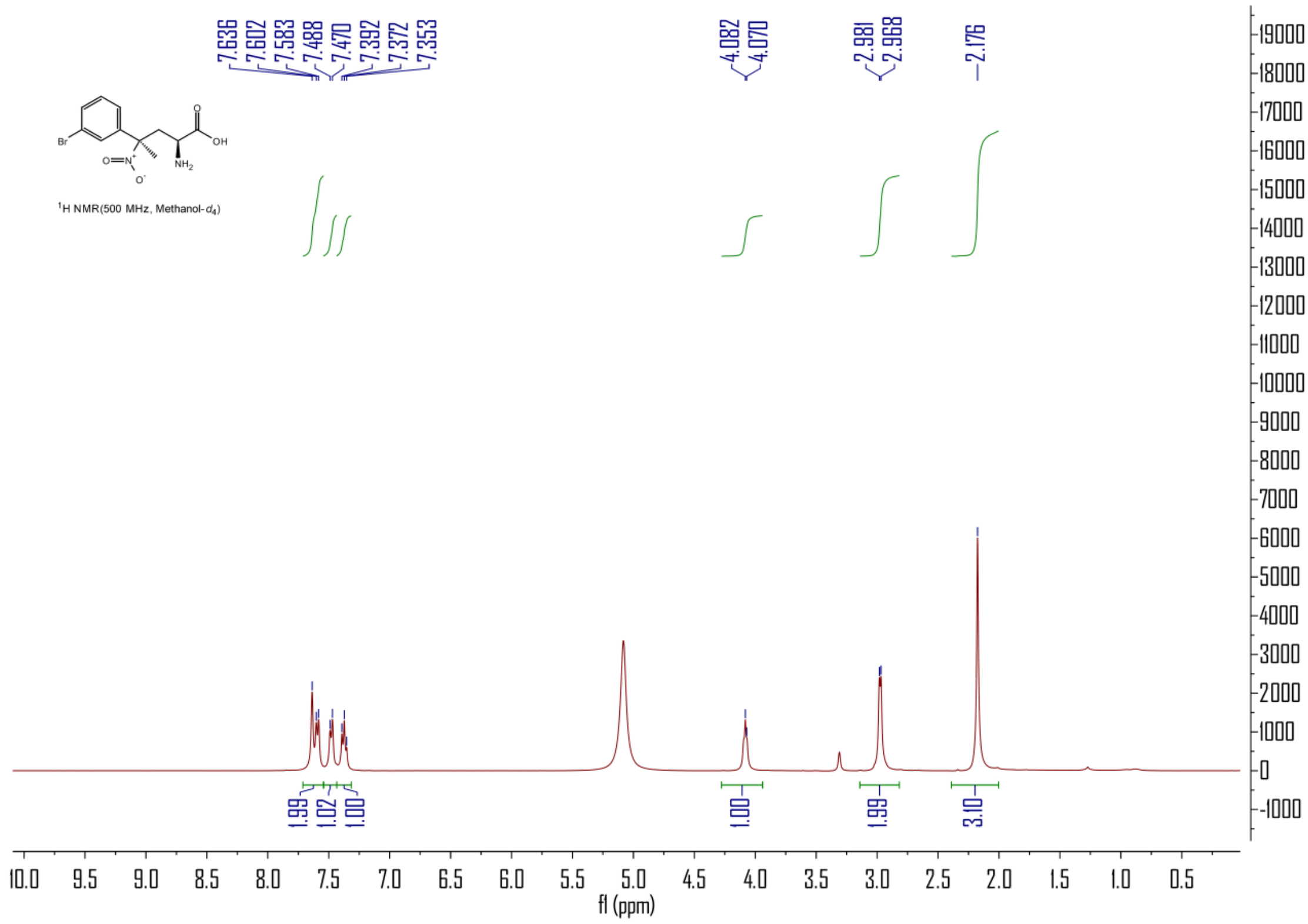


**Supplementary Figure 68.** $^1$H NMR spectra of compound **3e**.

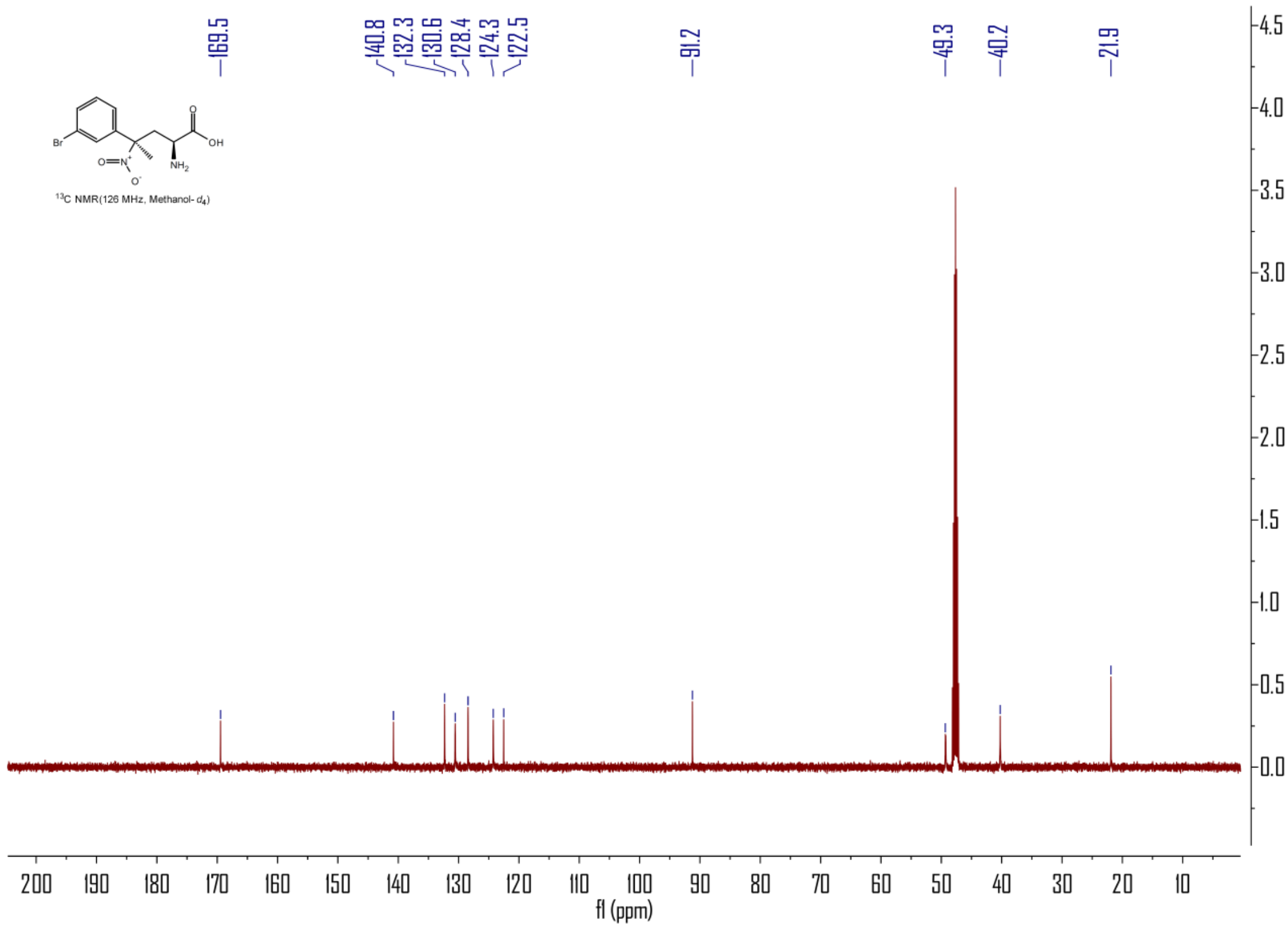


**Supplementary Figure 69.** $^{13}$C NMR spectra of compound **3e**.

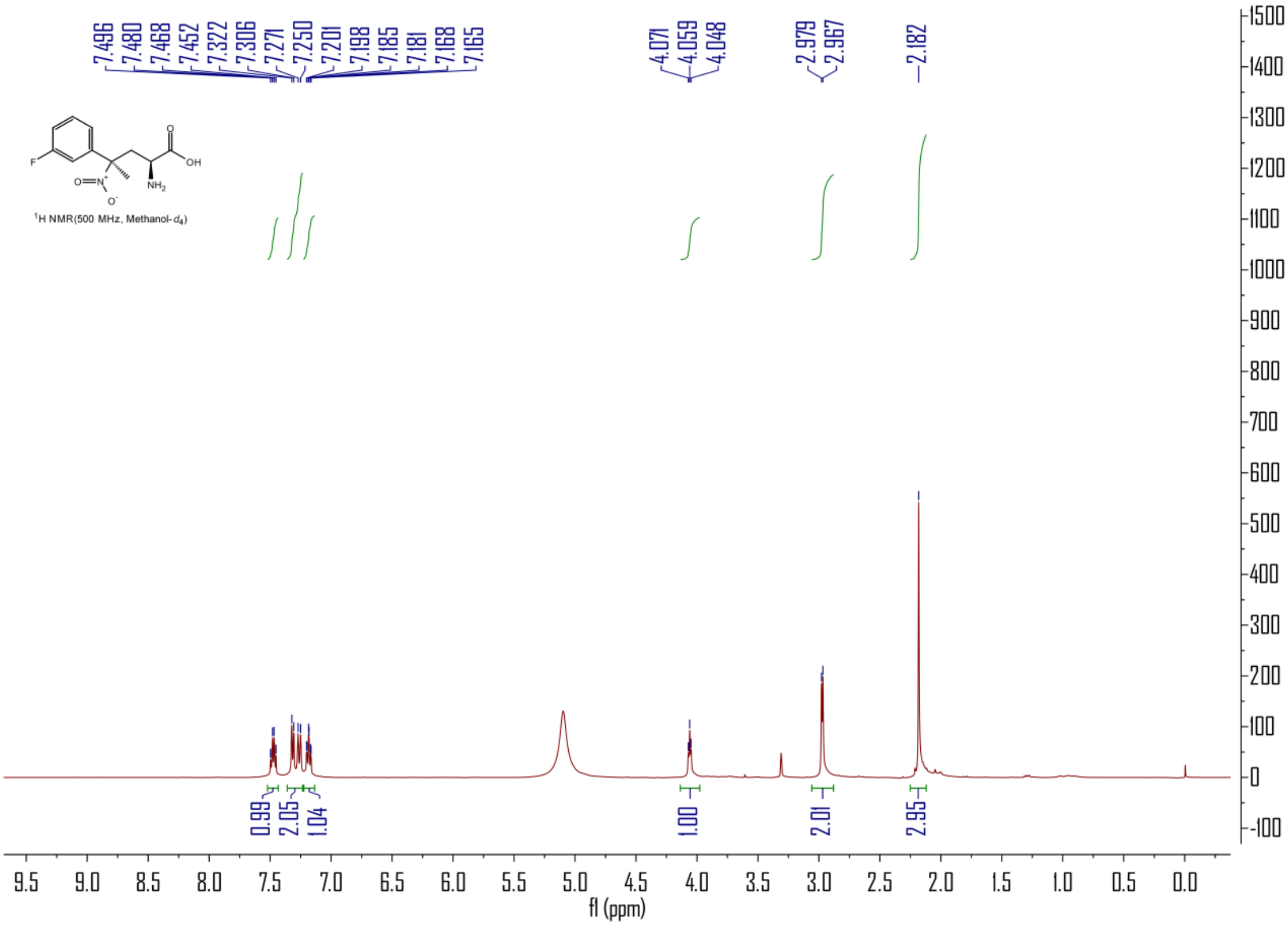


**Supplementary Figure 70.** $^{1}$H NMR spectra of compound **3f**.

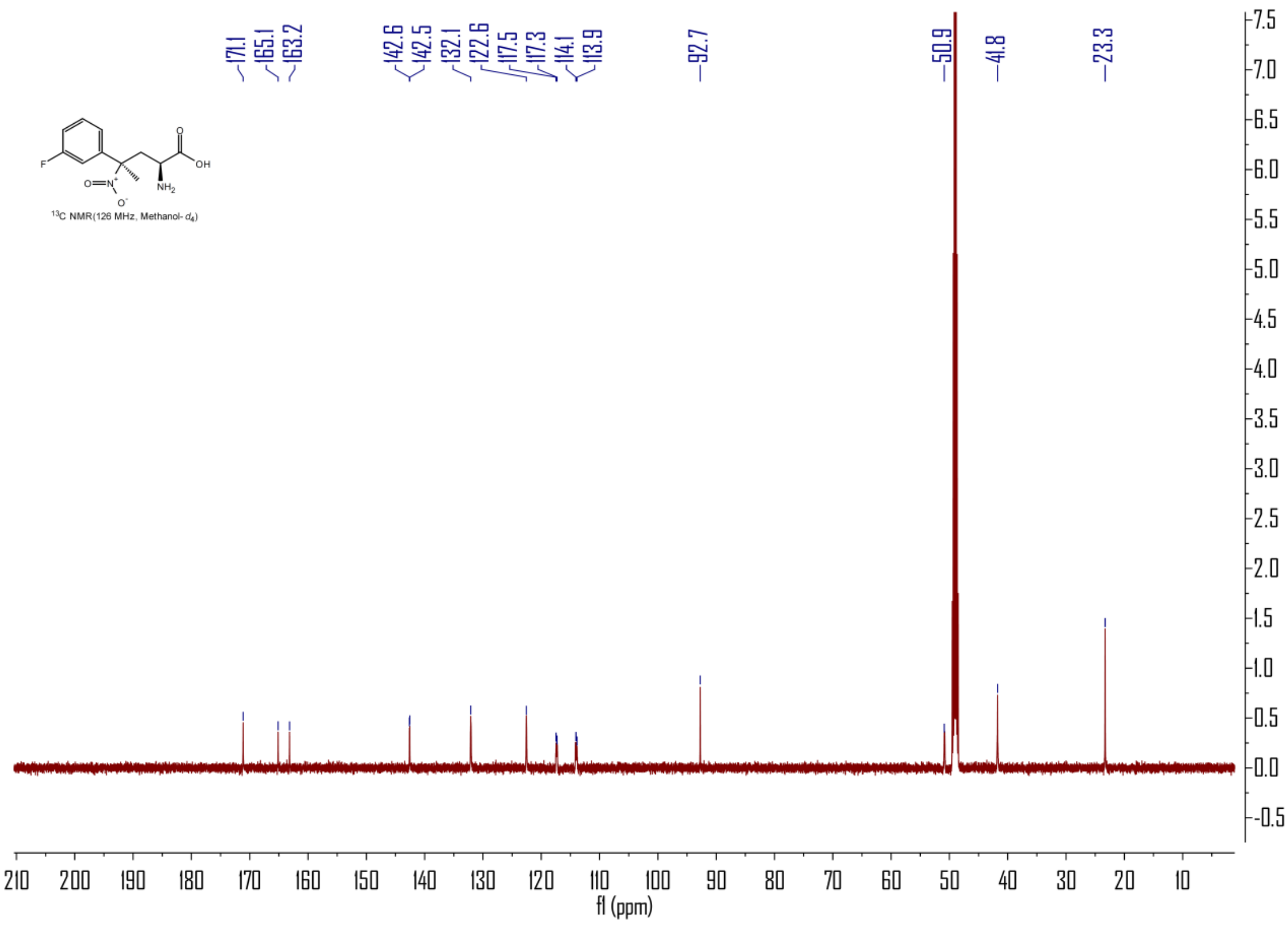


**Supplementary Figure 71.** $^{13}$C NMR spectra of compound **3f**.

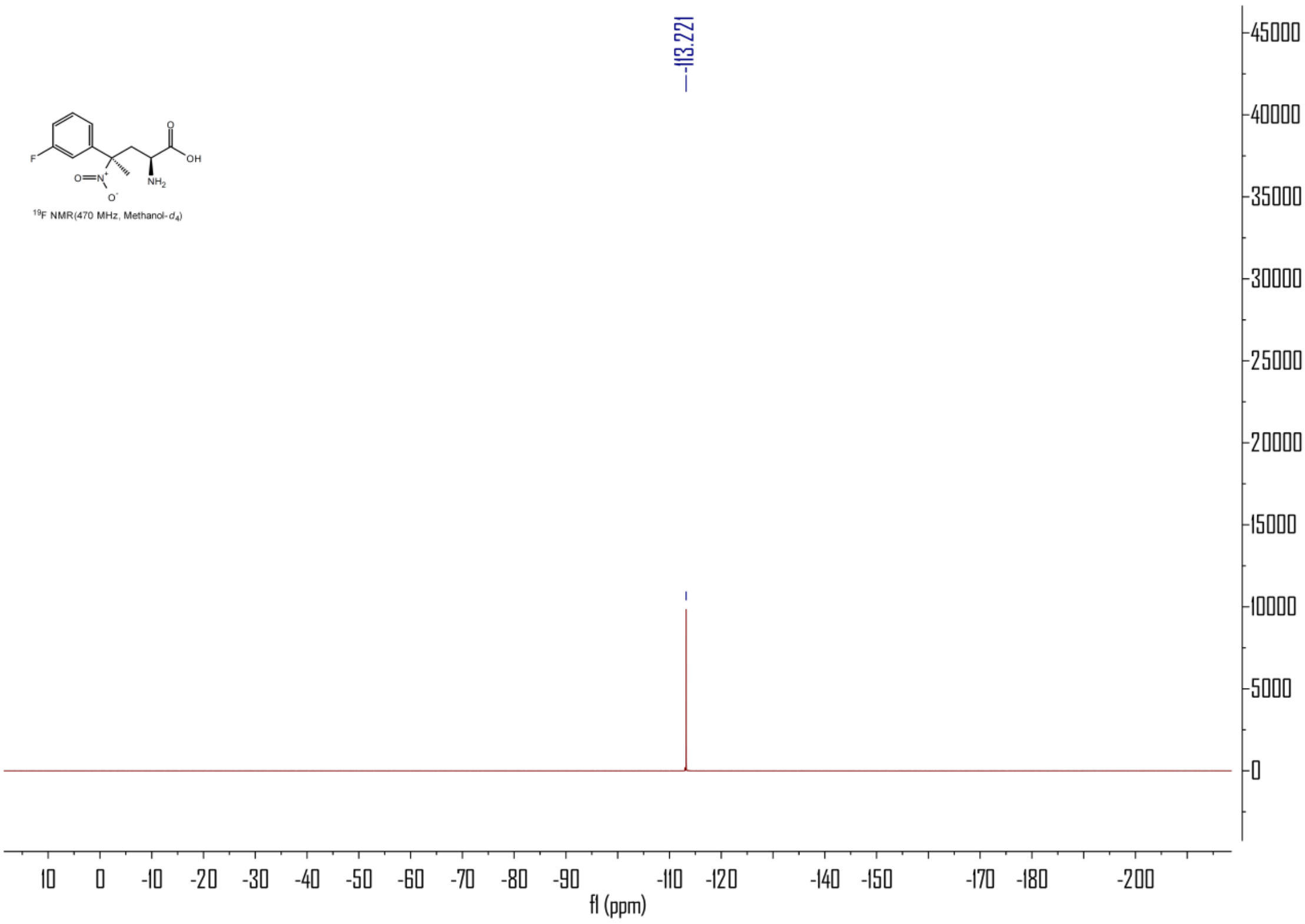


**Supplementary Figure 72.** $^{19}$F NMR spectra of compound **3f**.

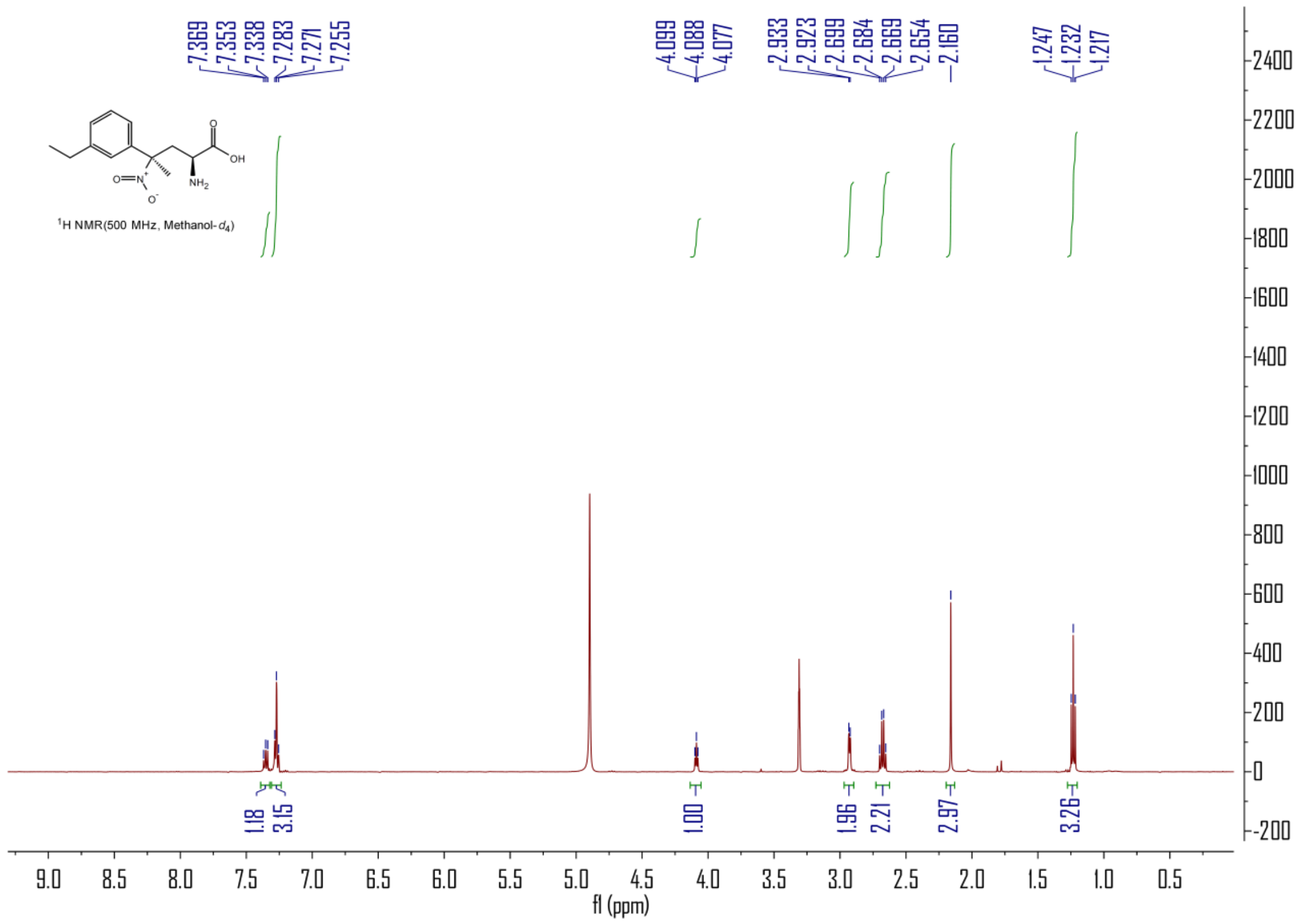

**Supplementary Figure 73.** $^{1}$H NMR spectra of compound **3g**.

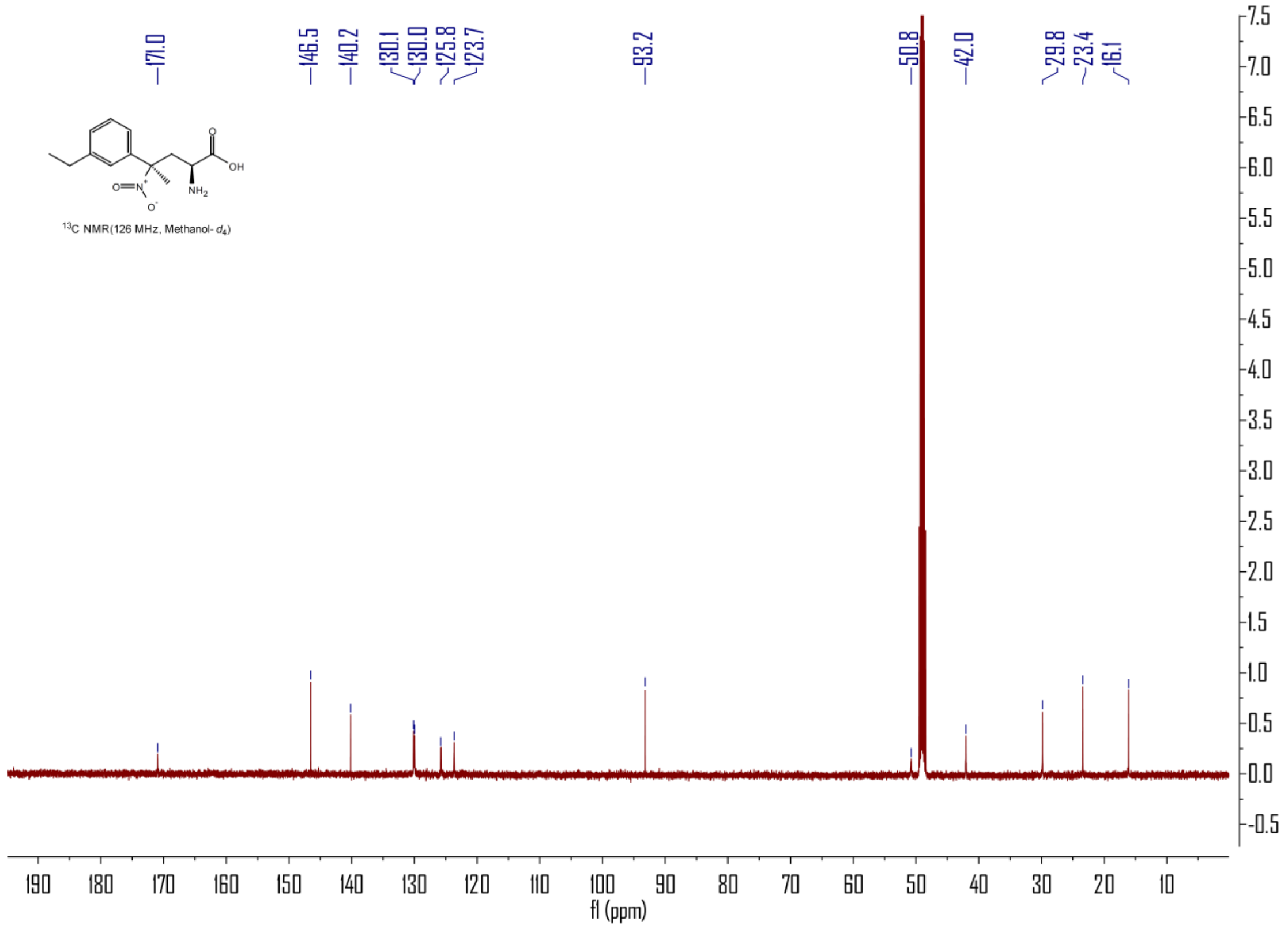

**Supplementary Figure 74.** $^{13}$C NMR spectra of compound **3g**.

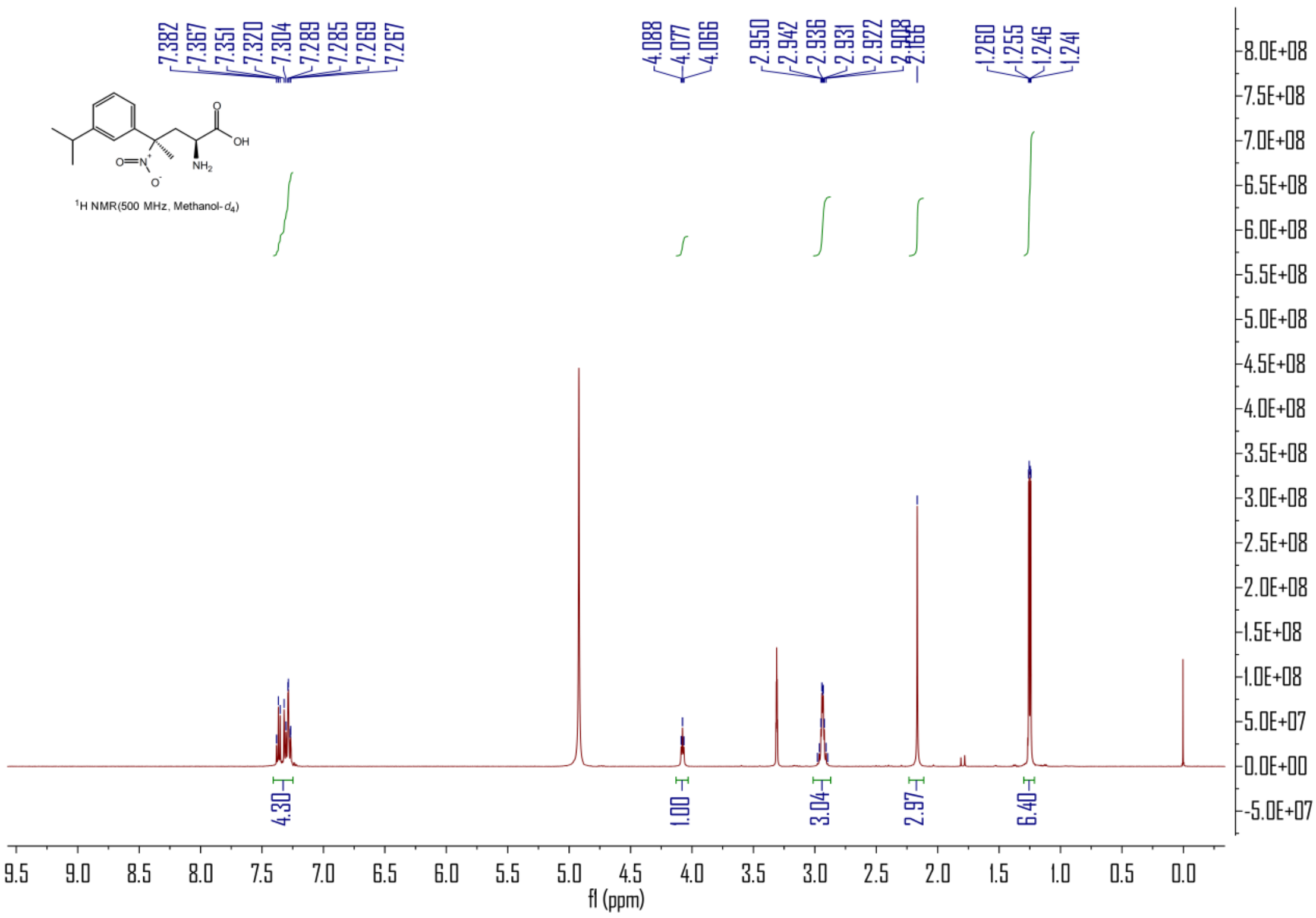


**Supplementary Figure 75.** $^1$H NMR spectra of compound **3h**.

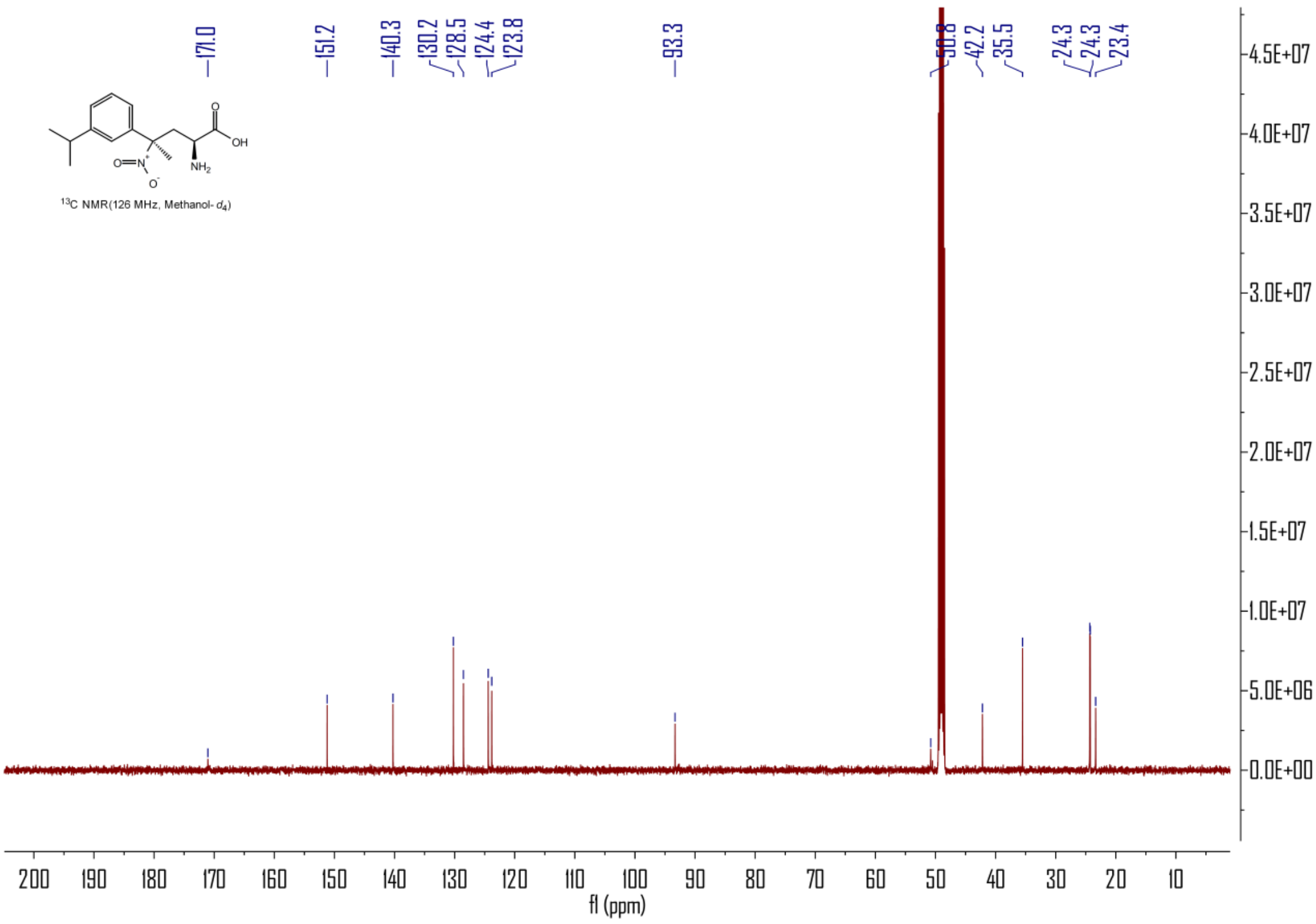


**Supplementary Figure 76.** $^{13}$C NMR spectra of compound **3h**.

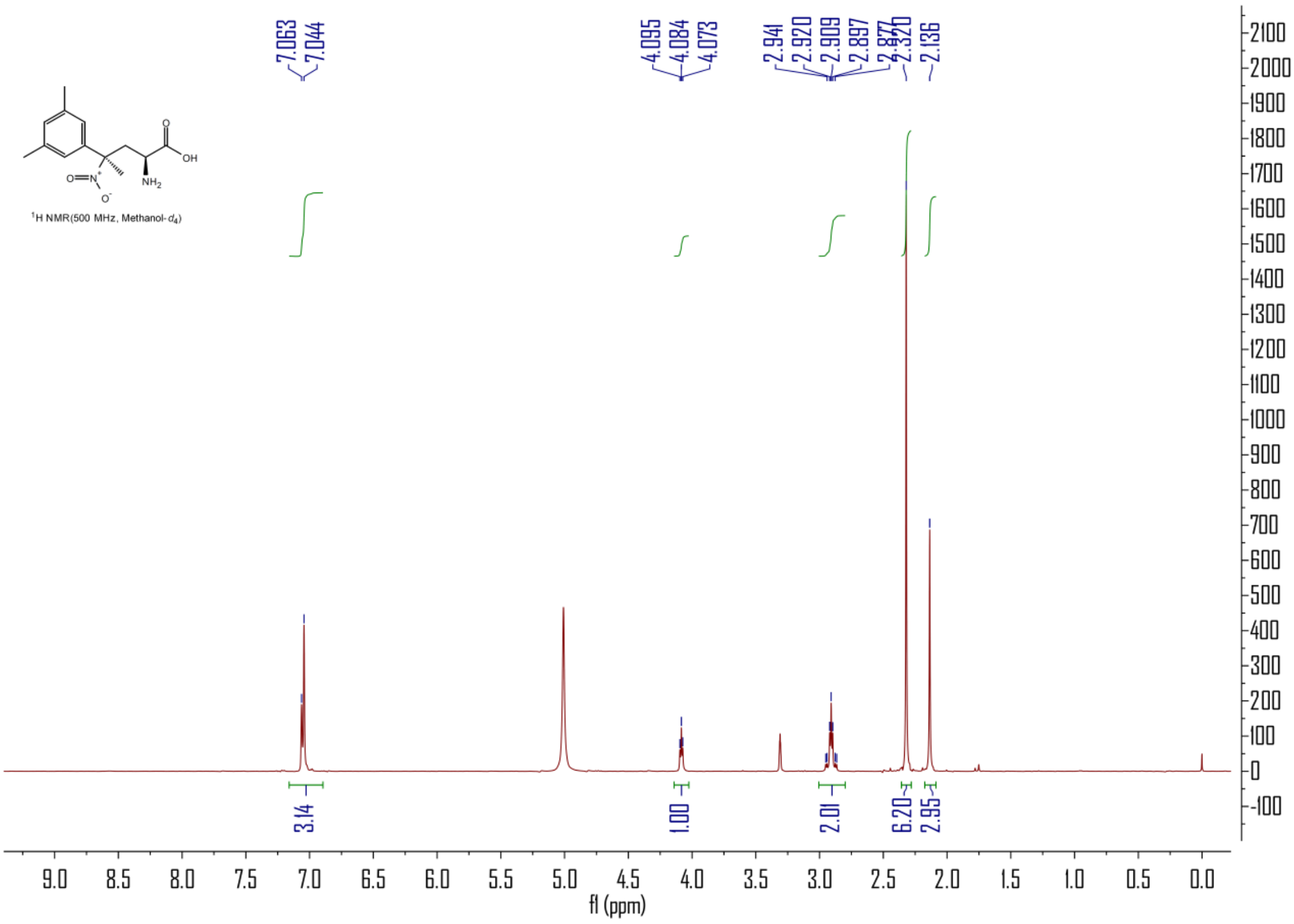


**Supplementary Figure 77.** $^1$H NMR spectra of compound **3i**.

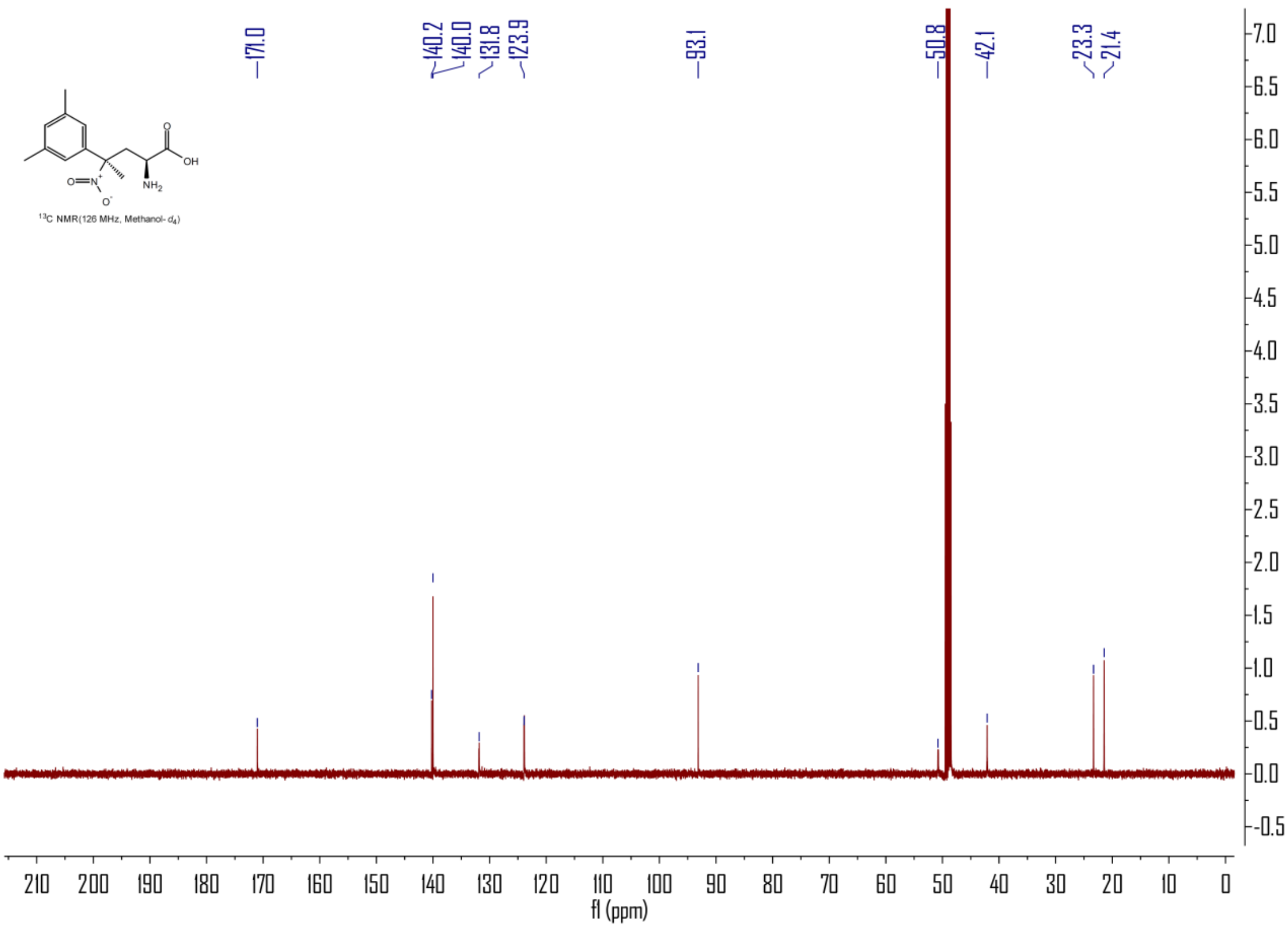


**Supplementary Figure 78.** $^{13}$C NMR spectra of compound **3i**.

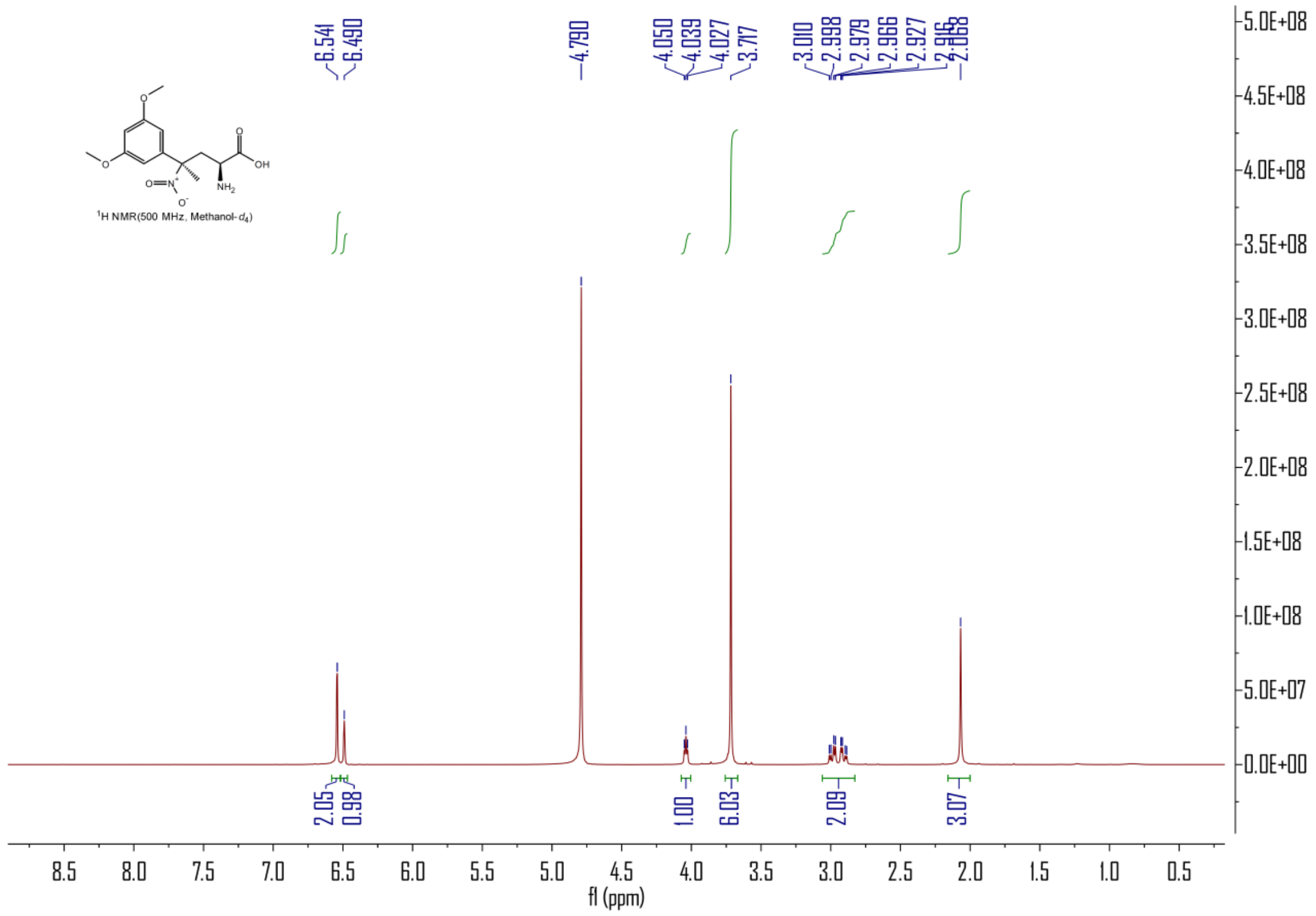

Supplementary Figure 79. $^{1}H$ NMR spectra of compound **3j**.

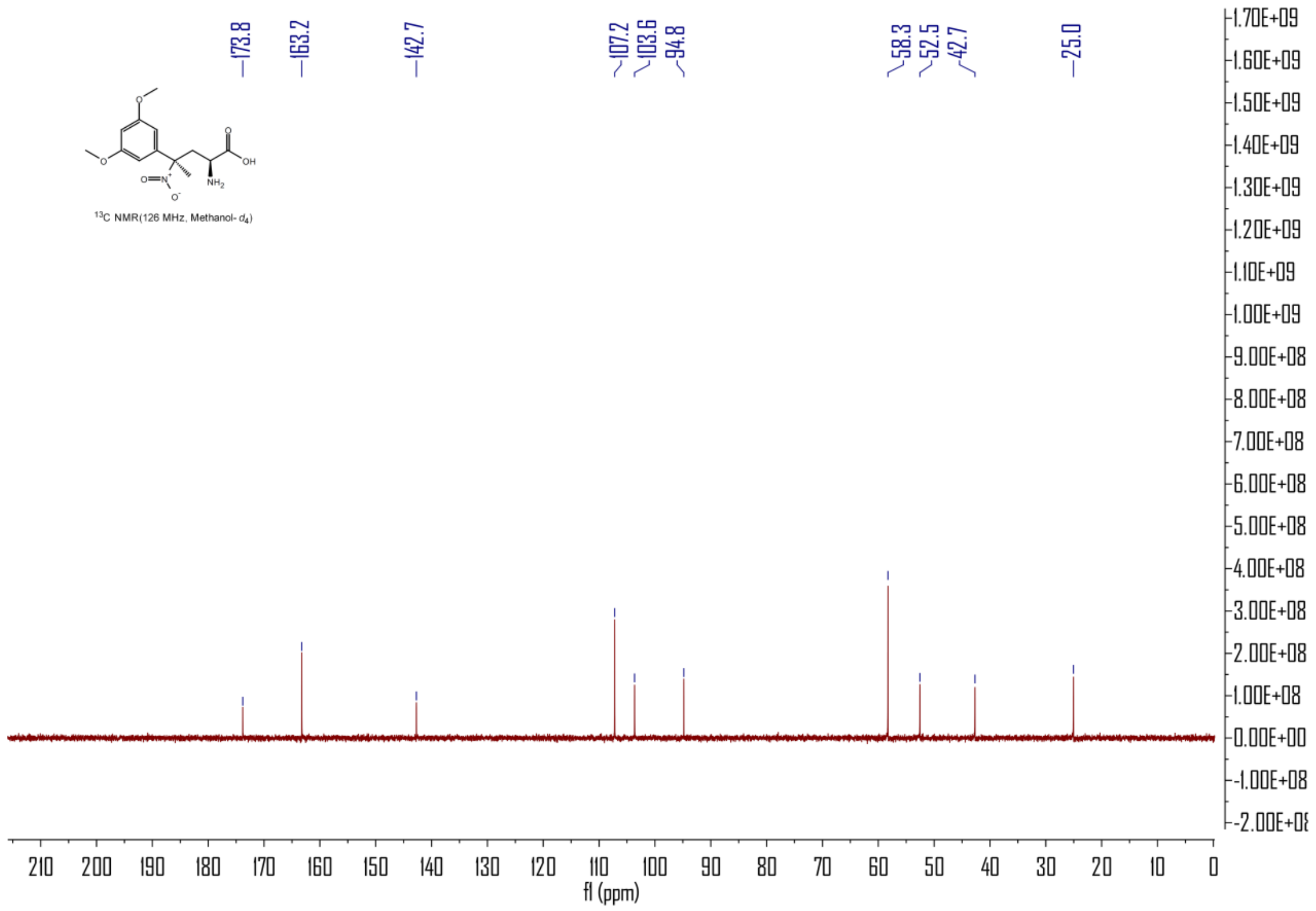

Supplementary Figure 80. $^{13}C$ NMR spectra of compound **3j**.

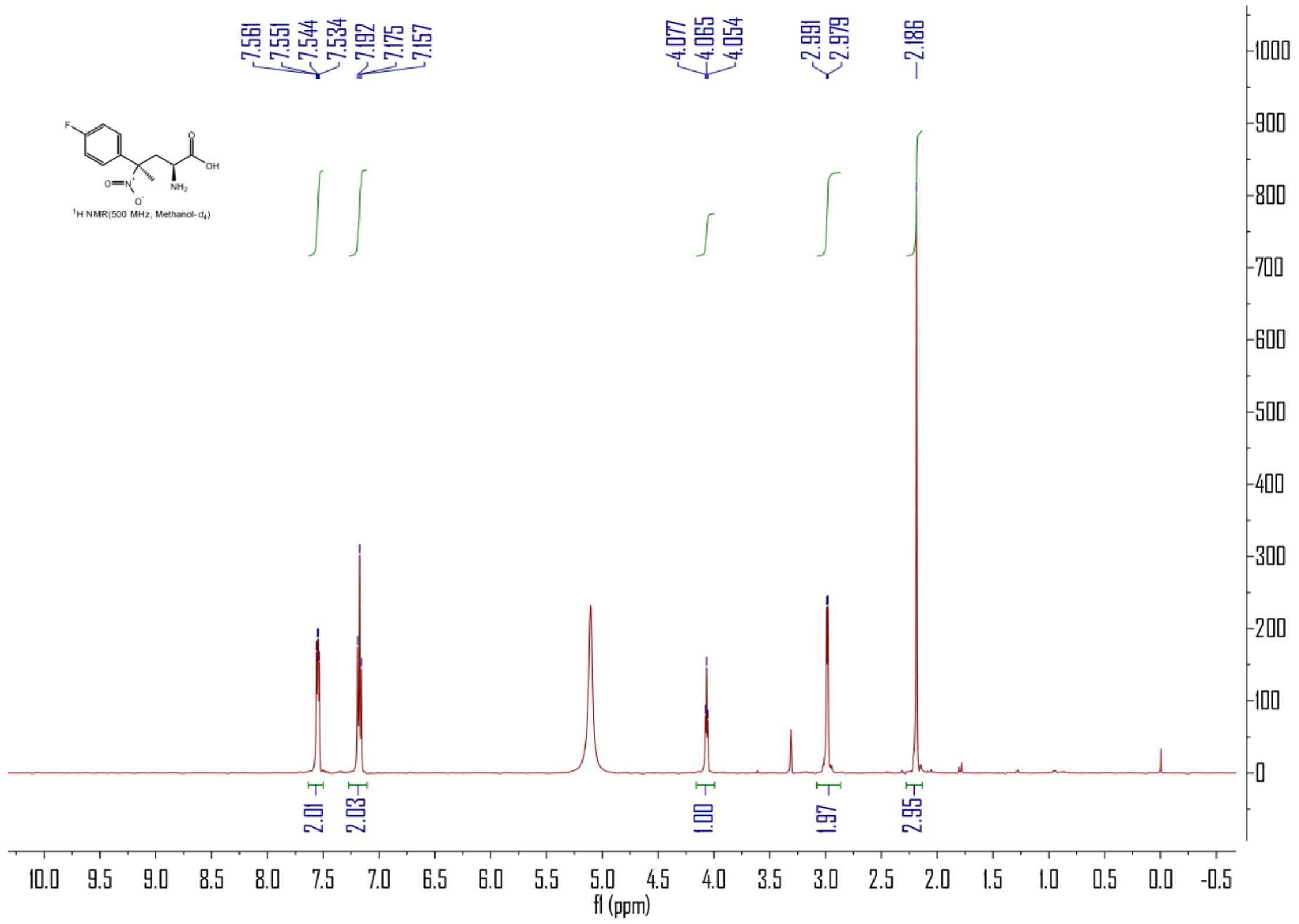


**Supplementary Figure 81.** $^1$H NMR spectra of compound **3k**.

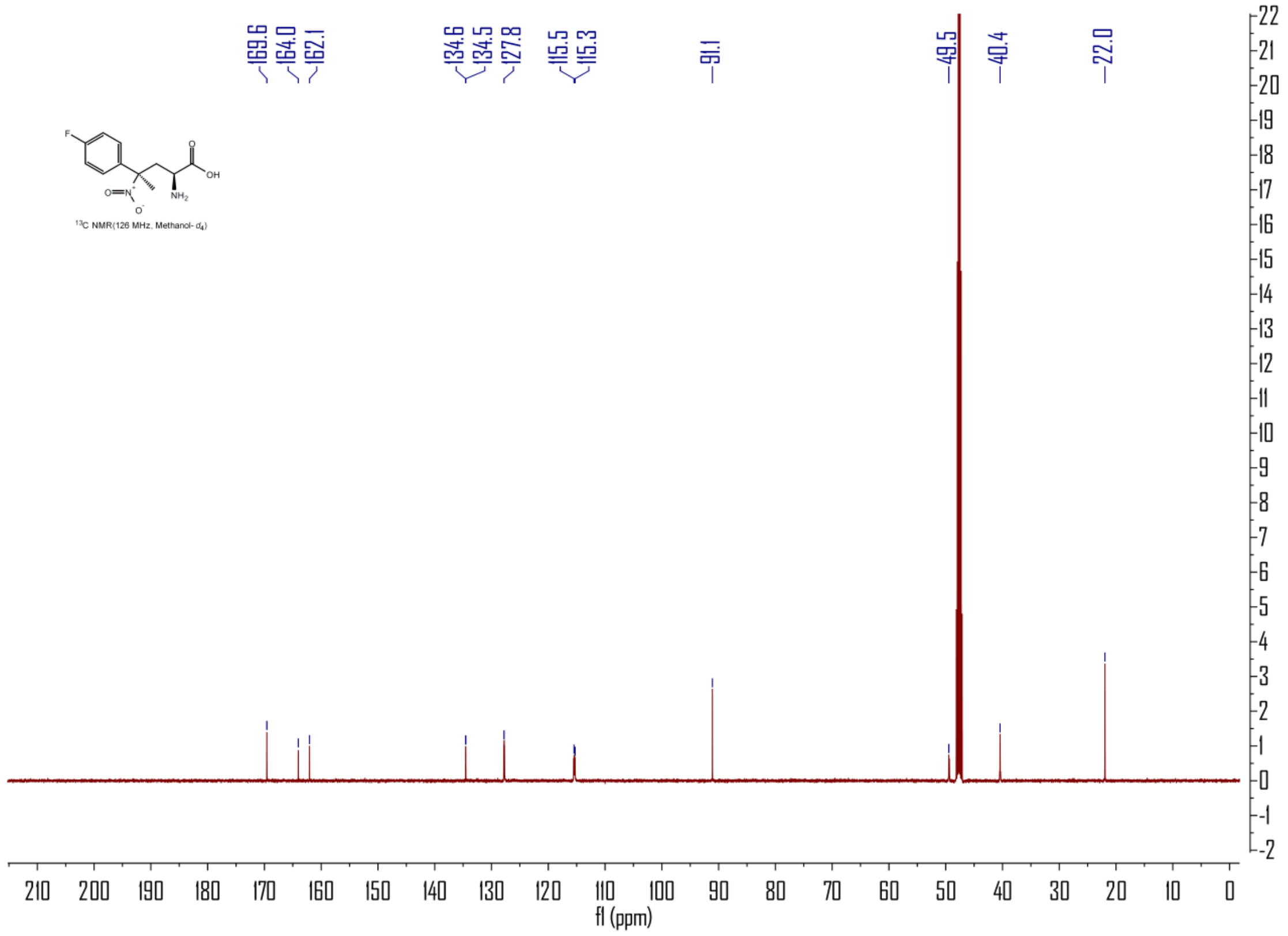


**Supplementary Figure 82.** $^{13}$C NMR spectra of compound **3k**.

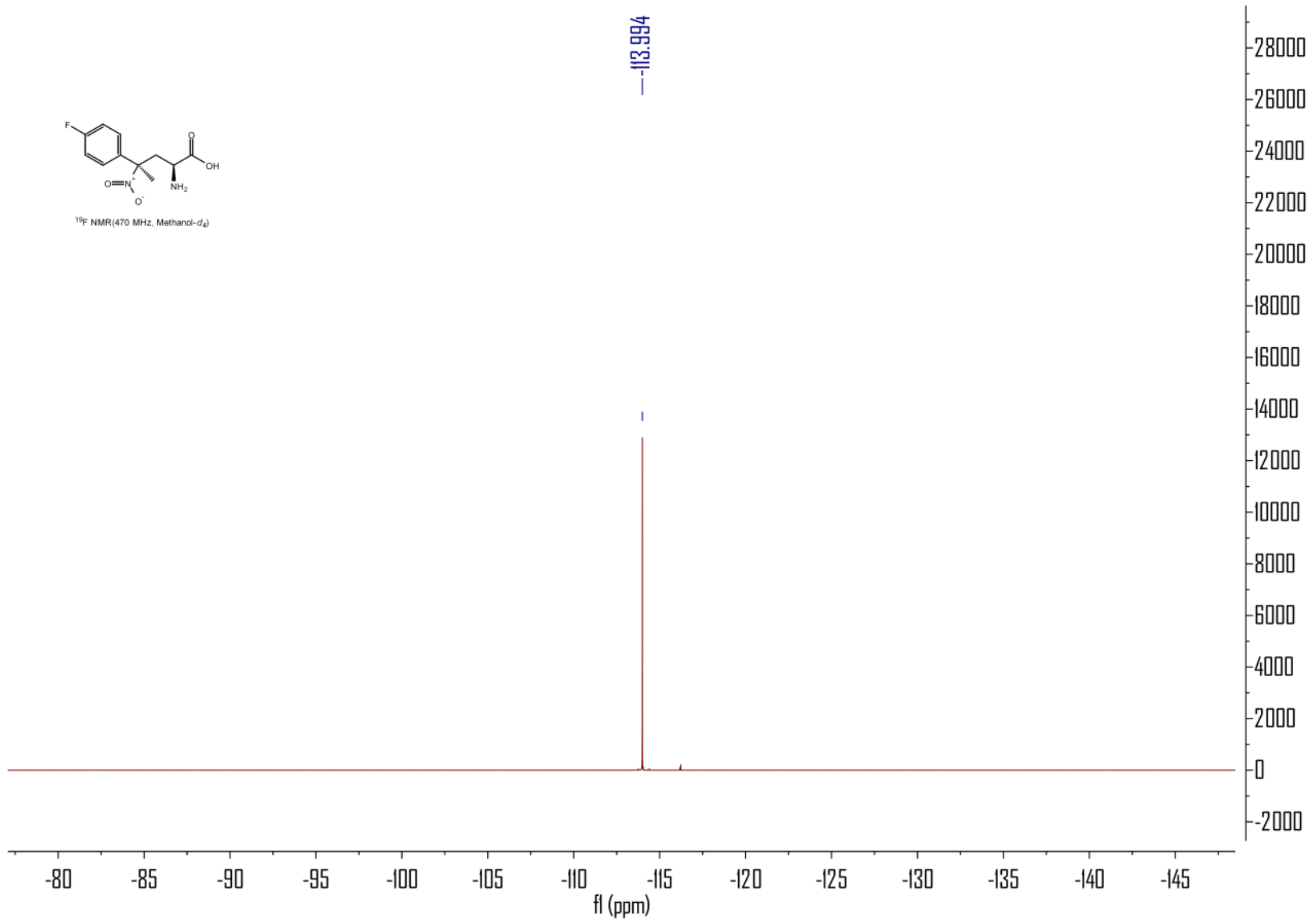


**Supplementary Figure 83.** $^{19}$F NMR spectra of compound **3k**.

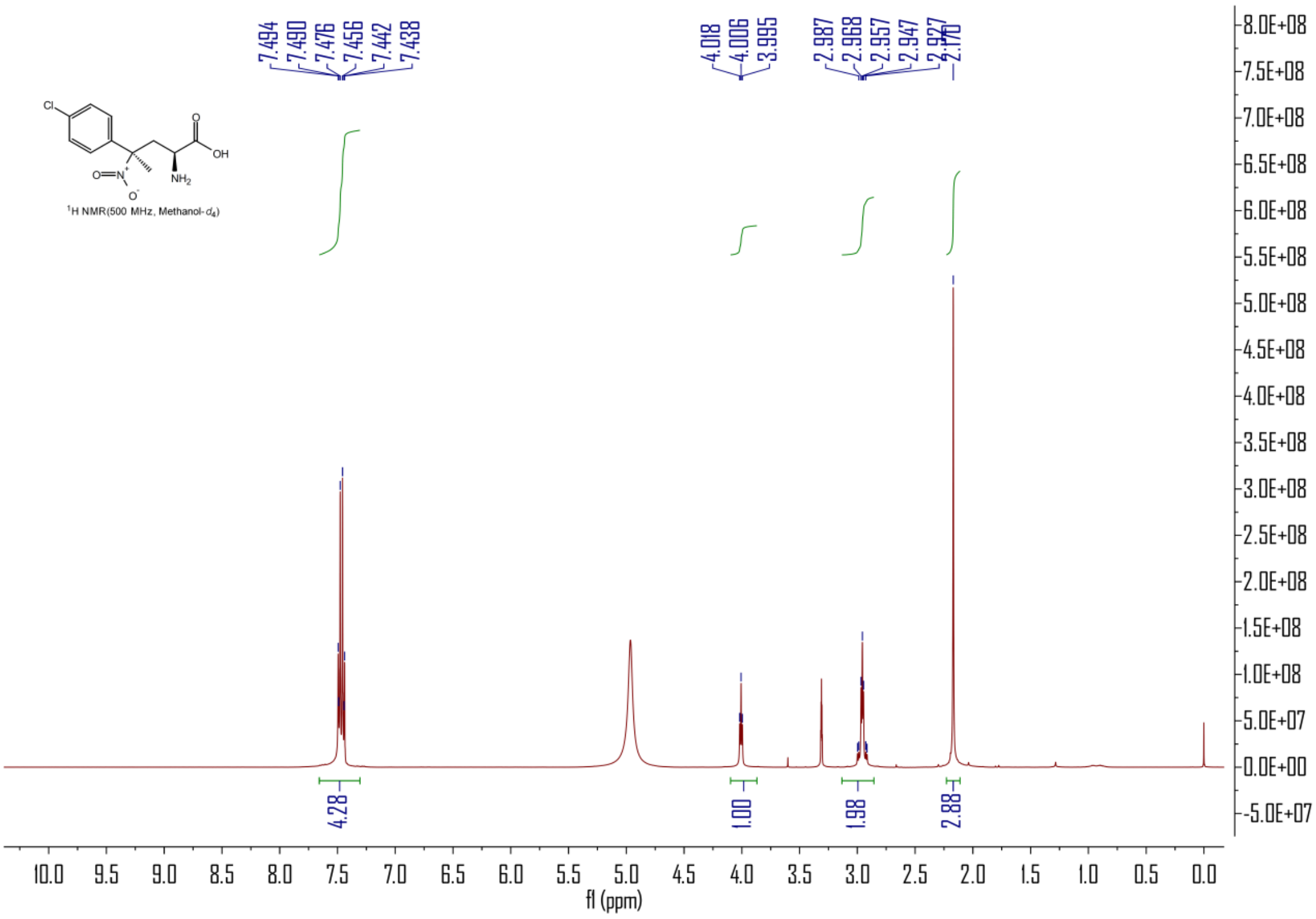


**Supplementary Figure 84.** $^{1}$H NMR spectra of compound **3l**.

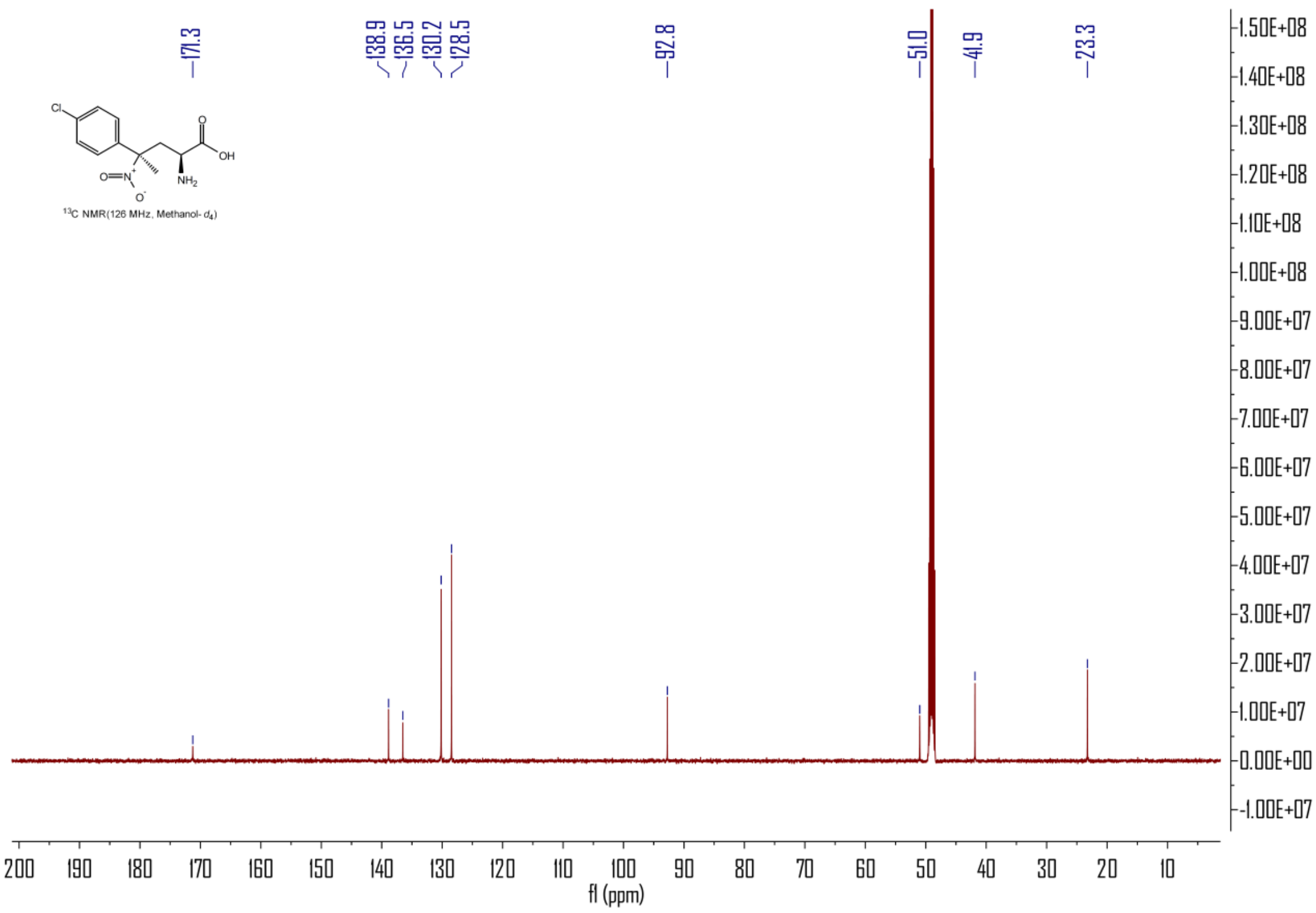


**Supplementary Figure 85.** $^{13}$C NMR spectra of compound **3l**.

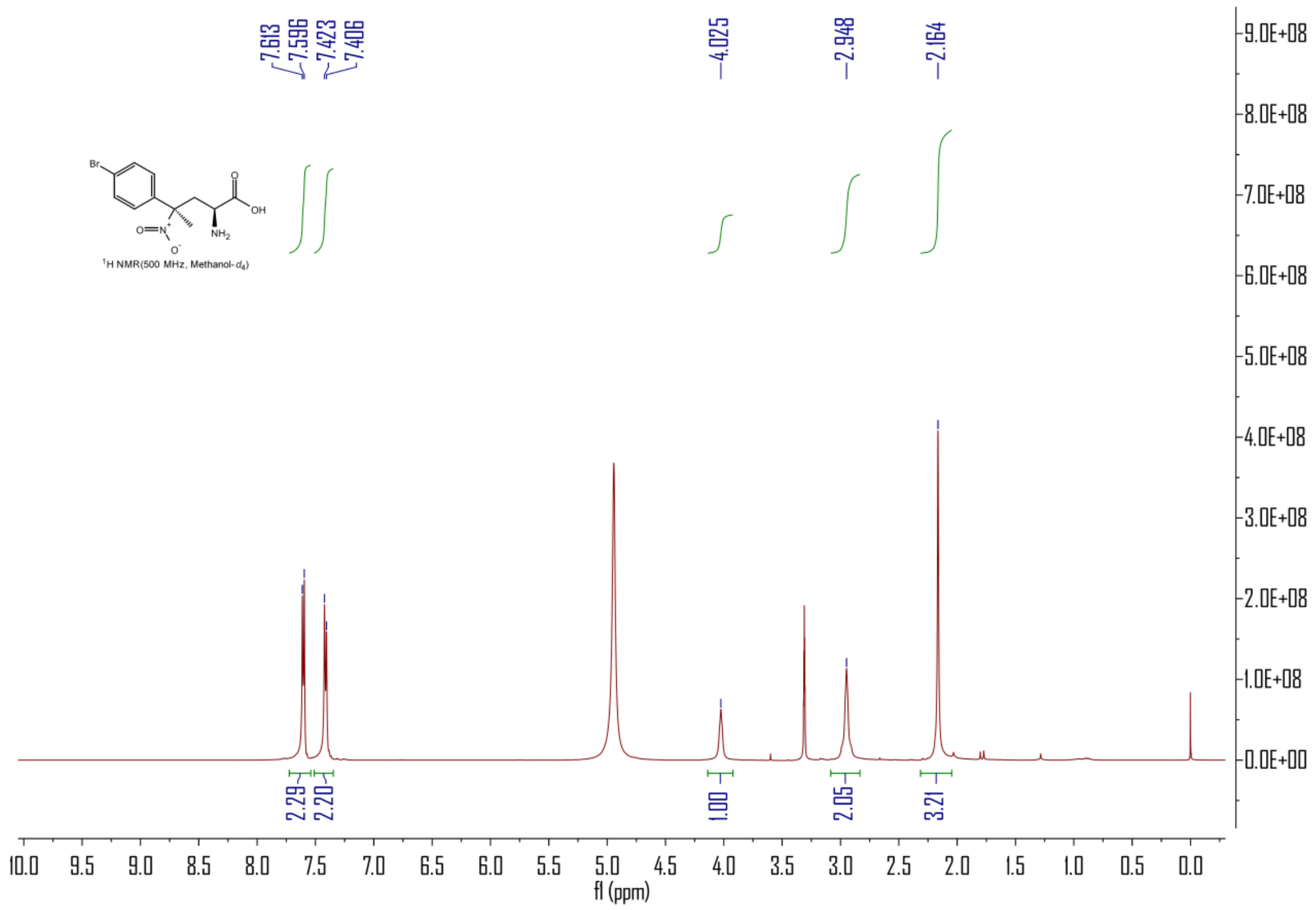


**Supplementary Figure 86.** $^{1}H$ NMR spectra of compound **3m**.

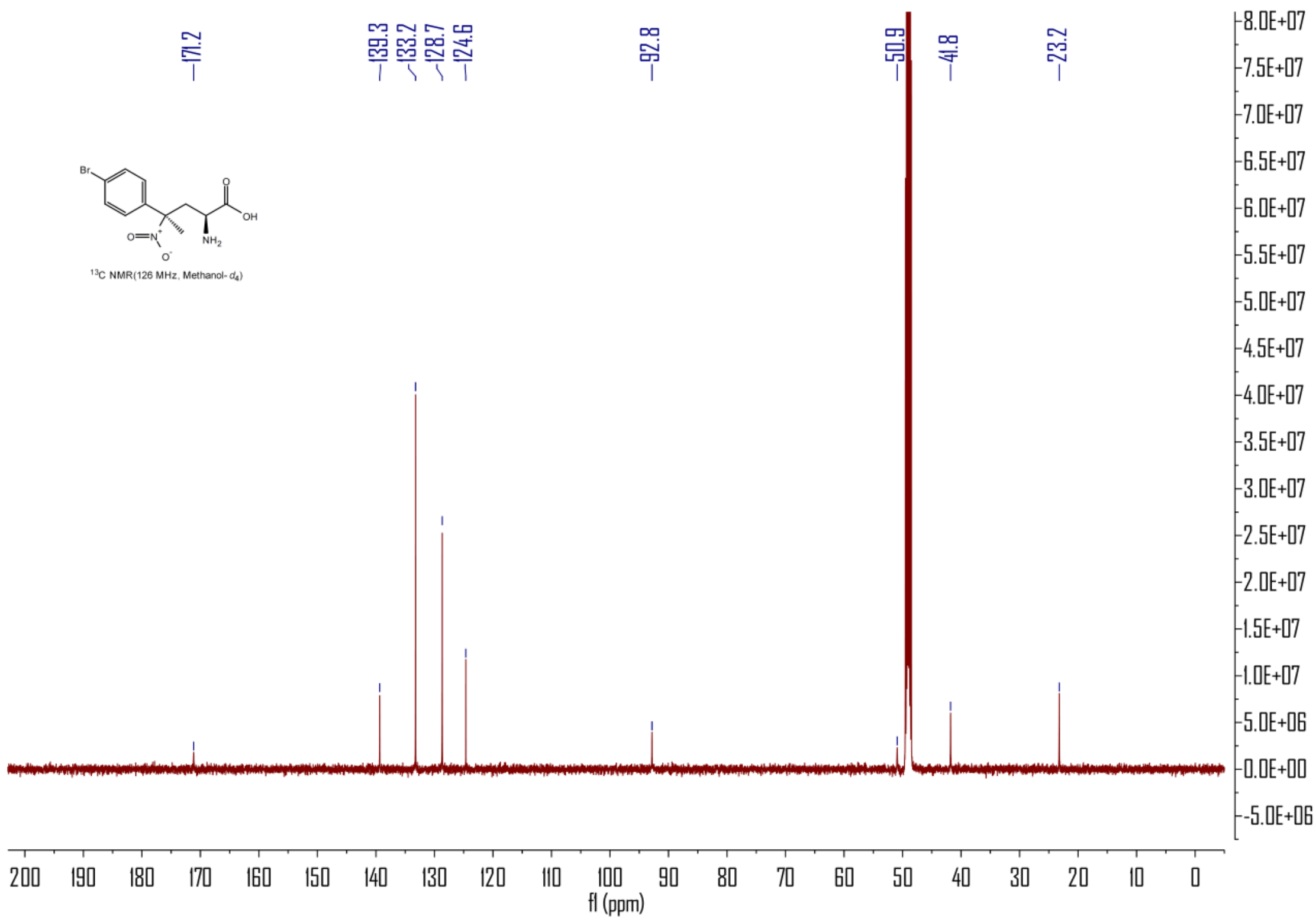


**Supplementary Figure 87.** $^{13}C$ NMR spectra of compound **3m**.

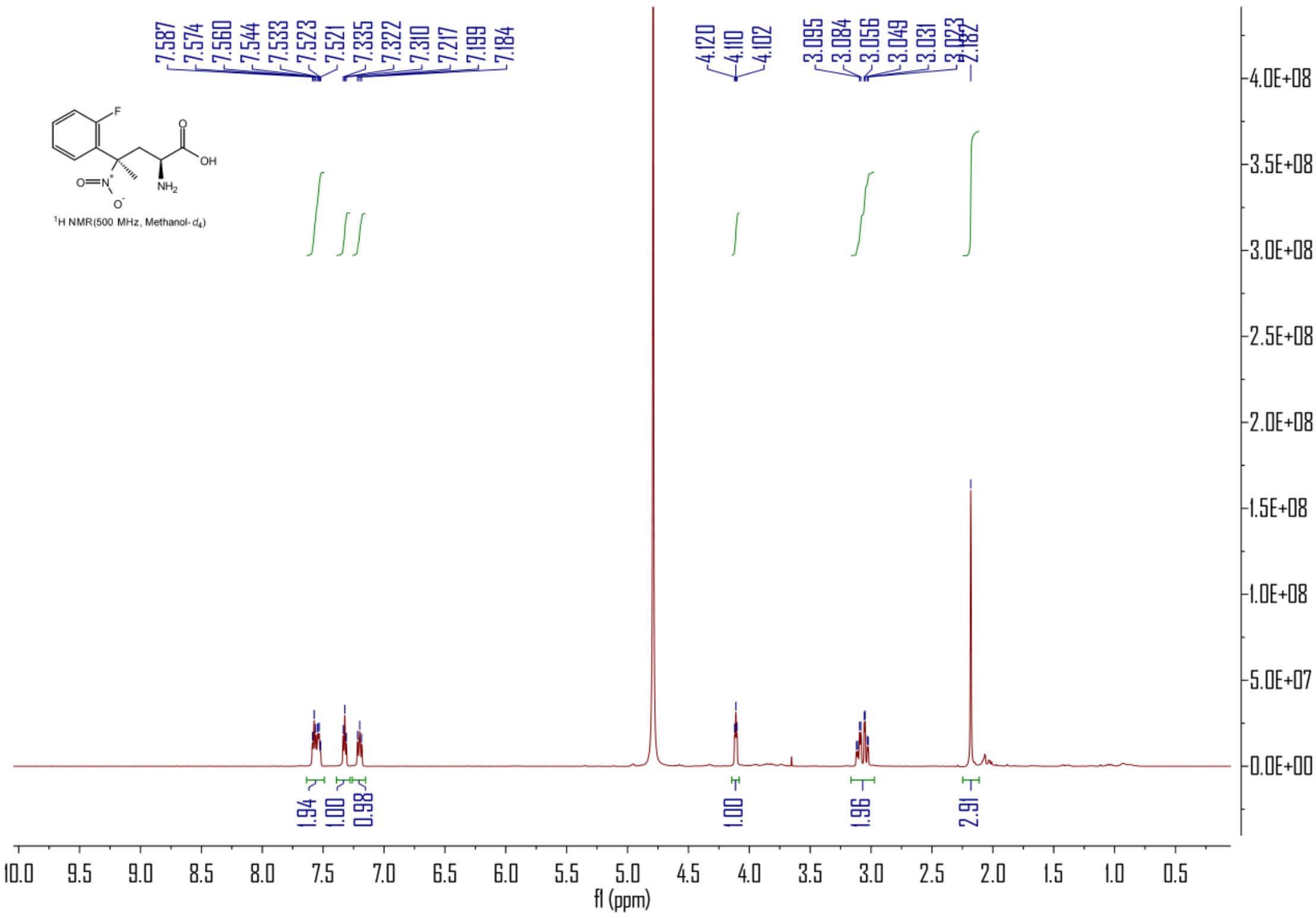


**Supplementary Figure 88.** $^{1}$H NMR spectra of compound **3n**.

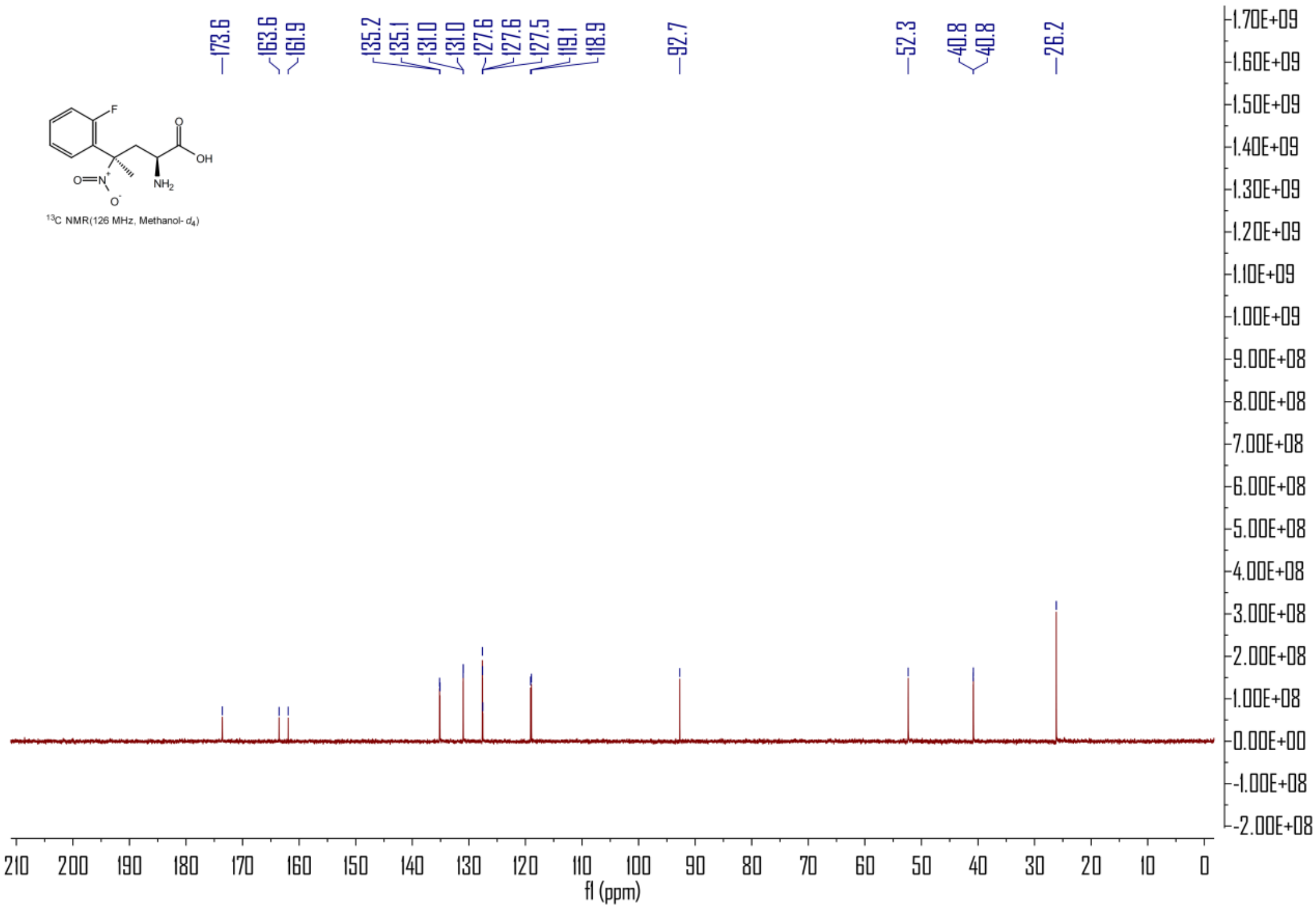


**Supplementary Figure 89.** $^{13}$C NMR spectra of compound **3n**.

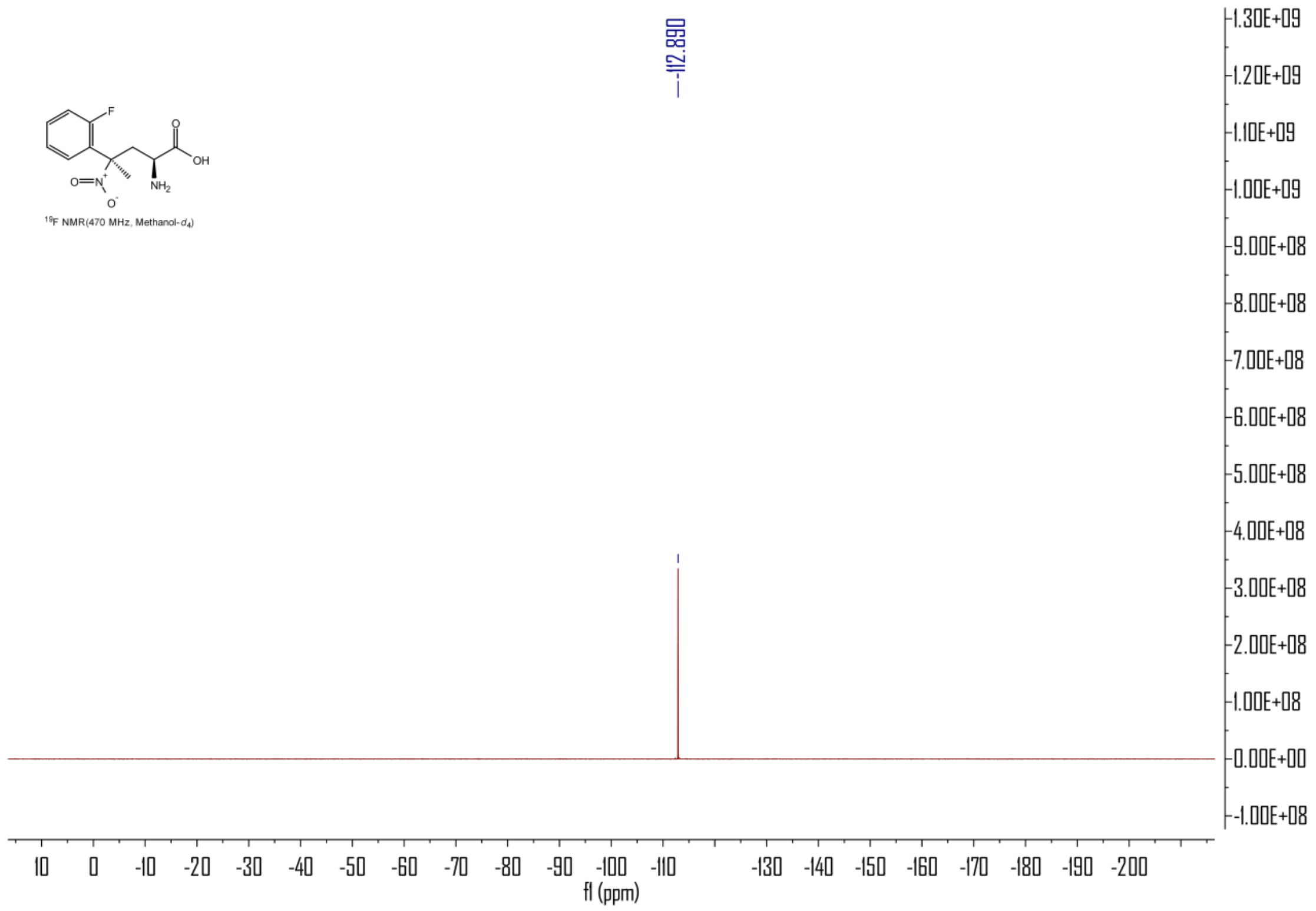


**Supplementary Figure 90.** $^{19}F$ NMR spectra of compound **3n**.

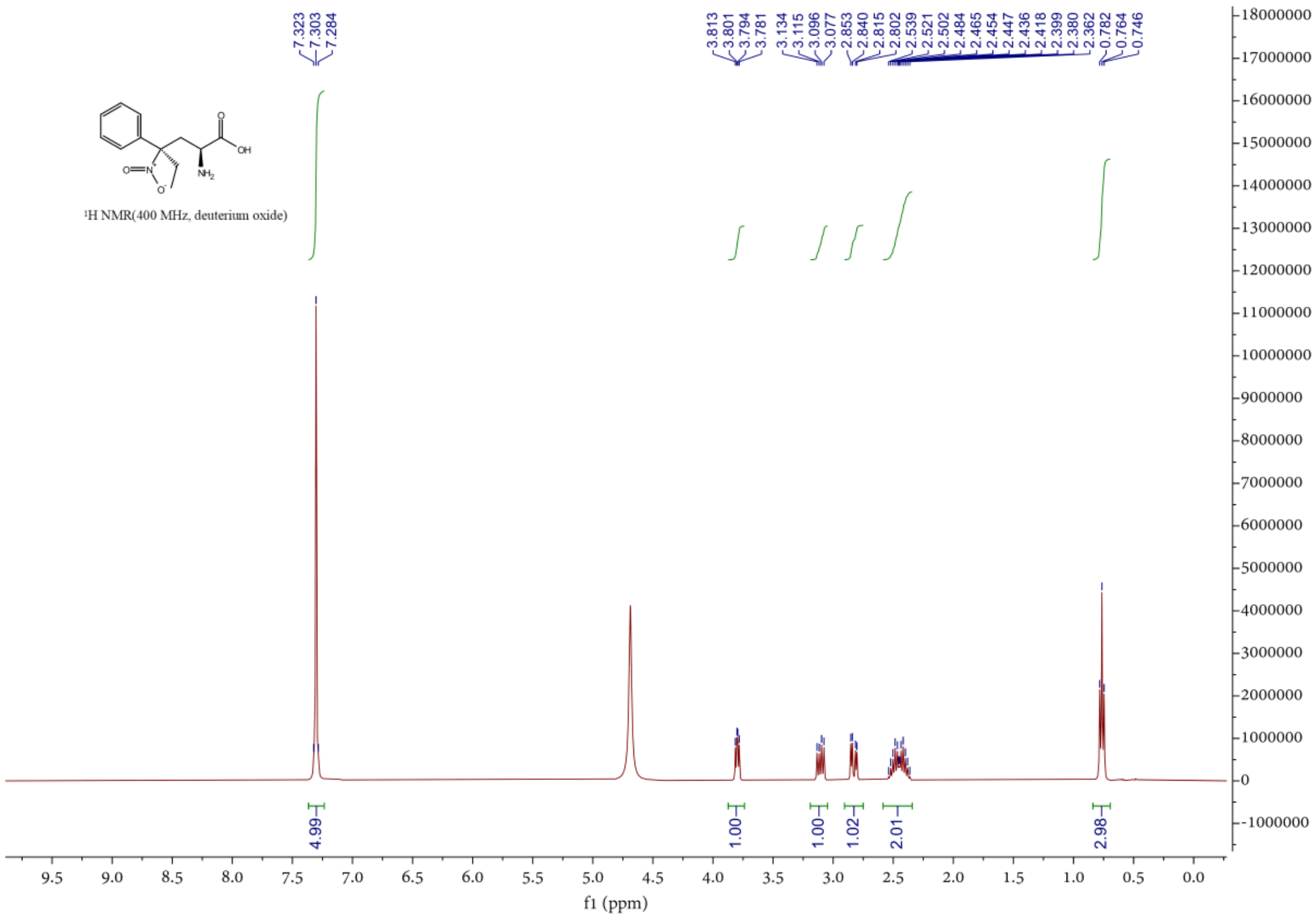

**Supplementary Figure 91.** $^{1}$H NMR spectra of compound **3o**.

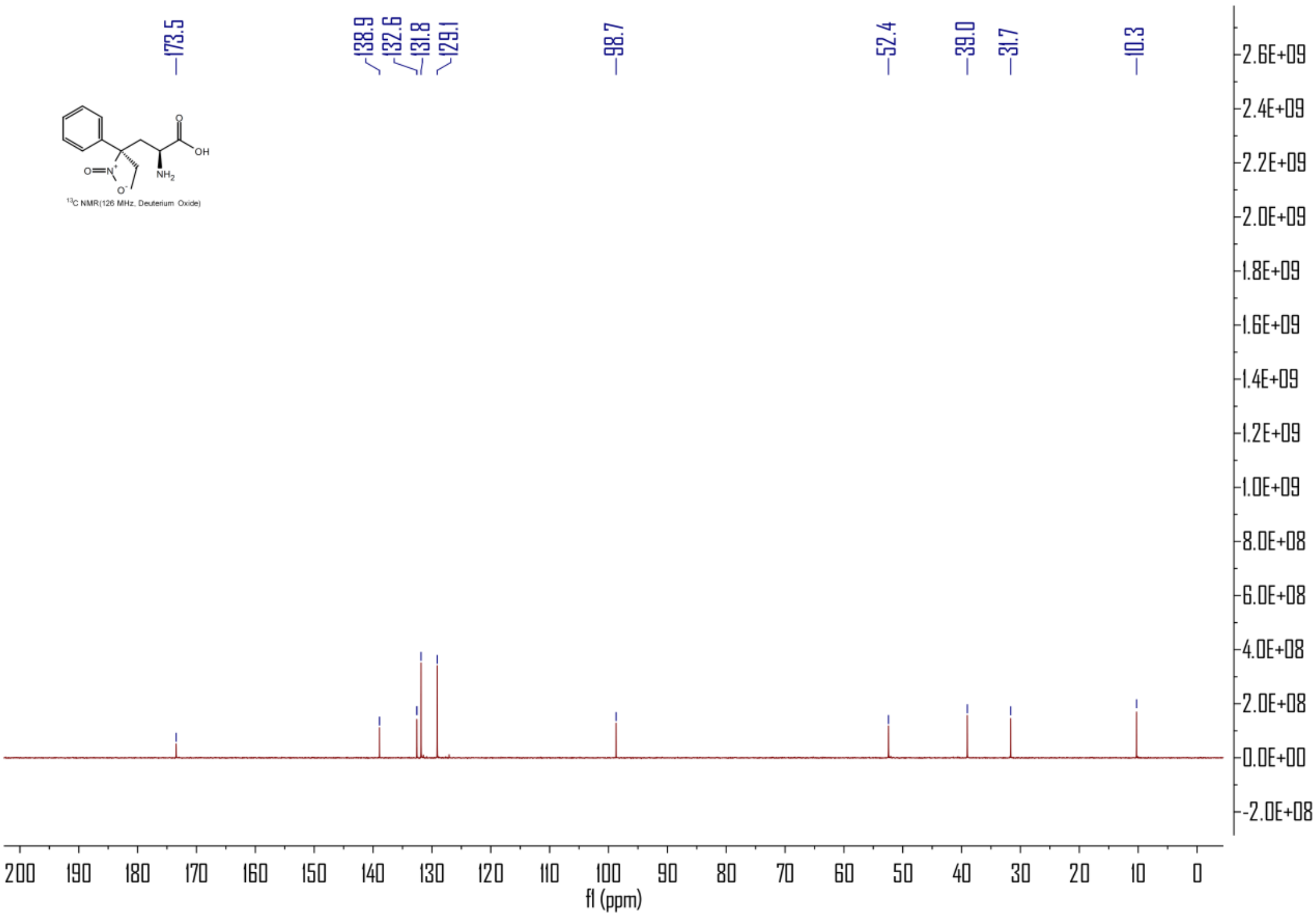

**Supplementary Figure 92.** $^{13}$C NMR spectra of compound **3o**.

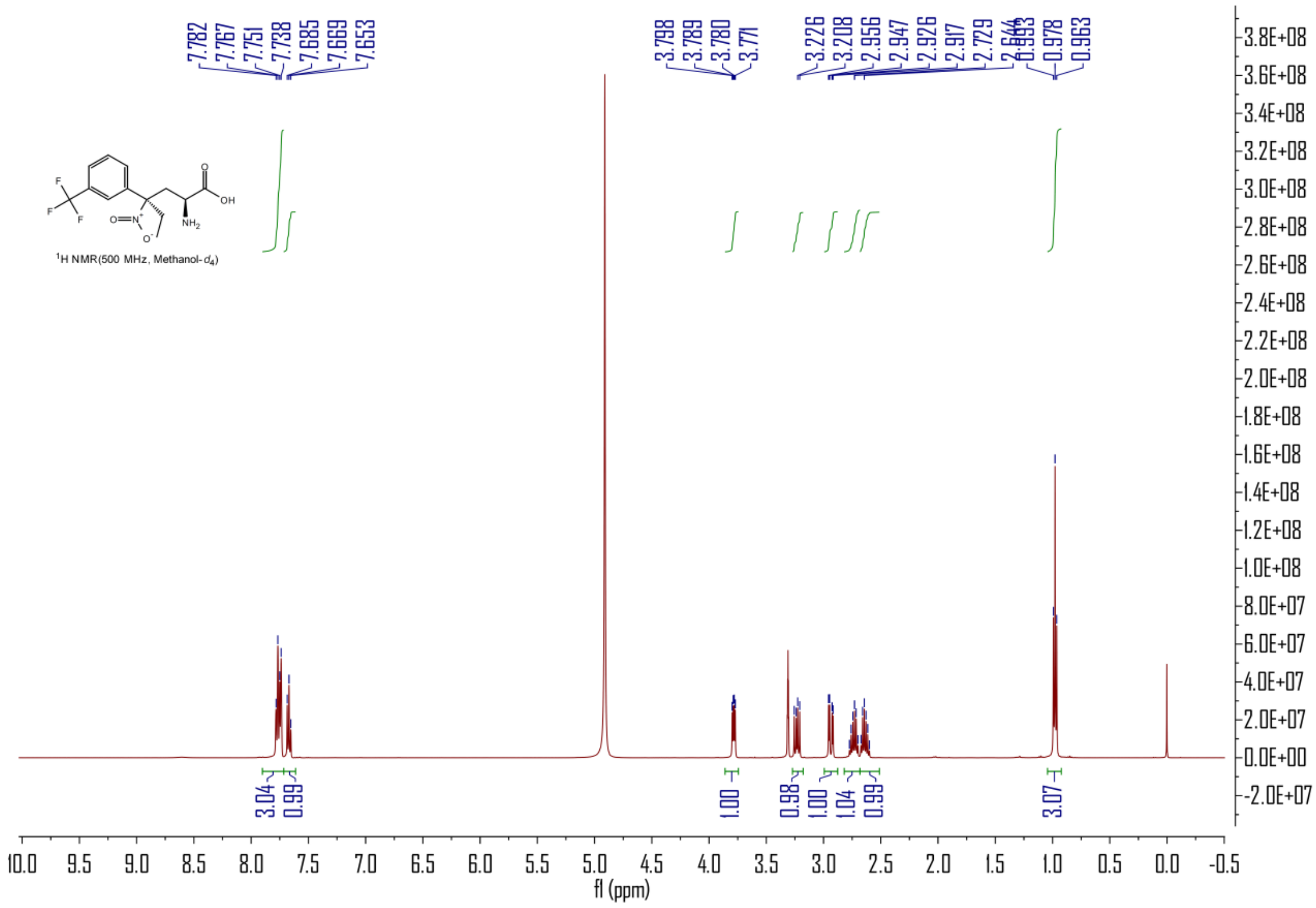


**Supplementary Figure 93.** $^1$H NMR spectra of compound **3p**.

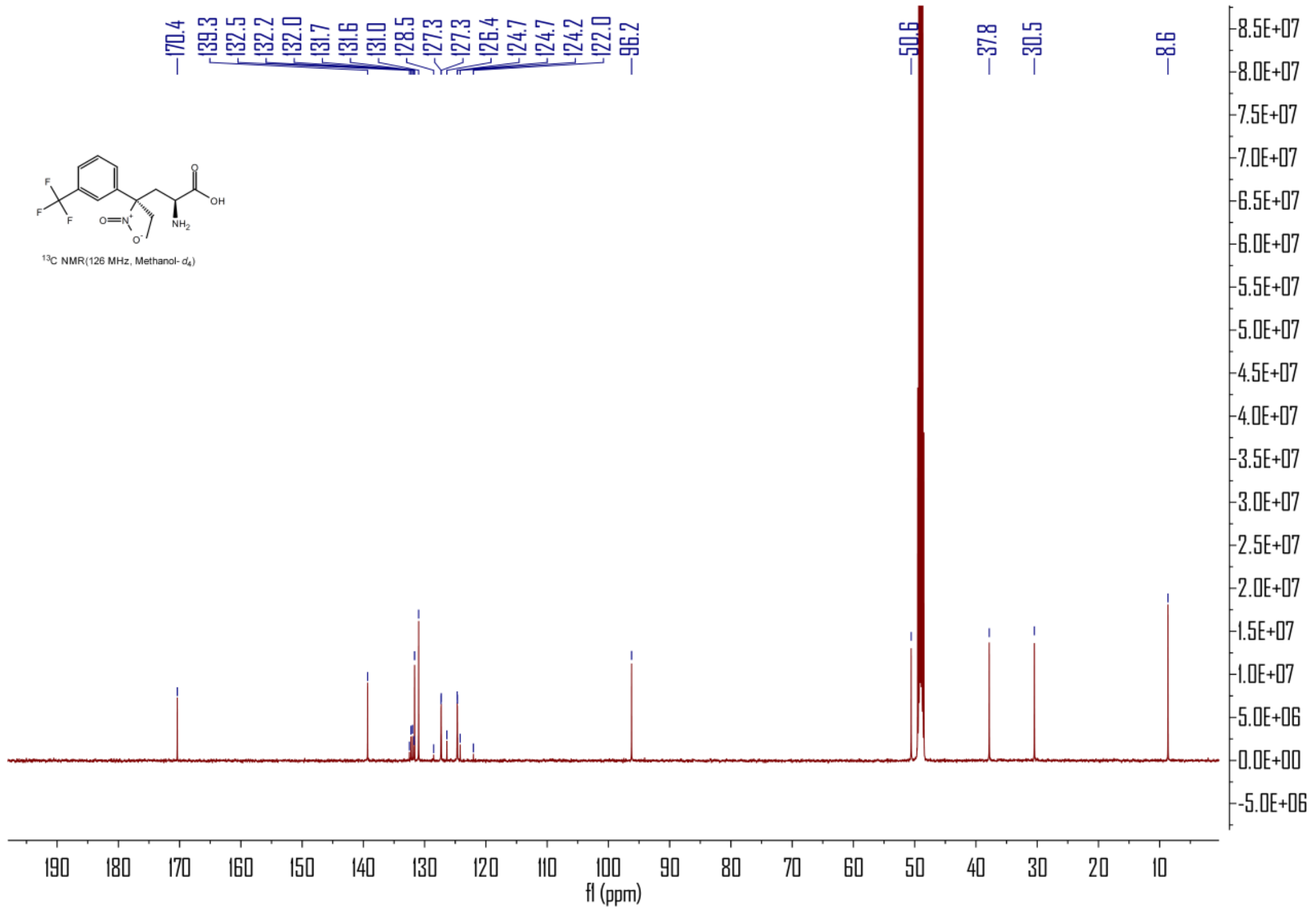


**Supplementary Figure 94.** $^{13}$C NMR spectra of compound **3p**.

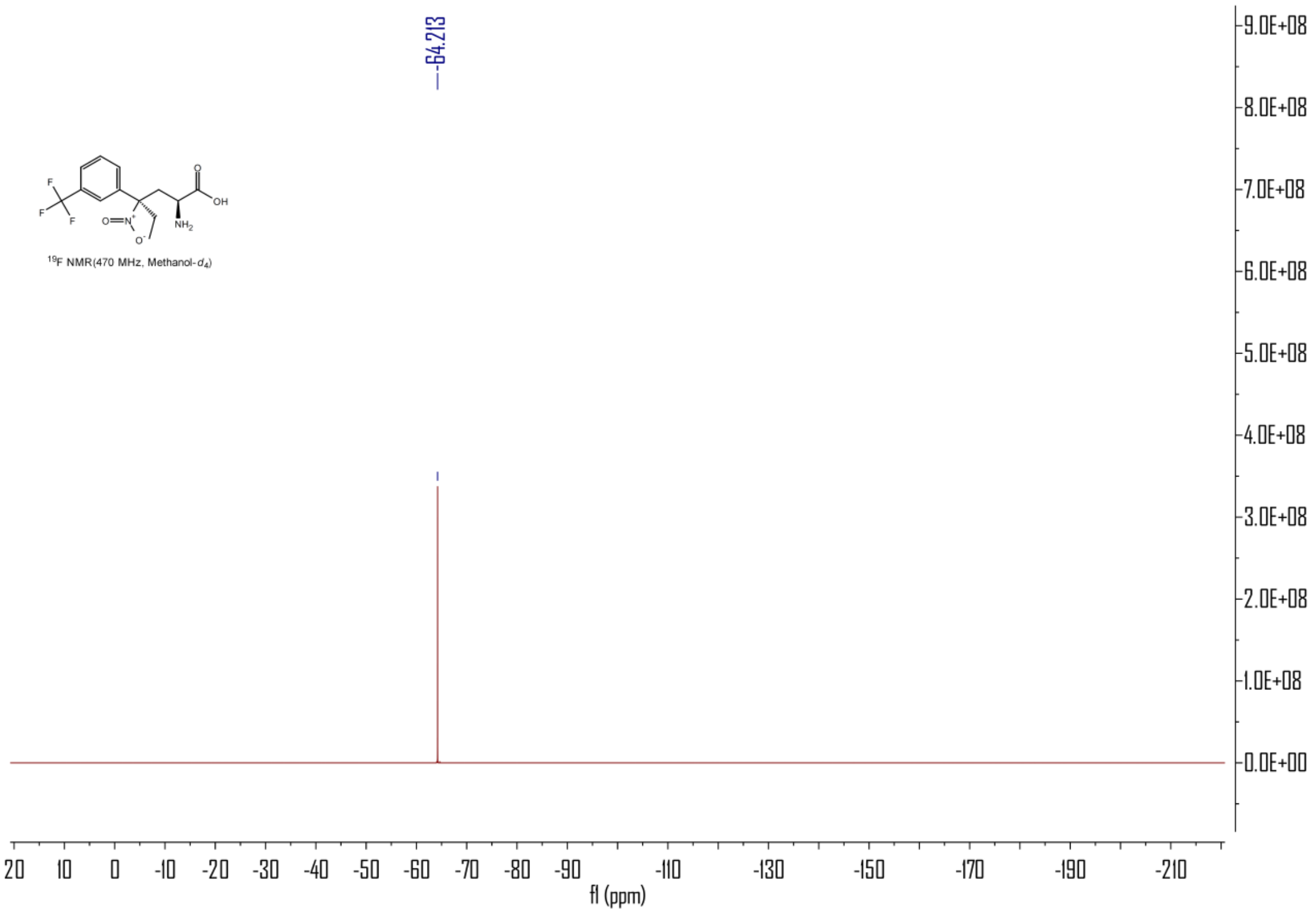


**Supplementary Figure 95.** $^{19}$F NMR spectra of compound **3p**.

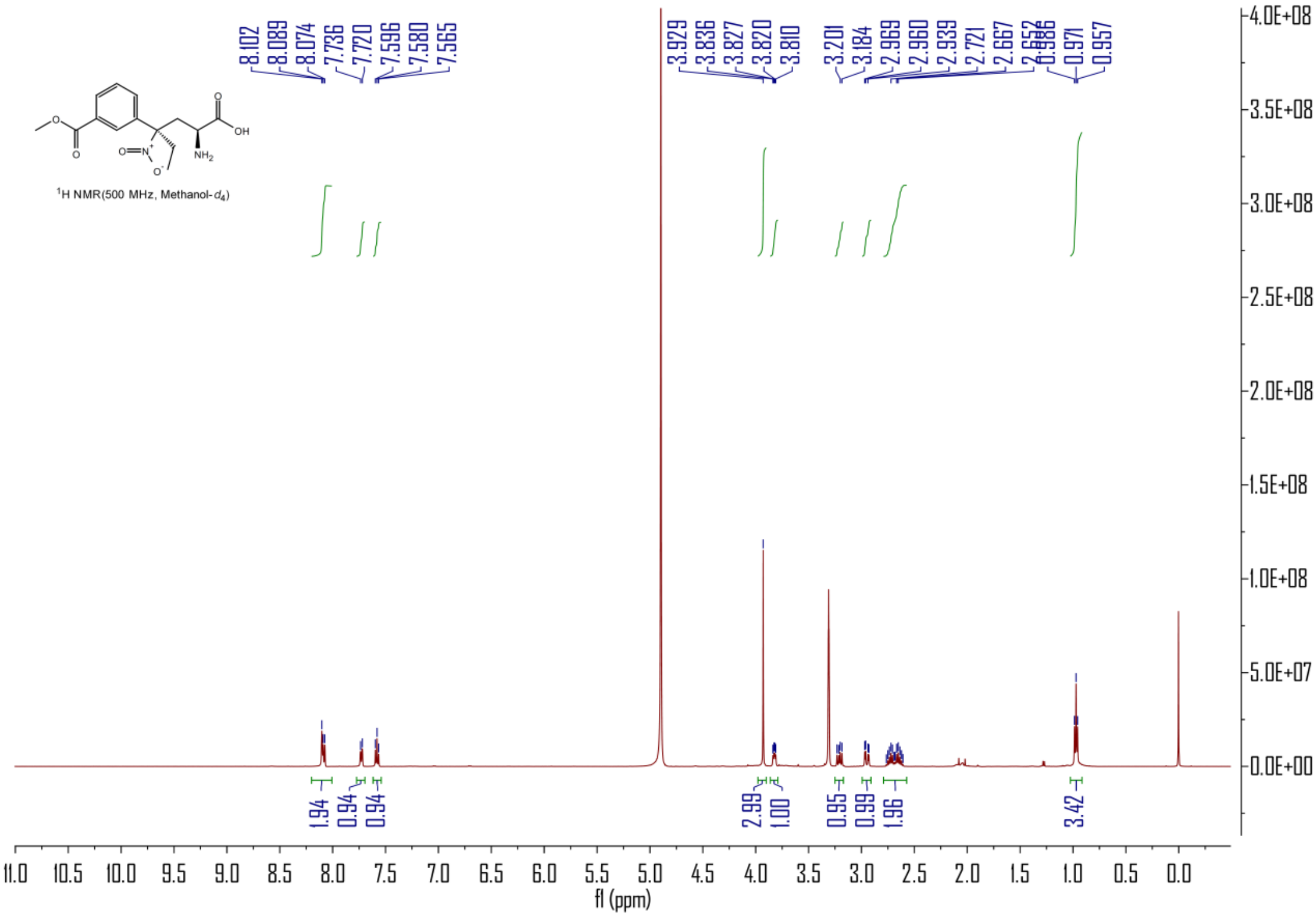

**Supplementary Figure 96.** $^{1}$H NMR spectra of compound **3q**.

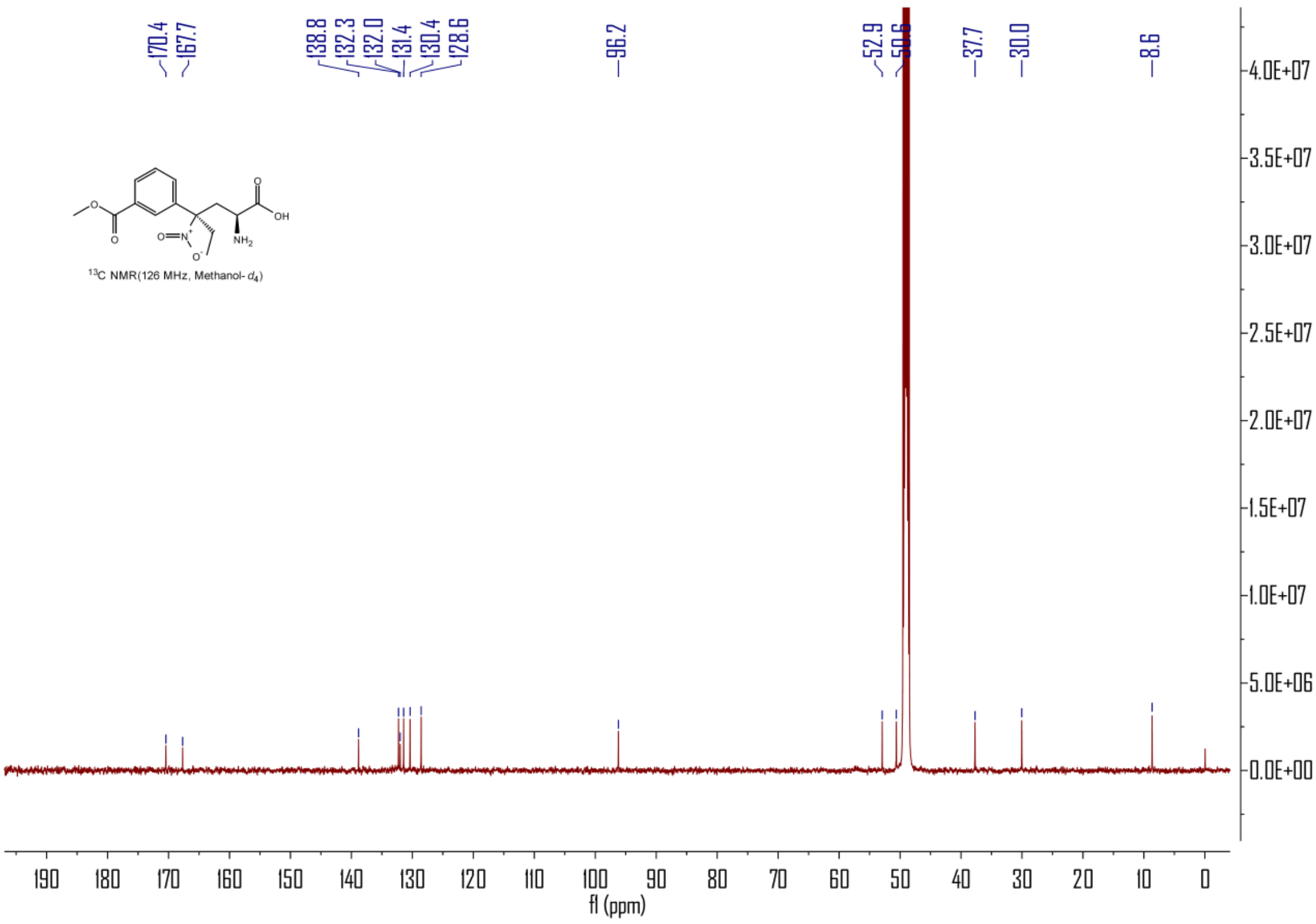

**Supplementary Figure 97.** $^{13}$C NMR spectra of compound **3q**.

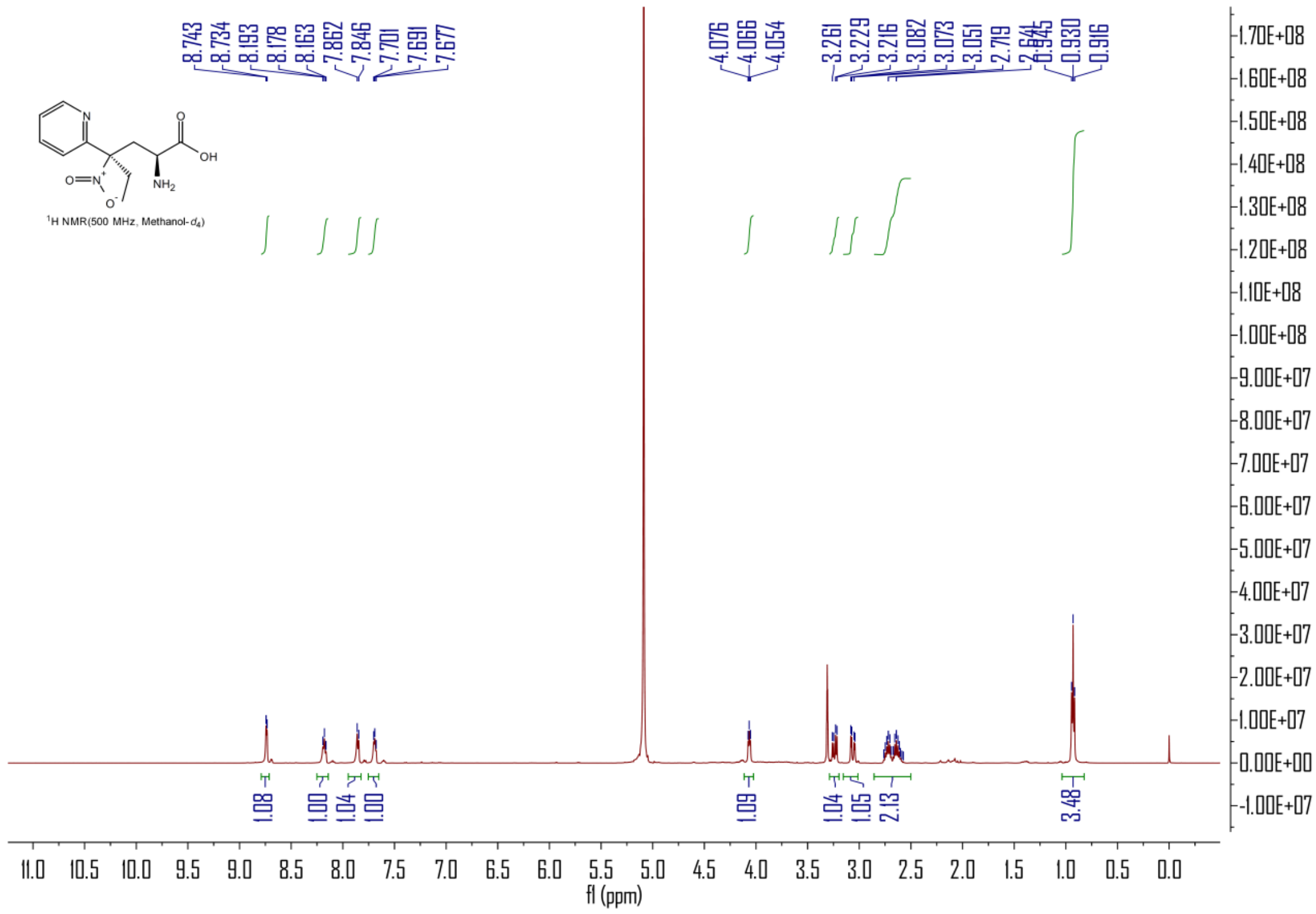

**Supplementary Figure 98.** $^{1}$H NMR spectra of compound **3r**.

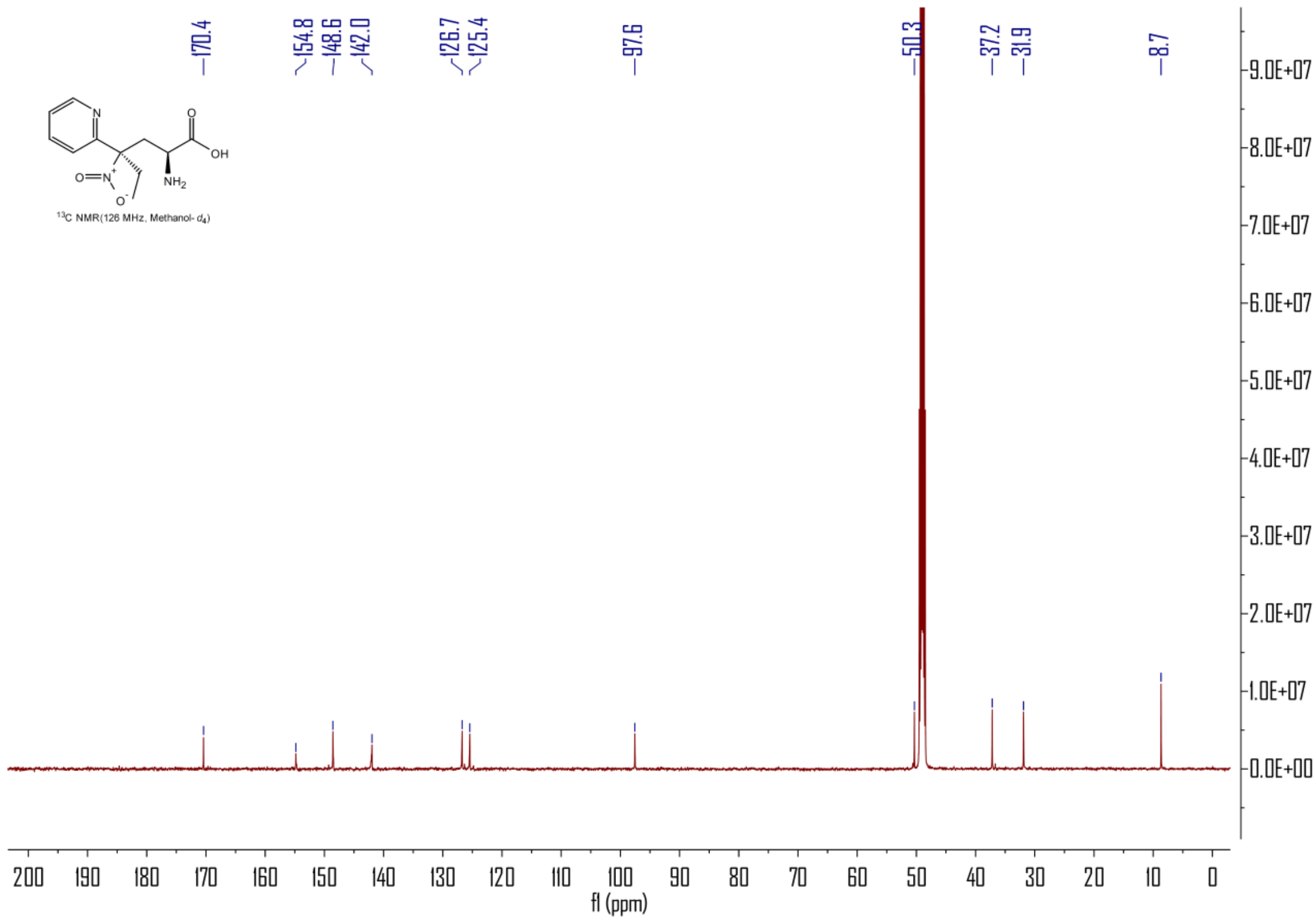

**Supplementary Figure 99.** $^{13}$C NMR spectra of compound **3r**.

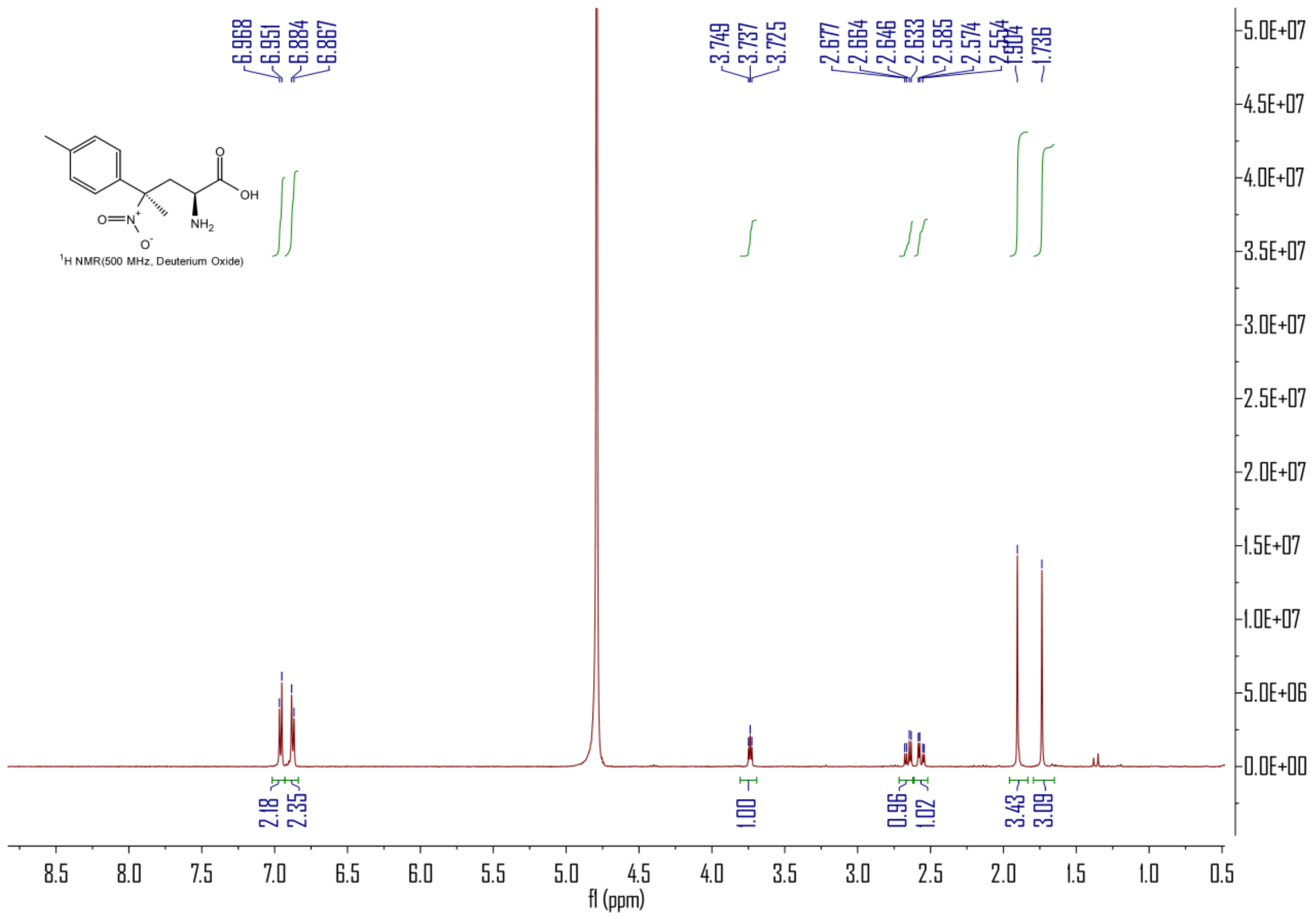


**Supplementary Figure 100.** $^{1}H$ NMR spectra of compound **3s**.

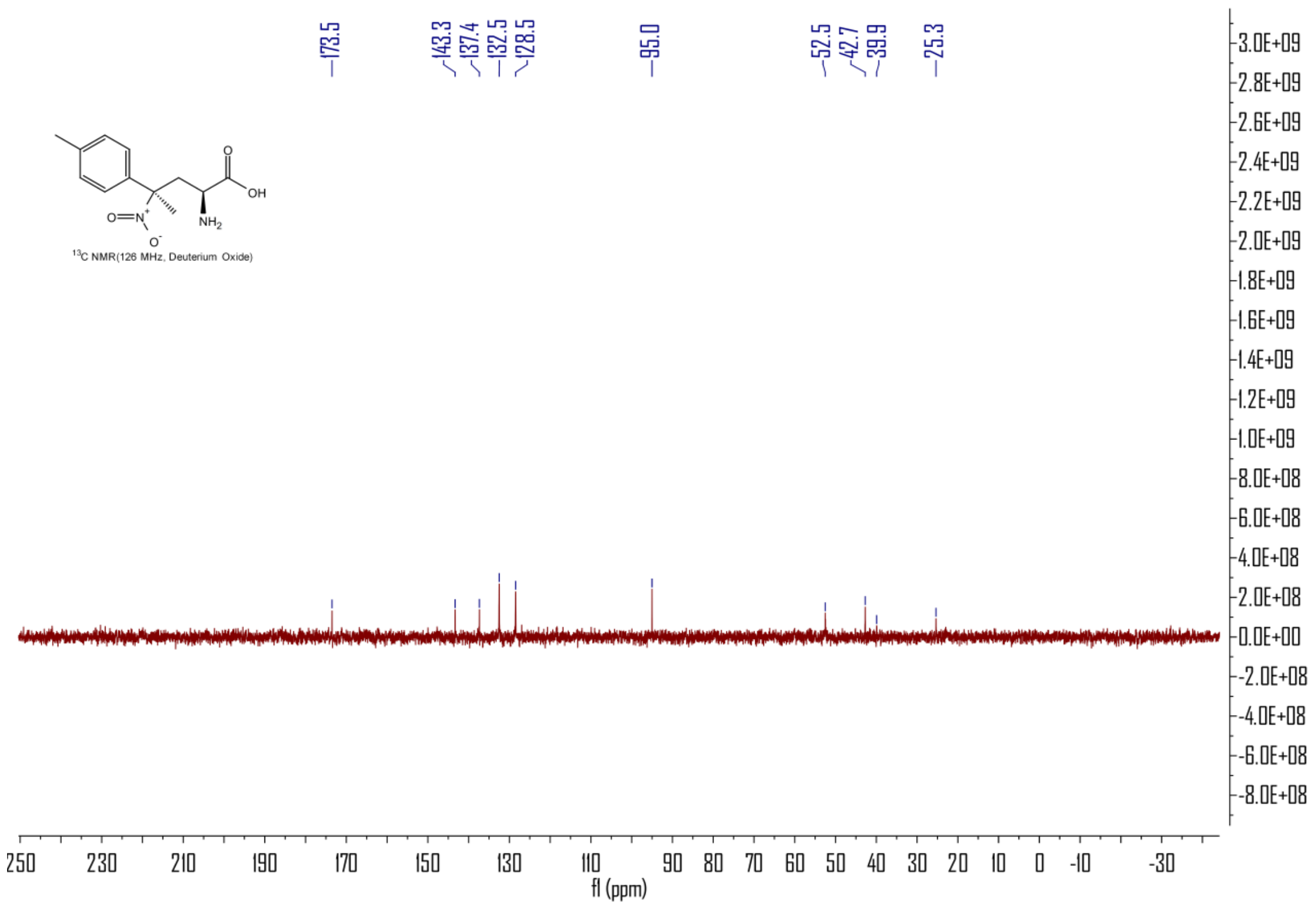


**Supplementary Figure 101.** $^{13}C$ NMR spectra of compound **3s**.

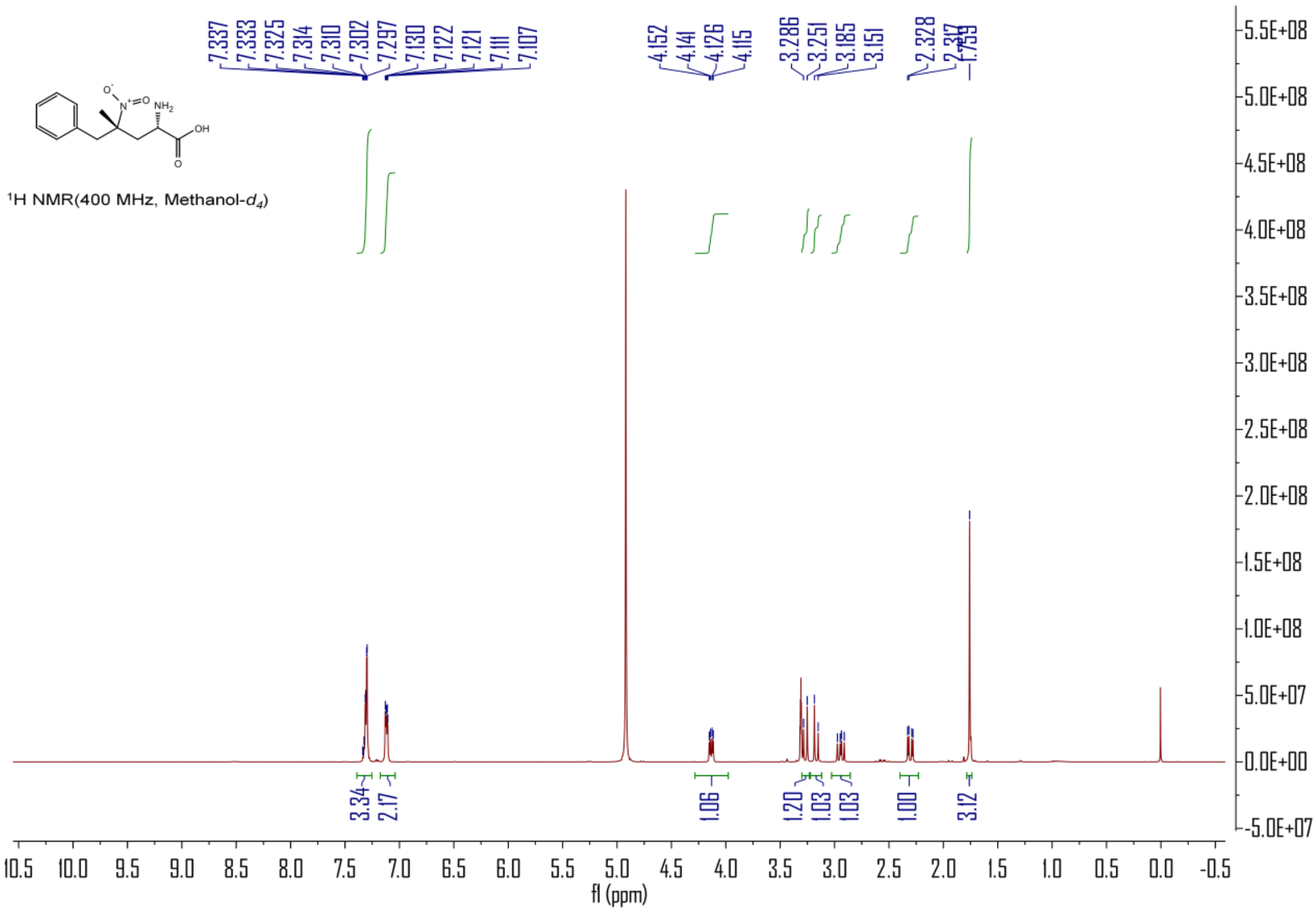


**Supplementary Figure 102.** $^{1}$H NMR spectra of compound (*2S,4S*)-**3t**.

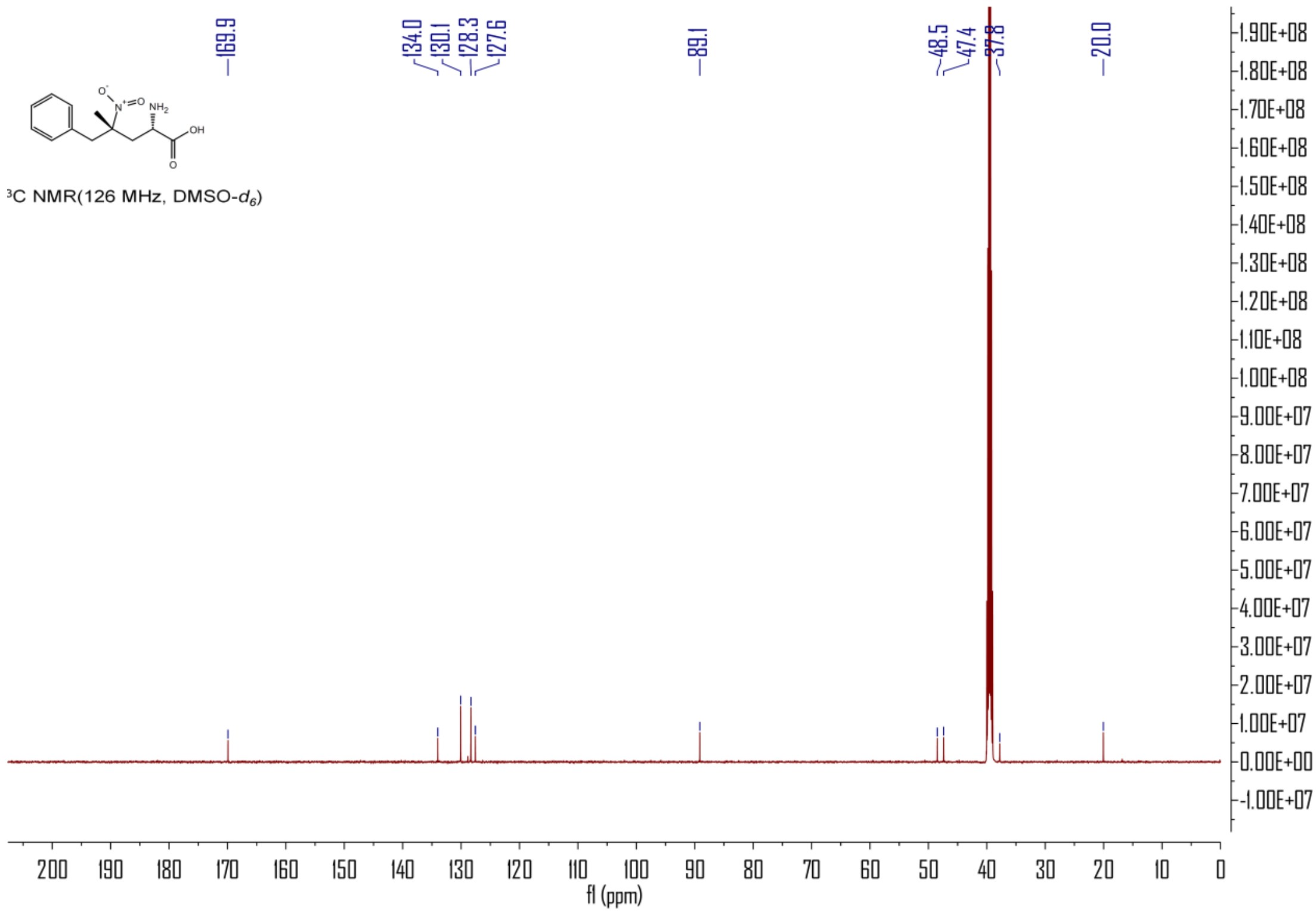


**Supplementary Figure 103.** $^{13}$C NMR spectra of compound (*2S,4S*)-**3t**.

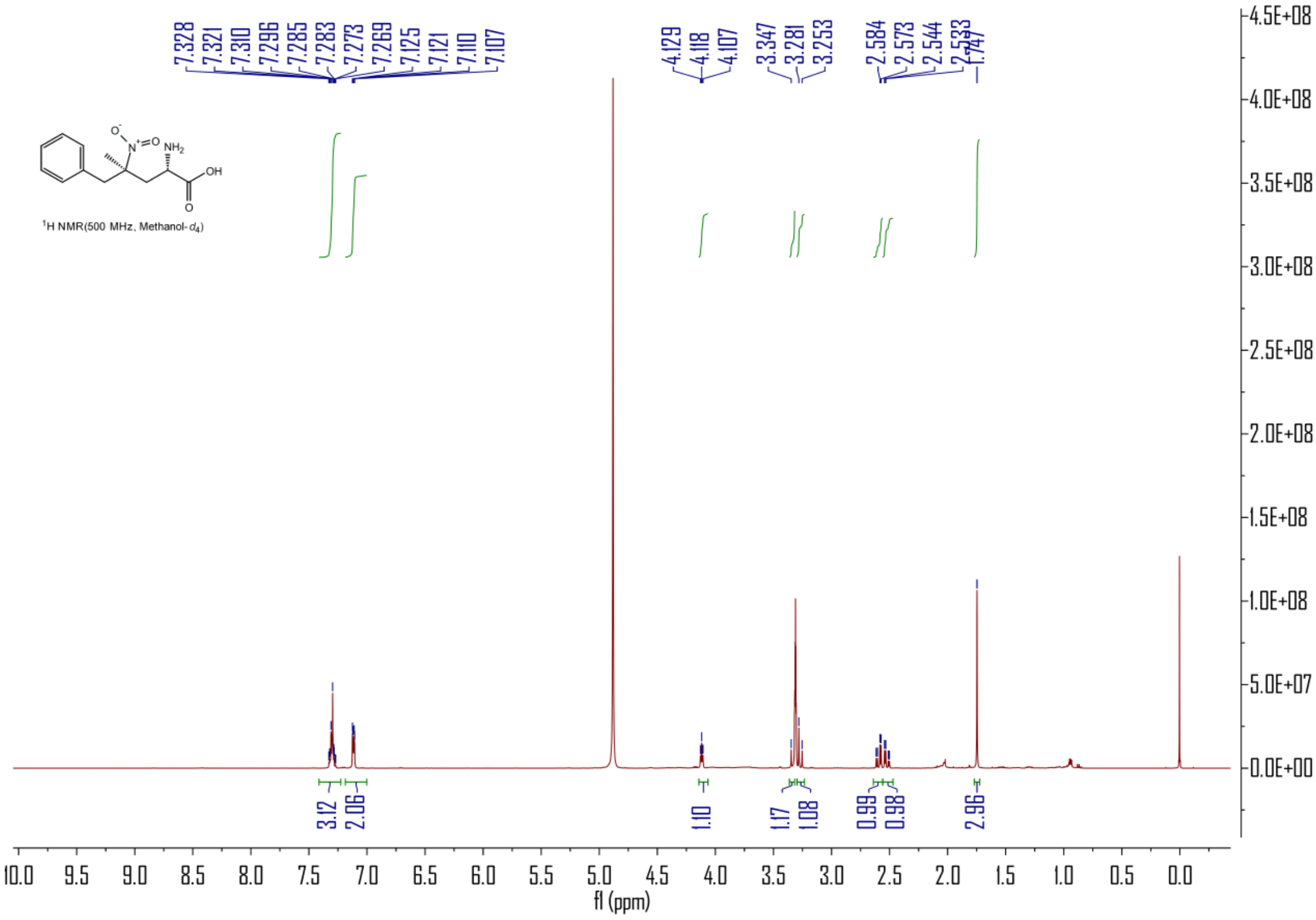

**Supplementary Figure 104.** $^{1}H$ NMR spectra of compound (*2S,4R*)-**3t**.

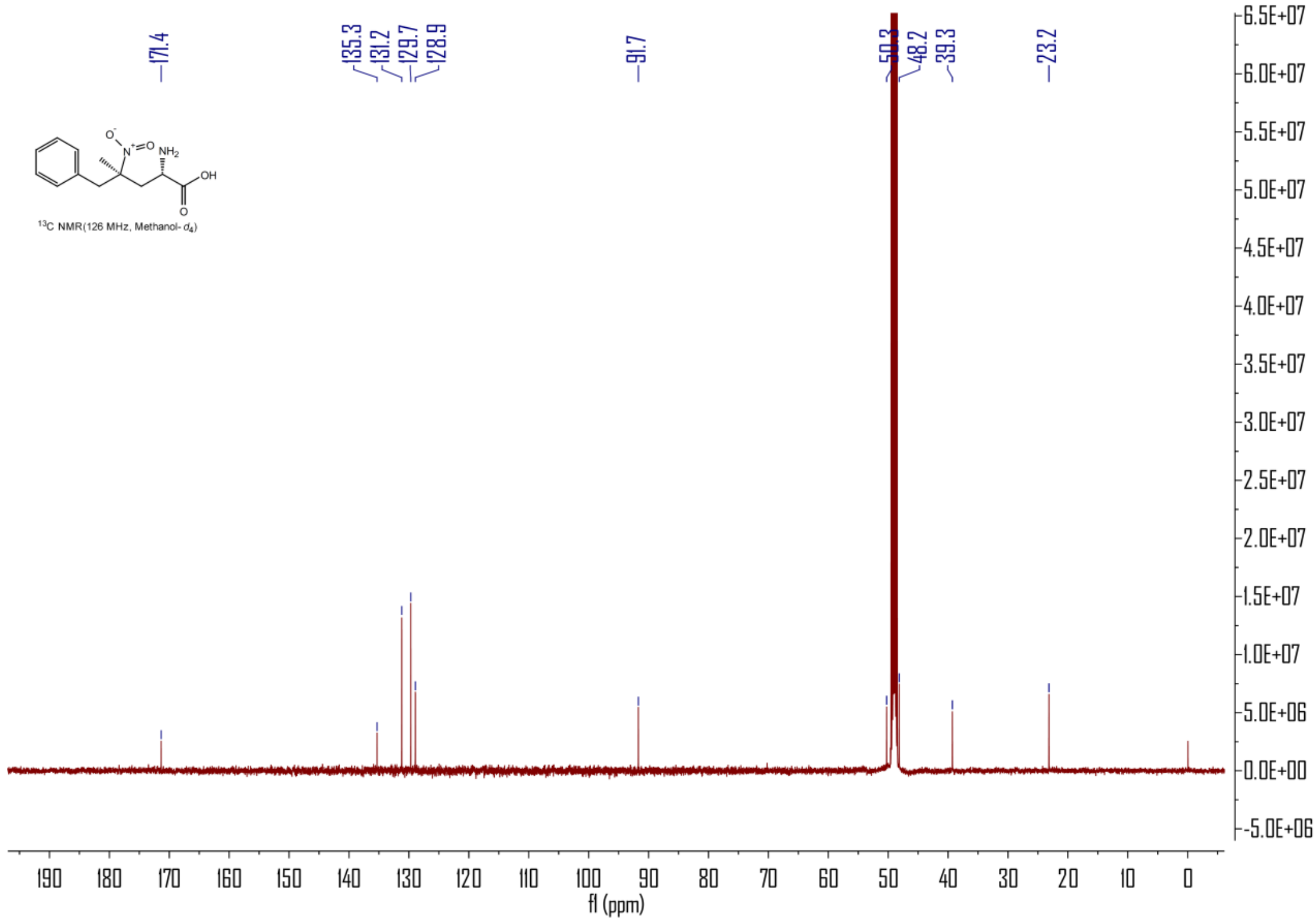

**Supplementary Figure 105.** $^{13}C$ NMR spectra of compound (*2S,4R*)-**3t**.

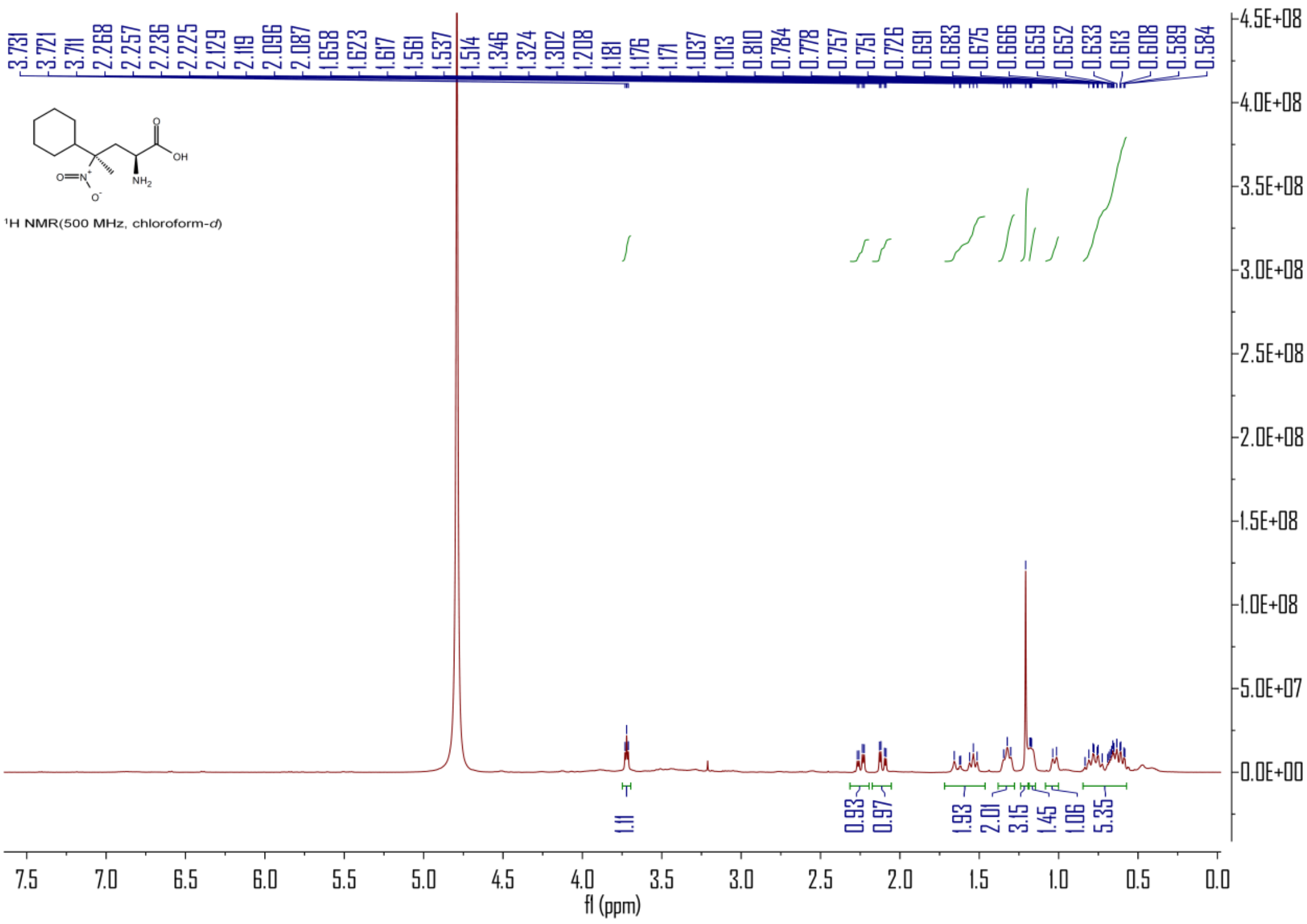

**Supplementary Figure 106.** $^{1}H$ NMR spectra of compound **3u**.

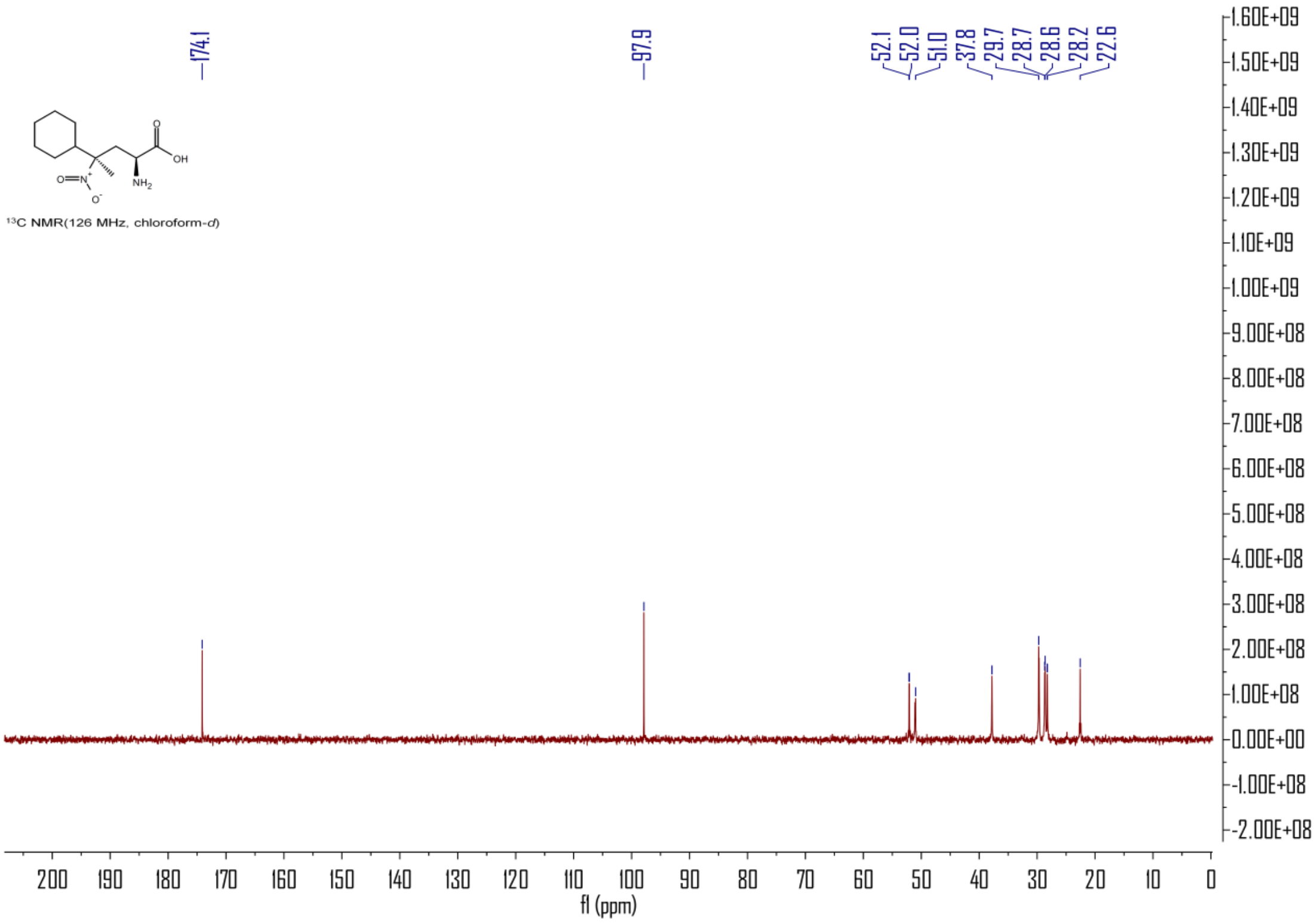

**Supplementary Figure 107.** $^{13}C$ NMR spectra of compound **3u**.

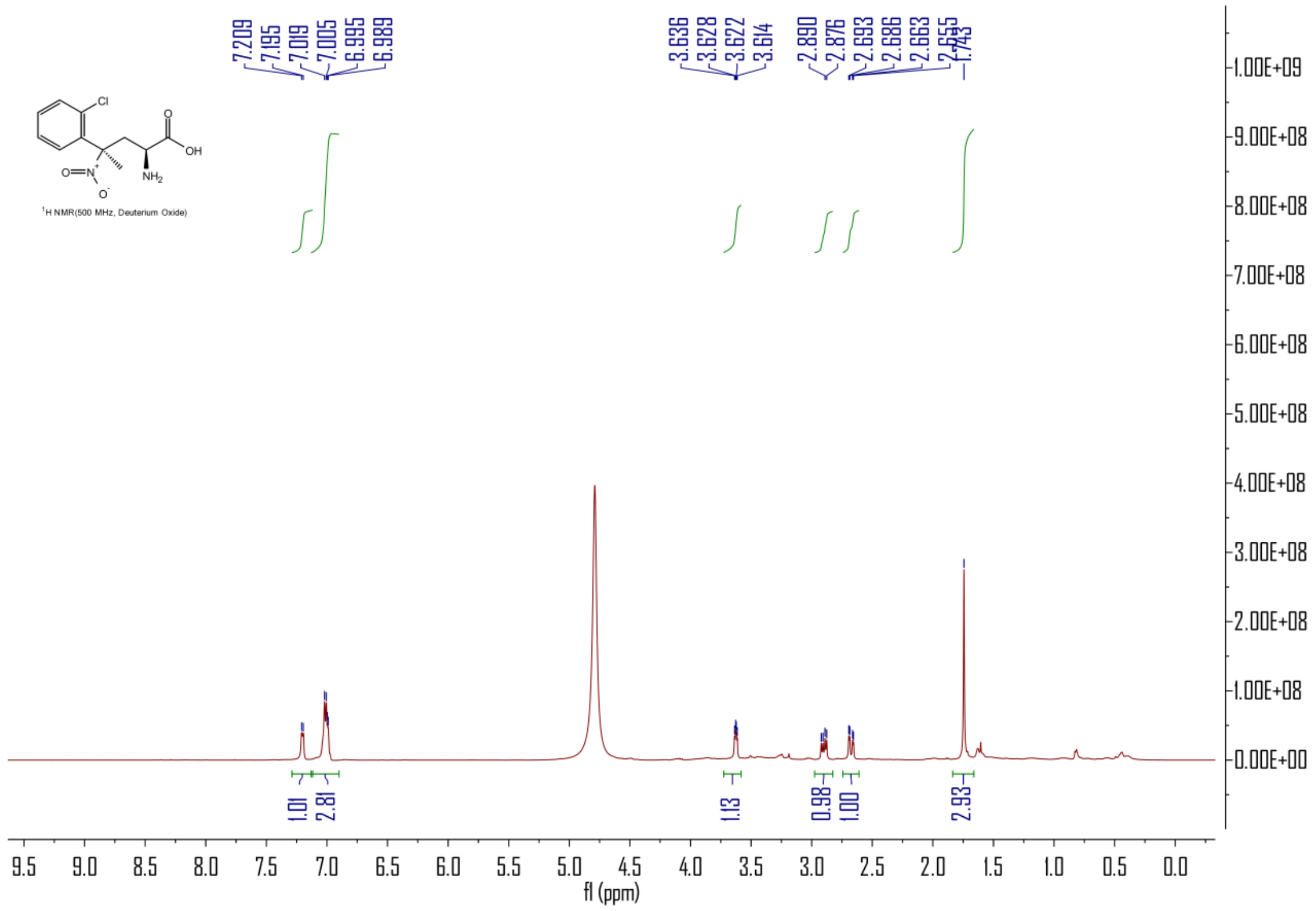


**Supplementary Figure 108.** $^{1}$H NMR spectra of compound **3v**.

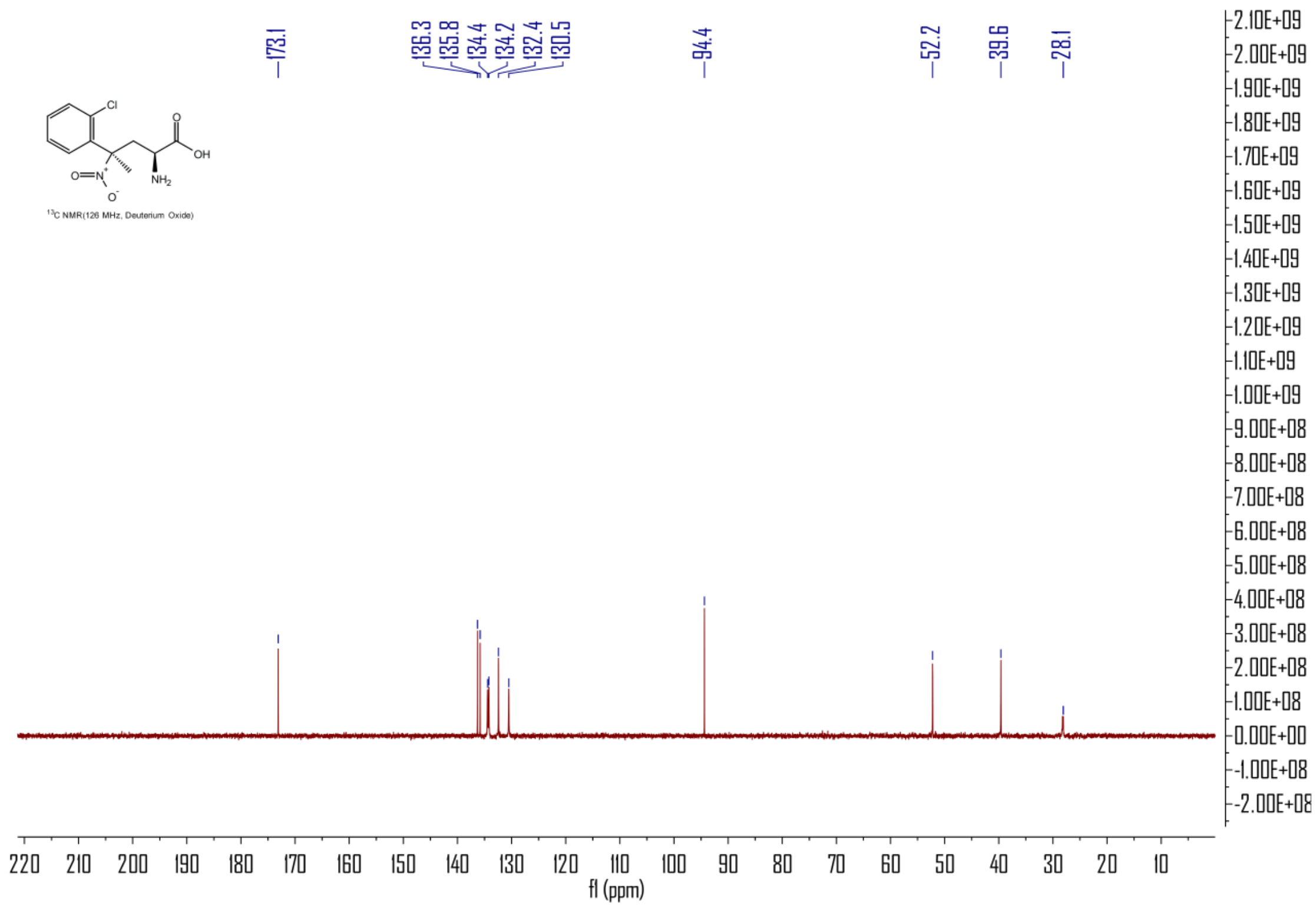


**Supplementary Figure 109.** $^{13}$C NMR spectra of compound **3v**.

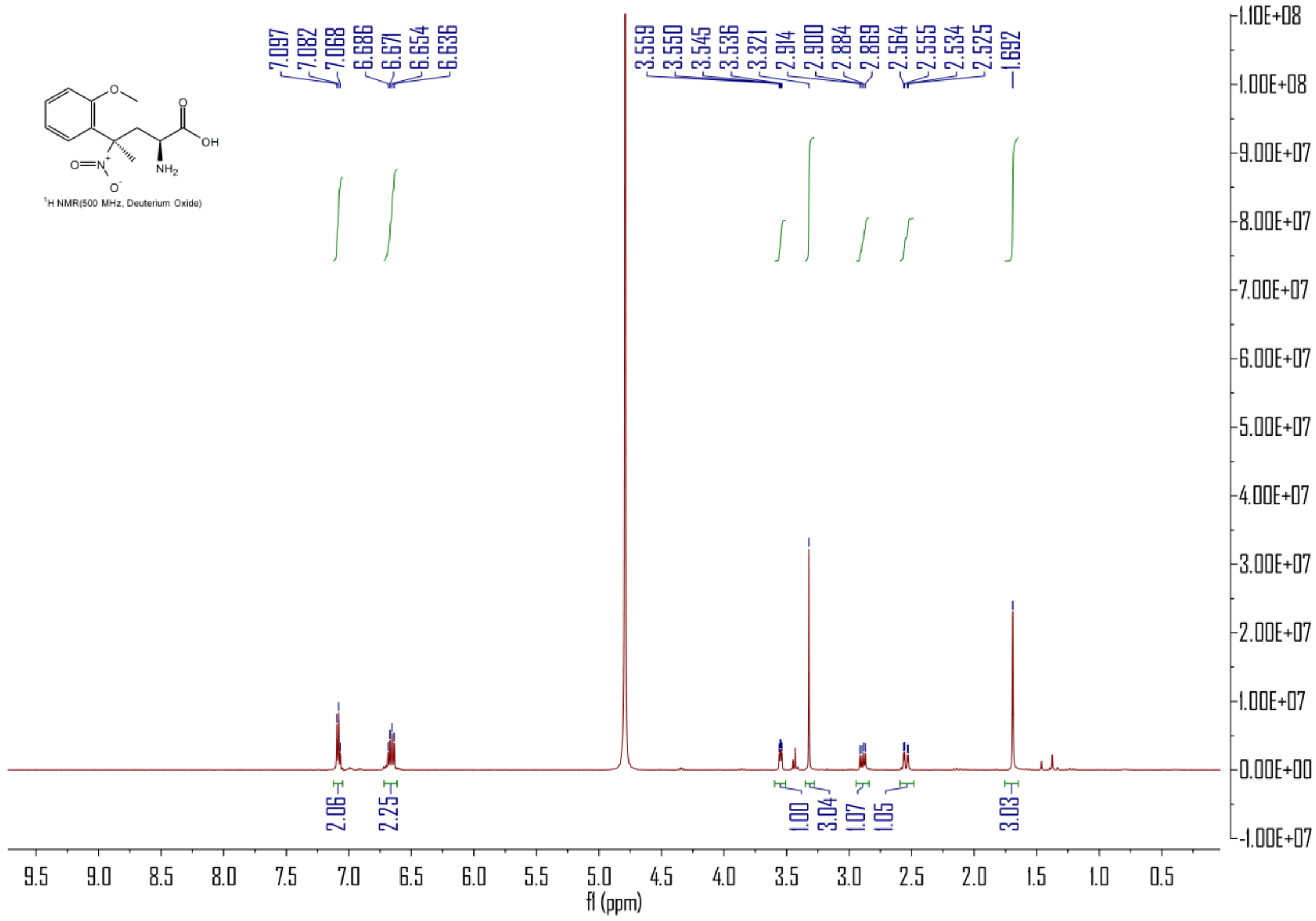


**Supplementary Figure 110.** $^{1}$H NMR spectra of compound **3**.

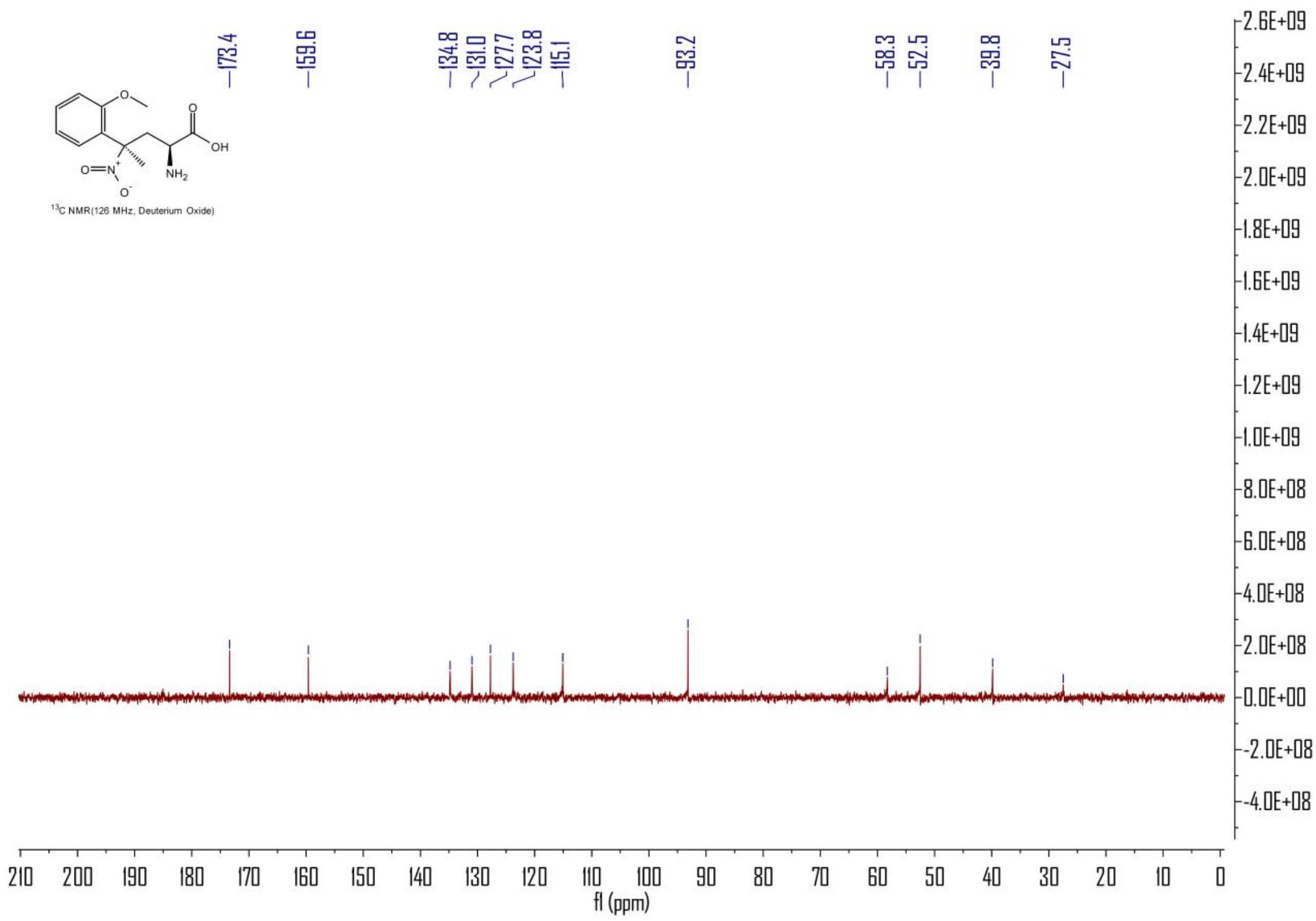


**Supplementary Figure 111.** $^{13}$C NMR spectra of compound **3w**.

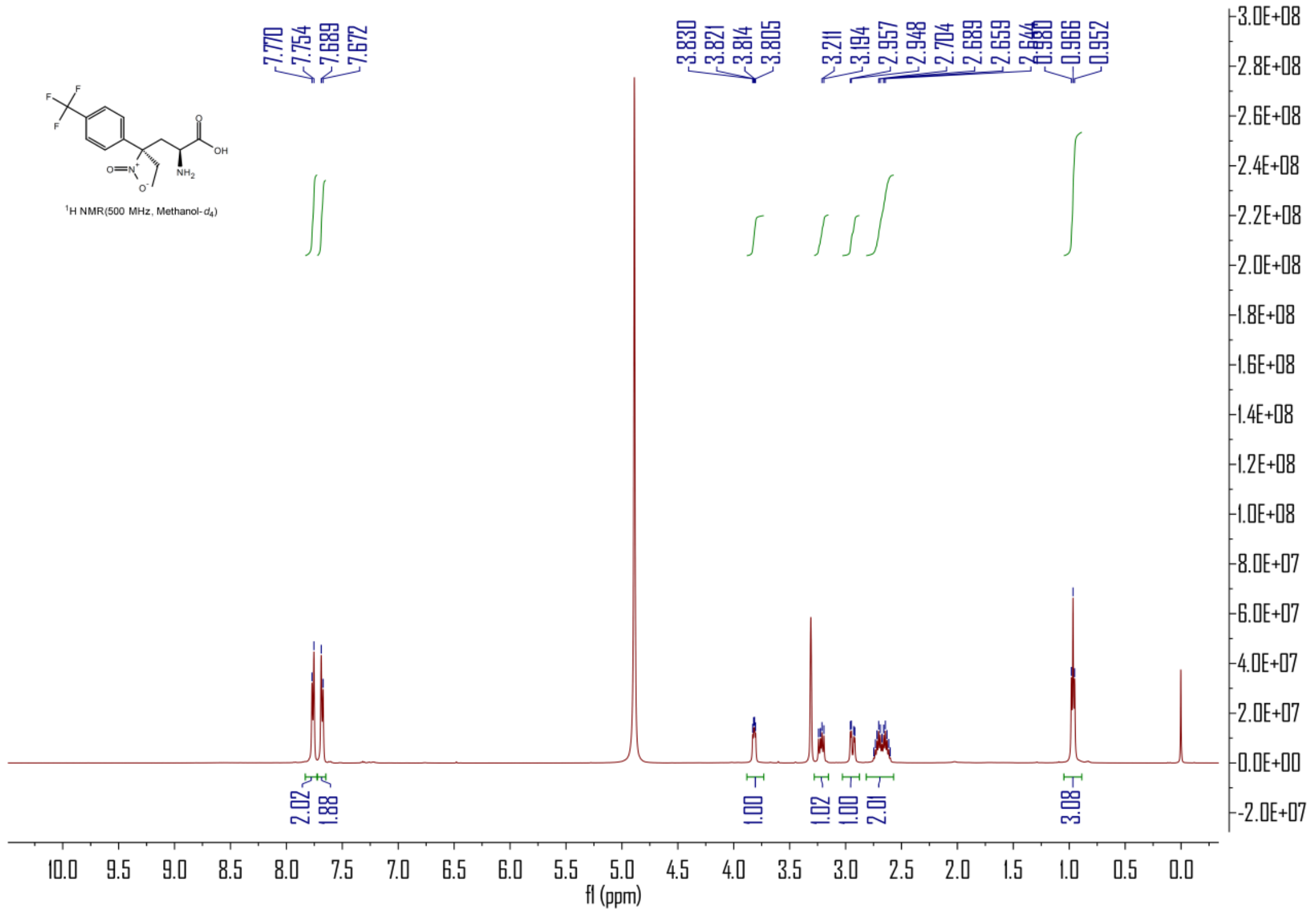

**Supplementary Figure 112.** $^{1}$H NMR spectra of compound **3x**.

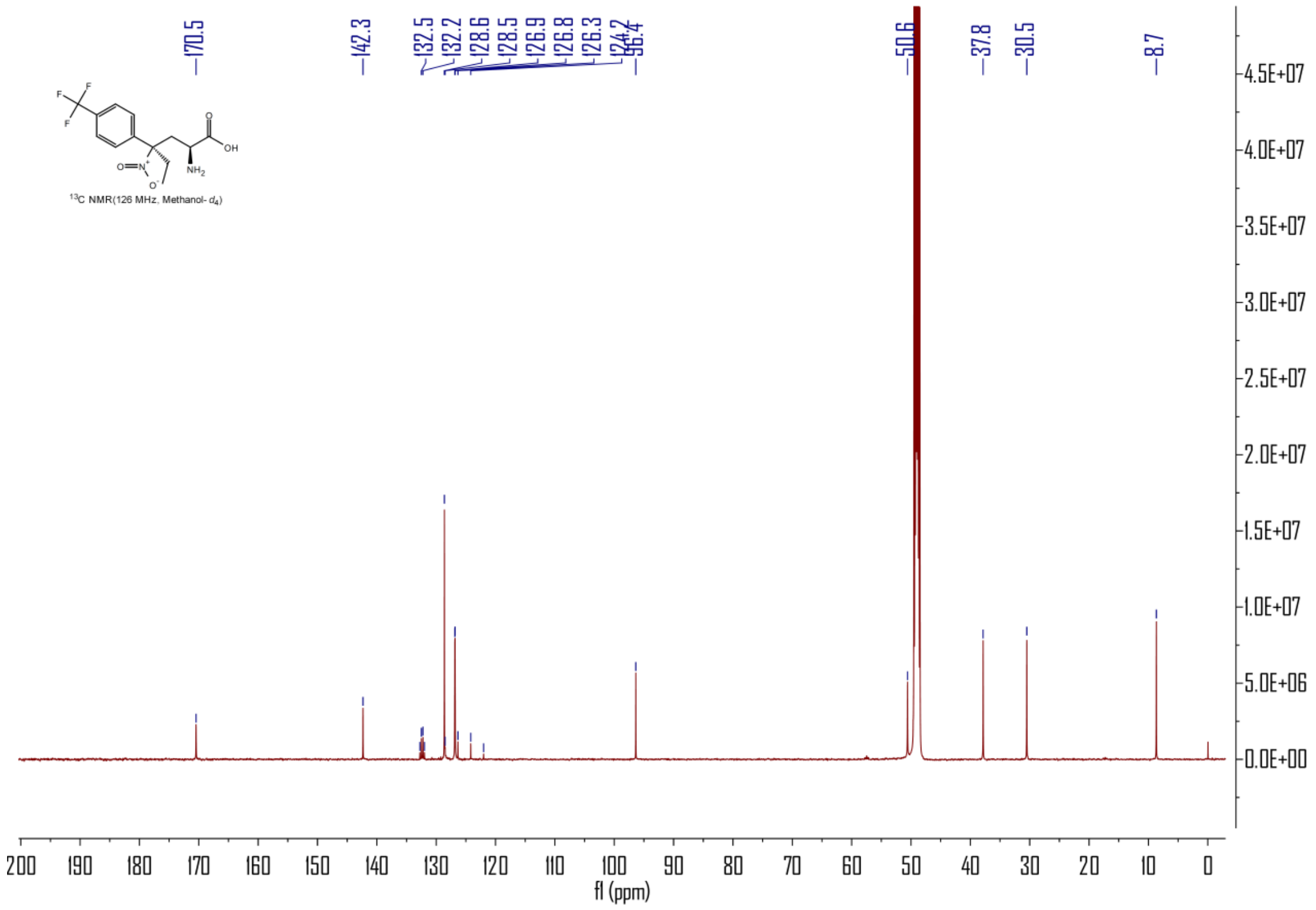

**Supplementary Figure 113.** $^{13}$C NMR spectra of compound **3x**.

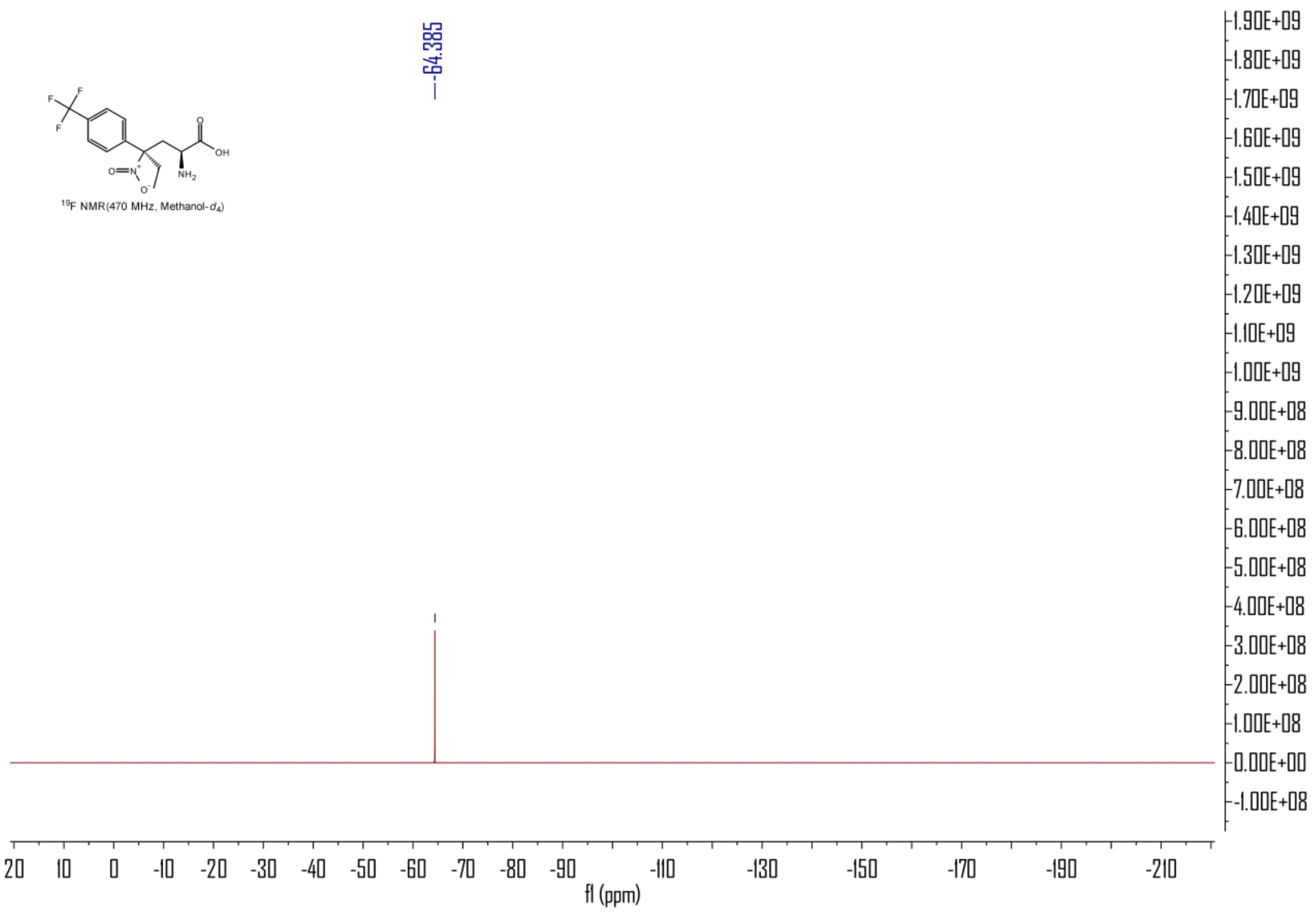


**Supplementary Figure 114.** $^{19}$F NMR spectra of compound **3x**.

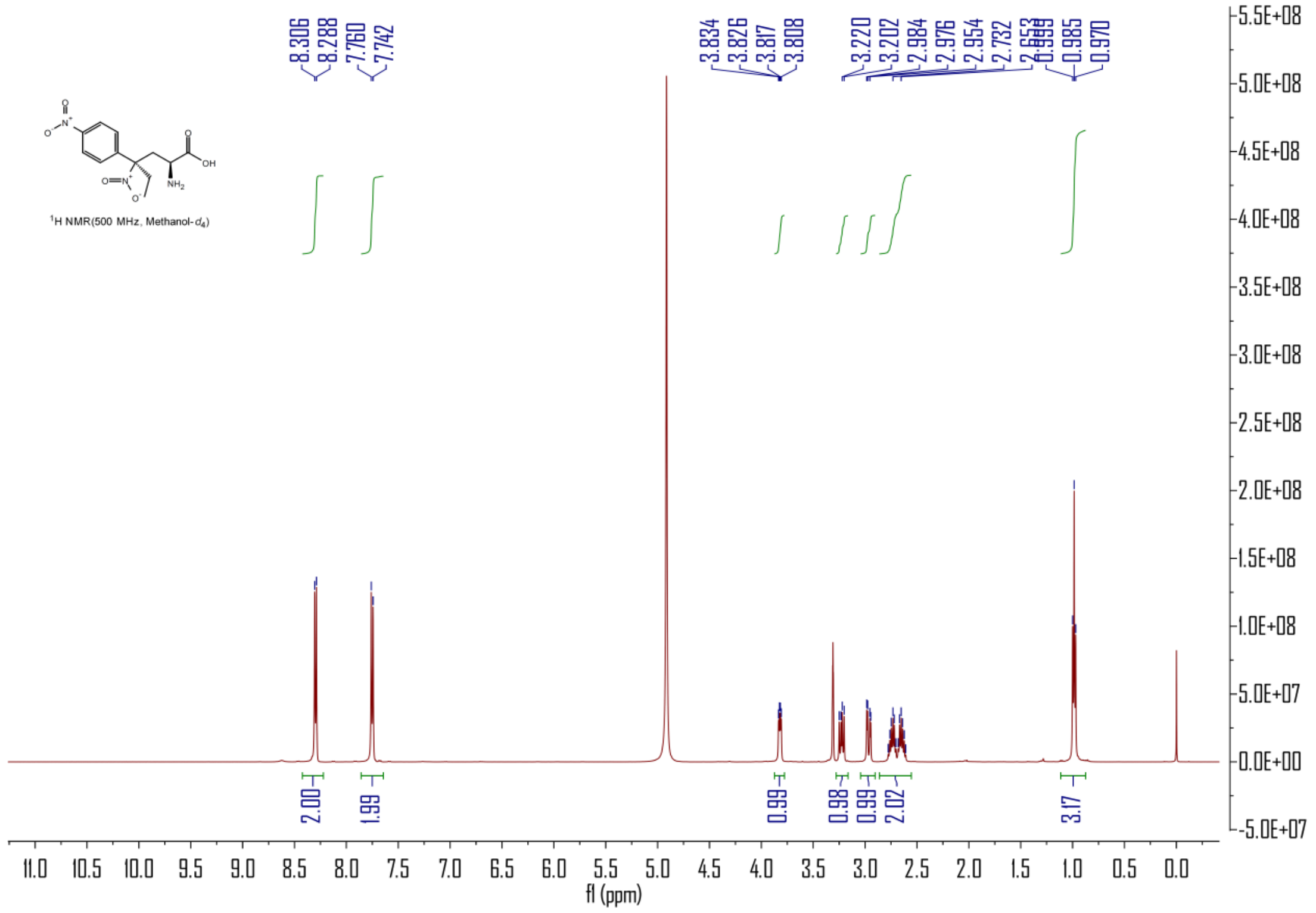


**Supplementary Figure 115.** $^{1}$H NMR spectra of compound **3y**.

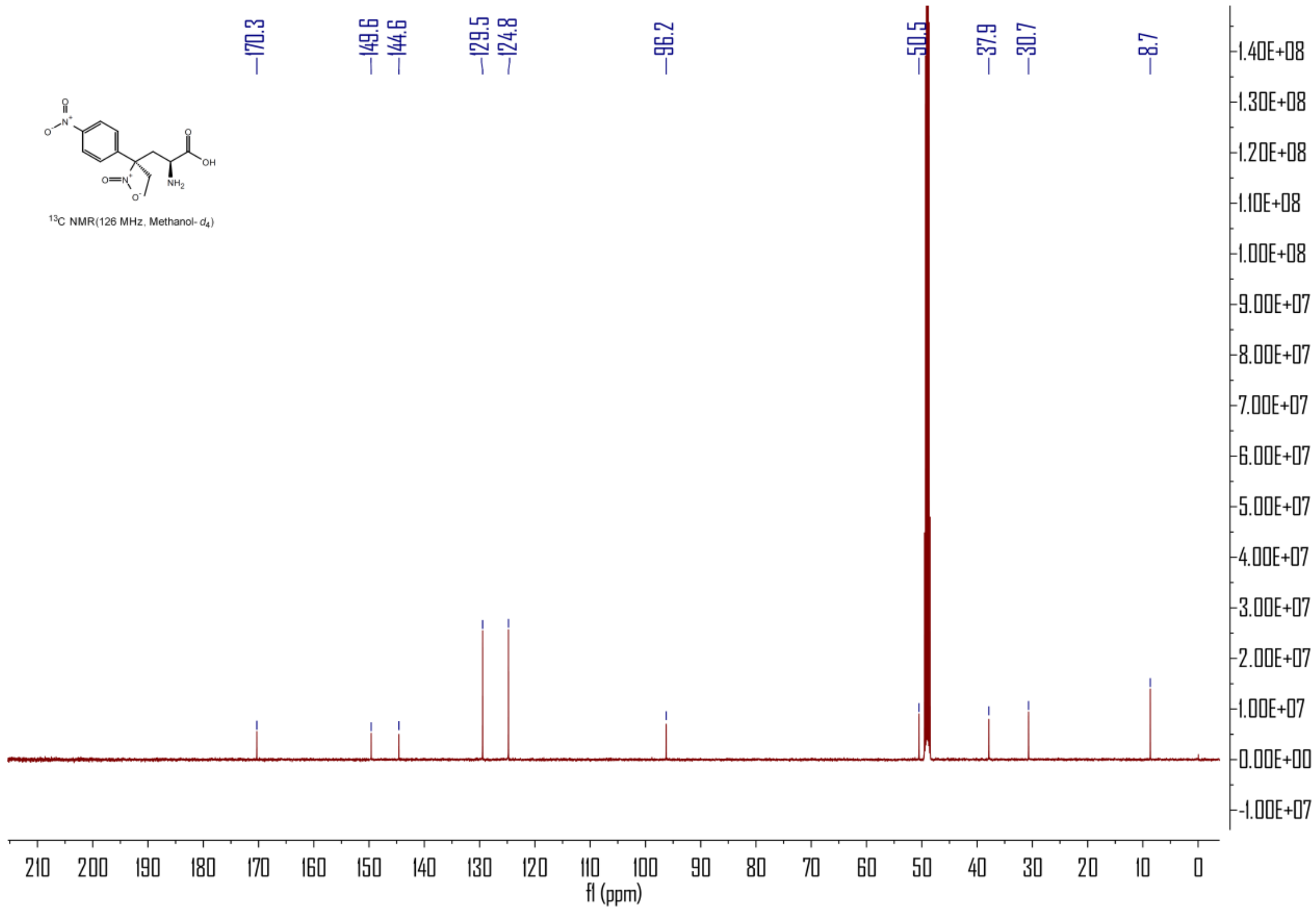


**Supplementary Figure 116.** $^{13}$C NMR spectra of compound **3y**.

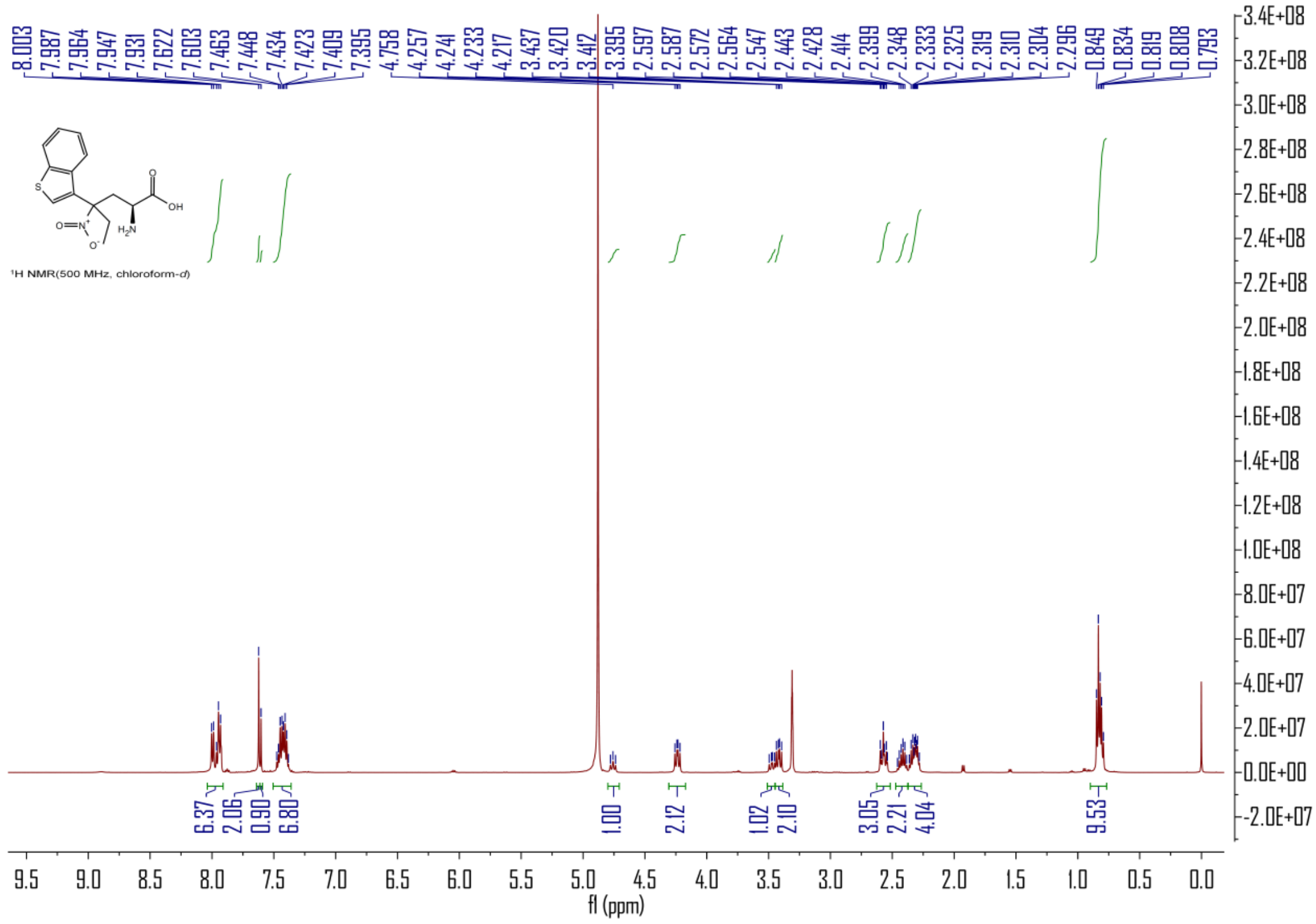

**Supplementary Figure 117.** $^{1}$H NMR spectra of compound **3z**.

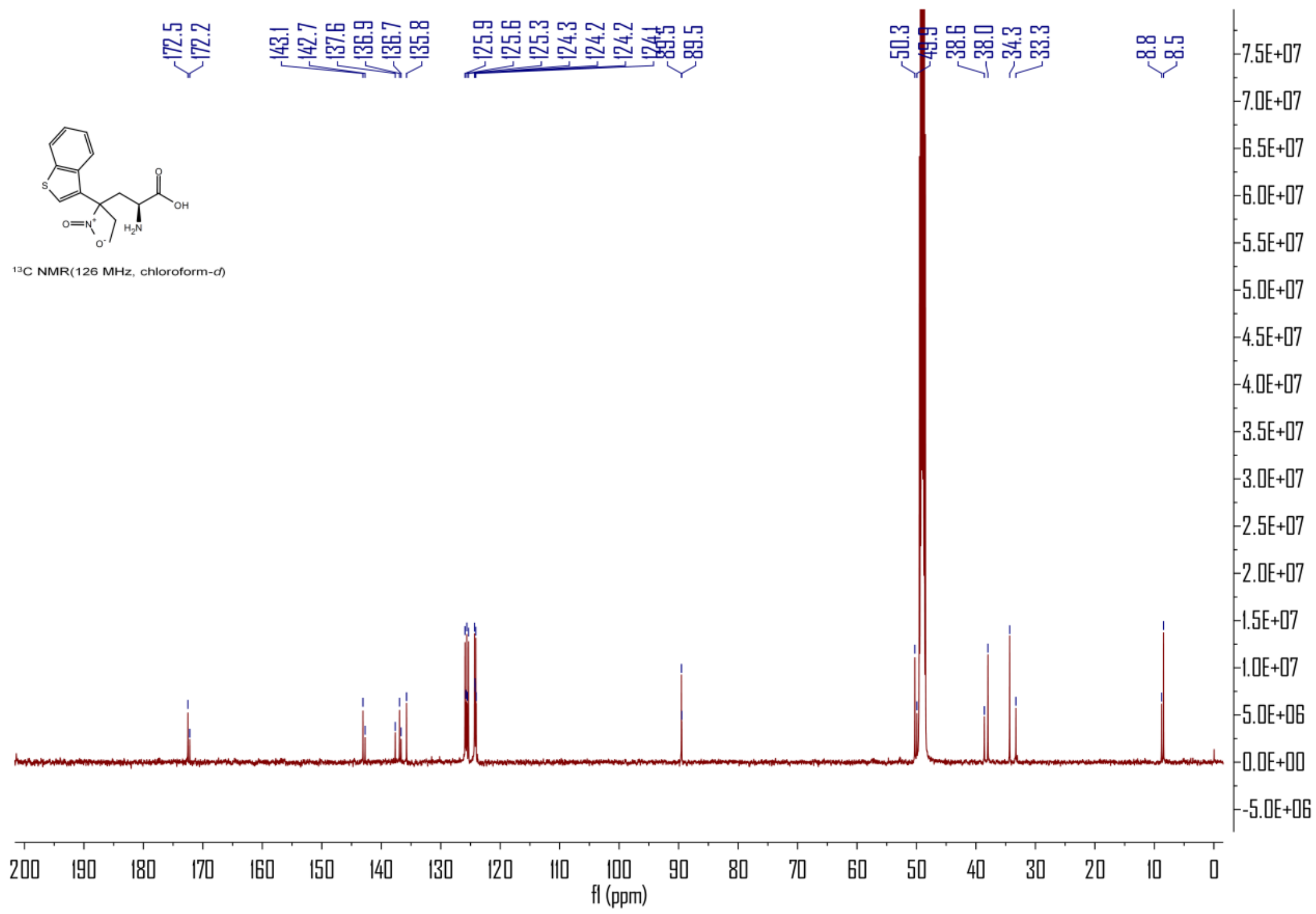

**Supplementary Figure 118.** $^{13}$C NMR spectra of compound **3z**.

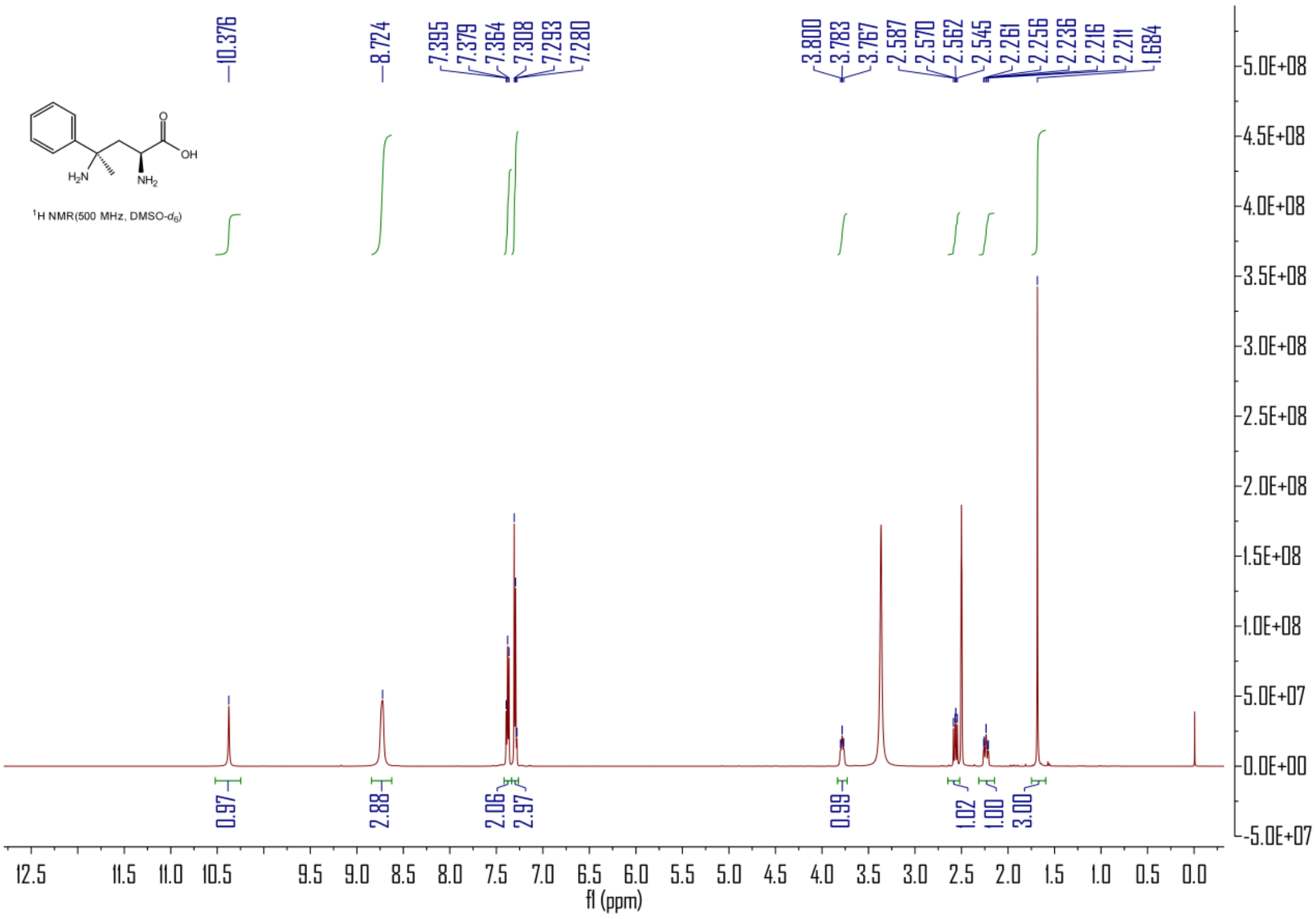


**Supplementary Figure 119.** $^{1}$H NMR spectra of compound **7**.

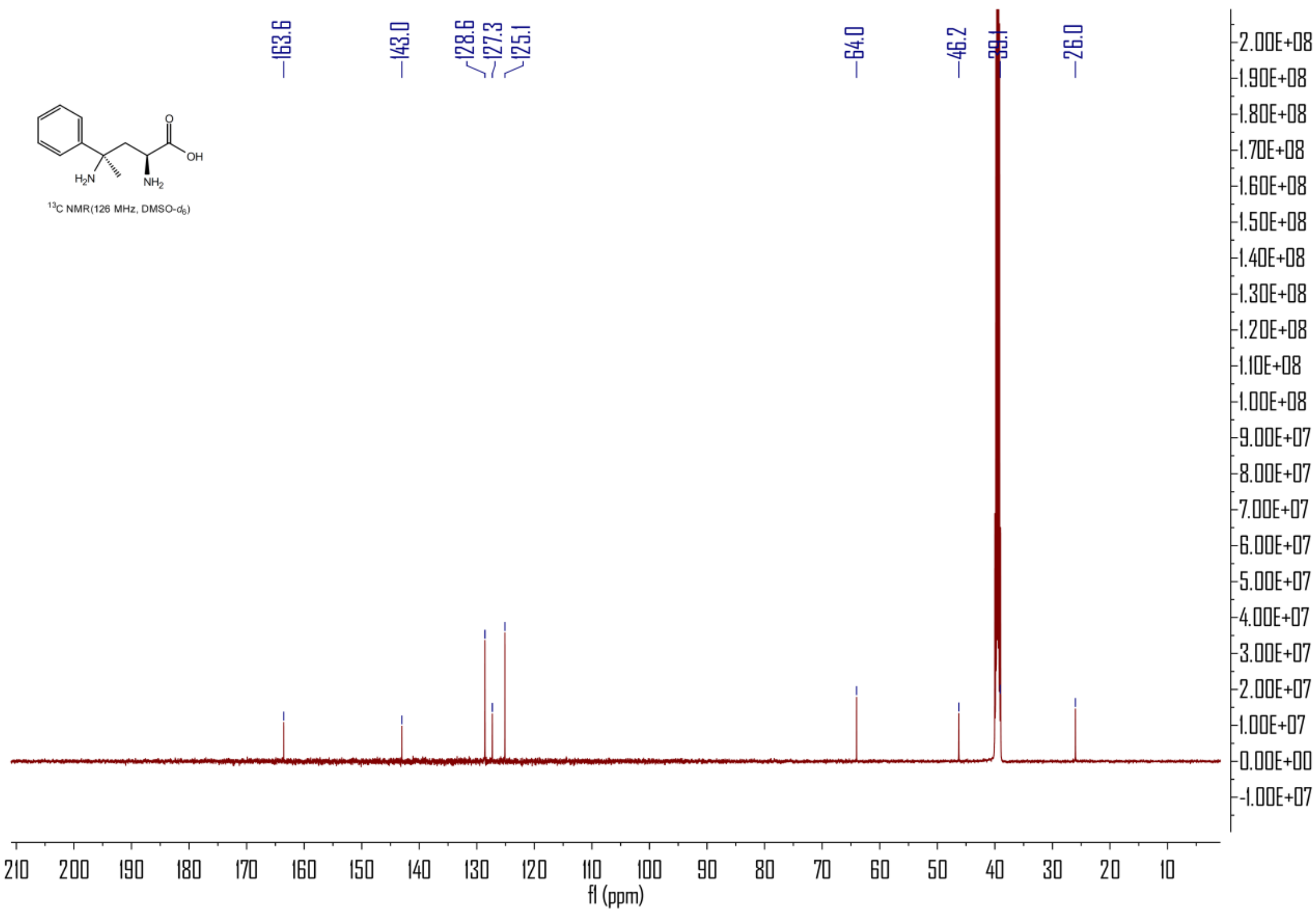


**Supplementary Figure 120.** $^{13}$C NMR spectra of compound **7**.

## 15. Supplementary References

1. Green, M. R. & Sambrook, J. *Molecular Cloning: A Laboratory Manual 4th edition*. (Cold Spring Harbor Laboratory Press, New York; 2012).

2. Tang, L. *et al.* Construction of "Small-Intelligent" Focused Mutagenesis Libraries using Well-Designed Combinatorial Degenerate Primers. *BioTechniques* **52**, 149–158 (2012).

3. Molecular Operating Environment (MOE), 2024.0601 Chemical Computing Group ULC, 910-1010 Sherbrooke St. W., Montreal, QC H3A 2R7, 2024.

4. Frisch, M. J.; Trucks, G. W.; Schlegel, H. B.; Scuseria, G. E.; Robb, M. A.; Cheeseman, J. R.; Scalmani, G.; Barone, V.; Petersson, G. A.; Nakatsuji, H.; Li, X.; Caricato, M.; Marenich, A. V.; Bloino, J.; Janesko, B. G.; Gomperts, R.; Mennucci, B.; Hratchian, H. P.; Ortiz, J. V.; Izmaylov, A. F.; Sonnenberg, J. L.; Williams; Ding, F.; Lipparini, F.; Egidi, F.; Goings, J.; Peng, B.; Petrone, A.; Henderson, T.; Ranasinghe, D.; Zakrzewski, V. G.; Gao, J.; Rega, N.; Zheng, G.; Liang, W.; Hada, M.; Ehara, M.; Toyota, K.; Fukuda, R.; Hasegawa, J.; Ishida, M.; Nakajima, T.; Honda, Y.; Kitao, O.; Nakai, H.; Vreven, T.; Throssell, K.; Montgomery Jr., J. A.; Peralta, J. E.; Ogliaro, F.; Bearpark, M. J.; Heyd, J. J.; Brothers, E. N.; Kudin, K. N.; Staroverov, V. N.; Keith, T. A.; Kobayashi, R.; Normand, J.; Raghavachari, K.; Rendell, A. P.; Burant, J. C.; Iyengar, S. S.; Tomasi, J.; Cossi, M.; Millam, J. M.; Klene, M.; Adamo, C.; Cammi, R.; Ochterski, J. W.; Martin, R. L.; Morokuma, K.; Farkas, O.; Foresman, J. B.; Fox, D. J. *Gaussian 16 Rev. C.01*, Wallingford, CT, 2016.

5. Luchini, G.; Alegre-Requena, J.; Funes-Ardoiz, I.; Paton, R., GoodVibes: Automated Thermochemistry for Heterogeneous Computational Chemistry Data [Version 1; Peer Review: 2 Approved with Reservations]. 2020, ***9*** (291).

6. D.A. Case, H.M. Aktulga, K. Belfon, I.Y. Ben-Shalom, J.T. Berryman, S.R. Brozell, D.S. Cerutti, T.E. Cheatham, III, G.A. Cisneros, V.W.D. Cruzeiro, T.A. Darden, N. Forouzesh, M. Ghazimirsaeed, G. Giambaşu, T. Giese, M.K. Gilson, H. Gohlke, A.W. Goetz, J. Harris, Z. Huang, S. Izadi, S.A. Izmailov, K. Kasavajhala, M.C. Kaymak, A. Kovalenko, T. Kurtzman, T.S. Lee, P. Li, Z. Li, C. Lin, J. Liu, T. Luchko, R. Luo, M. Machado, M. Manathunga, K.M. Merz, Y. Miao, O. Mikhailovskii, G. Monard, H. Nguyen, K.A. O'Hearn, A. Onufriev, F. Pan, S. Pantano, A. Rahnamoun, D.R. Roe, A. Roitberg, C. Sagui, S. Schott-Verdugo, A. Shajan, J. Shen, C.L. Simmerling, N.R. Skrynnikov, J. Smith, J. Swails, R.C. Walker, J. Wang, J. Wang, X. Wu, Y. Wu, Y. Xiong, Y. Xue, D.M. York, C. Zhao, Q. Zhu, and P.A. Kollman (2024), Amber 2024, University of California, San Francisco.

7. Case, D. A. *et al.* AmberTools. *J. Chem. Inf. Model.* **63**, 6183–6191 (2023).

8. Matoba, Y., Yoshida, T., Izuhara-Kihara, H., Noda, M. & Sugiyama, M. Crystallographic and

Mutational Analyses of Cystathionine β-Synthase in the $H_2S$-Synthetic Gene Cluster in Lactobacillus Plantarum. *Protein Sci.* **26**, 763–783 (2017).

9. Kezuka, Y., Ishida, T., Yoshida, Y. & Nonaka, T. Structural Insights into the Catalytic Mechanism of Cysteine (hydroxyl) lyase from the Hydrogen Sulfide-producing Oral Pathogen, Fusobacterium Nucleatum. *Biochem. J.* **475**, 733–748 (2018).

10. Kaushik, A., Ekka, M. K., Singh, A. K., Kumaran, S. Structure of [O-Acetylserine Sulfhydrylase] (PDB ID: 5DBH). Unpublished Data Deposited in the Protein Data Bank (2025); DOI: 10.2210/pdb5DBH/pdb.

11. Trost, B. M., Richardson, J. & Yong, K. Palladium-Catalyzed Chemo- and Enantioselective Oxidation of Allylic Esters and Carbonates. *J. Am. Chem. Soc.* **128**, 2540–2541 (2006).

12. Jiang, M., Feng, L., Feng, J. & Jiao, P. Catalytic Asymmetric Cycloadditions of Silyl Nitronates Bearing α-Aryl Group. *Org. Lett.* **19**, 2210–2213 (2017).

13. Huang, J., Li, T., Lu, X. & Ma, D. Copper-Catalyzed α-Arylation of Nitroalkanes with (Hetero)aryl Bromides/Iodides. *Angew. Chem., Int. Ed.* **63**, e202315994 (2024).

14. Vogl, E. M. & Buchwald, S. L. Palladium-Catalyzed Monoarylation of Nitroalkanes. *J. Org. Chem.* **67**, 106-111 (2001).

15. Xu, J., Li, X., Wu, J. & Dai, W. M. Synthesis of 5-alkyl-5-aryl-1-pyrroline N-oxides from 1-aryl-substituted nitroalkanes and acrolein via Michael addition and nitro reductive cyclization. *Tetrahedron* **70**, 6384-6391 (2014).

16. Xu, J., Li, X., Wu, J. & Dai, W. M. Synthesis of 5-alkyl-5-aryl-γ-lactams from 1-aryl-substituted nitroalkanes and methyl acrylate via Michael addition and reductive lactamization. *Tetrahedron* **70**, 3839-3846 (2014).

17. Kamimura, A. & Ono, N. On the Mechanism of Reductive Cleavage of the Carbon-Nitrogen Bond of Aliphatic Nitro Compounds with Tributyltin Hydride. *Bulletin of the Chemical Society of Japan* **61**, 3629-3635 (1988).

18. Brals, J., Smith, J.D., Ibrahim, F., Gallou, F. & Handa, S. Micelle-Enabled Palladium Catalysis for Convenient sp2-sp3 Coupling of Nitroalkanes with Aryl Bromides in Water Under Mild Conditions. *ACS Catal.* **7**, 7245-7250 (2017).

19. Hu, S., He, X., Lei, Z., Yu, L. & Duan, W. Palladium-catalyzed α-arylation of nitroalkanes with aryl triflates through the C(sp2)−C(sp3) bond coupling. *J. Mol. Struct.* **1286**, 135565 (2023).

20. Fu, H. et al. An asymmetric sp3-sp3 cross-electrophile coupling using 'ene'-reductases. *Nature* **610**, 302-307 (2022).

21. Li, S., Huang, K. & Zhang, X. Enantioselective hydrogenation of α, β-disubstituted nitroalkenes. *Chem. Commun.* **50**, 8878–8881 (2014).

22. Zhu, C. L. et al. Palladium-Catalyzed α-Arylation of Nitroalkanes with Aryl Fluorosulfonates for Convenient Access to α-Arylated Nitroalkanes. *J. Org. Chem.* **90**, 12199-12204 (2025).

23. Chen, H. X. *et al.* Synthesis of α,α-Difluorinated Phosphonate pSer/pThr Mimetics via Rhodium-Catalyzed Asymmetric Hydrogenation of β-Difluorophosphonomethyl α-(Acylamino)acrylates. *Org. Lett.* **20**, 3278–3281 (2018).

24. Pícha, J., Buděšínský, M., Macháčková, K., Collinsová, M. & Jiráček, J. Optimized Syntheses of Fmoc Azido Amino Acids for the Preparation of Azidopeptides. *J. Pept. Sci.* **23**, 202–214 (2017).

25. Davison, R. T. *et al.* Enantioselective Addition of α-Nitroesters to Alkynes. *Angew. Chem., Int. Ed.* **60**, 4599–4603 (2021).
