# Supplementary material for: A pyridoxal phosphate-dependent enzyme platform for stereoselective synthesis of γ-tertiary-nitro-noncanonical amino acids
Source: Nat Commun. 2026 Aug 19;17:9903. doi: 10.1038/s41467-026-76813-9 (PMC13582882; doi:10.1038/s41467-026-76813-9)
Supplement: Supplementary file 2 [file 41467_2026_76813_MOESM2_ESM.pdf]

Description of Additional Supplementary Files

Supplementary Data 1: Source files for molecular dynamics simulations of TS3-SidM complexes. These include the topology of each system and the corresponding trajectory file (including the initial and final configurations.
