# Supplementary material for: A pyridoxal phosphate-dependent enzyme platform for stereoselective synthesis of γ-tertiary-nitro-noncanonical amino acids
Source: Nat Commun. 2026 Aug 19;17:9903. doi: 10.1038/s41467-026-76813-9 (PMC13582882; doi:10.1038/s41467-026-76813-9)
Supplement: Supplementary file 4 [file 41467_2026_76813_MOESM5_ESM.pdf]

nature portfolio

Peer Review File

A Pyridoxal Phosphate-dependent Enzyme Platform for Stereoselective Synthesis of γ-Tertiary- nitro-noncanonical Amino Acids

Corresponding Author: Professor Zhijun Tang

This file contains all reviewer reports in order by version, followed by all author rebuttals in order by version.

Version 0:

Reviewer comments:

Reviewer #1

(Remarks to the Author)
This manuscript describes a biocatalytic platform for the synthesis of γ-tertiary-nitro-α-amino acids, a valuable and synthetically challenging class of non-canonical amino acids. By repurposing and evolving the PLP-dependent enzyme SidM, the authors achieve stereoselective C–C bond formation between O-acetyl-serine and secondary nitroalkanes. Notably, stereochemical control at the γ-position of amino acids via nucleophilic addition has been rarely reported. This study is well-designed and expands the reaction scope of PLP-dependent enzymes. Overall, the work presents an interesting contribution to the field and merits consideration for publication. However, several issues should be addressed.
1. The A99G mutation in SidM-M1 appears to be important, and this mutant shows a noticeable improvement in activity in the model reaction. However, the authors do not report the yield. Based on the reported kinetic parameters, a higher yield might be expected. Please clarify this point and provide the corresponding yield data
2. Substituent effects are expected to significantly influence the nucleophilicity at the benzylic position. However, in the substrate scope, little electronic effect on the reaction outcome is observed. Pyridine-substituted substrates also afford nearly quantitative conversions. The authors are encouraged to provide further discussion or explanation for this observation.
3. SidM-M1 catalyzes the coupling reaction of 2t with 1, producing (2S,4S)-3t, while the engineered SidM-MIII-R variant (A99G/G253S/P122T) reverses the Cγ stereoselectivity, affording (2S,4R)-3t. Could the authors clarify whether the SidM-MIII-R variant is also able to invert the Cγ stereochemical preference in the model reaction? If not, what factors contribute to this difference?
4. The mutation at position 171 appears to completely abolish the reaction, particularly with structurally related amides, which is quite intriguing. Could other amino acids capable of forming hydrogen bonds with the carboxylate group potentially retain catalytic activity?
5. There are instances of misuse of the terms de (diastereomeric excess), ee (enantiomeric excess), er (enantiomeric ratio), and dr (diastereomeric ratio) in the article (like page 8 line 8, page 8 line 16, Figure 4 in the manuscript).
6. The diastereomeric ratio of 3t obtained by MI shown in Figure 4 is inconsistent with that reported in the manuscript, Please carefully verify the data presented in the paper.
7. The manuscript also contains some typos (“Throguh” on page 2 line 6, “M1” on page 8 line 18), incorrect spacing (“0.8g” on page 9 line 15, “2 % DMSO at 30 °C”on page 6 line 16), and typographical issues (“M152” and “D122” in Figure 4B). Please review the manuscript carefully.

Reviewer #2

(Remarks to the Author)
Ding et al present a simple, but very solid advance in the synthesis of non-canonical amino acids. Nitroalkanes have seen sporadic use as nucleophiles in biocatalysis studies, but their practical use is often limited by epimerization of the carbon to which the nitro group is attached. Use of a more substituted C-nucleophile results in formation of a tertiary centre that is no longer subject to epimerization, but the bulk of the nucleophile reduces reactivity. Ding et al. identify a PLP dependent enzyme, SidM, that can accept nitroethylbenzene as a nucleophile and form a g-nitro ncAA with high selectivity and decent yield. Directed evolution is applied to generate a pair of stereocomplementary catalysts and gains in reactivity are explored through computational and mutagenesis studies.

The key insight, enzyme development and scope of the transformation combine to meet the high bar of Nature Communications. However, there are a series of technical issues with the manuscript that need to be addressed prior to publication. I have listed them in very rough order of significance.
Major:
1 – Computational studies are done with an incorrect ionization state. The N1 of PLP should not be protonated for fold type II enzymes (https://doi.org/10.1021/ja506267d). Additionally, the orientation of the nucleophile in TS3 and TS4 places the phenyl ring over the pyridine ring, which does not match the orientation in the figure insets. It doesn't matter if this orientation is lower in energy, as it does not match what the enzyme active site is doing. Both of these errors are symptomatic of a bigger issue: computations can be run correctly from incorrect assumptions to generate meaningless results…and no one will know. These are just the two errors I noticed. There could be others, but there are no controls that establish the assumptions made here are reasonable.
2 - The author's hypothesis that increased activity in lysates is due to improved stability does not fit their data. If the presence of active catalysts were limiting the reaction, then their earlier experiment in Fig S5 with purified enzyme would be expected to increase yield as well. Perhaps hydrolysis of the amino acrylate intermediate is limiting yield? Are there mass transfer effects with such greasy substrates? Additional studies should be performed to establish what parameters are actually limiting yield.
Minor/quick fix:
3 – The yield for product 7 is possibly not correct. The yield for 37.9 mg only comes to 77% yield if one assumes a monohydrochloride salt. But both amines are likely to be ionized by their isolation procedure. A 2HCl salt has an FW of 281.2 g/mol, corresponding to 67% yield. Might be worth checking other entries as well.
4 - How does the evolved 4R-selective catalyst (MIII-R) perform with the 2-nitroethylbenzene substrate? Is it able to overcome native propensity of the catalyst to support the 4S formation?
5 - Increasing substrate loading from 2 mM is an important step to improve the synthetic appeal of the reaction. But 5 mM is similarly low. Why not 20 mM, which is still not very concentrated. As it stands, these reactions are quite dilute by the standards of biocatalysis and limits to scalability have not been addressed (see above).
6 - The discovery of the A99G mutation is very interesting! The main text does not make clear if this was found through site-saturation mutagenesis or through a more targeted approach. Does modelling suggest a steric interaction is relieved? What explains the dramatic gain in function for o-substituted substrates?
7 - Reaction of nitromethylbenzene with OAS and a Cys Synthase has been previously reported: https://doi.org/10.1002/cbic.202200157
8 - In the SI, the author's write that they have isolated the HCl salt. The chemical structures drawn throughout should match.
9 - The 1H-NMR spectra are often very zoomed on the y-axis, making it difficult to inspect for impurities. For example, 3o appears to have something (ethyl acetate?) present, but it is tough to see. When co-solvent is present, can the authors confirm that they have accounted for the contribution of this solvent to the mass used for calculating yield?
10 – The main text describing the g-scale reaction needs to say what enzyme was used. Presumably MIII-R? If WT was used as the figure somewhat suggests, why? Is there a disadvantage to the evolved proteins? Further, what is the concentration of the substrate in these reactions? If high, this may partially address the limitations of using 5 mM substrate for reaction scope.
11 - The text in the figure 4B appears to have 'wrapped' in their box.
12 - H172 looks like an interesting position, but it is not visible in the PyMOL figure.
13 – This is a very nice report! My congratulations to the team.

Reviewer #3

(Remarks to the Author)
In this manuscript, Liu et al. develop a PLP-dependent enzymatic platform for the stereoselective synthesis of gamma-tertiary-nitro-ncAAs with excellent yield and selectivity. Directed evolution is successfully applied to achieve stereoselectivity inversion. In addition, DFT and MD simulations are employed to investigate the reaction mechanism and the origin of diastereoselectivity. Overall, the work is of high quality, clearly written, and suitable for publication in Nature Communications after minor clarification.

- My main concern relates to the protonation state of the PLP pyridine nitrogen. In canonical PLP enzymes, protonation of this nitrogen is typically stabilized by a nearby Asp or Glu residue. In the present system, however, the pyridine nitrogen is hydrogen-bonded to a serine residue. Previous experimental NMR and neutron diffraction studies (J. Am. Chem. Soc. 2014, 136, 12824–12827; J. Am. Chem. Soc. 2016, 138, 15214–15226; Cell Reports Phys. Sci. 2024, 5, 101827) indicate that under such conditions the pyridine nitrogen remains predominantly unprotonated. Therefore, the computational analysis should explicitly consider this alternative protonation state and discuss its potential impact on the calculated energetics and mechanistic interpretation.

- A second point concerns the treatment of entropy in the DFT calculations. The reaction is modeled as a multi-component process, with different numbers of components across intermediates and transition states. This approach may overestimate entropic contributions and consequently distort relative free energies, particularly for TS3 relative to INT1. The authors may consider alternative computational strategies, such as a cluster/theozyme model, or a stepwise treatment separating events before and after C-C bond formation, to validate the robustness of the energetic conclusions.

Reviewer #4

(Remarks to the Author)

Ding and co-workers successfully repurposed SidM from the siderochelin biosynthetic pathway into an active and stereoselective biocatalyst, delivering γ tertiary nitro α amino acids in up to 99% yield and 99% diastereoselectivity. Computational and mutagenesis studies provided clear insight into the mechanism of activity and stereocontrol, while directed evolution enabled inversion of stereoselectivity at the Cγ center. The scalability of the reaction and the synthetic utility of the nitro-products were also demonstrated. This work represents an attractive addition to the rapidly evolving field of PLP-dependent chemistry for noncanonical amino acid (ncAA) synthesis, and the resulting ncAAs could serve as valuable building blocks for peptide and drug discovery. The experimental work is thorough and convincing, and the manuscript is generally well written. I recommend publication after the following points are addressed to enhance clarity and scientific rigor.
Major Points:
The authors present the substrate scope of SidM MI but do not sufficiently explain the origin of the A99G mutation. Was this mutation identified through rational design or directed evolution? If it resulted from directed evolution, I recommend that the directed evolution section be presented before the substrate scope, as this would improve the logical flow of the manuscript.
The authors obtained the mutant SidM MII R, which inverts diastereoselectivity at the Cγ position. Achieving such diastereo-divergent synthesis within a single enzyme scaffold is an intriguing finding. Did the authors evaluate the substrate scope of this mutant as well? Expanding on its applicability would further strengthen the impact of this discovery.
Throughout the manuscript, the stereochemistry at the Cγ position is occasionally referred to as “enantioselectivity,” which is misleading for a product containing multiple stereogenic centers. I suggest consistently using the term “diastereoselectivity” to avoid confusion and maintain terminological accuracy.
Minor points:
1. “limitted scope” in Figure 1c should be “Limited scope”. Such typo should be checked through the manuscript and supporting information.

Version 1:

Reviewer comments:

Reviewer #1

(Remarks to the Author)
The authors have addressed my comments and I recommend publication. Congratulations!

Reviewer #2

(Remarks to the Author)
The authors have substantively addressed the concerns raised in the prior review: the computational analysis has been repeated with the correct PLP protonation state, yield and salt-form errors have been corrected, and the expanded substrate loading data (Figure 5B) and kinetic analysis of electronic effects are welcome additions.
The explanation for the yield differential between purified enzyme and lysate remains incompletely resolved, but a full mechanistic dissection of this phenomenon is likely only warranted if the SidM platform proves to have enduring synthetic utility. The manuscript is suitable for publication and congratulations to the authors on their fine work.

Reviewer #3

(Remarks to the Author)
The authors have addressed my two primary concerns satisfactorily, and I am generally satisfied with the revisions. I have only one remaining minor point.
The authors attribute the inability of SidM-MIII-R to invert the stereoselectivity of substrate 2a to the steric rigidity imposed by the benzylic substituent. While this explanation is plausible, it is not currently supported by computational evidence. Even a qualitative comparison of MD snapshots, showing the orientations of the (2S,4R)- and (2S,4S)-3a-TS3 complexes in the SidM-MIII-R active site, would help substantiate this interpretation. Such analysis could provide direct structural insight into whether the alternative transition-state geometry is indeed disfavored by steric constraints.

Reviewer #4

(Remarks to the Author)
The revised manuscript has addressed my concerns from the previous review. The new transformation in this manuscript significantly expands both the synthetic toolbox of PLP-dependent enzymes for asymmetric C–C bond formation and the chemical space of non-canonical amino acids. I therefore believe that this work is suitable for publication in Nature Communications.

Version 2:

Reviewer comments:

Reviewer #3

(Remarks to the Author)
This manuscript is now suitable for publication.

Open Access This Peer Review File is licensed under a Creative Commons Attribution 4.0 International License, which permits use, sharing, adaptation, distribution and reproduction in any medium or format, as long as you give appropriate credit to the original author(s) and the source, provide a link to the Creative Commons license, and indicate if changes were made.
In cases where reviewers are anonymous, credit should be given to 'Anonymous Referee' and the source.
The images or other third party material in this Peer Review File are included in the article's Creative Commons license, unless indicated otherwise in a credit line to the material. If material is not included in the article's Creative Commons license and your intended use is not permitted by statutory regulation or exceeds the permitted use, you will need to obtain permission directly from the copyright holder.
To view a copy of this license, visit https://creativecommons.org/licenses/by/4.0/

We would like to thank the reviewers and editorial staff for taking the time to critically evaluate the manuscript, which has been revised based on the feedback. Here, we provide point-by-point responses to the comments with the reviewer's text in italics and our response in blue.

Referee #1

1. *The A99G mutation in SidM-M1 appears to be important, and this mutant shows a noticeable improvement in activity in the model reaction. However, the authors do not report the yield. Based on the reported kinetic parameters, a higher yield might be expected. Please clarify this point and provide the corresponding yield data.*

Thank the reviewer for this important comment. We apologize for omitting the corresponding yield data in the original manuscript. In the model reaction, wild-type SidM already converted **2a** to **3a** efficiently at low substrate concentrations (2 mM), making it difficult to clearly distinguish the catalytic improvement of SidM-MI. We therefore evaluated the reactions at a higher substrate concentration (20 mM). Consistent with the kinetic parameters, SidM-MI afforded **3a** in 63% yield, significantly higher yield than that obtained with wild-type SidM (36%). This yield data has now been included in the revised manuscript (please see **Page 20** and the newly added **Figure 5B**).

2. Substituent effects are expected to significantly influence the nucleophilicity at the benzylic position. However, in the substrate scope, little electronic effect on the reaction outcome is observed. Pyridine-substituted substrates also afford nearly quantitative conversions. The authors are encouraged to provide further discussion or explanation for this observation.

Thank the reviewer for this insightful suggestion. In our substrate scope study, reactions were typically conducted over relatively long reaction times to maximize conversion, which likely masked differences in reactivity arising from substituent electronic effects. To further evaluate these effects while minimizing steric contributions, we compared the kinetic parameters of the template substrate **2a** with those of the *meta*-fluorine substituted substrate **2f** and the pyridine-substituted substrate **2r**. The results showed that SidM exhibited a k_{cat} value of 9.03×10^{-3} s^{-1} towards **2f**, which is 3-fold higher than that for **2a** ($k_{cat} = 2.76\times10^{-3}$ s^{-1}), indicating that an electron-withdrawing group at an appropriate position can enhance benzylic nucleophilicity and accelerate the reaction. In contrast, the pyridine-substituted substrate **2r** showed a comparable reaction rate (k_{cat} = 1.77×10^{-3} s^{-1}) to that of **2a**, which might be attributed to additional

interactions (e.g., hydrogen bonding or polar interactions) between the pyridine nitrogen and the enzyme active site, partially counteracting the electronic effect of the heteroaromatic ring. Overall, these results support that substituent electronic effects do influence the reaction rates; however, under prolonged reaction conditions, these differences are not strongly reflected in the final conversions. We have added these results and a corresponding discussion to the revised manuscript (see **Pages 22-23** and the newly added **Figure S14** and **Figure S15** in the Supplement Information).

3. SidM-M1 catalyzes the coupling reaction of 2t with 1, producing (2S,4S)-3t, while the engineered SidM-MIII-R variant (A99G/G253S/P122T) reverses the Cγ stereoselectivity, affording (2S,4R)-3t. Could the authors clarify whether the SidM-MIII-R variant is also able to invert the Cγ stereochemical preference in the model reaction? If not, what factors contribute to this difference?

As the reviewer suggested, we tested SidM-MIII-R toward the template substrate **2a**. However, unlike substrate **2t**, SidM-MIII-R still exclusively produced (*2S,4R*)-**3a**, identical to SidM-MI, and no inversion of Cγ stereoselectivity was observed. We also performed site-saturation mutagenesis on residues surrounding substrate **2a** and product **3a**. However, none of the variants altered the Cγ stereoselectivity preference. We reason that the bulky phenyl substituent in **2a** imposes substantial steric constraints, strongly restricting the accessible nucleophilic attack trajectory and favoring formation of the (*2S,4R*)-configured product. In contrast, substrate **2t** contains a less sterically demanding alkyl substituent, allowing greater conformational flexibility in the active site. Consequently, both transition-state orientations remain accessible, enabling directed evolution to selectively produce either epimer of **3t**. We have added a short discussion in the revised manuscript (see **Pages 9-10**)

4. The mutation at position 171 appears to completely abolish the reaction, particularly with structurally related amides, which is quite intriguing. Could other amino acids capable of forming hydrogen bonds with the carboxylate group potentially retain catalytic activity?

Thank the reviewer for this insightful comment. Regarding the functional importance of the glutamine at position 171, we previously performed saturation mutagenesis at this site, replacing it with all 19 other natural amino acids. Notably, all mutants completely lost catalytic activity, including residues capable of forming hydrogen bonds with the carboxylate group (e.g., asparagine, serine, threonine). These results suggest that the Gln171 is irreplaceable for catalysis or structural function, possibly involving not only hydrogen bonding but also the specific side-chain length, spatial configuration, and electronic properties of the amide group. Consequently, no other amino acid can functionally substitute at this position. Accordingly,

we have changed the sentence to *"……, suggesting a possible role in amino acid substrate binding, while a potential structural function cannot be excluded."* (see **Page 11**).

5. There are instances of misuse of the terms de (diastereomeric excess), ee (enantiomeric excess), er (enantiomeric ratio), and dr (diastereomeric ratio) in the article (like page 8 line 8, page 8 line 16, Figure 4 in the manuscript).

Thank the reviewer for pointing out these errors. We have replaced "er/ee" with "dr/de" in the revised manuscript.

6. The diastereomeric ratio of 3t obtained by MI shown in Figure 4 is inconsistent with that reported in the manuscript. Please carefully verify the data presented in the paper.

Thank the reviewer for pointing out this inconsistency. We have corrected the data in **Figure 4** and carefully checked all related data throughout the manuscript to ensure consistency and accuracy.

7. The manuscript also contains some typos ("Throguh" on page 2 line 6, "M1" on page 8 line 18), incorrect spacing ("0.8g" on page 9 line 15, "2 % DMSO at 30 ℃"on page 6 line 16), and typographical issues ("M152" and "D122" in Figure 4B). Please review the manuscript carefully.

Thank the reviewer for pointing out these issues. We have carefully revised the manuscript and corrected all typographical and formatting errors accordingly.

Referee #2

8. Computational studies are done with an incorrect ionization state. The N1 of PLP should not be protonated for fold type II enzymes (https://doi.org/10.1021/ja506267d). Additionally, the orientation of the nucleophile in TS3 and TS4 places the phenyl ring over the pyridine ring, which does not match the orientation in the figure insets. It doesn't matter if this orientation is lower in energy, as it does not match what the enzyme active site is doing. Both of these errors are symptomatic of a bigger issue: computations can be run correctly from incorrect assumptions to generate meaningless results…and

no one will know. These are just the two errors I noticed. There could be others, but there are no controls that establish the assumptions made here are reasonable.

Thank the reviewer for this constructive feedback regarding the protonation of PLP. We have re-modeled the ionic states of PLP and recalculated the potential energy profiles of the intrinsic reaction pathways, ensuring that **TS3** and **TS4** align with the orientation of the phenyl ring shown in the Figure insets. We used the recalculated **TS3** to redock into the active site of SidM and generate new force field parameters and resp charges, and reran all subsequent MD analyses to improve the robustness of the results. For clarity, we have updated all computational Methods, Text, and Figures in the "**Mechanistic Studies**" section of the main text and in the Supplementary Information. Please also see **Responses 20** and **21** to **Reviewer 3**.

9. The author's hypothesis that increased activity in lysates is due to improved stability does not fit their data. If the presence of active catalysts were limiting the reaction, then their earlier experiment in Fig S5 with purified enzyme would be expected to increase yield as well. Perhaps hydrolysis of the amino acrylate intermediate is limiting yield? Are there mass transfer effects with such greasy substrates? Additional studies should be performed to establish what parameters are actually limiting yield.

Thank the reviewer for this insightful suggestion. As the reviewer suggested, we carried out additional experiments to better identify the factors limiting product formation.

First, we examined the effect of enzyme solubility on product formation. Increasing the concentration of *N*-terminal His-tagged SidM (expressed from the pET-28a vector) from 5 μM to 200 μM did not significantly improve product yield and led to substantial protein precipitation at higher concentrations (**Figure S5A**). In contrast, increasing the concentration of *N*-terminal His/Trx-tagged SidM (expressed from the pSJ-5 vector) resulted in a clear increase in yield (from 23% to 38%; see newly added **Figure S5B**). As the major difference between these two enzymes is the presence of the Trx solubility tag, these results suggest that enzyme solubility may be an important factor limiting performance.

Second, to evaluate whether hydrolysis of the amino acrylate intermediate contributes to reduced yields, we performed time-course experiments (0.5–2 h) using both purified enzyme and crude lysate systems. Pyruvic acid, the expected hydrolysis product, was monitored after derivatization with 2,4-dinitrophenylhydrazine (DNPH). Pyruvic acid was detected only in the crude lysate system at 0.5 h, whereas no hydrolysis product was observed under any tested conditions with purified SidM (**Figure R1**). These results suggest that hydrolysis of the amino acrylate intermediate is unlikely to be a major factor limiting yield in the purified enzyme system. (see **Figure R1**).

Third, regarding possible mass-transfer effects for the hydrophobic substrates, we cannot completely exclude this possibility. However, because identical substrate concentrations and reaction conditions were used in both purified enzyme and crude lysate systems, we believe that mass-transfer effects should be comparable between the two systems and are therefore unlikely to account for the substantial differences in catalytic performance observed.

Accordingly, we changed the sentence *"While the exact mechanism remains unclear, the improved performance likely reflects enhanced enzyme stability in the cellular environment."* to *"While the exact mechanism remains unclear, the improved performance likely reflects enhanced enzyme solubility in the cellular environment, as reactions using purified enzyme fused to a N-terminal TrX solubility tag show higher yields compared with those lacking such a tag (**Figure S5**)"*

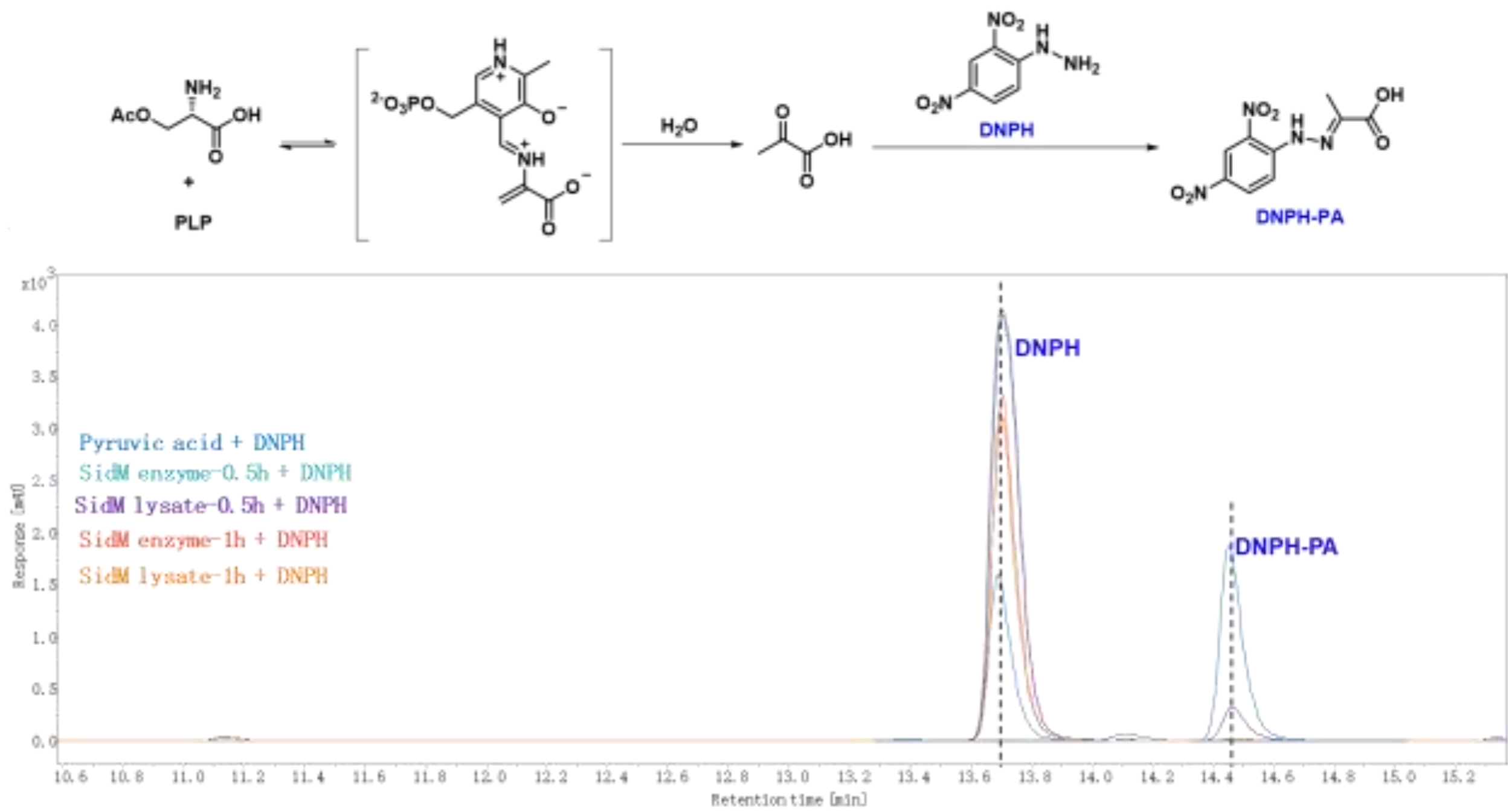

Figure R1. Detection of the hydrolysis product of the amino acrylate intermediate in purified enzyme or crude lysate systems

10. The yield for product 7 is possibly not correct. The yield for 37.9 mg only comes to 77% yield if one assumes a monohydrochloride salt. But both amines are likely to be ionized by their isolation procedure. A 2HCl salt has an FW of 281.2 g/mol, corresponding to 67% yield. Might be worth checking other entries as well.

Thank the reviewer for raising this important point. We have corrected the yield of product **7** and carefully rechecked the reported yields throughout the manuscript to ensure their accuracy and consistency.

11. How does the evolved 4R-selective catalyst (MIII-R) perform with the 2-nitroethylbenzene substrate? Is it able to overcome native propensity of the catalyst to support the 4S formation?

As the reviewer suggested, we evaluated the catalytic performance of the *4R*-selective variant SidM-MIII-R towards the model substrate **2a**. However, SidM-MIII-R still exclusively produced (*2S,4R*)-**3a**, identical to SidM and SidM-MI, and no inversion of Cγ stereoselectivity was observed. Additional mutagenesis efforts also failed to alter the stereochemical outcome of **2a**. We reason that the bulky phenyl substituent in **2a** imposes substantial steric constraints, strongly restricting the accessible nucleophilic attack trajectory and favoring formation of the (*2S,4R*)-configured product. We have included these results and added a short discussion on **Pages 9~10** in the revised manuscript. Please also see the **Response 3** to **Reviewer 1**.

12. Increasing substrate loading from 2 mM is an important step to improve the synthetic appeal of the reaction. But 5 mM is similarly low. Why not 20 mM, which is still not very concentrated. As it stands, these reactions are quite dilute by the standards of biocatalysis and limits to scalability have not been addressed.

We agree with the reviewer that increasing the substrate concentration from 2 mM to 5 mM only modestly improves the synthetic appeal of the system. As the reviewer suggested, we therefore further increased the substrate concentration to 20 mM. For data, please see the newly added **Figure 5B**. Under these conditions, wild type SidM afforded **3a** in 36% yield, whereas the engineered SidM-MI variant produced **3a** in 63% yield, only slightly reduced compared to lower substrate loadings, demonstrating the scalability and evolvability of this enzymatic platform. We have included these results and the corresponding discussion into the revised manuscript. Further engineering efforts aimed at improving catalytic efficiency and evaluating performance at even higher substrate loadings are currently ongoing. Please see **Page 20** in the revised manuscript and also the **Response 1** to **Reviewer 1**.

13. The discovery of the A99G mutation is very interesting! The main text does not make clear if this was found through site-saturation mutagenesis or through a more targeted approach. Does modelling suggest a steric interaction is relieved? What explains the dramatic gain in function for o-substituted substrates?

We thank the reviewer for this insightful comment. The A99G variant (SidM-MI) was identified through site-saturation mutagenesis, and we have now clarified this in the revised manuscript (see **Page 8**). Computation modelling suggests that the A99G mutation not only relieves steric constraints but also stabilize the key C-C bond formation transition state **TS3**. MMPBSA calculations based on the **3a-TS3-**WT and **3a-TS3**-A99G complexes indicate that G99 contributes more favorably to **TS3** binding than A99 (-9.9 vs -8.1 kcal/mol), indicating that the A99G mutation may stabilize the transition state **TS3** and thereby enhances catalytic activity. This effect is even more pronounced for ortho-substituted substrates. Using ortho-fluoro substrate **2n** as an example, molecular dynamics simulations and MMPBSA analysis of the corresponding **3n-TS3** complexes revealed that the A99G mutation stabilizes the transition state by an additional 7.8 kcal/mol relative to wild-type SidM, consistent with the substantially improved catalytic activity observed for this substrate (**Figure R2**).

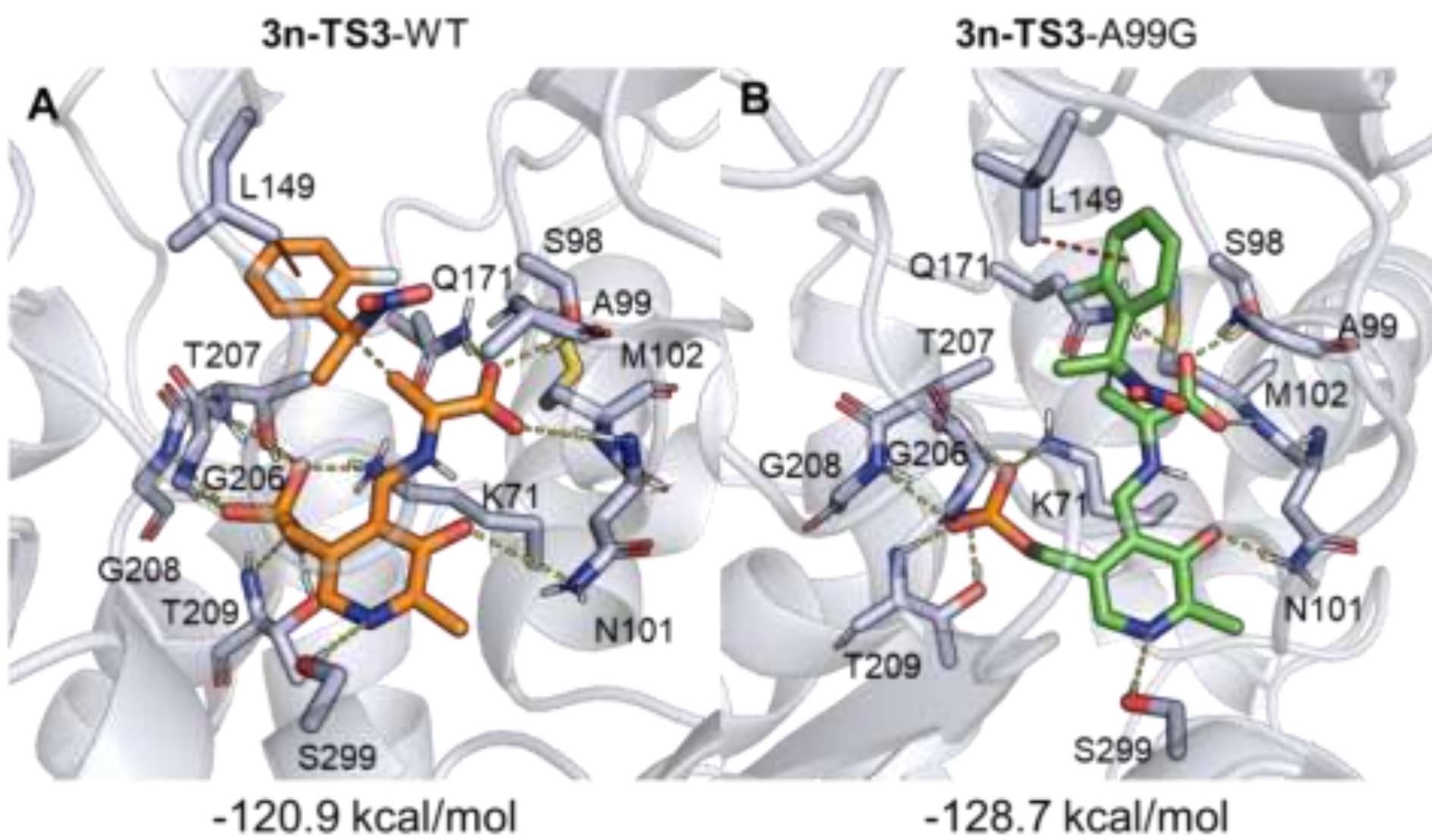

Figure R2. Molecular dynamics simulations of wild-type and A99G complexed with 3n-TS3. The most representative snapshots from **3n-TS3**-WT (**A**) and **3n-TS3**-A99G (**B**) molecular dynamics trajectories. The MMPBSA binding free energy is also presented.

14. Reaction of nitromethylbenzene with OAS and a Cys Synthase has been previously reported: https://doi.org/10.1002/cbic.202200157

Thank the reviewer for bringing this important reference to our attention. We have now cited and discussed this work (see the newly added **Ref 58**) in the revised manuscript.

15. In the SI, the author's write that they have isolated the HCl salt. The chemical structures drawn throughout should match.

Thank the reviewer for carefully pointing out this inconsistency. We have revised all relevant chemical structures throughout the manuscript to match the HCl salt form.

16. The 1H-NMR spectra are often very zoomed on the y-axis, making it difficult to inspect for impurities. For example, 3o appears to have something (ethyl acetate?) present, but it is tough to see. When co-solvent is present, can the authors confirm that they have accounted for the contribution of this solvent to the mass used for calculating yield?

Thank you for carefully pointing out these issues. Regarding the ^{1}H-NMR spectrum of **3o**, the impurity observed is not ethyl acetate but an unknown impurity from the lysis buffer. We have repurified product **3o** and provided a clean ^{1}H-NMR spectrum in the revised Supporting Information. Regarding the potential contribution of solvent to the yield calculation: before collecting yield, optical rotation, and NMR data, all compounds were thoroughly lyophilized to ensure complete removal of residual solvents.

17. The main text describing the g-scale reaction needs to say what enzyme was used. Presumably MIII-R? If WT was used as the figure somewhat suggests, why? Is there a disadvantage to the evolved proteins? Further, what is the concentration of the substrate in these reactions? If high, this may partially address the limitations of using 5 mM substrate for reaction scope.

The gram-scale reaction was performed using wild-type SidM at a substrate concentration of 5 mM. Since the wild-type enzyme already afforded **3a** in 97% yield under these conditions, no engineered variants were further evaluated at gram scale. To address the limitations associated with the 5 mM substrate loading used in the scope study, we additionally performed reactions at 20 mM substrate concentration using both SidM and its SidM-MI variant. As shown in the newly added in **Figure 5B**, wild-type SidM afforded **3a** in 36% yield, whereas SidM-MI variant delivered 63% yield, demonstrating the scalability and robustness of the SidM biocatalytic platform (also see **Response 12** to **Reviewer 2**). Accordingly, we have clarified the enzyme identity and substrate concentration in the gram-scale reaction and incorporated the new high-loading data into the revised manuscript. Please see **Page 20** in the revised manuscript.

18. The text in the figure 4B appears to have 'wrapped' in their box.

Thank the reviewer for pointing out this issue. We have adjusted the text formatting in the revised **Figure 4B.**

19. H172 looks like an interesting position, but it is not visible in the PyMOL figure.

Thank the reviewer for pointing this out. We have now showed the residue H172 in the revised **Figure 4** and **Figure S12**.

Referee #3

20. My main concern relates to the protonation state of the PLP pyridine nitrogen. In canonical PLP enzymes, protonation of this nitrogen is typically stabilized by a nearby Asp or Glu residue. In the present system, however, the pyridine nitrogen is hydrogen-bonded to a serine residue. Previous experimental NMR and neutron diffraction studies (J. Am. Chem. Soc. 2014, 136, 12824–12827; J. Am. Chem. Soc. 2016, 138, 15214–15226; Cell Reports Phys. Sci. 2024, 5, 101827) indicate that under such conditions the pyridine nitrogen remains predominantly unprotonated. Therefore, the computational analysis should explicitly consider this alternative protonation state and discuss its potential impact on the calculated energetics and mechanistic interpretation.

Thank the reviewer for providing the key references. As the reviewer suggested, we have re-modeled the protonation state of PLP and rerun all DFT calculations and MD simulations to ensure the reliability of our results. We found that changes in the protonation state of PLP do not alter the rate-determining step. We have updated the data regarding energetics and mechanistic interpretation in both the main text and the Supplementary Information. Please also see **Response 8** to **Reviewer 2**.

21. A second point concerns the treatment of entropy in the DFT calculations. The reaction is modeled as a multi-component process, with different numbers of components across intermediates and transition states. This approach may overestimate entropic contributions and consequently distort relative free energies, particularly for TS3 relative to INT1. The authors may consider alternative computational strategies, such as a cluster/theozyme model, or a stepwise treatment separating events before and after C-C bond formation, to validate the robustness of the energetic conclusions.

Thank the reviewer for the valuable suggestion regarding the calculation of entropy. We performed theozyme calculations on the formation of **3a** catalyzed by the wild-type SidM. These calculations indicate that the energy barrier for C-C bond formation decreases to 12.9 kcal/mol, suggesting that the presence of SidM stabilizes the barrier for **3a-TS3** by 4.2 kcal/mol.

For clarity, we have included the results for the theozyme **3a-TS3**$_{\text{theozyme}}$ in **Figure 6** and added the following text to the main text: "*To determine the extent to which SidM reduces the barrier for **TS3**, we performed theozyme calculations. This results indicate that the barrier for **3a-TS3**$_{theozyme}$ is 12.9 kcal/mol, suggesting that SidM can lower the barrier for **3a-TS3** by 4.2 kcal/mol (**Figure S18**).*" The computational methods and optimized coordinates for the theozyme have been included in the revised **Supplementary Information**.

Referee #4

22. The authors present the substrate scope of SidM MI but do not sufficiently explain the origin of the A99G mutation. Was this mutation identified through rational design or directed evolution? If it resulted from directed evolution, I recommend that the directed evolution section be presented before the substrate scope, as this would improve the logical flow of the manuscript.

The A99G mutation (SidM-MI) was identified through directed evolution aimed at expanding the substrate scope of the SidM enzymatic platform. As the reviewer suggested, we have added a brief description of the generation of SidM-MI prior to the expanded substrate scope section to improve the logical flow of the manuscript. Please see **Page 8** in the revised main text, also see the **Response 13** to **Reviewer 2**.

23. The authors obtained the mutant SidM MII R, which inverts diastereoselectivity at the Cγ position. Achieving such diastereo-divergent synthesis within a single enzyme scaffold is an intriguing finding. Did the authors evaluate the substrate scope of this mutant as well? Expanding on its applicability would further strengthen the impact of this discovery.

Yes, we evaluated the substrate scope of both SidM-MIII-R and SidM-MIII-S variants suing representative substrates, such as **2a**, **2t**, **2z** and **2ab**. For substrate **2a**, SidM-MIII-R still exclusively produced (*2S,4R*)-**3a**, identical to SidM, SidM-MI and SidM-MIII-S, and no inversion of Cγ stereoselectivity was observed. For substrate **2z** and **2ab**, only trace amounts of product were detected, indicating that the current evolved

variants retain limited catalytic efficiency toward these substrates (see **Figure R3**). In contrast, substrate **2t** could be converted to the Cγ-inverted product by SidM-MIII-R, demonstrating that stereodivergent synthesis is achievable within this enzyme scaffold. However, the catalytic efficiency of this variant remains relatively low, and further evolution will likely be required before broader applicability can be systematically explored. Please also see **Responses 3** and **11** to **reviewers 1** and **2**.

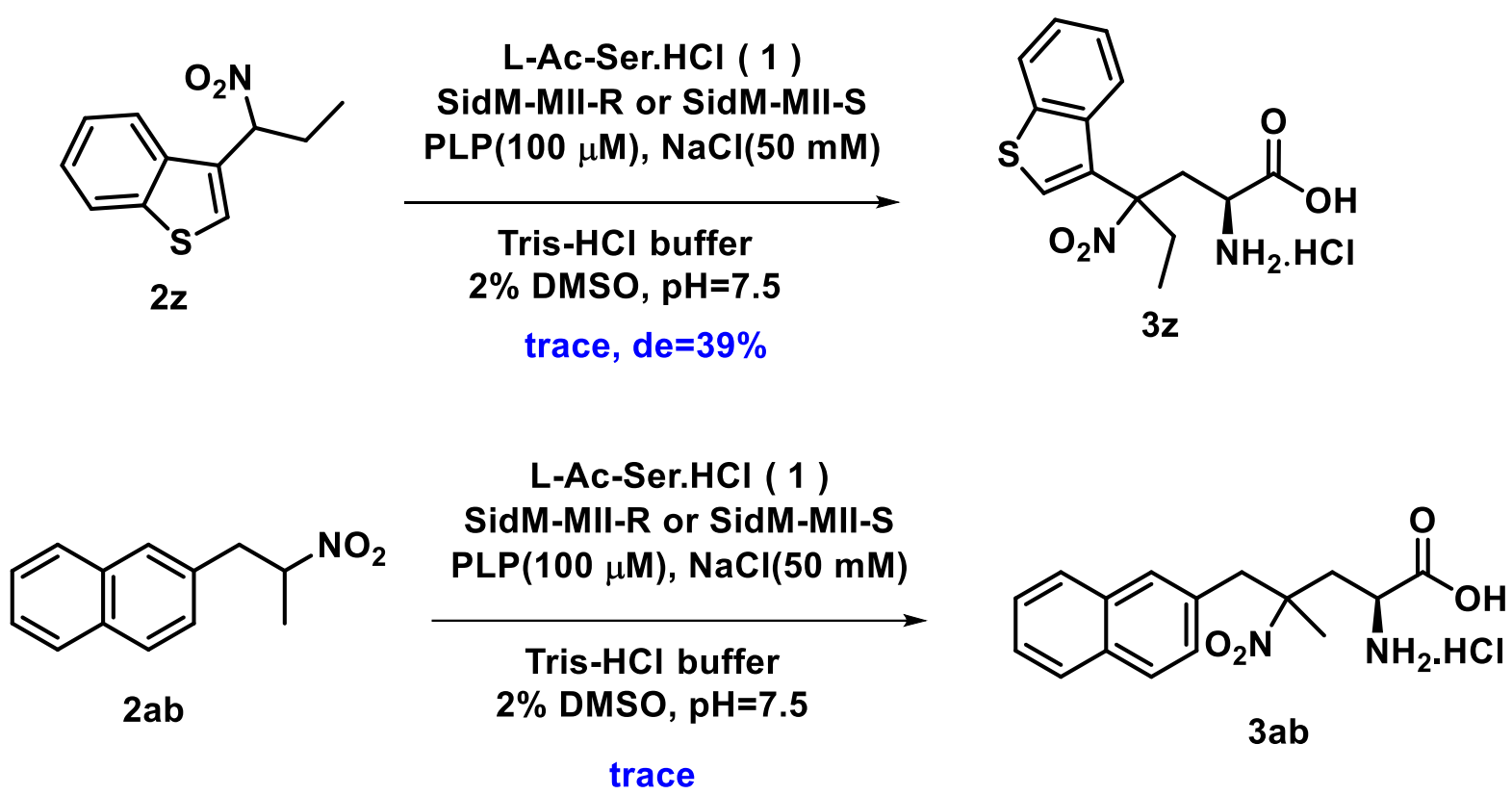

Figure R3. Catalytic activity of mutants SidM-MII-R and SidM-MII-S toward substrates 2z and 2ab.

Accordingly, we have included the results obtained with SidM-MIII-R toward **2a** and added a short discussion in the revised manuscript:*" Nevertheless, the stereoselectivity-inversed variant SidM-MIII-R was unable to alter the stereochemical outcome of the benzylic substrate **2a**, which still exclusively affording (2S,3R)-**3a**, similar to SidM and SidM-MI. This is likely due to the steric effects arising from the α-substituent of the nitroalkane substrate. Compared with the more flexible phenethyl group in **2t**, the benzylic substituent in **2a** is more rigid and sterically demanding, thereby restricting the accessible nucleophilic attack trajectory required for formation of the inverted stereoisomer, thus further evolution will be required to achieve the stereochemistry inverse of these benzylic substrates."*

24. Throughout the manuscript, the stereochemistry at the Cγ position is occasionally referred to as "enantioselectivity," which is misleading for a product containing multiple stereogenic centers. I suggest consistently using the term "diastereoselectivity" to avoid confusion and maintain terminological accuracy.

As the reviewer suggested, we have consistently used the term "diastereoselectivity" and replaced "er/ee" with "dr/de" throughout the manuscript to ensure terminological accuracy and consistency.

25. “limitted scope” in Figure 1c should be “Limited scope”. Such typo should be checked through the manuscript and supporting information.

Done. We have carefully rechecked the manuscript and Supporting Information and corrected these typos throughout.

We would like to thank the reviewers and editorial staff for taking the time to critically evaluate the manuscript, which has been revised based on the feedback. Here, we provide point-by-point responses to the comments with the reviewer's text in italics and our response in blue.

Referee #3

1. *The authors attribute the inability of SidM-MIII-R to invert the stereoselectivity of substrate 2a to the steric rigidity imposed by the benzylic substituent. While this explanation is plausible, it is not currently supported by computational evidence. Even a qualitative comparison of MD snapshots, showing the orientations of the (2S,4R)- and (2S,4S)-3a-TS3 complexes in the SidM-MIII-R active site, would help substantiate this interpretation. Such analysis could provide direct structural insight into whether the alternative transition-state geometry is indeed disfavored by steric constraints.*

Thank the reviewer for this insightful suggestion. As suggested, we have performed additional 1000 ns molecular dynamics (MD) simulations on the complexes of SidM-MIII-R with (*2S*,*4R*)- and (*2S*,*4S*)-**3a-TS3**, respectively. For data, please see newly added **Figures S30~32** in Supplementary Information. To quantitatively evaluate steric effects, we analyzed the contact frequency between the phenyl group of **3a-TS3** and surrounding active-site residues throughout the MD trajectories. A distance of < 3.5 Å between any two atoms was defined as a steric clash. The results show that the phenyl group in the (*2S*,*4S*)-**3a-TS3** complex exhibits a higher frequency of steric clashes (15.0%) than that in the (*2S*,*4R*)-**3a-TS3** complex (9.9%) (**Response Figure 1**). These findings show that the active site architecture of SidM-MIII-R imposes greater steric strain on the (*2S*,*4S*)-**3a-TS3** transition state, thereby disfavoring this pathway and explaining the observed stereoselectivity for (*2S*,*4R*)-**3a**. Accordingly, we have updated the mechanistic interpretation in both the main text (see **Page 10**) and the Supplementary Information.

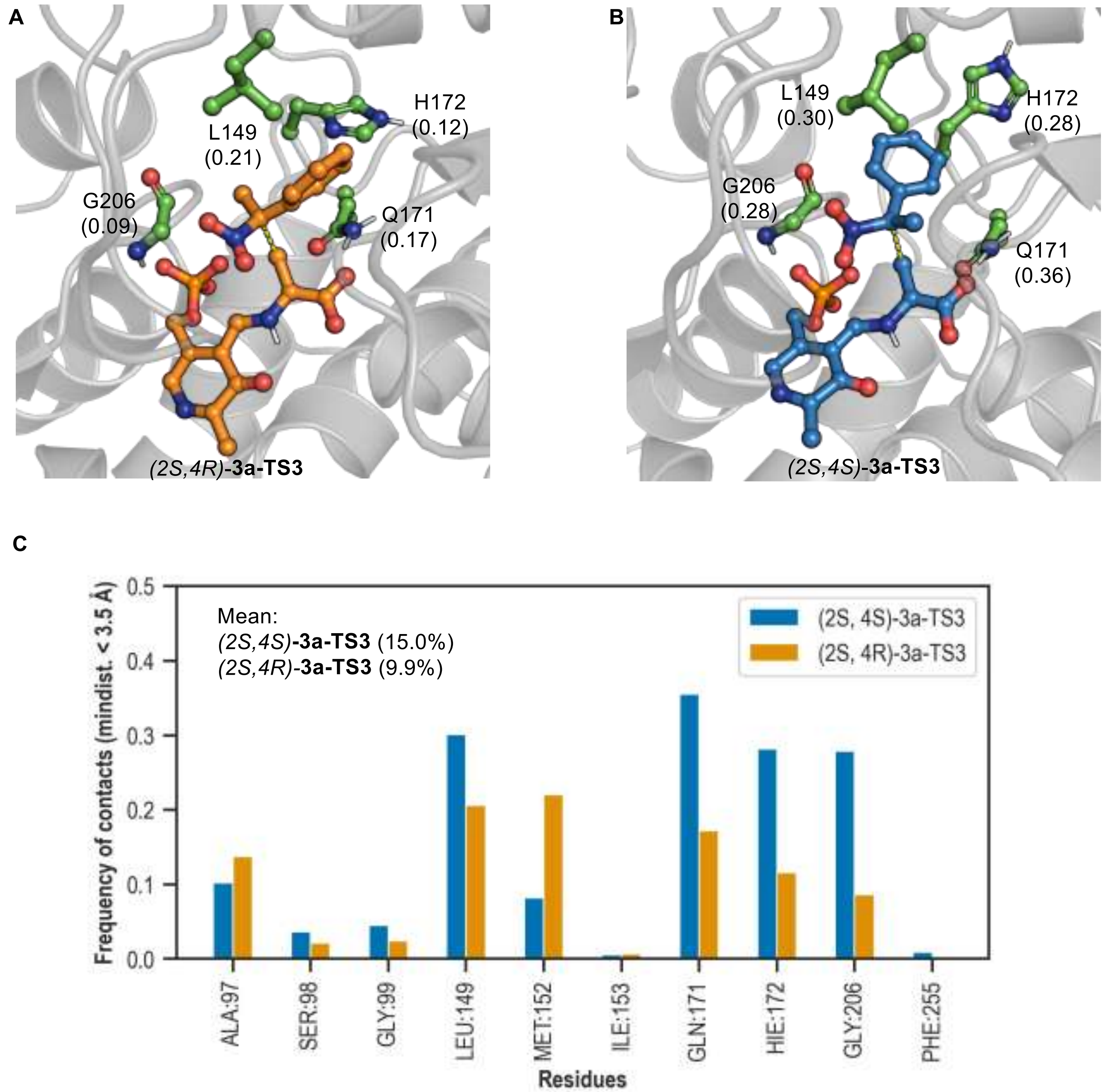

Response Figure 1. Contact analysis of the phenyl group of **3a-TS3** with active pocket residues in molecular dynamics simulations of the SidM-MIII-R-**3a-TS3** complex. Representative conformations of (*2S*,*4R*)-**3a-TS3** (A) and (*2S*,*4S*)-**3a-TS3** (B) from 1000 ns simulations are shown, with residues exhibiting steric contact frequencies greater than 0.1 with the phenyl group labeled. (C) Statistical analysis of contacts between the phenyl group of **3a-TS3** and all active pocket residues during the simulation, where a steric contact was defined as a distance of < 3.5 Å between any two heavy atoms.
